# Geochemistry and X-ray diffraction data from rock salts and saltwork wastes of Canada: data compilation

**DOI:** 10.1016/j.dib.2026.112941

**Published:** 2026-06-06

**Authors:** Cristine Joy Yap, Magnus Roland Marun, Pavel Kabanov, Jaxon Dii Horne, Peter Giles, Paul Durling, Frank Brunton, Tomasz Toboła, Piotr Kukiałka, Susan Johnson

**Affiliations:** a Geological Survey of Canada - Calgary, Natural Resources Canada 3303 33 Street Northwest, Calgary, Alberta T2L 2A7, Canada; b Dept. of Earth, Energy and Environment, University of Calgary, 844 Campus Dr NW, Calgary, Alberta T2L 2A3, Canada; c Geological Survey of Canada - Atlantic, Natural Resources Canada 1 Challenger Drive P.O. Box 1006 Dartmouth, Nova Scotia B2Y 4A2, Canada; d Ontario Geological Survey, Government of Ontario Willet Green Miller Ctr, Level B7, 933 Ramsey Lake Rd Sudbury, Ontario P3E 6B5, Canada; e Faculty of Geology, Geophysics & Environmental Protection, Akademia Górniczo-Hutnicza w Krakowie University of Science and Technology, 30 Mickiewicz Avenue. 30-059 Krakow, Poland; f Kukiałka Consulting Ltd., 207 Glamorgan Pl SW Calgary, AB T3E 5B9 Canada; g Geological Surveys South, Government of New Brunswick Hugh John Flemming Forestry Centre P. O. Box 6000 Fredericton, New Brunswick E3B 5H1, Canada

**Keywords:** Critical minerals, REE, Geochemistry, Salt, Canadian, Lithium, Economic geology

## Abstract

This report compiles geochemical data from major salt formations and associated beds occurring in the subsurface (∼200–2000 m) of sedimentary basins within onshore Canada. Included are new project data (obtained since 2022), those published in research papers, and non-confidential data from national and provincial databases. Rock materials are primarily diamond-bit cores from wells drilled for oil and gas, salt mines, salt caverns, or potash exploration. Four data categories are reported: (1) lithogeochemistry data are whole-rock analytical results acquired predominantly with 4-acid digestion on ICP instrumentation; (2) brined cores – salt samples put into solution with deionised water and analysed with ICP instrumentation; (3) a small set of real industrial brines from salt/potash mines and salt cavern storage operations. The category (4) includes new semi-quantitative X-ray diffraction (XRD) data made on whole rocks (3 clay-fraction samples also included), as well as insoluble residues from brined samples. The brined core data simulate the chemical composition of brines produced by industrial solution mining; they are reported in NaCl saturated notation. A large portion of lithogeochemistry data comes from non-salt beds associated with salt deposits: silty marls, carbonates (“red beds”), and anhydrites. The range of reported elements varies between datasets. New analyses were performed to assess trace elements from the “Critical Minerals” list (Li, Rb, … REE+Y), along with major elements that make up lithic impurities in salts. This compilation provides data support for ongoing Government of Canada research programs, analytical publications, and is intended for broader industry (solution mining, etc.) and public use.

Specifications Table

**Table:** 

Subject	Earth & Environmental Sciences
Specific subject area	Geochemistry of rock salts and associated evaporitic strata: whole-rock lithogeochemistry with 4-acid digestion; brine aqueous geochemistry; whole-rock X-ray diffraction.
Type of data	Raw, chart, filtered.
Data collection	This report compiles geochemical data from major salt formations and associated evaporitic beds (marls, carbonates, anhydrites) occurring in the subsurface of onshore Canada. Included are previously unpublished (“new”) project data from 780 samples, those published in research papers (554 samples), and non-confidential data from national and provincial databases (1813 samples), totalling 3147 samples. Many samples were analysed several times with different analytical protocols. In total, we report 2997 whole-rock lithogeochemistry data, 172 aqueous geochemistry data from brined salt cores, a small set of real industrial brines and brined tailings sediments (37 samples), 281 bulk-rock XRD data, and 3 clay-fraction XRD data. The brined core data simulate the chemical composition of brines produced by industrial solution mining. New analyses were performed to assess trace elements from the “Critical Minerals” list (Li, Rb,… REYs), along with major elements (Al, Ca, Mg, Fe, K, S…) that make up lithic impurities in salts.
Data source location	Secondary data are available at their specified locations, as listed in the source. Primary (new) data are available through the Sample and Analysis Management System (SAMS) of Natural Resources Canada - Geological Survey of Canada – Calgary.
Data accessibility	Repository name: Mendeley. Data identification number: doi:10.17632/y4gcf884x8.6. Direct URL to data: https://data.mendeley.com/datasets/y4gcf884x8/6. To access data, access the following supplementary files:
	• Appendix A_Whole-rock_lithogeochemistry.xlsx. • Appendix B industrial brines_and_brined_cores_recalculated.xlsx. • Appendix C_Diffractograms of brined core insoluble residues.xlsx. • Appendix D_ Whole-rock X-ray diffraction data.xlsx. • Appendix E_Original RTG lab data from diluted brines.xlsx. • Appendix F_Report on field sampling.pdf. • Appendix G_collection of ActLab certificates for new data.zip.
	Original data and information for each sample (columns “GSC curation, C#” in supplementary tables) are also recorded in SAMS. Access to SAMS: Natural Resources Canada - Geological Survey of Canada – Calgary, address: 3303, 33rd St. NW Calgary, AB, T2L 2N2, reachable by telephone at +1–403–292–7000 and by email calgary.commissionaire@nrcan.gc.ca.
Related research article	Kabanov, P., 2025. Clean energy storage potential (hydrogen, CAES) in salt formations of Western Canada: geological review. Geoenergy 3, doi:10.1144/geoenergy2025-005. Kabanov, P., Pinet, N. Brunton, F., Ardakani, O.H., Deblonde, C., Dewing, K.E., Giles, P.S., Durling, P., Henning, M., Arts, A.E., and Utting, N. 2026. Halite formations of Canada for clean energy storage (hydrogen, CAES): A geologic review. Earth Science Reviews, 105,441. doi:10.1016/j.earscirev.2026.105441.

## 1 Value of the Data

**Fig. 1:**
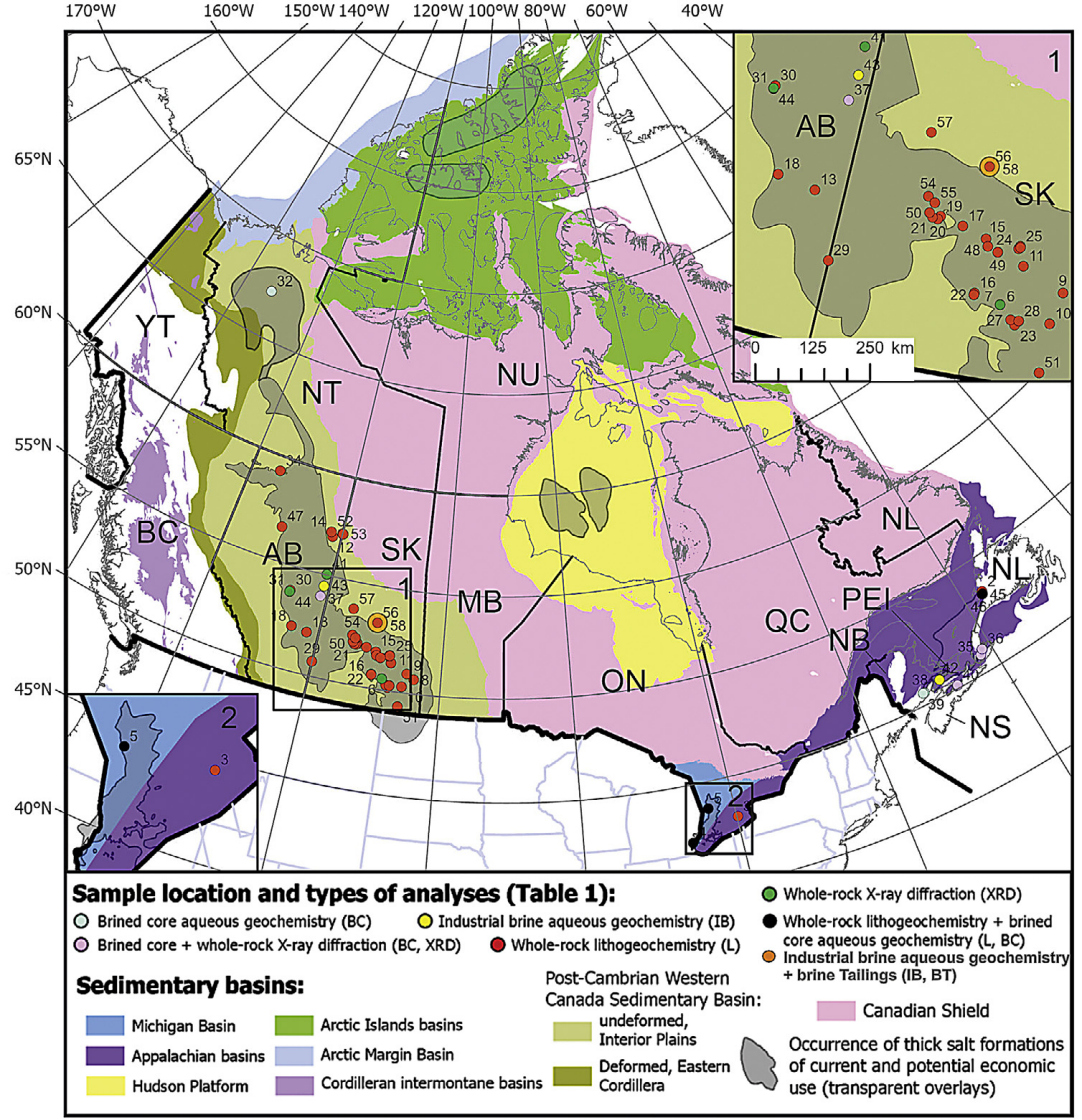
Location of samples on the simplified Map of sedimentary basins of Canada (derived from Mossop et al. [6]. Only basins with thick salt units and the Canadian Shield are visualised. Occurrence of thick salts from Kabanov et al. [4]. Locality numbers correspond to the Map ID column in Table 1. Inset maps of southern WCSB (1) and SW Ontario (2) detail regions with crowded features. Provinces: BC = British Columbia; AB = Alberta; SK = Saskatchewan; MB = Manitoba; ON = Ontario; QC = Quebec; NL = Newfoundland and Labrador; NB = New Brunswick; PEI = Prince Edward Island. Territories: YT = Yukon; NT = Northwest Territories; NU = Nunavut.
• Data versatility and wide range of reported elements: whole-rock ICP lithogeochemistry with 4-acid (near-total) digestion; brined salt cores resolving contents of Li and REYs; a small set of new data from industrial brines (salt and potash mines, salt storage caverns);
• Geochemical data are compiled from original secondary sources unchanged, in their original notations; ranges of elements, physical measurements, and species vary between datasets;
• Reports new whole-rock XRD data from salt exploration drill cores;
• Informs industry stakeholders (e.g., solution mining for gas storage caverns, potash) on salt impurities and contents of trace elements, including Li, Rb,… REE+Yttrium (further REY);
• Supports ongoing Government of Canada research programs and analytical publications.

• The first Canada-wide compilation of raw geochemical data from rock salt deposits of current and potential economic use, as well as associated evaporitic strata (Fig. 1);

## 2 Geologic & Economic Context

This report compiles geochemical data from major salt formations and, partly, associated evaporitic beds in the subsurface of sedimentary basins within Canada (Fig. 1). Rock materials are diamond-bit cores from wells drilled for oil and gas, salt mines and caverns, and potash exploration. The drilling depth range of sampled cores is ∼150–2000 m, which is the depth of economic use of salts below the base of groundwater protection depth (BGWP). The limit of ∼2000 m is an approximate maximum depth where cavern walls are stable in homogenous halites [1]., and very few cores are cut in salts at greater depths, given the fact that economic use of salts there is complicated by increasingly unmanageable salt creep leading to cavern collapse [2]. The BGWP is a threshold below which groundwater salinity exceeds 4000 mg/L of total dissolved solids [3].

Many datasets collected here, especially primary data gathered since 2022, include a range of trace elements from the *Critical Raw Materials* list, such as Li, Rb, Mg, K/potash…, REY (rare earth elements + yttrium). The major element data, on the other hand, inform solution mining operations about impurities to consider in mining design. For example, considerations for H_2_ storage in salt caverns must include variable amounts of sulphate minerals (mostly anhydrite and gypsum) that co-occur with halites in variable amounts [1]. At temperatures below 80–90 °C (usual T range at depths < 2000 m), hydrogen is highly reactive with sulphates in the aqueous phase. This microbially catalysed reaction leads to the erosion of sulphate partings in the cavern walls and roof, as well as to reservoir contamination with H_2_S. Therefore, total sulphur data from geochemical datasets will be essential for selecting the best (least sulphatic) halite units for H_2_ storage-withdrawal [4].

**Fig. 2:**
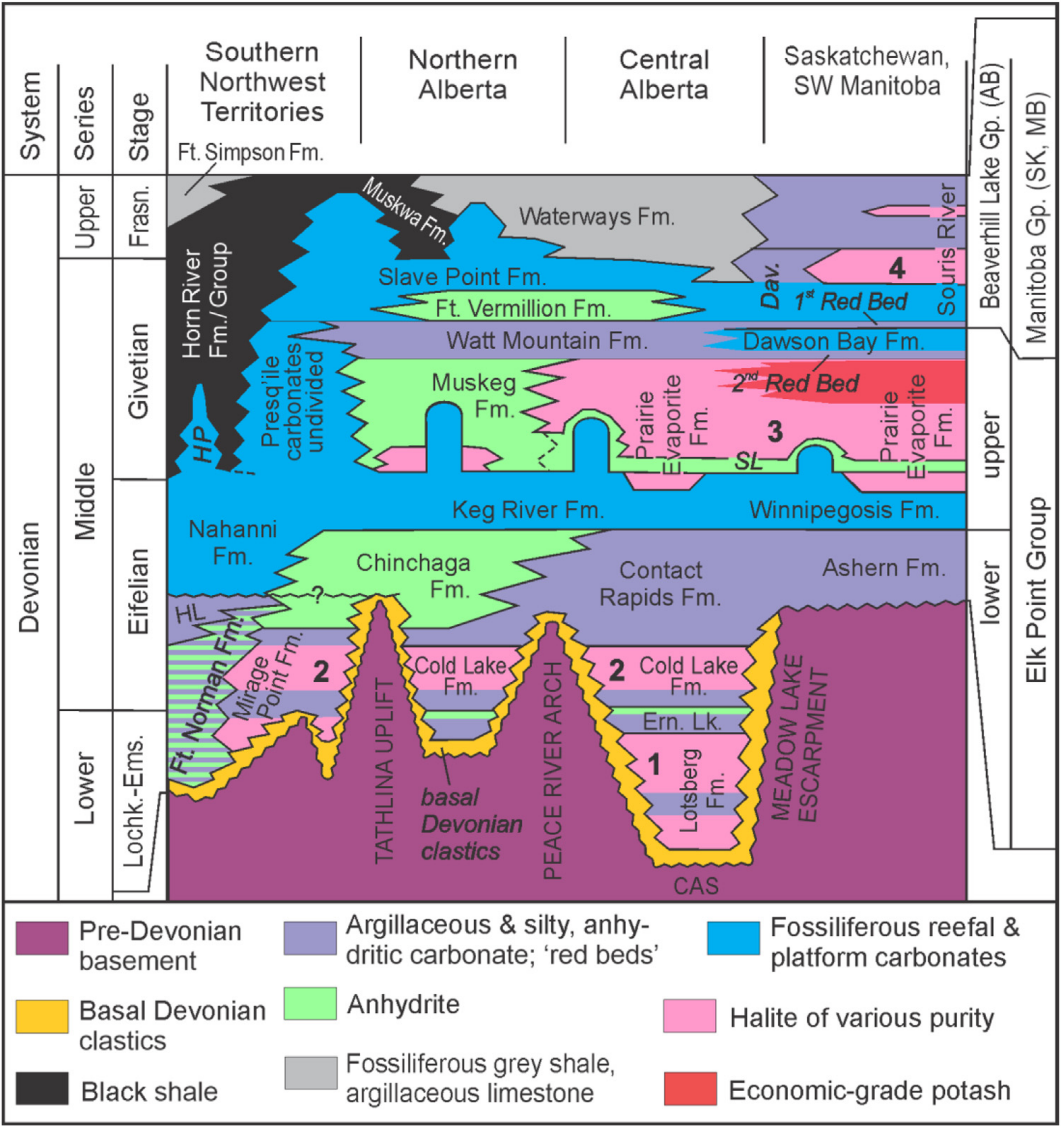
Lower-Middle Devonian evaporitic formations of WCSB. Thick salt beds of current or potential economic use: 1. Upper Lotsberg halite; 2. Cold Lake halite; 3. Prairie Evaporite halites, anhydrites, potash salts; 4. Davidson evaporite (halite) (adapted from [5]). Abbreviations: Frasn. = Frasnian; Lochk.-Ems. = Lochkovian-Emsian; Dav. = Davidson Member of Souris River Formation; SL = Shell Lake Member; HP = Horn Plateau carbonate pinnacles; HL = Headless Formation; Cold Lk. Formation = Cold Lake Formation; Ern. Lk. = Ernestina Lake Formation. CAS = Central Alberta sub-basin of the Alberta Basin, part of WCSB. Member-rank stratigraphic units appear in italics.

**Fig. 3:**
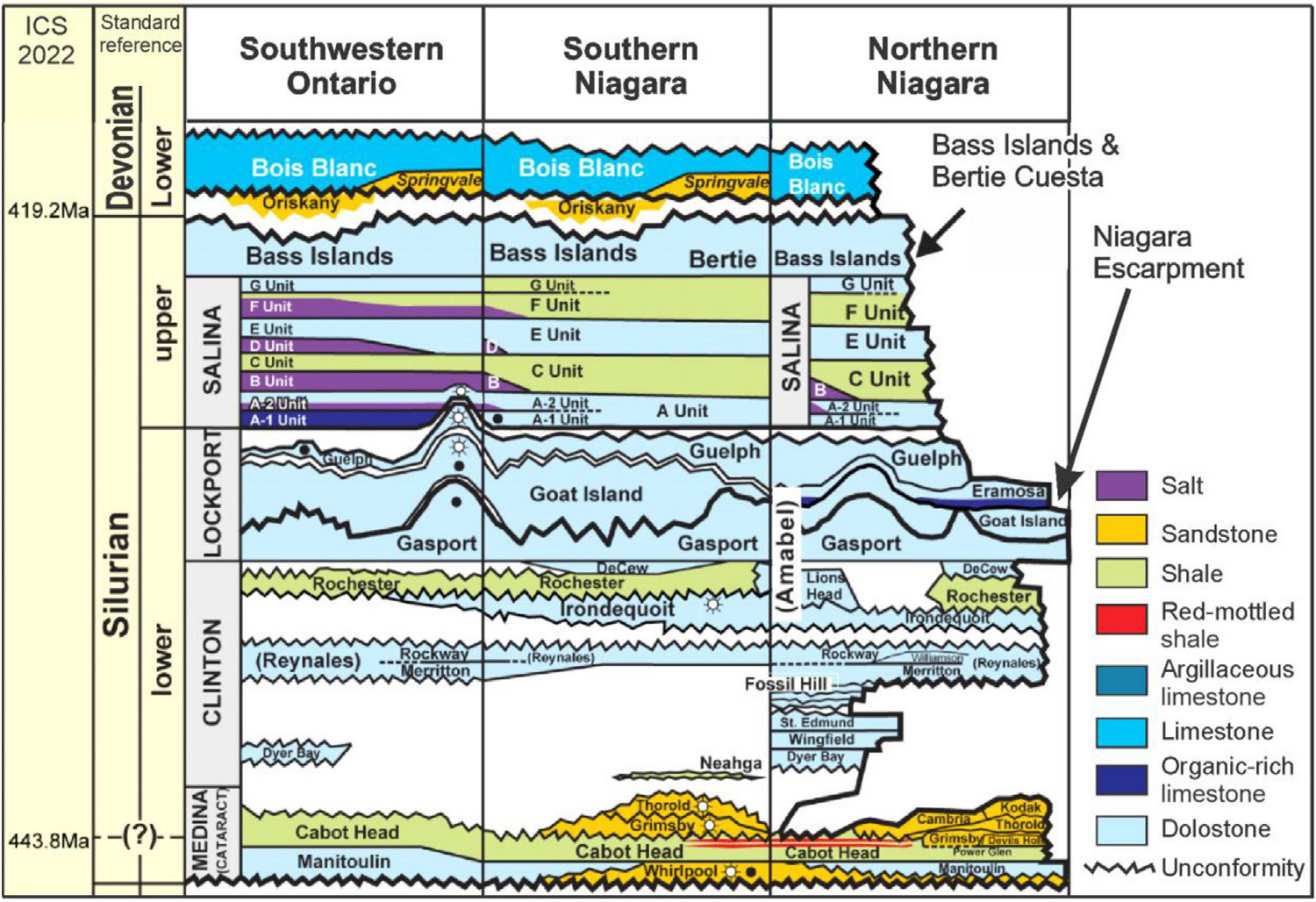
Lithostratigraphic chart of the Silurian system in southern Ontario modified from [7]. Halites of economic use occur in the Salina Group include A-2 Salt and B Salt.

**Fig. 4:**
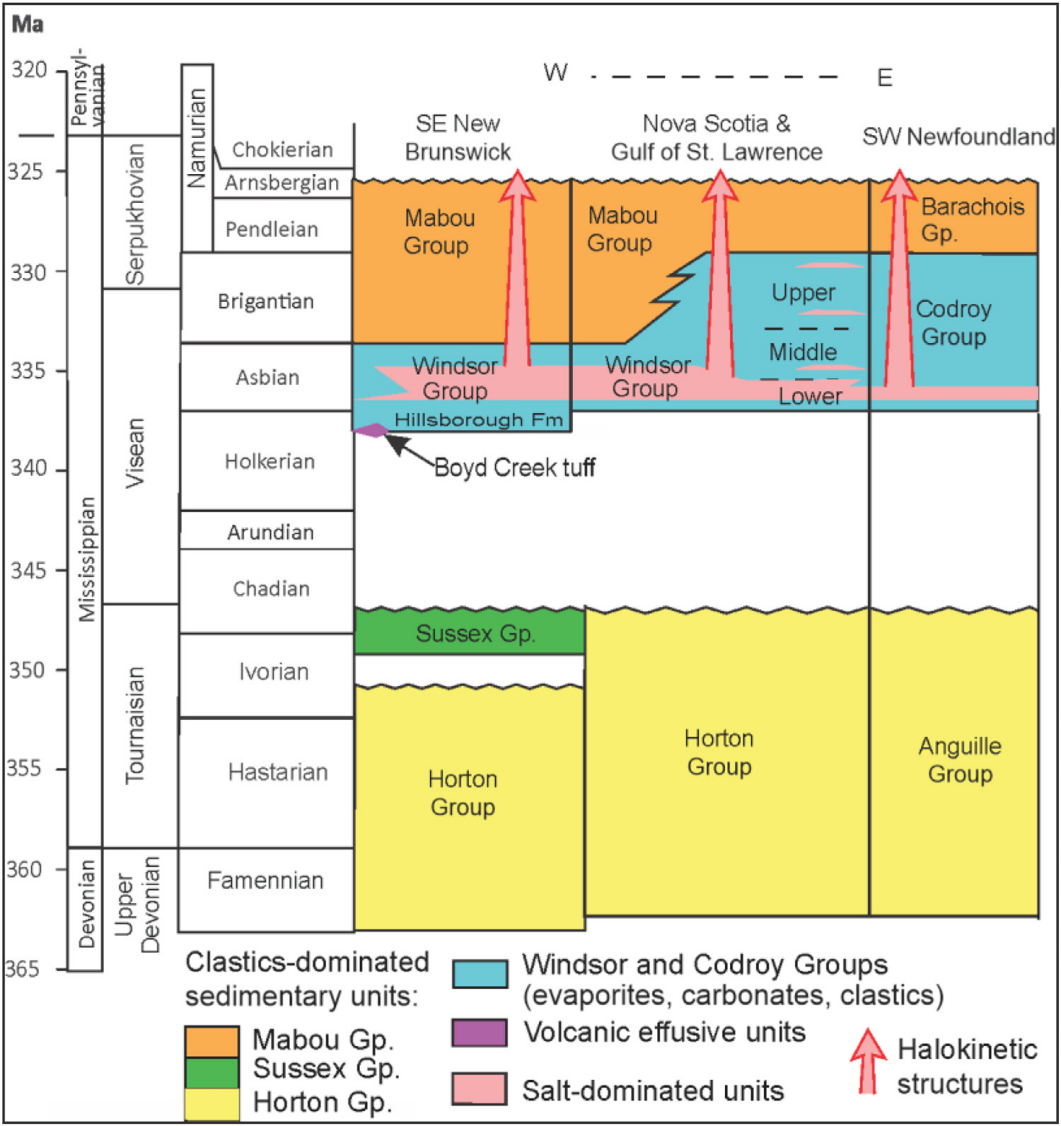
Latest Devonian and Mississippian stratified units of the Maritimes Basin, simplified to group level. The time scale follows [8], as slightly modified by [9].

**Fig. 5:**
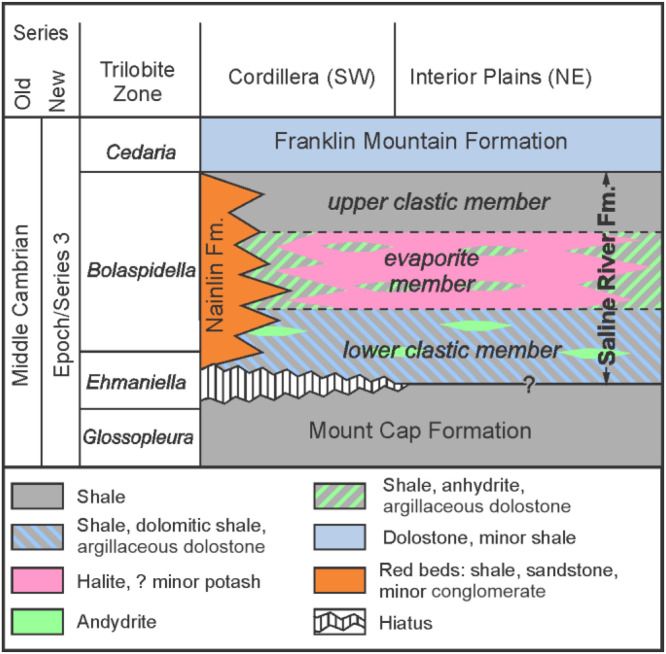
Middle Cambrian formations of the Northwest Territories within the northern WCSB. Halite-dominated salt beds occur in the evaporite member of the Saline River Formation [4].

Fig. 2, Fig. 3, Fig. 4, Fig. 5 provide graphic reference to stratigraphic assignments of samples in Table 1 and linked data tables. These evaporitic successions occur in the Lower-Middle Devonian of the Western Canada Sedimentary Basin (WCSB; Fig. 2), the Upper Silurian of the NE flank of the Michigan Basin in the southern Ontario (Fig. 3), and in the Mississippian of the Maritimes Basin of eastern Canada (Fig. 4). In addition, we report one sample set from the core intersecting Middle Cambrian salts of the northern WCSB (Fig. 5). These evaporitic successions and their salt cavern potentials are reviewed in linked papers [4,5]. Data from the Devonian of the WCSB are most extensive (data counts in Table 1). This extensive dataset represents both salts of economic use and non-salt evaporitic strata interbedding, overlying, or underlying thick salt deposits (Fig. 2).

**Table 1:** Summary of data and sample locations. Types of analysis column: L = whole-rock lithogeochemistry; BC = Brined core aqueous geochemistry; XRD = whole-rock X-ray diffraction; IB = industrial brine aqueous geochemistry; BT = brined tailings sediment. UWI is Universal Well Identifier in Dominion Land Survey (DLS) system (provinces of Alberta and Saskatchewan in this publication). Data status refers to new, sampling authorization where applicable, or previously published source.

Map ID	Data status (new, sampling authorization where appicable, or previously published source)	Appendix and Source File	Well or mine name	UWI (WCSB wells)	Latitude (NAD83)	Longitude (NAD83)	Sedimentary Basin	Material type	Stratigraphic Units	Types of analysis (Data Counts)
1	Collins & Northcott, [16]	App.A: 01LMARI	Captain Cook 5 (CC5)		48.4040653	−58.5155946	Maritime	Core	Codroy Road Formation	L (21)
2	Collins & Northcott, [16]	App.A: 01LMARI	Captain Cook 4 (CC4)		48.4134398	−58.5121839	Maritime	Core	Codroy Road Formation	L (36)
3	Miles et al., [23]	App.A: 02LMICH	Domtar Gypsum Mine		43.0789745	−79.93944505	Michigan	Core	Salina Group	L (47)
4	New	App.A: 04LMICH; App.B: 02BMICH	MTO X11–5 Gold		42.29296	−83.08473	Michigan	Core	Salina Group salt beds	L (34), BC (12)
5	New	App.A: 04LMICH; App.B: 01BMICH	89–102-V		43.738944	−81.695528	Michigan	Core	Salina Group salt beds	L (6), BC (3)
6	Hill et al., [18]	App.A: 10LWCSB	TRANSGAS VICTORIA PLAINS 5–23–18–19W2	101/05–23–018–19W2/00	50.532028	−104.5217075	WCSB	Core	Dawson Bay, Prairie Evaporite, Souris River Fms.	L (32)
7	New	App.A: 01LWCSB, 09LWCSB; App.B: 04BWCSB; App.D: 04XWCSB	TRANSGAS VICTORIA PLAINS 9–22–18–19W2	101/09–22–018–19W2/00	50.5383519	−104.5308442	WCSB	Core	Dawson Bay, Prairie Evaporite, Winnipegosis Fms.	L (209), BC (16), XRD (25)
8	Hill et al., [18]	App.A: 10LWCSB	Mosaic K3 Esterhazy 4–3–20–1W2	121/04–03–020–01W2/00	50.6808472	−102.0749728	WCSB	Core	Dawson Bay, Prairie Evaporite, Souris River Fms.	L (10)
9	Hill et al., [18]	App.A: 10LWCSB	BHPB 09–28–022–05W2	131/09–28–022–05W2/00	50.9263161	−102.6371102	WCSB	Core	Dawson Bay, Prairie Evaporite, Souris River Fms.	L (19)
10	Hill et al., [18]	App.A: 10LWCSB	Canada Golden Fortune Glenavon 16–19–15–7	141/16–29–015–07W2/00	50.2938966	−102.9244311	WCSB	Core	Dawson Bay, Prairie Evaporite, Souris River Fms.	L (13)
11	Hill et al., [18]	App.A: 10LWCSB	Encanto Sundance Lestock 2–30 2–30–27–14W2	111/02–30–027–14W2/00	51.3589589	−103.9722637	WCSB	Core	Dawson Bay, Prairie Evaporite, Souris River Fms.	L (13)
12	Lopez et al., [21]	App. A: 11LWCSB	PC LEWIS 5–24–91–6	100/05–24–091–06W4/00	56.907685	−110.832315	WCSB	Core	Keg River	L (10)
13	Meek et al., [22]	App. A: 10LWCSB	PENN WEST PROVOST 6–4–37–16	100/06–04–037–16W4/00	52.03625	−110.9578056	WCSB	Core	Dawson Bay, Prairie Evaporite, Souris River Fms.	L (1)
14	Lopez et al., [21]	App. A: 11LWCSB	IMP 13 DV-01 STEEPBK 4–2–94–7	103/04–02–094–07W4/00	57.122271	−111.018575	WCSB	Core	Contact Rapids	L (2)
15	Hill et al., [18]	App. A: 10LWCSB	PCS 13–25–032–24 W2	131/13–25–032–24W2/00	51.7785175	−105.2846391	WCSB	Core	Dawson Bay, Prairie Evaporite, Souris River Fms.	L (16)
16	Hill et al., [18]	App. A: 10LWCSB	KSPC FINDLATER 3–14–20–25W2	101/03–14–020–25W2/00	50.6887542	−105.3677481	WCSB	Core	Dawson Bay, Prairie Evaporite, Souris River Fms.	L (17)
17	Hill et al., [18]	App. A: 10LWCSB	PCS ALLAN SWD 16–28–034–01W3	111/16–28–034–01W3/00	51.9511667	−106.0774166	WCSB	Core	Dawson Bay, Prairie Evaporite, Souris River Fms	L (8)
18	Meek et al., [22]	App. A: 12LWCSB	PENN WEST PROVOST 6–4–37–16	100/06–04–037–16W4/00	52.147566	−112.232271	WCSB	Core	Red River, Winnipegosis formations	L (16)
19	Hill et al., [18]	App. A: 10LWCSB	PCS Saskatoon 10–6–36–6 W3M	141/10–06–036–06W3/00	52.0663889	−106.8438889	WCSB	Core	Dawson Bay Formation, Prairie Evaporite Formation, Souris River Formation	L (15)
20	Hill et al., [18]	App. A: 10LWCSB	AGRIUM VANSCOY 1–15–35–7 W3	141/01–15–035–07W3/00	52.0014517	−106.910026	WCSB	Core	Dawson Bay, Prairie Evaporite, Souris River Fms.	L (21)
21	Sasketchwan Geological Survey	App. A: 13LWCSB	Nutrien Vanskoy Potash mine	miscellaneous	52.0092597	−107.087118	WCSB	Potash ore	Prairie Evaporite	L (137)
22	Sasketchwan Geological Survey	App. A: 14LWCSB	K+S Potash Bethune mine	miscellaneous	50.6489856	−105.3701616	WCSB	Potash ore	Prairie Evaporite	L (155)
23	Sasketchwan Geological Survey	App. A: 15LWCSB	Rio Tinto Sedley 4–22–14–15 W2	111/04–22–014–15W2/00	50.179528	−103.979972	WCSB	Core	Prairie Evaporite	L (107)
24	Sasketchwan Geological Survey	App. A: 16LWCSB	Karnalyte Wynyard 3A11–27–31–16 W2M	111/11–27–031–16W2/00	51.686806	−104.193639	WCSB	Core	Prairie Evaporite	L (259)
25	Sasketchwan Geological Survey	App. A: 16LWCSB	Karnalyte Wynyard DD 2A11–12–3D6–32–16W2	111/06–12–032–16W2/00	51.729528	−104.146417	WCSB	Core	Prairie Evaporite	L (274)
26	Sasketchwan Geological Survey	App. A: 17LWCSB	Karnalyte Wynyard 13–36–31–16 W2M	121/13–36–031–16W2/00	51.703944	−104.153361	WCSB	Core	Prairie Evaporite	L (596)
27	Sasketchwan Geological Survey	App. A: 18LWCSB	RIO TINTO KRONAU 16–16–15–16W2	141/16–16–015–16W2/00	50.265322	−104.137444	WCSB	Core	Prairie Evaporite	L (129)
28	Sasketchwan Geological Survey	App. A: 19LWCSB	RIO TINTO ODESSA 13–16–15–14W2	141/13–16–015–14W2/00	50.265289	−103.881333	WCSB	Core	Prairie Evaporite	L (130)
29	Sasketchwan Geological Survey	App. A: 20LWCSB	CDN LAND MEDHAT 14–36–20–1	100/14–36–020–01W4/00	50.742995	−110.019251	WCSB	Core	Prairie Evaporite	L (26)
30	Tobola & Kukialka, [10]	App. A: 21LWCSB	PEMBINA NGL 22A REDWATER 7–12–56–22	100/07–12–056–22-W4/0	53.823917	−113.134581	WCSB	Core	Upper Lotsberg halite	L (21)
31	Tobola & Kukialka, [10]	App. A: 21LWCSB	PEMBINA NGL 21A REDWATER 2–12–56–22	100/02–12–056–22-W4/1	53.820736	−113.135943	WCSB	Core	Upper Lotsberg halite	L (59)
32	New (SR–2024–001 granted by OROGO, Northwest Territories)	App. B: 07BWCSB	Mobil Colville E-15	300/E–15–6720–12,615/0	67.238515	−126.309202	WCSB (north)	Core	Evaporite member of Saline River Formation	BC (11)
33	New (GOS-5885, 5892, 5897, 5899 by Alberta Energy Regulator); partly published (Kabanov et al., 2024)	App. A: 01LWCSB, 02LWCSB, 03LWCSB, 09LWCSB App.B: 09BWCSB; App.D: 01XWCSB	PMC140FT SASK 7–23–55–22	100/07–23–055–22W4/03	53.763396	−113.15587	WCSB	Core	Watt Mountain, Prairie Evaporite, Keg River, Contact Rapids, Ernestina Lake, Lotsberg Fms.	L (331), BC (17), XRD (89)
34	Lopez et al., [21]	App. A: 11LWCSB	CHEVRON LUTOSE 1–22–118–20	100/01–22–118–20W5/00	59.258043	−117.30467	WCSB	Core	Muskeg Fms.	L (1)
35	New	App. B: 02BMARI; App. D: 02XMARI	Chevron-Irving Malagawatch 2 (CM-2)		45.8672222	−60.9230556	Maritime	Core	Windsor Group	BC (7), XRD (23)
36	New	App. B: 02BMARI, App. D: 02XMARI	Kempt Head - 84–1		46.0758333	−60.6425	Maritime	Core	Windsor Group	BC (11), XRD (42)
37	New (GOS-6023 granted by Alberta Energy Regulator)	App.B: 05BWCSB App.D: 05XWCSB	CNRL 8B WD LIND 8–13–57–5	100/08–13–057–05W4/00	53.923969	−110.6039	WCSB	Core	Cold Lake Formation	BC (8), XRD (6)
38	New	App.B: 02BMARI	IMC#3		45.578206	−65.545728	Maritime	Core	Cassidy Lake Formation	BC (14)
39	New	App.B: 02BMARI	IMC#4		45.569762	−65.608263	Maritime	Core	Cassidy Lake Formation	BC (4)
40	New	App.B: 02BMARI; App.D: 02XMARI	Stewiacke Borax 1 (SB-1)		45.155	−63.3205556	Maritime	Core	Windsor Group	BC (9), XRD (33)
41	New (GOS-7296 granted by Alberta Energy Regulator)	App.A: 08LWCSB App.B: 08BWCSB; App.D: 03XWCSB	CVE FCCL SALT-2 FISHER 2–10–70–4	100/02–10–070–04W4/00	55.042081	−110.521519	WCSB	Core	Watt Mountain Formation, Prairie Evaporite	L (25), BC (15), XRD (55)
42	New	App. B: 01BMARI	Nappan Salt Mine		45.78333	−64.23417	Maritime	Industrial Brine	Windsor Group	IB (12)
43	New	App. B: 03BWCSB	Fort Kent cavern operation		54.45206	−110.48923	WCSB	Industrial Brine	Upper Lotsberg	IB (15)
44	New	App. B: 03BWCSB	Keyera Ft. Sask cavern operation		53.748846	−113.157692	WCSB	Industrial Brine	Upper Lotsberg	IB (2)
45	New	App. B:03BMARI, App. A. 02LMARI	PF-1		48.3143743	−58.573255	Maritime	Core	Woodville Formation	L (5), BC (5)
46	New	App. B:03BMARI, App. A. 02LMARI	LR98–1		48.3090534	−58.5872525	Maritime	Core	Woodville Formation	L (11), BC (13)
47	Lazowski et al., [20]	App. A: 22LWCSB	Otter Lake Core	100/01–01–089–10W5/00	56.684691	−115.4409	WCSB	Core	Fort Vermilion Formation	L (16)
48	Hill et al., [19]	App.A: 10LWCSB	Well Licence 69A013 06–10–031–23W2	141/06–10–031–23W2/00	51.64033	−105.1837301	WCSB	Core	Prairie Evaporite Formation	L (13)
49	Hill et al., [19]	App.A: 10LWCSB	Well Licence 65I095 16–02–030–21W2	131/16–02–030–21W2/00	51.5461703	−104.8458897	WCSB	Core	Prairie Evaporite Formation	L (11)
50	Hill et al., [19]	App.A: 10LWCSB	Well Licence 03G097 05–15–036–09W3	111/05–15–036–09W3/00	52.0897196	−107.2133293	WCSB	Core	Prairie Evaporite Formation	L (8)
51	Hill et al., [19]	App.A: 10LWCSB	Well Licence 87K022 12–20–004–08W2	131/12–20–004–08W2/00	49.3139502	−103.0583405	WCSB	Core	Prairie Evaporite, Winnipegosis Fms.	L (17)
52	Hill et al., [19]	App.A: 10LWCSB	Well Licence 10I303 05–20–094–25W3	101/05–20–094–25W3/00	57.16697	−109.998928	WCSB	Core	Dawson Bay Formation	L (13)
53	Hill et al., [19]	App.A: 10LWCSB	Well Licence 10I151 13–15–094–25W3	101/13–15–094–25W3/00	57.1600402	−109.945462	WCSB	Core	Dawson Bay Formation	L (10)
54	Hill et al., [19]	App.A: 10LWCSB	Well Licence 12G213 01–03–040–10W3	121/01–03–040–10W3/00	52.4069997	−107.3532203	WCSB	Core	Dawson Bay, Prairie Evaporite Fms.	L (21)
55	Hill et al., [19]	App.A: 10LWCSB	Well Licence 66F092 13–28–038–08W3	111/13–28–038–08W3/00	52.3009201	−107.0932497	WCSB	Core	Dawson Bay, Prairie Evaporite, Souris River Fms.	L (24)
56	Hill et al., [19]	App.A: 10LWCSB	Well Licence 67F077 04–19–048–24W2	101/04–19–048–24W2/00	53.2009199	−105.5378099	WCSB	Core	Dawson Bay, Winnipegosis Fms.	L (14)
57	Hill et al., [19]	App.A: 10LWCSB	Well Licence 68I044 08–14–054–12W3	121/08–14–054–12W3/00	53.6621704	−107.6582593	WCSB	Core	Dawson Bay, Prairie Evaporite Fms.	L (13)
58	Hill et al., [19]	App.B: 06BWCSB	Allan Potash Mine	N/A	53.2009199	−105.5378099	WCSB	Industrial Brine	Prairie Evaporite	IB (8)

## 3 Data Description

In this compilation, datasets are assessed for provenance (peer-reviewed literature, government agencies, national geological surveys, recognised standards bodies) and transparent methodology. The geochemistry data files are organised in multi-tabular Microsoft Excel files (Appendices A, B, D, E) where tables/source files are made of number, letters “L”, “B”, “X” (whole-rock lithogeochemistry, XRD, or brine geochemistry, respectively), and the first four letters of a sedimentary basin name (WCSB = Western Canadian Sedimentary Basin, MARI = Maritimes Basin, and MICH = Michigan Basin). Within each source file, each record is assigned a UniqueID constructed as the SourceCode and a row number (e.g., 01LWCSB1, 01LWCSB2). Most records provide verifiable geolocation (latitude/longitude with the stated coordinate reference system), sampling date/time, units, detection/quantification limits (LQL and UQL), and concise descriptions of field and laboratory methods. Units and reference frames are harmonised before analysis to enable processing of various data sheets concatenated using Pandas, a Python library that provides fast, flexible data frame and series structures for cleaning, reshaping, and analysing tabular data.

Noteworthy with the major element data, new lithogeochemical data were commercially acquired at ActLabs Ltd. These include Al, Ca, Mg, Fe, K, S with the representative lower and upper detection/quantification limits (further LQL and UQL). They also include Na with the UQL ranging between 3–10 wt.%. This limit of Na detection is not representative for halite-rich samples. Additionally, selected lab protocols do not report Si, however, a wealth of Si/SiO2 data are available from previously published sources collected in Appendix A.

Data are available in the following supplementary files depending on their type and analytical method:

• Appendix A_Whole-rock_lithogeochemistry.xlsx.
• Appendix B_ Appendix B_industrial brines_and_brined_cores.xlsx.
• Appendix C_Diffractograms of brined core insoluble residues.xlsx.
• Appendix D_Whole-rock_X-ray_diffraction_data.xlsx.
• Appendix E_Original RTG lab data from diluted brines.xlsx.
• Appendix F_Report on field sampling.pdf.
• Appendix G_collection of ActLab certificates for new data.zip.

## 4 Experimental Design, Materials and Methods

This section summarises lab protocols for new (previously unpublished) data obtained at the University of Calgary. Lab protocols for previously published data are available in publications listed in Table 1. Previously unpublished data is reported for which data aquisition methodology is betailed in Toboła and Kukiałka [10].

### 4.1 Whole-rock lithogeochemistry

#### 4.1.1 (Appendix A, source files 01LWCSB, 04LMICH)

Whole rock lithogeochemical analysis is performed by Activation Laboratories Ltd. As standard procedure, Activation Labs crushes the entire core sample, and sub-samples are pulverised to 95% passing 105 µm using mild steel mills [11]. In 4 acid digestion, samples undergo a vigorous digestion using a combination of concentrated hydrochloric (HCl), nitric (HNO3), perchloric (HClO4), and hydrofluoric (HF) acids [11]. Activation Labs 4-acid whole-rock lithogeochemistry analyses are performed according to procedures for codes 1F2 and ultra-trace 6 (UT6). 1F2 represents analysis conducted via Inductively Coupled Plasma Optical Emission Spectrometry (ICP-OES), and UT6 analysis utilises a combination of ICP-OES and ICP-MS (Inductively Coupled Plasma Mass Spectrometry) to achieve lower detection limits. To ensure reproducibility, digestion is performed in an automated microprocessor-designed hotbox [11]. Activation Labs is ISO/IEC 17,025:2017 accredited (incorporating ISO 9001:2015 and ISO 9002 standards). The laboratory is accredited by the Standards Council of Canada (SCC) for mineral testing and is subject to routine audits by four regulatory agencies.

### 4.2 Industrial brines and brined cores

#### 4.2.1 Appendix B, source files 01BMARI, 01BMICH, 02BMARI, 02BWCSB, 03BWCSB, 04BWCSB, 05BWCSB, 06BWCSB, 07BWCSB

The industrial brines and brined core samples were analysed in 2024–2025 at the Reactive Transport Group facilities in the Department of Earth, Energy and Environment at the University of Calgary. The work involved analysis of leachate and brine samples, with brine REE separated and preconcentrated using the cation-exchange approach of Chevis et al. [12], implemented via an in-house standard of practice. Solid samples were leached by digesting 1 g of material in 1 kg of deionised water on a rotator for 30–60 min. Following leaching, conductivity was measured (between ∼125 and 1500 µS/cm). Solutions were filtered using 0.45 µm polyvinylidene fluoride (PVDF) syringe filters (Millipore Millex-HV), and ∼120 mL was preserved for anion and cation/REE analyses. The density was calculated from conductivity and ranged from ∼0.9 to 1.3 g/cm3, with a resolution of 0.1 g/cm3. To ensure that TDS was <2000 mg/L for ICP-OES and < 1000 mg/L for ICP-MS, from preserved aliquots for anions and cations/REE, 100 µL subsamples were diluted to 10 mL prior to analysis. Cation aliquots were preserved with 100 µL ultrapure nitric acid and stored at 4 °C until analysis and were analysed within 14 days of preservation. Anions (chloride and sulphate) were measured by ion chromatography, major cations by ICP-OES, and REE by ICP-MS.

Chloride and sulphate (Cl⁻ and SO₄²⁻) concentrations were measured by ion chromatography (IC) using a Thermo Scientific Dionex ICS-2000 system. Sample aliquots were diluted with deionised water and calibrated (R² > 0.999) using serial dilutions of certified standards (Ricca Chemical Company). Certified 1000 mg L⁻¹ standards were used for SO₄²⁻, with calibration ranges of 5, 10, and 20 mg L⁻¹, while Cl⁻ calibration ranges were 100, 200, and 300 mg L⁻¹. Samples were analysed in duplicate, and mean concentrations are reported. Analytical precision for IC measurements is generally better than ±5%.

Quantification of YREEs was performed using a ThermoFisher iCAP™ Triple Quadrupole ICP-MS at the University of Calgary. Analyses were conducted in both single-quadrupole kinetic energy discrimination (SQ-KED, He collision gas) and triple-quadrupole oxygen reaction mode (TQ-O₂) to resolve known polyatomic interferences. Instrument tuning was optimised daily to maintain low CeO⁺ and Ce²⁺ formation (<2.0% and 2.5%, respectively). External calibration (0.001–10 µg L⁻¹) was performed using NIST-traceable standards, with ¹¹⁵In used as an internal standard. Total isotopes measured included 139La, 140Ce, 141Pr, 143Nd, 145Nd, 146Nd, 147Sm, 149Sm, 151Eu, 153Eu, 155Gd, 157Gd, 158Gd, 159 Tb, 161Dy, 163Dy, 165Ho, 166Er, 167Er, 169Tm, 172Yb, 173Yb, and 175Lu. To mitigate known polyatomic and oxide interferences (e.g., BaO⁺ on Eu, CeO⁺ and NdO⁺ on adjacent masses), selected REEs were additionally quantified in triple-quadrupole oxygen reaction mode (TQ-O₂) by monitoring mass-shifted oxide species (e.g., LaO⁺, CeO⁺, NdO⁺, SmO⁺, EuO⁺, GdO⁺), enabling interference-free determination. In TQ-O₂ mode, REEs susceptible to isobaric or oxide-based interferences (including Eu, Gd, Tb, Dy, and Ho) were quantified using their corresponding oxide product ions (M⁺ to MO⁺ mass shifts of +16 amu). Both native-mass and O₂-shifted measurements were evaluated, and final concentrations were reported from the mode yielding the lowest background and highest analytical precision. Samples were measured in quintuplicate (*n* = 5), and mean values were reported when RSD ≤ 5%. Instrument performance was monitored using procedural blanks, drift corrections, and certified reference materials. Canadian certified reference materials project standards (e.g., CCRMP REE-2) were run intermittently to assess background and matrix effects, as well as unknowns, to monitor instrument accuracy and inter-run drift. Recovery rates for REEs were within ±10% of certified values.

Quantification of minor and major cations (Fe, Al, K, Ba, Li, Mn, Si, Ca, Mg, Sr, and Na) were quantified using a Varian 725-ES ICP-OES operated in radial view configuration at the University of Calgary. The instrument was wavelength-calibrated daily using an Agilent ICP-OES Wavelength Calibration Solution (Part No 6610,030,100), containing a multi-element mixture to ensure accurate spectral alignment across the 190–800 nm range. Quantification was performed using the following analytical emission lines selected for optimal sensitivity and minimal spectral interference: Fe (259.940 nm), Ca (317.933 nm), Mg (279.553 nm), Na (589.592 nm), K (766.491 nm), Mn (257.610 nm), Al (396.152 nm), Si (251.611 nm), Ba (455.403 nm), Sr (407.771 nm), and Li (670.784 nm). Line selection was confirmed using manufacturer-supplied interference tables and calibration standard checks. Calibration curves were constructed using NIST-traceable Aristar® 1000 µg mL⁻¹ ICP standards (VWR BDH Chemicals), diluted to working concentrations. External calibration for ICP-OES analysis was performed using multi-element calibration standards prepared with concentration ranges tailored to each analyte to ensure a linear detector response and optimal sensitivity across the expected concentration ranges. Calibration points spanned 0–10 mg L⁻¹ for Al, K, Li, Mn, Si, Ba, Fe, and Sr; 0–40 mg L⁻¹ for Ca and Mg; and 0–100 mg L⁻¹ for Na. Calibration curves were verified every 10 samples, and linearity was confirmed for all elements (R² ≥ 0.999). Both 18.2 MΩ·cm deionised water and 2% trace-metal-grade HNO₃ rinse blanks were analysed after each block to monitor carryover and baseline stability. Each sample was analysed in quintuplicate (*n* = 5), with precision assessed via relative standard deviation (RSD), which was typically ≤2% and never exceeded 5% across the measured concentration range. Accuracy was evaluated using method blanks, sample duplicates, and spike-recovery tests, with recoveries ranging from 92% to 105% for all reported elements. The method detection limit (MDL) for analytes was <0.001 mg L⁻¹. Detection limits for all elements were determined using a signal-to-noise ratio of 3:1, verified against method blanks and low-level calibration standards for each analytical run. No significant matrix interferences or spectral overlaps were observed under the selected operating conditions.

Original results are reported in Appendix E and Appendix B (tabs 04BWCSB, 04XWCSB, 07BWCSB, 02BMARI, 05BWCSB), with values noted as uncorrected and corrected for dilution factors, respectively. Insoluble residues from brine samples were qualitatively analysed by XRD.

### 4.3 Brine REY separation and preconcentration by cation exchange

Brine REYs were separated and preconcentrated using a cation-exchange method modified from [12], implemented as an in-house standard operating procedure. This approach was selected because REY 258 concentrations in natural waters, including seawater-derived and industrial brines, typically range from 259 picomolar to low nanomolar, whereas matrix elements such as Na, Ca, Ba, Mg, and Sr occur at 260 millimolar to molar concentrations [[13], [14]]. Direct analysis of such solutions without matrix removal leads to severe spectral and matrix interferences (e.g., BaO⁺ on Eu, Ca- and Na-based polyatomics, ionisation suppression) that cannot be fully resolved by digestion alone or by calibration-based corrections. The column-based preconcentration step is therefore critical to ensure enrichment of REYs into a measurable concentration range and to allow the quantitative removal of high-matrix cations (Na, Ca, Ba, Mg) that interfere with accurate REY determination by ICP-MS.

Depending on initial sample volume (30–60 mL) and final reconstitution volume (10 mL), the effective REY preconcentration factor was approximately 3x to 6x, while simultaneously reducing the absolute matrix load by more than an order of magnitude. This matrix separation is essential for resolving true REY abundances in brines and salts and is more effective than total-digestion approaches, which dissolve all matrix components and can exacerbate interference and signal suppression [15].

The protocol comprises three stages: 1) Resin Cleaning; 2) Sample Loading; 3) Elution. In this section, sample loading refers specifically to loading REY-bearing brine (or other aqueous) solutions onto the cation-exchange column so REY bind to the resin, followed by sequential rinses and REY elution. Resin was pre-cleaned using sequential strong-acid soaks of trace metal grade (3–10 M HCl and 6–7 M HNO₃; 3 mL per step with 15-minute soaks; Table 2), followed by a final ∼30-minute rinse with 6 mL Milli-Q water, and stored wet with 5 mL Milli-Q water to prevent drying and bed separation, in a dark, dry setting to limit evaporation. REY-bearing aqueous samples were column-loaded by passing 30–60 mL (staged as ∼10 mL every ∼15 min, repeated ∼3 times) through Bio-Rad Poly-Prep columns packed with ∼2 mL AG 50W-X8 resin (100–200 mesh, H⁺ form), so REY were retained while major solutes passed through to waste. After column loading, the resin was rinsed with 3 mL of 1.75 M HCl (to remove Fe) and then with 3 mL of 2.00 M HNO₃ (to remove Ba), with both fractions discarded as waste. REY were eluted using 8.00 M HNO₃, collected 5 mL into Falcon tubes as the REY elution fraction. These targeted rinse steps are critical for minimising oxide-based interferences (e.g., BaO⁺ on Eu) during subsequent ICP-MS analysis. The collected REY elution fraction was dried down by evaporation on a 90 °C hotplate in a disposable beaker that was half-covered with Parafilm. After dry-down, the REY fraction was reconstituted to 10 mL in dilute nitric acid prior to ICP-MS analysis.

**Table 2:** Acid solutions used for sequential cleaning of AG 50W-X8 cation-exchange resin. Stock concentrations, target molarities (M), and volumes required to prepare 100 mL of each solution are listed in order of application.

Acid Solution	Stock Acid Concentration	Target Concentration (M)	Volume Needed (mL)	Milli-Q Water (mL)
3 M HCl (1st)	12M	3M	25.0	75.0
6 M HCl (2nd)	12M	6M	50.0	50.0
7 M HNO₃ (3rd)	16M	7M	43.75	56.25
10 M HCl (4th)	12M	10M	83.3	16.7
6 M HNO₃ (5th)	15.7M	6M	37.97	62.03
3 M HCl (6th)	12M	3M	25.0	75.0
Milli-Q Water (7th)	-	-	-	100

This column-based separation and preconcentration approach is widely used in REY geochemistry for aqueous and brine samples because it provides superior analytical accuracy compared with bulk or total digestion methods when REYs are present at ultra-trace levels. Total digestion, including four-acid (HF-based) approaches, dissolves the entire matrix but does not remove interfering elements and can worsen spectral overlaps and matrix suppression effects in high-salinity samples. In contrast, cation-exchange separation isolates the REY fraction, reduces matrix load, improves detection limits, and enables accurate determination of true REY concentrations in salts and brines. This approach was necessary because high-salinity brines contain major ions (Na, Ca, Ba) at concentrations that cause severe matrix and spectral interferences during ICP-MS analysis, which are not mitigated by total digestion alone [16].

### 4.4 Adjustments & considerations

The method follows a stepwise acid-cleaning procedure to ensure complete removal of contaminants. Properly diluted HCl and HNO₃ are used for resin conditioning and preconcentration. The sample loading process aligns with standard preconcentration methods for ICP-MS analysis. Ferric iron coprecipitation is an available contingency in the Chevis et al. [12] workflow for very high-REE matrices; this work proceeds directly via column separation and elution without specifying coprecipitation.

### 4.5 X-ray diffraction

#### 4.5.1 Appendix D source files: 01XWCSB, 02XMARI, 03XWCSB

One hundred and fifty-five samples from four drill cores were commercially analysed at Activation Laboratories, Ancaster, Ontario. Whole-rock mineral composition was analysed in every sample. In addition, clay speciation was examined in three clay-fraction samples from the Fisher 2–10–70–4 core. Diffractograms for each sample are collected in lab certificates (Appendix G).

For the semi-quantitative XRD analysis, a portion of each pulverised sample was loaded into a standard holder. For clay speciation analysis, a portion of each sample was dispersed in distilled water, and the < 4 µm size fraction was separated by gravity settling of particles in suspension. Oriented slides of the < 4 µm size fraction were prepared by placing a portion of the suspension onto a glass slide. The oriented slides were analysed air-dry and after treatment with ethylene glycol.

The X-ray diffraction analysis was performed on a Bruker D8 Endeavour diffractometer equipped with a Cu X-ray source and operating at the following conditions: 40 kV and 40 mA; range 4 - 70 ° 2θ; step size 0.02 ° 2θ; time per step 0.5 *sec*; fixed divergence slit, angle 0.30; sample rotation 15 rpm. The PDF4/Minerals ICDD database was used to identify minerals. The quantities of the crystalline mineral phases were determined using the Rietveld method. The Rietveld method is based on calculating the full diffraction pattern from crystal structure data. The relative proportions of clay minerals in the < 4 µm size fraction were calculated using the relative ratios of their basal-peak areas.

### 4.6 X-ray diffraction

#### 4.6.1 Appendix D source file 04XWCSB

This batch of samples from the potash exploration core of Regina area, Saskatchewan, was analysed at Geological Survey of Canada, Ottawa. Twenty-five whole-rock XRD data were obtained (Appendix D) using the same instrumentation and analytical protocol as in previously reported XRD results [17].

The mineralogy of bulk materials and clay-size separates is determined by X-ray powder diffraction analysis (XRD). Bulk samples are micronised using a McCrone mill in isopropyl alcohol or distilled water until a grain size of about 5- 10 µm is obtained. The samples are dried and then back pressed into an aluminium holder to produce a randomly oriented specimen. X-ray patterns of the pressed powders or air-dried samples are recorded on a Bruker D8 Advance Powder Diffractometer equipped with a Lynx-Eye Detector, Co Kα radiation set at 35 kV and 40 mA, over a range of 2–86° 2θ. The samples are also X-rayed following saturation with ethylene glycol (2–86° 2θ) and heat treatment at 550 °C for 2 h (2–35° 2θ).

### 4.7 Mineral identification and quantitative analysis

Initial identification of minerals is made using EVA (Bruker AXS, Inc.) software, with comparisons to reference mineral patterns in Powder Diffraction Files (PDF) from the International Centre for Diffraction Data (ICDD) and other available databases. Quantitative analysis is carried out using TOPAS (Bruker AXS, Inc.), a PC-based program that performs Rietveld refinement (RR) of XRD spectra. This is based on a whole-pattern-fitting algorithm. It relies on having mineralogical structure files (.cif) such that the reference minerals are as close a match to the unknown as possible.

## Limitations

Note that the 4-acid digestion method used in 1F2 and UT6 protocols does not provide accurate determinations of REYs, whereas primary data on brined cores analysed at the University of Calgary deliver more precise REY results on solubles. The compiled dataset is not uniformly distributed across formations (See Table 3). The Prairie Evaporite Formation dominates the record, accounting for over 2100 entries across all data types, while formations such as the Muskeg Formation, Red River Formation, and Cold Lake Formation contribute fewer than ten entries each. Intermediate representation is found for the Lotsberg Formation, Windsor Group, Salina Group, and Contact Rapids Formation, yet even among these formations, the coverage varies considerably by data type. For instance, the Windsor Group is well represented in XRD (100 entries) and brined core (27 entries) yet entirely absent from lithogeochemistry, whereas the Lotsberg Formation contributes 188 lithogeochemistry entries and 77 XRD entries but only 8 brined core entries. Similarly, the Salina Group appears in lithogeochemistry (87 entries) and brined core (15 entries) but is absent from XRD records. This uneven sampling reflects differences in the geographic extent, economic relevance, and exploration history of each formation rather than a deliberate analytical choice, and it follows that generalisations drawn from the dataset are more robust for heavily sampled units such as the Prairie Evaporite Formation than for those with limited entries. Results for underrepresented formations may not capture the full range of variability in thickness, composition, or spatial distribution.

**Table 3:** Summary of data entries by geological formation and analytical data type. XRD = X-ray diffraction, L = lithogeochemistry, BC = brined core. Blank cells indicate no data available for that formation-method combination. Formations are listed in descending order of total entry count. FormationFormationFormationFormationFormationFormationFormationFormationFormationFormationFormationFormationFormationFormationFormationFormationFormation.

Formation Name	L	BC	XRD	Total
Cassidy Lake	0	18	0	18
Codroy Road	74	0	0	74
Cold Lake	0	8	9	17
Contact Rapids	62	0	0	62
Dawson Bay	189	0	1	190
Elk Point	1	0	0	1
Ernestina Lake	18	0	12	30
Fort Vermillion	16	0	0	16
Keg River	40	0	0	40
Upper Lotsberg	198	0	77	275
Muskeg	4	0	0	4
Prairie Evaporite	2234	74	78	2386
Red River	4	0	0	4
Salina	87	15	0	102
Saline River	0	12	0	12
Souris River	15	0	0	15
Watt Mountain	30	0	2	32
Windsor Group	0	27	100	127
Winnipegosis	25	0	2	27
Woodville	**0**	**18**	**0**	**18**
Industrial salt	0	37	0	37
TOTAL	**2997**	**209**	**281**	**3487**

## Ethics Statement

The authors have read and follow the *ethical requirements* for publication in Data in Brief and confirming that the current work does not involve human subjects, animal experiments, or any data collected from social media platforms.

## CRediT Author Statement

**Cristine (Joy) Yap:** main data preparation, sample and data curation, lab works; **Magnus Roland Marun:** secondary data acquisition, main data compilation, methodology, and writing; **Pavel Kabanov:** conceptualization, data organization, writing, funding acquisition; **Jaxon Dii Horne**: analytical methodology, instruments, lab works; **Peter Giles, Paul Durling, Frank Brunton,** and **Susan Johnson**: sample collection and geological context support; **Tomasz Toboła** and **Piotr Kukiałka**: sampling, analytical methodology, lab works.

## Data Availability

Mendeley DataGeochemistry and X-ray diffraction data from rock salts and saltwork wastes of Canada: data compilation (Original data).

## Appendix Supplementary materials

Image, application 1

Image, application 2

Image, application 3

Image, application 4

Image, application 5

Image, application 6

Image, application 7

Image, application 8

Image, application 9

Image, application 10

Image, application 11

Image, application 12

Image, application 13
