# Supplementary material for: Geochemistry and X-ray diffraction data from rock salts and saltwork wastes of Canada: data compilation
Source: Data Brief. 2026 Jun 6;67:112941. doi: 10.1016/j.dib.2026.112941 (PMC13292661; doi:10.1016/j.dib.2026.112941)
Supplement: Supplementary file 5 [file mmc5.docx]

# **APPENDIX** **F** REPORT ON FIELD SAMPLING

####

#### ALLAN POTASH MINE, Saskatchewan Potash Corporation, NUTRIEN Ltd.

Sampling of Tailings Management Area (TMA)

GSC/NRCan: Pavel Kabanov and Stephen Grasby

Nutrien: Curtis Ferguson and Chantel Coburn

Date of visit: September 05, 2024


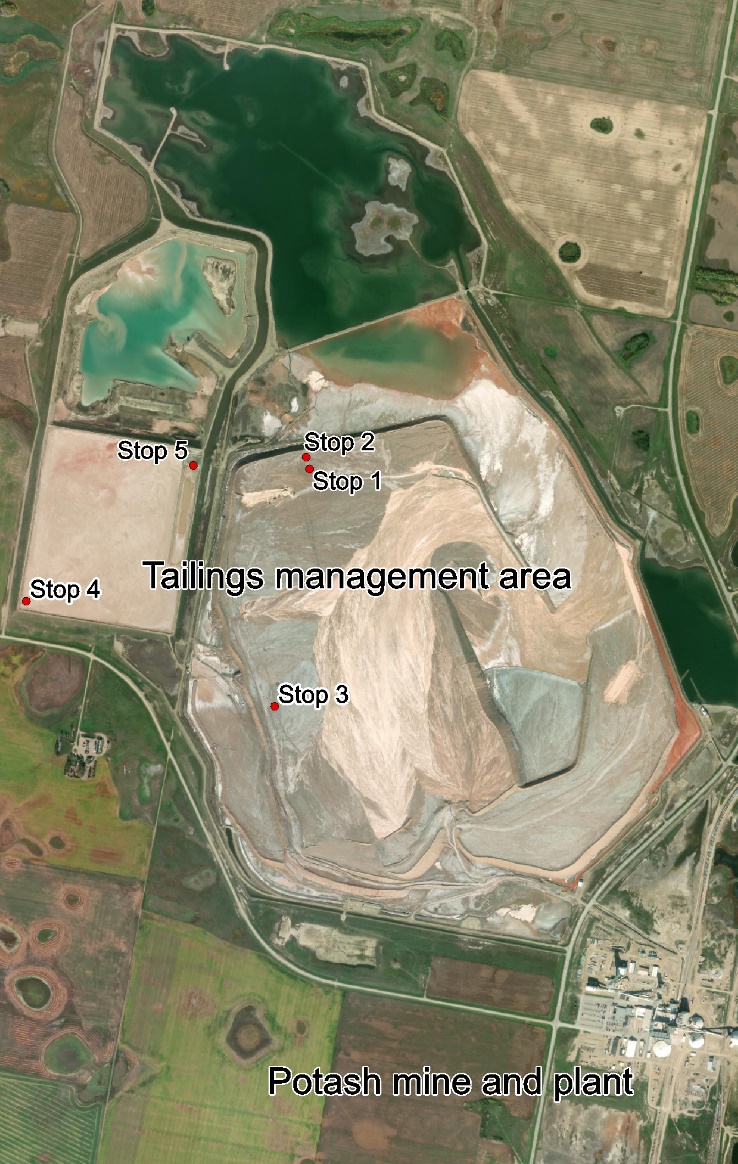
Samples have been taken at five stops within the Allan Mine TMA (Fig. 1). Solid precipitates of different induration (reflecting the age of deposition) were collected at Stops 1-4 (Figs. 2-5). Brine was accessed at Stop 5 where primary pond of plant-derived brine outflows into a broader pond. Location (degrees decimals, NAD83), description, and other essential information on each sample are provided in supplementary Table 1 (solids) and Table 2 (brines).

**Figure 1.** Sampling stops at Allan Potash TMA


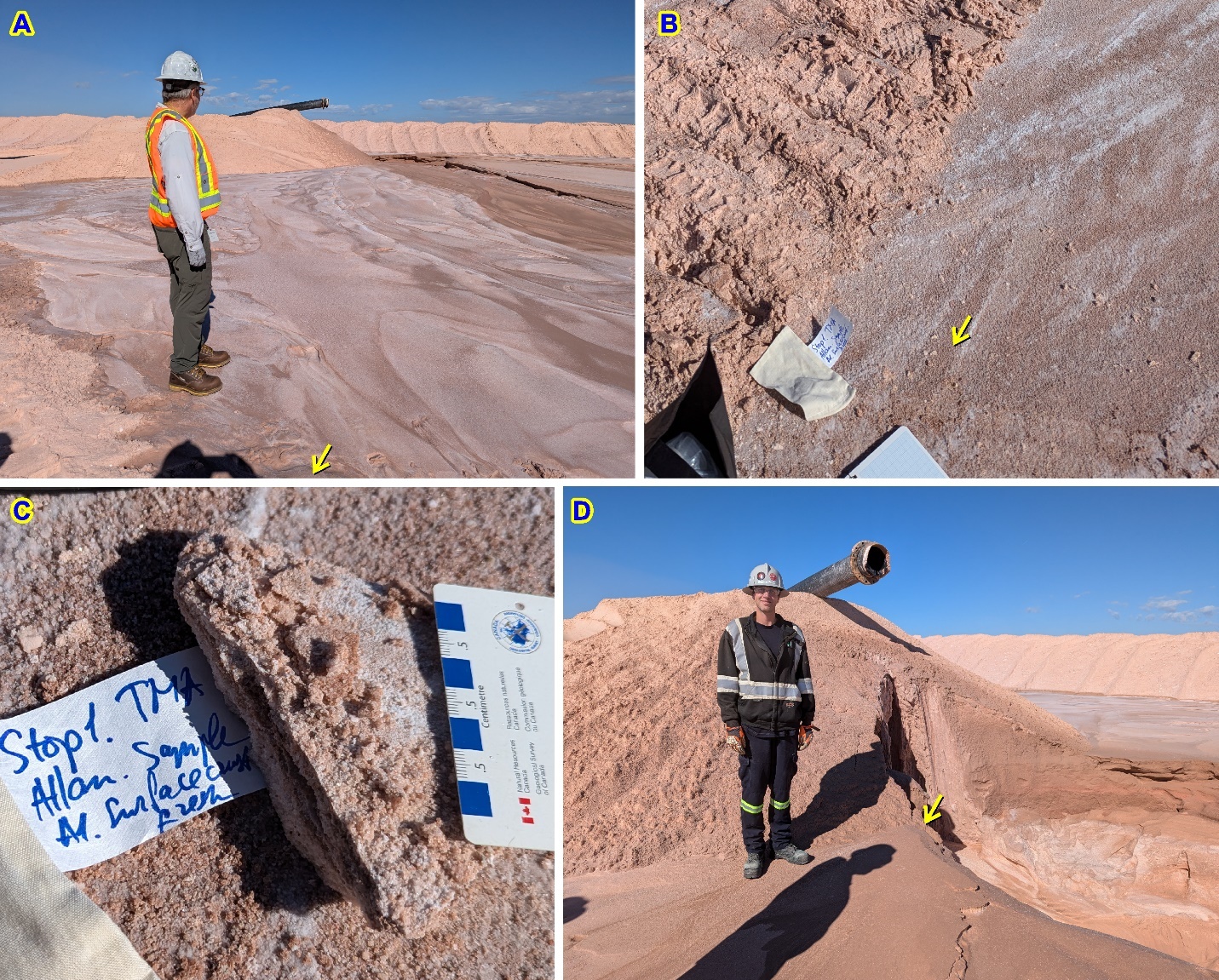


**Figure 2.** Allan Potash TMA: (A-C) Recent deposit at Stop1; arrows on (A, B) locate sample 1; (C) is the actual piece of surface crust taken as Sample 1 at Stop 1. (D) Stop 2. Fresh salt deposit under tailings deposition pipeline; arrow points at sampling spot.


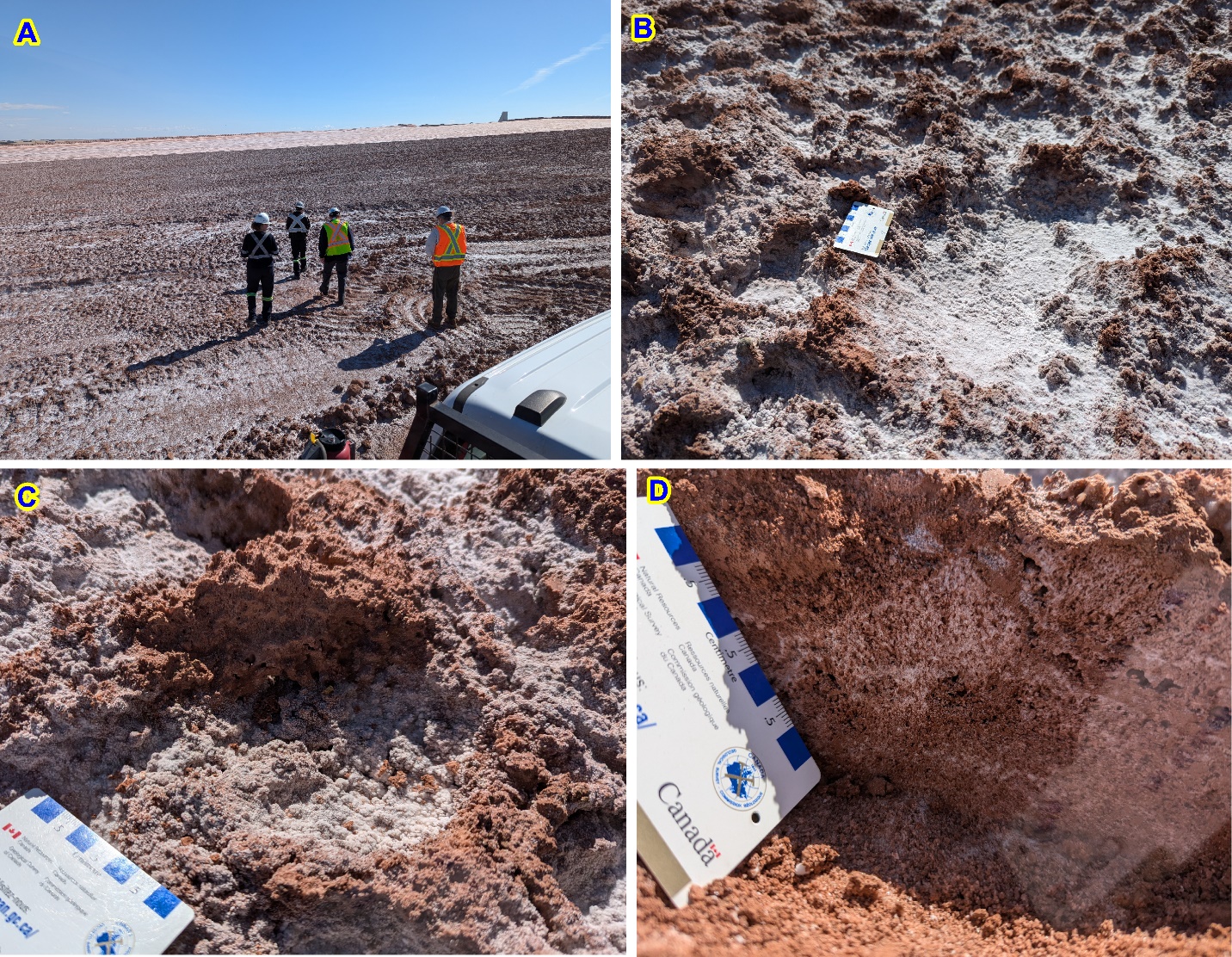


**Figure 3.** Allan Potash TMA, Stop 3. Decadal-old deposit: (A) general view; (B, C) close-up views of the surface with ridges and spurs of brown material (Stop 3, sample 2) and salt-encrusted depressions (sauce pans) between ridges. Sample 1 was collected predominantly from saucepan salt crusts. (D) knife cut of the brown ridge.

**
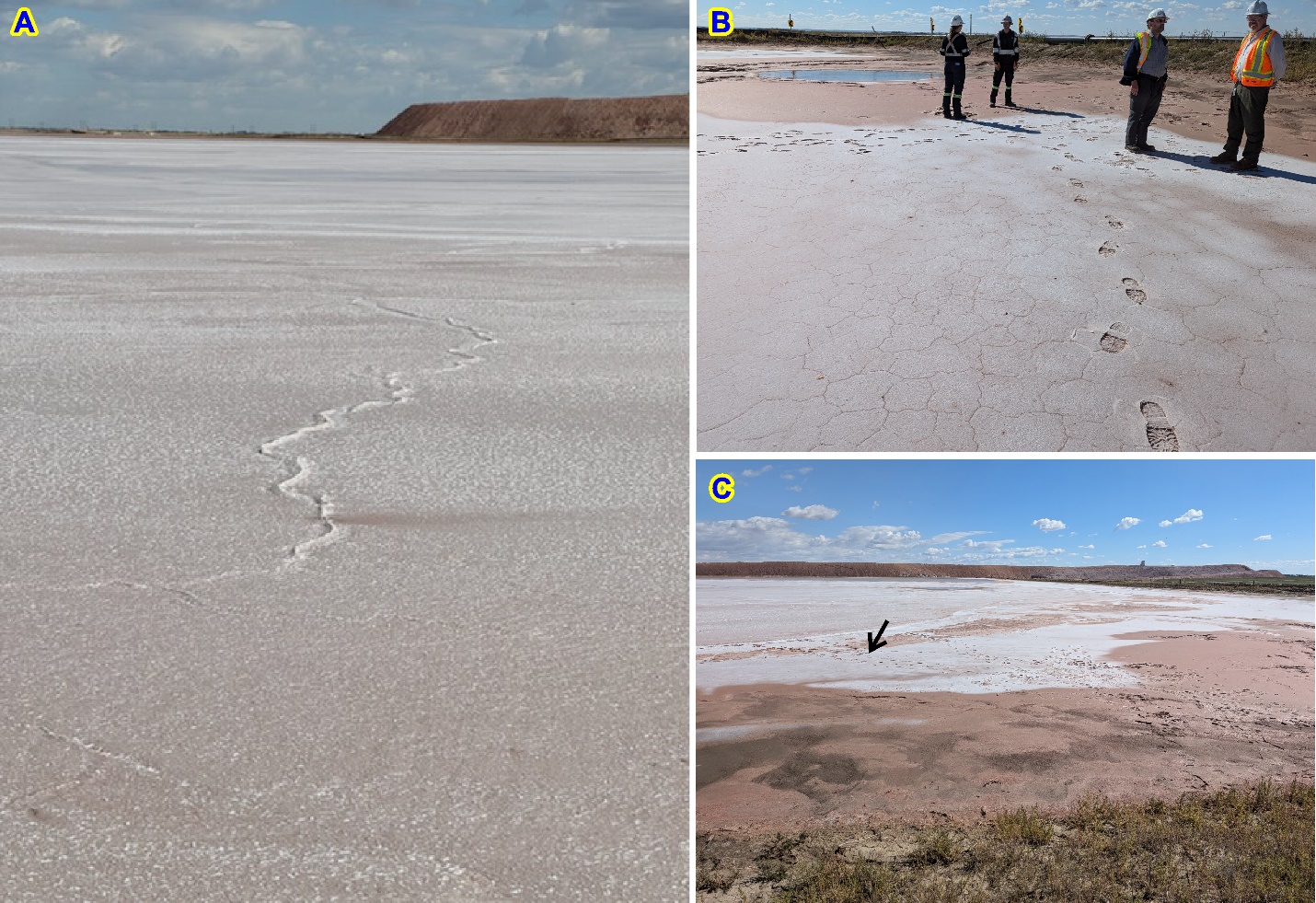
**

**Figure 4.** Allan Potash TMA, Stop 4. Clay slurry tailing, FTMA Sell 1. (A) view from the sampling point at salt flat; note salt ridges originated from expanded salt crust. (B) Access point at the margin of salt flat; note desiccation polygons formed through shrinkage of sub-crust “mud”. (C) Sampling point (arrow) near the margin of salt flat.

**
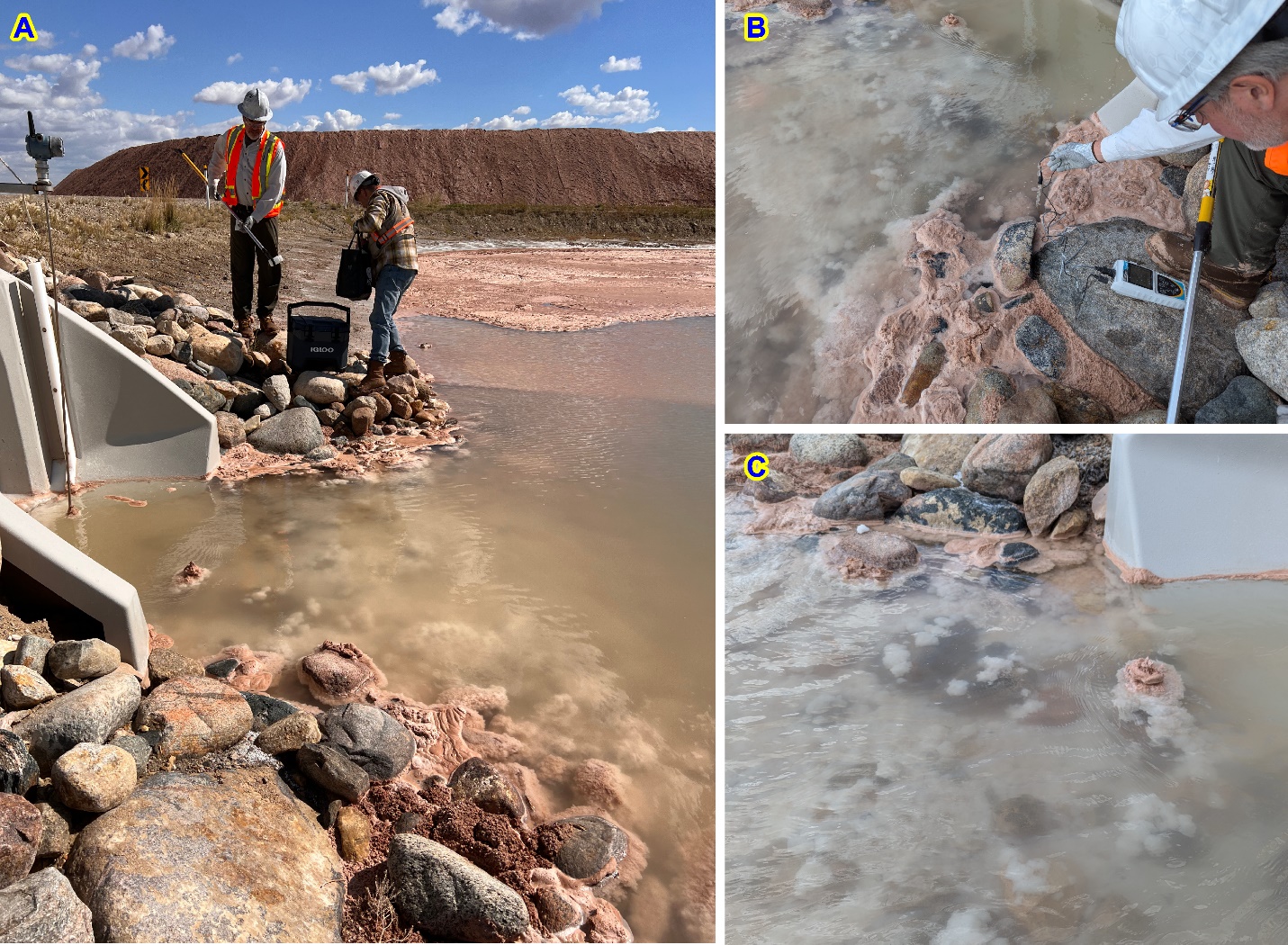
**

**Figure 5**. Allan Potash TMA, Stop 5. Brine and benthic precipitate sampling at pond outflow pipe. (A) Sampling in action. (B) pH, conductivity, TDS measurements of the brine. (C) Closer view at the outflow with crystal precipitates at bottom.

#### Fort Kent Geothermal Waste Cavern Facility, PURE ENVIRONMENTAL Ltd.

Brine sampling from a cavern making site

GSC/NRCan: Pavel Kabanov

Arranger: Piotr Kukiałka

Facility representatives : Brad Healey (manager), Shawn Lynch

Date of visit: September 06, 2024

The Fort Kent facility of Pure Environmental Ltd. emplaces and operates waste disposal caverns in the upper Lotsberg halite (Fig. 6). The facility accepts AER-regulated solid and liquid oilfield waste, imported non-hazardous recyclable waste, and Alberta generated non-oilfield waste including process water, wash fluids and hydrovac slurries (https://pureenviro.eco/facilities/fort-kent.html). The Facility utilises wastewater for cavern making and waste disposal, mostly produced water from oilsands SAGD operations. On September 06, we sampled brine from Cavern # 1 and Cavern #2 (UWI: 102/09-14-063-04W). At that date both caverns were at the stage of expansion by solution mining. Brine returning from caverns mixes solutes from the upper Lotsberg halite unit and produced water, thus its mineral composition does not represent the Lotsberg alone. The brine is being disposed to the porous dolostone of the Keg River Formation (injection well, Fig. 6B). Three samples of produced water from SAGD were sampled as well. See supplementary Excel spreadsheet for data.


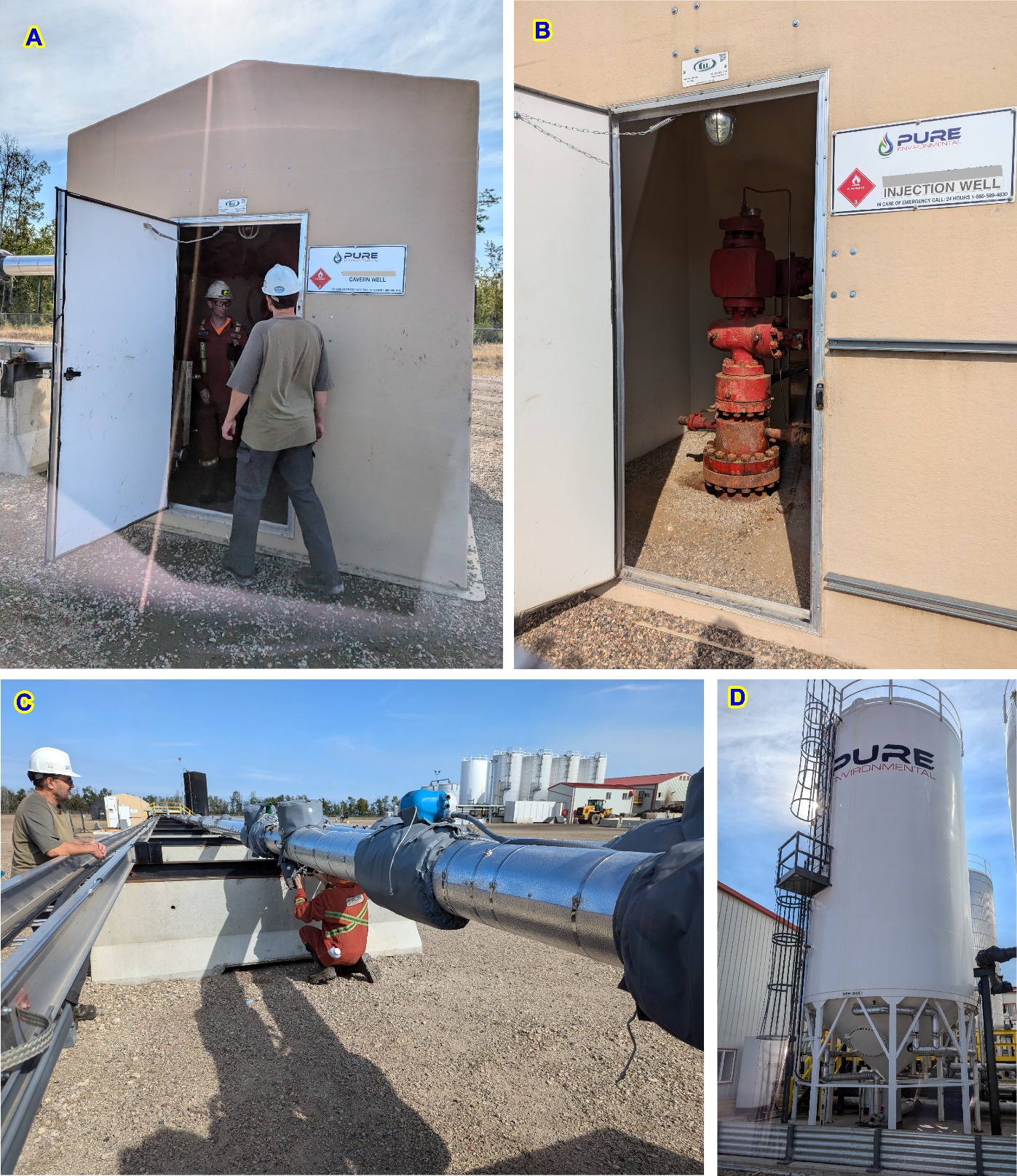


**Figure 6**. Fort Kent Geothermal Waste Cavern Facility: (A) cavern well heard; (B) brine injection well head; (C) process of brine sampling from pipe line; (D) brine tank at the plant.

#### COMPASS MINERALS TABLE SALT MINE, AMHERST, NOVA SCOTIA

Nappan Salt Mine, Compass Minerals Ltd., was visited on August 29, 2024. Samples were taken from three brine ponds (Fig. 7).

Mine representatives:

Wayne McConnell

Visitors:

Benjamin Tutolo (University of Calgary)

Peter Giles (GSC – Atlantic)

Pavel Kabanov (GSC – Calgary)


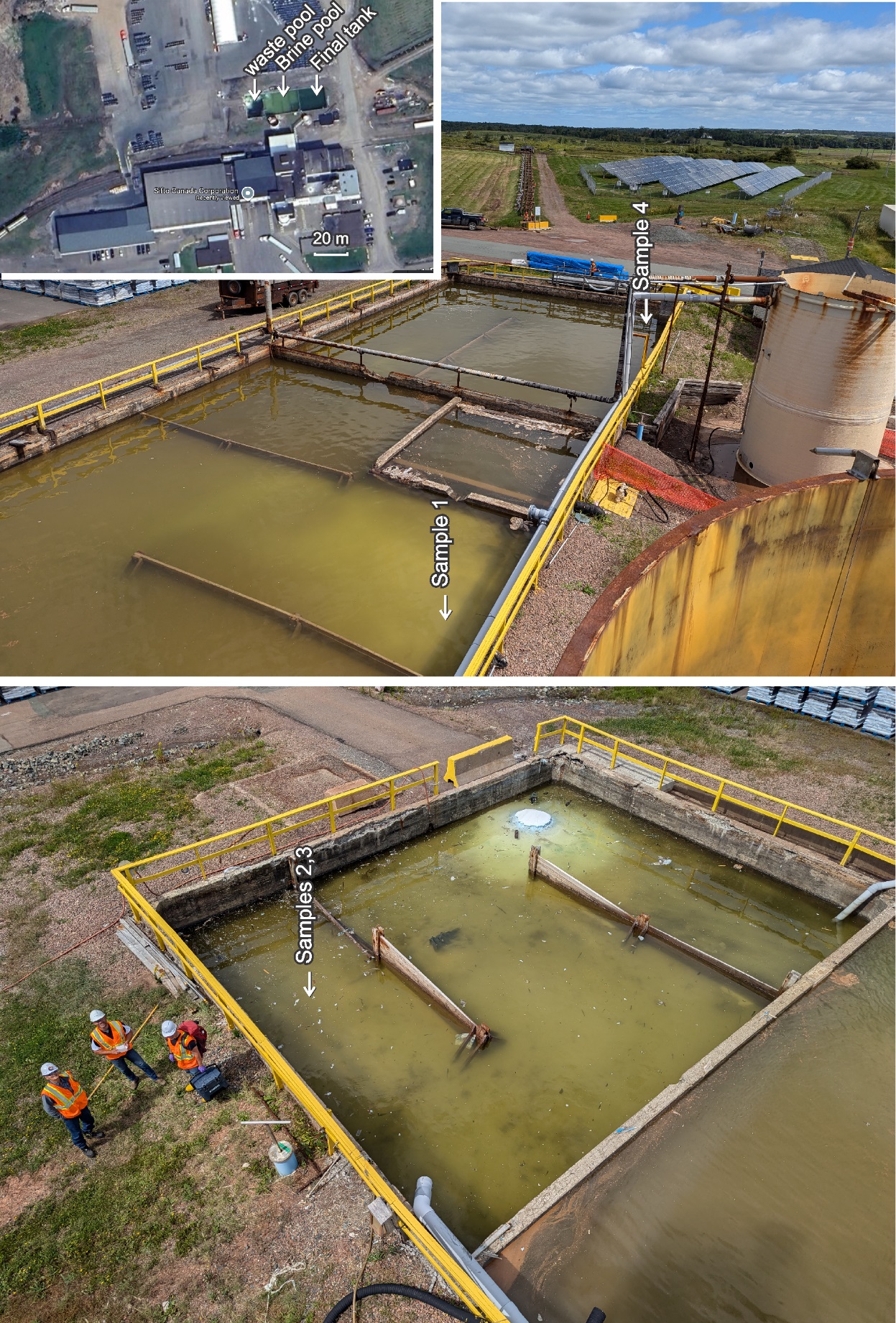


**Figure 7**. Nappan salt mine of Compass Minerals (formerly SIFTO Inc.) showing location of brine samples. Brine pools are labelled on satellite image (upper left). Images from 29-08-2024.

**Sample 1 taken from brine pool**

- 125 mL filtered (anions)
- 125 mL filtered (raw)
- ~124 mL filtered for cations (to be acidified at days’ end)

Temp = 20.9C

pH = 6.90

EC = 166.7 ppt

**Sample 2 taken from dilute upper layer of waste pond**

- 125 mL filtered (anions)
- 125 mL filtered (raw)
- 125 mL filtered for cations (to be acidified at days’ end)

Temp = 27.5C

pH = 6.6 – 6.87

EC =113.1 ppt

**Sample 3 taken from lower, denser brine in waste pond**

- 125 mL filtered (anions)
- 125 mL filtered (raw)
- 125 mL filtered for cations (to be acidified at days’ end)

pH = 6.79

Temp = 22.8C

EC = 124.6 ppt

**Sample #4 taken from corner at final tank**

- 125 mL filtered (anions)
- 125 mL filtered (raw)
- Anion = 2x 50 mL Falcon tubes

pH = 7.20

T=30.3

EC = 154.5 ppt
