# Supplementary material for: Geochemistry and X-ray diffraction data from rock salts and saltwork wastes of Canada: data compilation
Source: Data Brief. 2026 Jun 6;67:112941. doi: 10.1016/j.dib.2026.112941 (PMC13292661; doi:10.1016/j.dib.2026.112941)
Supplement: Supplementary file 7 [file mmc7.pdf]

**Report No.:**	**A23-17837**
**Report Date:**	**10-Jan-24**
**Date Submitted:**	**04-Dec-23**
**Your Reference:**	**GNES-H2GS**

Geological Survey of Canada
3303 33rd St NW
Calgary Alberta T2-2A7
Canada

ATTN: Pavel Kabanov

CERTIFICATE OF ANALYSIS

164 Pulp samples were submitted for analysis.

The following analytical package(s) were requested:		Testing Date:
1F2	QOP Total (Total Digestion ICPOES)	2023-12-13 09:07:43

REPORT **A23-17837**

This report may be reproduced without our consent. If only selected portions of the report are reproduced, permission must be obtained. If no instructions were given at time of sample submittal regarding excess material, it will be discarded within 90 days of this report. Our liability is limited solely to the analytical cost of these analyses. Test results are representative only of material submitted for analysis.

Notes:

Values which exceed the upper limit should be assayed for accurate numbers.

Refer to the Scope of Accreditation for information on accredited elements.

SCC Accredited LAB
LAB Accrédité CCN TM

LabID: 266

CERTIFIED BY:

Mark Vandergeest
Quality Control Coordinator

ACTIVATION LABORATORIES LTD.
41 Bittern Street, Ancaster, Ontario, Canada, L9G 4V5
TELEPHONE +905 648-9611 or +1.888.228.5227 FAX +1.905.648.9613
E-MAIL Ancaster@actlabs.com ACTLABS GROUP WEBSITE www.actlabs.com

Analyte Symbol	Ag	Al	As	Ba	Be	Bi	Ca	Cd	Co	Cr	Cu	Fe	Ga	K	Mg	Li	Mn	Mo	Na	Ni	P	Pb	Sb
Unit Symbol	ppm	%	ppm	ppm	ppm	ppm	%	ppm	ppm	ppm	ppm	%	ppm	%	%	ppm	ppm	ppm	%	ppm	%	ppm	ppm
Lower Limit	0.3	0.01	3	7	1	2	0.01	0.3	1	1	1	0.01	1	0.01	0.01	1	1	1	0.01	1	0.001	3	5
Method Code	TD-ICP	TD-ICP	TD-ICP	TD-ICP	TD-ICP	TD-ICP	TD-ICP	TD-ICP	TD-ICP	TD-ICP	TD-ICP	TD-ICP	TD-ICP	TD-ICP	TD-ICP	TD-ICP	TD-ICP	TD-ICP	TD-ICP	TD-ICP	TD-ICP	TD-ICP	TD-ICP
C-644531	< 0.3	5.25	7	98	< 1	< 2	16.0	< 0.3	9	47	21	2.15	11	2.56	1.97	35	310	4	0.12	33	0.020	77	< 5
C-644532	< 0.3	7.16	32	307	2	< 2	1.51	< 0.3	20	57	55	2.75	17	4.48	1.60	63	331	13	4.47	52	0.173	70	< 5
C-644534	< 0.3	3.91	4	95	1	< 2	9.82	< 0.3	7	42	82	2.06	10	2.38	4.05	50	231	1	1.18	20	0.049	4	< 5
C-644535	< 0.3	4.17	4	93	< 1	< 2	11.2	< 0.3	7	43	156	1.95	11	2.46	2.97	43	175	1	0.73	19	0.066	4	< 5
C-644536	< 0.3	2.62	< 3	73	< 1	< 2	17.7	< 0.3	6	46	7	1.24	7	2.12	2.75	45	145	< 1	0.24	16	0.028	< 3	< 5
C-644537	< 0.3	2.17	< 3	62	< 1	< 2	12.9	< 0.3	5	48	6	1.03	5	1.77	3.00	42	137	< 1	0.14	13	0.028	< 3	< 5
C-644538	< 0.3	6.39	< 3	126	2	< 2	4.32	< 0.3	9	60	35	4.32	19	2.44	7.93	347	201	< 1	0.60	36	0.048	< 3	< 5
C-644540	< 0.3	3.02	< 3	142	< 1	< 2	6.36	< 0.3	5	43	3	1.27	7	2.49	4.06	51	119	< 1	> 10.0	14	0.032	< 3	< 5
C-644541	< 0.3	0.02	< 3	< 7	< 1	< 2	0.08	< 0.3	< 1	2	< 1	0.01	< 1	0.07	0.02	< 1	29	< 1	> 10.0	< 1	< 0.001	< 3	< 5
C-644542	< 0.3	< 0.01	< 3	< 7	< 1	< 2	0.05	< 0.3	< 1	16	< 1	0.01	< 1	0.27	0.02	< 1	7	< 1	> 10.0	< 1	< 0.001	< 3	< 5
C-644543	< 0.3	0.49	< 3	21	< 1	< 2	1.13	< 0.3	< 1	18	1	0.21	< 1	0.52	0.66	11	37	< 1	> 10.0	2	0.005	< 3	< 5
C-644544	< 0.3	2.46	< 3	116	< 1	< 2	8.42	< 0.3	4	31	9	0.99	6	2.25	4.42	47	88	< 1	> 10.0	12	0.025	4	< 5
C-644545	< 0.3	0.07	< 3	< 7	< 1	< 2	0.39	< 0.3	1	16	3	0.04	< 1	0.33	0.10	2	11	< 1	> 10.0	2	< 0.001	< 3	< 5
C-644546	< 0.3	< 0.01	< 3	< 7	< 1	< 2	0.25	< 0.3	< 1	16	< 1	< 0.01	< 1	0.14	0.02	< 1	11	< 1	> 10.0	< 1	< 0.001	< 3	< 5
C-644547	< 0.3	0.03	< 3	17	< 1	< 2	0.22	< 0.3	< 1	7	1	0.02	< 1	0.06	0.03	< 1	10	< 1	> 10.0	< 1	< 0.001	< 3	< 5
C-644548	< 0.3	< 0.01	< 3	< 7	< 1	< 2	0.38	< 0.3	< 1	26	< 1	< 0.01	< 1	0.15	0.01	< 1	17	< 1	> 10.0	< 1	< 0.001	< 3	< 5
C-644549	< 0.3	0.02	< 3	65	< 1	< 2	0.09	< 0.3	< 1	3	3	0.02	< 1	0.31	0.01	< 1	22	< 1	> 10.0	< 1	< 0.001	< 3	< 5
C-644551	< 0.3	< 0.01	< 3	< 7	< 1	< 2	0.09	< 0.3	< 1	26	2	< 0.01	< 1	0.16	< 0.01	< 1	14	< 1	> 10.0	< 1	< 0.001	< 3	< 5
C-644552	< 0.3	< 0.01	< 3	< 7	< 1	< 2	0.08	< 0.3	< 1	19	< 1	< 0.01	< 1	0.06	< 0.01	< 1	14	< 1	> 10.0	< 1	< 0.001	< 3	< 5
C-644553	< 0.3	1.44	3	38	< 1	< 2	5.78	< 0.3	4	9	19	0.64	3	1.42	3.81	39	75	5	> 10.0	8	0.015	< 3	< 5
C-644554	< 0.3	0.49	< 3	16	< 1	< 2	2.37	< 0.3	< 1	16	3	0.19	< 1	0.34	0.39	17	23	< 1	> 10.0	2	0.005	< 3	< 5
C-644556	< 0.3	0.06	< 3	< 7	< 1	< 2	0.12	< 0.3	< 1	3	5	0.03	< 1	0.11	0.07	2	4	< 1	> 10.0	1	< 0.001	< 3	< 5
C-644557	< 0.3	0.02	< 3	24	< 1	< 2	0.22	< 0.3	< 1	3	< 1	< 0.01	< 1	0.17	0.03	< 1	5	< 1	> 10.0	< 1	< 0.001	< 3	< 5
C-644558	< 0.3	0.03	< 3	< 7	< 1	< 2	0.13	< 0.3	< 1	9	< 1	0.02	< 1	0.14	0.07	< 1	5	< 1	> 10.0	< 1	< 0.001	< 3	< 5
C-644559	< 0.3	0.03	< 3	67	< 1	< 2	0.33	< 0.3	< 1	2	< 1	0.01	< 1	0.22	0.02	< 1	6	< 1	> 10.0	< 1	< 0.001	< 3	< 5
C-644560	< 0.3	< 0.01	< 3	< 7	< 1	< 2	0.22	< 0.3	< 1	2	< 1	< 0.01	< 1	0.11	< 0.01	< 1	3	< 1	> 10.0	< 1	< 0.001	< 3	< 5
C-644561	< 0.3	0.05	< 3	92	< 1	< 2	0.13	< 0.3	< 1	3	< 1	0.01	< 1	0.35	0.02	< 1	3	< 1	> 10.0	< 1	< 0.001	< 3	< 5
C-644562	< 0.3	2.86	< 3	109	< 1	< 2	5.70	< 0.3	5	22	12	1.25	8	2.34	3.76	63	117	< 1	> 10.0	15	0.032	5	< 5
C-644563	< 0.3	0.06	4	40	< 1	< 2	0.54	< 0.3	< 1	3	3	0.01	< 1	0.28	0.02	< 1	8	< 1	> 10.0	< 1	< 0.001	3	< 5
C-644564	< 0.3	6.95	17	192	2	< 2	3.24	< 0.3	18	51	35	3.28	20	2.75	3.13	135	206	5	2.87	42	0.058	22	< 5
C-644565	< 0.3	0.01	< 3	15	< 1	< 2	0.04	< 0.3	< 1	4	< 1	< 0.01	< 1	0.15	< 0.01	< 1	6	< 1	> 10.0	< 1	< 0.001	< 3	< 5
C-644566	< 0.3	0.02	< 3	< 7	< 1	< 2	0.25	< 0.3	< 1	1	2	0.01	< 1	0.06	0.04	< 1	6	< 1	> 10.0	< 1	< 0.001	< 3	< 5
C-644567	< 0.3	0.03	< 3	21	< 1	< 2	0.32	< 0.3	< 1	2	2	0.02	< 1	0.13	0.07	< 1	4	< 1	> 10.0	< 1	< 0.001	< 3	< 5
C-644568	< 0.3	0.57	< 3	19	< 1	< 2	2.01	< 0.3	1	8	4	0.26	1	0.45	0.85	14	28	< 1	> 10.0	3	0.004	< 3	< 5
C-644569	< 0.3	3.81	4	148	< 1	< 2	9.11	< 0.3	7	23	14	1.75	10	3.01	3.19	99	155	1	8.35	23	0.025	< 3	< 5
C-644570	< 0.3	4.44	< 3	144	1	< 2	8.82	< 0.3	7	38	7	2.04	12	2.53	4.92	110	216	< 1	3.56	20	0.029	< 3	< 5
C-644571	< 0.3	4.46	9	64	1	< 2	11.1	< 0.3	11	26	22	1.91	12	2.09	3.58	103	138	< 1	0.79	24	0.027	4	< 5
C-644572	< 0.3	< 0.01	< 3	< 7	< 1	< 2	0.03	< 0.3	< 1	3	1	< 0.01	< 1	0.08	< 0.01	< 1	5	< 1	> 10.0	< 1	< 0.001	< 3	< 5
C-644574	< 0.3	0.04	< 3	32	< 1	< 2	0.24	< 0.3	< 1	2	< 1	0.02	< 1	0.13	0.09	1	7	< 1	> 10.0	< 1	< 0.001	< 3	< 5
C-644575	< 0.3	0.08	< 3	< 7	< 1	< 2	0.47	< 0.3	< 1	15	1	0.04	< 1	0.10	0.08	3	9	< 1	> 10.0	< 1	< 0.001	< 3	< 5
C-644576	< 0.3	0.02	< 3	< 7	< 1	< 2	1.39	< 0.3	< 1	3	< 1	0.01	< 1	0.13	0.05	< 1	4	< 1	> 10.0	< 1	< 0.001	< 3	< 5
C-644577	< 0.3	< 0.01	< 3	< 7	< 1	< 2	4.77	< 0.3	< 1	2	3	0.02	< 1	0.07	0.14	< 1	7	< 1	> 10.0	< 1	< 0.001	< 3	< 5
C-644578	< 0.3	0.02	< 3	23	< 1	< 2	0.31	< 0.3	< 1	4	< 1	< 0.01	< 1	0.16	0.01	< 1	3	< 1	> 10.0	< 1	< 0.001	< 3	< 5
C-644579	< 0.3	3.32	3	120	< 1	< 2	11.8	< 0.3	6	41	9	1.63	9	2.39	6.50	74	224	< 1	0.77	16	0.030	3	< 5
C-644580	< 0.3	3.39	< 3	137	< 1	< 2	13.1	< 0.3	5	26	4	1.36	9	2.33	1.98	62	167	< 1	5.41	13	0.021	< 3	< 5
C-644581	< 0.3	5.73	10	179	1	< 2	6.91	< 0.3	16	51	34	2.53	17	3.06	2.43	104	216	< 1	3.65	38	0.018	17	< 5
C-644582	< 0.3	7.08	6	208	2	< 2	8.87	< 0.3	9	51	7	2.84	18	4.79	2.45	117	225	< 1	1.99	32	0.027	< 3	< 5
C-644583	< 0.3	0.05	< 3	28	< 1	< 2	0.39	< 0.3	< 1	3	15	0.01	< 1	0.12	0.03	< 1	4	< 1	> 10.0	< 1	< 0.001	< 3	< 5
C-644584	< 0.3	2.03	4	85	< 1	< 2	19.2	< 0.3	5	22	11	1.24	5	1.52	5.59	43	263	2	1.40	13	0.020	9	< 5
C-644585	< 0.3	0.34	< 3	18	< 1	< 2	1.84	< 0.3	< 1	4	< 1	0.19	< 1	0.27	0.74	11	32	< 1	> 10.0	2	0.004	< 3	< 5
C-644586	< 0.3	1.86	< 3	43	< 1	< 2	11.5	< 0.3	4	25	4	1.01	4	0.93	2.16	74	92	< 1	9.76	9	0.012	< 3	< 5

Analyte Symbol	Ag	Al	As	Ba	Be	Bi	Ca	Cd	Co	Cr	Cu	Fe	Ga	K	Mg	Li	Mn	Mo	Na	Ni	P	Pb	Sb
Unit Symbol	ppm	%	ppm	ppm	ppm	ppm	%	ppm	ppm	ppm	ppm	%	ppm	%	%	ppm	ppm	ppm	%	ppm	%	ppm	ppm
Lower Limit	0.3	0.01	3	7	1	2	0.01	0.3	1	1	1	0.01	1	0.01	0.01	1	1	1	0.01	1	0.001	3	5
Method Code	TD-ICP	TD-ICP	TD-ICP	TD-ICP	TD-ICP	TD-ICP	TD-ICP	TD-ICP	TD-ICP	TD-ICP	TD-ICP	TD-ICP	TD-ICP	TD-ICP	TD-ICP	TD-ICP	TD-ICP	TD-ICP	TD-ICP	TD-ICP	TD-ICP	TD-ICP	TD-ICP
C-644587	< 0.3	4.84	8	132	1	< 2	19.1	< 0.3	8	34	9	2.01	13	2.06	2.07	85	211	< 1	0.32	22	0.031	5	< 5
C-644588	< 0.3	3.43	< 3	94	< 1	< 2	19.8	< 0.3	4	20	4	1.34	9	2.10	1.44	65	152	< 1	4.59	13	0.027	< 3	< 5
C-644589	< 0.3	2.95	5	117	< 1	< 2	11.7	< 0.3	6	21	19	1.64	9	2.26	6.63	62	263	< 1	2.48	17	0.031	< 3	< 5
C-644590	< 0.3	2.92	< 3	93	< 1	< 2	9.24	< 0.3	10	19	14	1.57	7	1.93	4.95	56	314	< 1	8.82	19	0.014	9	< 5
C-644591	< 0.3	0.95	< 3	28	< 1	< 2	1.49	< 0.3	1	7	12	0.35	2	0.64	0.47	22	39	< 1	> 10.0	5	0.008	< 3	< 5
C-644592	< 0.3	4.14	< 3	123	< 1	< 2	11.8	< 0.3	6	18	10	2.04	11	2.79	5.18	74	348	< 1	1.05	17	0.031	4	< 5
C-644593	< 0.3	2.36	4	85	< 1	< 2	22.0	< 0.3	4	13	10	1.15	6	1.77	2.49	45	231	< 1	5.28	10	0.009	4	< 5
C-644594	< 0.3	4.60	< 3	139	1	< 2	12.2	< 0.3	9	26	20	2.02	12	2.77	3.09	87	274	< 1	1.68	22	0.029	9	< 5
C-644595	< 0.3	6.54	7	189	2	< 2	7.28	< 0.3	17	51	38	2.91	18	4.16	2.71	141	254	2	0.29	43	0.031	30	< 5
C-644596	< 0.3	0.07	< 3	57	< 1	< 2	0.27	< 0.3	< 1	6	1	0.02	< 1	0.07	0.03	1	7	< 1	> 10.0	< 1	< 0.001	< 3	< 5
C-644597	< 0.3	0.01	< 3	16	< 1	< 2	0.27	< 0.3	< 1	9	< 1	< 0.01	< 1	0.17	< 0.01	< 1	3	< 1	> 10.0	< 1	< 0.001	< 3	< 5
C-644598	< 0.3	0.21	< 3	35	< 1	< 2	0.27	< 0.3	< 1	3	3	0.11	< 1	0.29	0.20	9	13	< 1	> 10.0	2	0.001	< 3	< 5
C-644599	< 0.3	0.02	< 3	< 7	< 1	< 2	0.12	< 0.3	< 1	2	< 1	0.02	< 1	0.07	0.03	< 1	5	< 1	> 10.0	< 1	< 0.001	< 3	< 5
C-644600	< 0.3	3.72	< 3	103	< 1	< 2	21.9	< 0.3	8	28	12	1.48	10	2.22	1.70	92	195	< 1	0.58	19	0.021	7	< 5
C-644601	< 0.3	1.30	< 3	46	< 1	< 2	19.9	< 0.3	2	12	3	0.53	4	0.93	0.72	47	85	< 1	3.11	6	0.015	< 3	< 5
C-644602	< 0.3	0.03	< 3	68	< 1	< 2	0.16	< 0.3	< 1	3	< 1	0.02	< 1	0.16	0.02	< 1	5	< 1	> 10.0	< 1	< 0.001	< 3	< 5
C-644603	< 0.3	1.34	< 3	73	< 1	< 2	4.57	< 0.3	3	10	4	0.71	3	0.69	2.99	84	121	< 1	> 10.0	7	0.021	< 3	< 5
C-644604	< 0.3	0.02	< 3	11	< 1	< 2	0.07	< 0.3	< 1	1	2	< 0.01	< 1	0.19	0.01	< 1	5	< 1	> 10.0	< 1	< 0.001	< 3	< 5
C-644605	< 0.3	< 0.01	< 3	< 7	< 1	< 2	0.14	< 0.3	< 1	< 1	< 1	< 0.01	< 1	0.06	< 0.01	< 1	4	< 1	> 10.0	< 1	< 0.001	< 3	< 5
C-644606	< 0.3	0.83	< 3	50	< 1	< 2	2.20	< 0.3	2	8	3	0.44	2	0.77	0.97	17	81	< 1	> 10.0	5	0.006	< 3	< 5
C-644607	< 0.3	5.03	< 3	134	1	< 2	6.55	< 0.3	9	31	13	1.97	13	3.13	1.54	92	161	< 1	9.59	24	0.022	7	< 5
C-644608	< 0.3	4.65	< 3	135	1	< 2	18.9	< 0.3	7	45	5	2.09	13	2.41	2.20	81	250	< 1	0.76	18	0.027	< 3	9
C-644609	< 0.3	1.60	< 3	49	< 1	< 2	5.57	< 0.3	3	10	8	0.74	4	1.28	0.83	29	95	< 1	> 10.0	7	0.005	< 3	< 5
C-644610	< 0.3	< 0.01	< 3	< 7	< 1	< 2	0.31	< 0.3	< 1	3	< 1	< 0.01	< 1	0.17	0.02	< 1	6	< 1	> 10.0	< 1	< 0.001	< 3	< 5
C-644611	< 0.3	< 0.01	< 3	< 7	< 1	< 2	0.43	< 0.3	< 1	2	< 1	< 0.01	< 1	0.06	0.01	< 1	7	< 1	> 10.0	< 1	< 0.001	< 3	< 5
C-644612	< 0.3	0.02	< 3	46	< 1	< 2	0.39	< 0.3	< 1	< 1	< 1	0.01	< 1	0.27	0.02	< 1	7	< 1	> 10.0	< 1	< 0.001	< 3	< 5
C-644613	< 0.3	3.51	< 3	97	< 1	< 2	13.7	< 0.3	5	25	7	1.57	9	2.11	2.27	92	189	< 1	3.81	16	0.024	< 3	< 5
C-644614	< 0.3	0.07	< 3	68	< 1	< 2	0.36	< 0.3	< 1	3	1	0.02	< 1	0.25	0.03	< 1	8	< 1	> 10.0	< 1	< 0.001	< 3	< 5
C-644615	< 0.3	< 0.01	< 3	< 7	< 1	< 2	0.11	< 0.3	< 1	12	< 1	< 0.01	< 1	0.14	0.01	< 1	12	< 1	> 10.0	< 1	< 0.001	< 3	< 5
C-644616	< 0.3	0.03	< 3	39	< 1	< 2	0.15	< 0.3	< 1	2	2	0.01	< 1	0.28	0.02	< 1	5	< 1	> 10.0	< 1	< 0.001	9	< 5
C-644617	< 0.3	0.11	< 3	< 7	< 1	< 2	1.00	< 0.3	< 1	9	2	0.07	< 1	0.12	0.20	3	21	< 1	> 10.0	< 1	0.002	< 3	< 5
C-644618	< 0.3	4.79	5	132	1	< 2	9.39	< 0.3	12	46	35	2.24	13	3.24	2.58	117	235	< 1	4.98	29	0.029	13	< 5
C-644619	< 0.3	3.67	< 3	121	< 1	< 2	13.1	< 0.3	8	38	15	1.48	9	2.22	2.86	83	250	< 1	3.92	17	0.026	7	< 5
C-644620	< 0.3	0.23	< 3	9	< 1	< 2	10.5	< 0.3	< 1	3	2	0.41	< 1	0.21	5.92	7	235	< 1	> 10.0	2	0.003	< 3	< 5
C-644621	< 0.3	5.05	< 3	146	1	< 2	7.73	< 0.3	8	30	10	2.15	13	3.19	4.84	169	270	< 1	2.29	24	0.027	< 3	< 5
C-644622	< 0.3	0.08	4	< 7	< 1	< 2	11.1	< 0.3	< 1	4	1	0.05	< 1	0.08	0.04	6	11	< 1	0.71	2	0.002	< 3	< 5
C-644623	< 0.3	4.95	< 3	162	1	< 2	7.30	< 0.3	7	25	12	2.10	13	2.86	4.53	167	259	< 1	3.73	22	0.035	5	< 5
C-644624	< 0.3	0.70	7	28	< 1	< 2	12.1	< 0.3	3	9	4	0.89	2	0.35	3.36	31	101	< 1	5.20	9	0.007	< 3	< 5
C-644625	< 0.3	1.74	< 3	58	< 1	< 2	8.56	< 0.3	4	15	11	0.98	5	0.99	5.17	67	194	1	> 10.0	12	0.017	7	< 5
C-644626	< 0.3	2.49	5	87	< 1	< 2	10.6	< 0.3	8	31	11	1.44	7	1.37	2.08	94	92	1	1.63	18	0.020	9	< 5
C-644627	< 0.3	2.71	< 3	92	< 1	< 2	9.17	< 0.3	5	23	8	1.46	7	1.47	4.40	87	234	< 1	8.65	13	0.024	< 3	< 5
C-644628	< 0.3	2.30	< 3	87	< 1	< 2	9.05	< 0.3	4	10	5	1.30	6	1.47	5.43	69	292	< 1	9.23	10	0.019	< 3	< 5
C-644629	< 0.3	0.88	< 3	35	< 1	< 2	30.5	< 0.3	2	8	3	0.41	3	0.48	0.82	35	149	< 1	5.87	5	0.013	< 3	< 5
C-644630	< 0.3	5.12	< 3	156	1	< 2	11.0	< 0.3	7	27	8	1.92	14	2.13	2.16	130	189	< 1	2.04	21	0.021	4	< 5
C-644631	< 0.3	0.37	< 3	15	< 1	< 2	13.8	< 0.3	2	33	6	0.16	1	0.23	0.14	11	16	< 1	0.62	4	0.004	27	< 5
C-644632	< 0.3	0.57	< 3	38	< 1	< 2	20.7	< 0.3	3	10	6	0.72	1	0.32	6.88	26	271	< 1	3.13	4	0.012	< 3	< 5
C-644633	< 0.3	1.20	< 3	58	< 1	< 2	9.09	< 0.3	2	10	7	0.68	3	0.65	6.00	55	167	< 1	> 10.0	6	0.012	4	< 5
C-644634	< 0.3	0.02	< 3	< 7	< 1	< 2	0.50	< 0.3	< 1	1	7	0.01	< 1	0.08	0.04	1	4	< 1	> 10.0	< 1	< 0.001	< 3	< 5
C-644635	< 0.3	0.16	< 3	18	< 1	< 2	1.00	< 0.3	< 1	2	< 1	0.09	< 1	0.15	0.25	7	14	< 1	> 10.0	< 1	0.002	< 3	< 5
C-644637	< 0.3	0.01	< 3	8	< 1	< 2	0.09	< 0.3	< 1	3	< 1	< 0.01	< 1	0.18	< 0.01	< 1	4	< 1	> 10.0	< 1	< 0.001	10	< 5
C-644638	< 0.3	2.83	< 3	62	< 1	< 2	7.35	< 0.3	5	24	5	1.17	7	1.48	1.37	64	90	< 1	> 10.0	13	0.015	< 3	< 5

Analyte Symbol	Ag	Al	As	Ba	Be	Bi	Ca	Cd	Co	Cr	Cu	Fe	Ga	K	Mg	Li	Mn	Mo	Na	Ni	P	Pb	Sb
Unit Symbol	ppm	%	ppm	ppm	ppm	ppm	%	ppm	ppm	ppm	ppm	%	ppm	%	%	ppm	ppm	ppm	%	ppm	%	ppm	ppm
Lower Limit	0.3	0.01	3	7	1	2	0.01	0.3	1	1	1	0.01	1	0.01	0.01	1	1	1	0.01	1	0.001	3	5
Method Code	TD-ICP	TD-ICP	TD-ICP	TD-ICP	TD-ICP	TD-ICP	TD-ICP	TD-ICP	TD-ICP	TD-ICP	TD-ICP	TD-ICP	TD-ICP	TD-ICP	TD-ICP	TD-ICP	TD-ICP	TD-ICP	TD-ICP	TD-ICP	TD-ICP	TD-ICP	TD-ICP
C-644639	< 0.3	0.27	< 3	7	< 1	< 2	1.38	< 0.3	1	3	5	0.11	< 1	0.18	0.12	7	24	< 1	> 10.0	2	0.002	< 3	< 5
C-644640	< 0.3	3.20	4	126	< 1	< 2	12.0	< 0.3	6	20	14	1.61	9	2.10	4.04	90	281	< 1	6.37	15	0.017	8	< 5
C-644641	< 0.3	< 0.01	< 3	< 7	< 1	< 2	0.58	< 0.3	< 1	1	< 1	< 0.01	< 1	0.03	0.01	< 1	8	< 1	> 10.0	< 1	< 0.001	< 3	< 5
C-644643	< 0.3	0.15	< 3	8	< 1	< 2	1.35	< 0.3	< 1	2	1	0.07	< 1	0.13	0.12	5	11	< 1	> 10.0	1	0.002	< 3	< 5
C-644644	< 0.3	1.96	10	93	< 1	< 2	10.9	< 0.3	6	20	21	0.94	5	1.04	1.51	73	101	2	> 10.0	11	0.017	7	< 5
C-666387	< 0.3	0.07	< 3	23	< 1	< 2	0.73	< 0.3	< 1	4	< 1	0.03	< 1	0.09	0.07	2	7	< 1	> 10.0	< 1	< 0.001	< 3	< 5
C-666388	< 0.3	3.51	< 3	113	< 1	< 2	20.9	< 0.3	7	37	7	1.63	9	2.02	2.43	74	370	< 1	4.02	18	0.027	< 3	< 5
C-666389	< 0.3	3.33	< 3	95	< 1	< 2	21.1	< 0.3	5	28	4	1.42	9	1.99	1.64	64	229	< 1	3.71	14	0.020	< 3	< 5
C-666390	< 0.3	6.36	8	159	1	< 2	6.58	< 0.3	10	45	17	2.98	18	2.99	4.29	169	252	1	3.86	28	0.056	11	< 5
C-666391	< 0.3	0.02	< 3	28	< 1	< 2	1.02	< 0.3	< 1	3	< 1	0.04	< 1	0.18	0.04	< 1	12	< 1	> 10.0	< 1	< 0.001	< 3	< 5
C-666392	< 0.3	0.05	< 3	< 7	< 1	< 2	0.24	< 0.3	< 1	1	1	0.02	< 1	0.17	0.03	1	7	< 1	> 10.0	< 1	< 0.001	< 3	< 5
C-666393	< 0.3	< 0.01	3	< 7	< 1	< 2	0.29	< 0.3	< 1	3	< 1	0.01	< 1	0.25	0.03	< 1	7	4	> 10.0	< 1	< 0.001	< 3	< 5
C-666394	< 0.3	0.02	< 3	< 7	< 1	< 2	0.35	< 0.3	< 1	5	< 1	0.01	< 1	0.22	0.04	< 1	6	< 1	> 10.0	< 1	< 0.001	< 3	< 5
C-666395	< 0.3	2.63	7	77	< 1	< 2	9.51	< 0.3	8	20	17	1.21	6	2.40	3.42	54	246	< 1	> 10.0	16	0.020	14	< 5
C-666396	< 0.3	< 0.01	< 3	< 7	< 1	< 2	0.08	< 0.3	< 1	5	3	< 0.01	< 1	0.18	< 0.01	< 1	3	< 1	> 10.0	< 1	< 0.001	6	< 5
C-666397	< 0.3	4.19	< 3	152	1	< 2	11.7	< 0.3	5	23	3	1.70	12	3.07	1.94	54	219	< 1	5.60	16	0.022	< 3	< 5
C-666398	< 0.3	5.47	< 3	148	1	< 2	9.03	< 0.3	7	36	5	1.79	15	2.93	1.43	58	205	< 1	6.72	22	0.021	< 3	< 5
C-666399	< 0.3	5.37	< 3	158	1	< 2	9.93	< 0.3	6	43	5	1.74	14	2.38	1.59	58	249	< 1	4.90	19	0.024	< 3	< 5
C-666400	< 0.3	0.96	< 3	44	< 1	< 2	13.9	< 0.3	1	10	3	0.76	3	0.84	7.86	24	276	< 1	5.49	3	0.012	< 3	< 5
C-666401	< 0.3	6.42	6	142	1	< 2	7.98	< 0.3	14	29	33	2.43	19	2.13	4.57	81	214	2	0.69	32	0.021	21	< 5
C-644550	< 0.3	< 0.01	< 3	16	< 1	< 2	0.07	< 0.3	< 1	8	< 1	< 0.01	< 1	0.23	< 0.01	< 1	4	< 1	> 10.0	< 1	< 0.001	< 3	< 5
C-644555	< 0.3	0.06	< 3	< 7	< 1	< 2	0.79	< 0.3	< 1	3	17	0.05	< 1	0.31	0.04	1	7	< 1	> 10.0	< 1	< 0.001	7	< 5
MTU X11-5 Gold 304.06m (98)	< 0.3	1.11	7	68	< 1	< 2	13.1	< 0.3	3	20	6	0.57	3	0.96	3.48	72	62	4	0.10	7	0.014	20	< 5
MTU X11-5 Gold 305.82m (99)	< 0.3	0.05	4	< 7	< 1	< 2	13.5	< 0.3	< 1	4	< 1	0.04	< 1	0.04	0.69	19	9	3	0.04	< 1	0.001	< 3	< 5
MTU X11-5 Gold 306.71m (99)	< 0.3	0.10	4	32	< 1	< 2	20.3	< 0.3	1	8	26	0.09	< 1	0.07	9.64	22	65	< 1	1.93	1	0.001	< 3	< 5
MTU X11-5 Gold 306.98m (99)	< 0.3	< 0.01	< 3	< 7	< 1	< 2	0.19	< 0.3	< 1	10	2	< 0.01	< 1	< 0.01	0.09	< 1	6	< 1	> 10.0	< 1	< 0.001	< 3	< 5
MTU X11-5 Gold 307.33m (100)	< 0.3	< 0.01	< 3	< 7	< 1	< 2	0.01	< 0.3	< 1	4	< 1	< 0.01	< 1	< 0.01	< 0.01	< 1	4	< 1	> 10.0	< 1	< 0.001	< 3	< 5
MTU X11-5 Gold 307.47m (100)	< 0.3	1.60	5	92	< 1	< 2	16.2	< 0.3	4	21	39	0.86	4	1.28	8.83	372	94	< 1	3.14	11	0.020	8	< 5
MTU X11-5 Gold 309.00m (100)	< 0.3	0.02	< 3	< 7	< 1	< 2	2.60	< 0.3	< 1	5	5	0.01	< 1	0.02	0.30	2	6	< 1	> 10.0	4	< 0.001	< 3	< 5
MTU X11-5 Gold 314.12m (102)	< 0.3	< 0.01	< 3	< 7	< 1	< 2	0.15	< 0.3	< 1	3	< 1	< 0.01	< 1	0.01	< 0.01	< 1	5	< 1	> 10.0	2	< 0.001	< 3	< 5
MTU X11-5 Gold 318.91m (104)	< 0.3	0.16	4	18	< 1	< 2	18.6	< 0.3	2	11	70	0.13	< 1	0.13	9.52	20	43	< 1	6.93	1	0.002	< 3	< 5
MTU X11-5 Gold 321.76m (105)	< 0.3	< 0.01	3	< 7	< 1	< 2	14.6	< 0.3	< 1	3	< 1	< 0.01	< 1	< 0.01	0.10	3	5	< 1	0.06	< 1	< 0.001	< 3	< 5
MTU X11-5 Gold 330.62m (107)	< 0.3	< 0.01	< 3	< 7	< 1	< 2	0.36	< 0.3	< 1	9	< 1	< 0.01	< 1	0.02	0.03	< 1	4	< 1	> 10.0	4	< 0.001	< 3	< 5
MTU X11-5 Gold 335.20m (109)	< 0.3	0.15	4	7	< 1	< 2	5.67	< 0.3	< 1	4	2	0.10	< 1	0.12	0.53	24	14	2	> 10.0	16	0.003	9	< 5
MTU X11-5 Gold 339.63m (111)	< 0.3	0.37	< 3	19	< 1	< 2	7.69	< 0.3	1	14	1	0.20	< 1	0.28	1.17	76	20	< 1	> 10.0	15	0.004	6	< 5
MTU X11-5 Gold 348.95m (116)	< 0.3	0.44	3	30	< 1	< 2	7.08	< 0.3	1	15	3	0.21	2	0.41	1.09	28	30	1	> 10.0	25	0.005	4	< 5
MTU X11-5 Gold 352.22m (117)	< 0.3	2.15	4	122	< 1	< 2	10.9	< 0.3	4	27	5	1.18	6	1.59	5.45	109	129	< 1	7.85	44	0.016	< 3	< 5
MTU X11-5 Gold 382.40m (133)	< 0.3	0.50	5	26	< 1	< 2	11.6	< 0.3	1	11	2	0.28	1	0.41	2.10	44	39	< 1	> 10.0	2	0.006	10	< 5
MTU X11-5 Gold	< 0.3	0.08	< 3	< 7	< 1	< 2	0.75	< 0.3	< 1	3	1	0.05	< 1	0.07	0.07	2	11	< 1	> 10.0	1	0.001	4	6

Analyte Symbol	Ag	Al	As	Ba	Be	Bi	Ca	Cd	Co	Cr	Cu	Fe	Ga	K	Mg	Li	Mn	Mo	Na	Ni	P	Pb	Sb
Unit Symbol	ppm	%	ppm	ppm	ppm	ppm	%	ppm	ppm	ppm	ppm	%	ppm	%	%	ppm	ppm	ppm	%	ppm	%	ppm	ppm
Lower Limit	0.3	0.01	3	7	1	2	0.01	0.3	1	1	1	0.01	1	0.01	0.01	1	1	1	0.01	1	0.001	3	5
Method Code	TD-ICP	TD-ICP	TD-ICP	TD-ICP	TD-ICP	TD-ICP	TD-ICP	TD-ICP	TD-ICP	TD-ICP	TD-ICP	TD-ICP	TD-ICP	TD-ICP	TD-ICP	TD-ICP	TD-ICP	TD-ICP	TD-ICP	TD-ICP	TD-ICP	TD-ICP	TD-ICP
388.40m (136)																							
MTU X11-5 Gold 395.94m (141)	< 0.3	0.04	< 3	< 7	< 1	< 2	0.13	< 0.3	< 1	5	10	0.08	< 1	0.03	0.07	1	7	< 1	> 10.0	< 1	< 0.001	< 3	< 5
MTU X11-5 Gold 433.97m (161)	< 0.3	0.94	4	52	< 1	< 2	14.3	< 0.3	2	11	3	0.39	3	0.70	4.72	88	81	2	5.15	4	0.009	< 3	< 5
MTU X11-5 Gold 434.26m (161)	< 0.3	0.04	< 3	< 7	< 1	< 2	0.64	< 0.3	< 1	2	< 1	0.02	< 1	0.04	0.10	4	8	< 1	> 10.0	1	< 0.001	< 3	< 5
MTU X11-5 Gold 437.25m (163)	< 0.3	0.06	< 3	< 7	< 1	< 2	1.79	< 0.3	< 1	18	< 1	0.03	< 1	0.05	0.32	7	11	2	> 10.0	3	< 0.001	< 3	< 5
MTU X11-5 Gold 439.96m (164)	< 0.3	0.04	< 3	< 7	< 1	< 2	1.10	< 0.3	< 1	19	< 1	0.02	< 1	0.04	0.23	3	13	< 1	> 10.0	2	< 0.001	< 3	< 5
MTU X11-5 Gold 443.80m (166)	< 0.3	< 0.01	< 3	< 7	< 1	< 2	0.89	< 0.3	< 1	8	2	< 0.01	< 1	0.01	0.05	< 1	8	< 1	> 10.0	< 1	< 0.001	< 3	< 5
MTU X11-5 Gold 447.32m (168)	< 0.3	0.04	6	< 7	< 1	< 2	0.35	< 0.3	< 1	8	3	0.03	< 1	0.05	0.12	2	6	< 1	> 10.0	1	< 0.001	< 3	< 5
MTU X11-5 Gold 450.85m (170)	< 0.3	0.03	< 3	< 7	< 1	< 2	1.04	< 0.3	< 1	7	4	0.02	< 1	0.04	0.16	3	6	< 1	> 10.0	4	< 0.001	< 3	< 5
MTU X11-5 Gold 455.79m (173)	< 0.3	< 0.01	15	< 7	< 1	< 2	0.30	< 0.3	< 1	3	1	< 0.01	< 1	0.02	0.04	< 1	8	2	> 10.0	< 1	< 0.001	< 3	< 5
MTU X11-5 Gold 457.45m (174)	< 0.3	< 0.01	5	< 7	< 1	< 2	0.56	< 0.3	< 1	4	1	< 0.01	< 1	0.01	0.03	< 1	6	1	> 10.0	< 1	< 0.001	< 3	< 5
MTU X11-5 Gold 459.28m (175)	< 0.3	0.03	< 3	< 7	< 1	< 2	0.69	< 0.3	< 1	1	11	0.02	< 1	0.03	0.08	1	12	1	> 10.0	< 1	< 0.001	< 3	< 5
MTU X11-5 Gold 459.89m (175)	< 0.3	0.02	< 3	< 7	< 1	< 2	0.07	< 0.3	< 1	3	3	0.03	< 1	0.03	0.04	1	3	< 1	> 10.0	< 1	< 0.001	< 3	< 5
MTU X11-5 Gold 463.76m (178)	< 0.3	< 0.01	< 3	< 7	< 1	< 2	1.04	< 0.3	< 1	1	< 1	< 0.01	< 1	0.01	0.05	< 1	6	< 1	> 10.0	< 1	< 0.001	< 3	< 5
MTU X11-5 Gold 468.36m (180)	< 0.3	0.02	< 3	< 7	< 1	< 2	2.27	< 0.3	< 1	< 1	< 1	0.01	< 1	0.03	0.17	3	7	< 1	> 10.0	2	< 0.001	< 3	< 5
MTU X11-5 Gold 475.37m (184)	< 0.3	0.07	< 3	14	< 1	< 2	4.10	< 0.3	< 1	10	1	0.05	< 1	0.07	0.57	33	12	2	> 10.0	5	0.001	< 3	< 5
MTU X11-5 Gold 479.52m (186)	< 0.3	< 0.01	< 3	< 7	< 1	< 2	0.57	< 0.3	< 1	1	< 1	< 0.01	< 1	0.02	0.04	2	5	< 1	> 10.0	2	< 0.001	< 3	< 5
MTU X11-5 Gold 492.48m (192)	< 0.3	< 0.01	< 3	< 7	< 1	< 2	0.02	< 0.3	< 1	< 1	< 1	< 0.01	< 1	< 0.01	0.02	< 1	3	< 1	> 10.0	< 1	< 0.001	< 3	< 5
89-102-V 2.74m (1)	< 0.3	0.01	3	< 7	< 1	< 2	3.10	< 0.3	< 1	3	< 1	0.01	< 1	0.02	0.09	< 1	10	< 1	> 10.0	< 1	< 0.001	< 3	< 5
89-102-V 5.40m (2)	< 0.3	< 0.01	< 3	< 7	< 1	< 2	1.05	< 0.3	< 1	9	< 1	< 0.01	< 1	0.01	0.03	< 1	4	< 1	> 10.0	< 1	< 0.001	< 3	< 5
89-102-V 11.49m (3)	< 0.3	0.04	< 3	< 7	< 1	< 2	0.50	< 0.3	< 1	3	3	0.02	< 1	0.02	0.01	28	7	< 1	> 10.0	< 1	0.001	< 3	< 5
89-102-V 13.72m (4)	< 0.3	< 0.01	< 3	< 7	< 1	< 2	0.25	< 0.3	< 1	1	1	< 0.01	< 1	< 0.01	0.01	< 1	6	< 1	> 10.0	< 1	< 0.001	< 3	< 5
89-102-V 17.85m (5)	< 0.3	0.02	< 3	11	< 1	< 2	20.8	< 0.3	< 1	< 1	5	0.01	< 1	0.01	0.32	< 1	16	< 1	> 10.0	< 1	< 0.001	< 3	< 5
89-102-V 20.02m (6)	< 0.3	0.01	5	48	< 1	< 2	21.6	< 0.3	< 1	4	5	< 0.01	1	< 0.01	0.63	1	14	3	2.38	< 1	< 0.001	< 3	< 5

Analyte Symbol	S	Sc	Sr	Te	Ti	Tl	U	V	W	Y	Zn	Zr
Unit Symbol	%	ppm	ppm	ppm	%	ppm	ppm	ppm	ppm	ppm	ppm	ppm
Lower Limit	0.01	4	1	2	0.01	5	10	2	5	1	1	5
Method Code	TD-ICP	TD-ICP	TD-ICP	TD-ICP	TD-ICP	TD-ICP	TD-ICP	TD-ICP	TD-ICP	TD-ICP	TD-ICP	TD-ICP
C-644531	1.78	8	173	< 2	0.23	< 5	10	66	< 5	9	18	48
C-644532	1.50	10	187	3	0.40	< 5	< 10	101	5	22	31	40
C-644534	5.59	7	421	6	0.22	< 5	< 10	46	< 5	12	25	63
C-644535	8.58	7	618	5	0.18	< 5	< 10	41	< 5	9	24	38
C-644536	12.0	5	734	< 2	0.12	< 5	< 10	33	< 5	6	17	27
C-644537	9.94	< 4	635	< 2	0.10	< 5	< 10	26	< 5	6	15	22
C-644538	1.67	12	214	4	0.26	< 5	< 10	72	< 5	11	41	45
C-644540	0.85	5	102	< 2	0.15	< 5	< 10	30	< 5	8	19	42
C-644541	0.05	< 4	3	< 2	< 0.01	< 5	< 10	< 2	< 5	< 1	< 1	< 5
C-644542	0.03	< 4	2	< 2	< 0.01	< 5	< 10	< 2	< 5	< 1	1	< 5
C-644543	0.29	< 4	21	< 2	0.02	< 5	< 10	6	< 5	< 1	4	6
C-644544	2.60	< 4	141	< 2	0.12	< 5	< 10	24	< 5	7	15	42
C-644545	0.23	< 4	11	< 2	< 0.01	< 5	< 10	< 2	< 5	< 1	< 1	< 5
C-644546	0.17	< 4	8	< 2	< 0.01	< 5	< 10	< 2	< 5	< 1	< 1	< 5
C-644547	0.15	< 4	9	< 2	< 0.01	< 5	< 10	< 2	< 5	< 1	< 1	< 5
C-644548	0.28	< 4	13	< 2	< 0.01	< 5	< 10	< 2	< 5	< 1	< 1	< 5
C-644549	0.04	< 4	5	< 2	< 0.01	< 5	< 10	< 2	< 5	< 1	10	< 5
C-644551	0.05	< 4	4	< 2	< 0.01	< 5	< 10	< 2	< 5	< 1	< 1	< 5
C-644552	0.05	< 4	6	< 2	< 0.01	< 5	< 10	< 2	< 5	< 1	< 1	< 5
C-644553	0.45	< 4	77	< 2	0.06	< 5	< 10	17	< 5	3	10	11
C-644554	1.87	< 4	95	< 2	0.02	< 5	< 10	5	< 5	< 1	4	7
C-644556	0.04	< 4	5	< 2	< 0.01	< 5	< 10	< 2	< 5	< 1	1	< 5
C-644557	0.13	< 4	11	< 2	< 0.01	< 5	< 10	< 2	< 5	< 1	< 1	< 5
C-644558	0.02	< 4	3	< 2	< 0.01	< 5	< 10	< 2	< 5	< 1	< 1	< 5
C-644559	0.19	< 4	11	< 2	< 0.01	< 5	< 10	< 2	< 5	< 1	1	< 5
C-644560	0.14	< 4	7	< 2	< 0.01	< 5	< 10	< 2	< 5	< 1	< 1	< 5
C-644561	0.04	< 4	7	< 2	< 0.01	< 5	10	< 2	< 5	< 1	2	< 5
C-644562	0.46	4	274	< 2	0.12	< 5	< 10	28	< 5	5	20	29
C-644563	0.38	< 4	19	< 2	< 0.01	< 5	< 10	< 2	< 5	< 1	3	< 5
C-644564	0.63	10	612	6	0.31	< 5	< 10	70	< 5	8	47	62
C-644565	0.01	< 4	3	< 2	< 0.01	< 5	< 10	< 2	< 5	< 1	2	< 5
C-644566	0.14	< 4	14	< 2	< 0.01	< 5	< 10	< 2	< 5	< 1	1	< 5
C-644567	0.13	< 4	15	< 2	< 0.01	< 5	< 10	< 2	< 5	< 1	2	< 5
C-644568	0.61	< 4	84	< 2	0.02	< 5	< 10	6	< 5	1	5	5
C-644569	1.76	6	347	< 2	0.15	< 5	< 10	37	< 5	6	26	34
C-644570	1.61	7	461	< 2	0.19	< 5	< 10	42	< 5	7	34	44
C-644571	7.67	7	669	3	0.19	< 5	< 10	41	< 5	6	28	48
C-644572	0.01	< 4	4	< 2	< 0.01	< 5	< 10	< 2	< 5	< 1	1	< 5
C-644574	0.06	< 4	12	< 2	< 0.01	< 5	< 10	< 2	< 5	< 1	2	< 5
C-644575	0.29	< 4	24	< 2	< 0.01	< 5	< 10	< 2	< 5	< 1	1	< 5
C-644576	1.07	< 4	41	< 2	< 0.01	< 5	< 10	< 2	< 5	< 1	< 1	< 5
C-644577	3.91	< 4	107	< 2	< 0.01	< 5	< 10	< 2	< 5	< 1	< 1	< 5
C-644578	0.21	< 4	10	< 2	< 0.01	< 5	< 10	< 2	< 5	< 1	< 1	< 5
C-644579	1.30	5	468	< 2	0.14	< 5	< 10	33	< 5	7	20	39
C-644580	0.89	5	561	< 2	0.15	< 5	< 10	32	< 5	7	21	36
C-644581	0.50	9	685	5	0.26	< 5	< 10	57	< 5	7	41	54
C-644582	0.79	11	962	3	0.28	< 5	< 10	63	< 5	8	40	59
C-644583	0.25	< 4	16	< 2	< 0.01	< 5	< 10	< 2	< 5	< 1	1	< 5
C-644584	5.19	< 4	401	< 2	0.09	< 5	< 10	22	< 5	5	15	25
C-644585	0.53	< 4	60	< 2	0.02	< 5	< 10	4	< 5	< 1	3	< 5
C-644586	6.57	< 4	526	< 2	0.07	< 5	< 10	18	< 5	3	14	16

Analyte Symbol	S	Sc	Sr	Te	Ti	Tl	U	V	W	Y	Zn	Zr
Unit Symbol	%	ppm	ppm	ppm	%	ppm	ppm	ppm	ppm	ppm	ppm	ppm
Lower Limit	0.01	4	1	2	0.01	5	10	2	5	1	1	5
Method Code	TD-ICP	TD-ICP	TD-ICP	TD-ICP	TD-ICP	TD-ICP	TD-ICP	TD-ICP	TD-ICP	TD-ICP	TD-ICP	TD-ICP
C-644587	0.16	8	615	4	0.12	< 5	< 10	28	< 5	9	28	29
C-644588	1.00	5	499	< 2	0.14	< 5	< 10	29	< 5	6	21	29
C-644589	3.50	5	246	< 2	0.15	< 5	< 10	33	< 5	10	22	38
C-644590	0.21	6	350	< 2	0.13	< 5	< 10	26	< 5	6	21	25
C-644591	0.35	< 4	113	< 2	0.03	< 5	< 10	11	< 5	1	6	9
C-644592	0.31	6	498	5	0.17	< 5	< 10	37	< 5	7	24	34
C-644593	0.23	< 4	420	< 2	0.09	< 5	< 10	22	< 5	6	15	18
C-644594	0.39	7	557	< 2	0.18	< 5	< 10	37	< 5	8	27	33
C-644595	0.54	10	728	< 2	0.29	< 5	< 10	69	< 5	8	42	59
C-644596	0.12	< 4	21	< 2	< 0.01	< 5	< 10	< 2	< 5	< 1	2	< 5
C-644597	0.19	< 4	8	< 2	< 0.01	< 5	< 10	< 2	< 5	< 1	< 1	< 5
C-644598	0.16	< 4	30	< 2	0.01	< 5	10	3	< 5	< 1	3	< 5
C-644599	0.02	< 4	12	< 2	< 0.01	< 5	< 10	< 2	< 5	< 1	< 1	< 5
C-644600	0.14	6	772	4	0.12	< 5	< 10	30	< 5	6	23	27
C-644601	6.79	< 4	699	< 2	0.05	< 5	< 10	15	< 5	2	9	8
C-644602	0.03	< 4	9	< 2	< 0.01	< 5	< 10	< 2	< 5	< 1	2	< 5
C-644603	0.16	< 4	249	< 2	0.06	< 5	10	15	< 5	2	9	16
C-644604	0.03	< 4	6	< 2	< 0.01	< 5	< 10	< 2	< 5	< 1	< 1	< 5
C-644605	0.09	< 4	7	< 2	< 0.01	< 5	< 10	< 2	< 5	< 1	< 1	< 5
C-644606	0.29	< 4	67	< 2	0.04	< 5	< 10	9	< 5	1	7	7
C-644607	0.23	7	522	2	0.12	< 5	< 10	26	< 5	6	31	26
C-644608	0.16	7	608	2	0.19	< 5	< 10	41	< 5	8	28	42
C-644609	0.07	< 4	171	< 2	0.06	< 5	< 10	15	< 5	2	11	12
C-644610	0.18	< 4	14	< 2	< 0.01	< 5	< 10	< 2	< 5	< 1	< 1	< 5
C-644611	0.27	< 4	17	< 2	< 0.01	< 5	< 10	< 2	< 5	< 1	< 1	< 5
C-644612	0.24	< 4	17	< 2	< 0.01	< 5	< 10	< 2	< 5	< 1	1	< 5
C-644613	1.28	5	435	< 2	0.14	< 5	< 10	34	< 5	6	23	34
C-644614	0.06	< 4	21	< 2	< 0.01	< 5	< 10	< 2	< 5	< 1	2	< 5
C-644615	0.06	< 4	8	< 2	< 0.01	< 5	< 10	< 2	< 5	< 1	< 1	< 5
C-644616	0.06	< 4	7	< 2	< 0.01	< 5	< 10	< 2	< 5	< 1	1	< 5
C-644617	0.53	< 4	42	< 2	< 0.01	< 5	< 10	< 2	< 5	< 1	1	< 5
C-644618	0.90	7	334	< 2	0.21	< 5	< 10	49	< 5	7	29	46
C-644619	0.37	6	477	< 2	0.16	< 5	< 10	35	< 5	7	21	37
C-644620	0.08	< 4	62	< 2	< 0.01	< 5	< 10	3	< 5	2	4	< 5
C-644621	0.21	7	451	3	0.20	< 5	10	55	< 5	7	32	39
C-644622	10.6	< 4	771	< 2	< 0.01	< 5	< 10	2	< 5	< 1	1	< 5
C-644623	0.21	8	346	< 2	0.12	< 5	< 10	32	< 5	7	31	29
C-644624	8.07	< 4	390	< 2	0.03	< 5	< 10	10	< 5	1	6	7
C-644625	0.24	< 4	128	2	0.08	< 5	< 10	22	< 5	4	13	22
C-644626	8.66	< 4	692	< 2	0.11	< 5	< 10	27	< 5	2	18	26
C-644627	1.32	4	336	3	0.11	< 5	< 10	28	< 5	4	21	30
C-644628	0.13	< 4	231	< 2	0.09	< 5	< 10	22	< 5	4	15	21
C-644629	0.42	< 4	351	< 2	0.03	< 5	10	11	< 5	5	8	8
C-644630	0.08	8	665	< 2	0.08	< 5	< 10	19	< 5	7	32	26
C-644631	13.2	< 4	642	< 2	0.02	< 5	< 10	4	< 5	< 1	3	< 5
C-644632	3.53	< 4	256	3	0.04	< 5	< 10	7	< 5	4	5	13
C-644633	0.12	< 4	108	2	0.07	< 5	10	15	< 5	4	9	19
C-644634	0.37	< 4	25	< 2	< 0.01	< 5	< 10	< 2	< 5	< 1	< 1	< 5
C-644635	0.51	< 4	39	< 2	< 0.01	< 5	< 10	2	< 5	< 1	2	< 5
C-644637	0.06	< 4	4	< 2	< 0.01	< 5	< 10	< 2	< 5	< 1	< 1	< 5
C-644638	2.69	4	352	< 2	0.11	< 5	< 10	25	< 5	4	18	23

Analyte Symbol	S	Sc	Sr	Te	Ti	Tl	U	V	W	Y	Zn	Zr
Unit Symbol	%	ppm	ppm	ppm	%	ppm	ppm	ppm	ppm	ppm	ppm	ppm
Lower Limit	0.01	4	1	2	0.01	5	10	2	5	1	1	5
Method Code	TD-ICP	TD-ICP	TD-ICP	TD-ICP	TD-ICP	TD-ICP	TD-ICP	TD-ICP	TD-ICP	TD-ICP	TD-ICP	TD-ICP
C-644639	0.27	< 4	32	< 2	0.01	< 5	< 10	3	< 5	< 1	2	< 5
C-644640	0.32	5	361	2	0.13	< 5	10	29	< 5	5	20	28
C-644641	0.40	< 4	24	< 2	< 0.01	< 5	< 10	< 2	< 5	< 1	< 1	< 5
C-644643	0.61	< 4	44	< 2	< 0.01	< 5	< 10	2	< 5	< 1	1	< 5
C-644644	3.25	< 4	357	< 2	0.08	< 5	< 10	20	< 5	4	14	20
C-666387	0.18	< 4	47	< 2	< 0.01	< 5	10	< 2	< 5	< 1	1	< 5
C-666388	0.31	5	459	2	0.16	< 5	< 10	31	< 5	8	21	36
C-666389	4.68	5	662	< 2	0.14	< 5	< 10	29	< 5	7	21	31
C-666390	0.58	8	385	4	0.26	< 5	< 10	58	< 5	7	40	51
C-666391	0.79	< 4	43	< 2	< 0.01	< 5	< 10	< 2	< 5	< 1	< 1	< 5
C-666392	0.09	< 4	9	< 2	< 0.01	< 5	< 10	< 2	< 5	< 1	< 1	< 5
C-666393	0.19	< 4	16	< 2	< 0.01	< 5	< 10	< 2	< 5	< 1	< 1	< 5
C-666394	0.23	< 4	19	< 2	< 0.01	< 5	< 10	< 2	< 5	< 1	< 1	< 5
C-666395	0.20	< 4	152	< 2	0.10	< 5	< 10	23	< 5	5	16	22
C-666396	0.05	< 4	3	< 2	< 0.01	< 5	< 10	< 2	< 5	< 1	< 1	< 5
C-666397	0.08	6	262	< 2	0.12	< 5	< 10	31	< 5	8	22	30
C-666398	0.28	8	330	< 2	0.20	< 5	< 10	41	< 5	8	29	47
C-666399	0.29	8	393	7	0.23	< 5	< 10	46	< 5	8	29	51
C-666400	0.19	< 4	140	< 2	0.04	< 5	< 10	11	< 5	4	6	9
C-666401	1.48	9	345	5	0.25	< 5	< 10	53	< 5	7	33	50
C-644550	0.04	< 4	3	< 2	< 0.01	< 5	< 10	< 2	< 5	< 1	1	< 5
C-644555	0.59	< 4	26	< 2	< 0.01	< 5	< 10	< 2	< 5	< 1	3	< 5
MTU X11-5 Gold 304.06m (98)	9.51	< 4	> 10000	< 2	0.06	< 5	< 10	13	< 5	2	7	20
MTU X11-5 Gold 305.82m (99)	12.7	< 4	1770	< 2	< 0.01	< 5	< 10	< 2	< 5	< 1	3	< 5
MTU X11-5 Gold 306.71m (99)	2.99	< 4	4040	< 2	< 0.01	< 5	< 10	3	< 5	< 1	1	< 5
MTU X11-5 Gold 306.98m (99)	0.01	< 4	15	< 2	< 0.01	< 5	< 10	< 2	< 5	< 1	1	< 5
MTU X11-5 Gold 307.33m (100)	< 0.01	< 4	2	< 2	< 0.01	< 5	< 10	< 2	< 5	< 1	< 1	< 5
MTU X11-5 Gold 307.47m (100)	2.88	< 4	1360	< 2	0.09	< 5	10	20	< 5	4	12	28
MTU X11-5 Gold 309.00m (100)	1.64	< 4	187	2	< 0.01	< 5	< 10	< 2	< 5	< 1	1	< 5
MTU X11-5 Gold 314.12m (102)	0.10	< 4	23	< 2	< 0.01	< 5	< 10	< 2	< 5	< 1	< 1	< 5
MTU X11-5 Gold 318.91m (104)	2.96	< 4	1720	< 2	< 0.01	< 5	< 10	4	< 5	< 1	3	< 5
MTU X11-5 Gold 321.76m (105)	13.1	< 4	1620	< 2	< 0.01	< 5	< 10	< 2	< 5	< 1	1	< 5
MTU X11-5 Gold 330.62m (107)	0.24	< 4	141	< 2	< 0.01	< 5	< 10	< 2	< 5	< 1	< 1	< 5
MTU X11-5 Gold 335.20m (109)	4.02	< 4	420	< 2	< 0.01	< 5	< 10	2	< 5	< 1	2	< 5
MTU X11-5 Gold 339.63m (111)	5.54	< 4	468	< 2	0.02	< 5	10	6	< 5	1	3	7
MTU X11-5 Gold 348.95m (116)	4.41	< 4	442	< 2	0.02	< 5	< 10	5	< 5	1	4	8
MTU X11-5 Gold 352.22m (117)	2.34	< 4	300	< 2	0.12	< 5	< 10	30	< 5	6	17	30
MTU X11-5 Gold 382.40m (133)	7.28	< 4	829	< 2	0.03	< 5	< 10	8	< 5	1	5	9
MTU X11-5 Gold	0.49	< 4	44	2	< 0.01	< 5	< 10	< 2	< 5	< 1	3	< 5

Analyte Symbol	S	Sc	Sr	Te	Ti	Tl	U	V	W	Y	Zn	Zr
Unit Symbol	%	ppm	ppm	ppm	%	ppm	ppm	ppm	ppm	ppm	ppm	ppm
Lower Limit	0.01	4	1	2	0.01	5	10	2	5	1	1	5
Method Code	TD-ICP	TD-ICP	TD-ICP	TD-ICP	TD-ICP	TD-ICP	TD-ICP	TD-ICP	TD-ICP	TD-ICP	TD-ICP	TD-ICP
388.40m (136)												
MTU X11-5 Gold 395.94m (141)	0.05	< 4	2	3	< 0.01	< 5	< 10	< 2	< 5	< 1	10	< 5
MTU X11-5 Gold 433.97m (161)	6.62	< 4	1130	< 2	0.03	< 5	< 10	12	< 5	3	6	13
MTU X11-5 Gold 434.26m (161)	0.36	< 4	51	< 2	< 0.01	< 5	< 10	< 2	< 5	< 1	1	< 5
MTU X11-5 Gold 437.25m (163)	0.94	< 4	126	< 2	< 0.01	< 5	< 10	< 2	< 5	< 1	< 1	< 5
MTU X11-5 Gold 439.96m (164)	0.53	< 4	87	< 2	< 0.01	< 5	< 10	< 2	< 5	< 1	1	< 5
MTU X11-5 Gold 443.80m (166)	0.57	< 4	70	< 2	< 0.01	< 5	< 10	< 2	< 5	< 1	3	< 5
MTU X11-5 Gold 447.32m (168)	0.11	< 4	16	< 2	< 0.01	< 5	< 10	< 2	< 5	< 1	1	< 5
MTU X11-5 Gold 450.85m (170)	0.56	< 4	109	< 2	< 0.01	< 5	< 10	< 2	< 5	< 1	2	< 5
MTU X11-5 Gold 455.79m (173)	0.16	< 4	24	< 2	< 0.01	< 5	< 10	< 2	< 5	< 1	3	< 5
MTU X11-5 Gold 457.45m (174)	0.37	< 4	47	< 2	< 0.01	< 5	< 10	< 2	< 5	< 1	3	< 5
MTU X11-5 Gold 459.28m (175)	0.40	< 4	53	< 2	< 0.01	< 5	< 10	< 2	< 5	< 1	1	< 5
MTU X11-5 Gold 459.89m (175)	< 0.01	< 4	3	< 2	< 0.01	< 5	< 10	< 2	< 5	< 1	< 1	< 5
MTU X11-5 Gold 463.76m (178)	0.69	< 4	84	< 2	< 0.01	< 5	< 10	< 2	< 5	< 1	< 1	< 5
MTU X11-5 Gold 468.36m (180)	1.52	< 4	170	< 2	< 0.01	< 5	< 10	< 2	< 5	< 1	< 1	< 5
MTU X11-5 Gold 475.37m (184)	2.74	< 4	1260	< 2	< 0.01	< 5	< 10	3	< 5	< 1	< 1	< 5
MTU X11-5 Gold 479.52m (186)	0.39	< 4	43	< 2	< 0.01	< 5	< 10	< 2	< 5	< 1	< 1	< 5
MTU X11-5 Gold 492.48m (192)	0.01	< 4	174	2	< 0.01	< 5	< 10	< 2	< 5	< 1	1	< 5
89-102-V 2.74m (1)	2.24	< 4	172	< 2	< 0.01	< 5	< 10	< 2	< 5	< 1	< 1	< 5
89-102-V 5.40m (2)	0.49	< 4	147	< 2	< 0.01	< 5	< 10	< 2	< 5	< 1	1	< 5
89-102-V 11.49m (3)	0.24	< 4	242	< 2	< 0.01	< 5	< 10	< 2	< 5	< 1	2	< 5
89-102-V 13.72m (4)	0.09	< 4	73	< 2	< 0.01	< 5	< 10	< 2	< 5	< 1	1	< 5
89-102-V 17.85m (5)	2.20	< 4	7040	< 2	< 0.01	< 5	< 10	< 2	< 5	< 1	1	< 5
89-102-V 20.02m (6)	3.86	< 4	> 10000	< 2	< 0.01	< 5	10	< 2	< 5	< 1	5	< 5

Analyte Symbol	Ag	Al	As	Ba	Be	Bi	Ca	Cd	Co	Cr	Cu	Fe	Ga	K	Mg	Li	Mn	Mo	Na	Ni	P	Pb	Sb
Unit Symbol	ppm	%	ppm	ppm	ppm	ppm	%	ppm	ppm	ppm	ppm	%	ppm	%	%	ppm	ppm	ppm	%	ppm	%	ppm	ppm
Lower Limit	0.3	0.01	3	7	1	2	0.01	0.3	1	1	1	0.01	1	0.01	0.01	1	1	1	0.01	1	0.001	3	5
Method Code	TD-ICP	TD-ICP	TD-ICP	TD-ICP	TD-ICP	TD-ICP	TD-ICP	TD-ICP	TD-ICP	TD-ICP	TD-ICP	TD-ICP	TD-ICP	TD-ICP	TD-ICP	TD-ICP	TD-ICP	TD-ICP	TD-ICP	TD-ICP	TD-ICP	TD-ICP	TD-ICP
OREAS 101b (4 Acid) Meas									45		419	10.5		1.45	1.23		908	19		11	0.108	16	
OREAS 101b (4 Acid) Cert									45		412	10.7		2.36	1.23		927	20.1		8.2	0.1118	23	
OREAS 101b (4 Acid) Meas									45		429	10.9		2.39	1.23		934	19		9	0.120	15	
OREAS 101b (4 Acid) Cert									45		412	10.7		2.36	1.23		927	20.1		8.2	0.1118	23	
OREAS 101b (4 Acid) Meas									46		413	10.3		1.97	1.26		927	20		11	0.110	15	
OREAS 101b (4 Acid) Cert									45		412	10.7		2.36	1.23		927	20.1		8.2	0.1118	23	
OREAS 101b (4 Acid) Meas									47		427	10.7		2.39	1.28		967	21		9	0.125	16	
OREAS 101b (4 Acid) Cert									45		412	10.7		2.36	1.23		927	20.1		8.2	0.1118	23	
OREAS 101b (4 Acid) Meas									46		418	10.1		2.54	1.21		946	20		9	0.116	14	
OREAS 101b (4 Acid) Cert									45		412	10.7		2.36	1.23		927	20.1		8.2	0.1118	23	
OREAS 98 (4 Acid) Meas	43.7					31			119		> 10000											308	17
OREAS 98 (4 Acid) Cert	45.1					97.2			121		14800 0.0											345	20.1
OREAS 98 (4 Acid) Meas	43.4					43			119		> 10000											315	12
OREAS 98 (4 Acid) Cert	45.1					97.2			121		14800 0.0											345	20.1
OREAS 98 (4 Acid) Meas	43.9					50			119		> 10000											317	12
OREAS 98 (4 Acid) Cert	45.1					97.2			121		14800 0.0											345	20.1
OREAS 98 (4 Acid) Meas	41.5					39			123		> 10000											306	7
OREAS 98 (4 Acid) Cert	45.1					97.2			121		14800 0.0											345	20.1
OREAS 903 (4 Acid) Meas	0.5	6.10	57	202	5	3	0.66	< 0.3	146	72	6530	4.37	16	3.02	0.75	18	696	7	0.03	60	0.110	12	< 5
OREAS 903 (4 Acid) Cert	0.432	5.89	49.7	197	4.42	8.90	0.625	0.200	131	73.0	6520	4.16	15.0	3.31	0.714	18.3	690	4.32	0.0300	54.0	0.107	11.3	1.57
OREAS 903 (4 Acid) Meas	0.7	6.12	51	204	5	5	0.65	< 0.3	145	85	6630	4.36	16	3.48	0.75	18	717	4	0.03	55	0.115	16	< 5
OREAS 903 (4 Acid) Cert	0.432	5.89	49.7	197	4.42	8.90	0.625	0.200	131	73.0	6520	4.16	15.0	3.31	0.714	18.3	690	4.32	0.0300	54.0	0.107	11.3	1.57
OREAS 903 (4 Acid) Meas	< 0.3	5.89	54	194	5	3	0.69	< 0.3	147	65	6590	4.27	17	2.28	0.75	19	712	5	0.03	57	0.105	8	< 5
OREAS 903 (4 Acid) Cert	0.432	5.89	49.7	197	4.42	8.90	0.625	0.200	131	73.0	6520	4.16	15.0	3.31	0.714	18.3	690	4.32	0.0300	54.0	0.107	11.3	1.57
OREAS 903 (4 Acid) Meas	0.6	5.80	53	213	5	4	0.68	< 0.3	146	70	6450	4.20	16	3.52	0.74	18	713	4	0.03	56	0.108	7	< 5
OREAS 903 (4 Acid) Cert	0.432	5.89	49.7	197	4.42	8.90	0.625	0.200	131	73.0	6520	4.16	15.0	3.31	0.714	18.3	690	4.32	0.0300	54.0	0.107	11.3	1.57
OREAS 903 (4 Acid) Meas	0.8	5.76	62	196	5	10	0.66	< 0.3	149	94	6910	4.17	16	3.40	0.74	18	774	5	0.07	58	0.121	7	< 5
OREAS 903 (4 Acid) Cert	0.432	5.89	49.7	197	4.42	8.90	0.625	0.200	131	73.0	6520	4.16	15.0	3.31	0.714	18.3	690	4.32	0.0300	54.0	0.107	11.3	1.57
OREAS 96 (4 Acid) Meas	11.2					17			50		> 10000											86	< 5

Analyte Symbol	Ag	Al	As	Ba	Be	Bi	Ca	Cd	Co	Cr	Cu	Fe	Ga	K	Mg	Li	Mn	Mo	Na	Ni	P	Pb	Sb
Unit Symbol	ppm	%	ppm	ppm	ppm	ppm	%	ppm	ppm	ppm	ppm	%	ppm	%	%	ppm	ppm	ppm	%	ppm	%	ppm	ppm
Lower Limit	0.3	0.01	3	7	1	2	0.01	0.3	1	1	1	0.01	1	0.01	0.01	1	1	1	0.01	1	0.001	3	5
Method Code	TD-ICP	TD-ICP	TD-ICP	TD-ICP	TD-ICP	TD-ICP	TD-ICP	TD-ICP	TD-ICP	TD-ICP	TD-ICP	TD-ICP	TD-ICP	TD-ICP	TD-ICP	TD-ICP	TD-ICP	TD-ICP	TD-ICP	TD-ICP	TD-ICP	TD-ICP	TD-ICP
OREAS 96 (4 Acid) Cert	11.5					26.3			49.9		39300											101	5.09
OREAS 96 (4 Acid) Meas	11.4					23			51		> 10000											89	5
OREAS 96 (4 Acid) Cert	11.5					26.3			49.9		39300											101	5.09
OREAS 96 (4 Acid) Meas	11.8					32			51		> 10000											95	< 5
OREAS 96 (4 Acid) Cert	11.5					26.3			49.9		39300											101	5.09
OREAS 96 (4 Acid) Meas	12.1					36			52		> 10000											95	< 5
OREAS 96 (4 Acid) Cert	11.5					26.3			49.9		39300											101	5.09
OREAS 96 (4 Acid) Meas	10.8					10			51		> 10000											88	< 5
OREAS 96 (4 Acid) Cert	11.5					26.3			49.9		39300											101	5.09
Oreas 77b (4 Acid) Meas	1.7	1.84	1750	24	< 1	7	2.86	0.7	1460	198	3430	28.8	6	0.34	2.68	20	637		0.44	> 10000		74	27
Oreas 77b (4 Acid) Cert	1.62	1.94	2050	118	0.470	3.44	3.06	1.20	1550	280	3426	29.9	4.61	0.361	2.59	18.8	640		0.434	113000		61.0	9.100
Oreas 72b (4 Acid) Meas	0.4	4.69	145	325	< 1	< 2	2.78	0.9	131	640	220	7.01	9	1.11	9.36	32	975	5	0.99	6730	0.026	8	< 5
Oreas 72b (4 Acid) Cert	0.230	4.79	146	330	1.02	0.680	2.79	0.310	131	771	222	6.84	11.7	1.14	9.59	33.3	1010	4.01	1.01	6860	0.0260	14.9	0.870
Oreas 72b (4 Acid) Meas	0.7	4.73	153	322	< 1	< 2	2.78	0.5	132	589	223	7.05	8	1.11	9.38	32	985	4	0.99	6820	0.026	10	< 5
Oreas 72b (4 Acid) Cert	0.230	4.79	146	330	1.02	0.680	2.79	0.310	131	771	222	6.84	11.7	1.14	9.59	33.3	1010	4.01	1.01	6860	0.0260	14.9	0.870
Oreas 72b (4 Acid) Meas	0.5	4.67	147	270	< 1	< 2	2.76	< 0.3	126	562	217	6.81	9	1.07	9.36	33	968	4	0.99	6280	0.027	11	< 5
Oreas 72b (4 Acid) Cert	0.230	4.79	146	330	1.02	0.680	2.79	0.310	131	771	222	6.84	11.7	1.14	9.59	33.3	1010	4.01	1.01	6860	0.0260	14.9	0.870
Oreas 72b (4 Acid) Meas	0.4	4.67	143	240	< 1	< 2	2.76	< 0.3	128	566	222	6.82	10	1.08	9.34	33	992	4	0.99	6400	0.026	12	8
Oreas 72b (4 Acid) Cert	0.230	4.79	146	330	1.02	0.680	2.79	0.310	131	771	222	6.84	11.7	1.14	9.59	33.3	1010	4.01	1.01	6860	0.0260	14.9	0.870
Oreas 72b (4 Acid) Meas	0.4	4.67	143	92	< 1	< 2	2.79	0.4	128	559	222	6.80	10	1.09	9.36	33	985	4	0.99	6430	0.027	12	< 5
Oreas 72b (4 Acid) Cert	0.230	4.79	146	330	1.02	0.680	2.79	0.310	131	771	222	6.84	11.7	1.14	9.59	33.3	1010	4.01	1.01	6860	0.0260	14.9	0.870
Oreas 72b (4 Acid) Meas	0.4	4.67	147	217	< 1	< 2	2.70	< 0.3	130	546	222	6.82	10	1.09	9.34	33	1010	4	1.03	6590	0.026	11	< 5
Oreas 72b (4 Acid) Cert	0.230	4.79	146	330	1.02	0.680	2.79	0.310	131	771	222	6.84	11.7	1.14	9.59	33.3	1010	4.01	1.01	6860	0.0260	14.9	0.870
OREAS 45f (4-Acid) Meas		9.73	9	206	1	< 2	0.09		45	387	360	14.7	28	0.22	0.24	20	232	< 1	0.06	271	0.026	7	< 5
OREAS 45f (4-Acid) Cert		10.2	9.67	206	1.20	0.210	0.0960		44.5	417	363	14.7	26.7	0.224	0.229	20.4	220	2.27	0.0630	256	0.0300	14.7	0.640
OREAS 45f (4-Acid) Meas		9.64	< 3	221	1	2	0.11		45	391	362	14.3	28	0.21	0.24	21	229	< 1	0.06	263	0.023	12	9
OREAS 45f (4-Acid) Cert		10.2	9.67	206	1.20	0.210	0.0960		44.5	417	363	14.7	26.7	0.224	0.229	20.4	220	2.27	0.0630	256	0.0300	14.7	0.640
OREAS 45f (4-Acid) Meas		9.87	4	227	1	3	0.10		47	436	387	14.7	29	0.22	0.24	21	241	< 1	0.07	270	0.028	12	< 5
OREAS 45f (4-Acid) Cert		10.2	9.67	206	1.20	0.210	0.0960		44.5	417	363	14.7	26.7	0.224	0.229	20.4	220	2.27	0.0630	256	0.0300	14.7	0.640

Analyte Symbol	Ag	Al	As	Ba	Be	Bi	Ca	Cd	Co	Cr	Cu	Fe	Ga	K	Mg	Li	Mn	Mo	Na	Ni	P	Pb	Sb
Unit Symbol	ppm	%	ppm	ppm	ppm	ppm	%	ppm	ppm	ppm	ppm	%	ppm	%	%	ppm	ppm	ppm	%	ppm	%	ppm	ppm
Lower Limit	0.3	0.01	3	7	1	2	0.01	0.3	1	1	1	0.01	1	0.01	0.01	1	1	1	0.01	1	0.001	3	5
Method Code	TD-ICP	TD-ICP	TD-ICP	TD-ICP	TD-ICP	TD-ICP	TD-ICP	TD-ICP	TD-ICP	TD-ICP	TD-ICP	TD-ICP	TD-ICP	TD-ICP	TD-ICP	TD-ICP	TD-ICP	TD-ICP	TD-ICP	TD-ICP	TD-ICP	TD-ICP	TD-ICP
OREAS 45f (4-Acid) Meas		9.58	4	202	1	< 2	0.10		47	422	382	14.1	27	0.22	0.23	21	250	1	0.11	268	0.028	8	< 5
OREAS 45f (4-Acid) Cert		10.2	9.67	206	1.20	0.210	0.0960		44.5	417	363	14.7	26.7	0.224	0.229	20.4	220	2.27	0.0630	256	0.0300	14.7	0.640
OREAS 70b (Fusion) Meas		3.67	154	200			2.94		75	751	49	5.41	9	0.58	13.0	34	1100			2030	0.023	11	
OREAS 70b (Fusion) Cert		3.80	143	202			3.08		83	1250	52	5.66	9.71	0.617	13.61	35.3	1160.000			2220.000	0.025	13.2	
OREAS 683 (4 Acid) Meas	< 0.3	7.05		195	< 1	< 2	5.16	< 0.3	83	8540	425	7.62	12	0.51	8.56	7	1240	< 1	1.05	1110	0.051	9	
OREAS 683 (4 Acid) Cert	0.172	7.15		188	0.56	0.16	5.23	0.072	85	9940	404	7.32	13.8	0.507	8.63	6.51	1200	1.00	1.03	1180	0.050	10.2	
OREAS 683 (4 Acid) Meas	0.5	7.06		185	< 1	< 2	5.06	0.6	83	8510	414	7.61	14	0.50	8.38	7	1170	5	1.01	1160	0.048	6	
OREAS 683 (4 Acid) Cert	0.172	7.15		188	0.56	0.16	5.23	0.072	85	9940	404	7.32	13.8	0.507	8.63	6.51	1200	1.00	1.03	1180	0.050	10.2	
OREAS 683 (4 Acid) Meas	< 0.3	6.98		186	< 1	< 2	5.04	0.3	80	7790	403	7.42	12	0.50	8.53	7	1160	2	1.03	1090	0.050	8	
OREAS 683 (4 Acid) Cert	0.172	7.15		188	0.56	0.16	5.23	0.072	85	9940	404	7.32	13.8	0.507	8.63	6.51	1200	1.00	1.03	1180	0.050	10.2	
OREAS 683 (4 Acid) Meas	< 0.3	7.01		188	< 1	< 2	5.12	0.4	80	8310	407	7.50	12	0.50	8.62	7	1170	2	1.04	1100	0.050	7	
OREAS 683 (4 Acid) Cert	0.172	7.15		188	0.56	0.16	5.23	0.072	85	9940	404	7.32	13.8	0.507	8.63	6.51	1200	1.00	1.03	1180	0.050	10.2	
OREAS 683 (4 Acid) Meas	< 0.3	6.57		163	< 1	< 2	4.77	0.3	78	8200	398	7.03	10	0.47	8.00	7	1150	2	1.01	1080	0.049	5	
OREAS 683 (4 Acid) Cert	0.172	7.15		188	0.56	0.16	5.23	0.072	85	9940	404	7.32	13.8	0.507	8.63	6.51	1200	1.00	1.03	1180	0.050	10.2	
OREAS 681 (4 Acid) Meas	< 0.3	7.65		432	1	< 2	5.97		51	1270	272	7.99	18	1.38	5.22	13	1310	1	1.62	481	0.140	4	< 5
OREAS 681 (4 Acid) Cert	0.118	7.91		442	1.41	0.0980	5.98		51.0	1640	264	7.47	17.6	1.35	5.19	13.0	1310	1.38	1.61	503	0.141	10.2	0.240
OREAS 681 (4 Acid) Meas	0.5	7.43		415	1	< 2	5.76		49	1340	258	7.59	13	1.32	5.01	13	1270	2	1.55	469	0.135	4	< 5
OREAS 681 (4 Acid) Cert	0.118	7.91		442	1.41	0.0980	5.98		51.0	1640	264	7.47	17.6	1.35	5.19	13.0	1310	1.38	1.61	503	0.141	10.2	0.240
OREAS 681 (4 Acid) Meas	< 0.3	7.75		439	1	< 2	5.89		49	1300	266	7.66	17	1.32	5.22	14	1270	1	1.60	468	0.143	8	< 5
OREAS 681 (4 Acid) Cert	0.118	7.91		442	1.41	0.0980	5.98		51.0	1640	264	7.47	17.6	1.35	5.19	13.0	1310	1.38	1.61	503	0.141	10.2	0.240
OREAS 681 (4 Acid) Meas	< 0.3	7.78		439	1	2	5.86		49	1330	276	7.62	18	1.32	5.24	14	1270	1	1.62	464	0.144	4	< 5
OREAS 681 (4 Acid) Cert	0.118	7.91		442	1.41	0.0980	5.98		51.0	1640	264	7.47	17.6	1.35	5.19	13.0	1310	1.38	1.61	503	0.141	10.2	0.240
OREAS 681 (4 Acid) Meas	< 0.3	7.53		394	1	< 2	5.57		49	1340	262	7.52	17	1.28	5.06	14	1260	1	1.60	464	0.138	5	< 5
OREAS 681 (4 Acid) Cert	0.118	7.91		442	1.41	0.0980	5.98		51.0	1640	264	7.47	17.6	1.35	5.19	13.0	1310	1.38	1.61	503	0.141	10.2	0.240
OREAS 148 (4 Acid) Meas		5.64	35	> 1000	37	15	0.98		7	98	353	3.18	31	1.58	0.50	4840	396	6	0.91	26	0.095	23	23
OREAS 148 (4 Acid) Cert		5.27	58.0	1000	36.2	18.9	0.872		6.31	60.0	338	3.02	29.2	1.47	0.454	4650	370	8.86	0.860	22.2	0.131	24.9	16.2
OREAS 148 (4 Acid) Meas		5.13	27	971	35	16	0.89		6	60	323	2.91	28	1.44	0.46	4440	360	8	0.84	23	0.074	20	10
OREAS 148 (4 Acid) Cert		5.27	58.0	1000	36.2	18.9	0.872		6.31	60.0	338	3.02	29.2	1.47	0.454	4650	370	8.86	0.860	22.2	0.131	24.9	16.2
OREAS 148 (4 Acid) Meas		5.21	29	> 1000	37	14	0.95		7	58	340	3.00	33	1.47	0.48	4670	399	5	0.87	24	0.079	22	11

Analyte Symbol	Ag	Al	As	Ba	Be	Bi	Ca	Cd	Co	Cr	Cu	Fe	Ga	K	Mg	Li	Mn	Mo	Na	Ni	P	Pb	Sb
Unit Symbol	ppm	%	ppm	ppm	ppm	ppm	%	ppm	ppm	ppm	ppm	%	ppm	%	%	ppm	ppm	ppm	%	ppm	%	ppm	ppm
Lower Limit	0.3	0.01	3	7	1	2	0.01	0.3	1	1	1	0.01	1	0.01	0.01	1	1	1	0.01	1	0.001	3	5
Method Code	TD-ICP	TD-ICP	TD-ICP	TD-ICP	TD-ICP	TD-ICP	TD-ICP	TD-ICP	TD-ICP	TD-ICP	TD-ICP	TD-ICP	TD-ICP	TD-ICP	TD-ICP	TD-ICP	TD-ICP	TD-ICP	TD-ICP	TD-ICP	TD-ICP	TD-ICP	TD-ICP
OREAS 148 (4 Acid) Cert		5.27	58.0	1000	36.2	18.9	0.872		6.31	60.0	338	3.02	29.2	1.47	0.454	4650	370	8.86	0.860	22.2	0.131	24.9	16.2
OREAS 148 (4 Acid) Meas		5.37	37	> 1000	36	15	0.98		6	81	351	3.10	33	1.52	0.50	4810	402	7	0.89	24	0.102	23	77
OREAS 148 (4 Acid) Cert		5.27	58.0	1000	36.2	18.9	0.872		6.31	60.0	338	3.02	29.2	1.47	0.454	4650	370	8.86	0.860	22.2	0.131	24.9	16.2
OREAS 148 (4 Acid) Meas		5.26	23	> 1000	38	15	0.96		6	48	335	3.00	31	1.47	0.48	4650	384	5	0.87	24	0.074	22	15
OREAS 148 (4 Acid) Cert		5.27	58.0	1000	36.2	18.9	0.872		6.31	60.0	338	3.02	29.2	1.47	0.454	4650	370	8.86	0.860	22.2	0.131	24.9	16.2
OREAS 148 (4 Acid) Meas		5.21	23	985	37	14	0.91		7	46	378	2.97	30	1.46	0.48	4650	387	7	0.92	24	0.073	22	23
OREAS 148 (4 Acid) Cert		5.27	58.0	1000	36.2	18.9	0.872		6.31	60.0	338	3.02	29.2	1.47	0.454	4650	370	8.86	0.860	22.2	0.131	24.9	16.2
Oreas 521 (4 Acid) Meas	1.2	4.67	303		< 1	8	3.82		371	35	5920	20.4	15	3.12	1.16	17	3070	134	0.97	78	0.081	5	< 5
Oreas 521 (4 Acid) Cert	0.89	4.77	336		0.9	6	3.86		386	31	6070	20.7	17	3.16	1.13	16	3210	138	0.98	73	0.081	9	6
Oreas 521 (4 Acid) Meas	1.2	4.69	297		< 1	4	3.78		357	50	5860	20.0	18	3.07	1.17	17	3060	131	0.97	69	0.082	4	6
Oreas 521 (4 Acid) Cert	0.89	4.77	336		0.9	6	3.86		386	31	6070	20.7	17	3.16	1.13	16	3210	138	0.98	73	0.081	9	6
Oreas 521 (4 Acid) Meas	1.1	4.81	321		< 1	5	3.91		373	34	5950	20.6	19	3.17	1.21	18	3140	140	1.01	71	0.085	7	6
Oreas 521 (4 Acid) Cert	0.89	4.77	336		0.9	6	3.86		386	31	6070	20.7	17	3.16	1.13	16	3210	138	0.978	73	0.081	9	6
Oreas 521 (4 Acid) Meas	1.0	4.53	303		< 1	3	3.67		369	31	5890	19.5	17	3.01	1.13	18	3040	133	1.00	70	0.080	4	6
Oreas 521 (4 Acid) Cert	0.89	4.77	336		0.9	6	3.86		386	31	6070	20.7	17	3.16	1.13	16	3210	138	0.978	73	0.081	9	6
OREAS 70b (4 Acid) Meas	< 0.3	3.93	151	209	< 1	< 2	3.06	0.6	79		55	5.93	8	0.62	13.7	34	1150	3	0.79	2180	0.023	18	< 5
OREAS 70b (4 Acid) Cert	0.2	3.87	148	202	1	0.8	3.05	0.4	78		52	5.52	10	0.62	13.4	34	1150	3	0.77	2180	0.022	14	0.6
OREAS 70b (4 Acid) Meas	0.3	3.71	152	203	< 1	< 2	2.97	0.4	75		49	5.45	9	0.59	13.0	34	1100	4	0.75	2000	0.023	11	< 5
OREAS 70b (4 Acid) Cert	0.2	3.87	148	202	1	0.8	3.05	0.4	78		52	5.52	10	0.62	13.4	34	1150	3	0.77	2180	0.022	14	0.6
OREAS 70b (4 Acid) Meas	0.4	3.60	129	181	< 1	< 2	2.83	0.3	75		47	5.31	8	0.56	12.6	33	1090	2	0.75	1980	0.022	11	< 5
OREAS 70b (4 Acid) Cert	0.2	3.87	148	202	1	0.8	3.05	0.4	78		52	5.52	10	0.62	13.4	34	1150	3	0.77	2180	0.022	14	0.6
OREAS 620 (4 Acid) Meas	39.6	6.64	52	202	2	4	1.74	161	15	32	1710	3.04	25	2.69	0.34	20	425	19	1.97	16	0.035	> 5000	19
OREAS 620 (4 Acid) Cert	38.5	6.72	50	2490	2	2	1.60	163	12	22	1730	2.94	24	2.63	0.34	20	440	9.5	1.94	15	0.035	7740	76
OREAS 620 (4 Acid) Meas	38.2	6.67	48	70	2	< 2	1.71	162	14	15	1710	2.93	25	1.79	0.35	20	412	9	1.91	15	0.036	> 5000	8
OREAS 620 (4 Acid) Cert	38.5	6.72	50	2500	2	2	1.60	163	12	22	1730	2.94	24	2.63	0.34	20	440	9	1.94	15	0.035	7740	80
OREAS 620 (4 Acid) Meas	40.1	6.87	53	787	2	< 2	1.79	168	14	22	1740	3.05	25	2.38	0.36	21	425	13	1.96	17	0.038	> 5000	28
OREAS 620 (4 Acid) Cert	38.5	6.72	50	2490	2	2	1.60	163	12	22	1730	2.94	24	2.63	0.34	20	440	9.5	1.94	15	0.035	7740	76
OREAS 620 (4 Acid) Meas	37.2	6.31	46	73	2	< 2	1.69	166	14	15	1820	2.94	24	2.05	0.34	20	427	8	2.01	15	0.035	> 5000	8
OREAS 620 (4 Acid) Cert	38.5	6.72	50	2500	2	2	1.60	163	12	22	1730	2.94	24	2.63	0.34	20	440	9	1.94	15	0.035	7740	80

Analyte Symbol	Ag	Al	As	Ba	Be	Bi	Ca	Cd	Co	Cr	Cu	Fe	Ga	K	Mg	Li	Mn	Mo	Na	Ni	P	Pb	Sb
Unit Symbol	ppm	%	ppm	ppm	ppm	ppm	%	ppm	ppm	ppm	ppm	%	ppm	%	%	ppm	ppm	ppm	%	ppm	%	ppm	ppm
Lower Limit	0.3	0.01	3	7	1	2	0.01	0.3	1	1	1	0.01	1	0.01	0.01	1	1	1	0.01	1	0.001	3	5
Method Code	TD-ICP	TD-ICP	TD-ICP	TD-ICP	TD-ICP	TD-ICP	TD-ICP	TD-ICP	TD-ICP	TD-ICP	TD-ICP	TD-ICP	TD-ICP	TD-ICP	TD-ICP	TD-ICP	TD-ICP	TD-ICP	TD-ICP	TD-ICP	TD-ICP	TD-ICP	TD-ICP
OREAS 620 (4 Acid) Meas	37.9	6.38	51	606	2	< 2	1.70	171	14	19	1850	2.99	24	2.62	0.34	21	451	11	2.02	18	0.037	> 5000	15
OREAS 620 (4 Acid) Cert	38.5	6.72	50	2490	2	2	1.60	163	12	22	1730	2.94	24	2.63	0.34	20	440	9.5	1.94	15	0.035	7740	76
OREAS 753 (4 Acid) Meas		8.04	12	17	115	< 2	0.12	1.7	< 1	31	19	0.88	16	2.01	0.01	9020	776	4	2.19	12	0.109	6	< 5
OREAS 753 (4 Acid) Cert		8.22	5.33	18.2	118	2.20	0.113	1.54	0.96	20.8	18.4	0.839	16.1	1.93	0.011	9850.000	740.000	3.32	2.16	10.8	0.111	10.9	0.27
OREAS 753 (4 Acid) Meas		7.45	6	18	110	< 2	0.12	1.6	< 1	14	18	0.82	16	1.85	< 0.01	8930	743	4	2.08	12	0.108	6	< 5
OREAS 753 (4 Acid) Cert		8.22	5.33	18.2	118	2.20	0.113	1.54	0.96	20.8	18.4	0.839	16.1	1.93	0.011	9850.000	740.000	3.32	2.16	10.8	0.111	10.9	0.27
OREAS 753 (4 Acid) Meas		7.86	5	18	113	< 2	0.13	1.8	< 1	15	19	0.84	16	1.89	< 0.01	9080	776	4	2.14	12	0.111	5	< 5
OREAS 753 (4 Acid) Cert		8.22	5.33	18.2	118	2.20	0.113	1.54	0.96	20.8	18.4	0.839	16.1	1.93	0.011	9850.000	740.000	3.32	2.16	10.8	0.111	10.9	0.27
OREAS 753 (4 Acid) Meas		7.16	6	17	114	2	0.12	1.5	< 1	16	20	0.81	15	1.87	< 0.01	8930	803	4	2.19	12	0.110	7	< 5
OREAS 753 (4 Acid) Cert		8.22	5.33	18.2	118	2.20	0.113	1.54	0.96	20.8	18.4	0.839	16.1	1.93	0.011	9850.000	740.000	3.32	2.16	10.8	0.111	10.9	0.27
OREAS 603c (4 Acid) Meas	> 100	6.56	1610		2	96	1.02	32.2	15	12	> 10000	4.22	23	1.72	0.17	28	607	59	1.78	29	0.045	> 5000	65
OREAS 603c (4 Acid) Cert	294	6	1560		2.28	89	1	31.4	14.9	19.4	12100	4	23.3	2	0.162	27.6	660	60	2	26.9	0.043	10428	400
OREAS 603c (4 Acid) Meas	> 100	6.31	1640		2	98	1.05	32.9	16	16	> 10000	4.15	25	2.29	0.17	29	648	59	1.78	28	0.044	> 5000	38
OREAS 603c (4 Acid) Cert	294	6	1560		2.28	89	1	31.4	14.9	19.4	12100	4	23.3	2	0.162	27.6	660	60	2	26.9	0.043	10428	400
OREAS 603c (4 Acid) Meas	> 100	6.13	1580		2	95	1.06	33.3	16	13	> 10000	4.15	24	2.22	0.17	29	630	59	1.79	28	0.045	> 5000	32
OREAS 603c (4 Acid) Cert	294	6	1560		2.28	89	1	31.4	14.9	19.4	12100	4	23.3	2	0.162	27.6	660	60	2	26.9	0.043	10428	400
OREAS 603c (4 Acid) Meas	> 100	6.21	1530		2	85	1.01	32.9	16	13	> 10000	4.08	23	1.74	0.17	29	629	58	1.86	28	0.044	> 5000	38
OREAS 603c (4 Acid) Cert	294	6	1560		2.28	89	1	31.4	14.9	19.4	12100	4	23.3	2	0.162	27.6	660	60	2	26.9	0.043	10428	400
OREAS 601c (4 acid) Meas	52.4	6.80	406		2	22	0.99	3.3	5	15	1180	2.36	23	2.64	0.18	27	225	6	1.91	9	0.038	322	8
OREAS 601c (4 acid) Cert	50.3	7.06	390.000		2.46	21.1	0.953	2.77	4.99	17.5	1160.000	2.41	23.5	2.72	0.169	26.7	230.000	3.66	1.94	6.83	0.039	328	37.2
OREAS 601c (4 acid) Meas	52.6	6.91	417		2	21	1.01	3.0	5	13	1150	2.38	23	2.74	0.17	27	228	5	1.91	8	0.039	319	6
OREAS 601c (4 acid) Cert	50.3	7.06	390.000		2.46	21.1	0.953	2.77	4.99	17.5	1160.000	2.41	23.5	2.72	0.169	26.7	230.000	3.66	1.94	6.83	0.039	328	37.2
OREAS 601c (4 acid) Meas	52.5	6.25	419		2	18	1.00	1.9	5	16	1170	2.28	24	2.68	0.18	28	227	4	1.88	8	0.039	325	8
OREAS 601c (4 acid) Cert	50.3	7.06	390.000		2.46	21.1	0.953	2.77	4.99	17.5	1160.000	2.41	23.5	2.72	0.169	26.7	230.000	3.66	1.94	6.83	0.039	328	37.2
OREAS 601c (4 acid) Meas	53.3	6.62	417		2	19	1.05	2.2	5	14	1170	2.34	25	2.72	0.18	28	236	4	1.92	8	0.039	347	8
OREAS 601c (4 acid) Cert	50.3	7.06	390.000		2.46	21.1	0.953	2.77	4.99	17.5	1160.000	2.41	23.5	2.72	0.169	26.7	230.000	3.66	1.94	6.83	0.039	328	37.2
OREAS 601c (4 acid) Meas	47.9	6.36	405		2	18	0.95	2.0	5	15	1210	2.24	23	2.28	0.17	28	235	4	1.92	7	0.039	344	11
OREAS 601c (4 acid) Cert	50.3	7.06	390.000		2.46	21.1	0.953	2.77	4.99	17.5	1160.000	2.41	23.5	2.72	0.169	26.7	230.000	3.66	1.94	6.83	0.039	328	37.2
OREAS 504 (4 Acid) Meas	3.1										> 10000							674					

Analyte Symbol	Ag	Al	As	Ba	Be	Bi	Ca	Cd	Co	Cr	Cu	Fe	Ga	K	Mg	Li	Mn	Mo	Na	Ni	P	Pb	Sb
Unit Symbol	ppm	%	ppm	ppm	ppm	ppm	%	ppm	ppm	ppm	ppm	%	ppm	%	%	ppm	ppm	ppm	%	ppm	%	ppm	ppm
Lower Limit	0.3	0.01	3	7	1	2	0.01	0.3	1	1	1	0.01	1	0.01	0.01	1	1	1	0.01	1	0.001	3	5
Method Code	TD-ICP	TD-ICP	TD-ICP	TD-ICP	TD-ICP	TD-ICP	TD-ICP	TD-ICP	TD-ICP	TD-ICP	TD-ICP	TD-ICP	TD-ICP	TD-ICP	TD-ICP	TD-ICP	TD-ICP	TD-ICP	TD-ICP	TD-ICP	TD-ICP	TD-ICP	TD-ICP
OREAS 504 (4 Acid) Cert	3.13										11370.000							643					
OREAS 504 (4 Acid) Meas	3.1										> 10000							669					
OREAS 504 (4 Acid) Cert	3.13										11370.000							643					
OREAS 504 (4 Acid) Meas	3.0										> 10000							665					
OREAS 504 (4 Acid) Cert	3.13										11370.000							643					
OREAS 504 (4 Acid) Meas	3.3										> 10000							685					
OREAS 504 (4 Acid) Cert	3.13										11370.000							643					
OREAS 504 (4 Acid) Meas	2.9										> 10000							646					
OREAS 504 (4 Acid) Cert	3.13										11370.000							643					
GMO-04 Meas	2.0		11			91					238							7920				34	< 5
GMO-04 Cert	1.930		4.50			95.00					239.50							7949.0				45.7	8.88
GMO-04 Meas	1.7		15			88					246							7930				37	6
GMO-04 Cert	1.930		4.50			95.00					239.50							7949.0				45.7	8.88
GMO-04 Meas	2.0		5			89					246							8030				37	7
GMO-04 Cert	1.930		4.50			95.00					239.50							7949.0				45.7	8.88
GMO-04 Meas	2.0		6			102					248							7780				37	< 5
GMO-04 Cert	1.930		4.50			95.00					239.50							7949.0				45.7	8.88
C-644546 Orig	< 0.3	< 0.01	< 3	< 7	< 1	< 2	0.25	< 0.3	< 1	17	< 1	< 0.01	< 1	0.14	0.02	< 1	12	< 1	> 10.0	< 1	< 0.001	< 3	< 5
C-644546 Dup	< 0.3	< 0.01	< 3	< 7	< 1	< 2	0.25	< 0.3	< 1	15	< 1	< 0.01	< 1	0.14	0.02	< 1	10	< 1	> 10.0	< 1	< 0.001	< 3	< 5
C-644558 Orig	< 0.3	0.03	< 3	< 7	< 1	< 2	0.13	< 0.3	< 1	17	2	0.02	< 1	0.14	0.07	< 1	5	< 1	> 10.0	< 1	< 0.001	< 3	< 5
C-644558 Dup	< 0.3	0.04	< 3	< 7	< 1	< 2	0.13	< 0.3	< 1	1	< 1	0.02	< 1	0.14	0.07	< 1	5	< 1	> 10.0	< 1	< 0.001	< 3	< 5
C-644568 Orig	< 0.3	0.56	< 3	19	< 1	< 2	1.98	< 0.3	1	7	4	0.26	1	0.45	0.84	14	28	< 1	> 10.0	3	0.004	< 3	< 5
C-644568 Dup	< 0.3	0.57	< 3	19	< 1	< 2	2.03	< 0.3	1	8	3	0.26	2	0.45	0.86	14	28	< 1	> 10.0	3	0.004	< 3	< 5
C-644599 Orig	< 0.3	0.02	< 3	< 7	< 1	< 2	0.12	< 0.3	< 1	2	< 1	0.01	< 1	0.07	0.03	< 1	6	< 1	> 10.0	< 1	< 0.001	< 3	< 5
C-644599 Dup	< 0.3	0.02	< 3	< 7	< 1	< 2	0.12	< 0.3	< 1	2	< 1	0.03	< 1	0.07	0.03	< 1	4	< 1	> 10.0	< 1	< 0.001	< 3	< 5
C-644611 Orig	< 0.3	< 0.01	< 3	< 7	< 1	< 2	0.43	< 0.3	< 1	2	< 1	< 0.01	< 1	0.06	0.01	< 1	7	< 1	> 10.0	< 1	< 0.001	< 3	< 5
C-644611 Dup	< 0.3	< 0.01	< 3	< 7	< 1	< 2	0.44	< 0.3	< 1	2	< 1	< 0.01	< 1	0.06	0.01	< 1	7	< 1	> 10.0	< 1	< 0.001	< 3	< 5
C-644626 Orig	< 0.3	2.47	5	87	< 1	< 2	11.4	< 0.3	8	27	10	1.42	7	1.36	2.06	93	91	1	1.61	18	0.020	9	< 5
C-644626 Dup	< 0.3	2.52	4	87	< 1	< 2	9.84	< 0.3	8	35	11	1.47	6	1.38	2.10	94	94	2	1.65	18	0.020	9	< 5
C-644638 Orig	< 0.3	2.81	< 3	62	< 1	< 2	7.33	< 0.3	5	29	4	1.16	7	1.46	1.37	64	92	< 1	> 10.0	13	0.015	< 3	< 5
C-644638 Dup	< 0.3	2.85	< 3	63	< 1	< 2	7.37	< 0.3	5	19	6	1.17	7	1.50	1.38	64	88	< 1	> 10.0	13	0.015	< 3	< 5
C-666396 Orig	< 0.3	< 0.01	< 3	< 7	< 1	< 2	0.08	< 0.3	< 1	7	3	< 0.01	< 1	0.18	< 0.01	< 1	3	< 1	> 10.0	< 1	< 0.001	9	< 5
C-666396 Dup	< 0.3	< 0.01	< 3	< 7	< 1	< 2	0.08	< 0.3	< 1	4	2	< 0.01	< 1	0.18	< 0.01	< 1	3	< 1	> 10.0	< 1	< 0.001	4	< 5
MTU X11-5 Gold 330.62m (107) Orig	< 0.3	< 0.01	< 3	< 7	< 1	< 2	0.37	< 0.3	< 1	16	< 1	< 0.01	< 1	0.02	0.03	< 1	3	< 1	> 10.0	6	< 0.001	< 3	< 5
MTU X11-5 Gold 330.62m (107) Dup	< 0.3	< 0.01	< 3	< 7	< 1	< 2	0.35	< 0.3	< 1	3	2	< 0.01	< 1	0.02	0.03	< 1	5	< 1	> 10.0	3	< 0.001	< 3	< 5
MTU X11-5 Gold 437.25m (163) Orig	< 0.3	0.06	< 3	< 7	< 1	< 2	1.77	< 0.3	< 1	14	< 1	0.03	< 1	0.05	0.32	7	8	2	> 10.0	3	< 0.001	< 3	< 5
MTU X11-5 Gold 437.25m (163) Dup	< 0.3	0.06	< 3	< 7	< 1	< 2	1.81	< 0.3	< 1	23	< 1	0.03	< 1	0.06	0.33	6	14	2	> 10.0	2	< 0.001	< 3	< 5
MTU X11-5 Gold 468.36m (180)	< 0.3	0.02	< 3	< 7	< 1	< 2	2.27	< 0.3	< 1	< 1	< 1	0.01	< 1	0.03	0.17	3	7	< 1	> 10.0	3	< 0.001	< 3	< 5

Analyte Symbol	Ag	Al	As	Ba	Be	Bi	Ca	Cd	Co	Cr	Cu	Fe	Ga	K	Mg	Li	Mn	Mo	Na	Ni	P	Pb	Sb
Unit Symbol	ppm	%	ppm	ppm	ppm	ppm	%	ppm	ppm	ppm	ppm	%	ppm	%	%	ppm	ppm	ppm	%	ppm	%	ppm	ppm
Lower Limit	0.3	0.01	3	7	1	2	0.01	0.3	1	1	1	0.01	1	0.01	0.01	1	1	1	0.01	1	0.001	3	5
Method Code	TD-ICP	TD-ICP	TD-ICP	TD-ICP	TD-ICP	TD-ICP	TD-ICP	TD-ICP	TD-ICP	TD-ICP	TD-ICP	TD-ICP	TD-ICP	TD-ICP	TD-ICP	TD-ICP	TD-ICP	TD-ICP	TD-ICP	TD-ICP	TD-ICP	TD-ICP	TD-ICP
Orig																							
MTU X11-5 Gold 468.36m (180) Dup	< 0.3	0.02	< 3	< 7	< 1	< 2	2.26	< 0.3	< 1	4	< 1	0.01	< 1	0.03	0.17	3	6	< 1	> 10.0	2	< 0.001	< 3	< 5
Method Blank	< 0.3	< 0.01	< 3	< 7	< 1	< 2	< 0.01	< 0.3	< 1		< 1	< 0.01	< 1	< 0.01	< 0.01	< 1		< 1	< 0.01	< 1	< 0.001	< 3	< 5
Method Blank	< 0.3	< 0.01	< 3	< 7	< 1	< 2	< 0.01	< 0.3	< 1		< 1	< 0.01	< 1	< 0.01	< 0.01	< 1		< 1	< 0.01	< 1	< 0.001	< 3	< 5
Method Blank	< 0.3	< 0.01	< 3	< 7	< 1	< 2	< 0.01	< 0.3	< 1	2	< 1	< 0.01	< 1	< 0.01	< 0.01	< 1		< 1	< 0.01	< 1	< 0.001	< 3	< 5
Method Blank	< 0.3	< 0.01	< 3	< 7	< 1	< 2	< 0.01	< 0.3	< 1	3	< 1	< 0.01	< 1	< 0.01	< 0.01	< 1		< 1	< 0.01	< 1	< 0.001	< 3	< 5
Method Blank	< 0.3	< 0.01	< 3	< 7	< 1	< 2	< 0.01	< 0.3	< 1		< 1	< 0.01	< 1	< 0.01	< 0.01	< 1		< 1	< 0.01	< 1	< 0.001	< 3	< 5
Method Blank	< 0.3	< 0.01	< 3	< 7	< 1	< 2	< 0.01	< 0.3	< 1	3	< 1	< 0.01	< 1	< 0.01	< 0.01	< 1		< 1	< 0.01	< 1	< 0.001	< 3	< 5
Method Blank	< 0.3	< 0.01	< 3	< 7	< 1	< 2	< 0.01	< 0.3	< 1	6	2	< 0.01	< 1	< 0.01	< 0.01	< 1		< 1	< 0.01	< 1	< 0.001	< 3	< 5
Method Blank	< 0.3	< 0.01	< 3	< 7	< 1	< 2	< 0.01	< 0.3	< 1	4	< 1	< 0.01	< 1	< 0.01	< 0.01	< 1		< 1	< 0.01	< 1	< 0.001	< 3	< 5
Method Blank	< 0.3	< 0.01	< 3	< 7	< 1	< 2	< 0.01	< 0.3	< 1	6	< 1	< 0.01	< 1	< 0.01	< 0.01	< 1		< 1	< 0.01	< 1	< 0.001	< 3	< 5
Method Blank	< 0.3	< 0.01	< 3	< 7	< 1	< 2	< 0.01	< 0.3	< 1	6	< 1	< 0.01	< 1	< 0.01	< 0.01	< 1		< 1	< 0.01	< 1	< 0.001	< 3	< 5
Method Blank	< 0.3	< 0.01	< 3	< 7	< 1	< 2	< 0.01	< 0.3	< 1		< 1	< 0.01	< 1	< 0.01	< 0.01	< 1		< 1	< 0.01	< 1	< 0.001	< 3	< 5
Method Blank	< 0.3	< 0.01	< 3	< 7	< 1	< 2	< 0.01	< 0.3	< 1	7	< 1	< 0.01	< 1	< 0.01	< 0.01	< 1		< 1	< 0.01	< 1	< 0.001	< 3	< 5
Method Blank	< 0.3	< 0.01	< 3	< 7	< 1	< 2	< 0.01	< 0.3	< 1		< 1	< 0.01	< 1	< 0.01	< 0.01	< 1		< 1	< 0.01	< 1	< 0.001	< 3	< 5
Method Blank	< 0.3	< 0.01	< 3	< 7	< 1	< 2	< 0.01	< 0.3	< 1	4	< 1	< 0.01	< 1	< 0.01	< 0.01	< 1		< 1	< 0.01	< 1	< 0.001	< 3	< 5
Method Blank	< 0.3	< 0.01	< 3	< 7	< 1	< 2	< 0.01	< 0.3	< 1		< 1	< 0.01	< 1	< 0.01	< 0.01	< 1	3	< 1	< 0.01	< 1	< 0.001	< 3	< 5
Method Blank	< 0.3	< 0.01	< 3	< 7	< 1	< 2	< 0.01	< 0.3	< 1		< 1	< 0.01	< 1	< 0.01	< 0.01	< 1		< 1	< 0.01	< 1	< 0.001	< 3	< 5
Method Blank	< 0.3	< 0.01	< 3	< 7	< 1	< 2	< 0.01	< 0.3	< 1		< 1	< 0.01	< 1	< 0.01	< 0.01	< 1		< 1	< 0.01	< 1	< 0.001	< 3	< 5
Method Blank	< 0.3	< 0.01	< 3	< 7	< 1	< 2	< 0.01	< 0.3	< 1		< 1	< 0.01	< 1	< 0.01	< 0.01	< 1	3	< 1	< 0.01	< 1	< 0.001	< 3	< 5
Method Blank	< 0.3	< 0.01	< 3	< 7	< 1	< 2	< 0.01	< 0.3	< 1		< 1	< 0.01	< 1	< 0.01	< 0.01	< 1		< 1	< 0.01	< 1	< 0.001	< 3	< 5

Analyte Symbol	S	Sc	Sr	Te	Ti	Tl	U	V	W	Y	Zn	Zr
Unit Symbol	%	ppm	ppm	ppm	%	ppm	ppm	ppm	ppm	ppm	ppm	ppm
Lower Limit	0.01	4	1	2	0.01	5	10	2	5	1	1	5
Method Code	TD-ICP	TD-ICP	TD-ICP	TD-ICP	TD-ICP	TD-ICP	TD-ICP	TD-ICP	TD-ICP	TD-ICP	TD-ICP	TD-ICP
OREAS 101b (4 Acid) Meas					0.34		380	79		136		
OREAS 101b (4 Acid) Cert					0.35		387	77		133		
OREAS 101b (4 Acid) Meas					0.36		390	81		137		
OREAS 101b (4 Acid) Cert					0.35		387	77		133		
OREAS 101b (4 Acid) Meas					0.36		380	77		128		
OREAS 101b (4 Acid) Cert					0.35		387	77		133		
OREAS 101b (4 Acid) Meas					0.37		390	80		134		
OREAS 101b (4 Acid) Cert					0.35		387	77		133		
OREAS 101b (4 Acid) Meas					0.36		390	78		128		
OREAS 101b (4 Acid) Cert					0.35		387	77		133		
OREAS 98 (4 Acid) Meas											1270	
OREAS 98 (4 Acid) Cert											1360	
OREAS 98 (4 Acid) Meas											1280	
OREAS 98 (4 Acid) Cert											1360	
OREAS 98 (4 Acid) Meas											1280	
OREAS 98 (4 Acid) Cert											1360	
OREAS 98 (4 Acid) Meas											1360	
OREAS 98 (4 Acid) Cert											1360	
OREAS 903 (4 Acid) Meas	0.50	11	83		0.29	< 5	< 10	84		23	26	98
OREAS 903 (4 Acid) Cert	0.500	10.2	77.0		0.192	0.620	7.58	74.0		22.5	24.3	152
OREAS 903 (4 Acid) Meas	0.50	11	84		0.29	< 5	< 10	83		22	27	155
OREAS 903 (4 Acid) Cert	0.500	10.2	77.0		0.192	0.620	7.58	74.0		22.5	24.3	152
OREAS 903 (4 Acid) Meas	0.51	11	87		0.31	< 5	< 10	82		23	28	13
OREAS 903 (4 Acid) Cert	0.500	10.2	77.0		0.192	0.620	7.58	74.0		22.5	24.3	152
OREAS 903 (4 Acid) Meas	0.51	11	85		0.30	< 5	< 10	81		23	27	83
OREAS 903 (4 Acid) Cert	0.500	10.2	77.0		0.192	0.620	7.58	74.0		22.5	24.3	152
OREAS 903 (4 Acid) Meas	0.48	11	85		0.30	< 5	< 10	87		22	27	161
OREAS 903 (4 Acid) Cert	0.500	10.2	77.0		0.192	0.620	7.58	74.0		22.5	24.3	152
OREAS 96 (4 Acid) Meas	4.28										447	

Analyte Symbol	S	Sc	Sr	Te	Ti	Tl	U	V	W	Y	Zn	Zr
Unit Symbol	%	ppm	ppm	ppm	%	ppm	ppm	ppm	ppm	ppm	ppm	ppm
Lower Limit	0.01	4	1	2	0.01	5	10	2	5	1	1	5
Method Code	TD-ICP	TD-ICP	TD-ICP	TD-ICP	TD-ICP	TD-ICP	TD-ICP	TD-ICP	TD-ICP	TD-ICP	TD-ICP	TD-ICP
OREAS 96 (4 Acid) Cert	4.19										457	
OREAS 96 (4 Acid) Meas	4.32										451	
OREAS 96 (4 Acid) Cert	4.19										457	
OREAS 96 (4 Acid) Meas	4.52										452	
OREAS 96 (4 Acid) Cert	4.19										457	
OREAS 96 (4 Acid) Meas	4.56										462	
OREAS 96 (4 Acid) Cert	4.19										457	
OREAS 96 (4 Acid) Meas	4.30										465	
OREAS 96 (4 Acid) Cert	4.19										457	
Oreas 77b (4 Acid) Meas		< 4	34	5	0.06	< 5	10	38	6	7	187	40
Oreas 77b (4 Acid) Cert		3.51	34.4	1.35	0.0640	1.37	1.71	33.6	3.07	6.55	205	37.9
Oreas 72b (4 Acid) Meas	1.44	13	64	3	0.21	< 5	< 10	75	9	13	90	86
Oreas 72b (4 Acid) Cert	1.49	12.8	63.8	0.0920	0.216	0.350	4.68	73.6	4.00	12.8	99.0	88.0
Oreas 72b (4 Acid) Meas	1.42	13	63	3	0.21	< 5	10	75	8	13	94	83
Oreas 72b (4 Acid) Cert	1.49	12.8	63.8	0.0920	0.216	0.350	4.68	73.6	4.00	12.8	99.0	88.0
Oreas 72b (4 Acid) Meas	1.44	12	64	2	0.21	< 5	< 10	72	< 5	12	91	84
Oreas 72b (4 Acid) Cert	1.49	12.8	63.8	0.0920	0.216	0.350	4.68	73.6	4.00	12.8	99.0	88.0
Oreas 72b (4 Acid) Meas	1.42	12	64	5	0.20	< 5	< 10	72	6	12	94	82
Oreas 72b (4 Acid) Cert	1.49	12.8	63.8	0.0920	0.216	0.350	4.68	73.6	4.00	12.8	99.0	88.0
Oreas 72b (4 Acid) Meas	1.48	12	63	4	0.21	< 5	< 10	72	< 5	12	93	81
Oreas 72b (4 Acid) Cert	1.49	12.8	63.8	0.0920	0.216	0.350	4.68	73.6	4.00	12.8	99.0	88.0
Oreas 72b (4 Acid) Meas	1.44	12	64	< 2	0.21	< 5	< 10	72	8	12	96	84
Oreas 72b (4 Acid) Cert	1.49	12.8	63.8	0.0920	0.216	0.350	4.68	73.6	4.00	12.8	99.0	88.0
OREAS 45f (4-Acid) Meas	0.03	36	25		0.44	< 5	< 10	124	< 5	10	35	97
OREAS 45f (4-Acid) Cert	0.0290	36.3	25.1		1.08	0.200	2.09	253	1.27	10.9	35.3	172
OREAS 45f (4-Acid) Meas	0.02	38	28		0.23	< 5	< 10	85	6	11	36	63
OREAS 45f (4-Acid) Cert	0.0290	36.3	25.1		1.08	0.200	2.09	253	1.27	10.9	35.3	172
OREAS 45f (4-Acid) Meas	0.03	40	27		0.62	< 5	< 10	168	< 5	11	36	130
OREAS 45f (4-Acid) Cert	0.0290	36.3	25.1		1.08	0.200	2.09	253	1.27	10.9	35.3	172

Analyte Symbol	S	Sc	Sr	Te	Ti	Tl	U	V	W	Y	Zn	Zr
Unit Symbol	%	ppm	ppm	ppm	%	ppm	ppm	ppm	ppm	ppm	ppm	ppm
Lower Limit	0.01	4	1	2	0.01	5	10	2	5	1	1	5
Method Code	TD-ICP	TD-ICP	TD-ICP	TD-ICP	TD-ICP	TD-ICP	TD-ICP	TD-ICP	TD-ICP	TD-ICP	TD-ICP	TD-ICP
OREAS 45f (4-Acid) Meas	0.03	37	27		0.61	< 5	< 10	170	7	11	36	120
OREAS 45f (4-Acid) Cert	0.0290	36.3	25.1		1.08	0.200	2.09	253	1.27	10.9	35.3	172
OREAS 70b (Fusion) Meas	0.31		73		0.17		< 10	64		8	103	60
OREAS 70b (Fusion) Cert	0.308		72		0.176		1.87	69		10.7	107	67
OREAS 683 (4 Acid) Meas	0.21	20	266		0.26		< 10	184	< 5	7	88	
OREAS 683 (4 Acid) Cert	0.205	19.7	276		0.263		0.58	187	1.23	8.02	92	
OREAS 683 (4 Acid) Meas	0.19	20	263		0.25		< 10	185	5	7	82	
OREAS 683 (4 Acid) Cert	0.205	19.7	276		0.263		0.58	187	1.23	8.02	92	
OREAS 683 (4 Acid) Meas	0.20	20	263		0.25		< 10	174	5	7	81	
OREAS 683 (4 Acid) Cert	0.205	19.7	276		0.263		0.58	187	1.23	8.02	92	
OREAS 683 (4 Acid) Meas	0.20	20	267		0.26		< 10	176	< 5	7	83	
OREAS 683 (4 Acid) Cert	0.205	19.7	276		0.263		0.58	187	1.23	8.02	92	
OREAS 683 (4 Acid) Meas	0.18	18	255		0.25		< 10	173	< 5	7	84	
OREAS 683 (4 Acid) Cert	0.205	19.7	276		0.263		0.58	187	1.23	8.02	92	
OREAS 681 (4 Acid) Meas	0.10	26	472		0.56		< 10	261	5	16	82	64
OREAS 681 (4 Acid) Cert	0.109	27.7	478		0.588		1.44	253	1.09	17.5	88.0	58.0
OREAS 681 (4 Acid) Meas	0.10	26	452		0.54		< 10	250	< 5	15	80	64
OREAS 681 (4 Acid) Cert	0.109	27.7	478		0.588		1.44	253	1.09	17.5	88.0	58.0
OREAS 681 (4 Acid) Meas	0.11	28	472		0.57		< 10	247	< 5	16	82	61
OREAS 681 (4 Acid) Cert	0.109	27.7	478		0.588		1.44	253	1.09	17.5	88.0	58.0
OREAS 681 (4 Acid) Meas	0.11	28	472		0.57		< 10	248	< 5	16	82	63
OREAS 681 (4 Acid) Cert	0.109	27.7	478		0.588		1.44	253	1.09	17.5	88.0	58.0
OREAS 681 (4 Acid) Meas	0.10	26	461		0.56		< 10	243	< 5	15	84	62
OREAS 681 (4 Acid) Cert	0.109	27.7	478		0.588		1.44	253	1.09	17.5	88.0	58.0
OREAS 148 (4 Acid) Meas		9	233		0.23	13	< 10	51	7	21	176	61
OREAS 148 (4 Acid) Cert		8.23	204		0.345	12.2	8.10	54.0	6.45	18.5	162	79.0
OREAS 148 (4 Acid) Meas		8	209		0.18	11	< 10	44	< 5	19	162	47
OREAS 148 (4 Acid) Cert		8.23	204		0.345	12.2	8.10	54.0	6.45	18.5	162	79.0
OREAS 148 (4 Acid) Meas		8	225		0.22	7	< 10	48	< 5	20	172	24

Analyte Symbol	S	Sc	Sr	Te	Ti	Tl	U	V	W	Y	Zn	Zr
Unit Symbol	%	ppm	ppm	ppm	%	ppm	ppm	ppm	ppm	ppm	ppm	ppm
Lower Limit	0.01	4	1	2	0.01	5	10	2	5	1	1	5
Method Code	TD-ICP	TD-ICP	TD-ICP	TD-ICP	TD-ICP	TD-ICP	TD-ICP	TD-ICP	TD-ICP	TD-ICP	TD-ICP	TD-ICP
OREAS 148 (4 Acid) Cert		8.23	204		0.345	12.2	8.10	54.0	6.45	18.5	162	79.0
OREAS 148 (4 Acid) Meas		9	235		0.26	11	< 10	50	< 5	20	172	76
OREAS 148 (4 Acid) Cert		8.23	204		0.345	12.2	8.10	54.0	6.45	18.5	162	79.0
OREAS 148 (4 Acid) Meas		8	226		0.19	11	< 10	45	< 5	20	171	33
OREAS 148 (4 Acid) Cert		8.23	204		0.345	12.2	8.10	54.0	6.45	18.5	162	79.0
OREAS 148 (4 Acid) Meas		8	224		0.18	11	< 10	45	< 5	20	175	33
OREAS 148 (4 Acid) Cert		8.23	204		0.345	12.2	8.10	54.0	6.45	18.5	162	79.0
Oreas 521 (4 Acid) Meas	1.73	14	88	4	0.37	< 5	40	211	54	18	27	122
Oreas 521 (4 Acid) Cert	1.80	14	160	0.8	0.39	0.3	30	209	92	20	24	123
Oreas 521 (4 Acid) Meas	1.78	14	95	6	0.39	< 5	40	204	54	18	25	119
Oreas 521 (4 Acid) Cert	1.80	14	160	0.8	0.39	0.3	30	209	92	20	24	123
Oreas 521 (4 Acid) Meas	1.84	14	89	7	0.42	< 5	40	212	72	18	26	123
Oreas 521 (4 Acid) Cert	1.80	14	160	0.8	0.39	0.3	30	209	92	20	24	123
Oreas 521 (4 Acid) Meas	1.70	14	78	6	0.41	< 5	30	207	89	18	27	121
Oreas 521 (4 Acid) Cert	1.80	14	160	0.8	0.39	0.3	30	209	92	20	24	123
OREAS 70b (4 Acid) Meas	0.30	13	76		0.18	< 5	< 10	70	6	10	108	66
OREAS 70b (4 Acid) Cert	0.31	12	74		0.18	0.3	2	67	5	10	112	66
OREAS 70b (4 Acid) Meas	0.31	12	72		0.17	< 5	< 10	65	6	8	102	61
OREAS 70b (4 Acid) Cert	0.31	12	74		0.18	0.3	2	67	5	10	112	66
OREAS 70b (4 Acid) Meas	0.29	11	71		0.17	< 5	< 10	63	5	8	103	58
OREAS 70b (4 Acid) Cert	0.31	12	74		0.18	0.3	2	67	5	10	112	66
OREAS 620 (4 Acid) Meas	2.51	5	124		0.16	< 5	< 10	23	< 5	12	> 10000	190
OREAS 620 (4 Acid) Cert	2.47	5	131		0.14	2	4	21	2	12	31500	202
OREAS 620 (4 Acid) Meas	2.48	6	107		0.15	< 5	< 10	22	< 5	13	> 10000	205
OREAS 620 (4 Acid) Cert	2.47	5	131		0.14	2	4	21	2	12	31500	202
OREAS 620 (4 Acid) Meas	2.60	6	128		0.16	< 5	< 10	23	< 5	14	> 10000	212
OREAS 620 (4 Acid) Cert	2.47	5	131		0.14	2	4	21	2	12	31500	202
OREAS 620 (4 Acid) Meas	2.47	5	109		0.16	< 5	< 10	23	< 5	12	> 10000	198
OREAS 620 (4 Acid) Cert	2.47	5	131		0.14	2	4	21	2	12	31500	202

Analyte Symbol	S	Sc	Sr	Te	Ti	Tl	U	V	W	Y	Zn	Zr
Unit Symbol	%	ppm	ppm	ppm	%	ppm	ppm	ppm	ppm	ppm	ppm	ppm
Lower Limit	0.01	4	1	2	0.01	5	10	2	5	1	1	5
Method Code	TD-ICP	TD-ICP	TD-ICP	TD-ICP	TD-ICP	TD-ICP	TD-ICP	TD-ICP	TD-ICP	TD-ICP	TD-ICP	TD-ICP
OREAS 620 (4 Acid) Meas	2.51	5	124		0.16	< 5	< 10	23	< 5	12	> 10000	198
OREAS 620 (4 Acid) Cert	2.47	5	131		0.14	2	4	21	2	12	31500	202
OREAS 753 (4 Acid) Meas	0.01	< 4	29		< 0.01	5	< 10	< 2	8	< 1	94	11
OREAS 753 (4 Acid) Cert	0.014	0.10	25.5		0.004	3.67	5.83	1.16	5.62	0.65	87	11.4
OREAS 753 (4 Acid) Meas	0.01	< 4	29		< 0.01	< 5	< 10	< 2	7	< 1	96	11
OREAS 753 (4 Acid) Cert	0.014	0.10	25.5		0.004	3.67	5.83	1.16	5.62	0.65	87	11.4
OREAS 753 (4 Acid) Meas	0.01	< 4	29		< 0.01	< 5	< 10	2	6	< 1	97	11
OREAS 753 (4 Acid) Cert	0.014	0.10	25.5		0.004	3.67	5.83	1.16	5.62	0.65	87	11.4
OREAS 753 (4 Acid) Meas	0.01	< 4	28		< 0.01	< 5	< 10	< 2	7	< 1	97	11
OREAS 753 (4 Acid) Cert	0.014	0.10	25.5		0.004	3.67	5.83	1.16	5.62	0.65	87	11.4
OREAS 603c (4 Acid) Meas	3.70	4	162	10	0.14	7	< 10	19	11	12	8410	167
OREAS 603c (4 Acid) Cert	4	4.14	270	23.8	0.142	4.18	4.42	18.6	8.99	11.6	8030	177
OREAS 603c (4 Acid) Meas	3.78	4	196	14	0.15	< 5	< 10	20	17	12	8330	181
OREAS 603c (4 Acid) Cert	4	4.14	270	23.8	0.142	4.18	4.42	18.6	8.99	11.6	8030	177
OREAS 603c (4 Acid) Meas	3.83	4	151	14	0.15	< 5	< 10	20	15	12	8540	183
OREAS 603c (4 Acid) Cert	4	4.14	270	23.8	0.142	4.18	4.42	18.6	8.99	11.6	8030	177
OREAS 603c (4 Acid) Meas	3.71	4	125	13	0.15	< 5	< 10	20	13	12	8640	183
OREAS 603c (4 Acid) Cert	4	4.14	270	23.8	0.142	4.18	4.42	18.6	8.99	11.6	8030	177
OREAS 601c (4 acid) Meas	1.58	< 4	220	< 2	0.14	< 5	< 10	16	10	11	440	172
OREAS 601c (4 acid) Cert	1.58	4.01	230	7.50	0.135	1.75	4.40	15.5	4.67	11.5	425	178
OREAS 601c (4 acid) Meas	1.58	< 4	210	3	0.14	< 5	< 10	17	10	11	438	170
OREAS 601c (4 acid) Cert	1.58	4.01	230	7.50	0.135	1.75	4.40	15.5	4.67	11.5	425	178
OREAS 601c (4 acid) Meas	1.62	< 4	216	6	0.14	< 5	< 10	16	8	11	444	183
OREAS 601c (4 acid) Cert	1.58	4.01	230	7.50	0.135	1.75	4.40	15.5	4.67	11.5	425	178
OREAS 601c (4 acid) Meas	1.64	4	218	6	0.15	< 5	< 10	17	8	12	454	187
OREAS 601c (4 acid) Cert	1.58	4.01	230	7.50	0.135	1.75	4.40	15.5	4.67	11.5	425	178
OREAS 601c (4 acid) Meas	1.53	< 4	209	5	0.14	< 5	< 10	17	9	11	456	180
OREAS 601c (4 acid) Cert	1.58	4.01	230	7.50	0.135	1.75	4.40	15.5	4.67	11.5	425	178
OREAS 504 (4 Acid) Meas	1.37											

Analyte Symbol	S	Sc	Sr	Te	Ti	Tl	U	V	W	Y	Zn	Zr
Unit Symbol	%	ppm	ppm	ppm	%	ppm	ppm	ppm	ppm	ppm	ppm	ppm
Lower Limit	0.01	4	1	2	0.01	5	10	2	5	1	1	5
Method Code	TD-ICP	TD-ICP	TD-ICP	TD-ICP	TD-ICP	TD-ICP	TD-ICP	TD-ICP	TD-ICP	TD-ICP	TD-ICP	TD-ICP
OREAS 504 (4 Acid) Cert	1.37											
OREAS 504 (4 Acid) Meas	1.35											
OREAS 504 (4 Acid) Cert	1.37											
OREAS 504 (4 Acid) Meas	1.39											
OREAS 504 (4 Acid) Cert	1.37											
OREAS 504 (4 Acid) Meas	1.44											
OREAS 504 (4 Acid) Cert	1.37											
OREAS 504 (4 Acid) Meas	1.31											
OREAS 504 (4 Acid) Cert	1.37											
GMO-04 Meas	0.63								< 5		125	
GMO-04 Cert	0.71								2.60		128.0	
GMO-04 Meas	0.62								11		128	
GMO-04 Cert	0.71								2.60		128.0	
GMO-04 Meas	0.71								< 5		129	
GMO-04 Cert	0.71								2.60		128.0	
GMO-04 Meas	0.70								< 5		128	
GMO-04 Cert	0.71								2.60		128.0	
C-644546 Orig	0.17	< 4	8	< 2	< 0.01	< 5	< 10	< 2	< 5	< 1	< 1	< 5
C-644546 Dup	0.17	< 4	8	< 2	< 0.01	< 5	< 10	< 2	< 5	< 1	< 1	< 5
C-644558 Orig	0.02	< 4	3	< 2	< 0.01	< 5	< 10	< 2	< 5	< 1	< 1	< 5
C-644558 Dup	0.02	< 4	3	< 2	< 0.01	< 5	< 10	< 2	< 5	< 1	< 1	< 5
C-644568 Orig	0.61	< 4	83	< 2	0.02	< 5	< 10	6	< 5	1	5	5
C-644568 Dup	0.62	< 4	85	< 2	0.02	< 5	< 10	6	< 5	1	5	5
C-644599 Orig	0.02	< 4	12	< 2	< 0.01	< 5	< 10	< 2	< 5	< 1	< 1	< 5
C-644599 Dup	0.03	< 4	12	< 2	< 0.01	< 5	< 10	< 2	< 5	< 1	11	< 5
C-644611 Orig	0.26	< 4	17	< 2	< 0.01	< 5	< 10	< 2	< 5	< 1	< 1	< 5
C-644611 Dup	0.27	< 4	17	< 2	< 0.01	< 5	< 10	< 2	< 5	< 1	< 1	< 5
C-644626 Orig	9.50	< 4	721	< 2	0.11	< 5	10	26	< 5	2	17	28
C-644626 Dup	7.81	< 4	663	< 2	0.12	< 5	< 10	27	< 5	2	18	24
C-644638 Orig	2.66	4	352	< 2	0.11	< 5	< 10	25	< 5	4	18	24
C-644638 Dup	2.73	4	352	< 2	0.11	< 5	10	25	< 5	4	18	23
C-666396 Orig	0.05	< 4	3	< 2	< 0.01	< 5	< 10	< 2	< 5	< 1	< 1	< 5
C-666396 Dup	0.05	< 4	3	< 2	< 0.01	< 5	< 10	< 2	< 5	< 1	< 1	< 5
MTU X11-5 Gold 330.62m (107) Orig	0.25	< 4	163	< 2	< 0.01	< 5	< 10	< 2	< 5	< 1	< 1	< 5
MTU X11-5 Gold 330.62m (107) Dup	0.23	< 4	119	< 2	< 0.01	< 5	< 10	< 2	< 5	< 1	< 1	< 5
MTU X11-5 Gold 437.25m (163) Orig	0.93	< 4	125	< 2	< 0.01	< 5	< 10	< 2	< 5	< 1	< 1	< 5
MTU X11-5 Gold 437.25m (163) Dup	0.95	< 4	128	< 2	< 0.01	< 5	< 10	< 2	< 5	< 1	2	< 5
MTU X11-5 Gold 468.36m (180)	1.52	< 4	171	< 2	< 0.01	< 5	< 10	< 2	< 5	< 1	2	< 5

Analyte Symbol	S	Sc	Sr	Te	Ti	Tl	U	V	W	Y	Zn	Zr
Unit Symbol	%	ppm	ppm	ppm	%	ppm	ppm	ppm	ppm	ppm	ppm	ppm
Lower Limit	0.01	4	1	2	0.01	5	10	2	5	1	1	5
Method Code	TD-ICP	TD-ICP	TD-ICP	TD-ICP	TD-ICP	TD-ICP	TD-ICP	TD-ICP	TD-ICP	TD-ICP	TD-ICP	TD-ICP
Orig												
MTU X11-5 Gold 468.36m (180) Dup	1.51	< 4	169	< 2	< 0.01	< 5	< 10	< 2	< 5	< 1	< 1	< 5
Method Blank	< 0.01	< 4	< 1	< 2	< 0.01	< 5	< 10	< 2	< 5	< 1	< 1	< 5
Method Blank	< 0.01	< 4	< 1	< 2	< 0.01	< 5	< 10	< 2	< 5	< 1	< 1	< 5
Method Blank	< 0.01	< 4	< 1	< 2	< 0.01	< 5	< 10	< 2	< 5	< 1	< 1	< 5
Method Blank	< 0.01	< 4	< 1	< 2	< 0.01	< 5	< 10	< 2	< 5	< 1	< 1	< 5
Method Blank	< 0.01	< 4	< 1	< 2	< 0.01	< 5	< 10	< 2	< 5	< 1	1	< 5
Method Blank	< 0.01	< 4	< 1	< 2	< 0.01	< 5	< 10	< 2	< 5	< 1	< 1	< 5
Method Blank	< 0.01	< 4	< 1	< 2	< 0.01	< 5	< 10	< 2	< 5	< 1	< 1	< 5
Method Blank	< 0.01	< 4	< 1	< 2	< 0.01	< 5	< 10	< 2	< 5	< 1	< 1	< 5
Method Blank	< 0.01	< 4	< 1	< 2	< 0.01	< 5	< 10	< 2	< 5	< 1	< 1	< 5
Method Blank	< 0.01	< 4	< 1	< 2	< 0.01	< 5	< 10	< 2	< 5	< 1	< 1	< 5
Method Blank	< 0.01	< 4	< 1	< 2	< 0.01	< 5	< 10	< 2	< 5	< 1	< 1	< 5
Method Blank	< 0.01	< 4	< 1	< 2	< 0.01	< 5	< 10	< 2	< 5	< 1	< 1	< 5
Method Blank	< 0.01	< 4	< 1	< 2	< 0.01	< 5	< 10	< 2	< 5	< 1	< 1	< 5
Method Blank	< 0.01	< 4	< 1	< 2	< 0.01	< 5	< 10	< 2	< 5	< 1	< 1	< 5
Method Blank	< 0.01	< 4	< 1	< 2	< 0.01	< 5	< 10	< 2	< 5	< 1	< 1	< 5
Method Blank	< 0.01	< 4	< 1	< 2	< 0.01	< 5	< 10	< 2	< 5	< 1	< 1	< 5
Method Blank	< 0.01	< 4	< 1	< 2	< 0.01	< 5	< 10	< 2	< 5	< 1	< 1	< 5
Method Blank	< 0.01	< 4	< 1	< 2	< 0.01	< 5	< 10	< 2	< 5	< 1	< 1	< 5
Method Blank	< 0.01	< 4	< 1	< 2	< 0.01	< 5	< 10	< 2	< 5	< 1	< 1	< 5
