# Supplementary material for: Geochemistry and X-ray diffraction data from rock salts and saltwork wastes of Canada: data compilation
Source: Data Brief. 2026 Jun 6;67:112941. doi: 10.1016/j.dib.2026.112941 (PMC13292661; doi:10.1016/j.dib.2026.112941)
Supplement: Supplementary file 8 [file mmc8.pdf]

Quality Analysis ... *Innovative Technologies*

**Report No.:**	**A24-01700**
**Report Date:**	**07-Mar-24**
**Date Submitted:**	**13-Feb-24**
**Your Reference:**	**GNES-H2GS**

Geological Survey of Canada
3303 33rd St NW
Calgary Alberta T2-2A7
Canada

ATTN: Pavel Kabanov

CERTIFICATE OF ANALYSIS

190 Pulp samples were submitted for analysis.

The following analytical package(s) were requested:		Testing Date:
1F2	QOP Total (Total Digestion ICPOES)	2024-02-22 11:58:35

REPORT **A24-01700**

This report may be reproduced without our consent. If only selected portions of the report are reproduced, permission must be obtained. If no instructions were given at time of sample submittal regarding excess material, it will be discarded within 90 days of this report. Our liability is limited solely to the analytical cost of these analyses. Test results are representative only of material submitted for analysis.

Notes:

Values which exceed the upper limit should be assayed for accurate numbers.

Refer to the Scope of Accreditation for information on accredited elements.

SCC Accredited LAB
LAB Accrédité CCN TM

LabID: 266

CERTIFIED BY:

Mark Vandergeest
Quality Control Coordinator

ACTIVATION LABORATORIES LTD.
41 Bittern Street, Ancaster, Ontario, Canada, L9G 4V5
TELEPHONE +905 648-9611 or +1.888.228.5227 FAX +1.905.648.9613
E-MAIL Ancaster@actlabs.com ACTLABS GROUP WEBSITE www.actlabs.com

Analyte Symbol	Ag	Al	As	Ba	Be	Bi	Ca	Cd	Co	Cr	Cu	Fe	Ga	K	Mg	Li	Mn	Mo	Na	Ni	P	Pb	Sb
Unit Symbol	ppm	%	ppm	ppm	ppm	ppm	%	ppm	ppm	ppm	ppm	%	ppm	%	%	ppm	ppm	ppm	%	ppm	%	ppm	ppm
Lower Limit	0.3	0.01	3	7	1	2	0.01	0.3	1	1	1	0.01	1	0.01	0.01	1	1	1	0.01	1	0.001	3	5
Method Code	TD-ICP	TD-ICP	TD-ICP	TD-ICP	TD-ICP	TD-ICP	TD-ICP	TD-ICP	TD-ICP	TD-ICP	TD-ICP	TD-ICP	TD-ICP	TD-ICP	TD-ICP	TD-ICP	TD-ICP	TD-ICP	TD-ICP	TD-ICP	TD-ICP	TD-ICP	TD-ICP
C-666568	< 0.3	0.25	< 3	16	< 1	< 2	29.9	< 0.3	< 1	4	1	0.13	2	0.31	1.68	2	74	< 1	7.39	2	0.004	< 3	< 5
C-666569	< 0.3	0.33	< 3	20	< 1	< 2	37.3	< 0.3	< 1	4	1	0.13	2	0.44	0.92	1	72	< 1	2.66	3	0.004	< 3	< 5
C-666570	< 0.3	0.24	< 3	17	< 1	< 2	33.5	< 0.3	< 1	4	1	0.12	2	0.25	1.57	2	89	< 1	5.72	3	0.003	< 3	< 5
C-666571	< 0.3	0.15	< 3	19	< 1	< 2	32.6	< 0.3	< 1	3	2	0.13	2	0.17	1.15	1	80	< 1	7.16	3	0.003	< 3	< 5
C-666572	< 0.3	0.37	< 3	40	< 1	< 2	26.1	< 0.3	< 1	7	2	0.25	2	0.41	4.16	3	120	< 1	6.60	5	0.005	< 3	< 5
C-666573	< 0.3	0.25	< 3	27	< 1	< 2	32.2	< 0.3	< 1	6	2	0.14	2	0.26	2.23	2	118	< 1	5.86	3	0.008	< 3	< 5
C-666574	< 0.3	1.85	< 3	87	< 1	< 2	28.7	< 0.3	3	21	8	0.88	6	1.20	3.13	19	155	< 1	1.48	16	0.019	< 3	< 5
C-666575	< 0.3	0.42	< 3	20	< 1	< 2	31.7	< 0.3	< 1	6	27	0.21	2	0.42	4.11	4	145	< 1	2.52	5	0.011	4	< 5
C-666576	< 0.3	0.47	< 3	21	< 1	< 2	30.6	< 0.3	< 1	7	3	0.24	2	0.48	4.50	4	150	< 1	3.06	4	0.013	< 3	< 5
C-666577	< 0.3	0.49	< 3	27	< 1	< 2	31.0	< 0.3	1	7	3	0.23	2	0.52	4.42	5	152	< 1	2.00	4	0.012	< 3	< 5
C-666578	< 0.3	0.67	< 3	27	< 1	< 2	28.0	< 0.3	1	8	5	0.33	3	0.70	5.48	8	166	< 1	2.94	6	0.022	< 3	< 5
C-666579	< 0.3	0.44	< 3	28	< 1	< 2	32.4	< 0.3	1	6	3	0.23	2	0.46	4.20	6	148	< 1	2.45	4	0.012	< 3	< 5
C-666580	< 0.3	0.42	< 3	18	< 1	< 2	31.1	< 0.3	< 1	7	4	0.27	2	0.42	3.96	6	151	< 1	3.31	5	0.013	< 3	< 5
C-666581	< 0.3	0.66	< 3	27	< 1	< 2	19.1	< 0.3	1	9	14	0.49	2	0.61	7.17	13	178	2	7.68	11	0.020	< 3	< 5
C-666582	< 0.3	1.84	4	70	< 1	< 2	13.2	< 0.3	3	21	19	0.96	5	1.34	8.23	48	213	< 1	5.03	16	0.018	8	< 5
C-666583	< 0.3	3.26	4	124	< 1	< 2	12.7	< 0.3	7	42	16	1.75	8	2.10	9.41	147	273	< 1	0.15	27	0.027	6	< 5
C-666584	< 0.3	3.48	8	114	< 1	< 2	11.7	< 0.3	9	32	15	1.94	8	1.73	10.5	261	297	< 1	0.18	30	0.031	< 3	< 5
C-666585	< 0.3	2.94	6	83	< 1	< 2	12.1	< 0.3	7	17	16	1.55	7	1.30	11.9	198	284	< 1	0.28	24	0.025	4	< 5
C-666586	< 0.3	7.44	6	288	2	< 2	2.18	< 0.3	15	76	18	3.27	18	2.29	8.13	444	315	< 1	0.37	74	0.058	< 3	< 5
C-666587	< 0.3	4.41	3	156	1	< 2	8.66	< 0.3	9	23	13	1.93	11	1.34	8.41	237	293	< 1	0.19	43	0.029	< 3	< 5
C-666588	< 0.3	3.10	4	118	< 1	< 2	11.4	< 0.3	6	14	6	1.37	8	1.72	9.51	185	249	< 1	1.34	26	0.027	< 3	< 5
C-666589	< 0.3	1.86	< 3	134	< 1	< 2	5.65	< 0.3	3	22	5	0.80	5	1.08	3.74	52	134	< 1	> 10.0	17	0.016	< 3	< 5
C-666590	< 0.3	0.08	< 3	54	< 1	< 2	0.51	< 0.3	< 1	8	< 1	0.04	< 1	0.08	0.25	1	14	< 1	> 10.0	1	0.001	< 3	< 5
C-666591	< 0.3	0.65	< 3	65	< 1	< 2	2.97	< 0.3	1	10	2	0.44	2	0.41	2.05	9	72	< 1	> 10.0	6	0.006	< 3	< 5
C-666592	< 0.3	0.07	< 3	28	< 1	< 2	0.26	< 0.3	< 1	4	6	0.03	< 1	0.07	0.14	1	9	< 1	> 10.0	< 1	< 0.001	< 3	< 5
C-666593	< 0.3	< 0.01	< 3	90	< 1	< 2	0.35	< 0.3	< 1	3	< 1	< 0.01	< 1	0.06	0.04	< 1	6	< 1	> 10.0	< 1	< 0.001	< 3	< 5
C-666594	< 0.3	0.02	< 3	8	< 1	< 2	0.12	< 0.3	< 1	7	< 1	0.02	< 1	0.04	0.05	< 1	7	< 1	> 10.0	< 1	< 0.001	< 3	< 5
C-666595	< 0.3	0.10	< 3	23	< 1	< 2	0.38	< 0.3	< 1	3	< 1	0.04	< 1	0.09	0.20	1	13	< 1	> 10.0	1	< 0.001	< 3	< 5
C-666596	< 0.3	0.14	< 3	13	< 1	< 2	0.67	< 0.3	< 1	7	2	0.15	< 1	4.20	0.57	3	27	< 1	> 10.0	1	0.001	< 3	< 5
C-666597	< 0.3	0.44	< 3	33	< 1	< 2	3.43	< 0.3	< 1	6	2	0.22	1	4.56	2.88	11	65	< 1	> 10.0	4	0.005	< 3	< 5
C-666598	< 0.3	0.02	< 3	30	< 1	< 2	0.11	< 0.3	< 1	40	< 1	< 0.01	< 1	0.78	0.08	< 1	6	< 1	> 10.0	< 1	< 0.001	< 3	< 5
C-666599	< 0.3	0.17	4	13	< 1	< 2	0.42	< 0.3	< 1	3	1	0.13	< 1	4.17	0.33	3	18	< 1	> 10.0	3	0.002	< 3	< 5
C-666600	< 0.3	3.74	5	169	< 1	< 2	7.85	< 0.3	7	25	5	1.71	10	2.86	7.27	61	276	< 1	0.70	32	0.042	< 3	< 5
C-666601	< 0.3	1.35	< 3	65	< 1	< 2	3.77	< 0.3	2	17	2	0.92	4	2.35	2.98	23	117	< 1	> 10.0	12	0.012	< 3	< 5
C-666602	< 0.3	0.02	< 3	25	< 1	< 2	0.06	< 0.3	< 1	< 1	1	< 0.01	< 1	0.07	0.04	< 1	3	< 1	> 10.0	< 1	< 0.001	3	< 5
C-666603	< 0.3	0.11	< 3	20	< 1	< 2	0.43	< 0.3	< 1	3	1	0.14	< 1	4.39	0.26	2	17	< 1	> 10.0	1	< 0.001	< 3	< 5
C-666604	< 0.3	0.13	5	22	< 1	< 2	0.48	< 0.3	< 1	11	4	0.15	< 1	4.43	0.30	2	18	< 1	> 10.0	4	< 0.001	< 3	< 5
C-666605	< 0.3	0.03	< 3	< 7	< 1	< 2	0.08	< 0.3	< 1	2	< 1	0.02	< 1	0.56	0.05	< 1	5	< 1	> 10.0	< 1	< 0.001	< 3	< 5
C-666606	< 0.3	0.05	< 3	16	< 1	< 2	0.27	< 0.3	< 1	5	< 1	0.04	< 1	4.59	0.12	< 1	14	< 1	> 10.0	< 1	< 0.001	< 3	< 5
C-666607	< 0.3	0.04	< 3	32	< 1	< 2	0.06	< 0.3	< 1	3	< 1	0.03	< 1	5.83	0.05	< 1	7	< 1	> 10.0	< 1	< 0.001	< 3	< 5
C-666608	< 0.3	0.37	< 3	32	< 1	< 2	1.03	< 0.3	< 1	5	1	0.39	1	6.80	0.64	6	34	< 1	9.87	3	0.004	< 3	< 5
C-666609	< 0.3	0.08	< 3	32	< 1	< 2	0.23	< 0.3	< 1	2	< 1	0.11	< 1	5.65	0.14	1	19	< 1	> 10.0	1	< 0.001	< 3	< 5
C-666610	< 0.3	0.66	< 3	153	< 1	< 2	1.38	< 0.3	1	15	3	0.54	2	4.45	1.16	13	61	< 1	> 10.0	6	0.006	< 3	< 5
C-666611	< 0.3	< 0.01	< 3	11	< 1	< 2	0.01	< 0.3	< 1	4	< 1	< 0.01	< 1	4.98	< 0.01	< 1	5	< 1	> 10.0	< 1	< 0.001	< 3	< 5
C-666612	< 0.3	< 0.01	< 3	15	< 1	< 2	< 0.01	< 0.3	< 1	2	< 1	< 0.01	< 1	4.31	< 0.01	< 1	2	< 1	> 10.0	< 1	< 0.001	< 3	< 5
C-666613	< 0.3	0.14	< 3	18	< 1	< 2	0.36	< 0.3	< 1	2	1	0.17	< 1	4.22	0.23	2	18	3	6.32	2	0.001	< 3	< 5
C-666614	< 0.3	0.10	< 3	12	< 1	< 2	0.20	< 0.3	< 1	5	< 1	0.06	< 1	2.11	0.12	2	13	< 1	> 10.0	1	< 0.001	8	< 5
C-666615	< 0.3	< 0.01	< 3	26	< 1	< 2	0.05	< 0.3	< 1	2	< 1	< 0.01	< 1	0.72	0.02	< 1	3	< 1	> 10.0	< 1	< 0.001	< 3	< 5
C-666616	< 0.3	0.21	< 3	27	< 1	< 2	0.46	< 0.3	< 1	2	9	0.12	1	1.34	0.35	4	23	< 1	> 10.0	3	0.002	< 3	< 5
C-666617	< 0.3	0.03	< 3	17	< 1	< 2	0.10	< 0.3	< 1	< 1	< 1	0.02	< 1	4.06	0.05	< 1	5	< 1	> 10.0	< 1	< 0.001	< 3	< 5
C-666618	< 0.3	0.11	< 3	30	< 1	< 2	0.38	< 0.3	< 1	4	< 1	0.12	< 1	5.01	0.18	2	15	< 1	6.51	1	< 0.001	< 3	< 5

Analyte Symbol	Ag	Al	As	Ba	Be	Bi	Ca	Cd	Co	Cr	Cu	Fe	Ga	K	Mg	Li	Mn	Mo	Na	Ni	P	Pb	Sb
Unit Symbol	ppm	%	ppm	ppm	ppm	ppm	%	ppm	ppm	ppm	ppm	%	ppm	%	%	ppm	ppm	ppm	%	ppm	%	ppm	ppm
Lower Limit	0.3	0.01	3	7	1	2	0.01	0.3	1	1	1	0.01	1	0.01	0.01	1	1	1	0.01	1	0.001	3	5
Method Code	TD-ICP	TD-ICP	TD-ICP	TD-ICP	TD-ICP	TD-ICP	TD-ICP	TD-ICP	TD-ICP	TD-ICP	TD-ICP	TD-ICP	TD-ICP	TD-ICP	TD-ICP	TD-ICP	TD-ICP	TD-ICP	TD-ICP	TD-ICP	TD-ICP	TD-ICP	TD-ICP
C-666619	< 0.3	1.08	< 3	68	< 1	< 2	3.98	< 0.3	2	14	2	0.45	4	1.93	2.79	26	91	< 1	> 10.0	10	0.010	< 3	< 5
C-666620	< 0.3	0.01	< 3	21	< 1	< 2	0.04	< 0.3	< 1	3	< 1	< 0.01	< 1	1.05	0.04	< 1	3	< 1	> 10.0	< 1	< 0.001	< 3	< 5
C-666621	< 0.3	0.61	< 3	52	< 1	< 2	2.56	< 0.3	< 1	8	3	0.55	2	4.37	1.74	16	82	< 1	2.54	6	0.006	< 3	< 5
C-666622	< 0.3	0.06	< 3	19	< 1	< 2	0.17	< 0.3	< 1	1	< 1	0.04	< 1	4.98	0.06	< 1	11	< 1	9.21	1	< 0.001	< 3	< 5
C-666623	< 0.3	0.30	< 3	49	< 1	< 2	0.69	< 0.3	< 1	3	< 1	0.17	1	1.79	0.53	5	27	< 1	> 10.0	3	0.002	< 3	< 5
C-666624	< 0.3	0.06	< 3	11	< 1	< 2	0.14	< 0.3	< 1	2	< 1	0.03	< 1	4.24	0.11	1	7	< 1	> 10.0	< 1	< 0.001	< 3	< 5
C-666625	< 0.3	2.43	5	100	< 1	< 2	5.09	< 0.3	3	29	3	1.67	6	1.65	3.78	64	141	< 1	> 10.0	21	0.020	< 3	< 5
C-666627	< 0.3	0.04	< 3	74	< 1	< 2	0.66	< 0.3	< 1	7	< 1	0.02	< 1	0.10	0.10	1	7	< 1	> 10.0	< 1	< 0.001	< 3	< 5
C-666628	< 0.3	0.01	< 3	57	< 1	< 2	0.27	< 0.3	< 1	2	< 1	< 0.01	< 1	0.99	0.04	< 1	8	< 1	> 10.0	< 1	< 0.001	< 3	< 5
C-666629	< 0.3	0.02	< 3	16	< 1	< 2	0.05	< 0.3	< 1	4	< 1	< 0.01	< 1	0.74	0.04	< 1	11	< 1	> 10.0	< 1	< 0.001	< 3	< 5
C-666630	< 0.3	< 0.01	< 3	12	< 1	< 2	0.11	< 0.3	< 1	3	< 1	< 0.01	< 1	1.40	0.02	< 1	5	< 1	> 10.0	< 1	< 0.001	< 3	< 5
C-666631	< 0.3	3.83	5	192	< 1	< 2	7.36	< 0.3	7	29	5	2.51	11	2.53	6.33	78	239	< 1	5.09	33	0.036	< 3	< 5
C-666632	< 0.3	0.21	< 3	28	< 1	< 2	1.48	< 0.3	< 1	3	< 1	0.11	1	0.31	0.33	4	15	< 1	> 10.0	2	0.002	24	< 5
C-666633	< 0.3	0.11	< 3	29	< 1	< 2	0.52	< 0.3	< 1	7	1	0.07	< 1	4.03	0.17	3	11	< 1	> 10.0	1	< 0.001	< 3	< 5
C-666634	< 0.3	< 0.01	< 3	< 7	< 1	< 2	0.18	< 0.3	< 1	2	< 1	< 0.01	< 1	0.61	0.03	< 1	5	< 1	> 10.0	< 1	< 0.001	< 3	< 5
C-666635	< 0.3	0.01	< 3	13	< 1	< 2	0.07	< 0.3	< 1	2	9	< 0.01	< 1	0.79	0.04	< 1	5	< 1	> 10.0	< 1	< 0.001	< 3	< 5
C-666636	< 0.3	0.02	< 3	12	< 1	< 2	0.17	< 0.3	< 1	3	< 1	0.02	< 1	2.60	0.05	< 1	4	< 1	> 10.0	< 1	< 0.001	< 3	< 5
C-666637	< 0.3	0.08	< 3	10	< 1	< 2	0.24	< 0.3	< 1	4	1	0.06	< 1	4.31	0.17	2	8	< 1	> 10.0	< 1	< 0.001	< 3	< 5
C-666638	< 0.3	0.02	< 3	23	< 1	< 2	0.08	< 0.3	< 1	1	< 1	0.01	< 1	1.54	0.06	< 1	3	< 1	> 10.0	< 1	< 0.001	< 3	< 5
C-666639	< 0.3	0.01	< 3	74	< 1	< 2	0.05	< 0.3	< 1	1	< 1	0.02	< 1	2.23	1.35	< 1	3	< 1	> 10.0	4	< 0.001	< 3	< 5
C-666640	< 0.3	0.04	< 3	98	< 1	< 2	0.35	< 0.3	< 1	2	< 1	0.04	< 1	1.23	0.88	1	7	< 1	> 10.0	1	< 0.001	< 3	< 5
C-666641	< 0.3	0.11	< 3	20	< 1	< 2	0.20	< 0.3	< 1	3	< 1	0.06	< 1	1.22	0.72	3	10	< 1	> 10.0	1	< 0.001	< 3	< 5
C-666642	< 0.3	< 0.01	< 3	49	< 1	< 2	0.16	< 0.3	< 1	9	< 1	< 0.01	< 1	2.30	0.73	< 1	5	< 1	> 10.0	< 1	< 0.001	< 3	< 5
C-666643	< 0.3	0.03	< 3	48	< 1	< 2	0.18	< 0.3	< 1	3	< 1	0.02	< 1	3.27	1.00	< 1	5	< 1	> 10.0	< 1	< 0.001	< 3	< 5
C-666644	< 0.3	0.02	< 3	11	< 1	< 2	0.21	< 0.3	< 1	4	< 1	0.01	< 1	3.96	0.44	< 1	6	< 1	> 10.0	< 1	< 0.001	< 3	< 5
C-666645	< 0.3	0.04	< 3	34	< 1	< 2	0.11	< 0.3	< 1	3	< 1	0.02	< 1	6.55	1.19	< 1	5	< 1	> 10.0	< 1	< 0.001	< 3	< 5
C-666646	< 0.3	0.24	< 3	43	< 1	< 2	0.79	< 0.3	< 1	3	< 1	0.10	< 1	2.85	0.62	5	18	< 1	> 10.0	2	0.002	< 3	< 5
C-666647	< 0.3	0.08	< 3	46	< 1	< 2	0.65	< 0.3	< 1	5	< 1	0.07	< 1	4.09	0.75	1	14	< 1	> 10.0	< 1	< 0.001	< 3	< 5
C-666648	< 0.3	0.10	< 3	33	< 1	< 2	0.40	< 0.3	< 1	5	< 1	0.05	< 1	4.09	0.21	2	15	< 1	> 10.0	1	< 0.001	< 3	< 5
C-666649	< 0.3	0.21	< 3	113	< 1	< 2	0.75	< 0.3	< 1	7	< 1	0.12	< 1	3.22	1.94	4	18	< 1	7.33	2	0.002	< 3	< 5
C-666650	< 0.3	0.12	< 3	56	< 1	< 2	0.48	< 0.3	< 1	3	< 1	0.14	< 1	3.85	1.54	2	13	< 1	> 10.0	3	0.001	15	< 5
C-666651	< 0.3	0.08	< 3	82	< 1	< 2	0.27	< 0.3	< 1	3	< 1	0.04	< 1	0.84	0.60	2	7	< 1	> 10.0	1	< 0.001	< 3	< 5
C-666652	< 0.3	0.05	< 3	39	< 1	< 2	0.18	< 0.3	< 1	2	< 1	0.05	< 1	4.76	1.10	1	7	< 1	> 10.0	1	< 0.001	< 3	< 5
C-666653	< 0.3	< 0.01	< 3	82	< 1	< 2	0.03	< 0.3	< 1	2	< 1	< 0.01	< 1	1.04	0.63	< 1	4	< 1	> 10.0	1	< 0.001	< 3	< 5
C-666654	< 0.3	< 0.01	< 3	138	< 1	< 2	0.04	< 0.3	< 1	2	< 1	0.01	< 1	1.77	0.96	< 1	4	< 1	> 10.0	< 1	< 0.001	< 3	< 5
C-666655	< 0.3	< 0.01	< 3	43	< 1	< 2	0.03	< 0.3	< 1	2	< 1	< 0.01	< 1	0.62	0.33	< 1	3	< 1	> 10.0	< 1	< 0.001	< 3	< 5
C-666656	< 0.3	0.01	< 3	18	< 1	< 2	0.09	< 0.3	< 1	8	< 1	0.09	< 1	3.25	3.71	< 1	8	< 1	> 10.0	< 1	< 0.001	5	< 5
C-666657	< 0.3	0.01	< 3	31	< 1	< 2	0.50	< 0.3	< 1	4	< 1	< 0.01	< 1	0.18	0.10	< 1	4	< 1	> 10.0	< 1	< 0.001	< 3	< 5
C-666658	< 0.3	< 0.01	< 3	12	< 1	< 2	0.02	< 0.3	< 1	3	< 1	0.04	< 1	4.50	3.16	< 1	4	< 1	0.30	2	< 0.001	< 3	< 5
C-666659	< 0.3	< 0.01	< 3	< 7	< 1	< 2	0.42	< 0.3	< 1	4	< 1	< 0.01	< 1	0.12	0.03	< 1	8	< 1	> 10.0	< 1	< 0.001	< 3	< 5
C-666660	< 0.3	0.88	< 3	42	< 1	< 2	12.1	< 0.3	2	14	1	0.36	3	0.65	2.07	32	45	< 1	0.45	7	0.012	< 3	< 5
C-666661	< 0.3	0.03	< 3	< 7	< 1	< 2	0.53	< 0.3	< 1	2	< 1	0.02	< 1	0.09	0.07	< 1	4	< 1	> 10.0	< 1	< 0.001	< 3	< 5
C-666662	< 0.3	0.01	< 3	8	< 1	< 2	0.22	< 0.3	< 1	5	< 1	< 0.01	< 1	0.06	< 0.01	< 1	5	< 1	> 10.0	< 1	< 0.001	9	< 5
C-666663	< 0.3	< 0.01	< 3	8	< 1	< 2	0.19	< 0.3	< 1	5	< 1	< 0.01	< 1	0.05	0.01	< 1	6	< 1	> 10.0	< 1	< 0.001	< 3	< 5
C-666664	< 0.3	< 0.01	< 3	24	< 1	< 2	0.49	< 0.3	< 1	9	< 1	< 0.01	< 1	0.05	0.03	< 1	7	< 1	> 10.0	< 1	< 0.001	6	< 5
C-666665	< 0.3	< 0.01	< 3	< 7	< 1	< 2	0.18	< 0.3	< 1	4	< 1	< 0.01	< 1	0.04	< 0.01	< 1	9	< 1	> 10.0	< 1	< 0.001	< 3	< 5
C-666667	< 0.3	0.24	< 3	22	< 1	< 2	3.15	< 0.3	< 1	5	< 1	0.12	1	0.19	0.43	8	15	< 1	> 10.0	2	0.003	< 3	< 5
C-666668	< 0.3	0.02	< 3	24	< 1	< 2	0.60	< 0.3	< 1	< 1	2	< 0.01	< 1	0.05	0.03	< 1	6	< 1	> 10.0	< 1	< 0.001	< 3	< 5
C-666669	< 0.3	0.14	< 3	32	< 1	< 2	4.82	< 0.3	< 1	3	2	0.07	1	0.13	0.28	6	12	< 1	> 10.0	1	0.001	< 3	< 5
C-666670	< 0.3	0.02	< 3	< 7	< 1	< 2	1.88	< 0.3	< 1	2	< 1	0.01	< 1	0.05	0.04	1	10	< 1	> 10.0	< 1	< 0.001	< 3	< 5
C-666671	< 0.3	0.95	< 3	54	< 1	< 2	12.2	< 0.3	2	17	3	0.46	3	0.57	1.85	47	48	< 1	0.13	9	0.011	< 3	< 5

Analyte Symbol	Ag	Al	As	Ba	Be	Bi	Ca	Cd	Co	Cr	Cu	Fe	Ga	K	Mg	Li	Mn	Mo	Na	Ni	P	Pb	Sb
Unit Symbol	ppm	%	ppm	ppm	ppm	ppm	%	ppm	ppm	ppm	ppm	%	ppm	%	%	ppm	ppm	ppm	%	ppm	%	ppm	ppm
Lower Limit	0.3	0.01	3	7	1	2	0.01	0.3	1	1	1	0.01	1	0.01	0.01	1	1	1	0.01	1	0.001	3	5
Method Code	TD-ICP	TD-ICP	TD-ICP	TD-ICP	TD-ICP	TD-ICP	TD-ICP	TD-ICP	TD-ICP	TD-ICP	TD-ICP	TD-ICP	TD-ICP	TD-ICP	TD-ICP	TD-ICP	TD-ICP	TD-ICP	TD-ICP	TD-ICP	TD-ICP	TD-ICP	TD-ICP
C-666672	< 0.3	0.08	< 3	11	< 1	< 2	2.84	< 0.3	< 1	7	< 1	0.04	< 1	0.07	0.38	4	10	< 1	> 10.0	1	< 0.001	< 3	< 5
C-666673	< 0.3	0.17	4	127	< 1	< 2	2.85	< 0.3	< 1	10	1	0.09	1	0.14	0.29	9	13	< 1	> 10.0	2	0.001	< 3	< 5
C-666674	< 0.3	0.10	< 3	9	< 1	< 2	2.10	< 0.3	< 1	4	1	0.06	1	0.11	0.33	7	11	< 1	> 10.0	< 1	< 0.001	< 3	< 5
C-666675	< 0.3	< 0.01	< 3	118	< 1	< 2	0.56	< 0.3	< 1	< 1	< 1	< 0.01	< 1	0.03	0.03	< 1	2	< 1	> 10.0	< 1	< 0.001	< 3	< 5
C-666676	< 0.3	< 0.01	< 3	24	< 1	< 2	0.19	< 0.3	< 1	3	< 1	< 0.01	< 1	0.06	0.02	< 1	4	< 1	> 10.0	< 1	< 0.001	< 3	< 5
C-666677	< 0.3	< 0.01	< 3	64	< 1	< 2	0.05	< 0.3	< 1	2	< 1	< 0.01	< 1	0.08	0.01	< 1	6	< 1	> 10.0	< 1	< 0.001	< 3	< 5
C-666679	< 0.3	< 0.01	< 3	< 7	< 1	< 2	0.66	< 0.3	< 1	5	< 1	< 0.01	< 1	0.06	0.02	< 1	3	< 1	> 10.0	< 1	< 0.001	< 3	< 5
C-666680	< 0.3	< 0.01	< 3	43	< 1	< 2	0.26	< 0.3	< 1	2	< 1	< 0.01	< 1	0.07	< 0.01	< 1	4	< 1	> 10.0	< 1	< 0.001	< 3	< 5
C-666681	< 0.3	< 0.01	< 3	< 7	< 1	< 2	0.34	< 0.3	< 1	7	2	< 0.01	< 1	0.05	0.01	< 1	15	< 1	> 10.0	< 1	< 0.001	< 3	< 5
C-666683	< 0.3	< 0.01	< 3	< 7	< 1	< 2	0.17	< 0.3	< 1	6	2	< 0.01	< 1	0.04	< 0.01	< 1	3	< 1	> 10.0	< 1	< 0.001	< 3	< 5
C-666684	< 0.3	< 0.01	< 3	31	< 1	< 2	0.23	< 0.3	< 1	6	5	< 0.01	< 1	0.06	< 0.01	< 1	6	< 1	> 10.0	< 1	< 0.001	< 3	< 5
C-666685	< 0.3	< 0.01	< 3	< 7	< 1	< 2	0.14	< 0.3	< 1	3	< 1	< 0.01	< 1	0.04	0.01	< 1	6	< 1	> 10.0	< 1	< 0.001	< 3	< 5
C-666686	< 0.3	< 0.01	< 3	< 7	< 1	< 2	0.05	< 0.3	< 1	2	< 1	< 0.01	< 1	0.03	0.03	< 1	5	< 1	> 10.0	< 1	< 0.001	4	< 5
C-666687	< 0.3	< 0.01	< 3	28	< 1	< 2	0.53	< 0.3	< 1	6	< 1	< 0.01	< 1	0.03	< 0.01	< 1	7	< 1	> 10.0	< 1	< 0.001	< 3	< 5
C-666688	< 0.3	0.60	< 3	34	< 1	< 2	1.90	< 0.3	1	11	3	0.29	2	0.39	1.56	57	30	< 1	> 10.0	6	0.005	7	< 5
C-666689	< 0.3	0.09	< 3	41	< 1	< 2	0.76	< 0.3	< 1	5	< 1	0.05	< 1	0.08	0.24	9	7	< 1	> 10.0	1	< 0.001	< 3	< 5
C-666690	< 0.3	< 0.01	< 3	74	< 1	< 2	0.06	< 0.3	< 1	6	1	< 0.01	< 1	0.02	0.02	< 1	3	< 1	> 10.0	< 1	< 0.001	< 3	< 5
C-666691	< 0.3	0.01	< 3	7	< 1	< 2	0.48	< 0.3	< 1	2	< 1	< 0.01	< 1	0.04	0.05	1	4	< 1	> 10.0	< 1	< 0.001	< 3	< 5
C-666692	< 0.3	0.04	< 3	49	< 1	< 2	0.97	< 0.3	< 1	2	< 1	0.03	< 1	0.04	0.15	2	5	< 1	> 10.0	< 1	< 0.001	< 3	< 5
C-666693	< 0.3	< 0.01	< 3	8	< 1	< 2	0.37	< 0.3	< 1	9	< 1	< 0.01	< 1	0.03	0.03	< 1	6	< 1	> 10.0	< 1	< 0.001	< 3	< 5
C-666696	< 0.3	< 0.01	< 3	< 7	< 1	< 2	0.08	< 0.3	< 1	2	< 1	< 0.01	< 1	< 0.01	< 0.01	< 1	5	< 1	> 10.0	< 1	< 0.001	< 3	< 5
C-666697	< 0.3	0.02	< 3	104	< 1	< 2	2.00	< 0.3	< 1	5	< 1	0.01	< 1	0.02	0.05	2	6	< 1	> 10.0	< 1	< 0.001	< 3	< 5
C-666698	< 0.3	0.01	< 3	< 7	< 1	< 2	8.71	< 0.3	< 1	13	< 1	0.02	< 1	0.03	0.14	5	9	< 1	> 10.0	< 1	< 0.001	< 3	< 5
C-666699	< 0.3	0.04	< 3	21	< 1	< 2	9.15	< 0.3	< 1	10	2	0.02	1	0.07	0.27	44	5	< 1	> 10.0	< 1	< 0.001	< 3	< 5
C-666700	< 0.3	0.01	< 3	32	< 1	< 2	9.91	< 0.3	< 1	5	< 1	< 0.01	< 1	0.03	0.26	8	5	1	> 10.0	< 1	< 0.001	< 3	< 5
C-666701	< 0.3	0.01	< 3	11	< 1	< 2	10.5	< 0.3	< 1	3	< 1	< 0.01	1	0.03	0.25	8	5	< 1	> 10.0	< 1	< 0.001	< 3	< 5
C-666702	< 0.3	0.03	< 3	< 7	< 1	< 2	12.3	< 0.3	< 1	4	< 1	0.02	1	0.05	1.19	40	7	< 1	0.03	< 1	< 0.001	< 3	< 5
C-666703	< 0.3	0.13	< 3	20	< 1	< 2	20.6	< 0.3	< 1	4	3	0.07	2	0.38	10.7	422	34	< 1	1.68	2	< 0.001	< 3	< 5
C-666704	< 0.3	< 0.01	< 3	< 7	< 1	< 2	12.5	< 0.3	< 1	4	< 1	< 0.01	2	< 0.01	1.09	4	8	< 1	0.02	< 1	< 0.001	< 3	< 5
C-666705	< 0.3	0.09	< 3	15	< 1	< 2	21.4	< 0.3	< 1	3	3	0.11	2	0.14	10.8	72	46	< 1	2.67	3	< 0.001	< 3	< 5
C-666706	< 0.3	0.03	< 3	< 7	< 1	< 2	20.4	< 0.3	< 1	3	< 1	0.07	2	0.08	10.7	35	45	< 1	5.02	1	< 0.001	< 3	< 5
C-666707	< 0.3	< 0.01	< 3	< 7	< 1	< 2	12.4	< 0.3	< 1	4	< 1	< 0.01	1	< 0.01	0.11	3	4	< 1	0.02	< 1	< 0.001	< 3	< 5
C-666709	< 0.3	0.01	< 3	< 7	< 1	< 2	12.4	< 0.3	< 1	3	< 1	0.02	2	0.04	4.54	23	18	< 1	4.86	1	< 0.001	< 3	< 5
C-666710	< 0.3	< 0.01	< 3	< 7	< 1	< 2	11.9	< 0.3	< 1	3	< 1	< 0.01	1	0.03	0.76	29	5	< 1	0.10	< 1	< 0.001	< 3	< 5
C-666711	< 0.3	0.01	< 3	< 7	< 1	< 2	11.4	< 0.3	< 1	4	< 1	< 0.01	2	0.04	1.94	29	9	< 1	0.87	< 1	< 0.001	< 3	< 5
C-666712	< 0.3	< 0.01	< 3	< 7	< 1	< 2	12.5	< 0.3	< 1	11	< 1	< 0.01	1	0.02	0.28	20	6	< 1	0.03	< 1	< 0.001	< 3	< 5
C-666713	< 0.3	< 0.01	< 3	< 7	< 1	< 2	11.6	< 0.3	< 1	13	< 1	< 0.01	2	0.02	0.68	15	5	< 1	0.72	< 1	< 0.001	7	< 5
C-666715	< 0.3	0.03	< 3	< 7	< 1	< 2	11.0	< 0.3	< 1	4	2	0.04	2	0.05	3.94	36	18	1	5.28	2	< 0.001	< 3	< 5
C-666716	< 0.3	< 0.01	< 3	< 7	< 1	< 2	10.8	< 0.3	< 1	2	< 1	< 0.01	1	0.02	0.92	20	7	< 1	0.03	< 1	< 0.001	< 3	< 5
C-666717	< 0.3	< 0.01	8	< 7	< 1	< 2	9.55	< 0.3	< 1	2	< 1	< 0.01	1	0.03	2.11	19	11	< 1	1.44	1	< 0.001	< 3	< 5
C-666718	< 0.3	< 0.01	< 3	18	< 1	< 2	10.7	< 0.3	< 1	4	2	< 0.01	1	0.01	0.40	12	5	< 1	0.03	< 1	< 0.001	< 3	< 5
C-666719	< 0.3	< 0.01	< 3	< 7	< 1	< 2	10.3	< 0.3	< 1	1	< 1	< 0.01	1	0.02	0.17	18	3	< 1	0.02	< 1	< 0.001	< 3	< 5
C-666720	< 0.3	< 0.01	< 3	< 7	< 1	< 2	8.59	< 0.3	< 1	6	< 1	< 0.01	1	0.02	0.74	18	6	< 1	0.11	< 1	< 0.001	< 3	< 5
C-666721	< 0.3	< 0.01	< 3	< 7	< 1	< 2	9.23	< 0.3	< 1	2	< 1	< 0.01	1	0.02	1.19	15	7	< 1	0.03	< 1	< 0.001	< 3	< 5
C-666722	< 0.3	< 0.01	< 3	< 7	< 1	< 2	9.82	< 0.3	< 1	5	< 1	< 0.01	1	< 0.01	0.44	6	7	< 1	0.02	< 1	< 0.001	< 3	< 5
C-666723	< 0.3	< 0.01	< 3	< 7	< 1	< 2	9.46	< 0.3	< 1	7	< 1	< 0.01	1	< 0.01	0.36	7	7	< 1	0.02	2	< 0.001	< 3	< 5
C-666725	< 0.3	< 0.01	< 3	21	< 1	< 2	3.74	< 0.3	< 1	4	< 1	< 0.01	1	0.02	0.07	1	3	< 1	> 10.0	< 1	< 0.001	< 3	< 5
C-666726	< 0.3	< 0.01	< 3	26	< 1	< 2	6.62	< 0.3	< 1	10	5	< 0.01	1	0.02	0.16	2	5	< 1	> 10.0	< 1	< 0.001	4	< 5
C-666727	< 0.3	< 0.01	< 3	8	< 1	< 2	3.08	< 0.3	< 1	3	< 1	< 0.01	< 1	0.02	0.06	1	5	< 1	> 10.0	< 1	< 0.001	< 3	< 5
C-666728	< 0.3	< 0.01	< 3	39	< 1	< 2	0.02	< 0.3	< 1	2	1	< 0.01	< 1	< 0.01	< 0.01	< 1	11	< 1	> 10.0	< 1	< 0.001	< 3	< 5
C-666729	< 0.3	< 0.01	< 3	8	< 1	< 2	0.03	< 0.3	< 1	2	< 1	< 0.01	< 1	< 0.01	< 0.01	< 1	5	< 1	> 10.0	< 1	< 0.001	< 3	< 5

Analyte Symbol	Ag	Al	As	Ba	Be	Bi	Ca	Cd	Co	Cr	Cu	Fe	Ga	K	Mg	Li	Mn	Mo	Na	Ni	P	Pb	Sb
Unit Symbol	ppm	%	ppm	ppm	ppm	ppm	%	ppm	ppm	ppm	ppm	%	ppm	%	%	ppm	ppm	ppm	%	ppm	%	ppm	ppm
Lower Limit	0.3	0.01	3	7	1	2	0.01	0.3	1	1	1	0.01	1	0.01	0.01	1	1	1	0.01	1	0.001	3	5
Method Code	TD-ICP	TD-ICP	TD-ICP	TD-ICP	TD-ICP	TD-ICP	TD-ICP	TD-ICP	TD-ICP	TD-ICP	TD-ICP	TD-ICP	TD-ICP	TD-ICP	TD-ICP	TD-ICP	TD-ICP	TD-ICP	TD-ICP	TD-ICP	TD-ICP	TD-ICP	TD-ICP
C-666730	< 0.3	< 0.01	< 3	94	< 1	< 2	0.82	< 0.3	< 1	3	< 1	< 0.01	< 1	0.02	0.02	< 1	9	< 1	> 10.0	< 1	< 0.001	< 3	< 5
C-666731	< 0.3	< 0.01	< 3	8	< 1	< 2	0.43	< 0.3	< 1	8	< 1	< 0.01	< 1	0.01	0.01	< 1	2	< 1	9.11	< 1	< 0.001	25	< 5
C-666732	< 0.3	< 0.01	< 3	13	< 1	< 2	0.16	< 0.3	< 1	2	< 1	< 0.01	< 1	< 0.01	< 0.01	< 1	9	< 1	> 10.0	< 1	< 0.001	< 3	< 5
C-666734	< 0.3	< 0.01	< 3	13	< 1	< 2	0.06	< 0.3	< 1	3	< 1	< 0.01	< 1	< 0.01	< 0.01	< 1	3	< 1	> 10.0	< 1	< 0.001	< 3	< 5
C-666735	< 0.3	< 0.01	< 3	12	< 1	< 2	1.10	< 0.3	< 1	3	< 1	< 0.01	< 1	0.01	0.05	< 1	7	< 1	> 10.0	< 1	< 0.001	< 3	< 5
C-666736	< 0.3	< 0.01	< 3	< 7	< 1	< 2	2.90	< 0.3	< 1	3	< 1	< 0.01	1	0.01	0.04	2	7	< 1	> 10.0	< 1	< 0.001	< 3	< 5
C-666737	< 0.3	< 0.01	< 3	< 7	< 1	< 2	1.23	< 0.3	< 1	1	< 1	< 0.01	< 1	< 0.01	0.02	< 1	5	< 1	> 10.0	< 1	< 0.001	< 3	< 5
C-666738	< 0.3	< 0.01	< 3	< 7	< 1	< 2	1.99	< 0.3	< 1	2	< 1	< 0.01	< 1	< 0.01	0.05	1	7	< 1	> 10.0	< 1	< 0.001	7	< 5
C-666739	< 0.3	< 0.01	< 3	< 7	< 1	< 2	0.66	< 0.3	< 1	3	< 1	< 0.01	< 1	< 0.01	< 0.01	< 1	11	< 1	> 10.0	< 1	< 0.001	3	< 5
C-666740	< 0.3	< 0.01	< 3	19	< 1	< 2	0.07	< 0.3	< 1	< 1	< 1	< 0.01	< 1	< 0.01	< 0.01	< 1	3	< 1	> 10.0	< 1	< 0.001	< 3	< 5
C-666741	< 0.3	< 0.01	< 3	12	< 1	< 2	1.75	< 0.3	< 1	3	< 1	< 0.01	< 1	< 0.01	0.02	< 1	11	< 1	> 10.0	< 1	< 0.001	< 3	< 5
C-666742	< 0.3	< 0.01	< 3	18	< 1	< 2	0.14	< 0.3	< 1	1	< 1	< 0.01	< 1	< 0.01	< 0.01	< 1	3	< 1	> 10.0	< 1	< 0.001	< 3	< 5
C-666743	< 0.3	< 0.01	< 3	< 7	< 1	< 2	0.15	< 0.3	< 1	5	< 1	< 0.01	< 1	< 0.01	< 0.01	< 1	8	< 1	> 10.0	< 1	< 0.001	< 3	< 5
C-666744	< 0.3	< 0.01	< 3	< 7	< 1	< 2	7.63	< 0.3	< 1	7	< 1	< 0.01	1	< 0.01	0.06	5	14	< 1	> 10.0	< 1	< 0.001	< 3	< 5
C-666745	< 0.3	< 0.01	< 3	< 7	< 1	< 2	0.58	< 0.3	< 1	2	< 1	< 0.01	< 1	< 0.01	0.01	< 1	4	< 1	> 10.0	< 1	< 0.001	< 3	< 5
C-666746	< 0.3	< 0.01	< 3	< 7	< 1	< 2	0.42	< 0.3	< 1	4	< 1	< 0.01	< 1	< 0.01	< 0.01	< 1	8	< 1	> 10.0	< 1	< 0.001	< 3	< 5
C-666747	< 0.3	< 0.01	< 3	< 7	< 1	< 2	2.31	< 0.3	< 1	2	< 1	< 0.01	< 1	0.01	0.07	3	5	< 1	> 10.0	< 1	< 0.001	< 3	< 5
C-666748	< 0.3	< 0.01	< 3	15	< 1	< 2	2.91	< 0.3	< 1	6	< 1	< 0.01	< 1	< 0.01	0.01	< 1	4	< 1	> 10.0	< 1	< 0.001	< 3	< 5
C-666749	< 0.3	< 0.01	< 3	9	< 1	< 2	0.37	< 0.3	< 1	3	< 1	< 0.01	< 1	< 0.01	0.01	< 1	6	< 1	> 10.0	< 1	< 0.001	< 3	< 5
C-666750	< 0.3	< 0.01	< 3	< 7	< 1	< 2	0.40	< 0.3	< 1	4	< 1	< 0.01	< 1	< 0.01	0.02	< 1	7	< 1	> 10.0	< 1	< 0.001	< 3	< 5
C-666751	< 0.3	< 0.01	< 3	< 7	< 1	< 2	0.55	< 0.3	< 1	3	< 1	< 0.01	< 1	< 0.01	0.01	< 1	22	< 1	> 10.0	< 1	< 0.001	< 3	< 5
C-666752	< 0.3	< 0.01	< 3	< 7	< 1	< 2	1.18	< 0.3	< 1	6	< 1	< 0.01	< 1	< 0.01	0.02	2	7	< 1	> 10.0	< 1	< 0.001	< 3	< 5
C-666753	< 0.3	< 0.01	< 3	9	< 1	< 2	1.56	< 0.3	< 1	4	< 1	< 0.01	< 1	< 0.01	0.04	< 1	5	< 1	> 10.0	< 1	< 0.001	< 3	< 5
C-666754	< 0.3	< 0.01	< 3	8	< 1	< 2	0.06	< 0.3	< 1	4	< 1	< 0.01	< 1	< 0.01	< 0.01	< 1	8	< 1	> 10.0	< 1	< 0.001	< 3	< 5
C-666755	< 0.3	< 0.01	< 3	< 7	< 1	< 2	7.15	< 0.3	< 1	6	< 1	< 0.01	< 1	< 0.01	0.16	2	11	< 1	> 10.0	< 1	< 0.001	< 3	< 5
C-666756	< 0.3	< 0.01	< 3	8	< 1	< 2	0.60	< 0.3	< 1	4	< 1	< 0.01	< 1	< 0.01	< 0.01	< 1	7	< 1	> 10.0	< 1	< 0.001	10	< 5
C-666757	< 0.3	< 0.01	< 3	24	< 1	< 2	0.14	< 0.3	< 1	3	< 1	< 0.01	< 1	< 0.01	0.02	< 1	3	< 1	> 10.0	< 1	< 0.001	< 3	< 5
C-666758	< 0.3	< 0.01	< 3	21	< 1	< 2	0.08	< 0.3	< 1	1	< 1	< 0.01	< 1	< 0.01	0.02	< 1	3	< 1	> 10.0	< 1	< 0.001	< 3	< 5
C-666759	< 0.3	< 0.01	< 3	< 7	< 1	< 2	8.24	< 0.3	< 1	5	< 1	0.01	1	< 0.01	1.62	< 1	11	< 1	0.04	< 1	< 0.001	< 3	< 5
C-666760	< 0.3	0.76	50	55	< 1	< 2	19.4	< 0.3	2	14	25	1.38	3	0.69	10.1	20	100	17	0.10	32	0.008	38	< 5
C-666761	< 0.3	0.27	3	19	< 1	< 2	23.0	< 0.3	< 1	7	7	0.30	2	0.25	11.5	6	100	< 1	0.29	6	0.002	< 3	< 5
C-666762	< 0.3	0.14	< 3	13	< 1	< 2	23.0	< 0.3	< 1	3	4	0.21	2	0.13	11.7	3	92	< 1	0.25	5	0.003	< 3	< 5
C-666678	< 0.3	0.67	5	49	< 1	< 2	9.12	< 0.3	2	11	6	0.34	3	0.45	1.95	33	37	< 1	> 10.0	7	0.008	< 3	< 5
C-666682	< 0.3	< 0.01	< 3	8	< 1	< 2	0.41	< 0.3	< 1	17	< 1	< 0.01	< 1	0.04	< 0.01	< 1	9	< 1	> 10.0	< 1	< 0.001	< 3	< 5
C-666695	< 0.3	< 0.01	< 3	18	< 1	< 2	0.01	< 0.3	< 1	1	< 1	< 0.01	< 1	0.03	< 0.01	< 1	7	< 1	> 10.0	< 1	< 0.001	< 3	< 5
C-666708	< 0.3	< 0.01	< 3	10	< 1	< 2	8.97	< 0.3	< 1	9	< 1	< 0.01	1	0.01	1.30	8	8	< 1	0.12	< 1	< 0.001	< 3	< 5

Analyte Symbol	S	Sc	Sr	Te	Ti	Tl	U	V	W	Y	Zn	Zr
Unit Symbol	%	ppm	ppm	ppm	%	ppm	ppm	ppm	ppm	ppm	ppm	ppm
Lower Limit	0.01	4	1	2	0.01	5	10	2	5	1	1	5
Method Code	TD-ICP	TD-ICP	TD-ICP	TD-ICP	TD-ICP	TD-ICP	TD-ICP	TD-ICP	TD-ICP	TD-ICP	TD-ICP	TD-ICP
C-666568	0.12	< 4	214	< 2	0.01	< 5	30	< 2	< 5	1	3	< 5
C-666569	0.14	< 4	248	< 2	0.01	< 5	10	< 2	< 5	< 1	3	< 5
C-666570	0.09	< 4	200	< 2	0.01	< 5	20	< 2	< 5	2	11	< 5
C-666571	0.16	< 4	223	< 2	< 0.01	< 5	30	< 2	< 5	< 1	17	< 5
C-666572	0.19	< 4	173	< 2	0.02	< 5	30	3	< 5	2	47	7
C-666573	0.12	< 4	280	< 2	0.01	< 5	20	2	< 5	1	7	< 5
C-666574	0.30	< 4	455	< 2	0.09	< 5	10	25	< 5	3	15	21
C-666575	0.11	< 4	362	< 2	0.02	< 5	10	4	< 5	2	6	< 5
C-666576	0.12	< 4	356	< 2	0.02	< 5	10	4	< 5	2	5	< 5
C-666577	0.13	< 4	391	< 2	0.02	< 5	10	4	< 5	3	5	< 5
C-666578	0.17	< 4	332	< 2	0.03	< 5	10	6	< 5	3	7	7
C-666579	0.14	< 4	343	< 2	0.02	< 5	10	4	< 5	3	5	< 5
C-666580	0.26	< 4	347	< 2	0.02	< 5	10	4	< 5	3	6	< 5
C-666581	0.30	< 4	127	< 2	0.03	< 5	30	9	< 5	4	8	6
C-666582	0.32	< 4	167	< 2	0.08	< 5	20	21	< 5	5	16	20
C-666583	0.71	6	231	< 2	0.17	< 5	< 10	41	< 5	9	24	43
C-666584	0.84	6	319	< 2	0.18	< 5	< 10	40	< 5	8	29	41
C-666585	0.56	5	272	< 2	0.13	< 5	< 10	33	< 5	6	25	30
C-666586	0.34	15	384	< 2	0.26	< 5	< 10	68	< 5	13	63	75
C-666587	0.05	9	267	< 2	0.12	< 5	< 10	30	< 5	10	39	34
C-666588	0.29	6	278	< 2	0.15	< 5	< 10	40	< 5	8	27	36
C-666589	1.03	< 4	164	< 2	0.10	< 5	60	28	< 5	5	17	23
C-666590	0.13	< 4	26	< 2	< 0.01	< 5	100	< 2	< 5	< 1	3	< 5
C-666591	0.25	< 4	94	< 2	0.03	< 5	80	9	< 5	2	7	8
C-666592	0.08	< 4	15	< 2	< 0.01	< 5	100	< 2	< 5	< 1	1	< 5
C-666593	0.28	< 4	30	< 2	< 0.01	< 5	90	< 2	< 5	< 1	< 1	< 5
C-666594	0.07	< 4	12	< 2	< 0.01	< 5	90	< 2	< 5	< 1	3	< 5
C-666595	0.13	< 4	23	< 2	< 0.01	< 5	90	< 2	< 5	< 1	3	< 5
C-666596	0.01	< 4	13	< 2	< 0.01	< 5	40	2	< 5	< 1	2	< 5
C-666597	0.02	< 4	61	< 2	0.02	< 5	60	6	< 5	1	4	< 5
C-666598	0.03	< 4	7	< 2	< 0.01	< 5	100	< 2	< 5	< 1	4	< 5
C-666599	0.06	< 4	13	< 2	0.01	< 5	60	3	< 5	< 1	2	< 5
C-666600	0.18	8	130	< 2	0.18	< 5	< 10	37	< 5	13	34	65
C-666601	0.53	< 4	110	< 2	0.08	< 5	70	18	< 5	4	12	18
C-666602	0.01	< 4	4	< 2	< 0.01	< 5	100	< 2	< 5	< 1	< 1	< 5
C-666603	0.07	< 4	10	< 2	< 0.01	< 5	50	2	< 5	< 1	2	< 5
C-666604	0.08	< 4	11	< 2	< 0.01	< 5	50	2	< 5	< 1	3	< 5
C-666605	0.04	< 4	4	< 2	< 0.01	< 5	100	< 2	< 5	< 1	< 1	< 5
C-666606	0.09	< 4	9	< 2	< 0.01	< 5	40	< 2	< 5	< 1	1	< 5
C-666607	0.02	< 4	3	< 2	< 0.01	< 5	80	< 2	< 5	< 1	< 1	< 5
C-666608	0.23	< 4	27	< 2	0.02	< 5	30	6	< 5	1	4	6
C-666609	0.05	< 4	7	< 2	< 0.01	< 5	30	2	< 5	< 1	2	< 5
C-666610	0.18	< 4	33	< 2	0.04	< 5	50	11	< 5	2	8	9
C-666611	< 0.01	< 4	2	< 2	< 0.01	< 5	50	< 2	< 5	< 1	< 1	< 5
C-666612	< 0.01	< 4	1	< 2	< 0.01	< 5	50	< 2	< 5	< 1	< 1	< 5
C-666613	0.06	< 4	10	< 2	< 0.01	< 5	20	3	< 5	< 1	2	< 5
C-666614	0.08	< 4	12	< 2	< 0.01	< 5	90	< 2	< 5	< 1	2	< 5
C-666615	0.02	< 4	5	< 2	< 0.01	< 5	100	< 2	< 5	< 1	< 1	< 5
C-666616	0.04	< 4	15	< 2	0.01	< 5	90	3	< 5	< 1	3	< 5
C-666617	0.04	< 4	7	< 2	< 0.01	< 5	80	< 2	< 5	< 1	1	< 5
C-666618	0.11	< 4	15	< 2	< 0.01	< 5	20	< 2	< 5	< 1	3	< 5

Analyte Symbol	S	Sc	Sr	Te	Ti	Tl	U	V	W	Y	Zn	Zr
Unit Symbol	%	ppm	ppm	ppm	%	ppm	ppm	ppm	ppm	ppm	ppm	ppm
Lower Limit	0.01	4	1	2	0.01	5	10	2	5	1	1	5
Method Code	TD-ICP	TD-ICP	TD-ICP	TD-ICP	TD-ICP	TD-ICP	TD-ICP	TD-ICP	TD-ICP	TD-ICP	TD-ICP	TD-ICP
C-666619	0.53	< 4	94	< 2	0.06	< 5	80	15	< 5	3	10	14
C-666620	< 0.01	< 4	7	< 2	< 0.01	< 5	100	< 2	< 5	< 1	3	< 5
C-666621	0.31	< 4	70	< 2	0.03	< 5	< 10	9	< 5	2	6	9
C-666622	0.09	< 4	10	< 2	< 0.01	< 5	30	< 2	< 5	< 1	1	< 5
C-666623	0.02	< 4	16	< 2	0.02	< 5	90	5	< 5	< 1	3	< 5
C-666624	< 0.01	< 4	6	< 2	< 0.01	< 5	80	< 2	< 5	< 1	2	< 5
C-666625	1.22	5	174	< 2	0.13	< 5	60	32	< 5	7	22	34
C-666627	0.47	< 4	31	< 2	< 0.01	< 5	100	< 2	< 5	< 1	1	< 5
C-666628	0.20	< 4	14	< 2	< 0.01	< 5	90	< 2	< 5	< 1	< 1	< 5
C-666629	0.01	< 4	7	< 2	< 0.01	< 5	90	< 2	< 5	< 1	1	< 5
C-666630	0.06	< 4	9	< 2	< 0.01	< 5	90	< 2	< 5	< 1	< 1	< 5
C-666631	0.42	8	173	< 2	0.23	< 5	20	57	< 5	11	32	57
C-666632	0.96	< 4	53	< 2	0.01	< 5	100	3	< 5	< 1	3	< 5
C-666633	0.28	< 4	27	< 2	< 0.01	< 5	50	2	< 5	< 1	2	< 5
C-666634	0.12	< 4	9	< 2	< 0.01	< 5	90	< 2	< 5	< 1	< 1	< 5
C-666635	0.03	< 4	10	< 2	< 0.01	< 5	100	< 2	< 5	< 1	1	< 5
C-666636	0.09	< 4	8	< 2	< 0.01	< 5	90	< 2	< 5	< 1	< 1	< 5
C-666637	0.02	< 4	7	< 2	< 0.01	< 5	90	< 2	< 5	< 1	1	< 5
C-666638	< 0.01	< 4	10	< 2	< 0.01	< 5	90	< 2	< 5	< 1	1	< 5
C-666639	0.01	< 4	5	< 2	< 0.01	< 5	80	< 2	< 5	< 1	1	< 5
C-666640	0.17	< 4	14	< 2	< 0.01	< 5	90	< 2	< 5	< 1	< 1	< 5
C-666641	0.07	< 4	13	< 2	< 0.01	< 5	90	< 2	< 5	< 1	1	< 5
C-666642	0.10	< 4	9	< 2	< 0.01	< 5	90	< 2	< 5	< 1	< 1	< 5
C-666643	0.07	< 4	8	< 2	< 0.01	< 5	80	< 2	< 5	< 1	< 1	< 5
C-666644	0.11	< 4	12	< 2	< 0.01	< 5	80	< 2	< 5	< 1	< 1	< 5
C-666645	< 0.01	< 4	3	< 2	< 0.01	< 5	40	< 2	< 5	< 1	1	< 5
C-666646	< 0.01	< 4	12	< 2	< 0.01	< 5	90	2	< 5	< 1	5	< 5
C-666647	0.42	< 4	31	< 2	< 0.01	< 5	60	< 2	< 5	< 1	1	< 5
C-666648	0.15	< 4	15	< 2	< 0.01	< 5	70	2	< 5	< 1	1	< 5
C-666649	0.19	< 4	21	< 2	0.01	< 5	20	3	< 5	< 1	3	< 5
C-666650	0.12	< 4	13	< 2	< 0.01	< 5	80	< 2	< 5	< 1	3	< 5
C-666651	< 0.01	< 4	5	< 2	< 0.01	< 5	90	< 2	< 5	< 1	2	< 5
C-666652	< 0.01	< 4	3	< 2	< 0.01	< 5	70	< 2	< 5	< 1	1	< 5
C-666653	< 0.01	< 4	5	< 2	< 0.01	< 5	90	< 2	< 5	< 1	< 1	< 5
C-666654	< 0.01	< 4	3	< 2	< 0.01	< 5	90	< 2	< 5	< 1	< 1	< 5
C-666655	< 0.01	< 4	1	< 2	< 0.01	< 5	90	< 2	< 5	< 1	< 1	< 5
C-666656	0.05	< 4	4	2	< 0.01	< 5	60	< 2	< 5	< 1	< 1	< 5
C-666657	0.41	< 4	15	< 2	< 0.01	< 5	100	< 2	< 5	< 1	< 1	< 5
C-666658	0.01	< 4	1	< 2	< 0.01	< 5	< 10	< 2	< 5	< 1	1	< 5
C-666659	0.34	< 4	11	< 2	< 0.01	< 5	100	< 2	< 5	< 1	< 1	< 5
C-666660	9.76	< 4	557	< 2	0.06	< 5	< 10	12	< 5	2	8	11
C-666661	0.42	< 4	20	< 2	< 0.01	< 5	100	< 2	< 5	< 1	< 1	< 5
C-666662	0.18	< 4	8	< 2	< 0.01	< 5	90	< 2	< 5	< 1	< 1	< 5
C-666663	0.14	< 4	8	< 2	< 0.01	< 5	100	< 2	< 5	< 1	< 1	< 5
C-666664	0.38	< 4	21	< 2	< 0.01	< 5	90	< 2	< 5	< 1	< 1	< 5
C-666665	0.14	< 4	8	< 2	< 0.01	< 5	100	< 2	< 5	< 1	< 1	< 5
C-666667	2.42	< 4	114	< 2	0.01	< 5	90	3	< 5	< 1	3	< 5
C-666668	0.47	< 4	20	< 2	< 0.01	< 5	100	< 2	< 5	< 1	1	< 5
C-666669	4.21	< 4	177	< 2	< 0.01	< 5	90	2	< 5	< 1	2	< 5
C-666670	1.61	< 4	68	< 2	< 0.01	< 5	90	< 2	< 5	< 1	< 1	< 5
C-666671	10.4	< 4	765	< 2	0.06	< 5	< 10	13	< 5	1	9	8

Analyte Symbol	S	Sc	Sr	Te	Ti	Tl	U	V	W	Y	Zn	Zr
Unit Symbol	%	ppm	ppm	ppm	%	ppm	ppm	ppm	ppm	ppm	ppm	ppm
Lower Limit	0.01	4	1	2	0.01	5	10	2	5	1	1	5
Method Code	TD-ICP	TD-ICP	TD-ICP	TD-ICP	TD-ICP	TD-ICP	TD-ICP	TD-ICP	TD-ICP	TD-ICP	TD-ICP	TD-ICP
C-666672	2.11	< 4	97	< 2	< 0.01	< 5	90	< 2	< 5	< 1	< 1	< 5
C-666673	2.31	< 4	118	< 2	< 0.01	< 5	90	2	< 5	< 1	5	< 5
C-666674	1.50	< 4	79	< 2	< 0.01	< 5	90	< 2	< 5	< 1	2	< 5
C-666675	0.45	< 4	22	2	< 0.01	< 5	100	< 2	< 5	< 1	< 1	< 5
C-666676	0.14	< 4	10	< 2	< 0.01	< 5	100	< 2	< 5	< 1	< 1	< 5
C-666677	0.03	< 4	4	< 2	< 0.01	< 5	90	< 2	< 5	< 1	< 1	< 5
C-666679	0.53	< 4	25	< 2	< 0.01	< 5	90	< 2	< 5	< 1	< 1	< 5
C-666680	0.20	< 4	10	< 2	< 0.01	< 5	90	< 2	< 5	< 1	< 1	< 5
C-666681	0.26	< 4	14	< 2	< 0.01	< 5	90	< 2	< 5	< 1	< 1	< 5
C-666683	0.12	< 4	7	< 2	< 0.01	< 5	90	< 2	< 5	< 1	< 1	< 5
C-666684	0.17	< 4	8	< 2	< 0.01	< 5	100	< 2	< 5	< 1	< 1	< 5
C-666685	0.09	< 4	7	< 2	< 0.01	< 5	90	< 2	< 5	< 1	2	< 5
C-666686	< 0.01	< 4	3	< 2	< 0.01	< 5	90	< 2	< 5	< 1	< 1	< 5
C-666687	0.41	< 4	20	< 2	< 0.01	< 5	90	< 2	< 5	< 1	< 1	< 5
C-666688	0.13	< 4	34	< 2	0.03	< 5	80	7	< 5	2	6	9
C-666689	0.41	< 4	27	< 2	< 0.01	< 5	90	< 2	< 5	< 1	1	< 5
C-666690	0.03	< 4	6	< 2	< 0.01	< 5	90	< 2	< 5	< 1	< 1	< 5
C-666691	0.33	< 4	19	< 2	< 0.01	< 5	90	< 2	< 5	< 1	< 1	< 5
C-666692	0.62	< 4	36	< 2	< 0.01	< 5	100	< 2	< 5	< 1	2	< 5
C-666693	0.27	< 4	16	< 2	< 0.01	< 5	90	< 2	< 5	< 1	< 1	< 5
C-666696	0.05	< 4	6	< 2	< 0.01	< 5	90	< 2	< 5	< 1	< 1	< 5
C-666697	1.59	< 4	82	< 2	< 0.01	< 5	90	< 2	< 5	< 1	< 1	< 5
C-666698	8.23	< 4	356	< 2	< 0.01	< 5	60	< 2	< 5	< 1	1	< 5
C-666699	8.54	< 4	443	< 2	< 0.01	< 5	60	< 2	< 5	< 1	1	< 5
C-666700	9.28	< 4	349	< 2	< 0.01	< 5	60	< 2	< 5	< 1	< 1	< 5
C-666701	9.89	< 4	380	< 2	< 0.01	< 5	50	< 2	< 5	< 1	< 1	< 5
C-666702	10.4	< 4	845	< 2	< 0.01	< 5	< 10	< 2	< 5	< 1	1	< 5
C-666703	2.28	< 4	241	< 2	< 0.01	< 5	10	< 2	< 5	< 1	3	< 5
C-666704	10.4	< 4	863	< 2	< 0.01	< 5	< 10	< 2	< 5	< 1	< 1	< 5
C-666705	1.19	< 4	187	< 2	< 0.01	< 5	10	< 2	< 5	< 1	2	< 5
C-666706	0.37	< 4	107	< 2	< 0.01	< 5	20	< 2	< 5	< 1	2	< 5
C-666707	11.9	< 4	760	< 2	< 0.01	< 5	< 10	< 2	< 5	< 1	2	< 5
C-666709	5.24	< 4	390	< 2	< 0.01	< 5	20	< 2	< 5	< 1	1	< 5
C-666710	10.6	< 4	847	< 2	< 0.01	< 5	< 10	< 2	< 5	< 1	< 1	< 5
C-666711	8.30	< 4	829	< 2	< 0.01	< 5	< 10	< 2	< 5	< 1	1	< 5
C-666712	11.8	< 4	912	< 2	< 0.01	< 5	< 10	< 2	< 5	< 1	< 1	< 5
C-666713	10.2	< 4	861	< 2	< 0.01	< 5	< 10	< 2	< 5	< 1	< 1	< 5
C-666715	4.80	< 4	432	< 2	< 0.01	< 5	20	< 2	< 5	< 1	2	< 5
C-666716	9.16	< 4	857	< 2	< 0.01	< 5	< 10	< 2	< 5	< 1	< 1	< 5
C-666717	5.86	< 4	650	< 2	< 0.01	< 5	< 10	< 2	< 5	< 1	2	< 5
C-666718	9.67	< 4	943	< 2	< 0.01	< 5	< 10	< 2	< 5	< 1	< 1	< 5
C-666719	9.62	< 4	886	< 2	< 0.01	< 5	< 10	< 2	< 5	< 1	< 1	< 5
C-666720	6.98	< 4	698	< 2	< 0.01	< 5	< 10	< 2	< 5	< 1	< 1	< 5
C-666721	6.99	< 4	806	< 2	< 0.01	< 5	< 10	< 2	< 5	< 1	< 1	< 5
C-666722	8.42	< 4	871	< 2	< 0.01	< 5	< 10	< 2	< 5	< 1	< 1	< 5
C-666723	8.08	< 4	662	< 2	< 0.01	< 5	< 10	< 2	< 5	< 1	< 1	< 5
C-666725	3.09	< 4	157	< 2	< 0.01	< 5	90	< 2	< 5	< 1	< 1	< 5
C-666726	5.81	< 4	268	< 2	< 0.01	< 5	80	< 2	< 5	< 1	4	< 5
C-666727	2.62	< 4	123	< 2	< 0.01	< 5	90	< 2	< 5	< 1	< 1	< 5
C-666728	< 0.01	< 4	2	< 2	< 0.01	< 5	90	< 2	< 5	< 1	< 1	< 5
C-666729	0.02	< 4	1	< 2	< 0.01	< 5	100	< 2	< 5	< 1	< 1	< 5

Analyte Symbol	S	Sc	Sr	Te	Ti	Tl	U	V	W	Y	Zn	Zr
Unit Symbol	%	ppm	ppm	ppm	%	ppm	ppm	ppm	ppm	ppm	ppm	ppm
Lower Limit	0.01	4	1	2	0.01	5	10	2	5	1	1	5
Method Code	TD-ICP	TD-ICP	TD-ICP	TD-ICP	TD-ICP	TD-ICP	TD-ICP	TD-ICP	TD-ICP	TD-ICP	TD-ICP	TD-ICP
C-666730	0.62	< 4	33	< 2	< 0.01	< 5	90	< 2	< 5	< 1	< 1	< 5
C-666731	0.33	< 4	15	< 2	< 0.01	< 5	< 10	< 2	< 5	< 1	< 1	< 5
C-666732	0.11	< 4	6	< 2	< 0.01	< 5	90	< 2	< 5	< 1	< 1	< 5
C-666734	0.03	< 4	3	< 2	< 0.01	< 5	100	< 2	< 5	< 1	< 1	< 5
C-666735	0.83	< 4	46	< 2	< 0.01	< 5	90	< 2	< 5	< 1	< 1	< 5
C-666736	2.51	< 4	114	< 2	< 0.01	< 5	90	< 2	< 5	< 1	< 1	< 5
C-666737	0.98	< 4	46	< 2	< 0.01	< 5	90	< 2	< 5	< 1	< 1	< 5
C-666738	1.55	< 4	83	< 2	< 0.01	< 5	90	< 2	< 5	< 1	< 1	< 5
C-666739	0.50	< 4	27	< 2	< 0.01	< 5	100	< 2	< 5	< 1	< 1	< 5
C-666740	0.05	< 4	4	< 2	< 0.01	< 5	90	< 2	< 5	< 1	< 1	< 5
C-666741	1.40	< 4	67	< 2	< 0.01	< 5	90	< 2	< 5	< 1	< 1	< 5
C-666742	0.10	< 4	7	< 2	< 0.01	< 5	90	< 2	< 5	< 1	< 1	< 5
C-666743	0.11	< 4	6	< 2	< 0.01	< 5	90	< 2	< 5	< 1	< 1	< 5
C-666744	7.08	< 4	325	< 2	< 0.01	< 5	60	< 2	< 5	< 1	< 1	< 5
C-666745	0.45	< 4	24	4	< 0.01	< 5	90	< 2	< 5	< 1	< 1	< 5
C-666746	0.32	< 4	18	< 2	< 0.01	< 5	90	< 2	< 5	< 1	< 1	< 5
C-666747	1.92	< 4	87	< 2	< 0.01	< 5	90	< 2	< 5	< 1	< 1	< 5
C-666748	2.55	< 4	104	< 2	< 0.01	< 5	90	< 2	< 5	< 1	< 1	< 5
C-666749	0.27	< 4	18	< 2	< 0.01	< 5	90	< 2	< 5	< 1	< 1	< 5
C-666750	0.30	< 4	21	< 2	< 0.01	< 5	90	< 2	< 5	< 1	< 1	< 5
C-666751	0.42	< 4	23	< 2	< 0.01	< 5	90	< 2	< 5	< 1	< 1	< 5
C-666752	0.94	< 4	50	< 2	< 0.01	< 5	90	< 2	< 5	< 1	< 1	< 5
C-666753	1.22	< 4	62	< 2	< 0.01	< 5	90	< 2	< 5	< 1	< 1	< 5
C-666754	0.04	< 4	3	< 2	< 0.01	< 5	90	< 2	< 5	< 1	< 1	< 5
C-666755	6.39	< 4	276	< 2	< 0.01	< 5	70	< 2	< 5	< 1	< 1	< 5
C-666756	0.46	< 4	21	< 2	< 0.01	< 5	90	< 2	< 5	< 1	< 1	< 5
C-666757	0.07	< 4	10	< 2	< 0.01	< 5	90	< 2	< 5	< 1	2	< 5
C-666758	0.05	< 4	4	< 2	< 0.01	< 5	90	< 2	< 5	< 1	< 1	< 5
C-666759	4.99	< 4	709	< 2	< 0.01	< 5	< 10	< 2	< 5	< 1	< 1	< 5
C-666760	1.34	< 4	75	< 2	0.04	< 5	10	17	< 5	3	5	10
C-666761	0.43	< 4	87	< 2	0.01	< 5	< 10	2	< 5	1	4	< 5
C-666762	0.44	< 4	93	< 2	< 0.01	< 5	< 10	< 2	< 5	1	7	< 5
C-666678	6.43	< 4	348	< 2	0.04	< 5	50	8	< 5	2	6	8
C-666682	0.32	< 4	17	< 2	< 0.01	< 5	90	< 2	< 5	< 1	2	< 5
C-666695	< 0.01	< 4	< 1	< 2	< 0.01	< 5	90	< 2	< 5	< 1	< 1	< 5
C-666708	6.21	< 4	755	< 2	< 0.01	< 5	< 10	< 2	< 5	< 1	< 1	< 5

Analyte Symbol	Ag	Al	As	Ba	Be	Bi	Ca	Cd	Co	Cr	Cu	Fe	Ga	K	Mg	Li	Mn	Mo	Na	Ni	P	Pb	Sb
Unit Symbol	ppm	%	ppm	ppm	ppm	ppm	%	ppm	ppm	ppm	ppm	%	ppm	%	%	ppm	ppm	ppm	%	ppm	%	ppm	ppm
Lower Limit	0.3	0.01	3	7	1	2	0.01	0.3	1	1	1	0.01	1	0.01	0.01	1	1	1	0.01	1	0.001	3	5
Method Code	TD-ICP	TD-ICP	TD-ICP	TD-ICP	TD-ICP	TD-ICP	TD-ICP	TD-ICP	TD-ICP	TD-ICP	TD-ICP	TD-ICP	TD-ICP	TD-ICP	TD-ICP	TD-ICP	TD-ICP	TD-ICP	TD-ICP	TD-ICP	TD-ICP	TD-ICP	TD-ICP
OREAS 101b (4 Acid) Meas									44		397	10.3		1.79	1.20		891	18		9	0.110	19	
OREAS 101b (4 Acid) Cert									45		412	10.7		2.36	1.23		927	20.1		8.2	0.1118	23	
OREAS 101b (4 Acid) Meas									45		413	10.9		2.27	1.25		920	19		10	0.119	19	
OREAS 101b (4 Acid) Cert									45		412	10.7		2.36	1.23		927	20.1		8.2	0.1118	23	
OREAS 101b (4 Acid) Meas									46		425	10.2		2.26	1.24		959	20		10	0.118	18	
OREAS 101b (4 Acid) Cert									45		412	10.7		2.36	1.23		927	20.1		8.2	0.1118	23	
OREAS 98 (4 Acid) Meas	40.6					57			112		> 10000											263	6
OREAS 98 (4 Acid) Cert	45.1					97.2			121		14800 0.0											345	20.1
OREAS 98 (4 Acid) Meas	42.3					74			119		> 10000											273	11
OREAS 98 (4 Acid) Cert	45.1					97.2			121		14800 0.0											345	20.1
OREAS 903 (4 Acid) Meas	0.4	5.80	54	193	5	2	0.66	< 0.3	144	66	6400	4.40	16	2.41	0.75	18	686	4	0.03	56	0.104	6	< 5
OREAS 903 (4 Acid) Cert	0.432	5.89	49.7	197	4.42	8.90	0.625	0.200	131	73.0	6520	4.16	15.0	3.31	0.714	18.3	690	4.32	0.0300	54.0	0.107	11.3	1.57
OREAS 903 (4 Acid) Meas	0.5	5.95	53	217	5	13	0.69	< 0.3	151	82	6500	4.52	16	3.06	0.77	19	726	5	0.03	59	0.111	7	< 5
OREAS 903 (4 Acid) Cert	0.432	5.89	49.7	197	4.42	8.90	0.625	0.200	131	73.0	6520	4.16	15.0	3.31	0.714	18.3	690	4.32	0.0300	54.0	0.107	11.3	1.57
OREAS 903 (4 Acid) Meas	0.7	6.15	55	224	5	4	0.69	0.3	154	82	6800	4.47	16	3.31	0.77	19	767	5	0.05	65	0.123	8	< 5
OREAS 903 (4 Acid) Cert	0.432	5.89	49.7	197	4.42	8.90	0.625	0.200	131	73.0	6520	4.16	15.0	3.31	0.714	18.3	690	4.32	0.0300	54.0	0.107	11.3	1.57
OREAS 96 (4 Acid) Meas	11.2					27			49		> 10000											83	5
OREAS 96 (4 Acid) Cert	11.5					26.3			49.9		39300											101	5.09
OREAS 96 (4 Acid) Meas	11.3					39			48		> 10000											83	< 5
OREAS 96 (4 Acid) Cert	11.5					26.3			49.9		39300											101	5.09
OREAS 96 (4 Acid) Meas	11.5					16			51		> 10000											88	< 5
OREAS 96 (4 Acid) Cert	11.5					26.3			49.9		39300											101	5.09
OREAS 96 (4 Acid) Meas	11.2					7			51		> 10000											88	< 5
OREAS 96 (4 Acid) Cert	11.5					26.3			49.9		39300											101	5.09
OREAS 520 (4 Acid) Meas	0.8	5.16	152		< 1	< 2	3.95		185	39	2840	17.0	18	3.31	1.21	17	2240	62	1.23	74	0.076	< 3	< 5
OREAS 520 (4 Acid) Cert	0.450	5.63	153		1.06	2.94	4.10		203	36.4	2930	16.4	18.7	3.46	1.19	16.9	2420	65.0	1.35	76.0	0.0740	5.85	3.21
OREAS 520 (4 Acid) Meas	0.8	5.52	134		< 1	< 2	4.09		198	38	3010	16.4	20	3.44	1.22	18	2360	60	1.38	78	0.075	< 3	< 5
OREAS 520 (4 Acid) Cert	0.450	5.63	153		1.06	2.94	4.10		203	36.4	2930	16.4	18.7	3.46	1.19	16.9	2420	65.0	1.35	76.0	0.0740	5.85	3.21
Oreas 72b (4 Acid) Meas	0.4	4.43	140	326	< 1	< 2	2.64	0.4	122	539	214	6.94	10	1.06	9.17	32	929	4	0.96	6120	0.026	10	< 5

Analyte Symbol	Ag	Al	As	Ba	Be	Bi	Ca	Cd	Co	Cr	Cu	Fe	Ga	K	Mg	Li	Mn	Mo	Na	Ni	P	Pb	Sb
Unit Symbol	ppm	%	ppm	ppm	ppm	ppm	%	ppm	ppm	ppm	ppm	%	ppm	%	%	ppm	ppm	ppm	%	ppm	%	ppm	ppm
Lower Limit	0.3	0.01	3	7	1	2	0.01	0.3	1	1	1	0.01	1	0.01	0.01	1	1	1	0.01	1	0.001	3	5
Method Code	TD-ICP	TD-ICP	TD-ICP	TD-ICP	TD-ICP	TD-ICP	TD-ICP	TD-ICP	TD-ICP	TD-ICP	TD-ICP	TD-ICP	TD-ICP	TD-ICP	TD-ICP	TD-ICP	TD-ICP	TD-ICP	TD-ICP	TD-ICP	TD-ICP	TD-ICP	TD-ICP
Oreas 72b (4 Acid) Cert	0.230	4.79	146	330	1.02	0.680	2.79	0.310	131	771	222	6.84	11.7	1.14	9.59	33.3	1010	4.01	1.01	6860	0.0260	14.9	0.870
Oreas 72b (4 Acid) Meas	0.3	4.49	142	285	< 1	< 2	2.66	0.4	126	544	221	7.09	10	1.10	9.31	33	943	4	0.99	6210	0.026	10	< 5
Oreas 72b (4 Acid) Cert	0.230	4.79	146	330	1.02	0.680	2.79	0.310	131	771	222	6.84	11.7	1.14	9.59	33.3	1010	4.01	1.01	6860	0.0260	14.9	0.870
Oreas 72b (4 Acid) Meas	0.4	4.49	139	317	< 1	< 2	2.66	0.3	125	568	216	6.84	10	1.04	9.02	32	984	3	0.96	6480	0.026	10	< 5
Oreas 72b (4 Acid) Cert	0.230	4.79	146	330	1.02	0.680	2.79	0.310	131	771	222	6.84	11.7	1.14	9.59	33.3	1010	4.01	1.01	6860	0.0260	14.9	0.870
OREAS 45f (4-Acid) Meas		9.97	6	223	1	< 2	0.10		47	418	384	15.1	28	0.22	0.24	21	250	< 1	0.09	283	0.026	12	< 5
OREAS 45f (4-Acid) Cert		10.2	9.67	206	1.20	0.210	0.0960		44.5	417	363	14.7	26.7	0.224	0.229	20.4	220	2.27	0.0630	256	0.0300	14.7	0.640
OREAS 70b (Fusion) Meas		3.57	151	197			2.93		73	706	48	5.80	8	0.56	13.0	33	1070			1960	0.023	9	
OREAS 70b (Fusion) Cert		3.80	143	202			3.08		83	1250	52	5.66	9.71	0.617	13.61	35.3	1160.000			2220.000	0.025	13.2	
OREAS 683 (4 Acid) Meas	< 0.3	6.79		179	< 1	< 2	4.95	< 0.3	76	8040	401	7.62	12	0.48	8.32	7	1100	2	0.99	1060	0.049	7	
OREAS 683 (4 Acid) Cert	0.172	7.15		188	0.56	0.16	5.23	0.072	85	9940	404	7.32	13.8	0.507	8.63	6.51	1200	1.00	1.03	1180	0.050	10.2	
OREAS 683 (4 Acid) Meas	< 0.3	6.78		180	< 1	< 2	4.92	< 0.3	77	8450	414	7.76	12	0.49	8.43	7	1110	5	0.99	1080	0.049	4	
OREAS 683 (4 Acid) Cert	0.172	7.15		188	0.56	0.16	5.23	0.072	85	9940	404	7.32	13.8	0.507	8.63	6.51	1200	1.00	1.03	1180	0.050	10.2	
OREAS 683 (4 Acid) Meas	< 0.3	6.93		172	< 1	< 2	4.98	0.4	79	8560	410	7.70	12	0.48	8.29	7	1200	< 1	1.01	1140	0.047	6	
OREAS 683 (4 Acid) Cert	0.172	7.15		188	0.56	0.16	5.23	0.072	85	9940	404	7.32	13.8	0.507	8.63	6.51	1200	1.00	1.03	1180	0.050	10.2	
OREAS 681 (4 Acid) Meas	0.3	7.47		418	1	< 2	5.61		45	1320	286	7.87	16	1.33	5.11	13	1190	1	1.46	445	0.139	< 3	< 5
OREAS 681 (4 Acid) Cert	0.118	7.91		442	1.41	0.0980	5.98		51.0	1640	264	7.47	17.6	1.35	5.19	13.0	1310	1.38	1.61	503	0.141	10.2	0.240
OREAS 681 (4 Acid) Meas	0.3	7.60		426	1	< 2	5.67		46	1420	270	8.00	17	1.34	5.20	13	1210	1	1.49	445	0.139	4	< 5
OREAS 681 (4 Acid) Cert	0.118	7.91		442	1.41	0.0980	5.98		51.0	1640	264	7.47	17.6	1.35	5.19	13.0	1310	1.38	1.61	503	0.141	10.2	0.240
OREAS 681 (4 Acid) Meas	< 0.3	7.55		418	1	< 2	5.67		47	1310	259	7.65	16	1.20	4.95	13	1290	< 1	1.56	466	0.138	4	< 5
OREAS 681 (4 Acid) Cert	0.118	7.91		442	1.41	0.0980	5.98		51.0	1640	264	7.47	17.6	1.35	5.19	13.0	1310	1.38	1.61	503	0.141	10.2	0.240
OREAS 70b (4 Acid) Meas	< 0.3	3.61	142	199	< 1	< 2	2.88	0.4	72		48	5.78	8	0.58	13.1	34	1050	4	0.76	1910	0.022	8	< 5
OREAS 70b (4 Acid) Cert	0.2	3.87	148	202	1	0.8	3.05	0.4	78		52	5.52	10	0.62	13.4	34	1150	3	0.77	2180	0.022	10	0.6
OREAS 70b (4 Acid) Meas	< 0.3	3.71	137	194	< 1	< 2	2.93	0.5	75		53	5.75	9	0.57	13.1	34	1150	3	0.76	2100	0.022	9	< 5
OREAS 70b (4 Acid) Cert	0.2	3.87	148	202	1	0.8	3.05	0.4	78		52	5.52	10	0.62	13.4	34	1150	3	0.77	2180	0.022	10	0.6
OREAS 620 (4 Acid) Meas	38.3	6.82	51	83	2	2	1.72	159	13	22	1680	3.09	25	2.05	0.35	20	418	8	1.75	16	0.037	> 5000	5
OREAS 620 (4 Acid) Cert	38.5	6.72	50	2500	2	2	1.60	163	12	22	1730	2.94	24	2.63	0.34	20	440	9	1.94	15	0.035	7740	80
OREAS 620 (4 Acid) Meas	38.6	6.77	51	78	2	< 2	1.71	157	13	18	1710	3.14	25	2.27	0.35	20	410	8	1.81	16	0.037	> 5000	7
OREAS 620 (4 Acid) Cert	38.5	6.72	50	2500	2	2	1.60	163	12	22	1730	2.94	24	2.63	0.34	20	440	9	1.94	15	0.035	7740	80

Analyte Symbol	Ag	Al	As	Ba	Be	Bi	Ca	Cd	Co	Cr	Cu	Fe	Ga	K	Mg	Li	Mn	Mo	Na	Ni	P	Pb	Sb
Unit Symbol	ppm	%	ppm	ppm	ppm	ppm	%	ppm	ppm	ppm	ppm	%	ppm	%	%	ppm	ppm	ppm	%	ppm	%	ppm	ppm
Lower Limit	0.3	0.01	3	7	1	2	0.01	0.3	1	1	1	0.01	1	0.01	0.01	1	1	1	0.01	1	0.001	3	5
Method Code	TD-ICP	TD-ICP	TD-ICP	TD-ICP	TD-ICP	TD-ICP	TD-ICP	TD-ICP	TD-ICP	TD-ICP	TD-ICP	TD-ICP	TD-ICP	TD-ICP	TD-ICP	TD-ICP	TD-ICP	TD-ICP	TD-ICP	TD-ICP	TD-ICP	TD-ICP	TD-ICP
OREAS 620 (4 Acid) Meas	38.9	6.67	53	77	2	< 2	1.71	157	13	26	1700	3.12	25	2.17	0.35	20	401	16	1.78	16	0.036	> 5000	8
OREAS 620 (4 Acid) Cert	38.5	6.72	50	2500	2	2	1.60	163	12	22	1730	2.94	24	2.63	0.34	20	440	9.5	1.94	15	0.035	7740	80
OREAS 620 (4 Acid) Meas	39.1	6.62	46	67	2	< 2	1.75	163	14	18	1630	2.91	23	1.36	0.34	19	422	10	1.79	16	0.038	> 5000	11
OREAS 620 (4 Acid) Cert	38.5	6.72	50	2500	2	2	1.60	163	12	22	1730	2.94	24	2.63	0.34	20	440	9	1.94	15	0.035	7740	76
OREAS 753 (4 Acid) Meas		8.28	< 3	20	115	3	0.13	1.6	< 1	17	18	0.89	17	1.68	0.01	8930	767	4	1.97	13	0.117	7	< 5
OREAS 753 (4 Acid) Cert		8.22	5.33	18.2	118	2.20	0.113	1.54	0.96	20.8	18.4	0.839	16.1	1.93	0.011	9850.000	740.000	3.32	2.16	10.8	0.111	10.9	0.27
OREAS 753 (4 Acid) Meas		7.64	8	19	112	2	0.12	1.7	1	18	22	0.90	17	1.93	0.01	9150	802	4	2.01	12	0.111	7	< 5
OREAS 753 (4 Acid) Cert		8.22	5.33	18.2	118	2.20	0.113	1.54	0.96	20.8	18.4	0.839	16.1	1.93	0.011	9850.000	740.000	3.32	2.16	10.8	0.111	10.9	0.27
OREAS 753 (4 Acid) Meas		8.20	3	19	116	3	0.12	1.7	< 1	18	19	0.87	16	1.94	< 0.01	9100	790	3	2.04	12	0.108	6	< 5
OREAS 753 (4 Acid) Cert		8.22	5.33	18.2	118	2.20	0.113	1.54	0.96	20.8	18.4	0.839	16.1	1.93	0.011	9850.000	740.000	3.32	2.16	10.8	0.111	10.9	0.27
OREAS 603c (4 Acid) Meas	> 100	6.22	1570		2	79	1.04	31.6	15	21	> 10000	4.34	24	2.23	0.16	28	632	57	1.64	28	0.045	> 5000	33
OREAS 603c (4 Acid) Cert	294	6	1560		2.28	89	1	31.4	14.9	19.4	12100	4	23.3	2	0.162	27.6	660	60	2	26.9	0.043	10428	400
OREAS 603c (4 Acid) Meas	> 100	6.37	1600		2	98	1.10	31.8	15	21	> 10000	4.39	24	2.11	0.22	28	621	55	1.68	29	0.047	> 5000	25
OREAS 603c (4 Acid) Cert	294	6	1560		2.28	89	1	31.4	14.9	19.4	12100	4	23.3	2	0.162	27.6	660	60	2	26.9	0.043	10428	400
OREAS 601c (4 acid) Meas	52.6	6.54	419		2	20	1.02	2.8	5	21	1160	2.43	25	2.70	0.18	28	230	4	1.79	8	0.040	320	9
OREAS 601c (4 acid) Cert	50.3	7.06	390.000		2.46	21.1	0.953	2.77	4.99	17.5	1160.000	2.41	23.5	2.72	0.169	26.7	230.000	3.66	1.94	6.83	0.039	328	37.2
OREAS 601c (4 acid) Meas	52.2	6.84	417		2	21	1.04	2.9	5	15	1150	2.47	24	2.68	0.18	28	232	4	1.78	8	0.040	314	6
OREAS 601c (4 acid) Cert	50.3	7.06	390.000		2.46	21.1	0.953	2.77	4.99	17.5	1160.000	2.41	23.5	2.72	0.169	26.7	230.000	3.66	1.94	6.83	0.039	328	37.2
OREAS 601c (4 acid) Meas	51.9	6.86	408		2	18	1.03	3.1	5	16	1170	2.33	24	2.30	0.18	28	237	4	1.86	7	0.039	318	12
OREAS 601c (4 acid) Cert	50.3	7.06	390.000		2.46	21.1	0.953	2.77	4.99	17.5	1160.000	2.41	23.5	2.72	0.169	26.7	230.000	3.66	1.94	6.83	0.039	328	37.2
OREAS 601c (4 acid) Meas	52.2	6.73	403		2	16	1.01	3.0	5	16	1200	2.31	24	2.82	0.18	28	233	4	1.86	7	0.039	314	10
OREAS 601c (4 acid) Cert	50.3	7.06	390.000		2.46	21.1	0.953	2.77	4.99	17.5	1160.000	2.41	23.5	2.72	0.169	26.7	230.000	3.66	1.94	6.83	0.039	328	37.2
OREAS 504 (4 Acid) Meas	3.2										> 10000							616					
OREAS 504 (4 Acid) Cert	3.13										11370.000							643					
OREAS 504 (4 Acid) Meas	3.3										> 10000							630					
OREAS 504 (4 Acid) Cert	3.13										11370.000							643					
OREAS 504 (4 Acid) Meas	3.3										> 10000							637					
OREAS 504 (4 Acid) Cert	3.13										11370.000							643					
OREAS 504 (4 Acid) Meas	3.2										> 10000							665					

Analyte Symbol	Ag	Al	As	Ba	Be	Bi	Ca	Cd	Co	Cr	Cu	Fe	Ga	K	Mg	Li	Mn	Mo	Na	Ni	P	Pb	Sb
Unit Symbol	ppm	%	ppm	ppm	ppm	ppm	%	ppm	ppm	ppm	ppm	%	ppm	%	%	ppm	ppm	ppm	%	ppm	%	ppm	ppm
Lower Limit	0.3	0.01	3	7	1	2	0.01	0.3	1	1	1	0.01	1	0.01	0.01	1	1	1	0.01	1	0.001	3	5
Method Code	TD-ICP	TD-ICP	TD-ICP	TD-ICP	TD-ICP	TD-ICP	TD-ICP	TD-ICP	TD-ICP	TD-ICP	TD-ICP	TD-ICP	TD-ICP	TD-ICP	TD-ICP	TD-ICP	TD-ICP	TD-ICP	TD-ICP	TD-ICP	TD-ICP	TD-ICP	TD-ICP
OREAS 504 (4 Acid) Cert	3.13										11370.000							643					
GMO-04 Meas	1.9		7			97					233							7060				39	< 5
GMO-04 Cert	1.930		4.50			95.00					239.50							7949.0				45.7	8.88
GMO-04 Meas	2.2		6			103					257							6910				38	< 5
GMO-04 Cert	1.930		4.50			95.00					239.50							7949.0				45.7	8.88
GMO-04 Meas	2.6		4			87					255							7730				35	6
GMO-04 Cert	1.930		4.50			95.00					239.50							7949.0				45.7	8.88
OREAS 45h (4-Acid) Meas	0.5	7.35	7	354	1	< 2	0.14		85	630	785	20.0	21	0.20	0.25	14	390	2	0.09	415	0.021	5	< 5
OREAS 45h (4-Acid) Cert	0.147	7.99	16.9	332	1.09	0.17	0.135		88	602	767	19.52	21.3	0.205	0.238	13.1	380.000	1.55	0.090	423	0.023	11.9	0.63
C-666581 Orig	< 0.3	0.67	3	27	< 1	< 2	19.4	< 0.3	1	9	14	0.50	2	0.62	7.22	13	179	2	7.74	11	0.020	3	< 5
C-666581 Dup	< 0.3	0.65	< 3	27	< 1	< 2	18.7	< 0.3	1	9	14	0.49	2	0.61	7.11	13	177	2	7.63	11	0.020	< 3	< 5
C-666591 Orig	< 0.3	0.66	< 3	65	< 1	< 2	2.96	< 0.3	1	9	2	0.45	2	0.42	2.07	10	72	< 1	> 10.0	6	0.006	< 3	< 5
C-666591 Dup	< 0.3	0.64	< 3	65	< 1	< 2	2.98	< 0.3	1	11	3	0.43	2	0.40	2.02	9	71	< 1	> 10.0	7	0.006	< 3	< 5
C-666601 Orig	< 0.3	1.33	< 3	64	< 1	< 2	3.72	< 0.3	2	17	2	0.90	4	2.41	2.93	22	117	< 1	> 10.0	12	0.012	< 3	< 5
C-666601 Dup	< 0.3	1.38	< 3	65	< 1	< 2	3.83	< 0.3	2	17	2	0.93	4	2.28	3.02	23	116	< 1	> 10.0	12	0.013	< 3	< 5
C-666614 Orig	< 0.3	0.10	< 3	12	< 1	< 2	0.21	< 0.3	< 1	5	< 1	0.06	< 1	2.07	0.12	2	15	< 1	> 10.0	1	< 0.001	8	< 5
C-666614 Dup	< 0.3	0.10	< 3	12	< 1	< 2	0.20	< 0.3	< 1	4	8	0.06	< 1	2.16	0.12	2	11	< 1	> 10.0	1	< 0.001	7	< 5
C-666632 Orig	< 0.3	0.21	< 3	28	< 1	< 2	1.49	< 0.3	< 1	3	< 1	0.11	1	0.31	0.33	4	14	< 1	> 10.0	2	0.002	26	< 5
C-666632 Dup	< 0.3	0.21	< 3	28	< 1	< 2	1.47	< 0.3	< 1	4	8	0.11	1	0.32	0.33	4	15	< 1	> 10.0	3	0.002	23	< 5
C-666644 Orig	< 0.3	0.02	< 3	10	< 1	< 2	0.20	< 0.3	< 1	5	1	0.01	< 1	3.69	0.42	< 1	7	< 1	> 10.0	< 1	< 0.001	< 3	< 5
C-666644 Dup	< 0.3	0.02	< 3	11	< 1	< 2	0.22	< 0.3	< 1	4	< 1	0.01	< 1	4.22	0.45	< 1	5	< 1	> 10.0	< 1	< 0.001	< 3	< 5
C-666659 Orig	< 0.3	< 0.01	< 3	< 7	< 1	< 2	0.42	< 0.3	< 1	5	< 1	< 0.01	< 1	0.13	0.03	< 1	8	< 1	> 10.0	< 1	< 0.001	< 3	< 5
C-666659 Dup	< 0.3	< 0.01	< 3	< 7	< 1	< 2	0.42	< 0.3	< 1	3	< 1	< 0.01	< 1	0.12	0.03	< 1	7	< 1	> 10.0	< 1	< 0.001	< 3	< 5
C-666671 Orig	< 0.3	0.94	< 3	54	< 1	< 2	11.9	< 0.3	2	18	3	0.45	4	0.57	1.85	47	48	< 1	0.13	9	0.011	< 3	< 5
C-666671 Dup	< 0.3	0.96	< 3	54	< 1	< 2	12.5	< 0.3	3	15	3	0.46	3	0.57	1.85	47	47	< 1	0.13	9	0.011	< 3	< 5
C-666688 Orig	< 0.3	0.60	< 3	34	< 1	< 2	1.89	< 0.3	2	10	3	0.29	2	0.38	1.54	57	30	< 1	> 10.0	5	0.005	7	< 5
C-666688 Dup	< 0.3	0.60	< 3	35	< 1	< 2	1.91	< 0.3	1	11	3	0.29	2	0.39	1.57	57	31	< 1	> 10.0	6	0.005	7	< 5
C-666704 Orig	< 0.3	< 0.01	< 3	< 7	< 1	< 2	13.1	< 0.3	< 1	5	7	< 0.01	1	< 0.01	1.09	4	6	< 1	0.02	< 1	< 0.001	< 3	< 5
C-666704 Dup	< 0.3	< 0.01	< 3	< 7	< 1	< 2	12.0	< 0.3	< 1	3	< 1	< 0.01	2	< 0.01	1.10	4	9	< 1	0.02	< 1	< 0.001	< 3	< 5
C-666729 Orig	< 0.3	< 0.01	< 3	9	< 1	< 2	0.03	< 0.3	< 1	2	< 1	< 0.01	< 1	< 0.01	< 0.01	< 1	4	< 1	> 10.0	< 1	< 0.001	< 3	< 5
C-666729 Dup	< 0.3	< 0.01	< 3	8	< 1	< 2	0.03	< 0.3	< 1	3	< 1	< 0.01	< 1	< 0.01	< 0.01	< 1	6	< 1	> 10.0	< 1	< 0.001	< 3	< 5
C-666731 Orig	< 0.3	< 0.01	< 3	8	< 1	< 2	0.43	< 0.3	< 1	8	< 1	< 0.01	< 1	0.01	0.01	< 1	3	< 1	9.25	< 1	< 0.001	32	< 5
C-666731 Dup	< 0.3	< 0.01	< 3	7	< 1	< 2	0.43	< 0.3	< 1	7	< 1	< 0.01	< 1	0.01	0.01	< 1	1	< 1	8.97	< 1	< 0.001	18	< 5
C-666749 Orig	< 0.3	< 0.01	< 3	9	< 1	< 2	0.34	< 0.3	< 1	4	< 1	< 0.01	< 1	< 0.01	0.01	< 1	6	< 1	> 10.0	< 1	< 0.001	< 3	< 5
C-666749 Dup	< 0.3	< 0.01	< 3	9	< 1	< 2	0.40	< 0.3	< 1	3	< 1	< 0.01	< 1	< 0.01	0.01	< 1	6	< 1	> 10.0	< 1	< 0.001	< 3	< 5
C-666682 Orig	< 0.3	< 0.01	< 3	8	< 1	< 2	0.42	< 0.3	< 1	31	< 1	< 0.01	< 1	0.04	< 0.01	< 1	13	< 1	> 10.0	< 1	< 0.001	< 3	< 5
C-666682 Dup	< 0.3	< 0.01	< 3	8	< 1	< 2	0.41	< 0.3	< 1	3	< 1	< 0.01	< 1	0.04	< 0.01	< 1	5	< 1	> 10.0	< 1	< 0.001	< 3	< 5
Method Blank	< 0.3	< 0.01	< 3	< 7	< 1	< 2	< 0.01	< 0.3	< 1	8	< 1	< 0.01	< 1	< 0.01	< 0.01	< 1	9	< 1	< 0.01	< 1	< 0.001	< 3	< 5
Method Blank	< 0.3	< 0.01	< 3	< 7	< 1	< 2	< 0.01	< 0.3	< 1	4	< 1	< 0.01	< 1	< 0.01	< 0.01	< 1	4	< 1	< 0.01	< 1	< 0.001	< 3	< 5
Method Blank	< 0.3	< 0.01	< 3	< 7	< 1	< 2	< 0.01	< 0.3	< 1	2	< 1	< 0.01	< 1	< 0.01	< 0.01	< 1	6	< 1	< 0.01	< 1	< 0.001	< 3	< 5
Method Blank	< 0.3	< 0.01	< 3	< 7	< 1	< 2	< 0.01	< 0.3	< 1	2	< 1	< 0.01	< 1	< 0.01	< 0.01	< 1	4	< 1	< 0.01	< 1	< 0.001	< 3	< 5
Method Blank	< 0.3	< 0.01	< 3	< 7	< 1	< 2	< 0.01	< 0.3	< 1		< 1	< 0.01	< 1	< 0.01	< 0.01	< 1	3	< 1	< 0.01	< 1	< 0.001	< 3	< 5
Method Blank	< 0.3	< 0.01	< 3	< 7	< 1	< 2	< 0.01	< 0.3	< 1	6	< 1	< 0.01	< 1	< 0.01	< 0.01	< 1	6	< 1	< 0.01	< 1	< 0.001	< 3	< 5
Method Blank	< 0.3	< 0.01	< 3	< 7	< 1	< 2	< 0.01	< 0.3	< 1	9	< 1	< 0.01	< 1	< 0.01	< 0.01	< 1	5	< 1	< 0.01	< 1	< 0.001	< 3	< 5
Method Blank	< 0.3	< 0.01	< 3	< 7	< 1	< 2	< 0.01	< 0.3	< 1	3	1	< 0.01	< 1	< 0.01	< 0.01	< 1	4	< 1	< 0.01	< 1	< 0.001	< 3	< 5
Method Blank	< 0.3	< 0.01	< 3	< 7	< 1	< 2	< 0.01	< 0.3	< 1	7	< 1	< 0.01	< 1	< 0.01	< 0.01	< 1	6	< 1	< 0.01	< 1	< 0.001	< 3	< 5
Method Blank	< 0.3	< 0.01	< 3	< 7	< 1	< 2	< 0.01	< 0.3	< 1	4	< 1	< 0.01	< 1	< 0.01	< 0.01	< 1	4	< 1	< 0.01	< 1	< 0.001	< 3	< 5
Method Blank	< 0.3	< 0.01	< 3	< 7	< 1	< 2	< 0.01	< 0.3	< 1		< 1	< 0.01	< 1	< 0.01	< 0.01	< 1	4	< 1	< 0.01	< 1	< 0.001	< 3	< 5
Method Blank	< 0.3	< 0.01	< 3	< 7	< 1	< 2	< 0.01	< 0.3	< 1	10	< 1	< 0.01	< 1	< 0.01	< 0.01	< 1	6	< 1	< 0.01	< 1	< 0.001	< 3	< 5

Analyte Symbol	Ag	Al	As	Ba	Be	Bi	Ca	Cd	Co	Cr	Cu	Fe	Ga	K	Mg	Li	Mn	Mo	Na	Ni	P	Pb	Sb
Unit Symbol	ppm	%	ppm	ppm	ppm	ppm	%	ppm	ppm	ppm	ppm	%	ppm	%	%	ppm	ppm	ppm	%	ppm	%	ppm	ppm
Lower Limit	0.3	0.01	3	7	1	2	0.01	0.3	1	1	1	0.01	1	0.01	0.01	1	1	1	0.01	1	0.001	3	5
Method Code	TD-ICP	TD-ICP	TD-ICP	TD-ICP	TD-ICP	TD-ICP	TD-ICP	TD-ICP	TD-ICP	TD-ICP	TD-ICP	TD-ICP	TD-ICP	TD-ICP	TD-ICP	TD-ICP	TD-ICP	TD-ICP	TD-ICP	TD-ICP	TD-ICP	TD-ICP	TD-ICP
Method Blank	< 0.3	< 0.01	< 3	< 7	< 1	< 2	< 0.01	< 0.3	< 1	3	< 1	< 0.01	< 1	< 0.01	< 0.01	< 1	3	< 1	< 0.01	< 1	< 0.001	< 3	< 5
Method Blank	< 0.3	< 0.01	< 3	< 7	< 1	< 2	< 0.01	< 0.3	< 1	1	< 1	< 0.01	< 1	< 0.01	< 0.01	< 1	2	< 1	< 0.01	< 1	< 0.001	< 3	< 5
Method Blank	< 0.3	< 0.01	< 3	< 7	< 1	< 2	< 0.01	< 0.3	< 1		< 1	< 0.01	< 1	< 0.01	< 0.01	< 1	6	< 1	< 0.01	< 1	< 0.001	< 3	< 5
Method Blank	< 0.3	< 0.01	< 3	< 7	< 1	< 2	< 0.01	< 0.3	< 1	4	5	< 0.01	< 1	< 0.01	< 0.01	< 1	6	< 1	< 0.01	< 1	< 0.001	< 3	< 5

Analyte Symbol	S	Sc	Sr	Te	Ti	Tl	U	V	W	Y	Zn	Zr
Unit Symbol	%	ppm	ppm	ppm	%	ppm	ppm	ppm	ppm	ppm	ppm	ppm
Lower Limit	0.01	4	1	2	0.01	5	10	2	5	1	1	5
Method Code	TD-ICP	TD-ICP	TD-ICP	TD-ICP	TD-ICP	TD-ICP	TD-ICP	TD-ICP	TD-ICP	TD-ICP	TD-ICP	TD-ICP
OREAS 101b (4 Acid) Meas					0.35		380	77		122		
OREAS 101b (4 Acid) Cert					0.35		387	77		133		
OREAS 101b (4 Acid) Meas					0.36		390	79		127		
OREAS 101b (4 Acid) Cert					0.35		387	77		133		
OREAS 101b (4 Acid) Meas					0.37		390	79		129		
OREAS 101b (4 Acid) Cert					0.35		387	77		133		
OREAS 98 (4 Acid) Meas											1280	
OREAS 98 (4 Acid) Cert											1360	
OREAS 98 (4 Acid) Meas											1320	
OREAS 98 (4 Acid) Cert											1360	
OREAS 903 (4 Acid) Meas	0.48	11	84		0.31	< 5	< 10	82		23	26	19
OREAS 903 (4 Acid) Cert	0.500	10.2	77.0		0.192	0.620	7.58	74.0		22.5	24.3	152
OREAS 903 (4 Acid) Meas	0.51	12	87		0.31	< 5	< 10	86		23	27	60
OREAS 903 (4 Acid) Cert	0.500	10.2	77.0		0.192	0.620	7.58	74.0		22.5	24.3	152
OREAS 903 (4 Acid) Meas	0.51	11	95		0.31	< 5	< 10	88		23	30	167
OREAS 903 (4 Acid) Cert	0.500	10.2	77.0		0.192	0.620	7.58	74.0		22.5	24.3	152
OREAS 96 (4 Acid) Meas	4.32										444	
OREAS 96 (4 Acid) Cert	4.19										457	
OREAS 96 (4 Acid) Meas	4.30										445	
OREAS 96 (4 Acid) Cert	4.19										457	
OREAS 96 (4 Acid) Meas	4.22										463	
OREAS 96 (4 Acid) Cert	4.19										457	
OREAS 96 (4 Acid) Meas	4.18										461	
OREAS 96 (4 Acid) Cert	4.19										457	
OREAS 520 (4 Acid) Meas	0.92	17	90	< 2	0.49	< 5	20	260	43	18	22	128
OREAS 520 (4 Acid) Cert	1.01	17.0	104	0.360	0.445	0.260	17.9	257	43.8	20.8	22.7	134
OREAS 520 (4 Acid) Meas	0.92	17	92	11	0.45	< 5	10	252	21	19	24	129
OREAS 520 (4 Acid) Cert	1.01	17.0	104	0.360	0.445	0.260	17.9	257	43.8	20.8	22.7	134
Oreas 72b (4 Acid) Meas	1.41	12	62	2	0.20	< 5	< 10	70	7	11	86	79

Analyte Symbol	S	Sc	Sr	Te	Ti	Tl	U	V	W	Y	Zn	Zr
Unit Symbol	%	ppm	ppm	ppm	%	ppm	ppm	ppm	ppm	ppm	ppm	ppm
Lower Limit	0.01	4	1	2	0.01	5	10	2	5	1	1	5
Method Code	TD-ICP	TD-ICP	TD-ICP	TD-ICP	TD-ICP	TD-ICP	TD-ICP	TD-ICP	TD-ICP	TD-ICP	TD-ICP	TD-ICP
Oreas 72b (4 Acid) Cert	1.49	12.8	63.8	0.0920	0.216	0.350	4.68	73.6	4.00	12.8	99.0	88.0
Oreas 72b (4 Acid) Meas	1.43	12	63	4	0.21	< 5	< 10	72	8	12	89	80
Oreas 72b (4 Acid) Cert	1.49	12.8	63.8	0.0920	0.216	0.350	4.68	73.6	4.00	12.8	99.0	88.0
Oreas 72b (4 Acid) Meas	1.39	12	63	2	0.20	< 5	< 10	71	< 5	11	92	78
Oreas 72b (4 Acid) Cert	1.49	12.8	63.8	0.0920	0.216	0.350	4.68	73.6	4.00	12.8	99.0	88.0
OREAS 45f (4-Acid) Meas	0.02	39	29		0.44	< 5	< 10	138	< 5	11	40	105
OREAS 45f (4-Acid) Cert	0.0290	36.3	25.1		1.08	0.200	2.09	253	1.27	10.9	35.3	172
OREAS 70b (Fusion) Meas	0.29		72		0.17		< 10	65		8	100	63
OREAS 70b (Fusion) Cert	0.308		72		0.176		1.87	69		10.7	107	67
OREAS 683 (4 Acid) Meas	0.19	19	260		0.25		< 10	174	< 5	7	76	
OREAS 683 (4 Acid) Cert	0.205	19.7	276		0.263		0.58	187	1.23	8.02	92	
OREAS 683 (4 Acid) Meas	0.19	19	261		0.25		< 10	173	< 5	7	76	
OREAS 683 (4 Acid) Cert	0.205	19.7	276		0.263		0.58	187	1.23	8.02	92	
OREAS 683 (4 Acid) Meas	0.19	19	254		0.25		< 10	168	< 5	7	78	
OREAS 683 (4 Acid) Cert	0.205	19.7	276		0.263		0.58	187	1.23	8.02	92	
OREAS 681 (4 Acid) Meas	0.10	27	455		0.56		< 10	238	< 5	15	75	61
OREAS 681 (4 Acid) Cert	0.109	27.7	478		0.588		1.44	253	1.09	17.5	88.0	58.0
OREAS 681 (4 Acid) Meas	0.10	27	462		0.56		< 10	242	< 5	16	78	62
OREAS 681 (4 Acid) Cert	0.109	27.7	478		0.588		1.44	253	1.09	17.5	88.0	58.0
OREAS 681 (4 Acid) Meas	0.10	26	467		0.55		< 10	239	< 5	15	82	62
OREAS 681 (4 Acid) Cert	0.109	27.7	478		0.588		1.44	253	1.09	17.5	88.0	58.0
OREAS 70b (4 Acid) Meas	0.29	12	74		0.17	< 5	< 10	64	7	8	95	61
OREAS 70b (4 Acid) Cert	0.31	12	74		0.18	0.3	2	67	5	10	110	66
OREAS 70b (4 Acid) Meas	0.29	12	73		0.17	< 5	< 10	64	5	8	102	64
OREAS 70b (4 Acid) Cert	0.31	12	74		0.18	0.3	2	67	5	10	112	66
OREAS 620 (4 Acid) Meas	2.52	6	124		0.16	< 5	< 10	23	< 5	13	> 10000	203
OREAS 620 (4 Acid) Cert	2.47	5	131		0.14	2	4	21	2	12	31500	202
OREAS 620 (4 Acid) Meas	2.52	5	118		0.16	< 5	< 10	23	< 5	13	> 10000	202
OREAS 620 (4 Acid) Cert	2.47	5	131		0.14	2	4	21	2	12	31500	202

Analyte Symbol	S	Sc	Sr	Te	Ti	Tl	U	V	W	Y	Zn	Zr
Unit Symbol	%	ppm	ppm	ppm	%	ppm	ppm	ppm	ppm	ppm	ppm	ppm
Lower Limit	0.01	4	1	2	0.01	5	10	2	5	1	1	5
Method Code	TD-ICP	TD-ICP	TD-ICP	TD-ICP	TD-ICP	TD-ICP	TD-ICP	TD-ICP	TD-ICP	TD-ICP	TD-ICP	TD-ICP
OREAS 620 (4 Acid) Meas	2.51	5	122		0.16	< 5	< 10	23	< 5	13	> 10000	204
OREAS 620 (4 Acid) Cert	2.47	5	131		0.14	2	4	21	2	12	31500	202
OREAS 620 (4 Acid) Meas	2.63	5	117		0.15	< 5	< 10	24	< 5	12	> 10000	193
OREAS 620 (4 Acid) Cert	2.47	5	131		0.14	2	4	21	2	12	31500	202
OREAS 753 (4 Acid) Meas	0.01	< 4	31		< 0.01	< 5	< 10	< 2	7	< 1	95	12
OREAS 753 (4 Acid) Cert	0.014	0.10	25.5		0.004	3.67	5.83	1.16	5.62	0.65	87	11.4
OREAS 753 (4 Acid) Meas	0.01	< 4	31		< 0.01	< 5	< 10	< 2	8	< 1	96	11
OREAS 753 (4 Acid) Cert	0.014	0.10	25.5		0.004	3.67	5.83	1.16	5.62	0.65	87	11.4
OREAS 753 (4 Acid) Meas	0.01	< 4	29		< 0.01	< 5	10	2	7	< 1	96	11
OREAS 753 (4 Acid) Cert	0.014	0.10	25.5		0.004	3.67	5.83	1.16	5.62	0.65	87	11.4
OREAS 603c (4 Acid) Meas	3.75	5	190	9	0.15	< 5	< 10	20	9	12	8480	176
OREAS 603c (4 Acid) Cert	4	4.14	270	23.8	0.142	4.18	4.42	18.6	8.99	11.6	8030	177
OREAS 603c (4 Acid) Meas	3.71	5	172	11	0.15	< 5	< 10	21	7	12	8450	179
OREAS 603c (4 Acid) Cert	4	4.14	270	23.8	0.142	4.18	4.42	18.6	8.99	11.6	8030	177
OREAS 601c (4 acid) Meas	1.64	< 4	223	4	0.15	< 5	< 10	17	9	11	451	184
OREAS 601c (4 acid) Cert	1.58	4.01	230	7.50	0.135	1.75	4.40	15.5	4.67	11.5	425	178
OREAS 601c (4 acid) Meas	1.62	4	228	2	0.15	< 5	< 10	17	9	11	440	179
OREAS 601c (4 acid) Cert	1.58	4.01	230	7.50	0.135	1.75	4.40	15.5	4.67	11.5	425	178
OREAS 601c (4 acid) Meas	1.60	4	218	3	0.14	< 5	< 10	17	9	11	450	179
OREAS 601c (4 acid) Cert	1.58	4.01	230	7.50	0.135	1.75	4.40	15.5	4.67	11.5	425	178
OREAS 601c (4 acid) Meas	1.59	< 4	219	3	0.15	< 5	< 10	17	9	11	443	179
OREAS 601c (4 acid) Cert	1.58	4.01	230	7.50	0.135	1.75	4.40	15.5	4.67	11.5	425	178
OREAS 504 (4 Acid) Meas	1.33											
OREAS 504 (4 Acid) Cert	1.37											
OREAS 504 (4 Acid) Meas	1.34											
OREAS 504 (4 Acid) Cert	1.37											
OREAS 504 (4 Acid) Meas	1.36											
OREAS 504 (4 Acid) Cert	1.37											
OREAS 504 (4 Acid) Meas	1.32											

Analyte Symbol	S	Sc	Sr	Te	Ti	Tl	U	V	W	Y	Zn	Zr
Unit Symbol	%	ppm	ppm	ppm	%	ppm	ppm	ppm	ppm	ppm	ppm	ppm
Lower Limit	0.01	4	1	2	0.01	5	10	2	5	1	1	5
Method Code	TD-ICP	TD-ICP	TD-ICP	TD-ICP	TD-ICP	TD-ICP	TD-ICP	TD-ICP	TD-ICP	TD-ICP	TD-ICP	TD-ICP
OREAS 504 (4 Acid) Cert	1.37											
GMO-04 Meas	0.66								< 5		123	
GMO-04 Cert	0.71								2.60		128.0	
GMO-04 Meas	0.65								6		127	
GMO-04 Cert	0.71								2.60		128.0	
GMO-04 Meas	0.63								< 5		131	
GMO-04 Cert	0.71								2.60		128.0	
OREAS 45h (4-Acid) Meas	0.03	60	30		0.64	< 5	< 10	206	< 5	10	40	115
OREAS 45h (4-Acid) Cert	0.035	57	27.1		0.878	0.15	1.68	263	0.99	10.40	39.7	131
C-666581 Orig	0.31	< 4	128	< 2	0.03	< 5	30	9	< 5	4	8	6
C-666581 Dup	0.30	< 4	127	< 2	0.03	< 5	30	9	< 5	4	8	6
C-666591 Orig	0.25	< 4	93	< 2	0.03	< 5	90	9	< 5	2	7	8
C-666591 Dup	0.25	< 4	94	< 2	0.03	< 5	80	10	< 5	2	8	8
C-666601 Orig	0.53	< 4	109	< 2	0.08	< 5	80	18	< 5	4	12	18
C-666601 Dup	0.53	< 4	111	< 2	0.08	< 5	60	19	< 5	4	13	19
C-666614 Orig	0.08	< 4	12	< 2	< 0.01	< 5	90	2	< 5	< 1	2	< 5
C-666614 Dup	0.08	< 4	12	< 2	< 0.01	< 5	90	< 2	< 5	< 1	2	< 5
C-666632 Orig	0.98	< 4	54	< 2	0.01	< 5	90	3	< 5	< 1	3	< 5
C-666632 Dup	0.95	< 4	52	< 2	0.01	< 5	100	3	< 5	< 1	2	< 5
C-666644 Orig	0.10	< 4	11	< 2	< 0.01	< 5	80	< 2	< 5	< 1	< 1	< 5
C-666644 Dup	0.12	< 4	12	< 2	< 0.01	< 5	80	< 2	< 5	< 1	< 1	< 5
C-666659 Orig	0.33	< 4	11	< 2	< 0.01	< 5	100	< 2	< 5	< 1	< 1	< 5
C-666659 Dup	0.34	< 4	11	< 2	< 0.01	< 5	90	< 2	< 5	< 1	< 1	< 5
C-666671 Orig	9.99	< 4	761	< 2	0.06	< 5	< 10	13	< 5	1	9	8
C-666671 Dup	10.7	< 4	769	< 2	0.06	< 5	< 10	13	< 5	1	9	9
C-666688 Orig	0.13	< 4	33	< 2	0.03	< 5	80	7	< 5	2	5	9
C-666688 Dup	0.13	< 4	34	< 2	0.03	< 5	80	7	< 5	2	6	8
C-666704 Orig	11.0	< 4	880	< 2	< 0.01	< 5	< 10	< 2	< 5	< 1	< 1	< 5
C-666704 Dup	9.71	< 4	846	< 2	< 0.01	< 5	< 10	< 2	< 5	< 1	< 1	< 5
C-666729 Orig	0.02	< 4	1	< 2	< 0.01	< 5	100	< 2	< 5	< 1	< 1	< 5
C-666729 Dup	0.02	< 4	1	< 2	< 0.01	< 5	90	< 2	< 5	< 1	< 1	< 5
C-666731 Orig	0.32	< 4	15	< 2	< 0.01	< 5	< 10	< 2	< 5	< 1	< 1	< 5
C-666731 Dup	0.33	< 4	15	< 2	< 0.01	< 5	< 10	< 2	< 5	< 1	< 1	< 5
C-666749 Orig	0.25	< 4	17	< 2	< 0.01	< 5	100	< 2	< 5	< 1	< 1	< 5
C-666749 Dup	0.30	< 4	19	< 2	< 0.01	< 5	90	< 2	< 5	< 1	4	< 5
C-666682 Orig	0.32	< 4	17	< 2	< 0.01	< 5	90	< 2	< 5	< 1	2	< 5
C-666682 Dup	0.32	< 4	16	< 2	< 0.01	< 5	90	< 2	< 5	< 1	2	< 5
Method Blank	< 0.01	< 4	< 1	< 2	< 0.01	< 5	< 10	< 2	< 5	< 1	< 1	< 5
Method Blank	< 0.01	< 4	< 1	< 2	< 0.01	< 5	< 10	< 2	< 5	< 1	< 1	< 5
Method Blank	< 0.01	< 4	< 1	< 2	< 0.01	< 5	< 10	< 2	< 5	< 1	< 1	< 5
Method Blank	< 0.01	< 4	< 1	3	< 0.01	< 5	< 10	< 2	< 5	< 1	< 1	< 5
Method Blank	< 0.01	< 4	< 1	< 2	< 0.01	< 5	< 10	< 2	< 5	< 1	< 1	< 5
Method Blank	< 0.01	< 4	< 1	< 2	< 0.01	< 5	< 10	< 2	< 5	< 1	< 1	< 5
Method Blank	< 0.01	< 4	< 1	< 2	< 0.01	< 5	< 10	< 2	< 5	< 1	< 1	< 5
Method Blank	< 0.01	< 4	< 1	< 2	< 0.01	< 5	< 10	< 2	< 5	< 1	< 1	< 5
Method Blank	< 0.01	< 4	< 1	< 2	< 0.01	< 5	< 10	< 2	< 5	< 1	< 1	< 5
Method Blank	< 0.01	< 4	< 1	< 2	< 0.01	< 5	< 10	< 2	< 5	< 1	< 1	< 5
Method Blank	< 0.01	< 4	< 1	< 2	< 0.01	< 5	< 10	< 2	< 5	< 1	< 1	< 5
Method Blank	< 0.01	< 4	< 1	< 2	< 0.01	< 5	< 10	< 2	< 5	< 1	1	< 5

Analyte Symbol	S	Sc	Sr	Te	Ti	Tl	U	V	W	Y	Zn	Zr
Unit Symbol	%	ppm	ppm	ppm	%	ppm	ppm	ppm	ppm	ppm	ppm	ppm
Lower Limit	0.01	4	1	2	0.01	5	10	2	5	1	1	5
Method Code	TD-ICP	TD-ICP	TD-ICP	TD-ICP	TD-ICP	TD-ICP	TD-ICP	TD-ICP	TD-ICP	TD-ICP	TD-ICP	TD-ICP
Method Blank	< 0.01	< 4	< 1	< 2	< 0.01	< 5	< 10	< 2	< 5	< 1	< 1	< 5
Method Blank	< 0.01	< 4	< 1	< 2	< 0.01	< 5	< 10	< 2	< 5	< 1	< 1	< 5
Method Blank	< 0.01	< 4	< 1	< 2	< 0.01	< 5	< 10	< 2	< 5	< 1	< 1	< 5
Method Blank	< 0.01	< 4	< 1	< 2	< 0.01	< 5	< 10	< 2	< 5	< 1	< 1	< 5
