# Supplementary material for: Geochemistry and X-ray diffraction data from rock salts and saltwork wastes of Canada: data compilation
Source: Data Brief. 2026 Jun 6;67:112941. doi: 10.1016/j.dib.2026.112941 (PMC13292661; doi:10.1016/j.dib.2026.112941)

X-ray Diffraction Analysis of One Hundred Samples

W.O. # A25-01103
Invoice # A25-01103

Client: GSC/NRCan

Attn: Pavel Kabanov

Date Reported: March 26, 2025

Activation Laboratories Ltd.
41 Bittern Street, Ancaster, ON L9G 4V5, Tel: +1.905.648.9611, Fax: +1.905.648.96123

Method

One hundred samples were submitted for semi-quantitative X-ray diffraction analysis. A portion of each pulverized sample was loaded into a standard holder. The X-ray diffraction analysis was performed on a Bruker D8 Endeavour diffractometer equipped with Cu X-ray source and operating at the following conditions: 40 kV and 40 mA; range 4 - 70 deg 2θ; step size 0.02 deg 2θ; time per step 0.5 sec; fixed divergence slit, angle 0.3^0; sample rotation 15 rpm. The PDF4/Minerals ICDD database was used for mineral identification. The quantities of the crystalline mineral phases were determined using Rietveld method. The Rietveld method is based on the calculation of the full diffraction pattern from crystal structure data.

Results

The minerals identified in the samples and their abundances are in Table 1 and the diffraction patterns are in Appendix 1.

Table 1. Mineral abundances (wt %)

Client ID	Actlabs ID	Halite	Sylvite	Anhydrite	Gypsum	Quartz	Muscovite	Chlorite	K feldspar	Dolomite
KH-499.20	A25-01103-1	95.9	1.1	1.5	n.d.	0.6	trace	trace	n.d.	0.9
KH-502.56	A25-01103-2	96.2	1.1	1.1	n.d.	1.3	trace	trace	n.d.	0.3
KH-509.63	A25-01103-3	98.1	1.2	0.6	n.d.	0.1	n.d.	n.d.	n.d.	n.d.
KH-522.95	A25-01103-4	98.4	1.2	0.3	n.d.	0.1	n.d.	n.d.	n.d.	n.d.
KH-530.76	A25-01103-5	97.9	1.1	0.6	n.d.	0.1	n.d.	n.d.	n.d.	0.3
KH-540.02	A25-01103-6	95.4	1.0	1.2	n.d.	1.7	trace	trace	n.d.	0.7
KH-549.09	A25-01103-7	87.9	1.0	3.1	n.d.	5.8	1.0	0.4	n.d.	0.8
KH-556.15	A25-01103-8	94.9	1.1	2.5	n.d.	1.5	trace	n.d.	n.d.	n.d.
KH-564.11	A25-01103-9	93.9	1.0	3.0	n.d.	1.1	trace	trace	n.d.	1.0
KH-573.38	A25-01103-10	80.6	1.0	4.6	trace	9.8	3.0	0.7	n.d.	0.3
KH-581.28	A25-01103-11	98.0	1.2	0.5	n.d.	trace	n.d.	n.d.	n.d.	0.3
KH-592.8	A25-01103-12	89.4	7.0	2.3	n.d.	0.2	n.d.	n.d.	n.d.	1.1
KH-603.42	A25-01103-13	92.9	1.1	4.1	trace	0.9	trace	trace	n.d.	1.0
KH-613.56	A25-01103-14	96.4	2.6	0.3	trace	0.7	trace	n.d.	n.d.	n.d.
KH-622.96	A25-01103-15	96.9	1.2	1.0	trace	0.6	trace	trace	n.d.	0.3
KH-636.28	A25-01103-16	97.1	1.1	0.6	n.d.	0.5	trace	trace	n.d.	0.7
KH-645.57	A25-01103-17	92.6	1.0	2.8	n.d.	1.6	0.4	0.3	n.d.	1.3
KH-655.09	A25-01103-18	96.9	1.1	0.6	n.d.	0.7	trace	trace	n.d.	0.7
KH-664.44**	A25-01103-19	96.9	1.1	1.4	n.d.	0.1	n.d.	n.d.	n.d.	0.5
KH-674.32	A25-01103-20	96.0	1.0	2.3	n.d.	0.1	n.d.	n.d.	n.d.	0.6
KH-682.03	A25-01103-21	97.9	1.2	0.4	n.d.	n.d.	n.d.	n.d.	n.d.	0.5
KIH-691.09	A25-01103-22	97.1	1.0	1.5	n.d.	n.d.	n.d.	n.d.	n.d.	0.4
KH-700.13	A25-01103-23	96.7	1.0	1.9	n.d.	n.d.	n.d.	n.d.	n.d.	0.4
KH-704.23	A25-01103-24	96.8	1.0	1.9	n.d.	n.d.	n.d.	n.d.	n.d.	0.3
KH-711.55	A25-01103-25	98.1	1.3	0.2	trace	n.d.	n.d.	n.d.	n.d.	0.4
KH-719.22	A25-01103-26	96.4	1.2	1.2	n.d.	0.4	n.d.	n.d.	n.d.	0.8
KH-726.7	A25-01103-27	97.0	1.2	1.5	n.d.	trace	n.d.	n.d.	n.d.	0.3

Client ID	Actlabs ID	Halite	Sylvite	Anhydrite	Gypsum	Quartz	Muscovite	Chlorite	K feldspar	Dolomite
KH-734.42	A25-01103-28	89.8	1.1	8.5	trace	0.3	trace	n.d.	n.d.	0.3
KH-741.84	A25-01103-29	97.5	1.3	0.3	n.d.	n.d.	n.d.	n.d.	n.d.	0.9
KH-749.3	A25-01103-30	93.6	1.0	1.4	0.3	1.8	1.0	0.3	n.d.	0.6
KH-756.79	A25-01103-31	94.8	1.3	3.3	0.3	trace	n.d.	n.d.	n.d.	0.3
KH-763.38	A25-01103-32	97.5	1.2	0.6	n.d.	0.4	n.d.	n.d.	n.d.	0.3
KH-771.73	A25-01103-33	85.8	0.6	13.1	n.d.	n.d.	n.d.	n.d.	n.d.	0.5
KH-779.39	A25-01103-34	97.8	1.3	0.9	n.d.	trace	n.d.	n.d.	n.d.	n.d.
KH-786.79	A25-01103-35	97.2	1.0	1.3	n.d.	n.d.	n.d.	n.d.	n.d.	0.5
KH-794.29	A25-01103-36	95.2	1.0	3.4	n.d.	0.4	trace	n.d.	n.d.	n.d.
KH-801.64	A25-01103-37	91.3	1.1	7.1	n.d.	trace	n.d.	n.d.	n.d.	0.5
KH-809.21	A25-01103-38	63.3	0.8	34.9	n.d.	0.6	trace	n.d.	n.d.	0.4
KH-816.95	A25-01103-39	97.0	1.1	1.8	n.d.	0.1	n.d.	n.d.	n.d.	n.d.
KH-824.29	A25-01103-40	97.0	1.5	1.4	n.d.	0.1	n.d.	n.d.	n.d.	n.d.
KH-832.84	A25-01103-41	90.0.	0.6	9.3	n.d.	0.1	trace	n.d.	n.d.	n.d.
KH-828.16	A25-01103-42	88.7	1.0	10.0	n.d.	0.3	n.d.	n.d.	n.d.	n.d.
SB-1-1922.0	A25-01103-43	96.6	1.1	1.1	0.8	0.1	n.d.	n.d.	n.d.	0.3
SB-1-1940.0	A25-01103-44	95.1	1.2	2.2	0.9	n.d.	n.d.	n.d.	n.d.	0.6
SB-1-1960.0	A25-01103-45	95.3	1.5	1.2	1.4	0.1	n.d.	n.d.	n.d.	0.5
SB-1-1983.5	A25-01103-46	84.0	0.8	8.7	6.2	n.d.	n.d.	n.d.	n.d.	0.3
SB-1-2000.0	A25-01103-47	96.7	1.1	1.9	trace	trace	n.d.	n.d.	n.d.	0.3
SB-1-2020	A25-01103-48	97.8	1.1	0.4	n.d.	n.d.	n.d.	n.d.	n.d.	0.7
SB-1-2040	A25-01103-49	97.8	1.4	0.6	trace	0.2	n.d.	n.d.	n.d.	n.d.
SB-1-2060	A25-01103-50	64.4	n.d.	35.6	trace	n.d.	n.d.	n.d.	n.d.	n.d.
SB-1-2080	A25-01103-51	95.0	0.9	3.7	trace	0.1	n.d.	n.d.	n.d.	0.3
SB-1-2100.0	A25-01103-52	97.5	1.3	1.2	trace	n.d.	n.d.	n.d.	n.d.	n.d.
SB-1-2120.0	A25-01103-53	97.1	1.0	1.9	trace	trace	n.d.	n.d.	n.d.	n.d.
SB-1-2140.0	A25-01103-54	97.7	1.4	0.6	trace	trace	n.d.	n.d.	n.d.	0.3
SB-1-2160.0	A25-01103-55	81.2	0.8	17.9	trace	0.1	n.d.	n.d.	n.d.	n.d.
SB-1-2180.0	A25-01103-56	85.7	1.2	7.0	trace	3.1	2.0	1.0	n.d.	n.d.

Client ID	Actlabs ID	Halite	Sylvite	Anhydrite	Gypsum	Quartz	Muscovite	Chlorite	K feldspar	Dolomite
SB-1-2200.0	A25-01103-57	98.2	1.1	0.4	n.d.	n.d.	n.d.	n.d.	n.d.	0.3
SB-1-2221.0	A25-01103-58	94.0	1.0	3.7	1.3	trace	n.d.	n.d.	n.d.	n.d.
SB-1-2240.0	A25-01103-59	96.3	0.9	2.4	trace	n.d.	trace	n.d.	n.d.	0.4
SB-1-2258.4	A25-01103-60	83.4	0.7	11.3	4.2	0.1	n.d.	n.d.	n.d.	0.3
SB-1-2280.0	A25-01103-61	92.0	1.0	6.3	trace	n.d.	n.d.	n.d.	n.d.	0.7
SB-1-2300.0	A25-01103-62	94.6	1.1	3.8	trace	n.d.	n.d.	n.d.	n.d.	0.5
SB-1-2320.0	A25-01103-63	89.9	0.9	8.7	trace	n.d.	n.d.	n.d.	n.d.	0.5
SB-1-2340.0	A25-01103-64	89.0	1.0	8.9	0.6	n.d.	n.d.	n.d.	n.d.	0.5
SB-1-2360.0	A25-01103-65	93.0	1.0	5.7	n.d.	trace	n.d.	n.d.	n.d.	0.3
SB-1-2380.0	A25-01103-66	92.0	1.0	7.0	trace	trace	n.d.	n.d.	n.d.	n.d.
SB-1-2400.0	A25-01103-67	97.2	1.2	1.6	n.d.	trace	n.d.	n.d.	n.d.	n.d.
SB-1-2420.0	A25-01103-68	90.6	0.9	7.3	0.5	n.d.	n.d.	n.d.	n.d.	0.7
SB-1-2430.0	A25-01103-69	92.0	0.8	6.6	0.3	n.d.	n.d.	n.d.	n.d.	0.3
SB-1-2440.0	A25-01103-70	87.9	0.6	10.9	0.3	trace	n.d.	n.d.	n.d.	0.3
SB-1-2450.0	A25-01103-71	91.7	0.7	7.3	trace	trace	n.d.	n.d.	n.d.	0.3
SB-1-2460.0	A25-01103-72	93.2	0.8	5.9	n.d.	0.1	n.d.	n.d.	n.d.	n.d.
SB-1-2470.1	A25-01103-73	91.0	0.9	7.8	trace	n.d.	n.d.	n.d.	n.d.	0.3
SB-1-2480.1	A25-01103-74	90.7	0.8	8.2	trace	trace	n.d.	n.d.	n.d.	0.3
SB-1-2485.1	A25-01103-75	88.5	0.5	11	n.d.	trace	n.d.	n.d.	n.d.	n.d.
SB-1-2490.1	A25-01103-76	92.5	1.2	5.8	trace	0.5	trace	n.d.	n.d.	n.d.
CM2-447.07	A25-01103-77	82.2	0.8	16.7	n.d.	n.d.	n.d.	n.d.	n.d.	0.3
CM2-452.00	A25-01103-78	97.0	1.1	1.6	n.d.	n.d.	n.d.	n.d.	n.d.	0.3
CM2-458.06	A25-01103-79	97.4	1.2	0.8	trace	trace	n.d.	n.d.	n.d.	0.6
CM2-460.27	A25-01103-80	95.1	3.6	0.9	n.d.	trace	n.d.	n.d.	n.d.	0.4
CM2-462.11	A25-01103-81	96.5	2.8	n.d.	n.d.	trace	n.d.	n.d.	n.d.	0.7
CM2-464.30	A25-01103-82	31.2	66.2	1.5	n.d.	1.1	trace	trace	n.d.	n.d.
CM2-465.86	A25-01103-83	69.5	27.7	2.4	n.d.	0.4	trace	n.d.	n.d.	n.d.
CM2-466.23	A25-01103-84	61.0	36.6	1.8	n.d.	0.3	n.d.	n.d.	n.d.	0.3
CM2-469.20	A25-01103-85	61.4	35.9	2.7	n.d.	trace	n.d.	n.d.	n.d.	n.d.

Client ID	Actlabs ID	Halite	Sylvite	Anhydrite	Gypsum	Quartz	Muscovite	Chlorite	K feldspar	Dolomite
CM2-473.02	A25-01103-86	97.2	1.8	1.0	trace	trace	n.d.	n.d.	n.d.	n.d.
CM2-480.49	A25-01103-87	52.0	47.3	0.3	trace	n.d.	n.d.	n.d.	n.d.	0.4
CM2-485.08	A25-01103-88	79.1	17.0	3.4	trace	n.d.	n.d.	n.d.	n.d.	0.5
CM2-490.52	A25-01103-89	64.6	29.6	5.5	n.d.	0.3	n.d.	n.d.	n.d.	n.d.
CM2-497.00	A25-01103-90	97.2	2.4	0.4	trace	n.d.	n.d.	n.d.	n.d.	n.d.
CM2-500.92	A25-01103-91	82.5	12.5	4.3	trace	0.7	trace	trace	n.d.	n.d.
CM2-507.38	A25-01103-92	83.8	15.6	0.6	n.d.	n.d.	n.d.	n.d.	n.d.	n.d.
CM2-510.96	A25-01103-93	94.2	5.0	n.d.	n.d.	trace	n.d.	n.d.	n.d.	0.8
CM2-513.32	A25-01103-94	64.8	33.0	1.9	n.d.	n.d.	n.d.	n.d.	n.d.	0.3
CM2-515.00	A25-01103-95	61.3	37.0	1.0	0.3	0.4	trace	n.d.	n.d.	n.d.
CM2-515.95	A25-01103-96	57.6	40.6	0.6	n.d.	0.9	trace	n.d.	n.d.	0.3
CM2-518.23	A25-01103-97	45.9	52.5	1.5	n.d.	0.1	n.d.	n.d.	n.d.	n.d.
CM2-523.48	A25-01103-98	28.2	32.4	n.d.	trace	8.7	9.0	n.d.	21.7	n.d.
CM2-526.98	A25-01103-99	91.2	7.3	1.1	n.d.	0.4	n.d.	n.d.	n.d.	n.d.
CM2-529.96	A25-01103-100	95.6	3.4	0.4	n.d.	0.6	trace	n.d.	n.d.	n.d.

Note: n.d. = not detected

Reported by:
Elitsa Hrischeva, PhD
Activation Laboratories Ltd.

APPENDIX 1

Diffraction Patterns

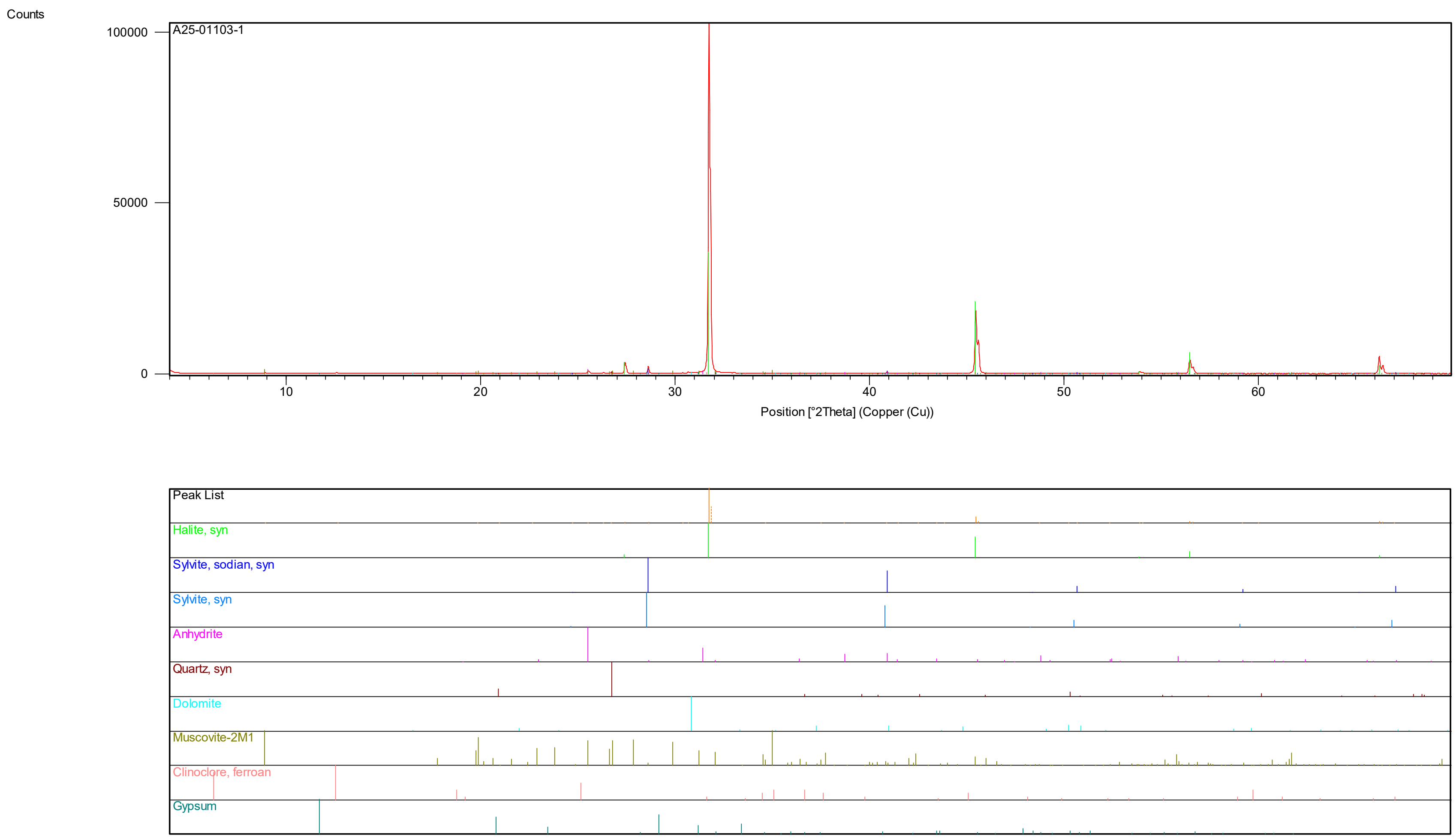
Counts
100000
50000
0
A25-01103-1
10
20
30
40
50
60
Position [°2Theta] (Copper (Cu))
Peak List
Halite, syn
Sylvite, sodian, syn
Sylvite, syn
Anhydrite
Quartz, syn
Dolomite
Muscovite-2M1
Clinoclore, ferroan
Gypsum

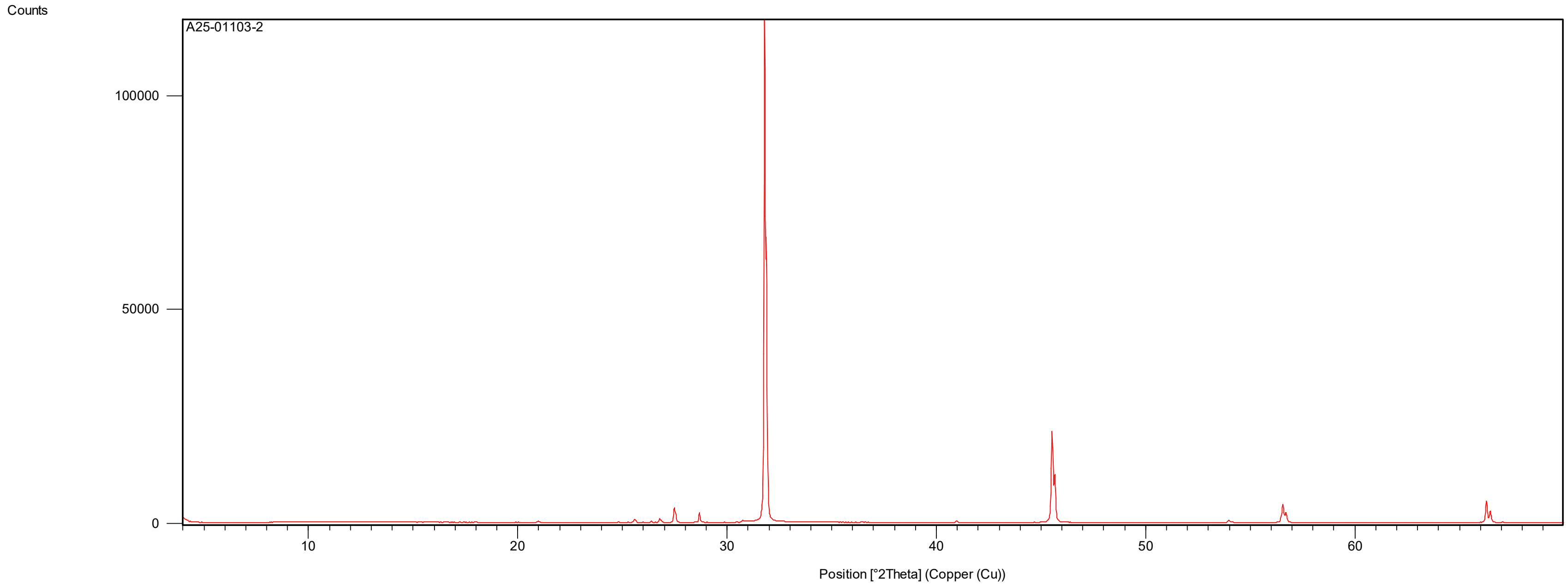
Counts
A25-01103-2
100000
50000
0
10
20
30
40
50
60
Position [°2Theta] (Copper (Cu))

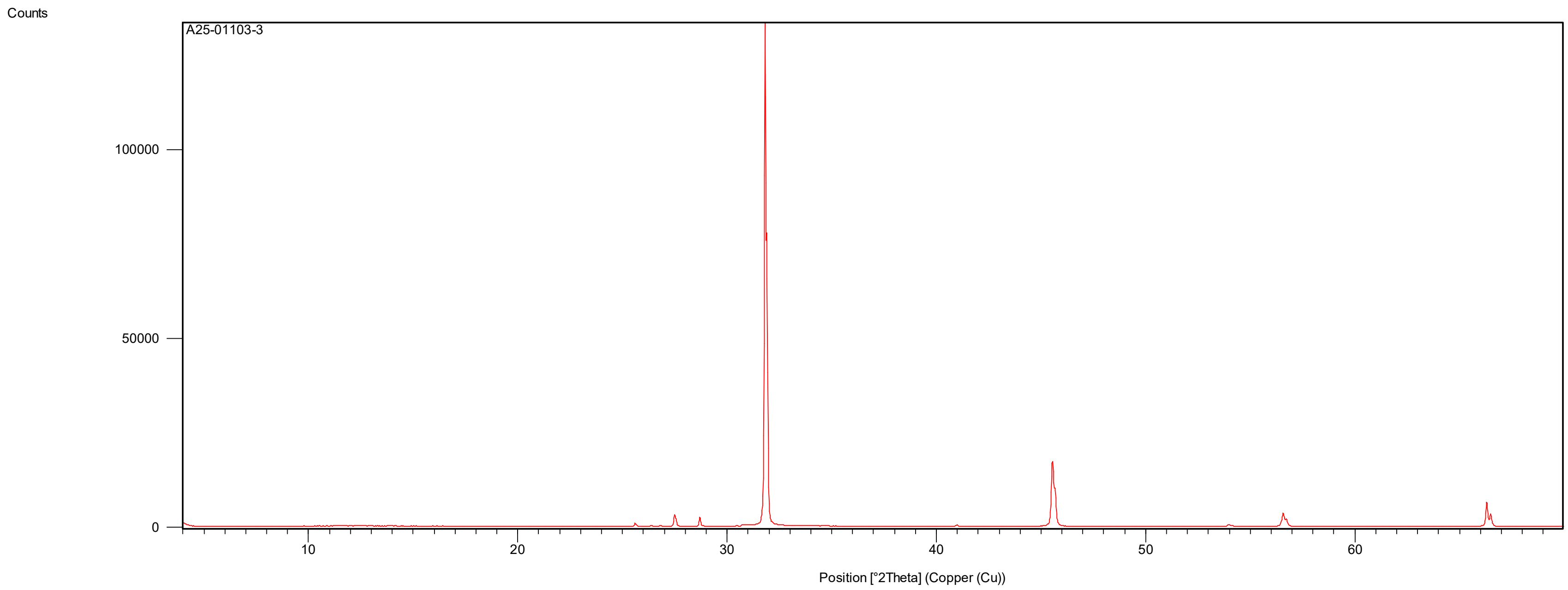
Counts
A25-01103-3
100000
50000
0
10
20
30
40
50
60
Position [°2Theta] (Copper (Cu))

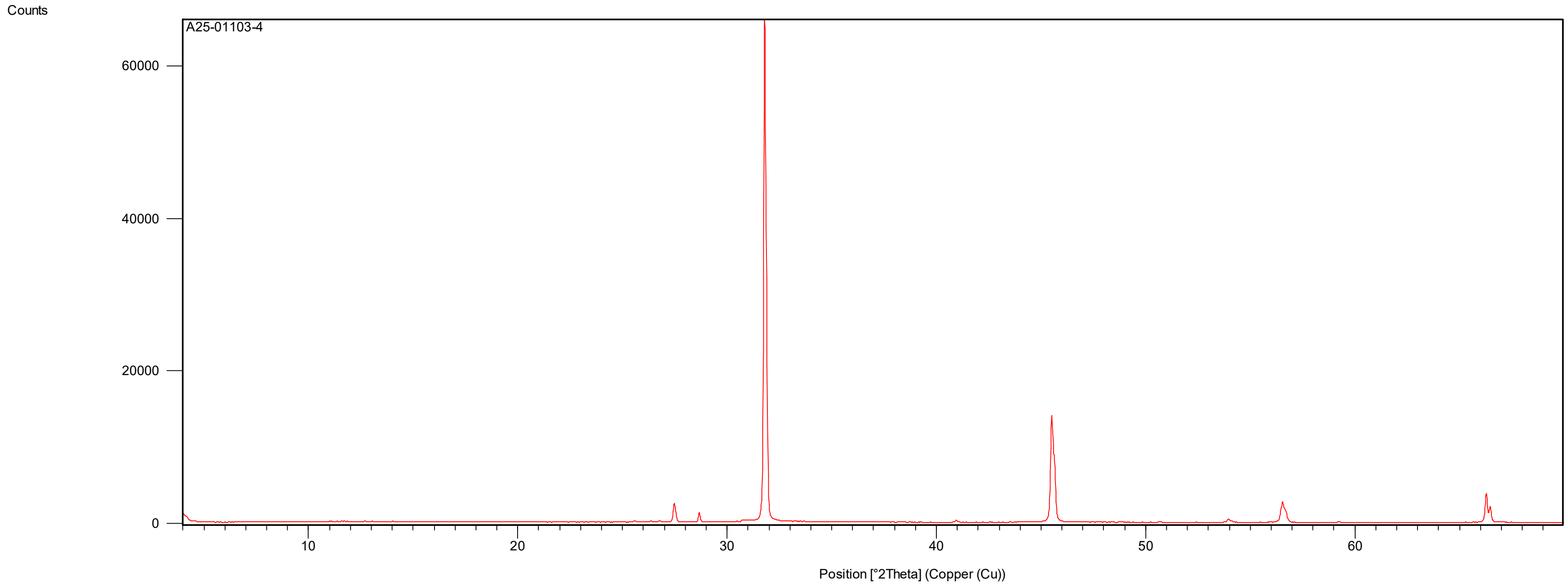
Counts
A25-01103-4
60000
40000
20000
0
10
20
30
40
50
60
Position [°2Theta] (Copper (Cu))

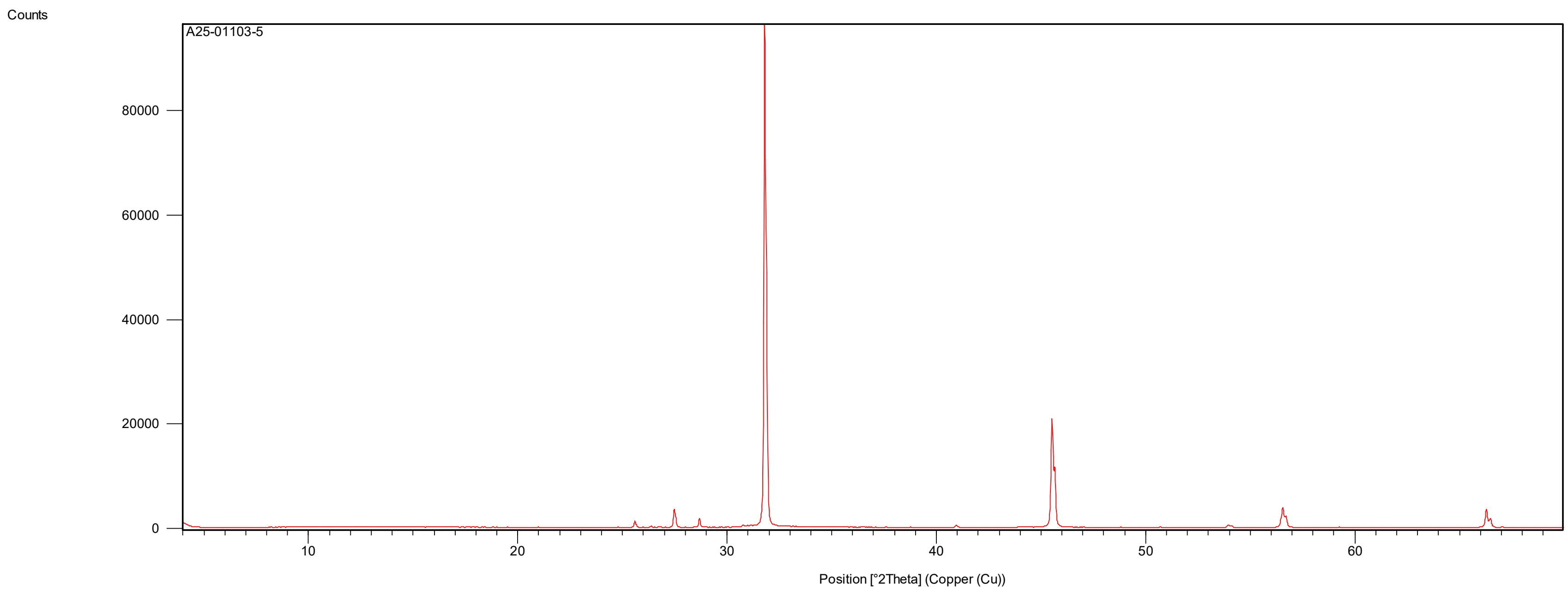
Counts
A25-01103-5
80000
60000
40000
20000
0
10
20
30
40
50
60
Position [°2Theta] (Copper (Cu))

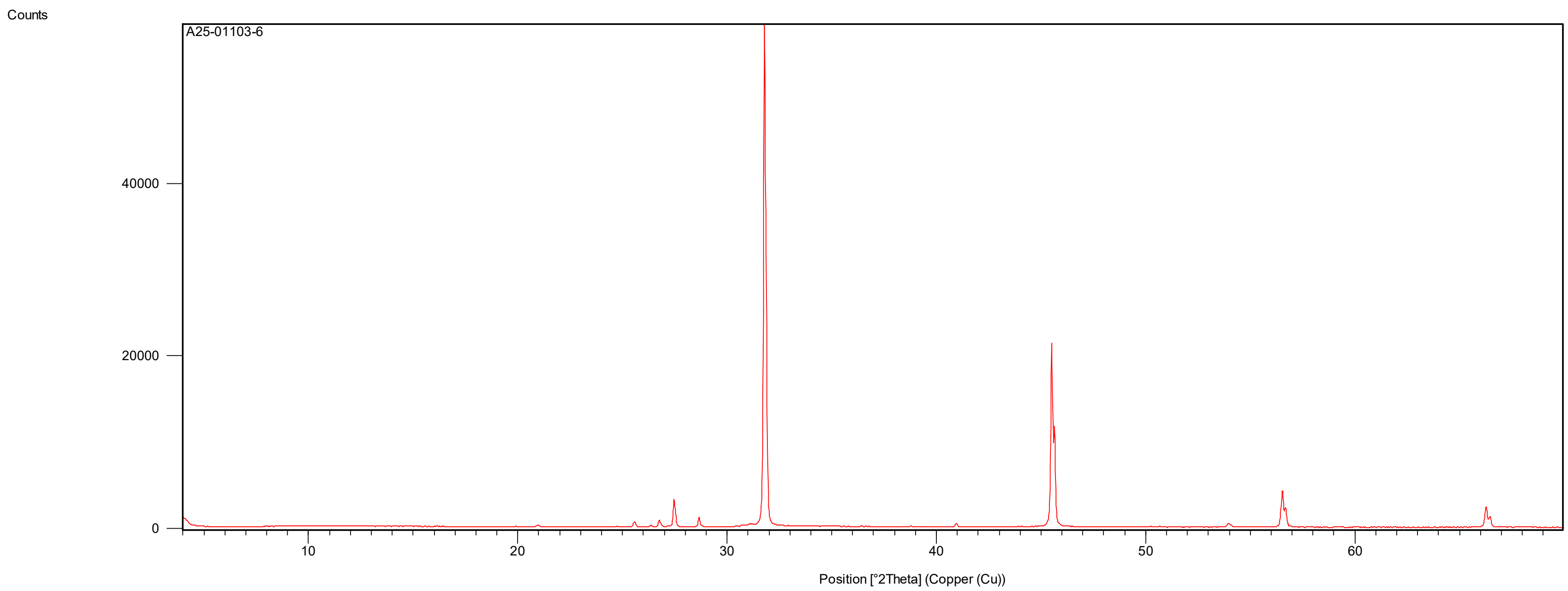
Counts
A25-01103-6
40000
20000
0
10
20
30
40
50
60
Position [°2Theta] (Copper (Cu))

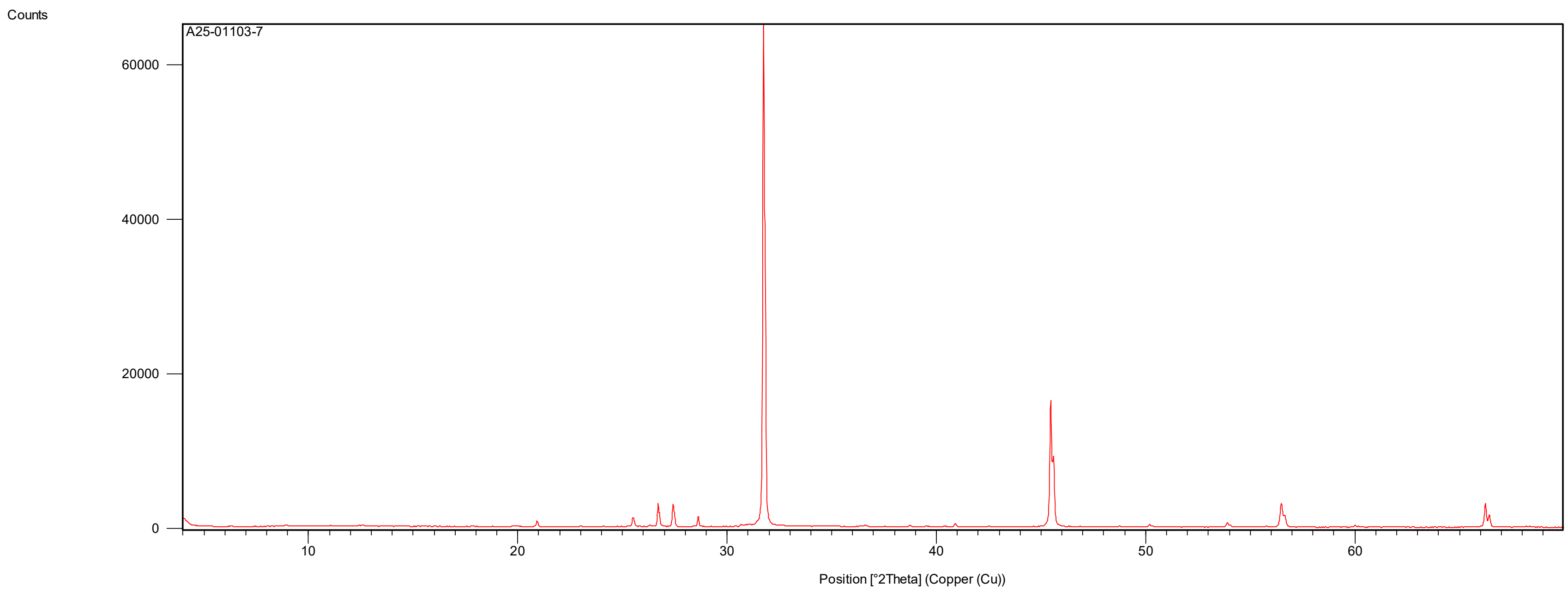
Counts
A25-01103-7
60000
40000
20000
0
10
20
30
40
50
60
Position [°2Theta] (Copper (Cu))

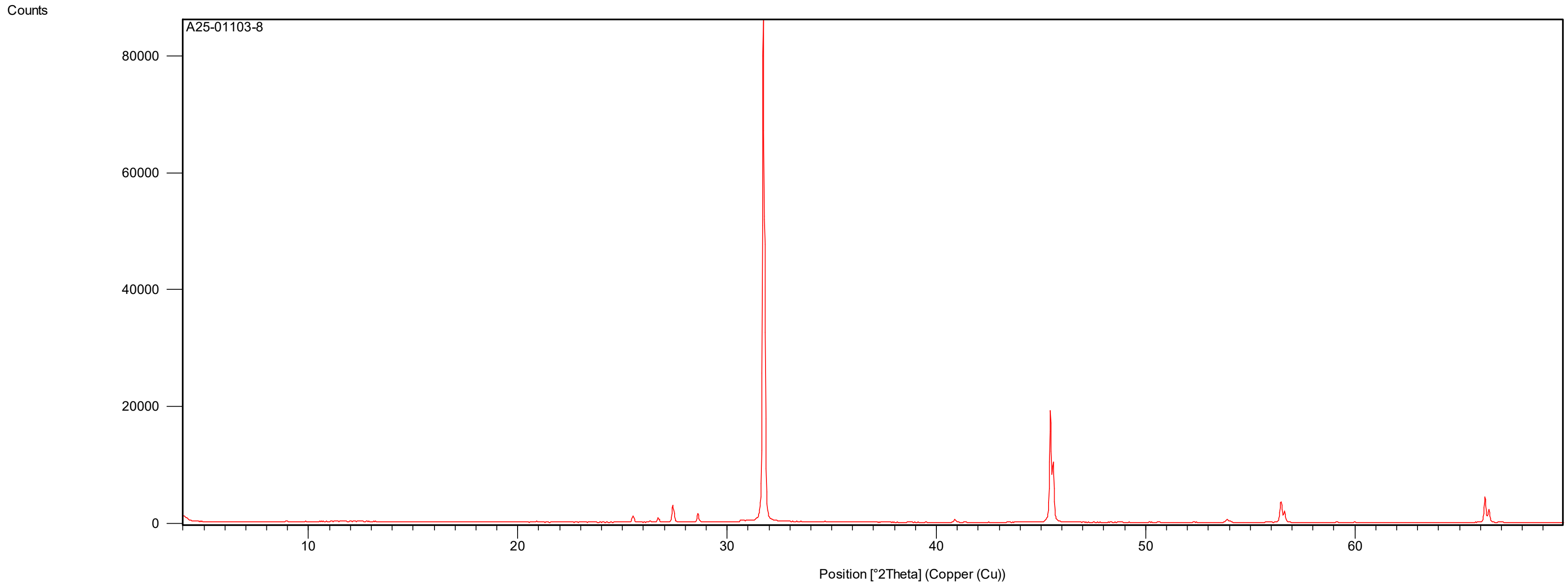
Counts
A25-01103-8
80000
60000
40000
20000
0
10
20
30
40
50
60
Position [°2Theta] (Copper (Cu))

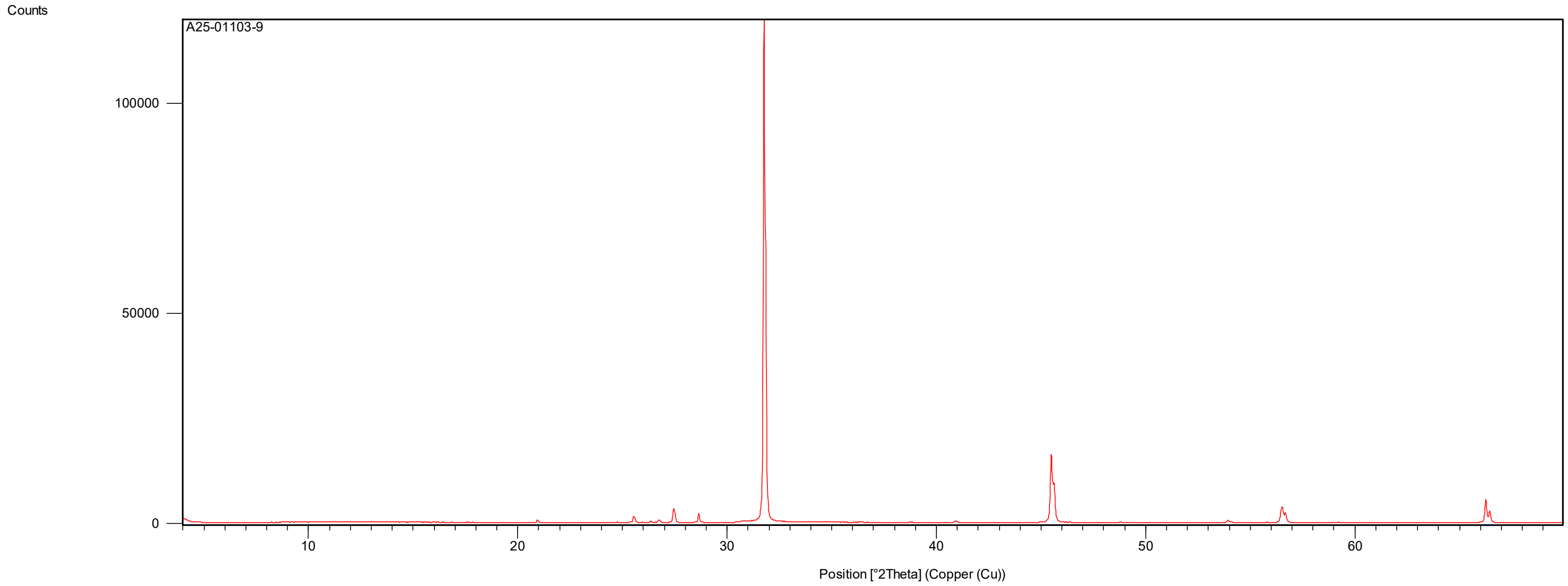
Counts
A25-01103-9
100000
50000
0
10
20
30
40
50
60
Position [°2Theta] (Copper (Cu))

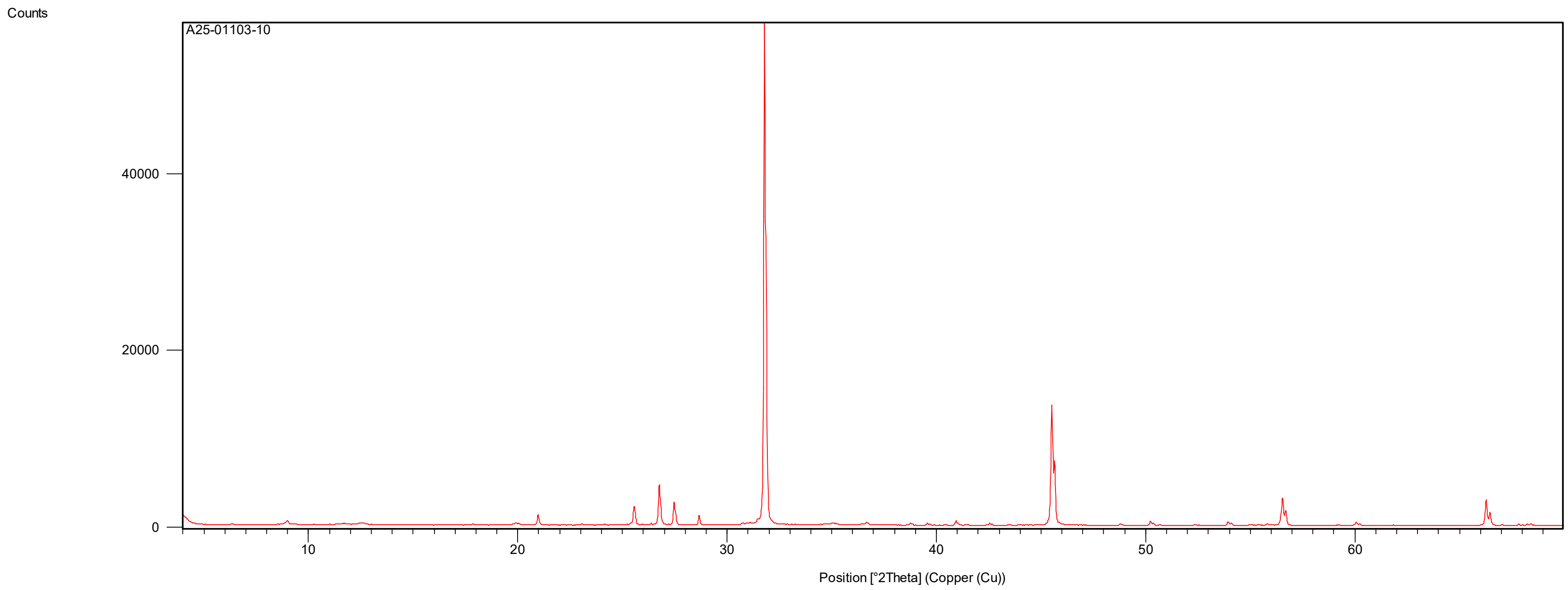
Counts
A25-01103-10
40000
20000
0
10
20
30
40
50
60
Position [°2Theta] (Copper (Cu))

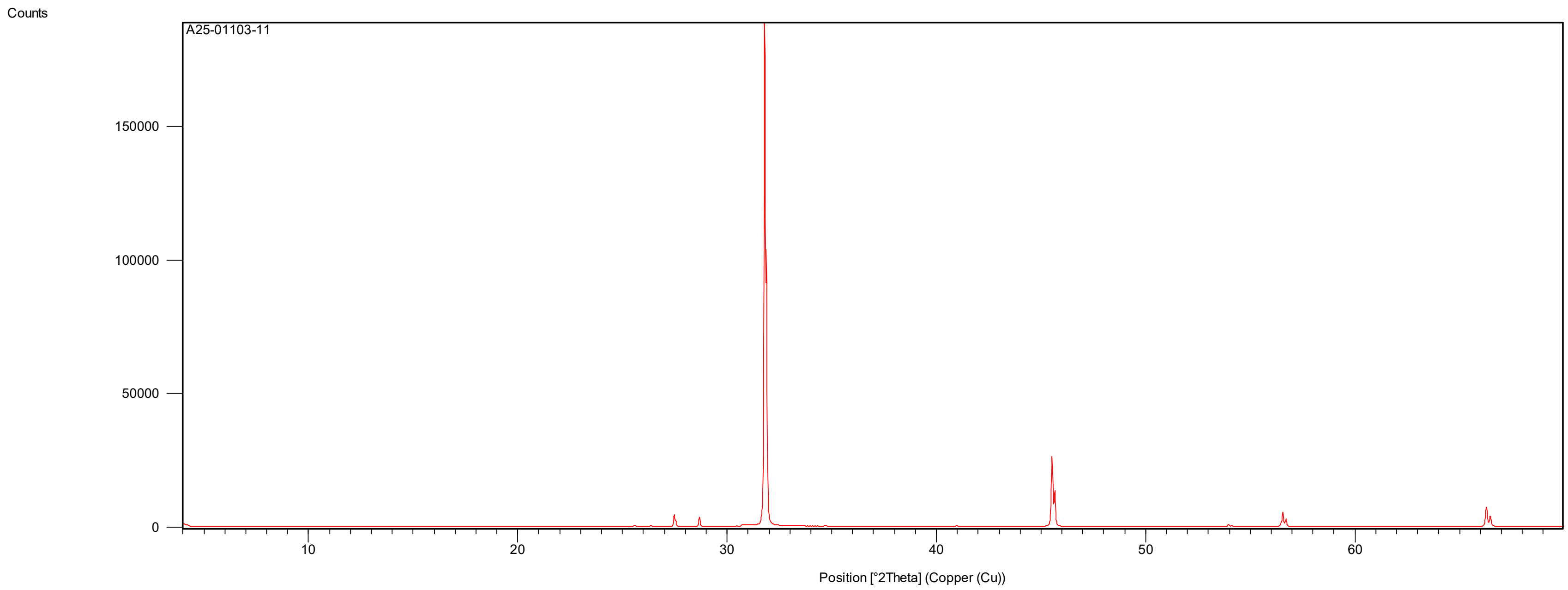
Counts
A25-01103-11
150000
100000
50000
0
10
20
30
40
50
60
Position [°2Theta] (Copper (Cu))

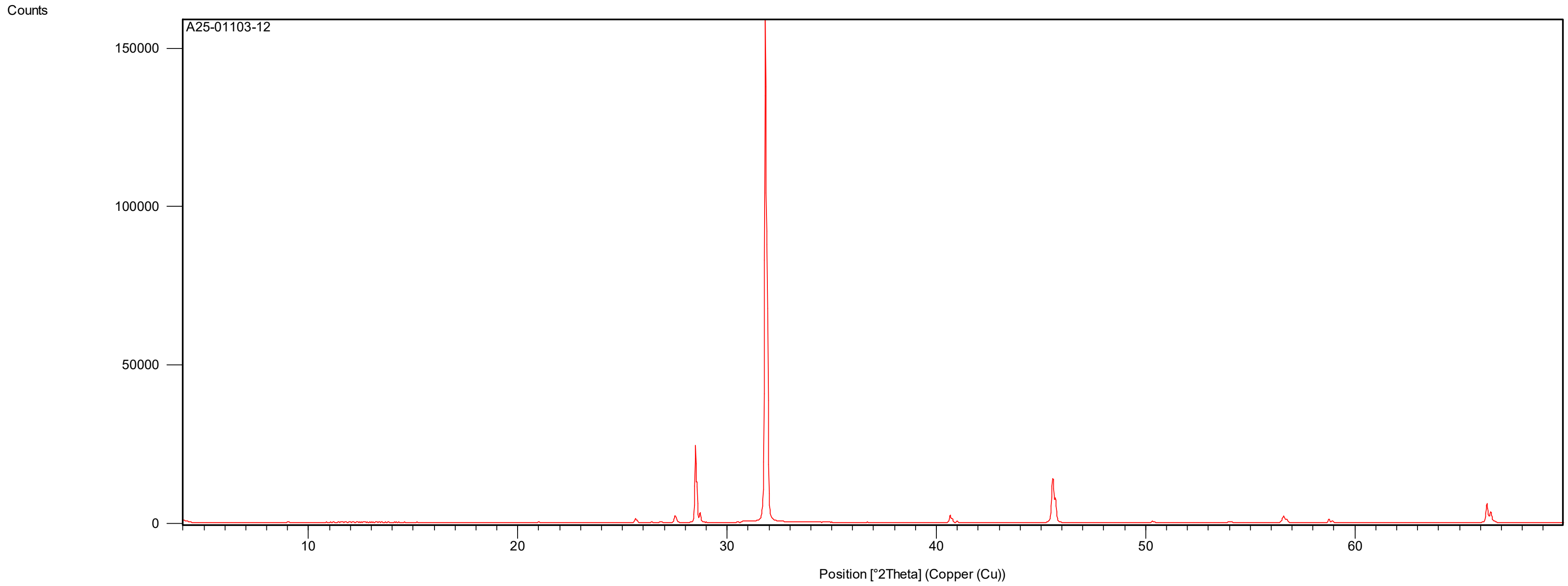
Counts
A25-01103-12
150000
100000
50000
0
10
20
30
40
50
60
Position [°2Theta] (Copper (Cu))

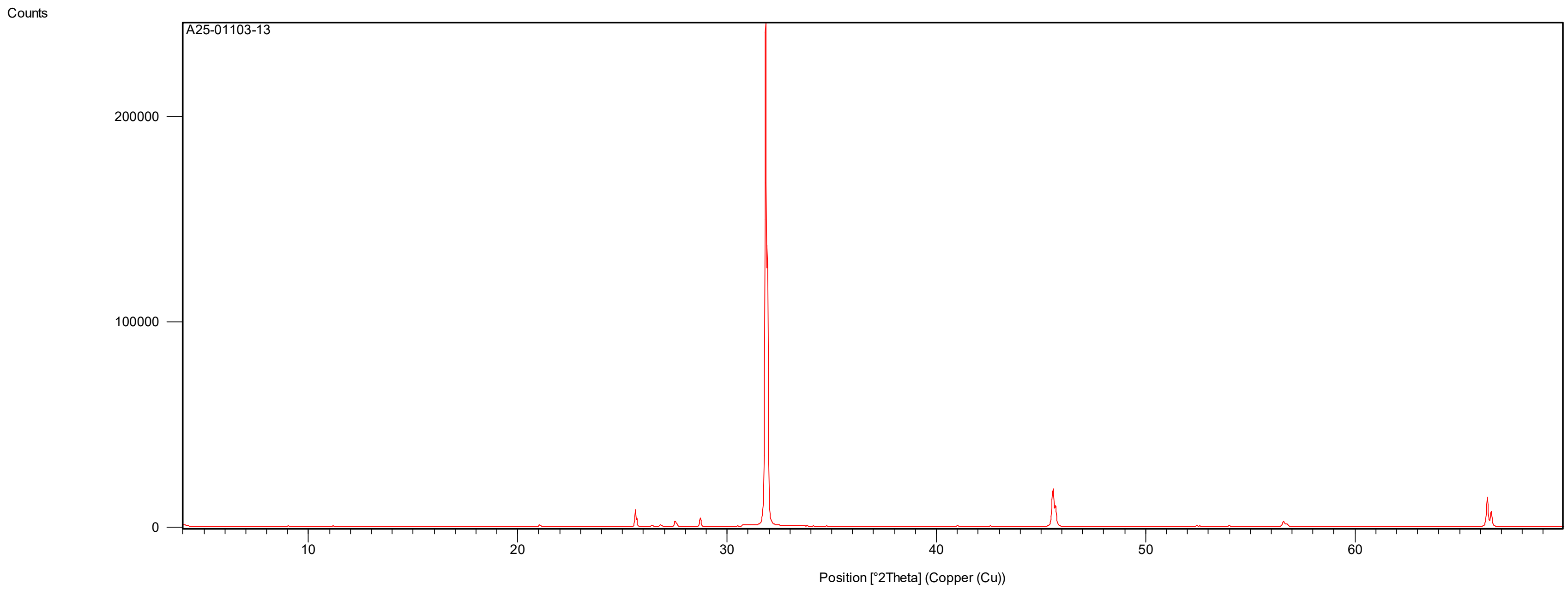
Counts
A25-01103-13
200000
100000
0
10
20
30
40
50
60
Position [°2Theta] (Copper (Cu))

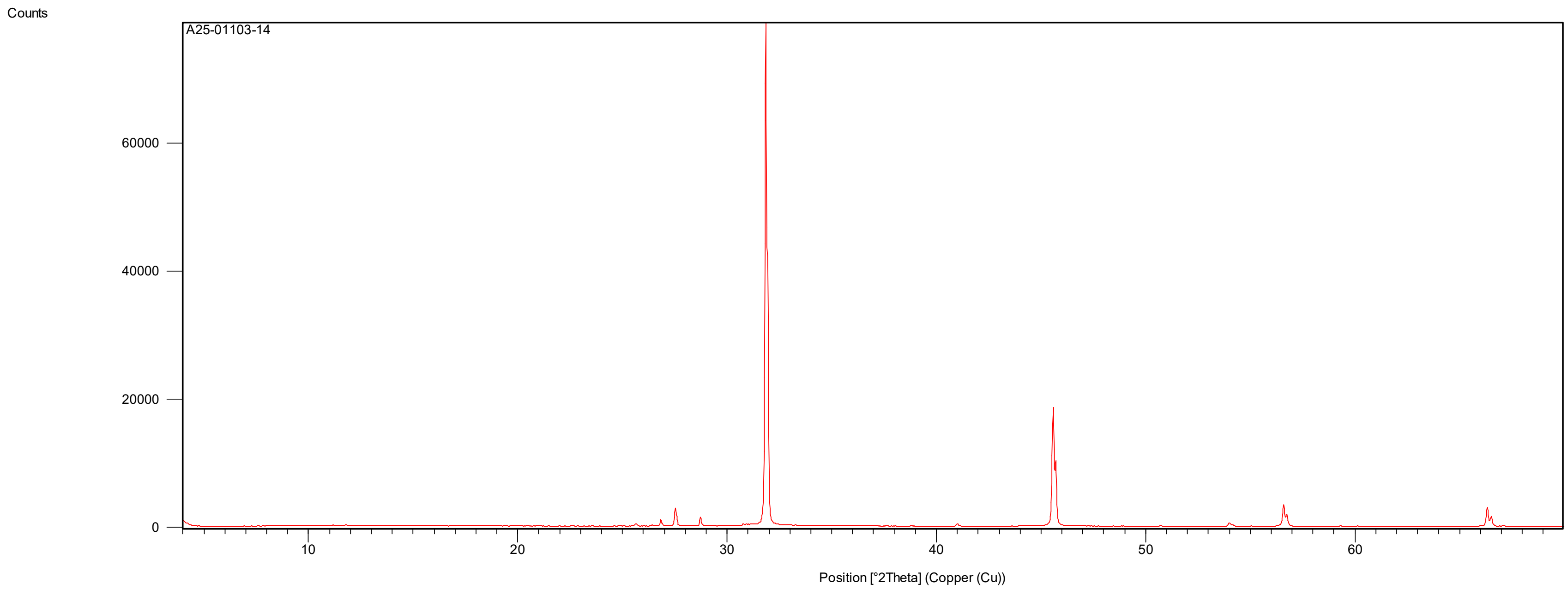
Counts
A25-01103-14
60000
40000
20000
0
10
20
30
40
50
60
Position [°2Theta] (Copper (Cu))

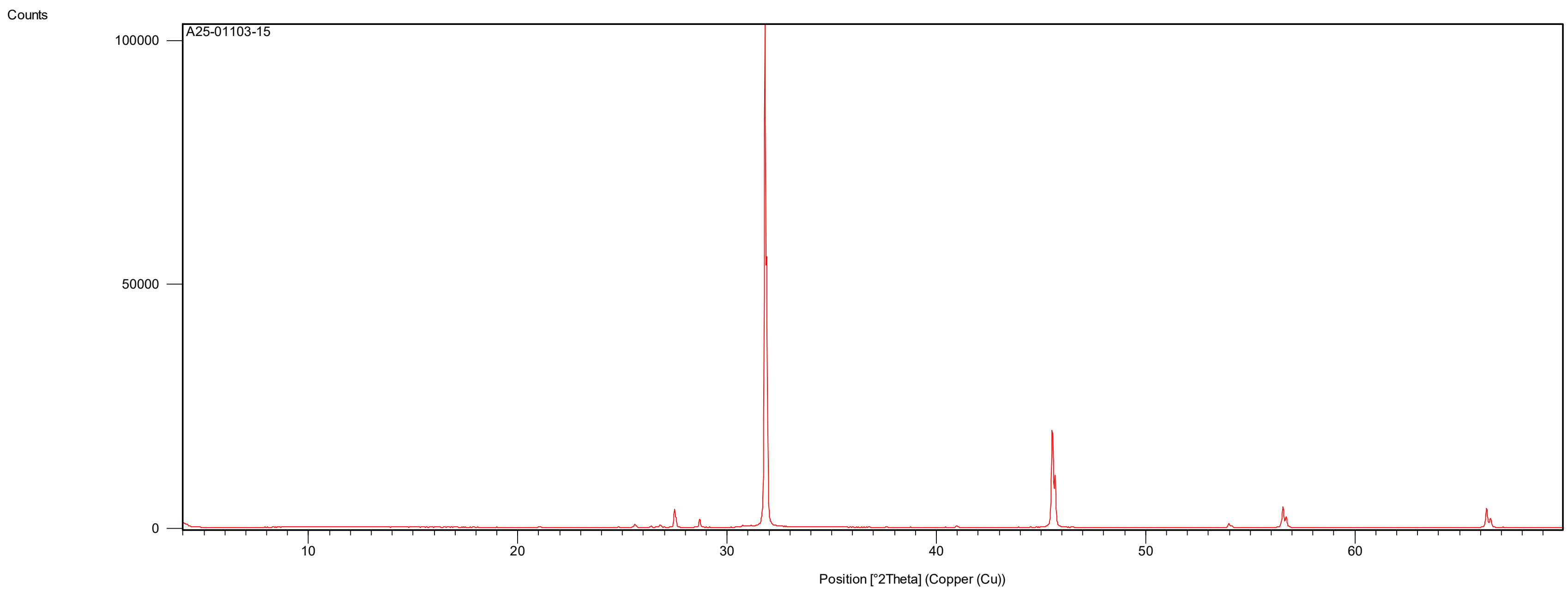
Counts
A25-01103-15
100000
50000
0
10
20
30
40
50
60
Position [°2Theta] (Copper (Cu))

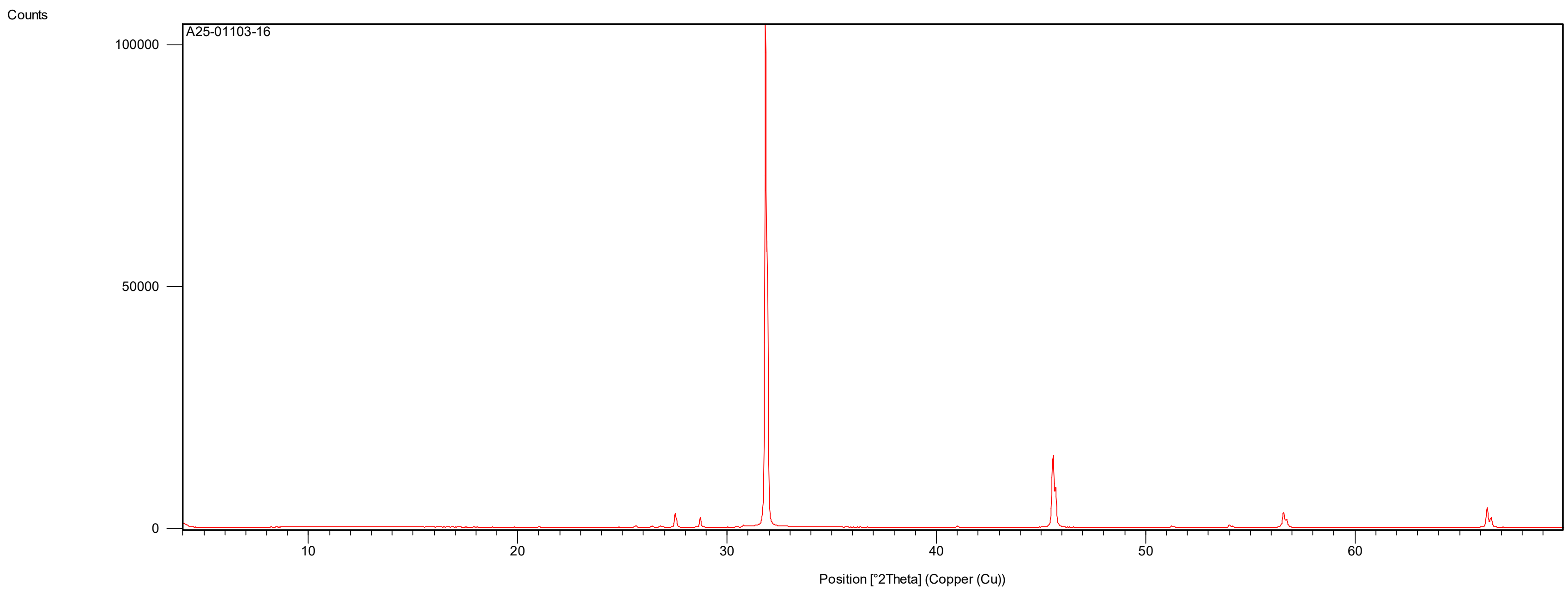
Counts
A25-01103-16
100000
50000
0
10
20
30
40
50
60
Position [°2Theta] (Copper (Cu))

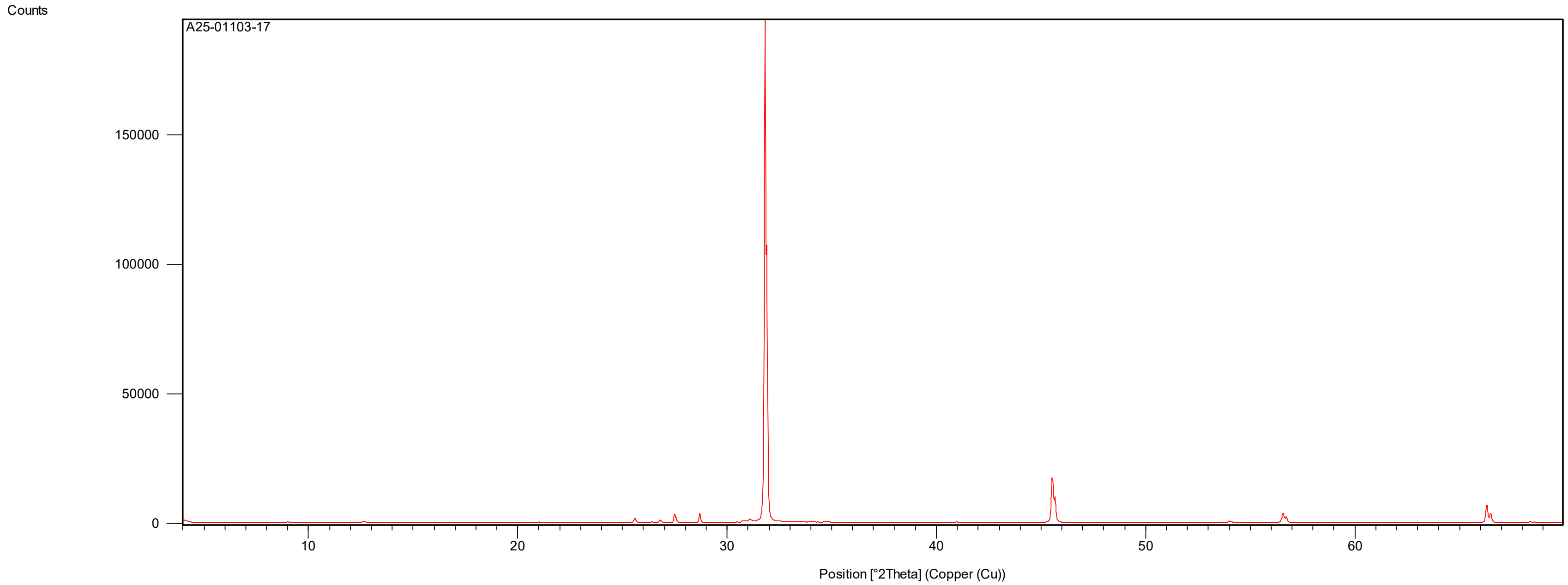
Counts
A25-01103-17
150000
100000
50000
0
10
20
30
40
50
60
Position [°2Theta] (Copper (Cu))

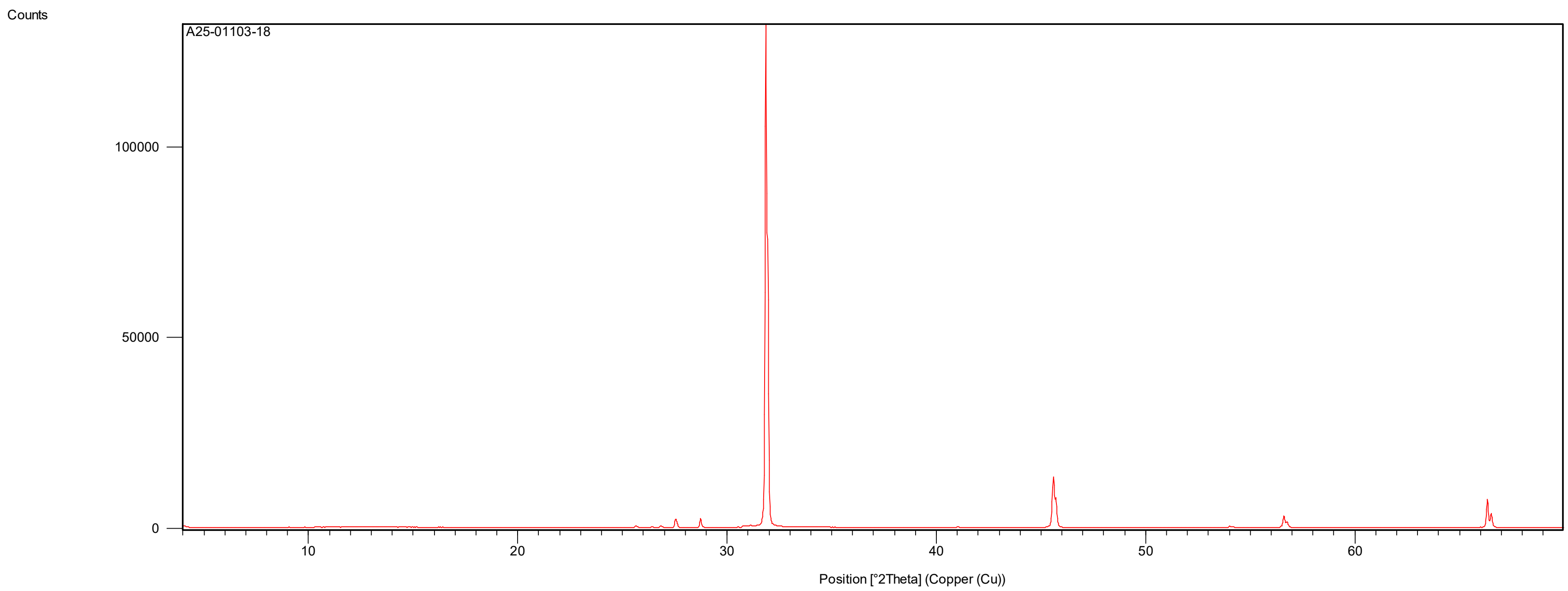
Counts
A25-01103-18
100000
50000
0
10
20
30
40
50
60
Position [°2Theta] (Copper (Cu))

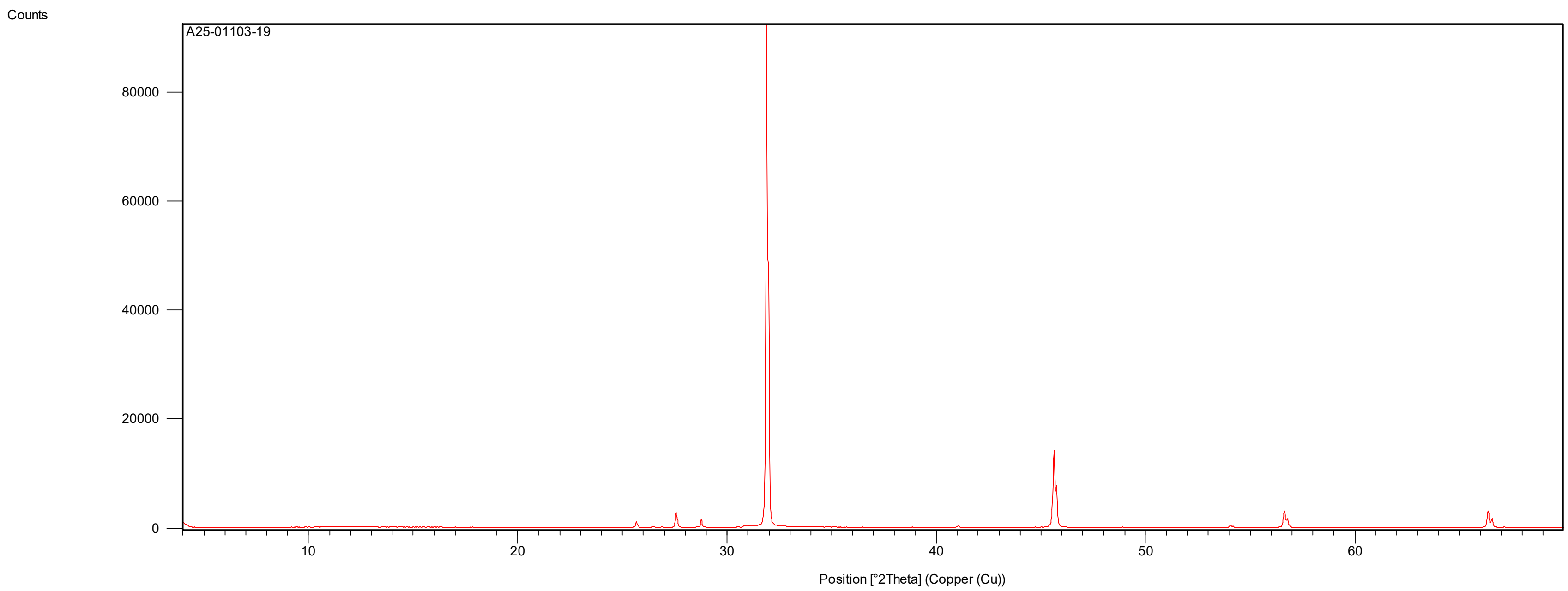
Counts
A25-01103-19
80000
60000
40000
20000
0
10
20
30
40
50
60
Position [°2Theta] (Copper (Cu))

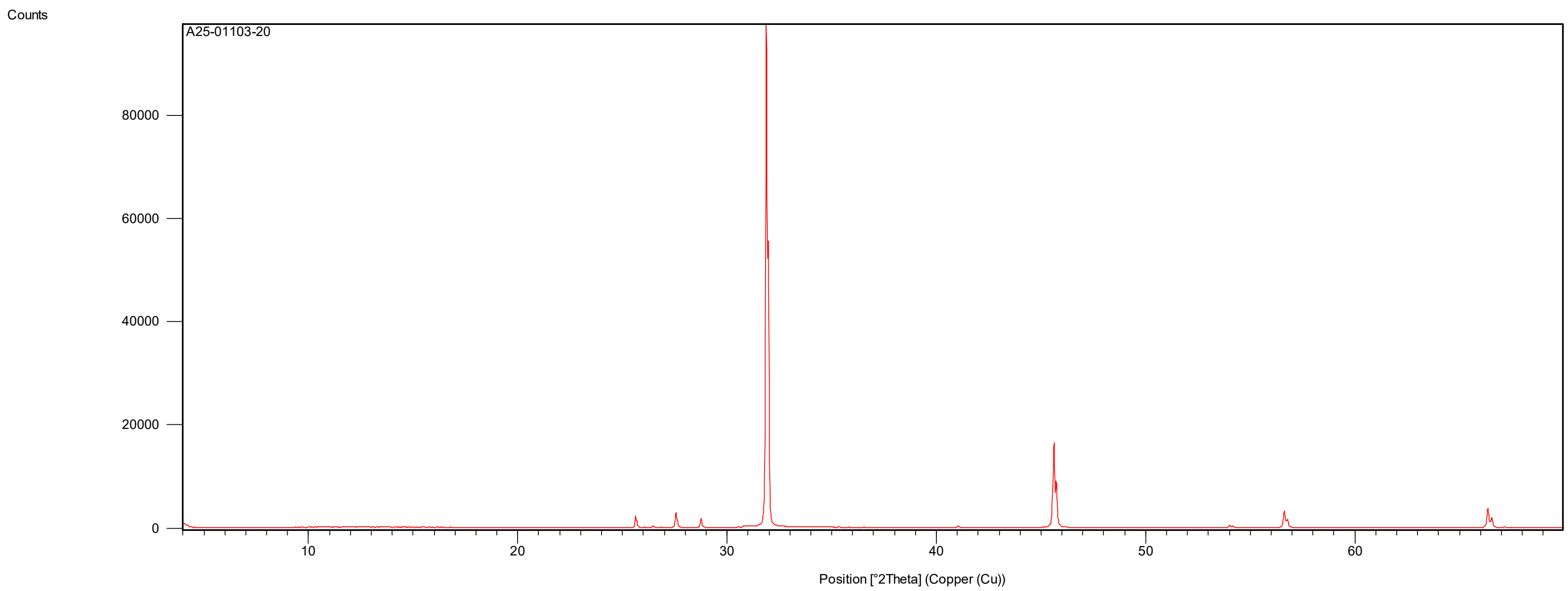
Counts
A25-01103-20
80000
60000
40000
20000
0
10
20
30
40
50
60
Position [°2Theta] (Copper (Cu))

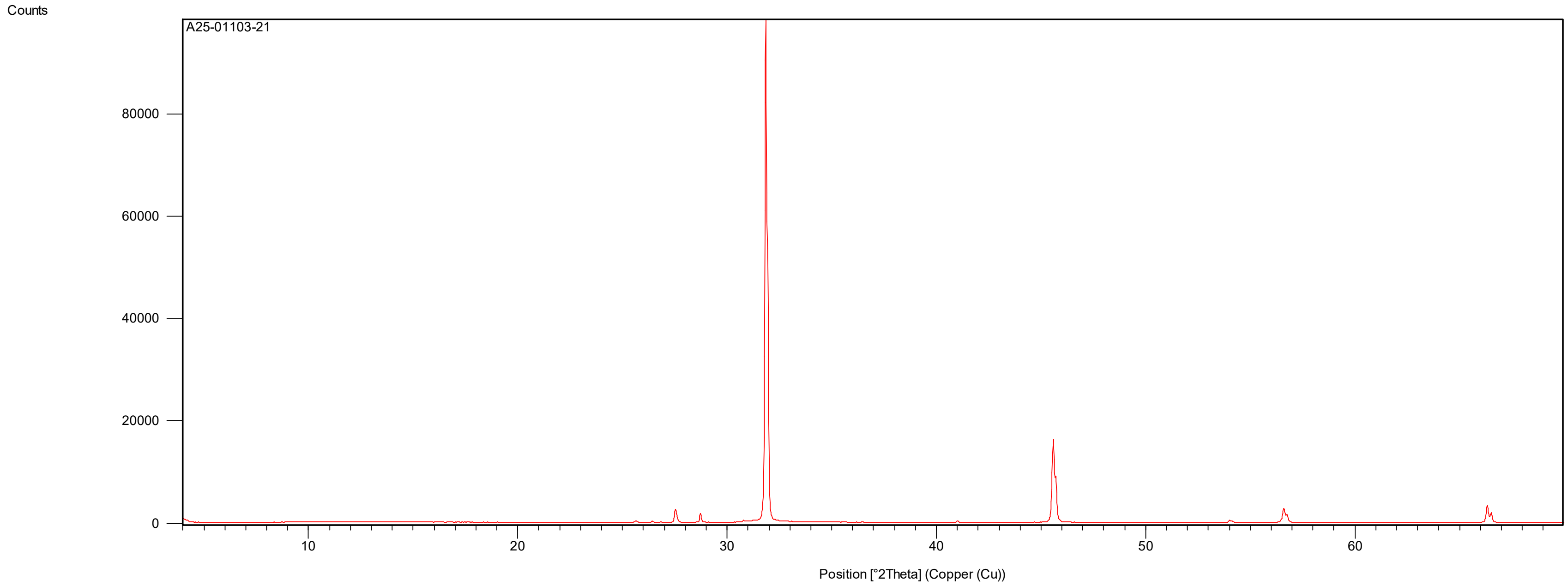
Counts
A25-01103-21
80000
60000
40000
20000
0
10
20
30
40
50
60
Position [°2Theta] (Copper (Cu))

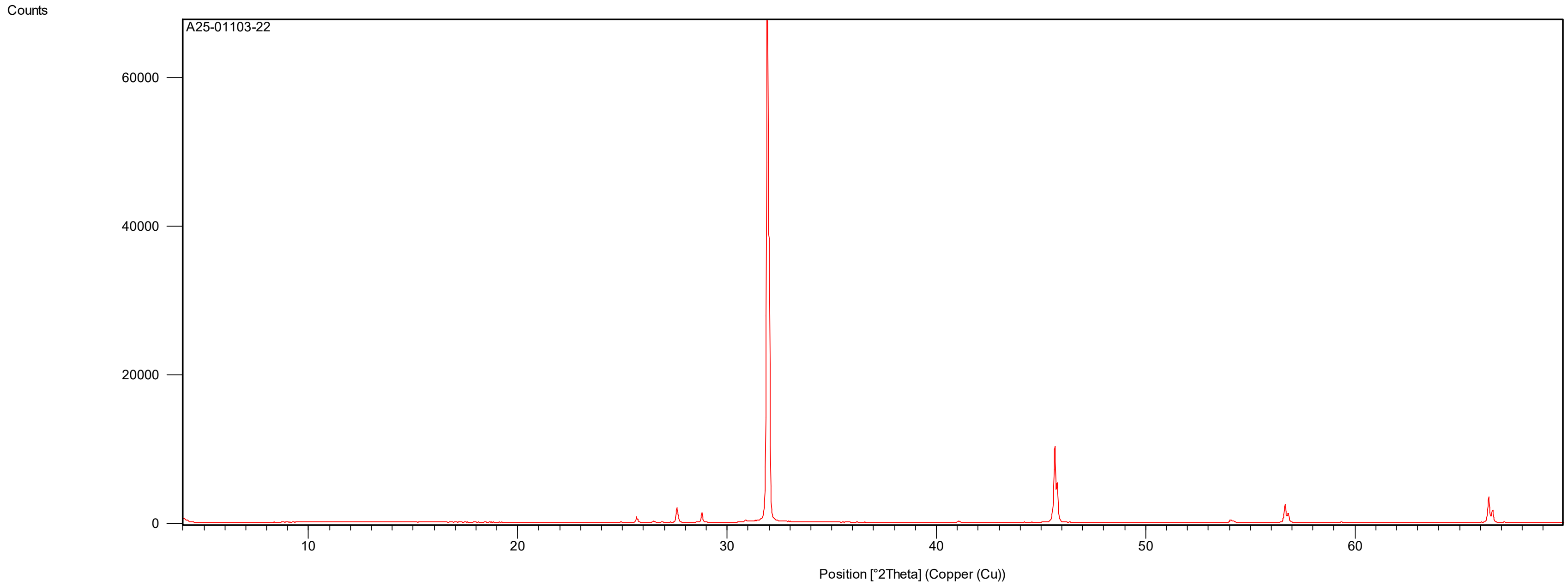
Counts
A25-01103-22
60000
40000
20000
0
10
20
30
40
50
60
Position [°2Theta] (Copper (Cu))

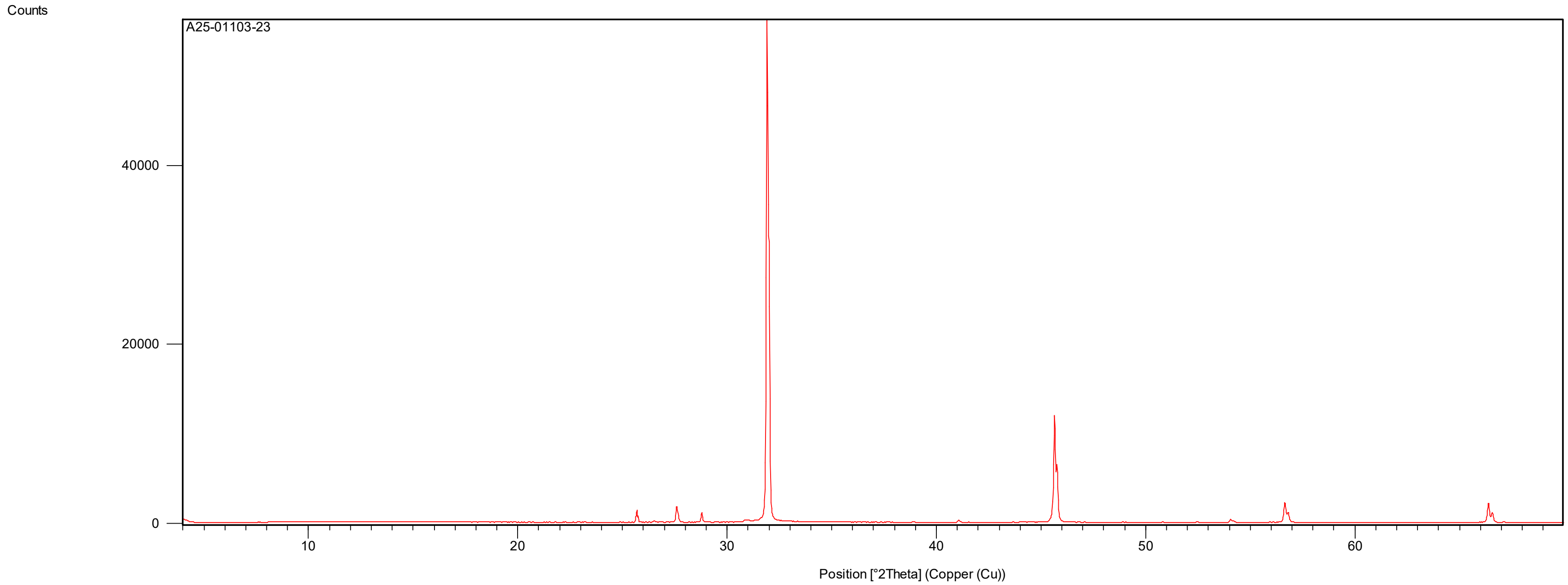
Counts
A25-01103-23
40000
20000
0
10
20
30
40
50
60
Position [°2Theta] (Copper (Cu))

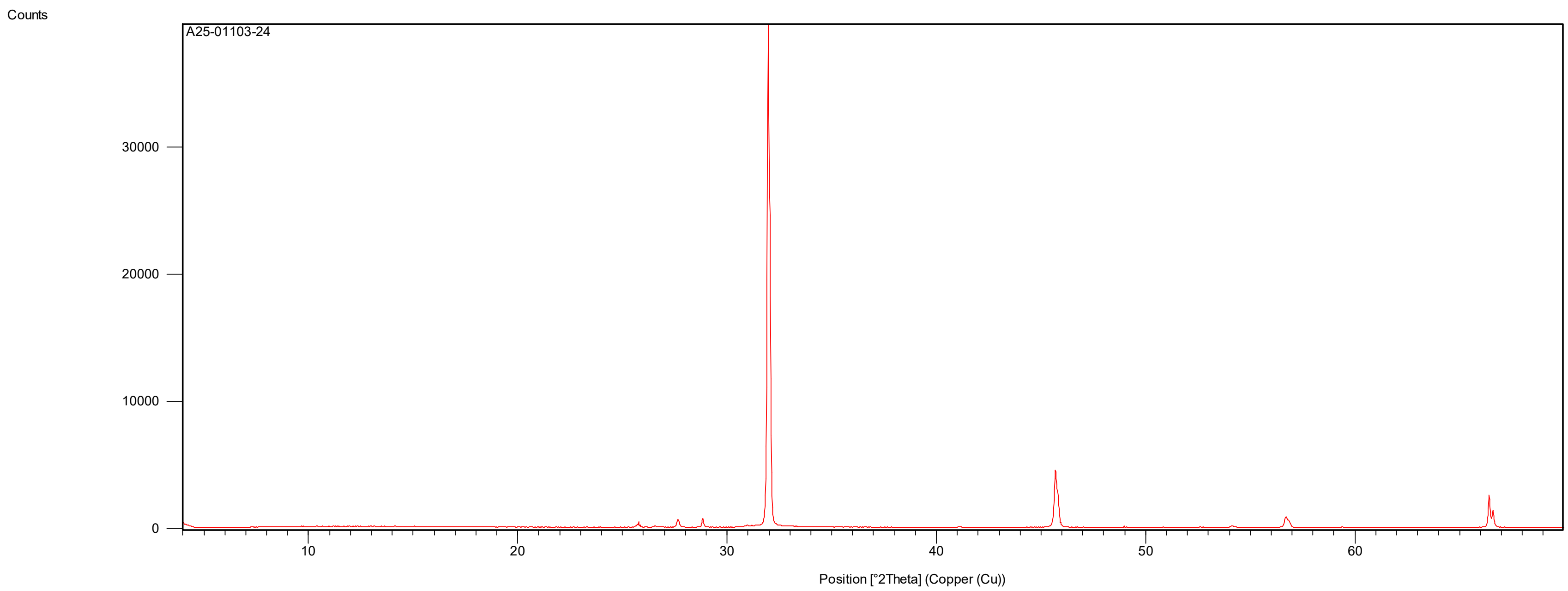

Counts
A25-01103-24
30000
20000
10000
0
10
20
30
40
50
60
Position [°2Theta] (Copper (Cu))

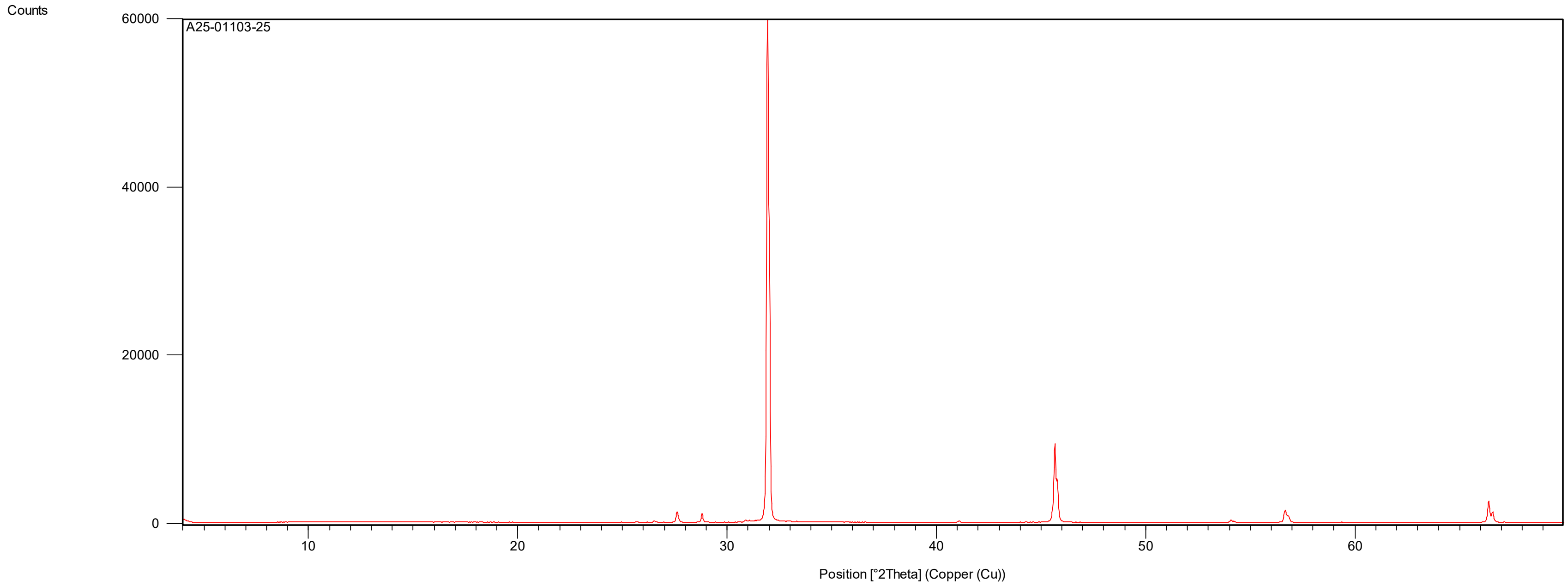
Counts
60000
40000
20000
0
A25-01103-25
10
20
30
40
50
60
Position [°2Theta] (Copper (Cu))

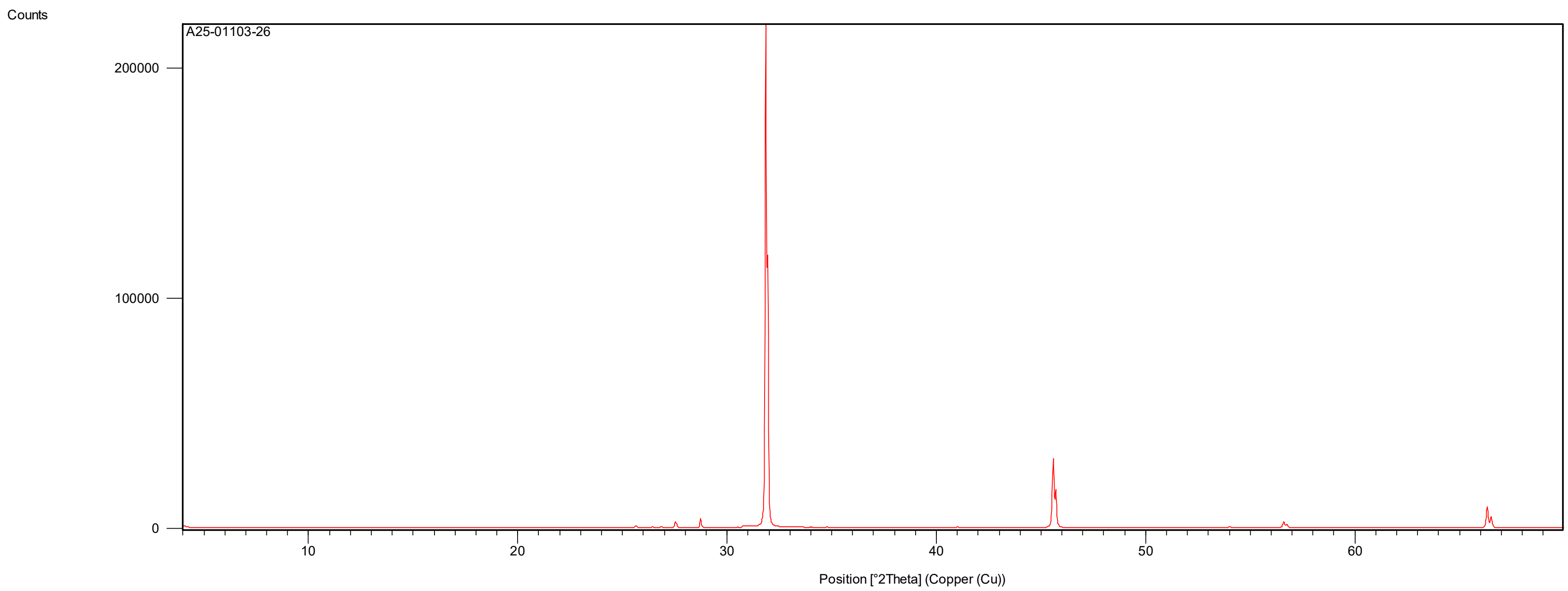
Counts
A25-01103-26
200000
100000
0
10
20
30
40
50
60
Position [°2Theta] (Copper (Cu))

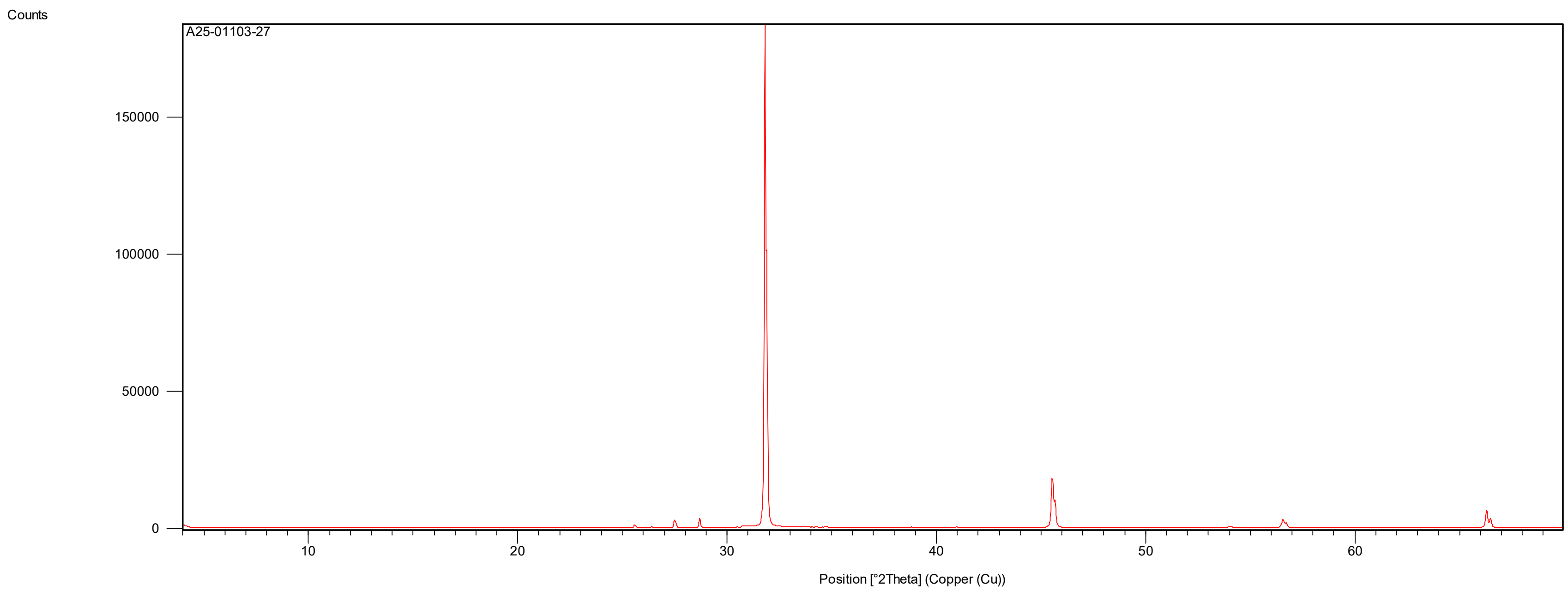
Counts
A25-01103-27
150000
100000
50000
0
10
20
30
40
50
60
Position [°2Theta] (Copper (Cu))

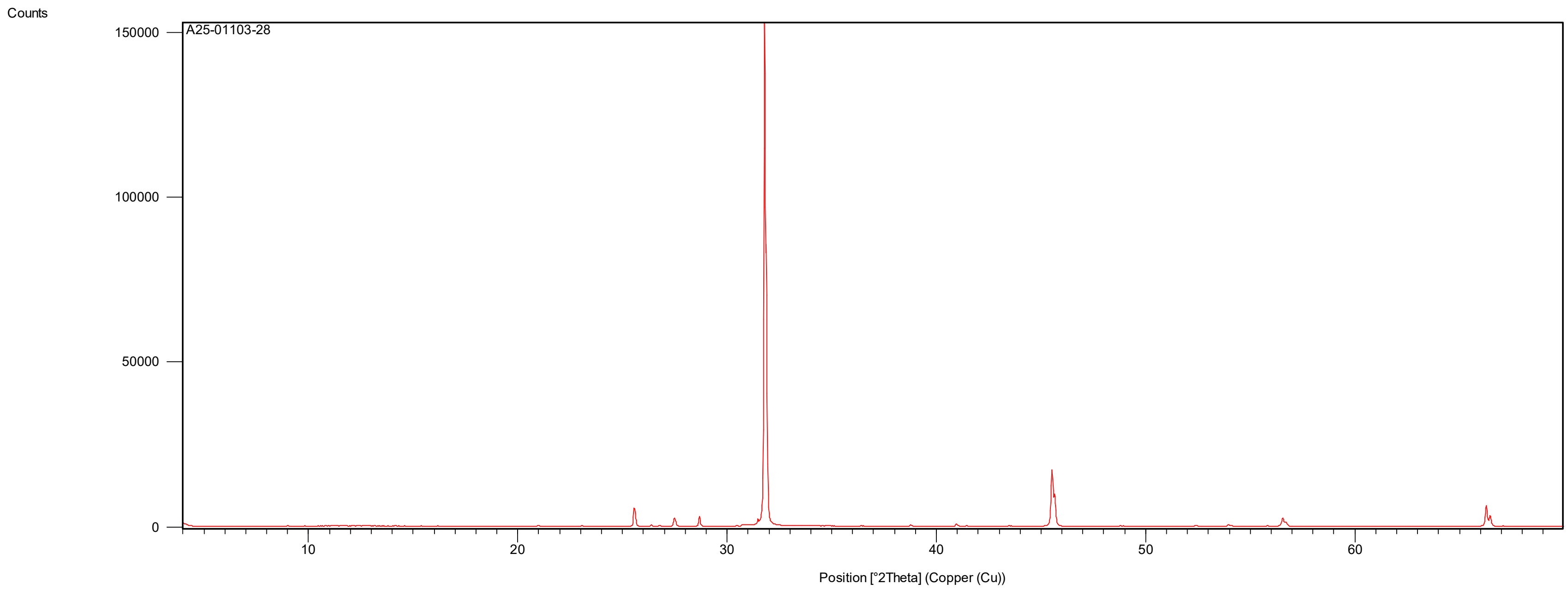
Counts
A25-01103-28
150000
100000
50000
0
10
20
30
40
50
60
Position [°2Theta] (Copper (Cu))

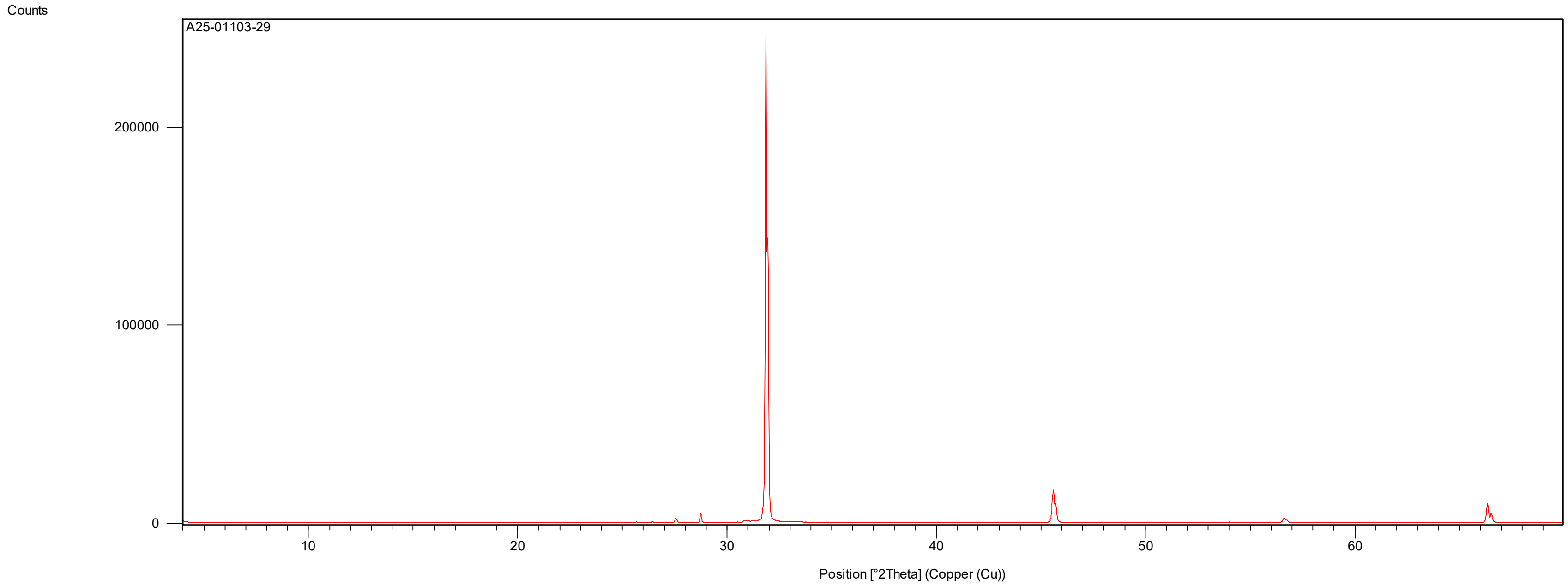
Counts
A25-01103-29
200000
100000
0
10
20
30
40
50
60
Position [°2Theta] (Copper (Cu))

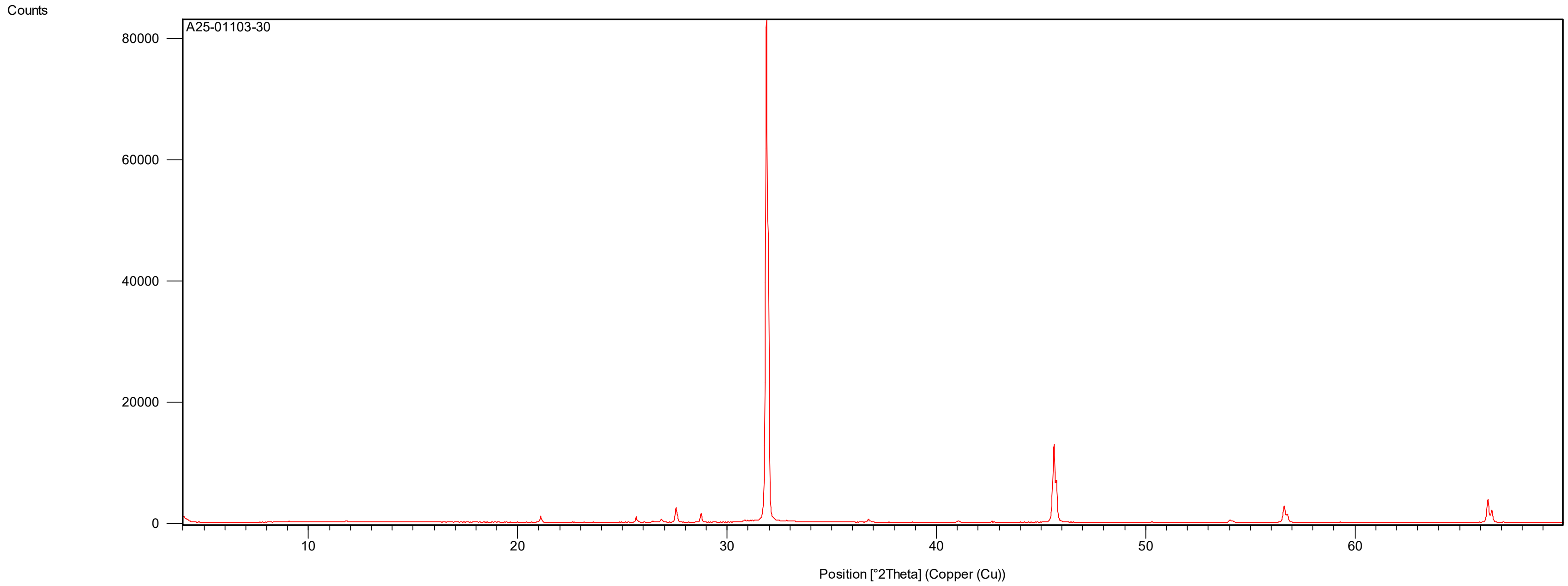
Counts
A25-01103-30
80000
60000
40000
20000
0
10
20
30
40
50
60
Position [°2Theta] (Copper (Cu))

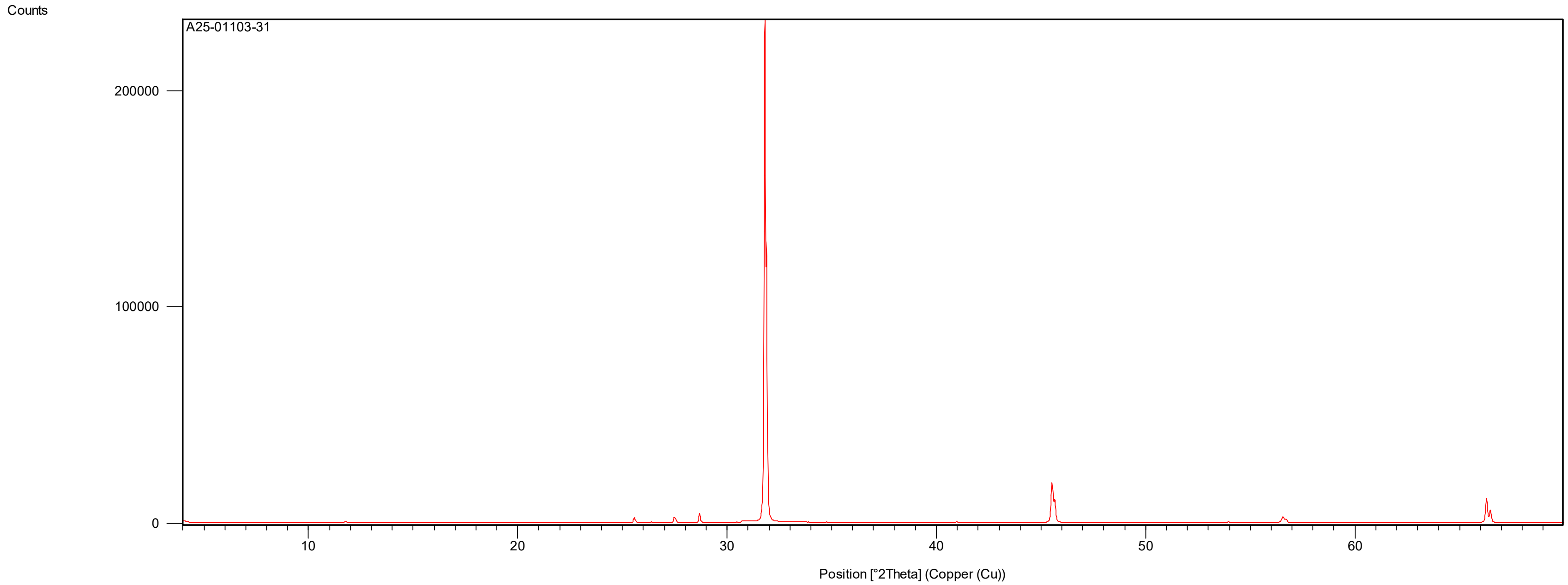
Counts
A25-01103-31
200000
100000
0
10
20
30
40
50
60
Position [°2Theta] (Copper (Cu))

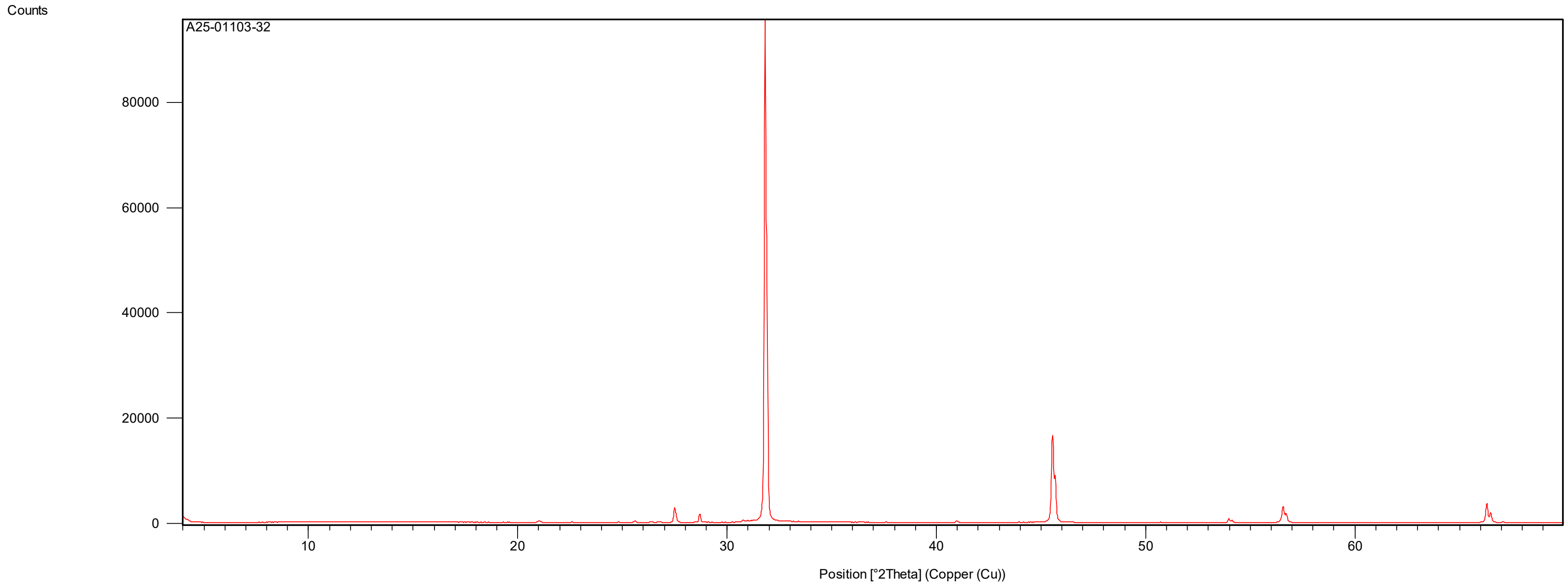
Counts
A25-01103-32
80000
60000
40000
20000
0
10
20
30
40
50
60
Position [°2Theta] (Copper (Cu))

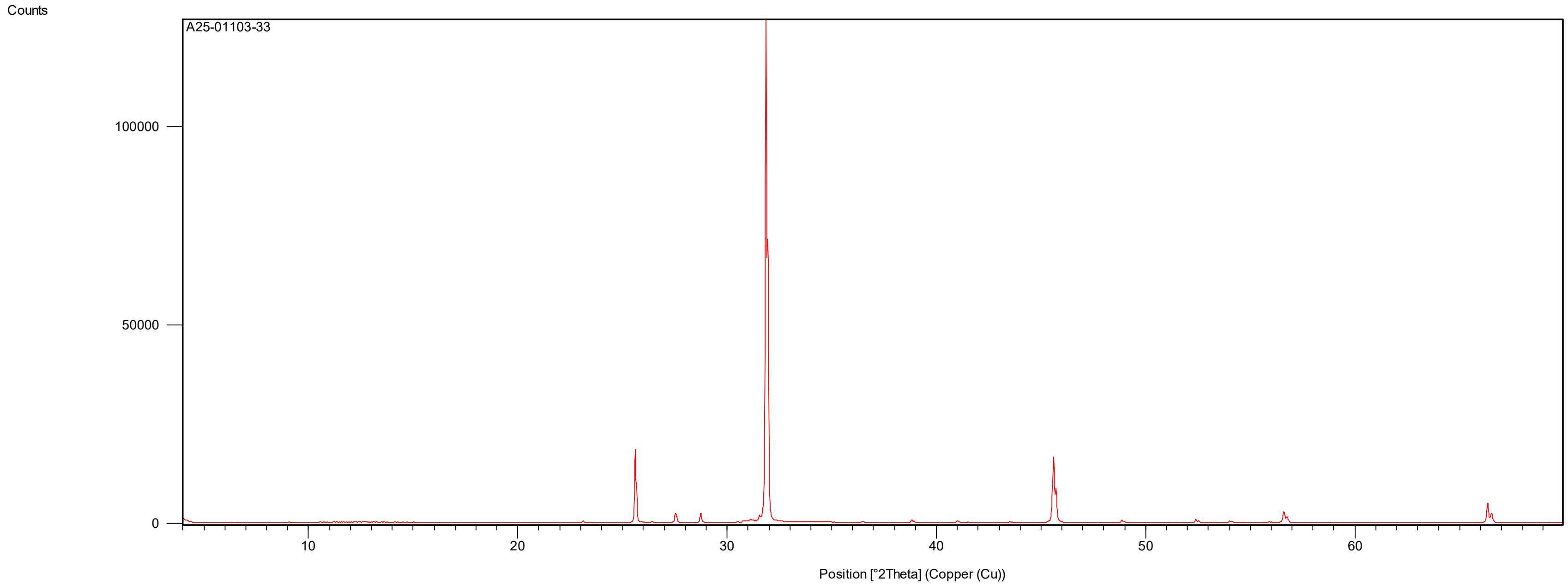
Counts
A25-01103-33
100000
50000
0
10
20
30
40
50
60
Position [°2Theta] (Copper (Cu))

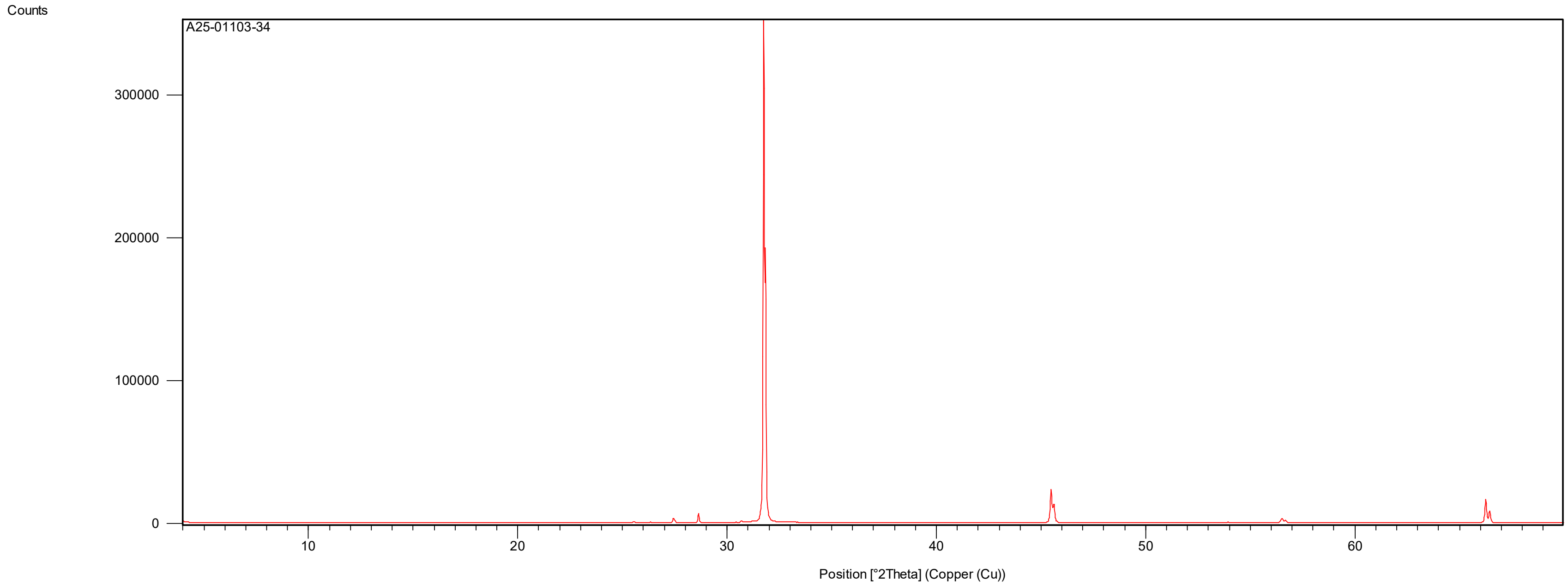
Counts
A25-01103-34
300000
200000
100000
0
10
20
30
40
50
60
Position [°2Theta] (Copper (Cu))

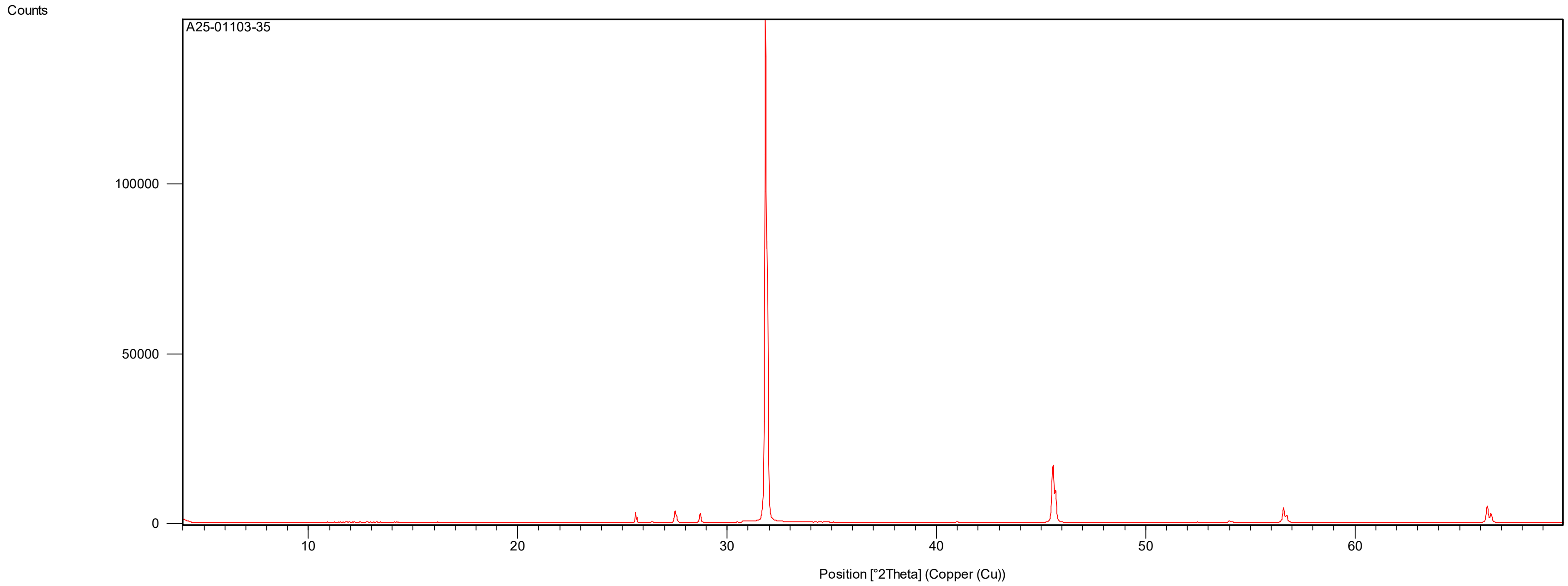
Counts
A25-01103-35
100000
50000
0
10
20
30
40
50
60
Position [°2Theta] (Copper (Cu))

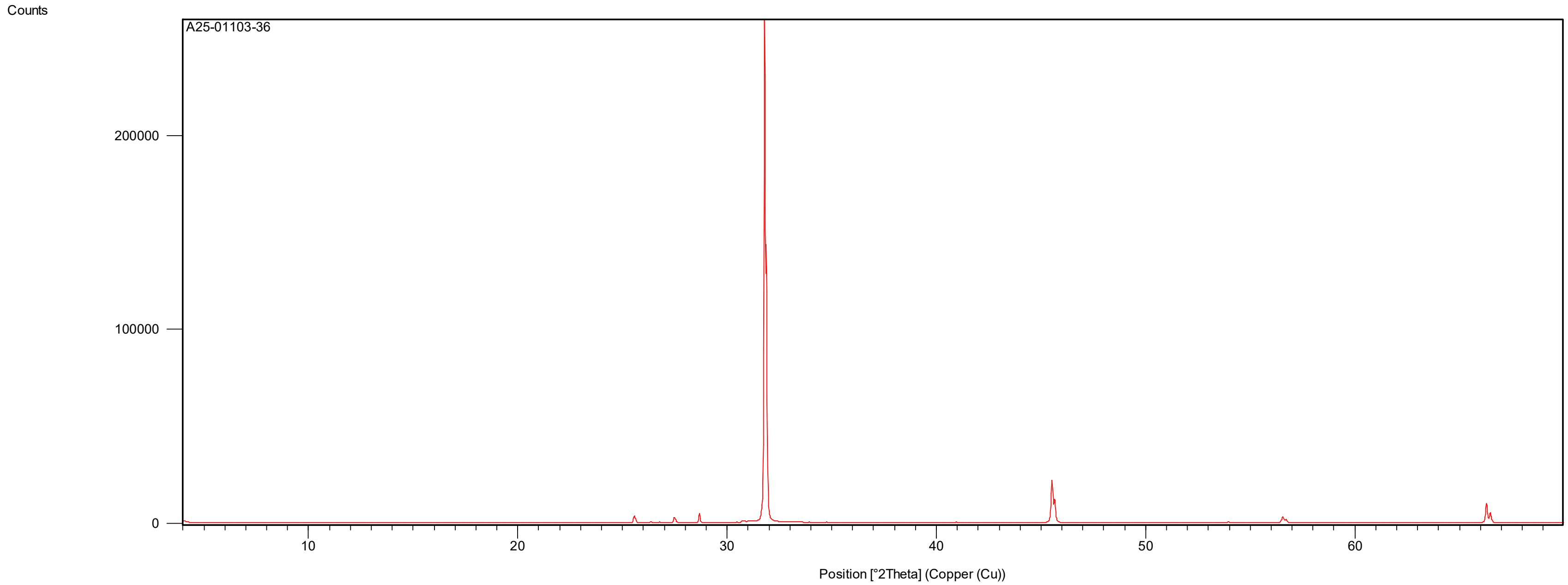
Counts
A25-01103-36
200000
100000
0
10
20
30
40
50
60
Position [°2Theta] (Copper (Cu))

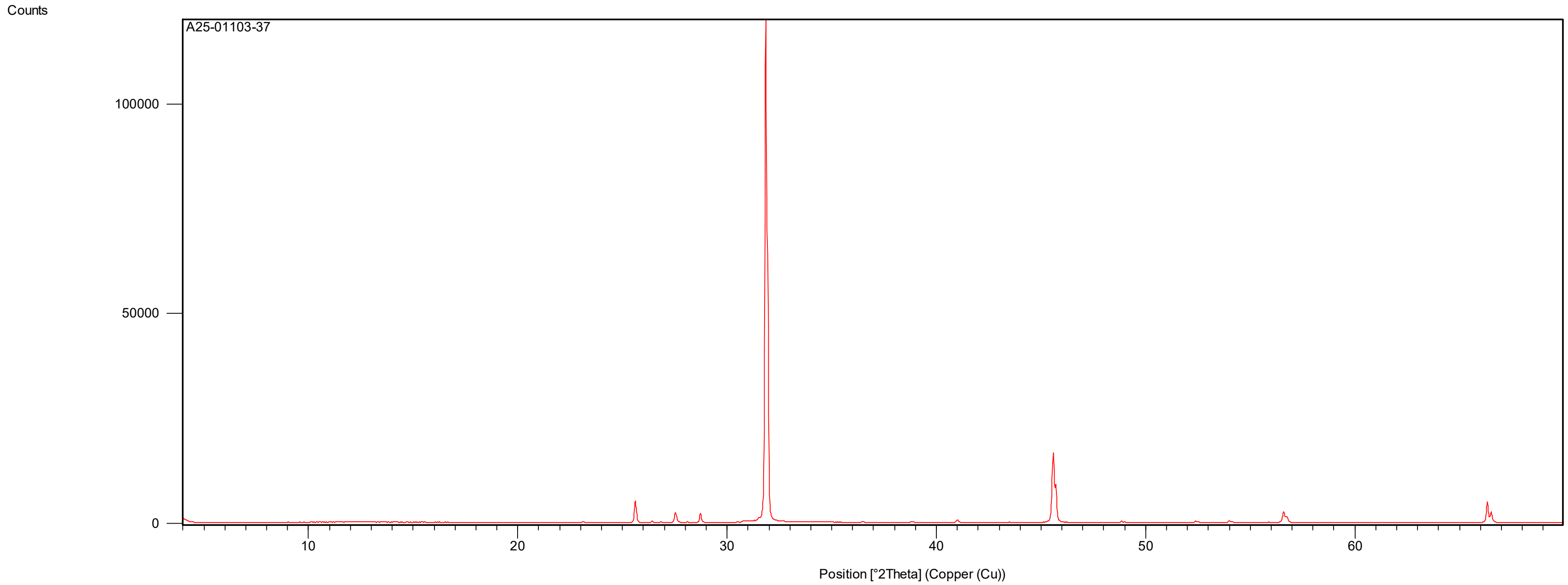
Counts
A25-01103-37
100000
50000
0
10
20
30
40
50
60
Position [°2Theta] (Copper (Cu))

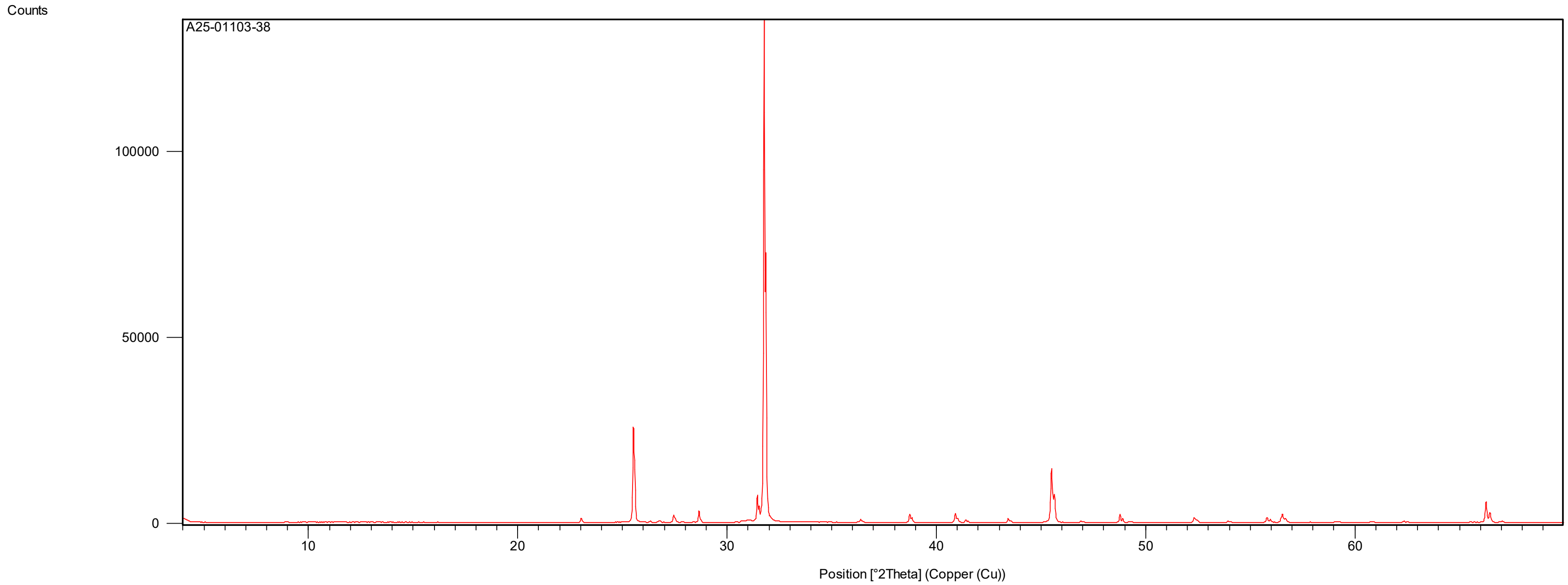
Counts
A25-01103-38
100000
50000
0
10
20
30
40
50
60
Position [°2Theta] (Copper (Cu))

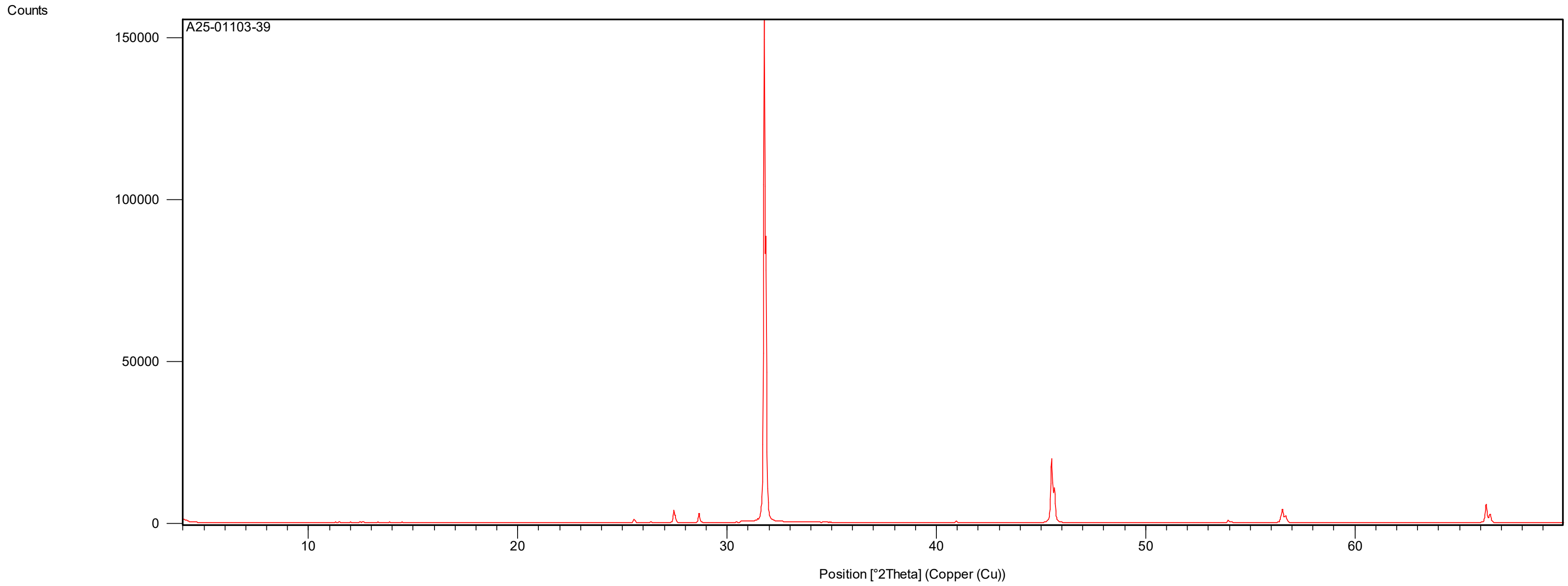
Counts
A25-01103-39
150000
100000
50000
0
10
20
30
40
50
60
Position [°2Theta] (Copper (Cu))

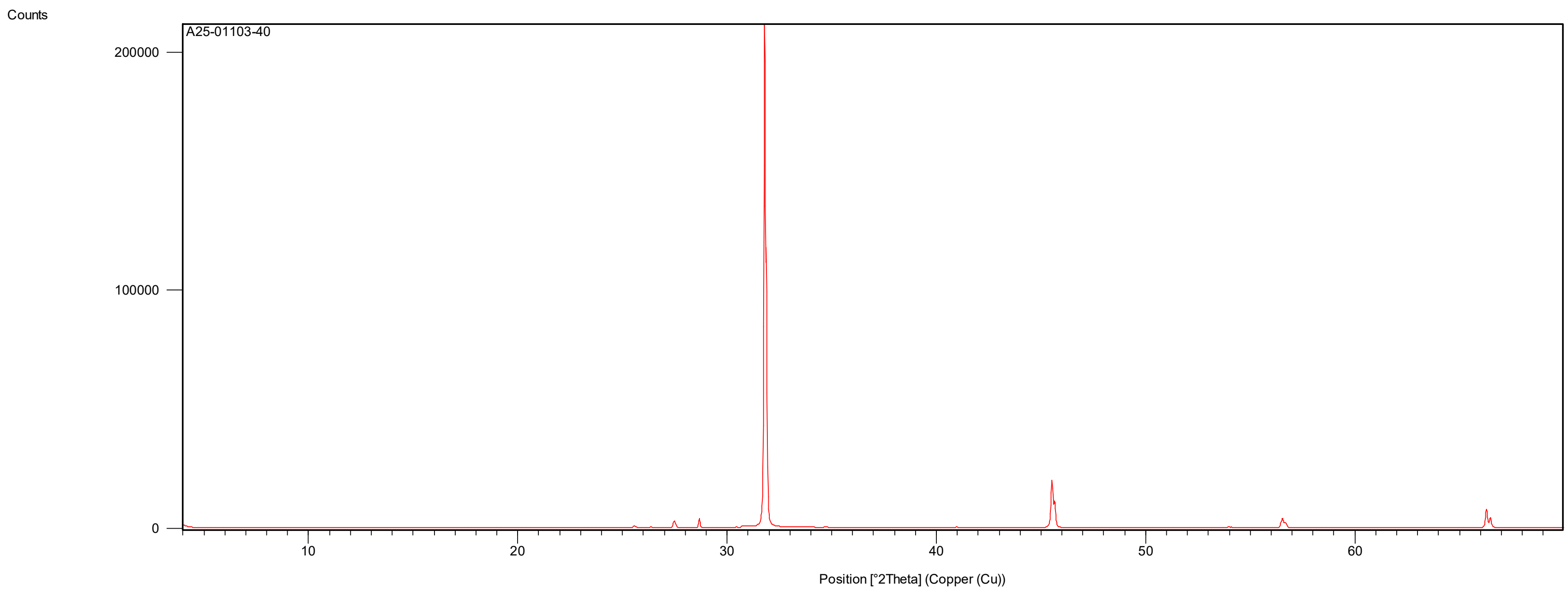
Counts
A25-01103-40
200000
100000
0
10
20
30
40
50
60
Position [°2Theta] (Copper (Cu))

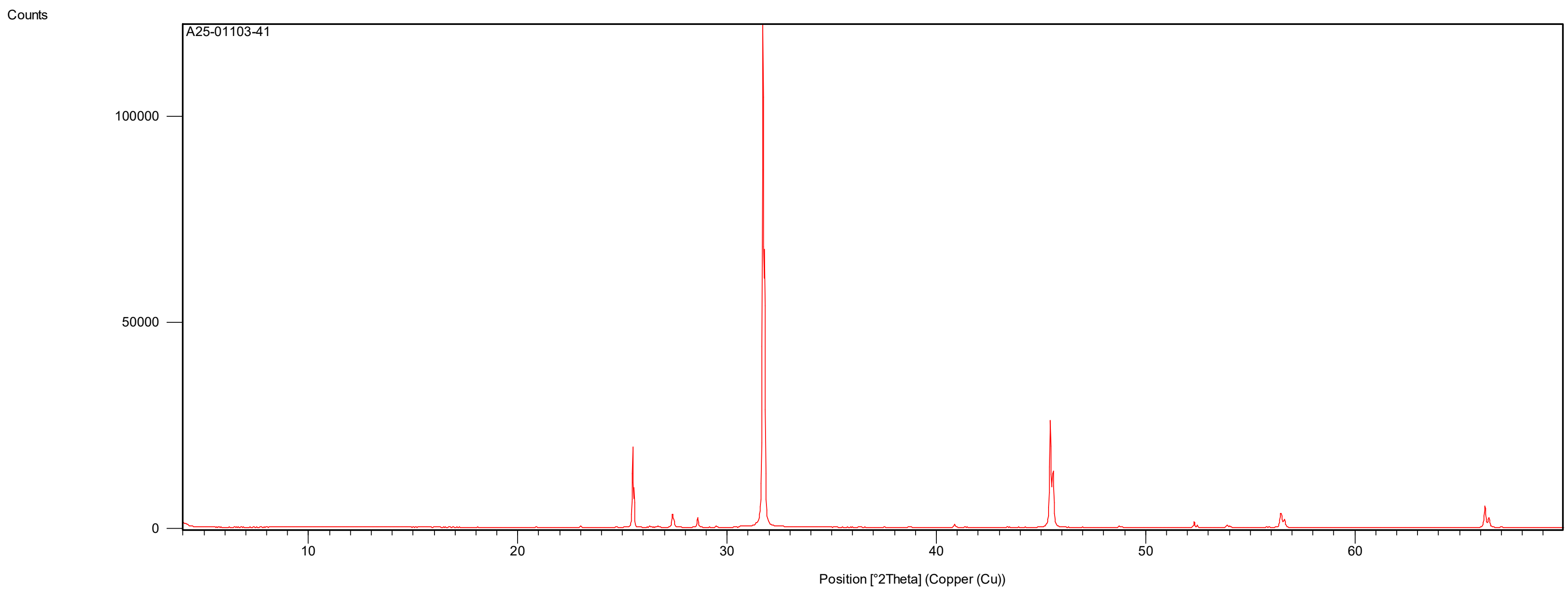
Counts
A25-01103-41
100000
50000
0
10
20
30
40
50
60
Position [°2Theta] (Copper (Cu))

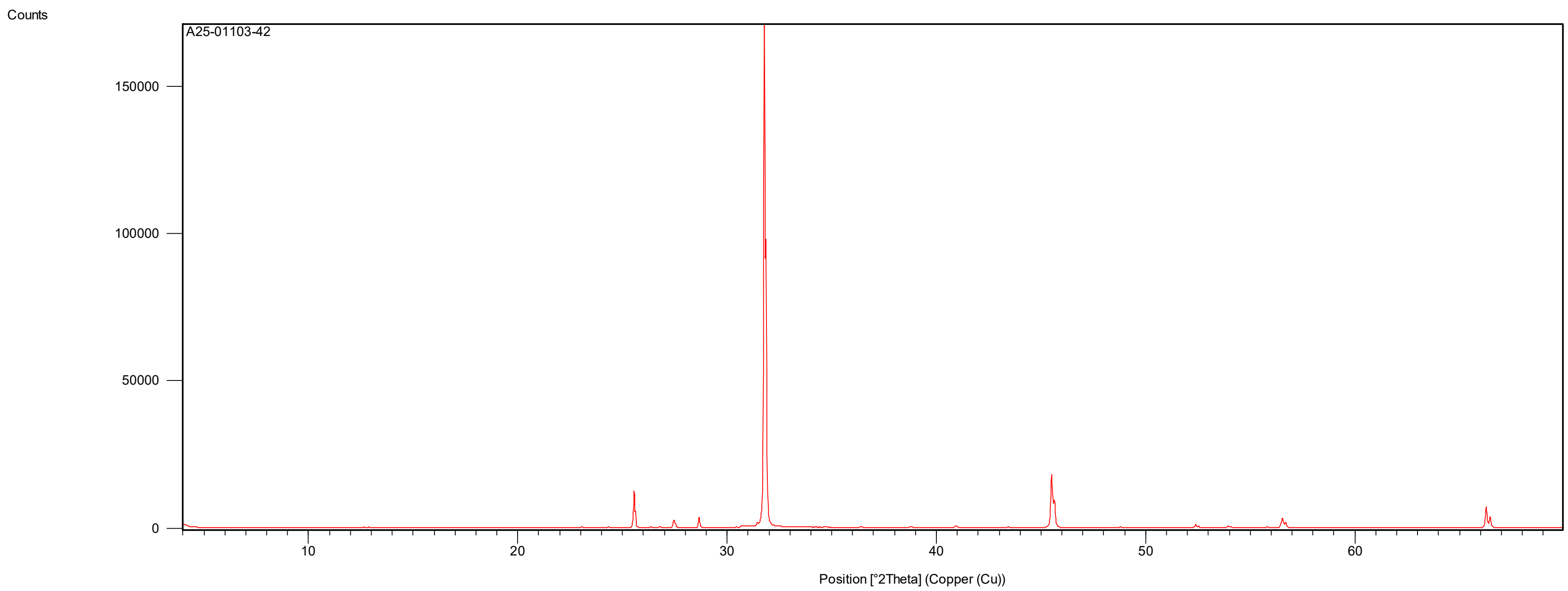
Counts
A25-01103-42
150000
100000
50000
0
10
20
30
40
50
60
Position [°2Theta] (Copper (Cu))

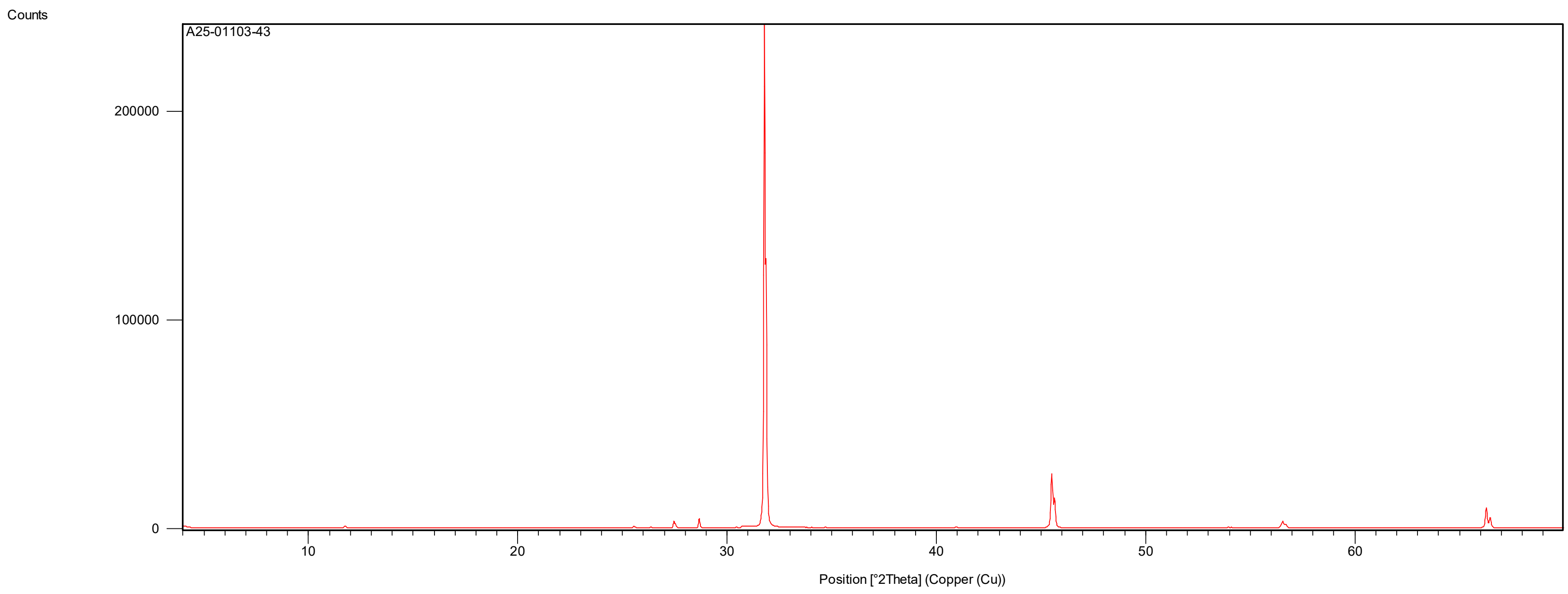
Counts
A25-01103-43
200000
100000
0
10
20
30
40
50
60
Position [°2Theta] (Copper (Cu))

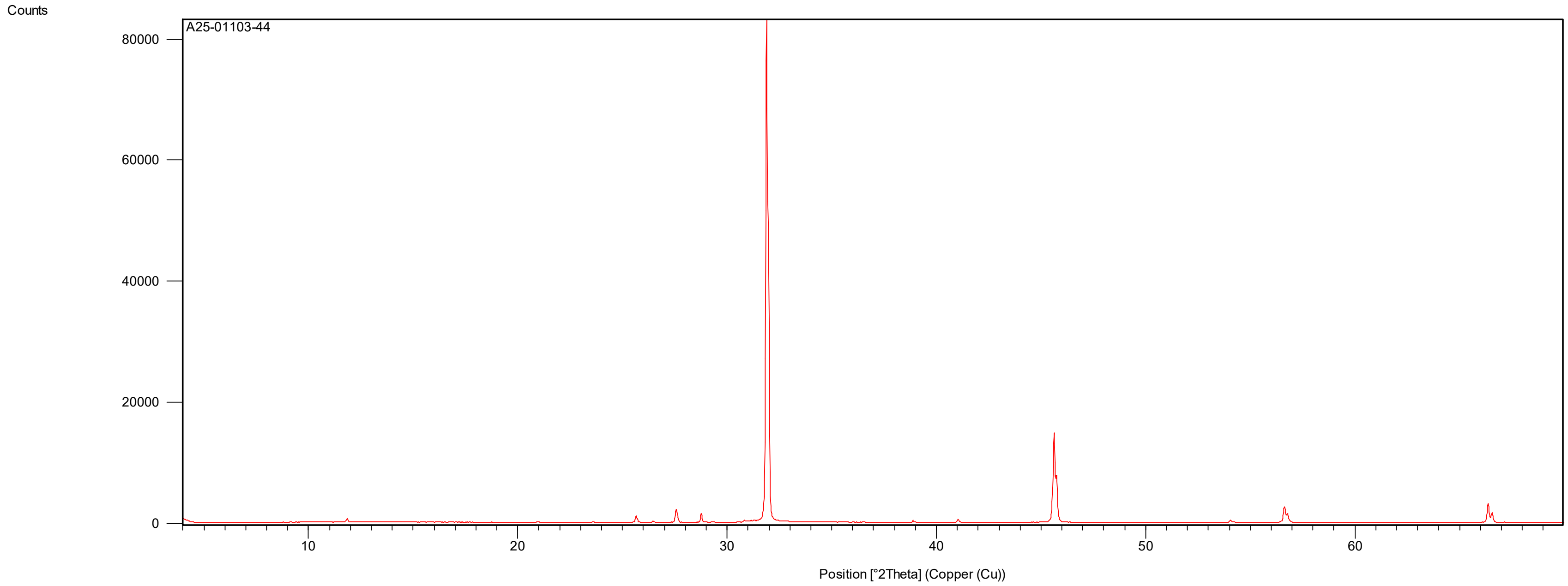
Counts
A25-01103-44
80000
60000
40000
20000
0
10
20
30
40
50
60
Position [°2Theta] (Copper (Cu))

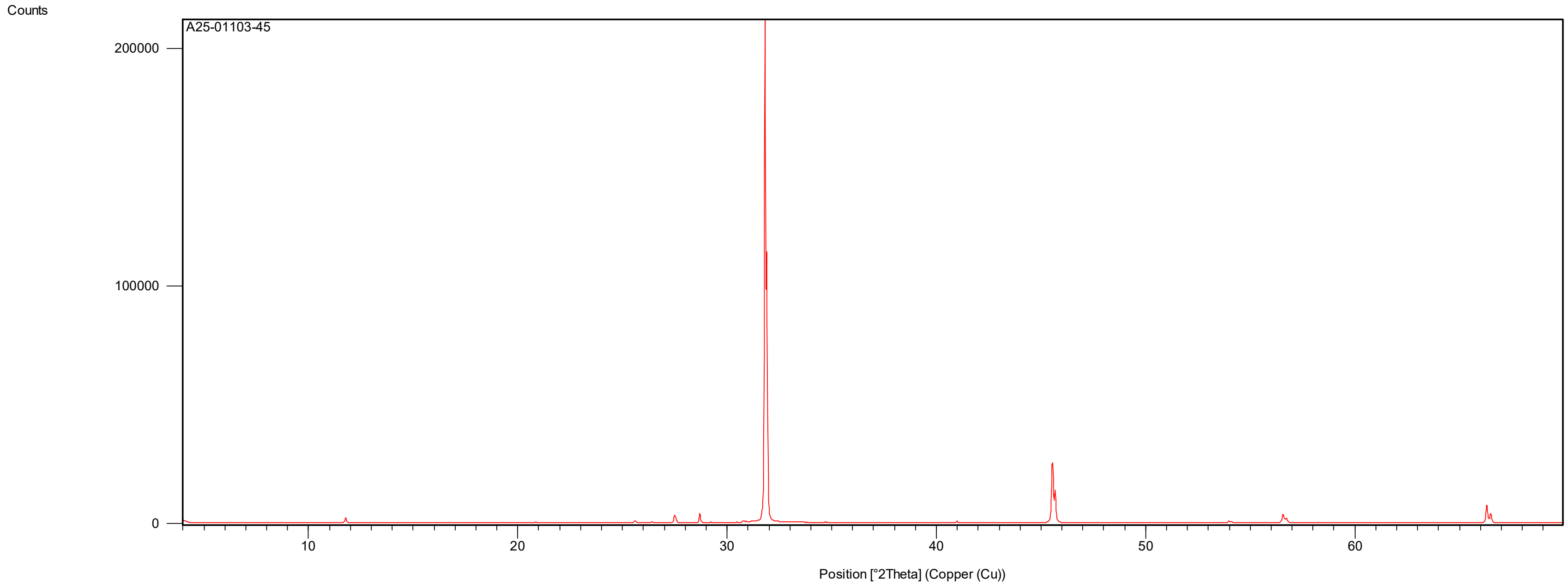
Counts
A25-01103-45
200000
100000
0
10
20
30
40
50
60
Position [°2Theta] (Copper (Cu))

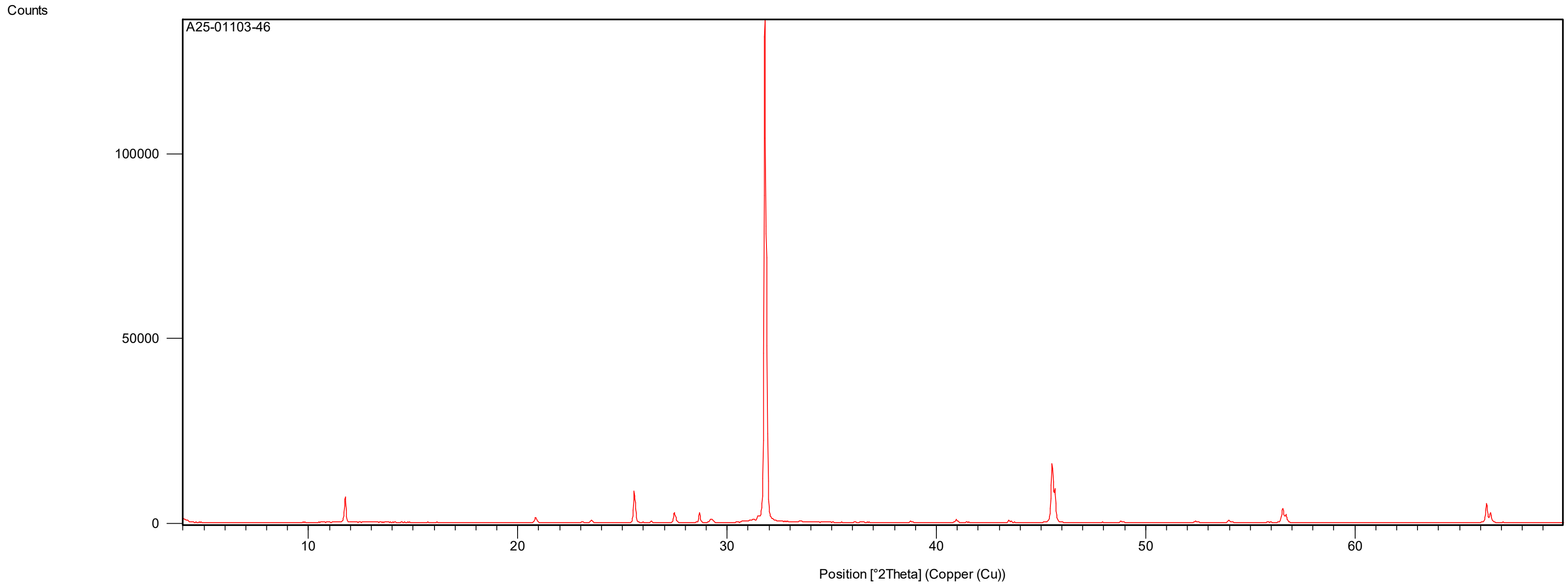
Counts
A25-01103-46
100000
50000
0
10
20
30
40
50
60
Position [°2Theta] (Copper (Cu))

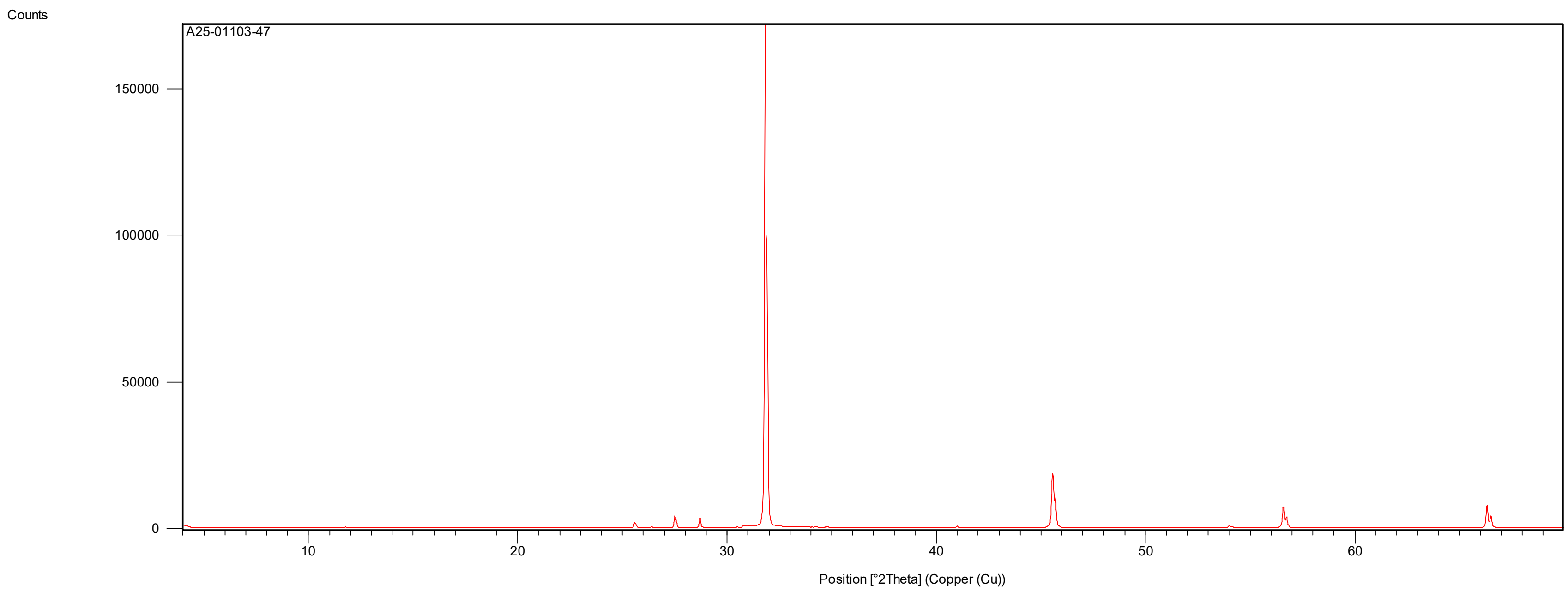
Counts
A25-01103-47
150000
100000
50000
0
10
20
30
40
50
60
Position [°2Theta] (Copper (Cu))

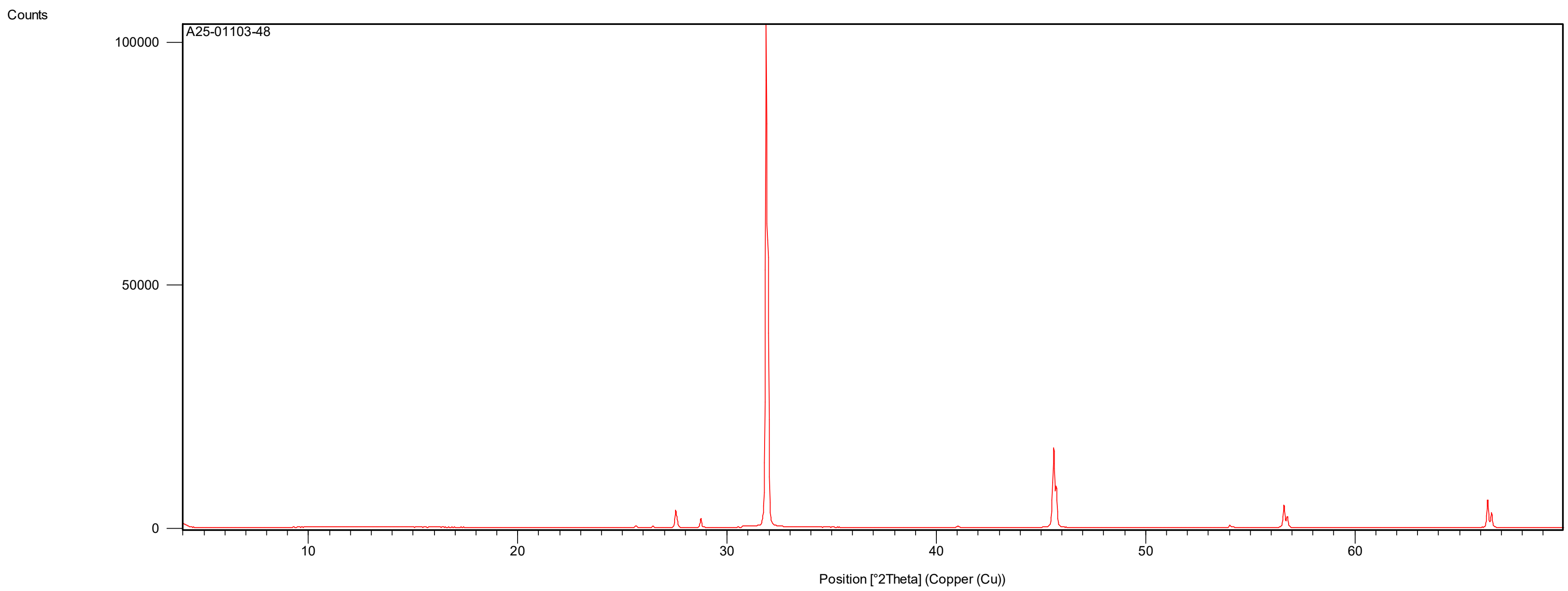
Counts
A25-01103-48
100000
50000
0
10
20
30
40
50
60
Position [°2Theta] (Copper (Cu))

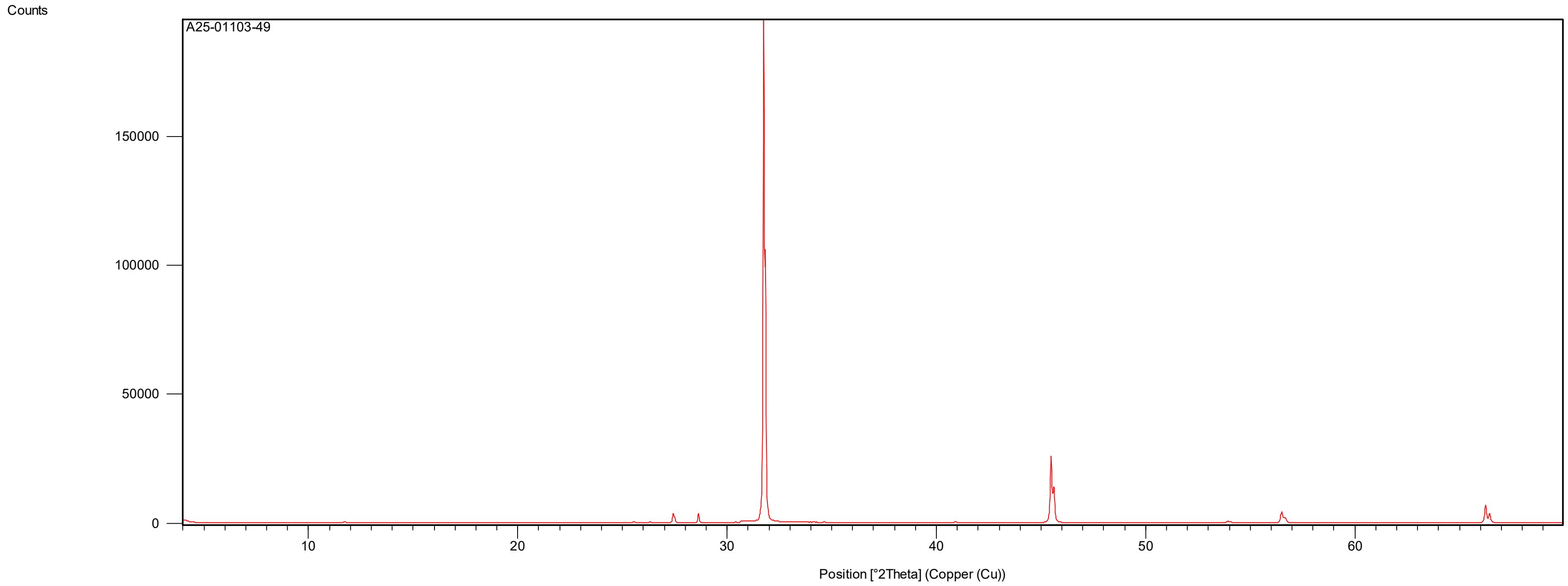
Counts
A25-01103-49
150000
100000
50000
0
10
20
30
40
50
60
Position [°2Theta] (Copper (Cu))

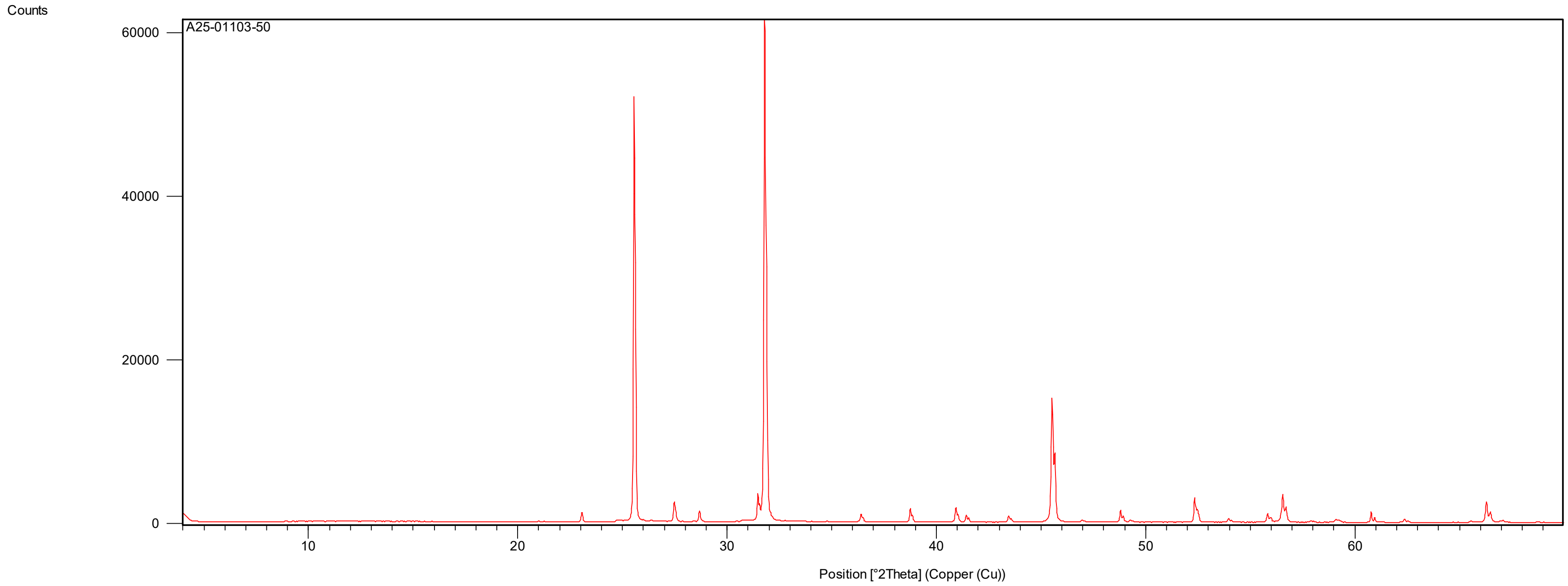
Counts
A25-01103-50
60000
40000
20000
0
10
20
30
40
50
60
Position [°2Theta] (Copper (Cu))

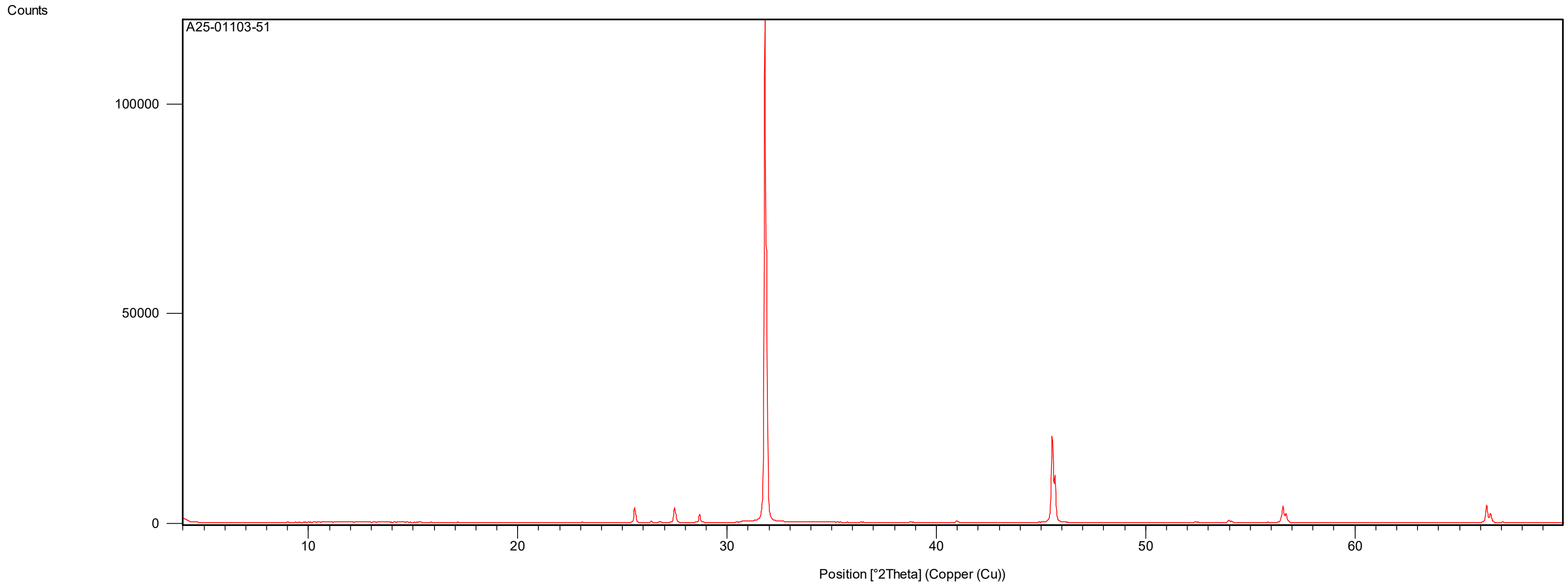
Counts
A25-01103-51
100000
50000
0
10
20
30
40
50
60
Position [°2Theta] (Copper (Cu))

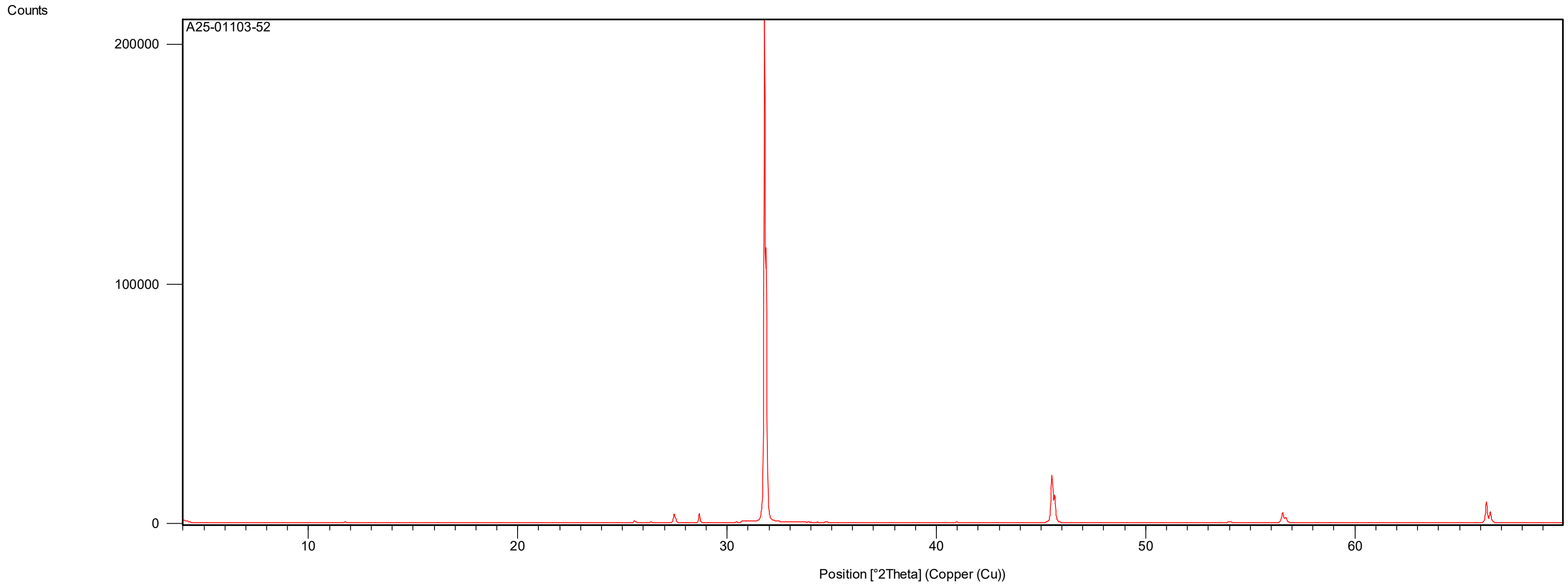
Counts
A25-01103-52
200000
100000
0
10
20
30
40
50
60
Position [°2Theta] (Copper (Cu))

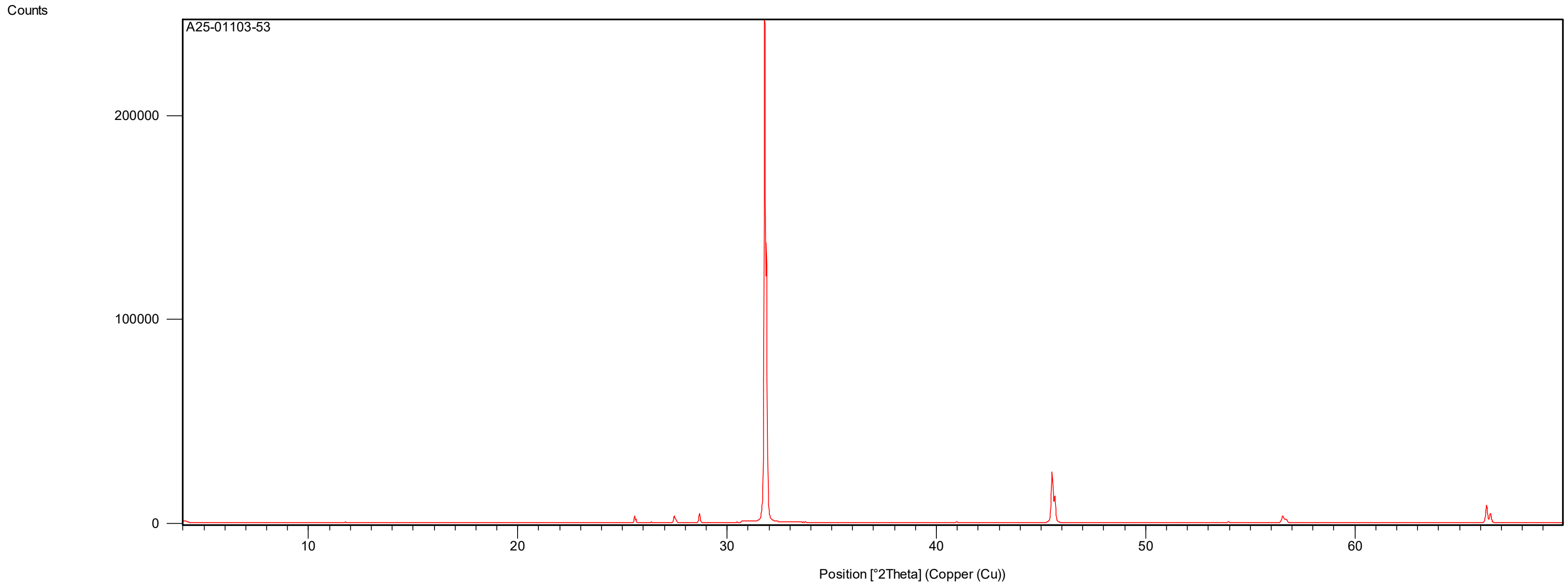
Counts
A25-01103-53
200000
100000
0
10
20
30
40
50
60
Position [°2Theta] (Copper (Cu))

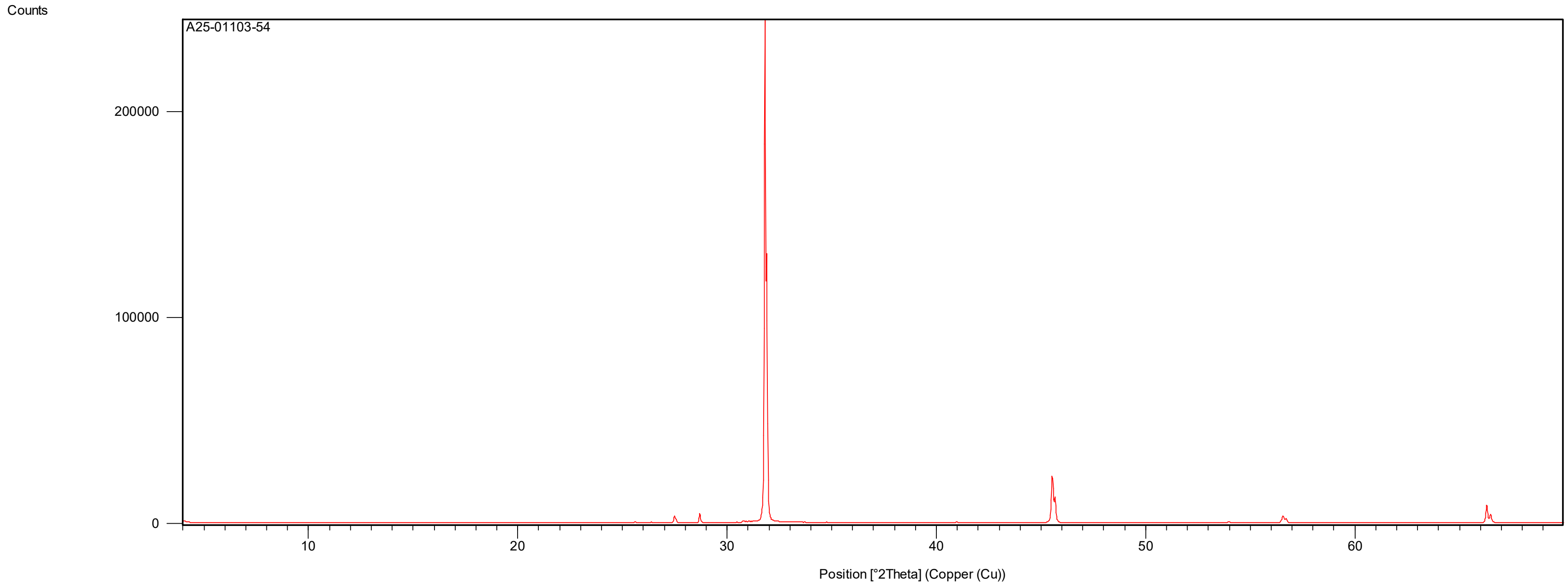
Counts
A25-01103-54
200000
100000
0
10
20
30
40
50
60
Position [°2Theta] (Copper (Cu))

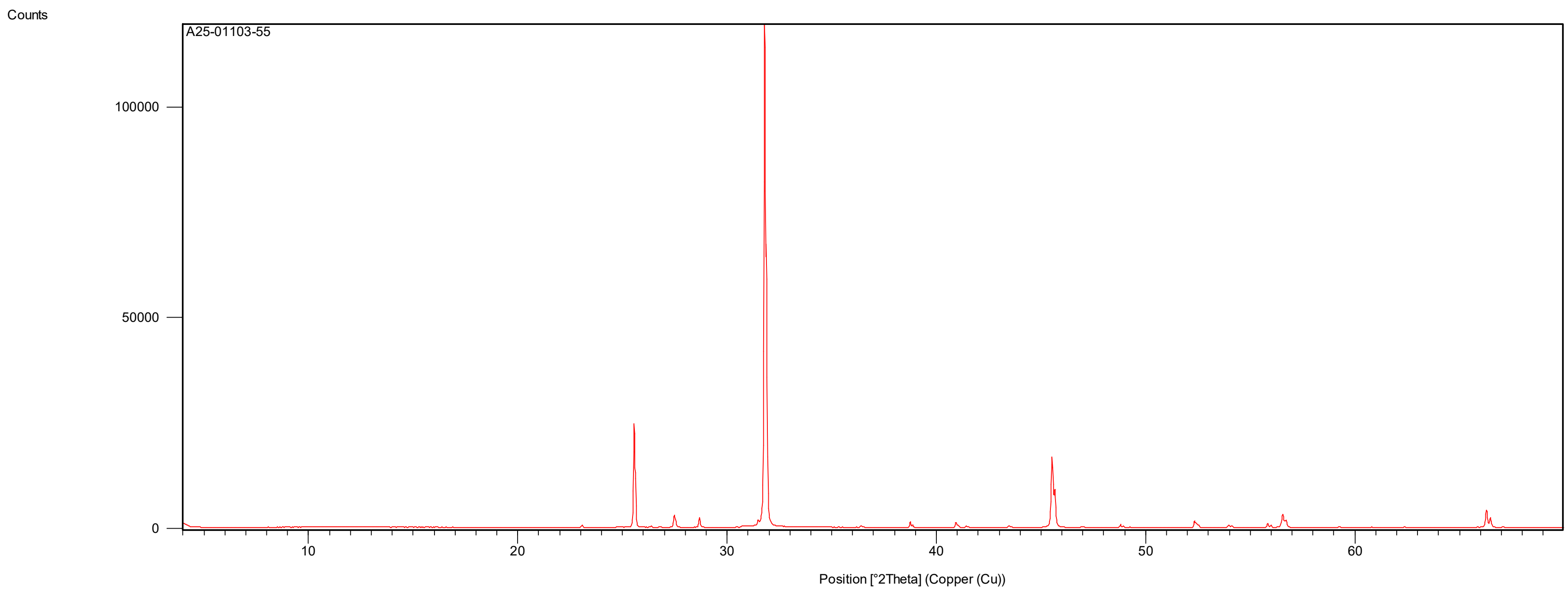
Counts
A25-01103-55
100000
50000
0
10
20
30
40
50
60
Position [°2Theta] (Copper (Cu))

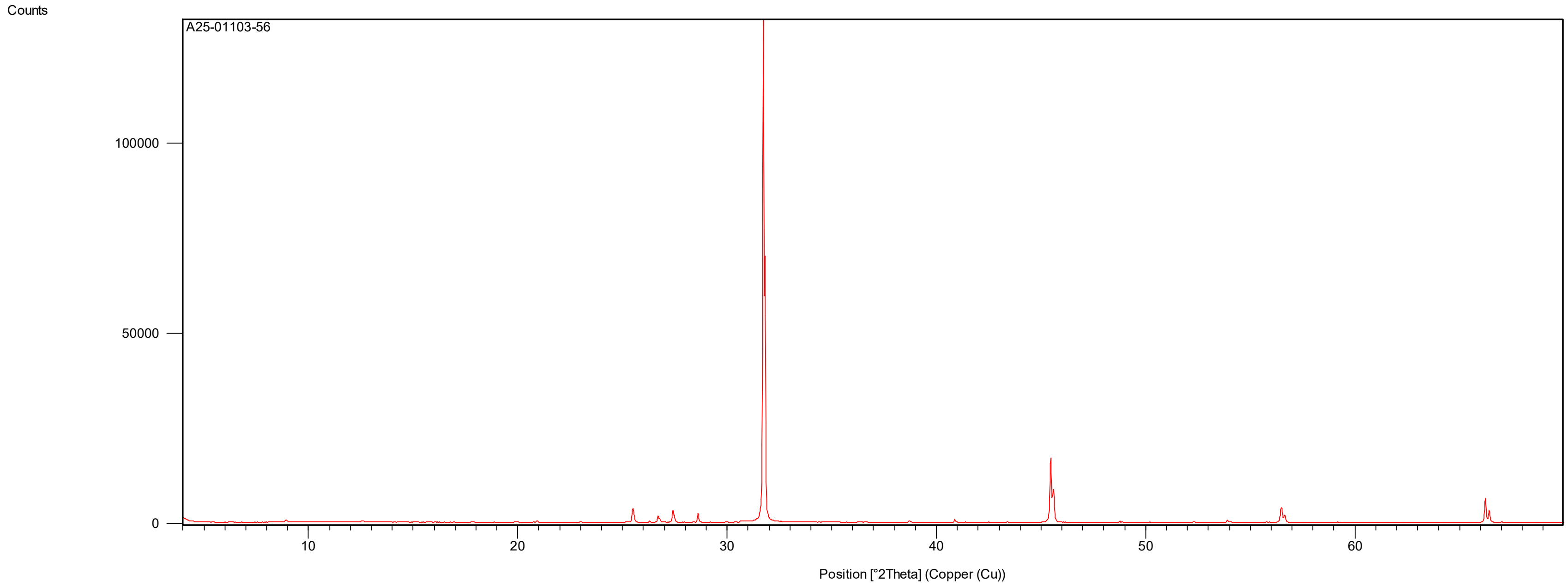
Counts
A25-01103-56
100000
50000
0
10
20
30
40
50
60
Position [°2Theta] (Copper (Cu))

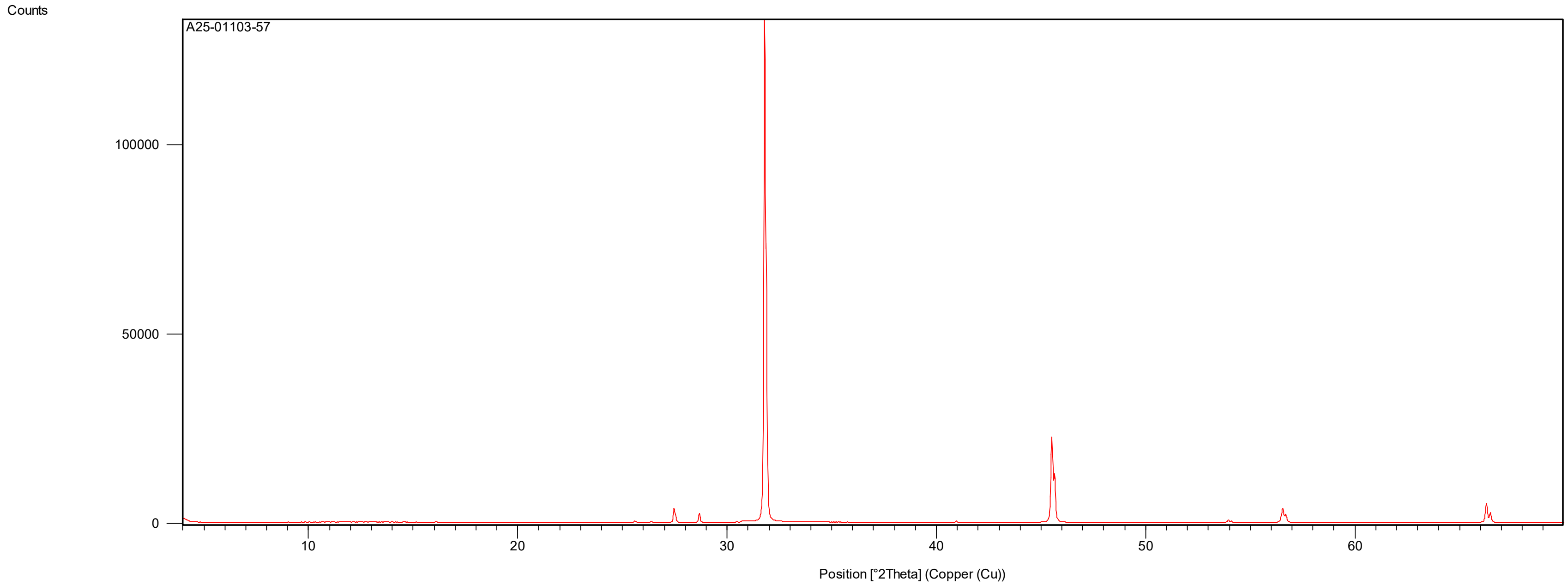
Counts
A25-01103-57
100000
50000
0
10
20
30
40
50
60
Position [°2Theta] (Copper (Cu))

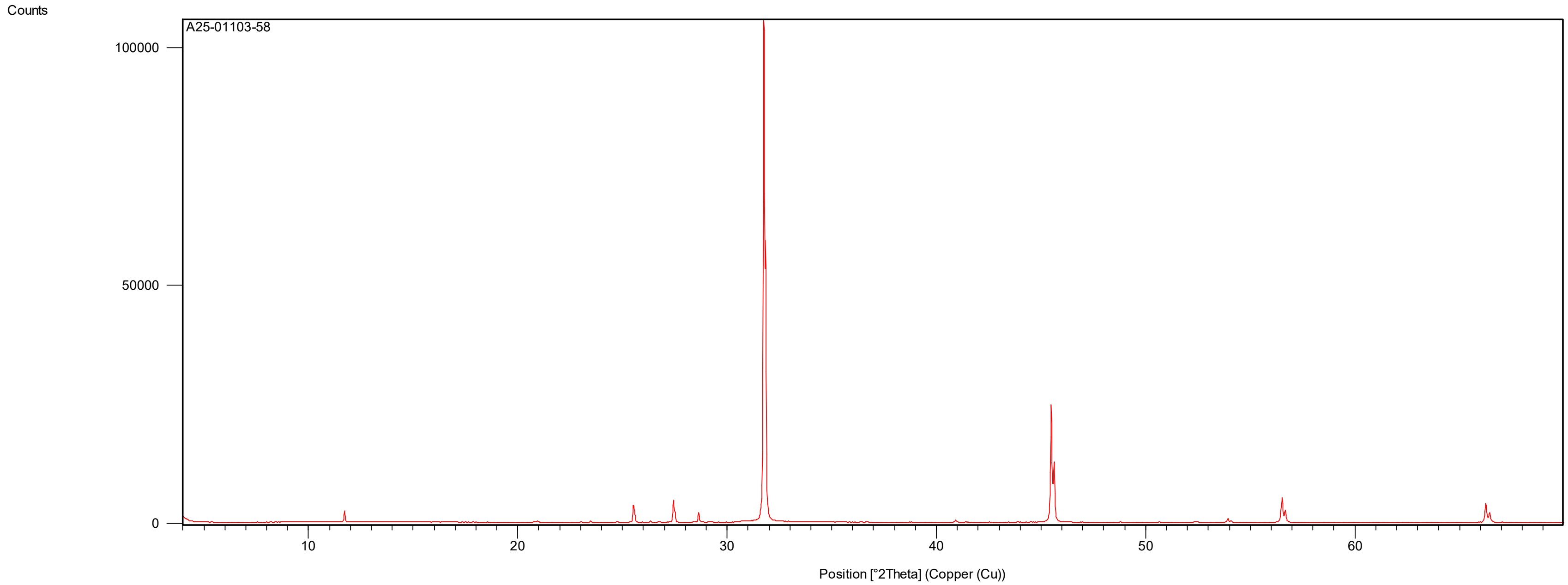
Counts
A25-01103-58
100000
50000
0
10
20
30
40
50
60
Position [°2Theta] (Copper (Cu))

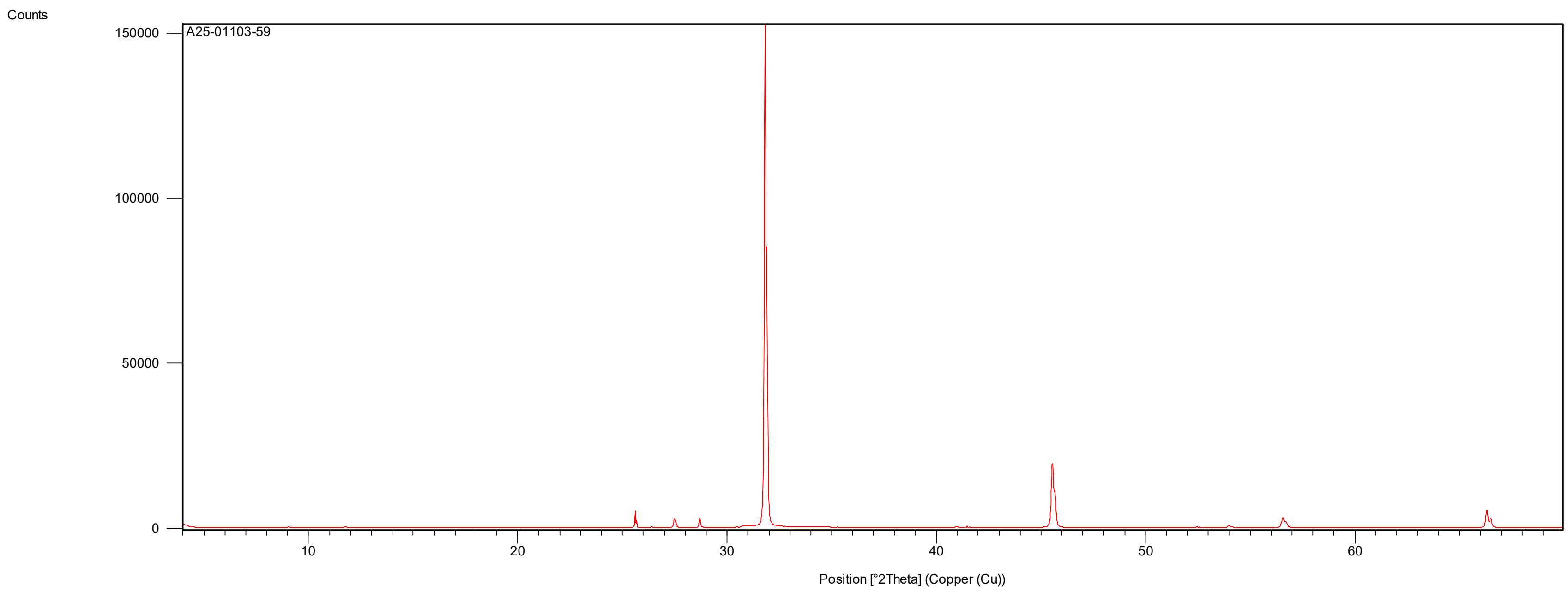
Counts
150000
100000
50000
0
A25-01103-59
10
20
30
40
50
60
Position [°2Theta] (Copper (Cu))

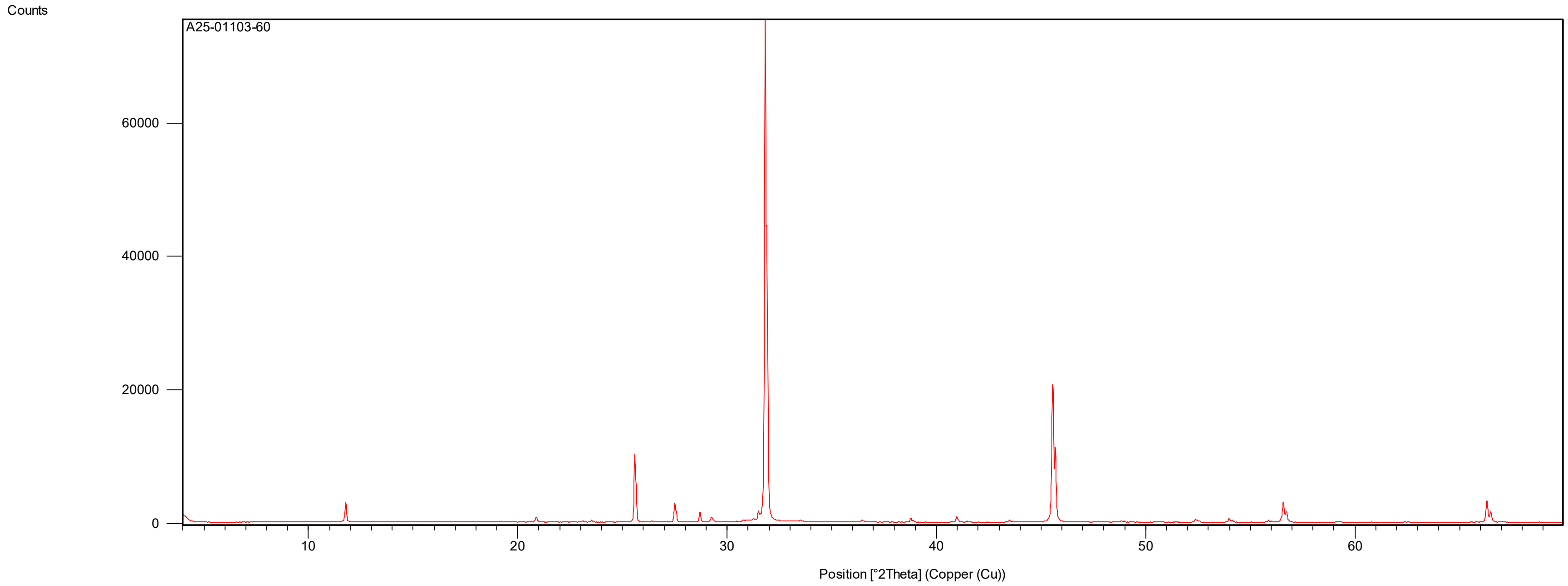
Counts
A25-01103-60
60000
40000
20000
0
10
20
30
40
50
60
Position [°2Theta] (Copper (Cu))

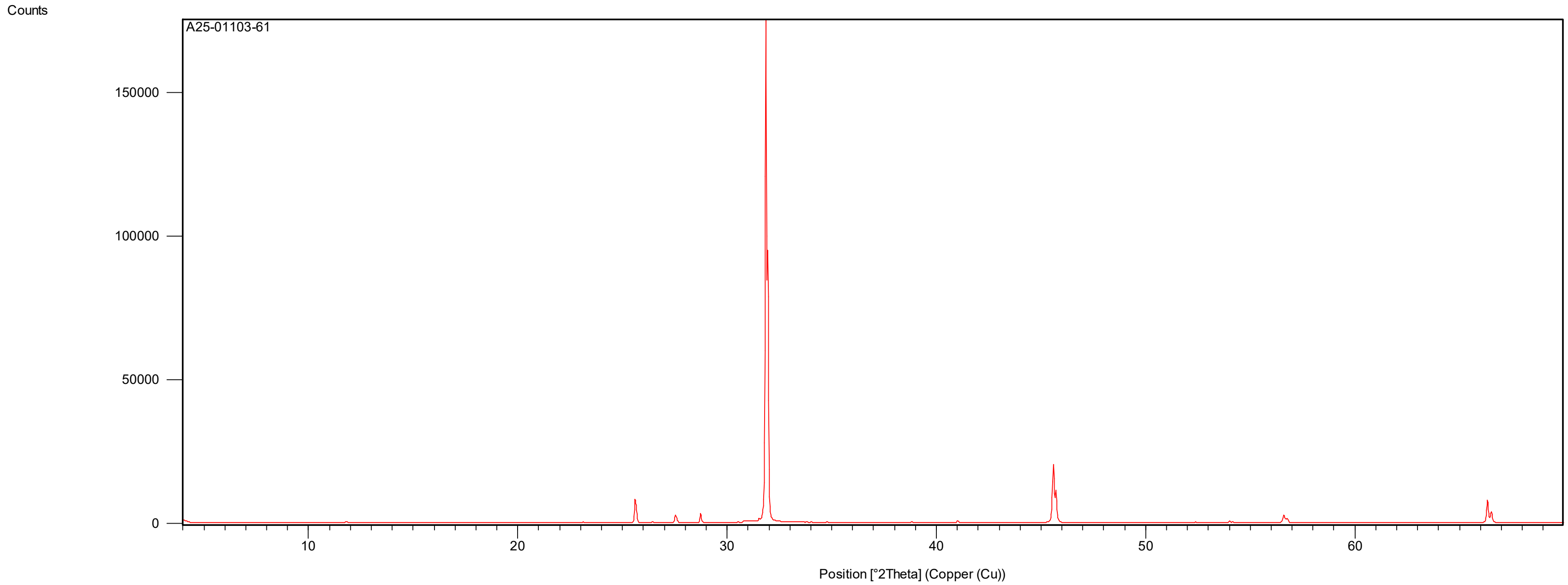
Counts
A25-01103-61
150000
100000
50000
0
10
20
30
40
50
60
Position [°2Theta] (Copper (Cu))

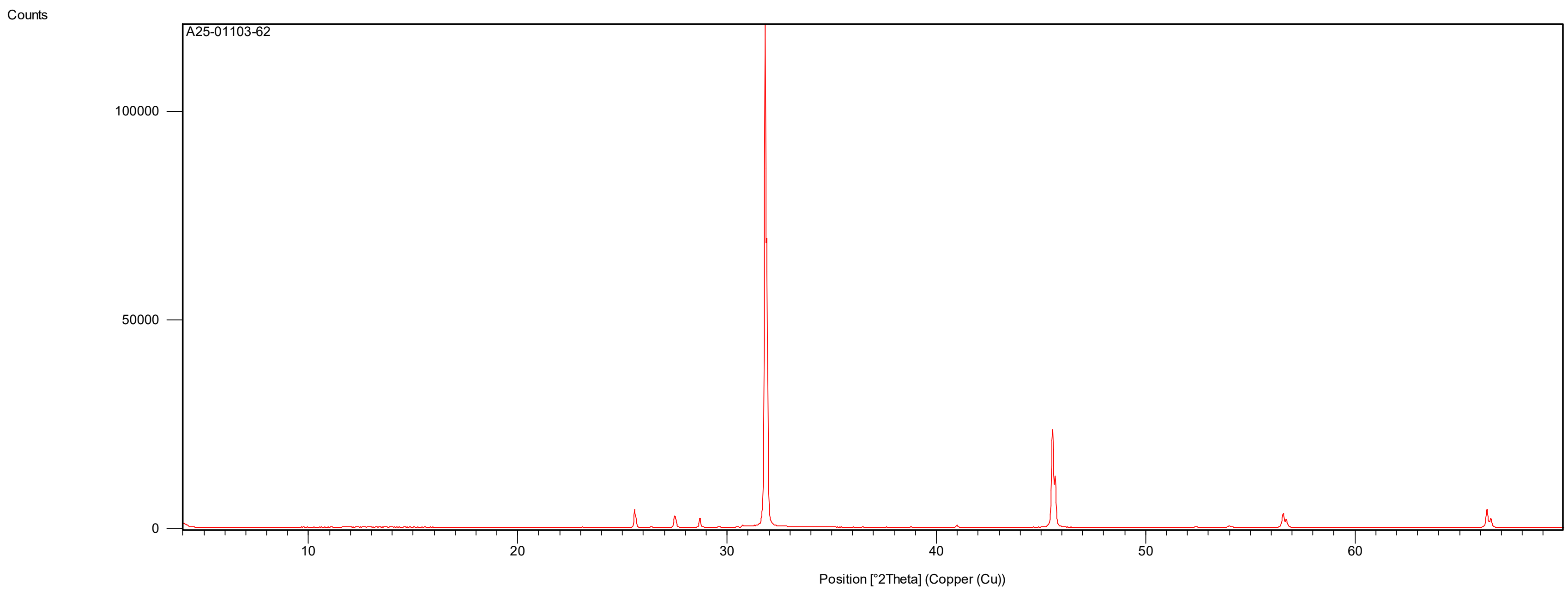
Counts
A25-01103-62
100000
50000
0
10
20
30
40
50
60
Position [°2Theta] (Copper (Cu))

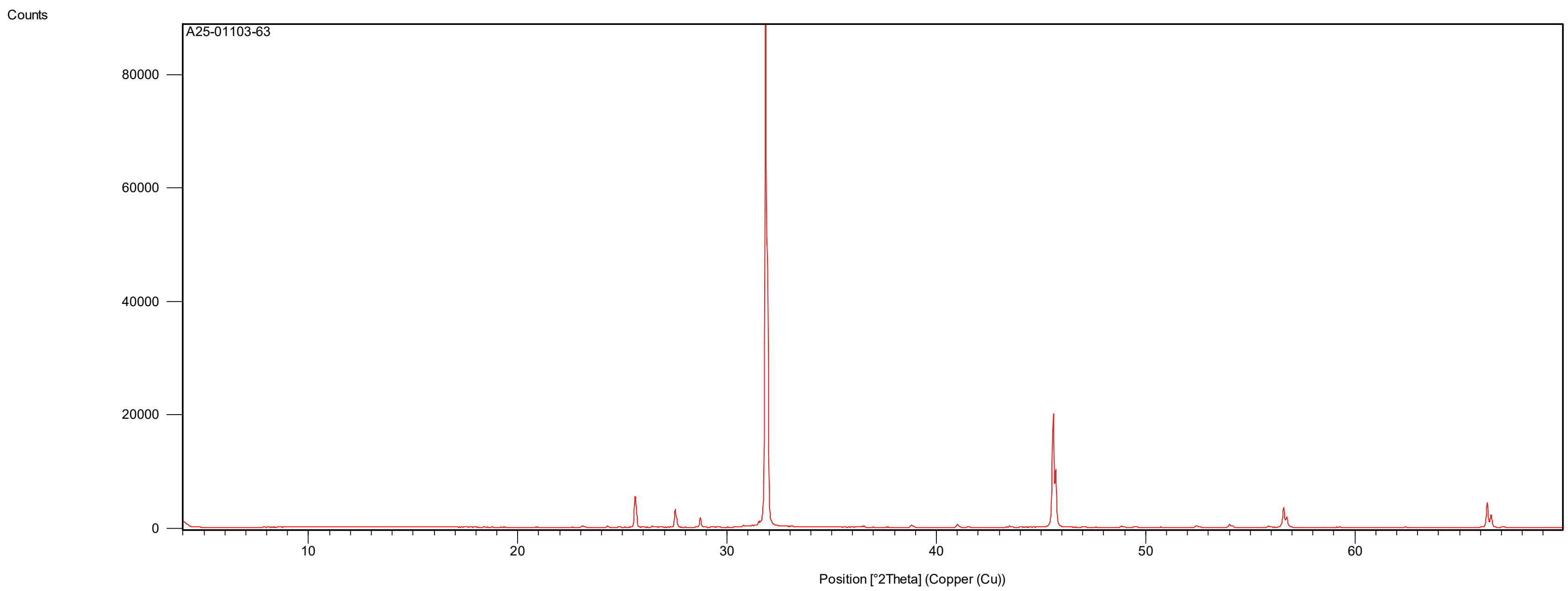
Counts
A25-01103-63
80000
60000
40000
20000
0
10
20
30
40
50
60
Position [°2Theta] (Copper (Cu))

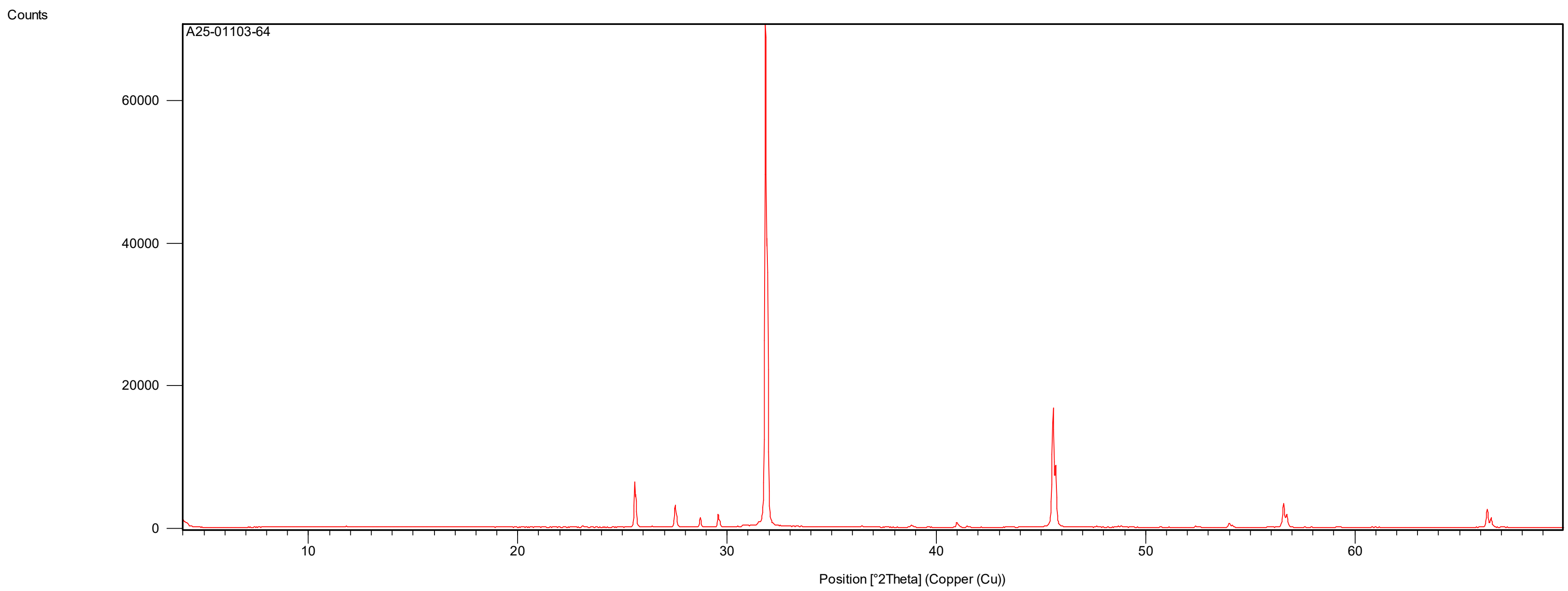
Counts
A25-01103-64
60000
40000
20000
0
10
20
30
40
50
60
Position [°2Theta] (Copper (Cu))

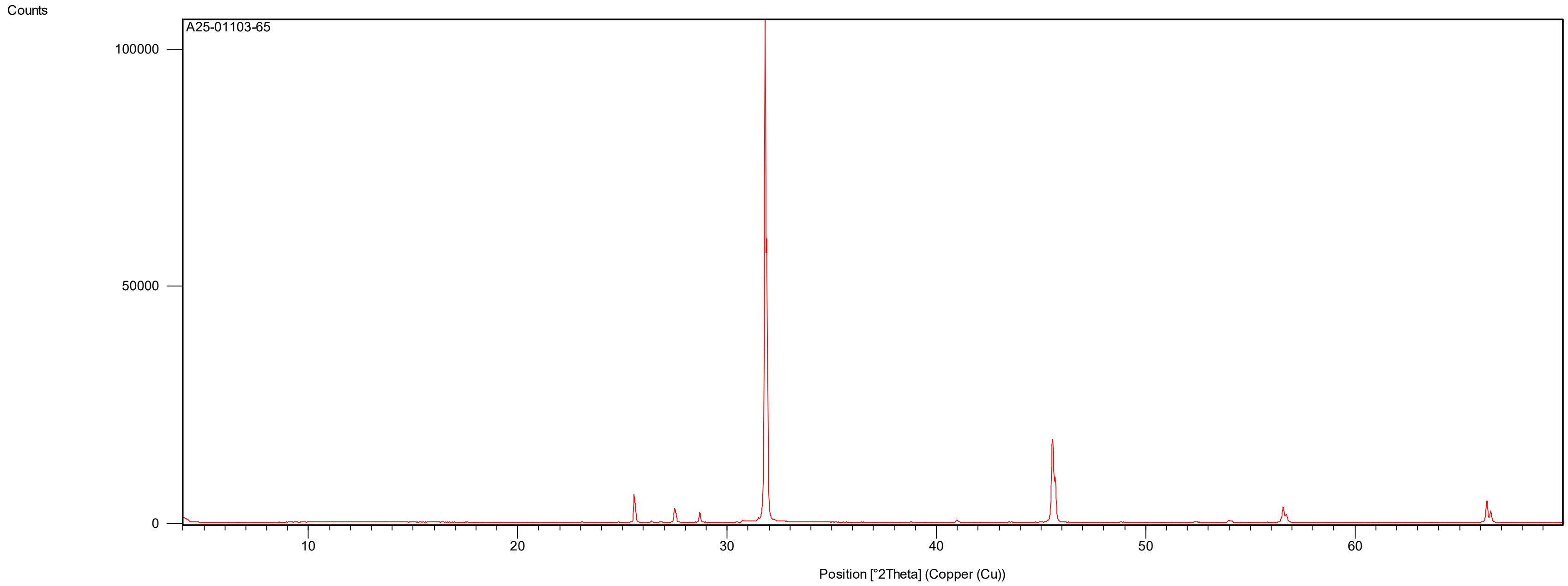
Counts
A25-01103-65
100000
50000
0
10
20
30
40
50
60
Position [°2Theta] (Copper (Cu))

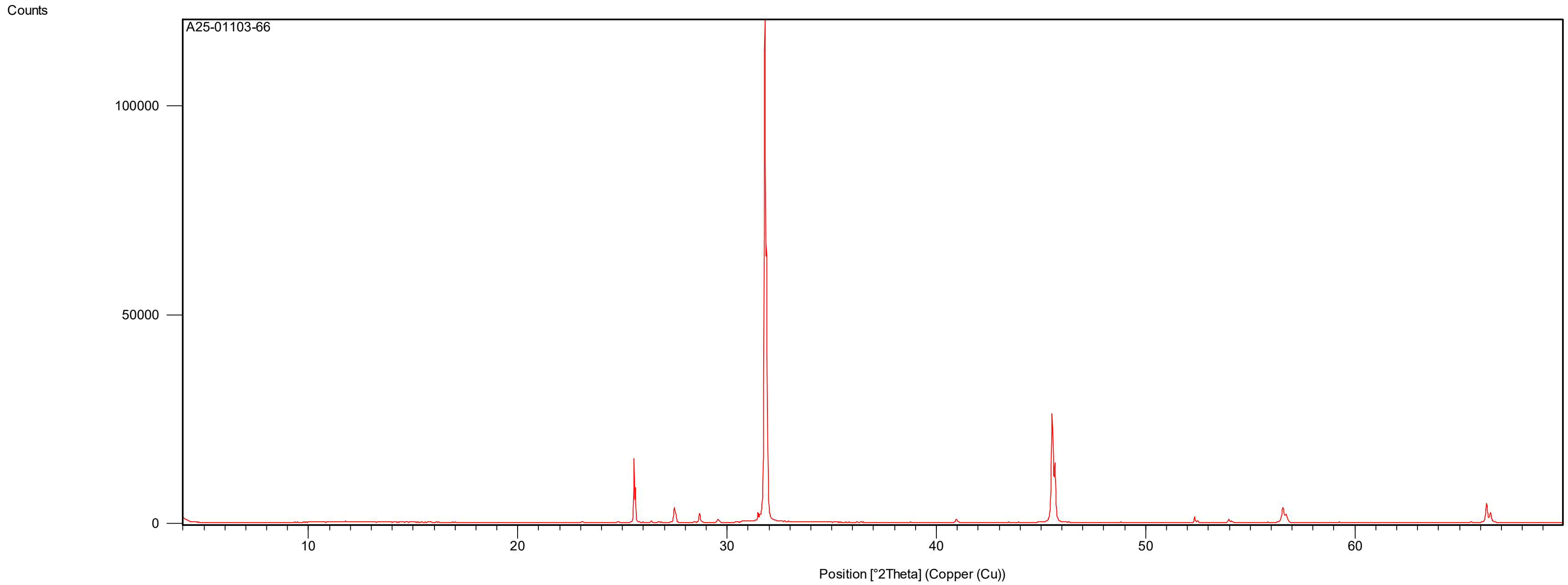
Counts
A25-01103-66
100000
50000
0
10
20
30
40
50
60
Position [°2Theta] (Copper (Cu))

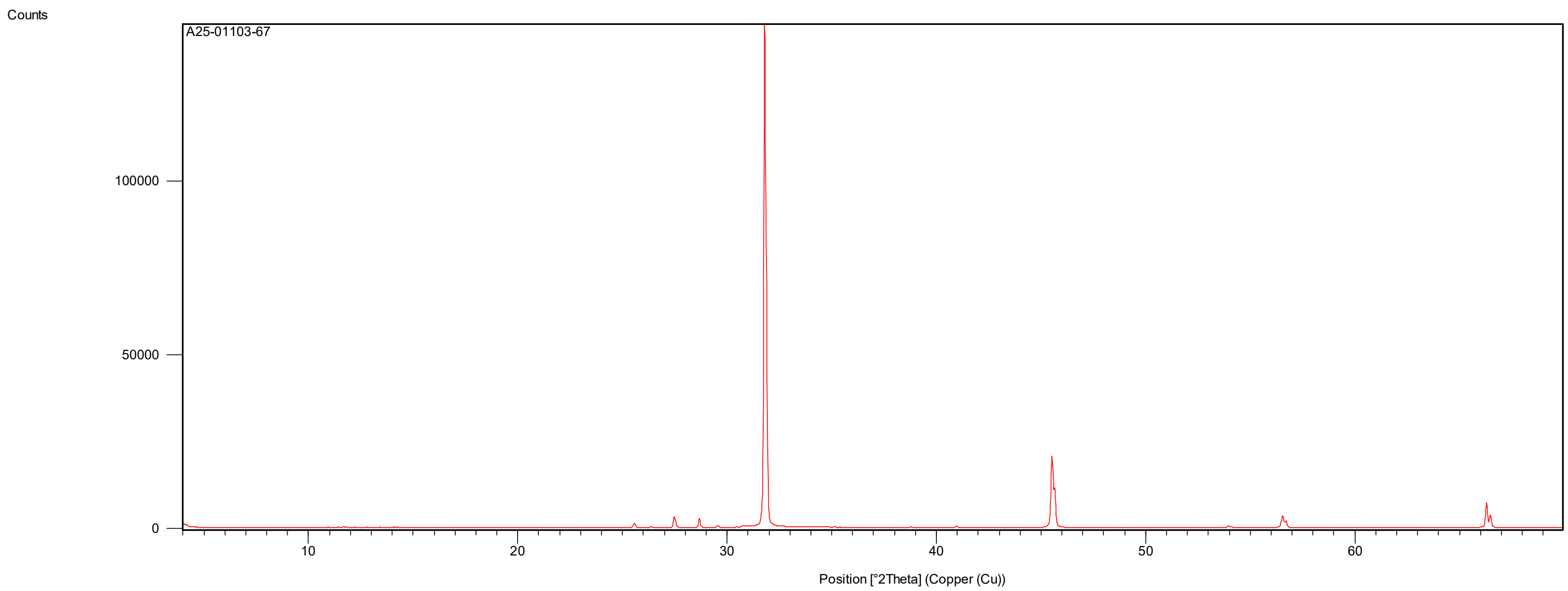
Counts
A25-01103-67
100000
50000
0
10
20
30
40
50
60
Position [°2Theta] (Copper (Cu))

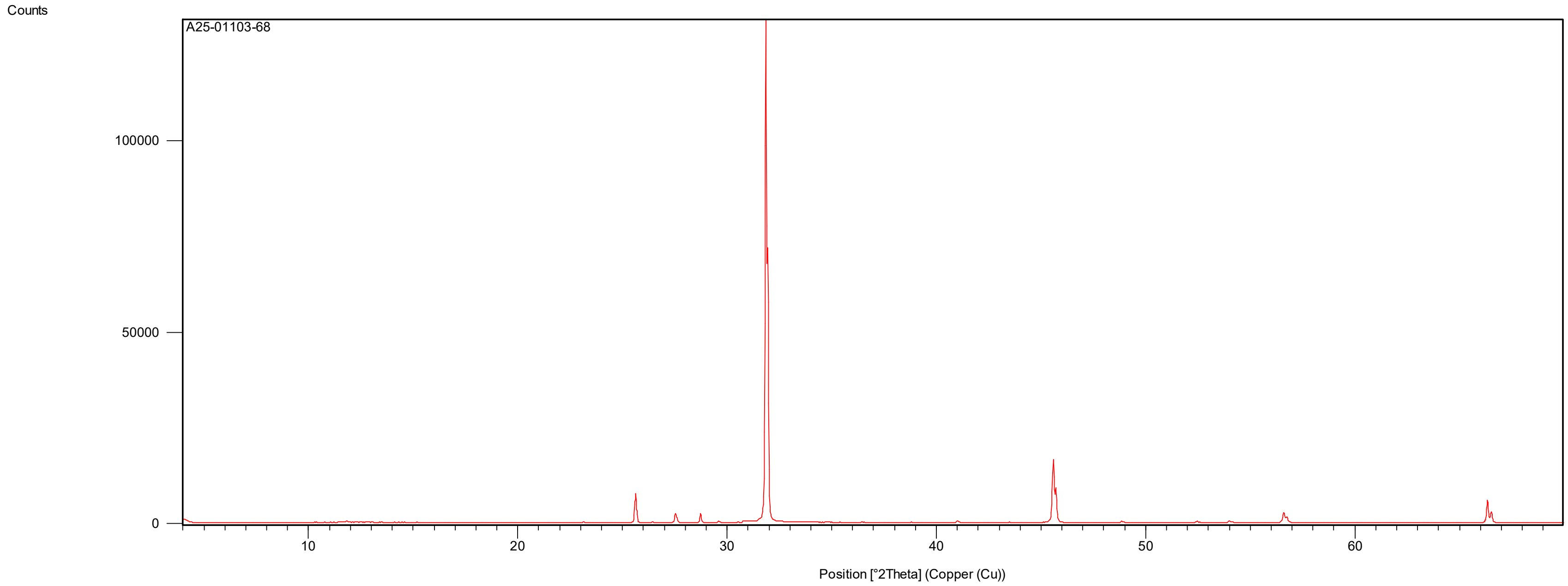
Counts
A25-01103-68
100000
50000
0
10
20
30
40
50
60
Position [°2Theta] (Copper (Cu))

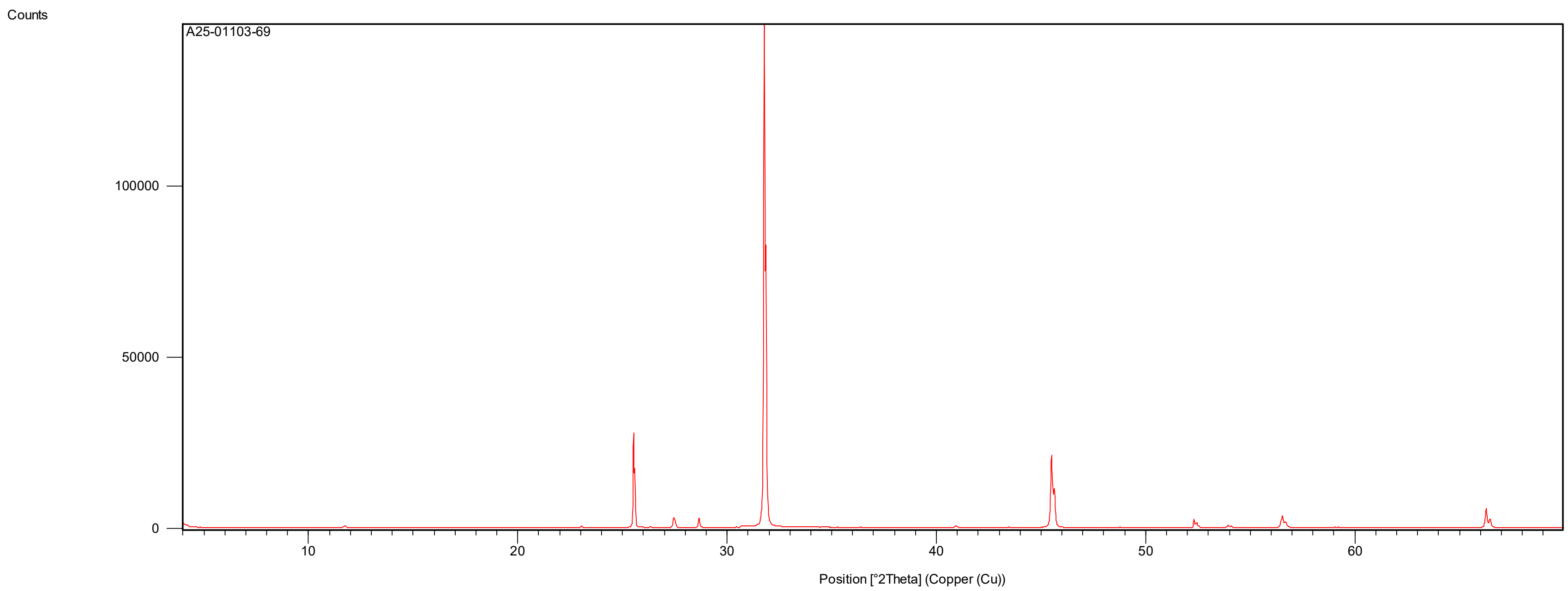

Counts
A25-01103-69
100000
50000
0
10
20
30
40
50
60
Position [°2Theta] (Copper (Cu))

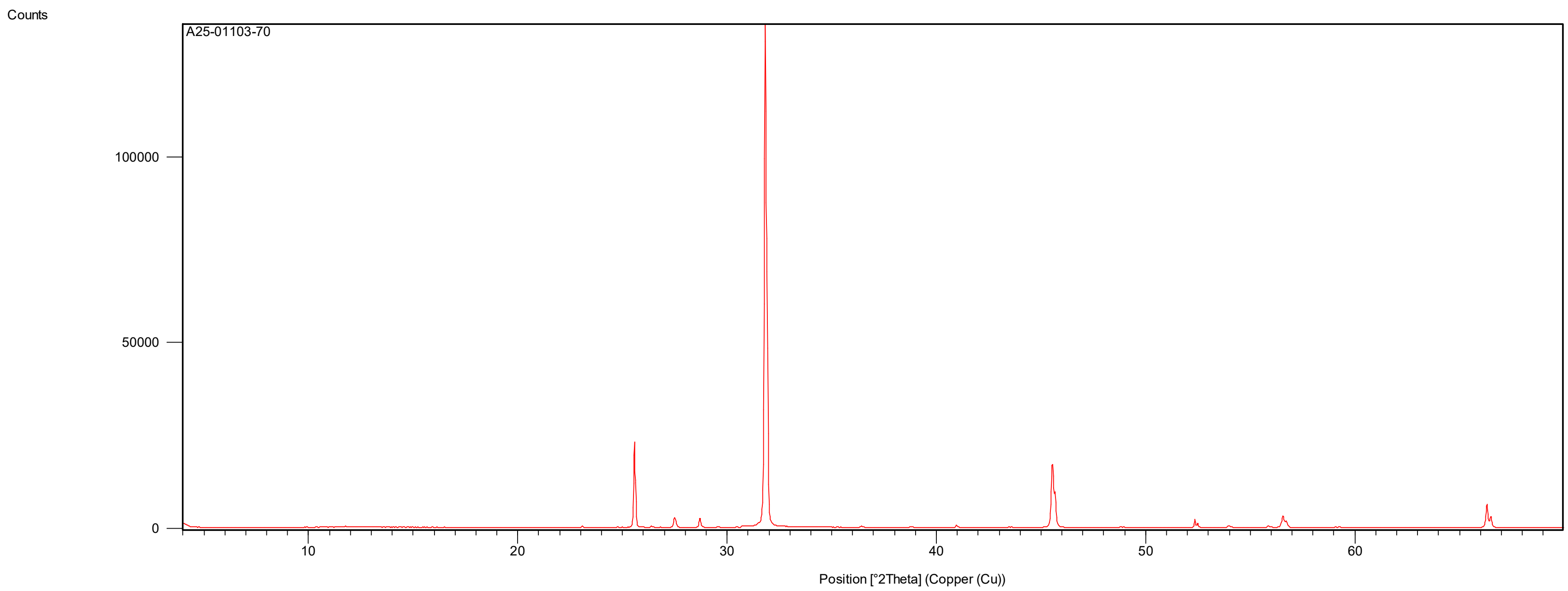
Counts
A25-01103-70
100000
50000
0
10
20
30
40
50
60
Position [°2Theta] (Copper (Cu))

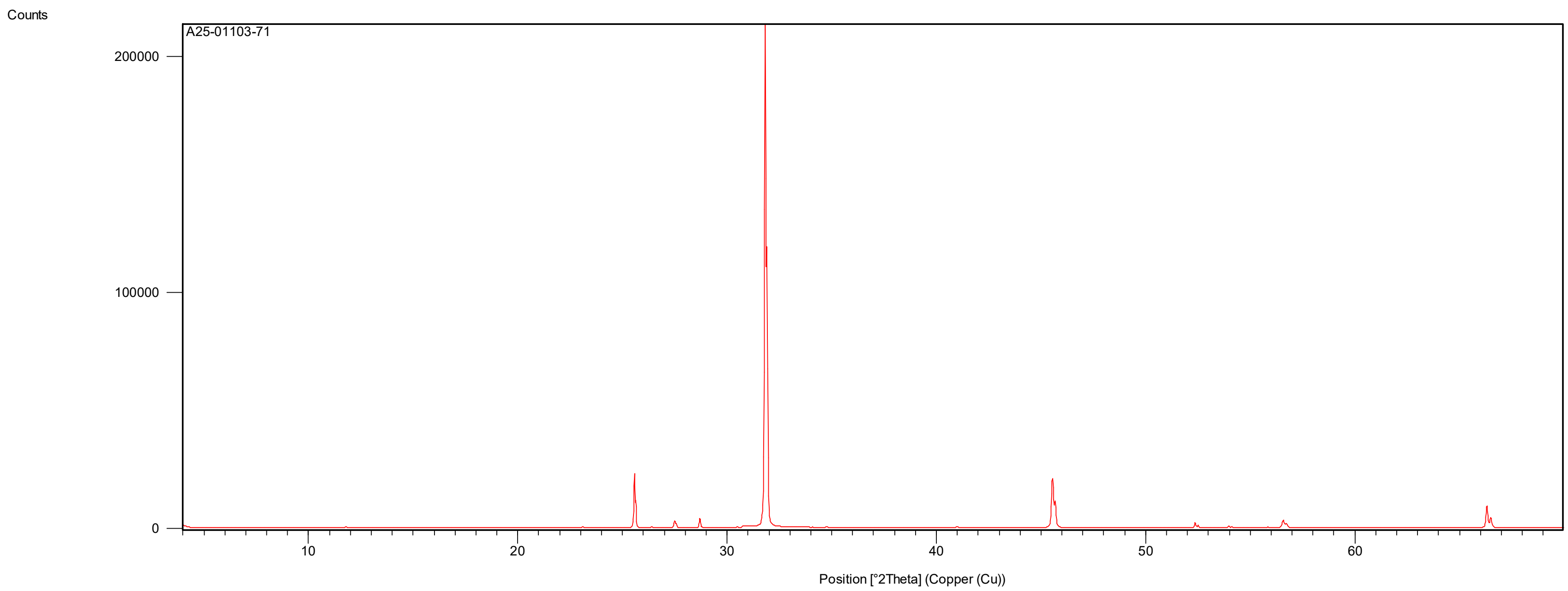
Counts
A25-01103-71
200000
100000
0
10
20
30
40
50
60
Position [°2Theta] (Copper (Cu))

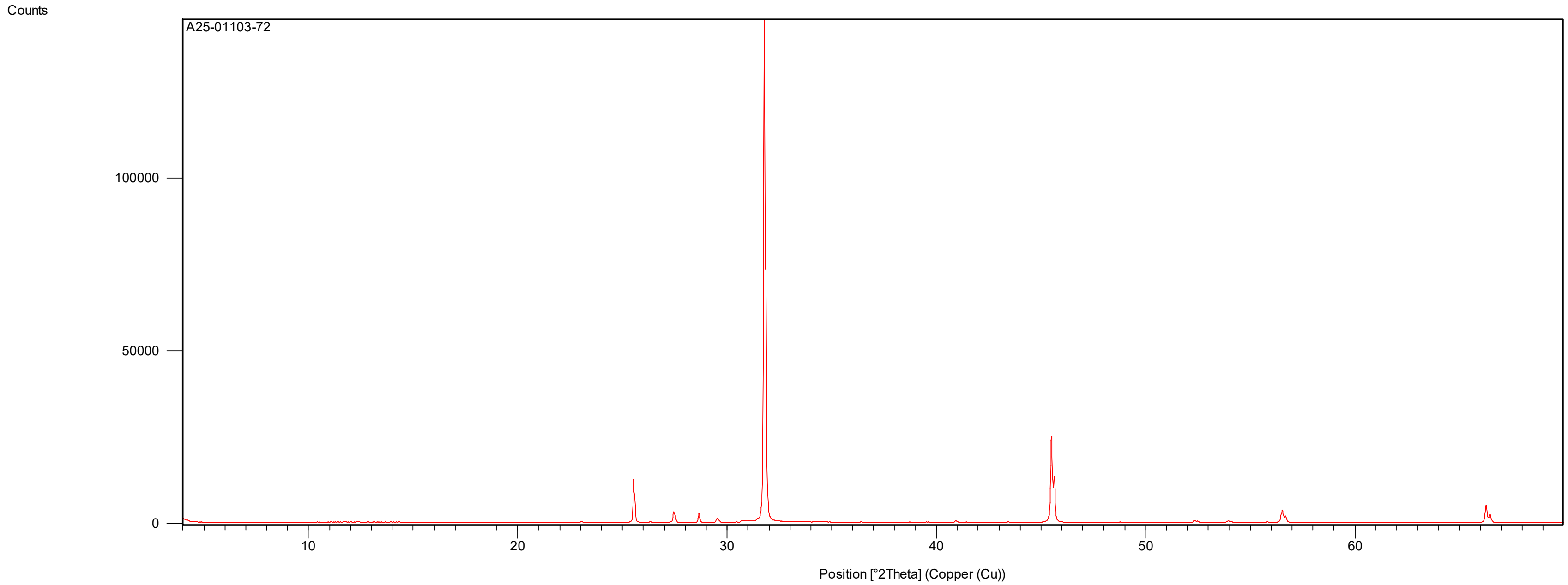
Counts
A25-01103-72
100000
50000
0
10
20
30
40
50
60
Position [°2Theta] (Copper (Cu))

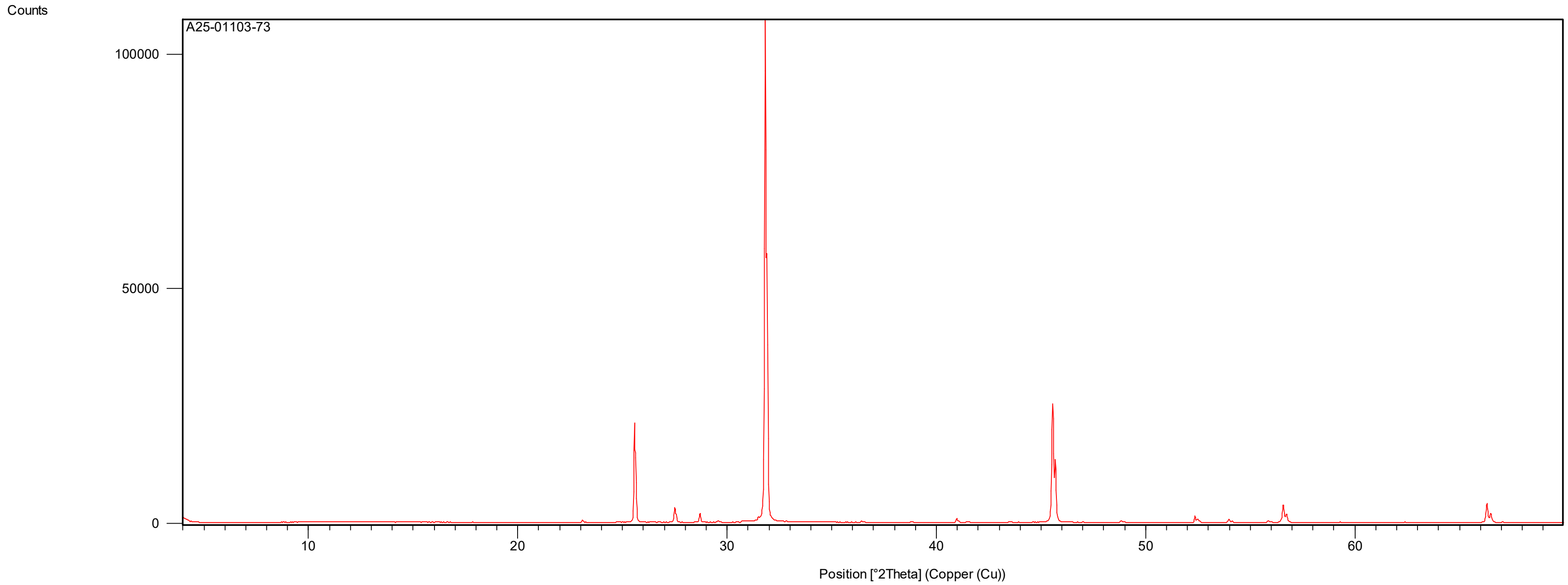

Counts
A25-01103-73
100000
50000
0
10
20
30
40
50
60
Position [°2Theta] (Copper (Cu))

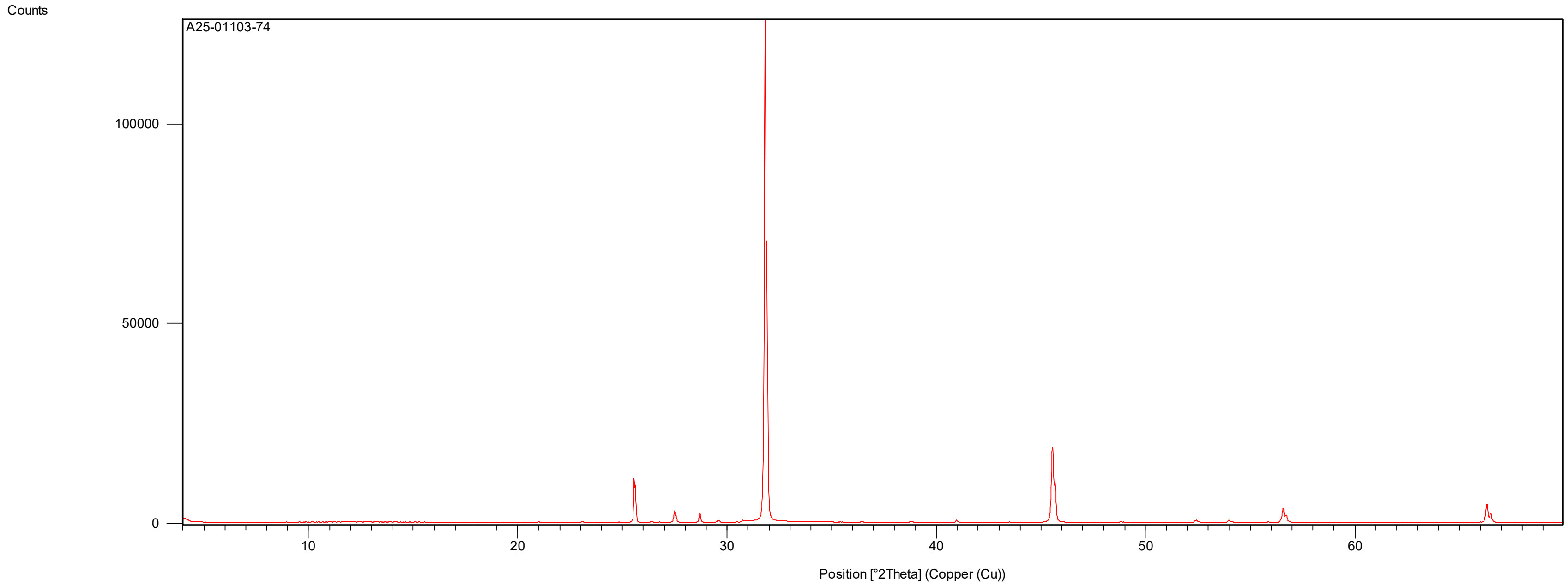
Counts
A25-01103-74
100000
50000
0
10
20
30
40
50
60
Position [°2Theta] (Copper (Cu))

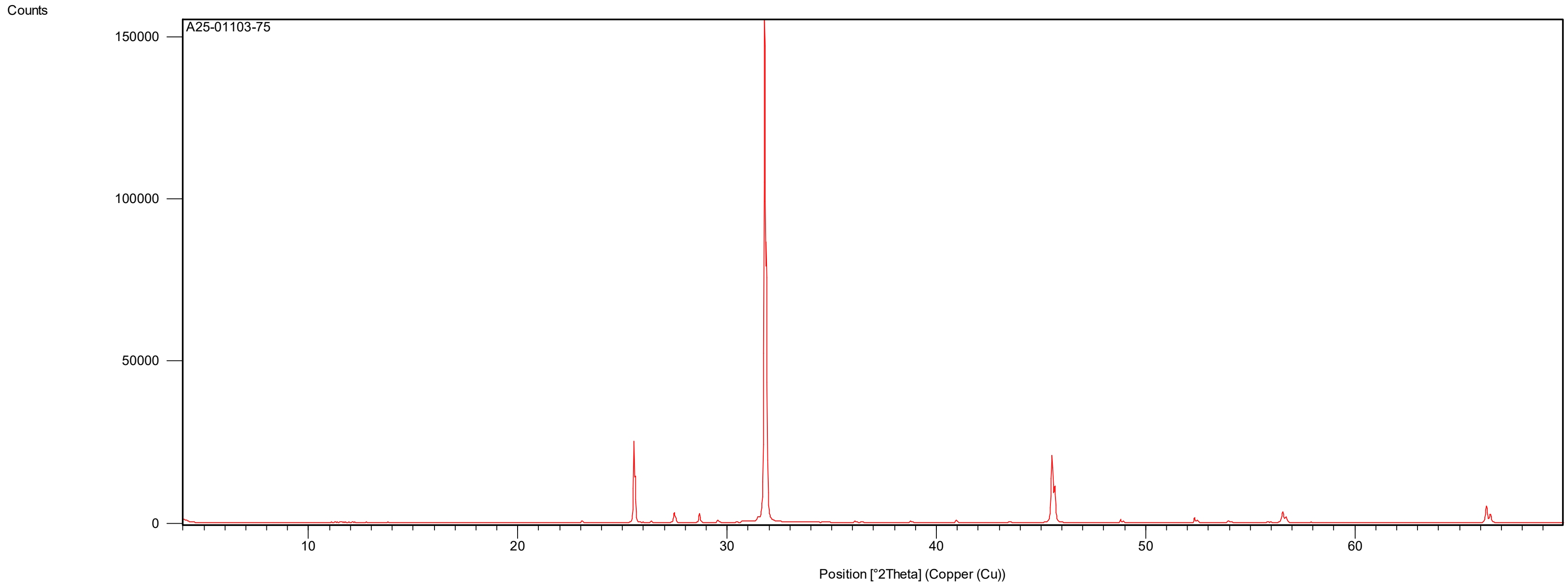
Counts
A25-01103-75
150000
100000
50000
0
10
20
30
40
50
60
Position [°2Theta] (Copper (Cu))

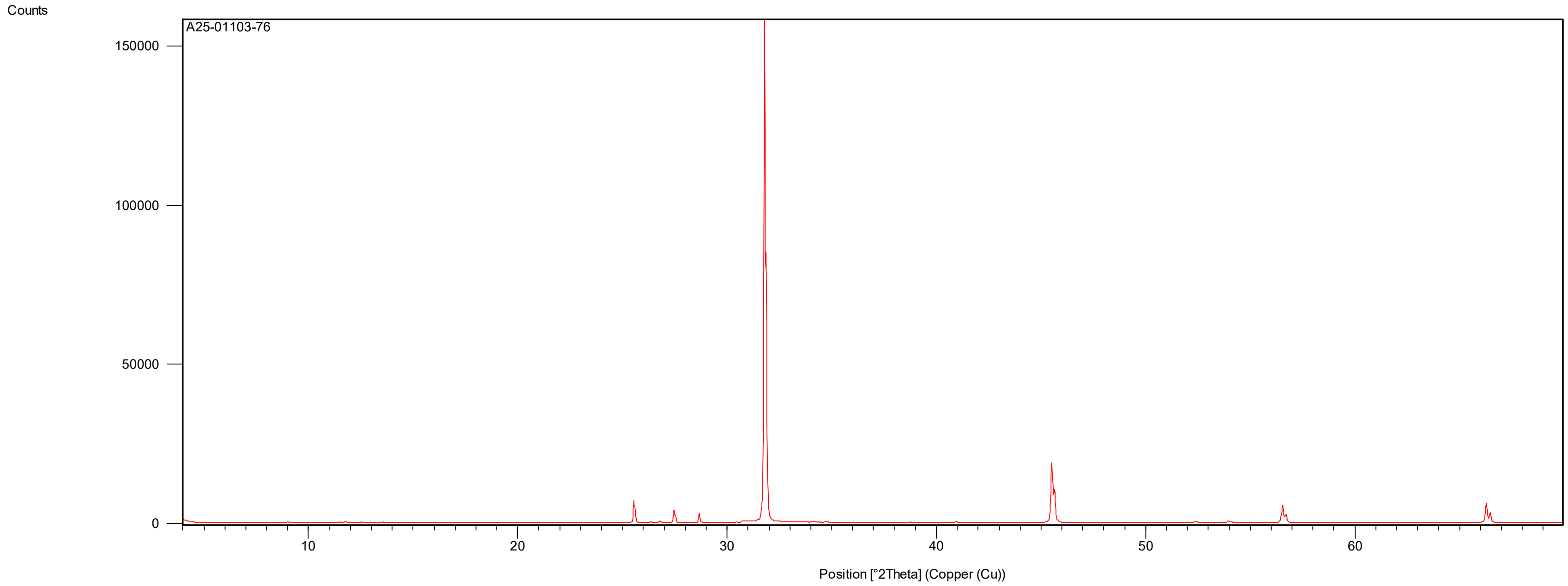
Counts
A25-01103-76
150000
100000
50000
0
10
20
30
40
50
60
Position [°2Theta] (Copper (Cu))

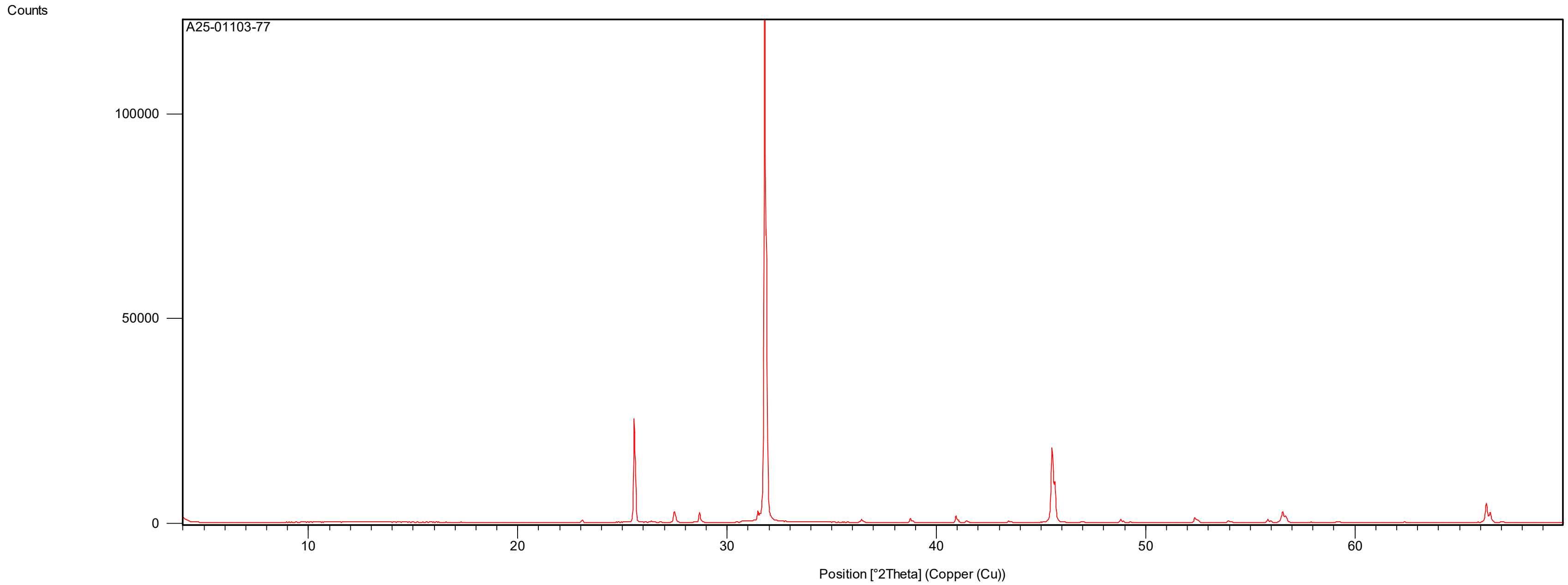

Counts
A25-01103-77
100000
50000
0
10
20
30
40
50
60
Position [°2Theta] (Copper (Cu))

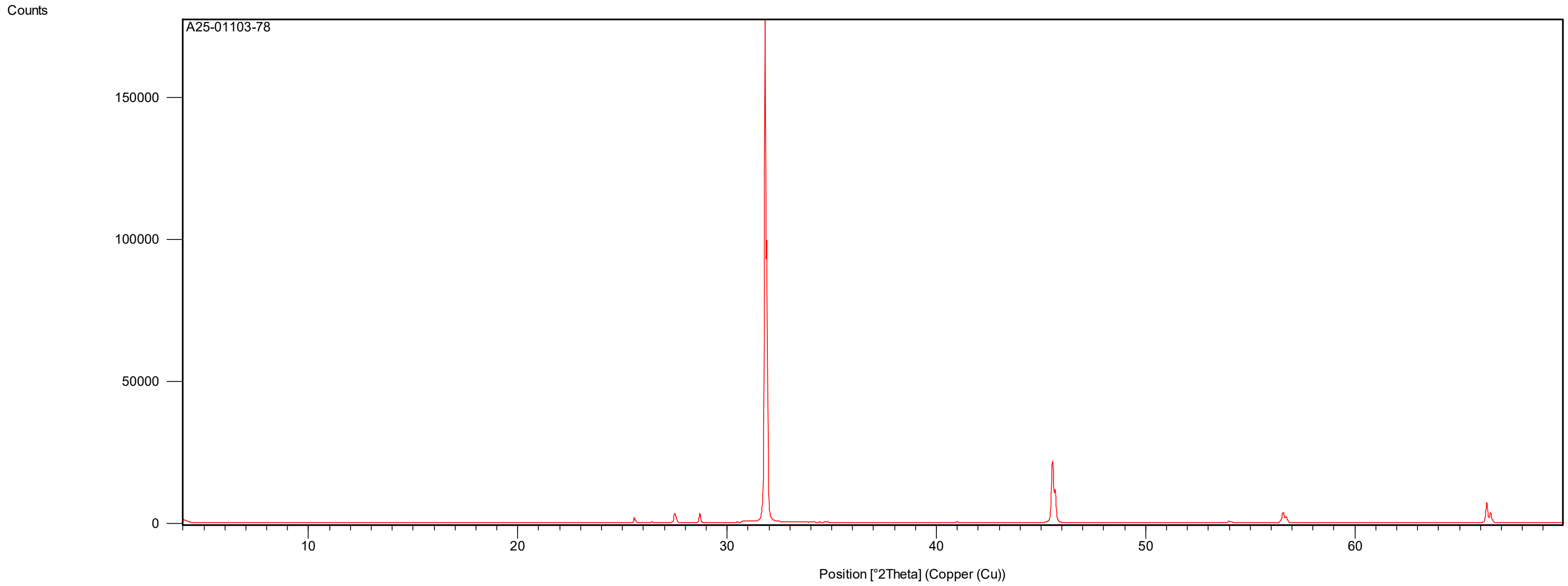
Counts
A25-01103-78
150000
100000
50000
0
10
20
30
40
50
60
Position [°2Theta] (Copper (Cu))

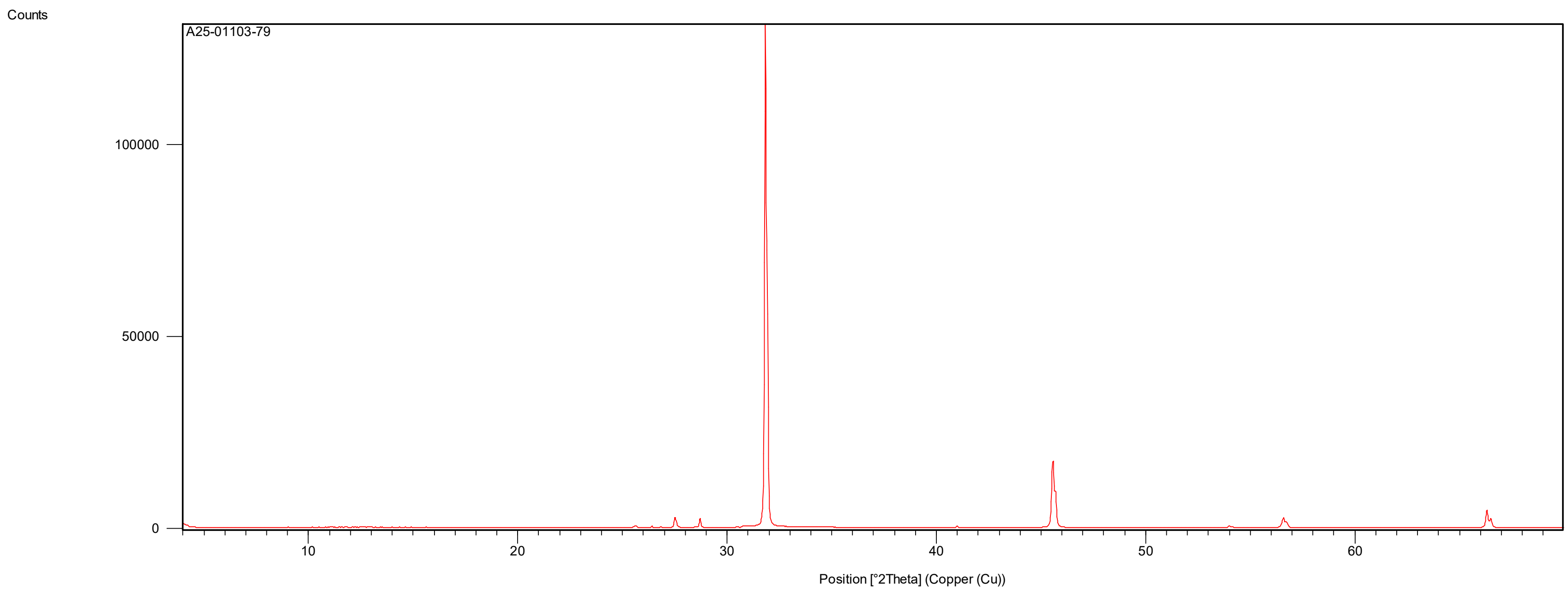
Counts
A25-01103-79
100000
50000
0
10
20
30
40
50
60
Position [°2Theta] (Copper (Cu))

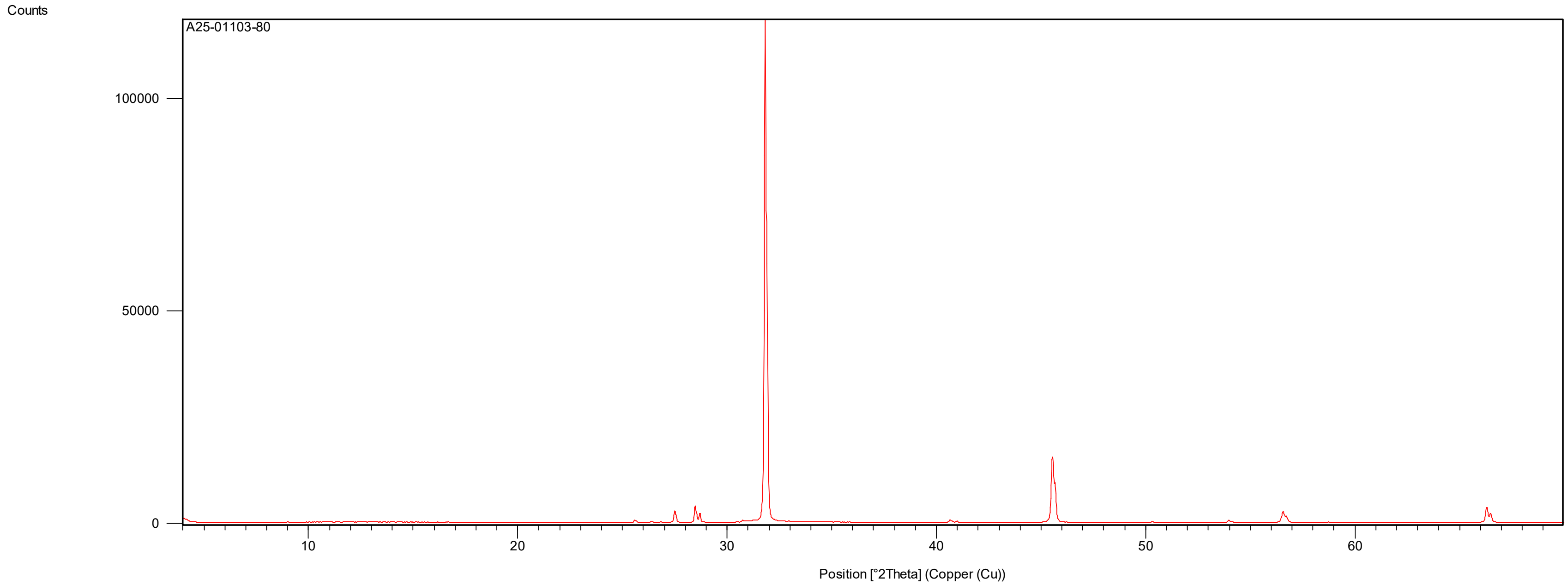
Counts
A25-01103-80
100000
50000
0
10
20
30
40
50
60
Position [°2Theta] (Copper (Cu))

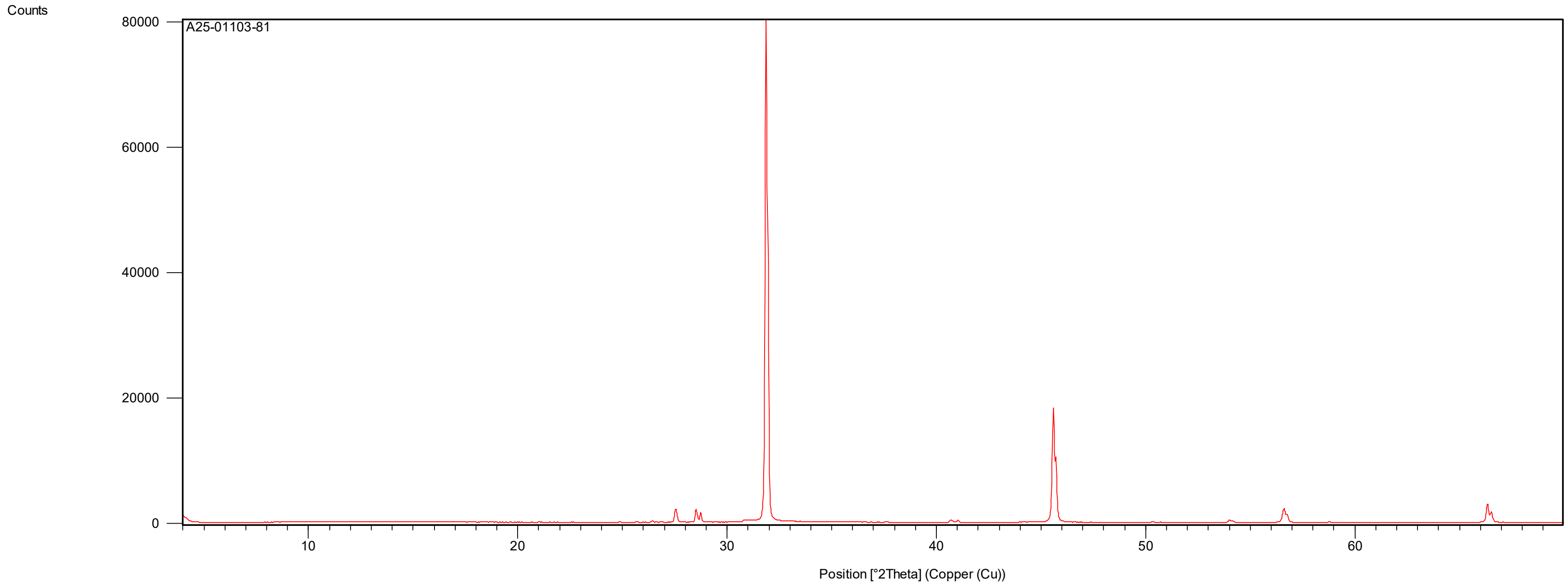
Counts
80000
60000
40000
20000
0
A25-01103-81
10
20
30
40
50
60
Position [°2Theta] (Copper (Cu))

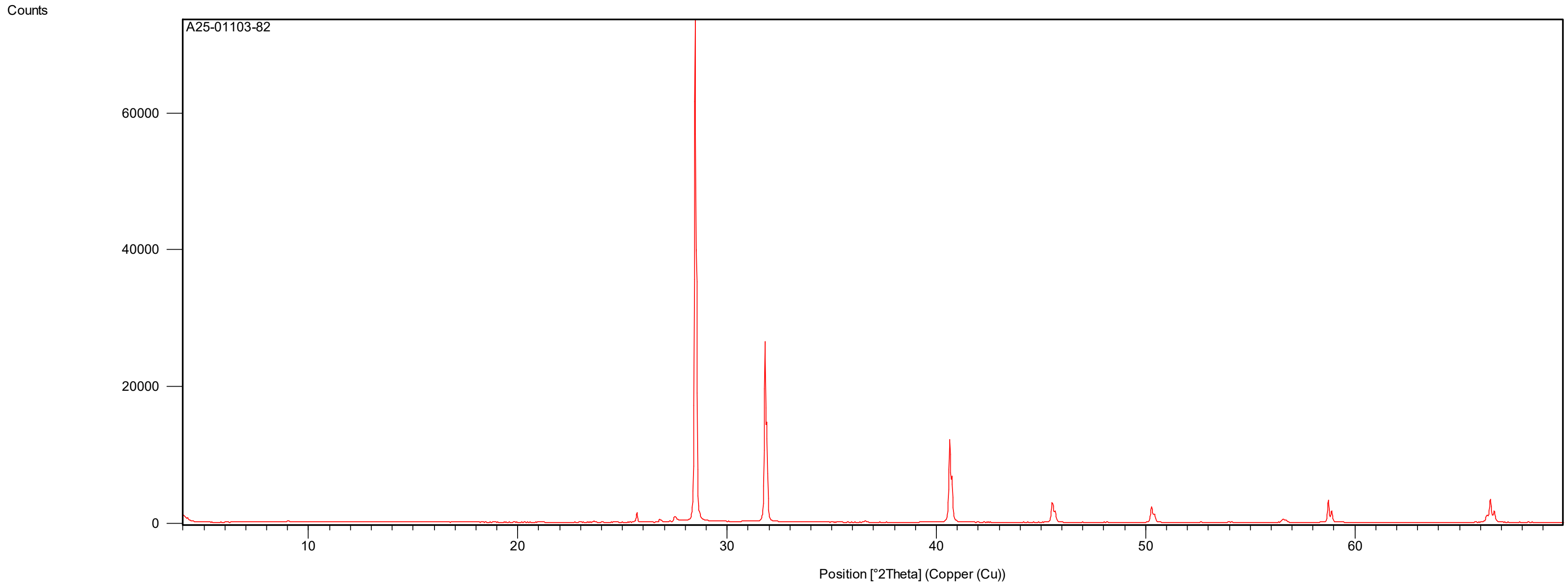
Counts
A25-01103-82
60000
40000
20000
0
10
20
30
40
50
60
Position [°2Theta] (Copper (Cu))

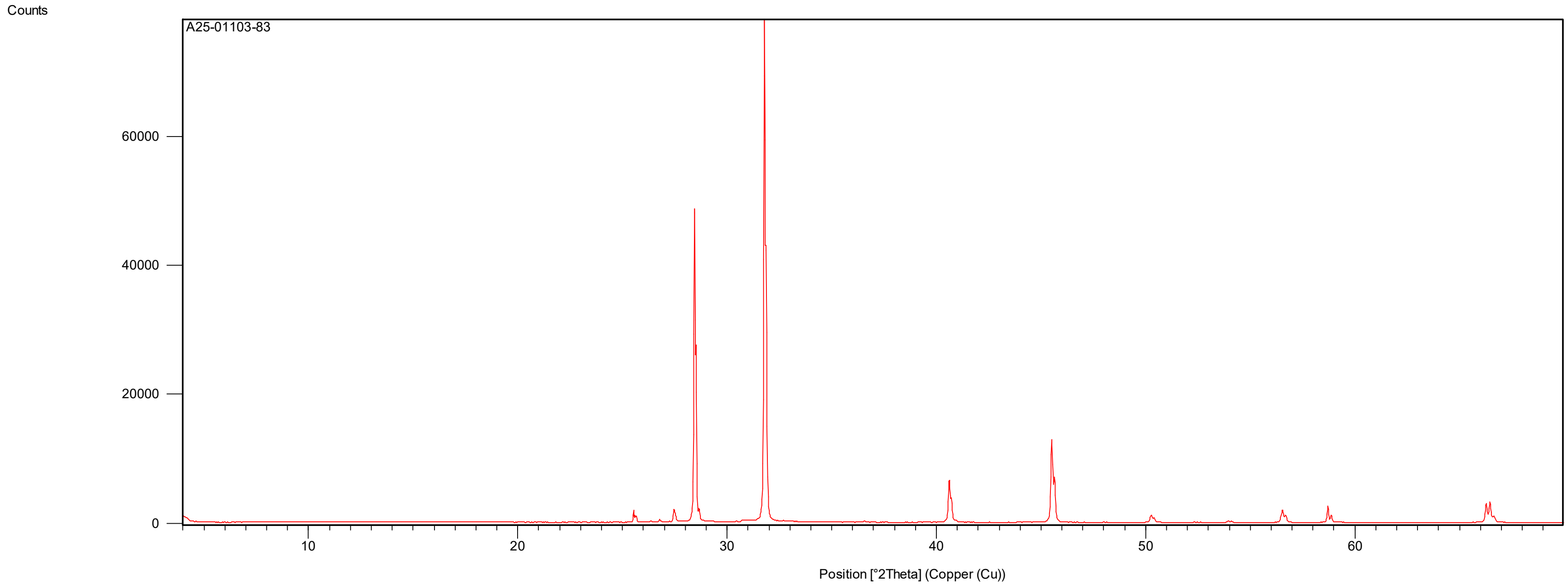
Counts
A25-01103-83
60000
40000
20000
0
10
20
30
40
50
60
Position [°2Theta] (Copper (Cu))

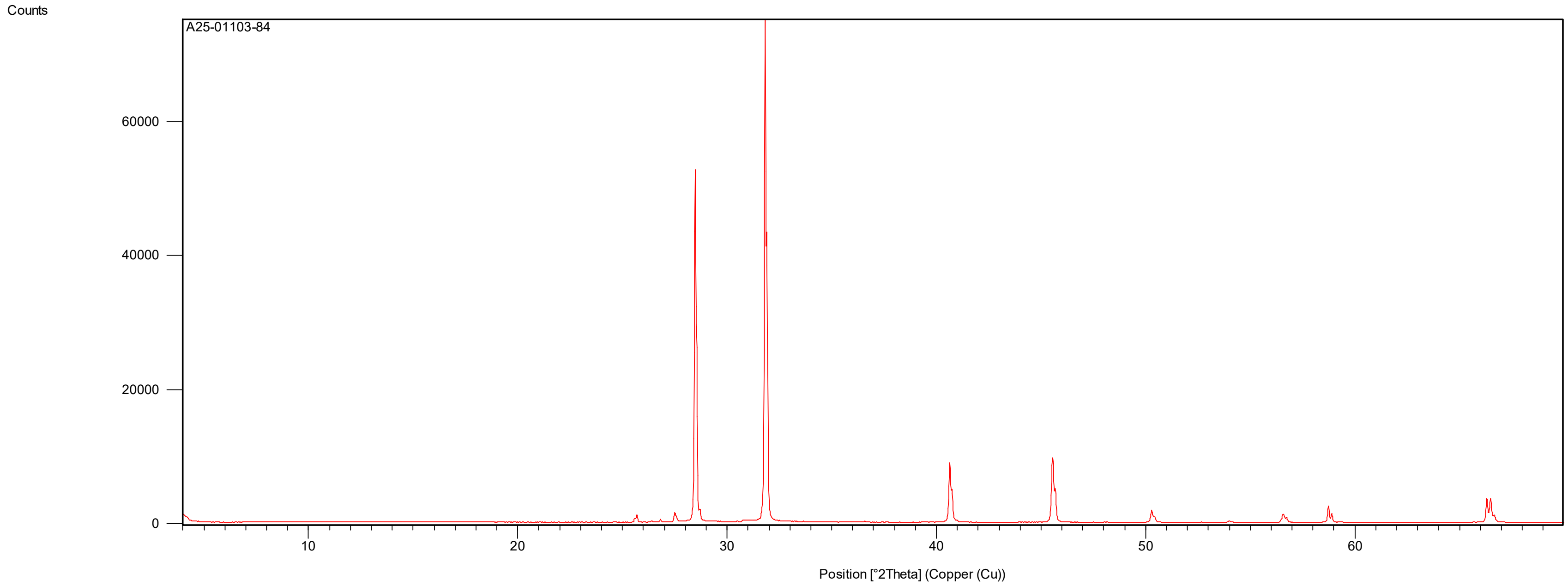
Counts
A25-01103-84
60000
40000
20000
0
10
20
30
40
50
60
Position [°2Theta] (Copper (Cu))

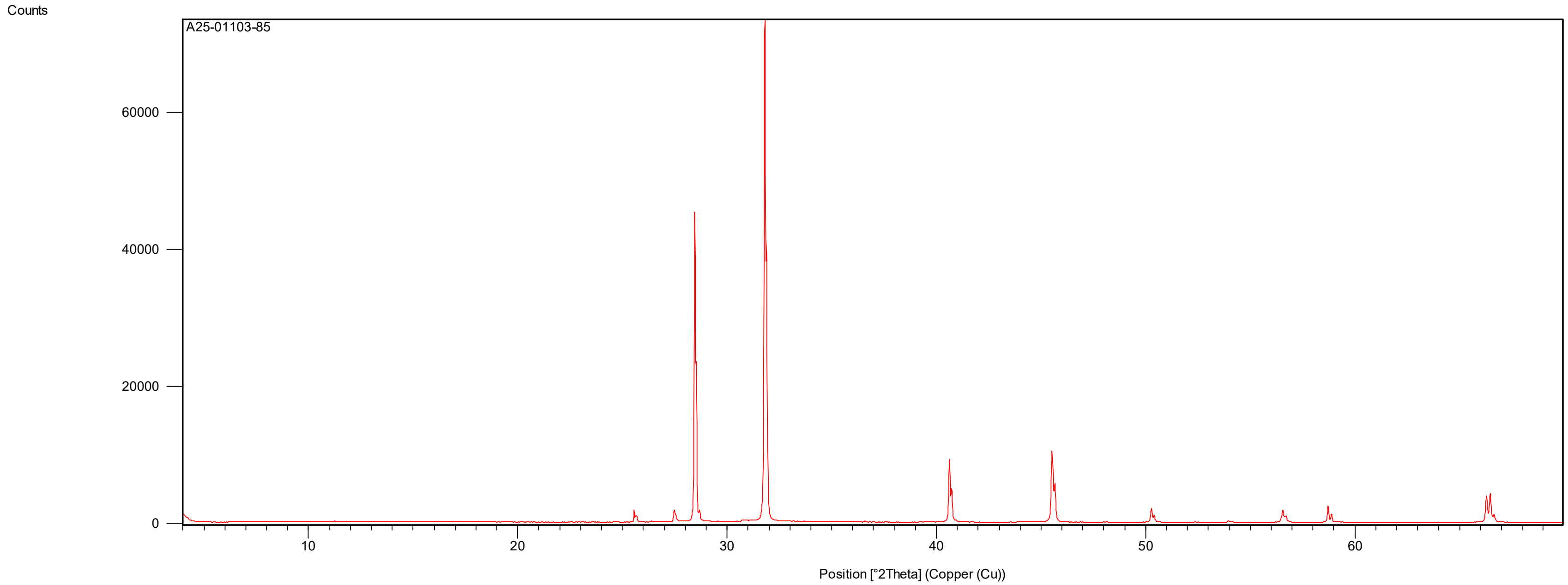
Counts
A25-01103-85
60000
40000
20000
0
10
20
30
40
50
60
Position [°2Theta] (Copper (Cu))

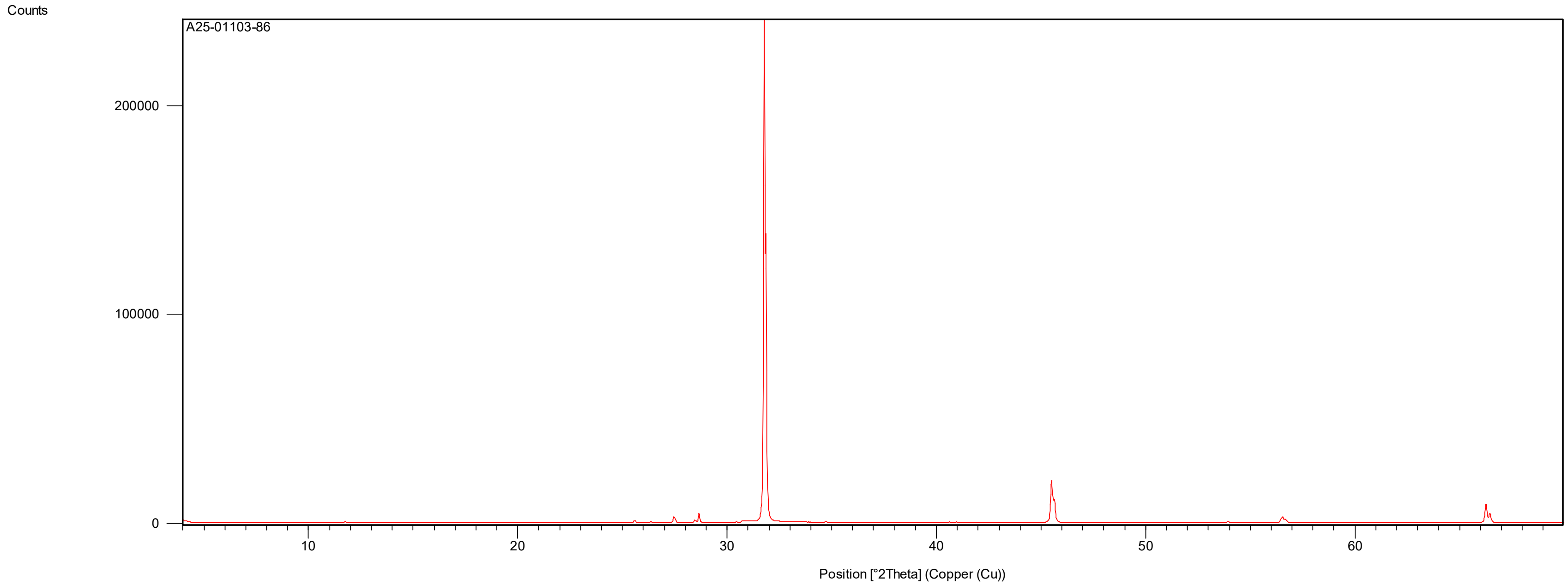
Counts
A25-01103-86
200000
100000
0
10
20
30
40
50
60
Position [°2Theta] (Copper (Cu))

Activation Laboratories Ltd. A25-01103

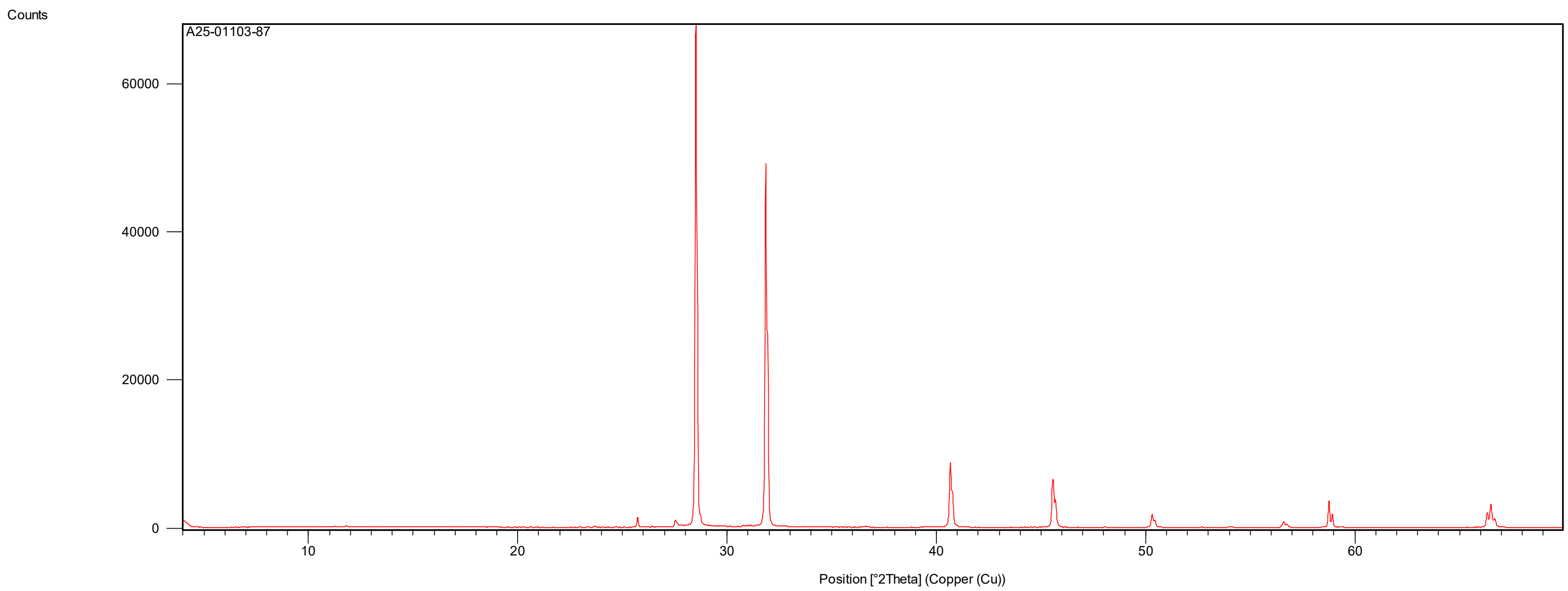

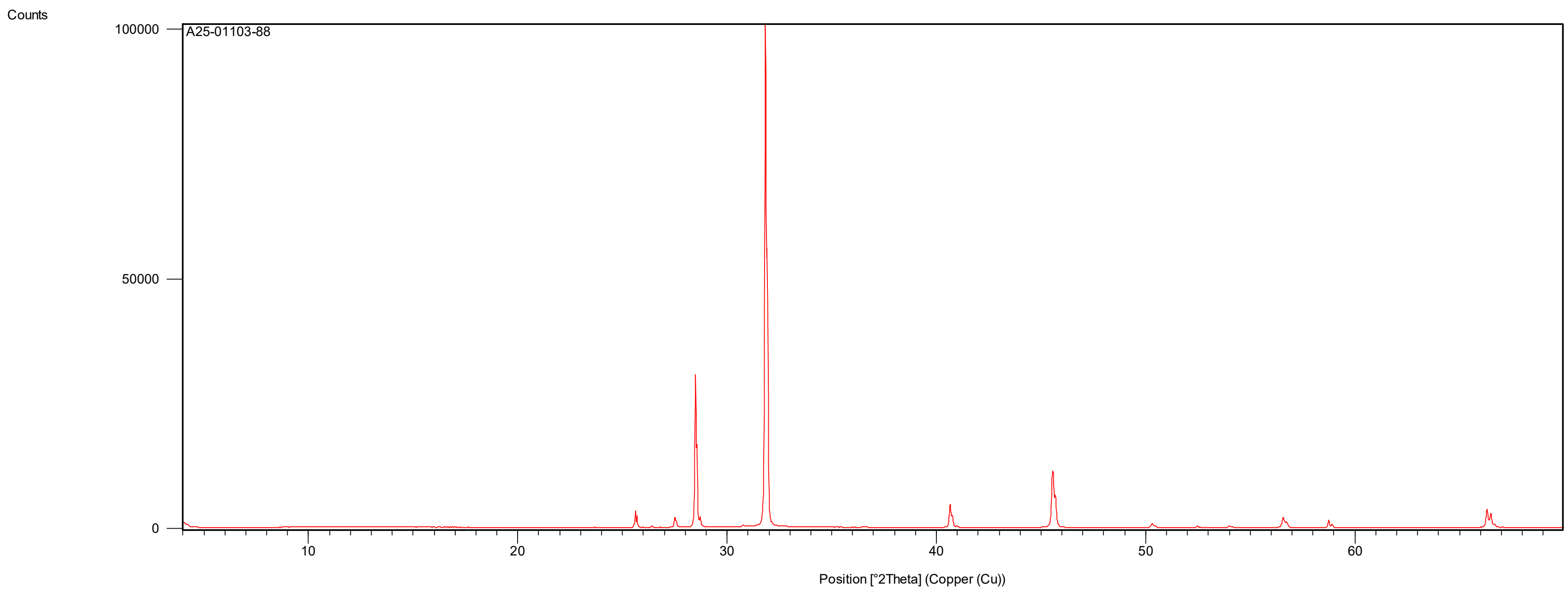

Counts
100000
50000
0
A25-01103-88
10
20
30
40
50
60
Position [°2Theta] (Copper (Cu))

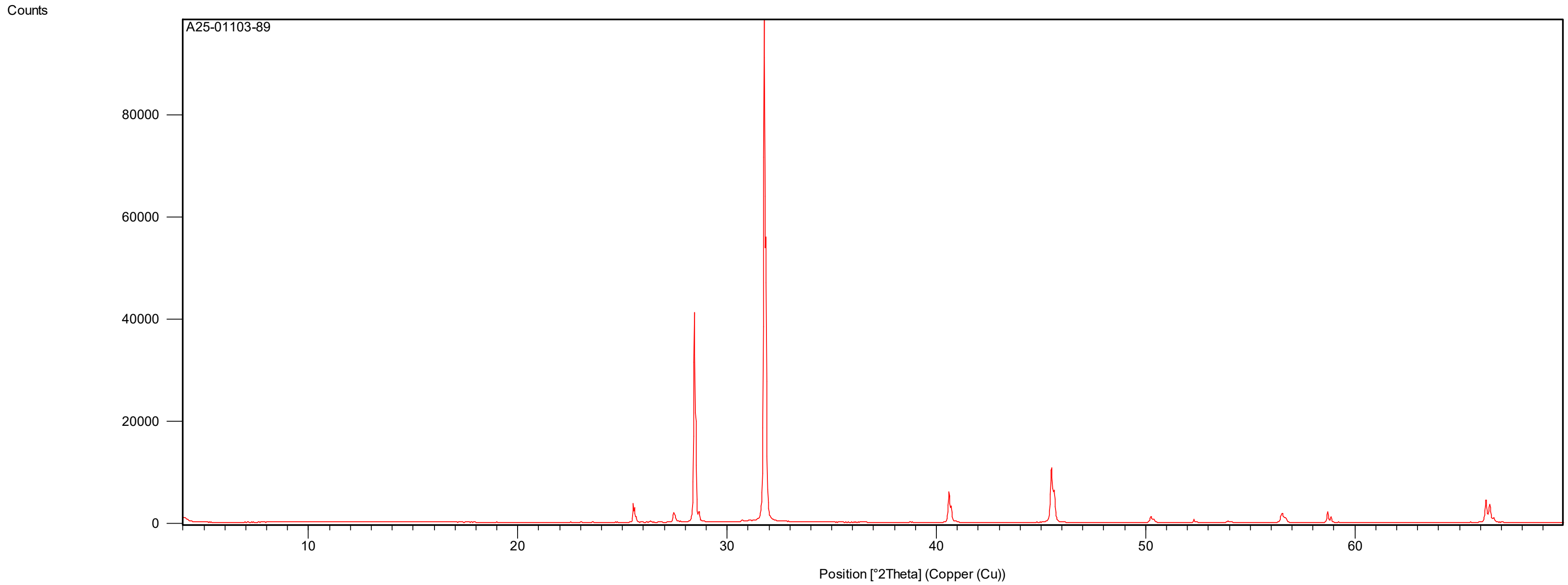
Counts
A25-01103-89
80000
60000
40000
20000
0
10
20
30
40
50
60
Position [°2Theta] (Copper (Cu))

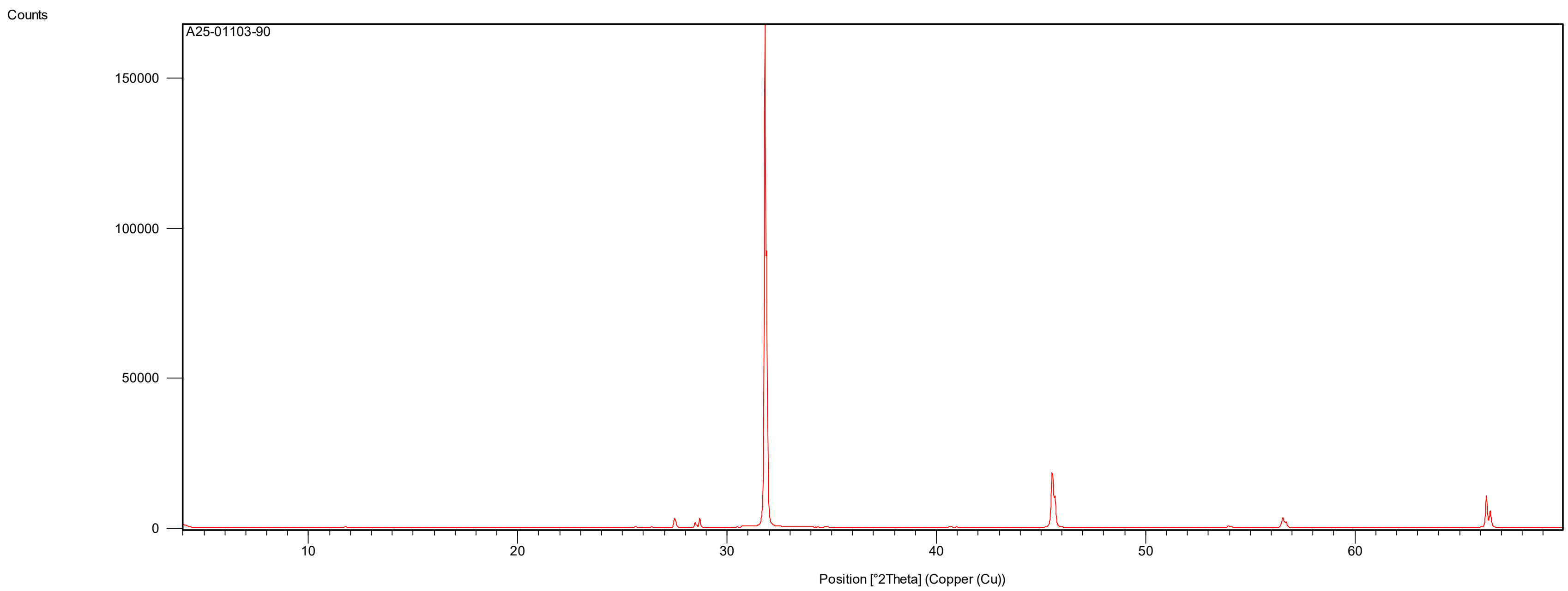
Counts
A25-01103-90
150000
100000
50000
0
10
20
30
40
50
60
Position [°2Theta] (Copper (Cu))

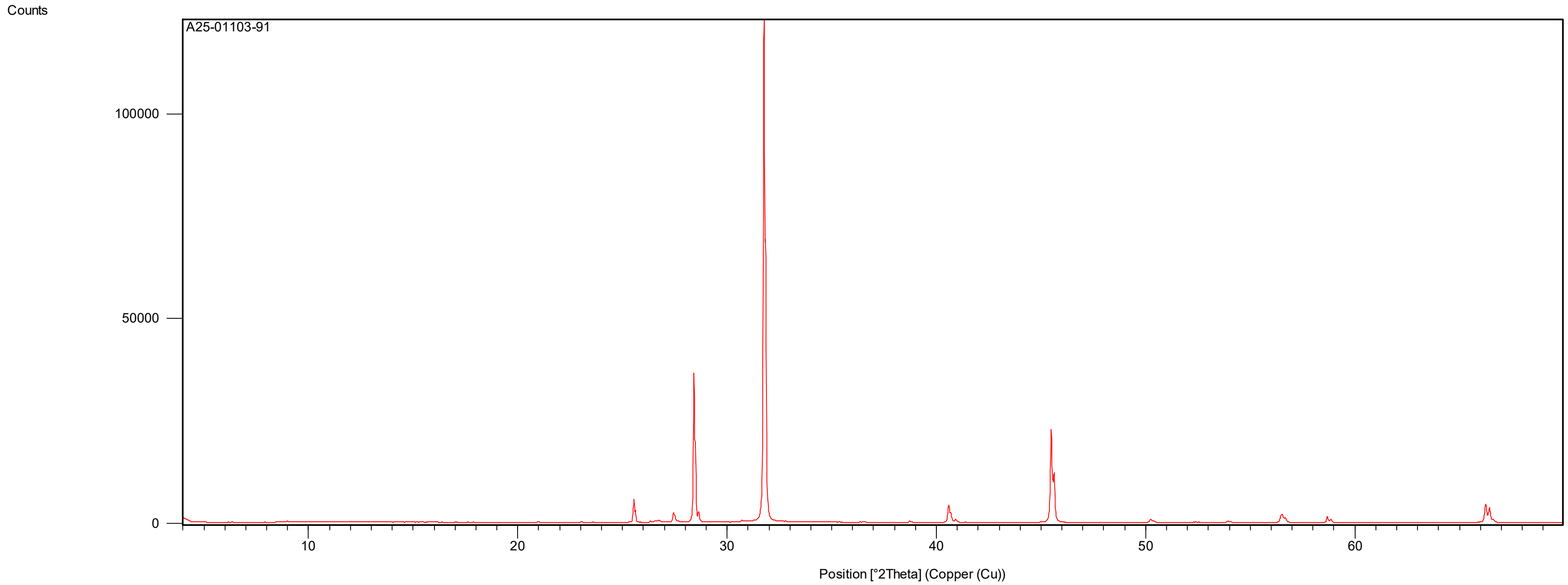
Counts
A25-01103-91
100000
50000
0
10
20
30
40
50
60
Position [°2Theta] (Copper (Cu))

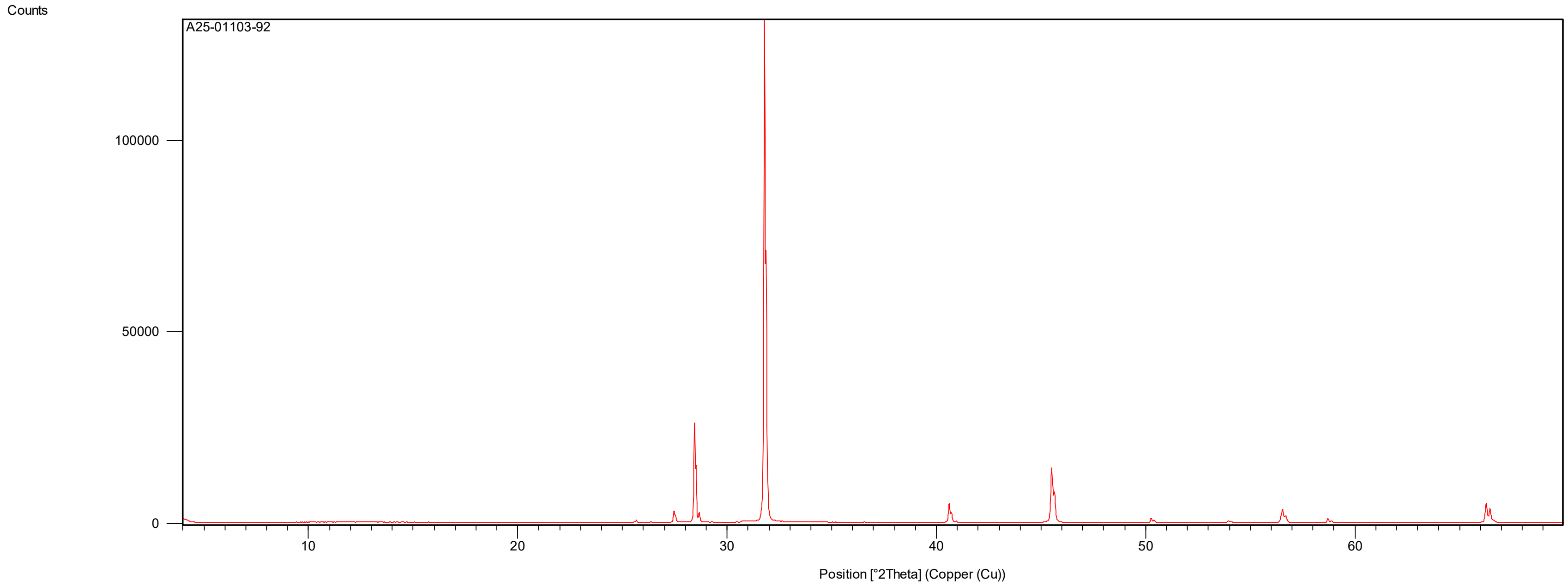
Counts
A25-01103-92
100000
50000
0
10
20
30
40
50
60
Position [°2Theta] (Copper (Cu))

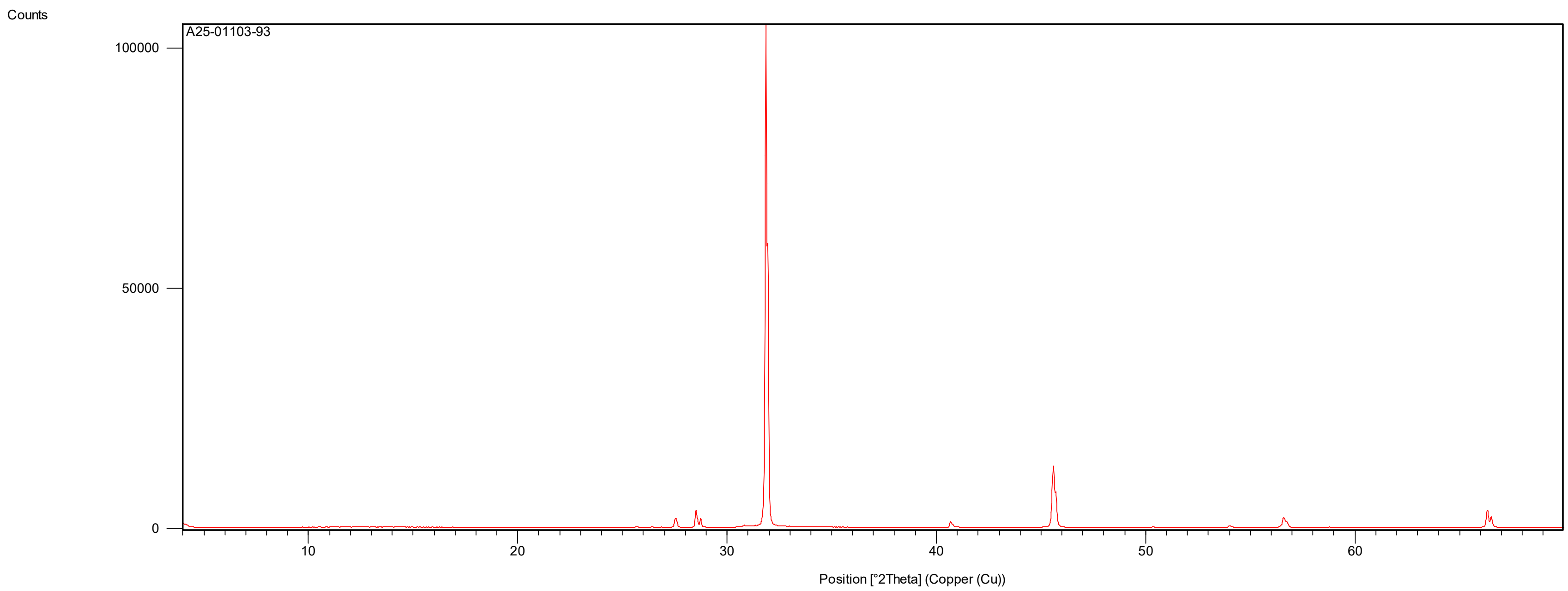
Counts
A25-01103-93
100000
50000
0
10
20
30
40
50
60
Position [°2Theta] (Copper (Cu))

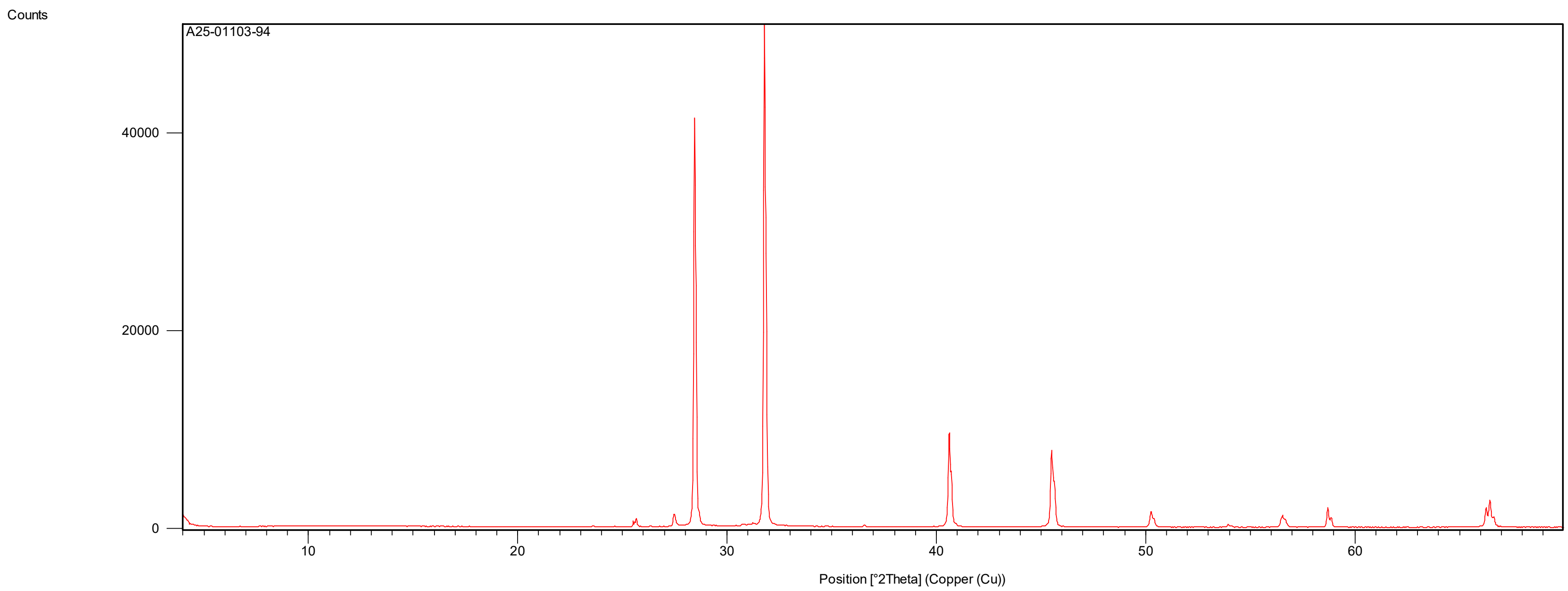
Counts
A25-01103-94
40000
20000
0
10
20
30
40
50
60
Position [°2Theta] (Copper (Cu))

Activation Laboratories Ltd. A25-01103

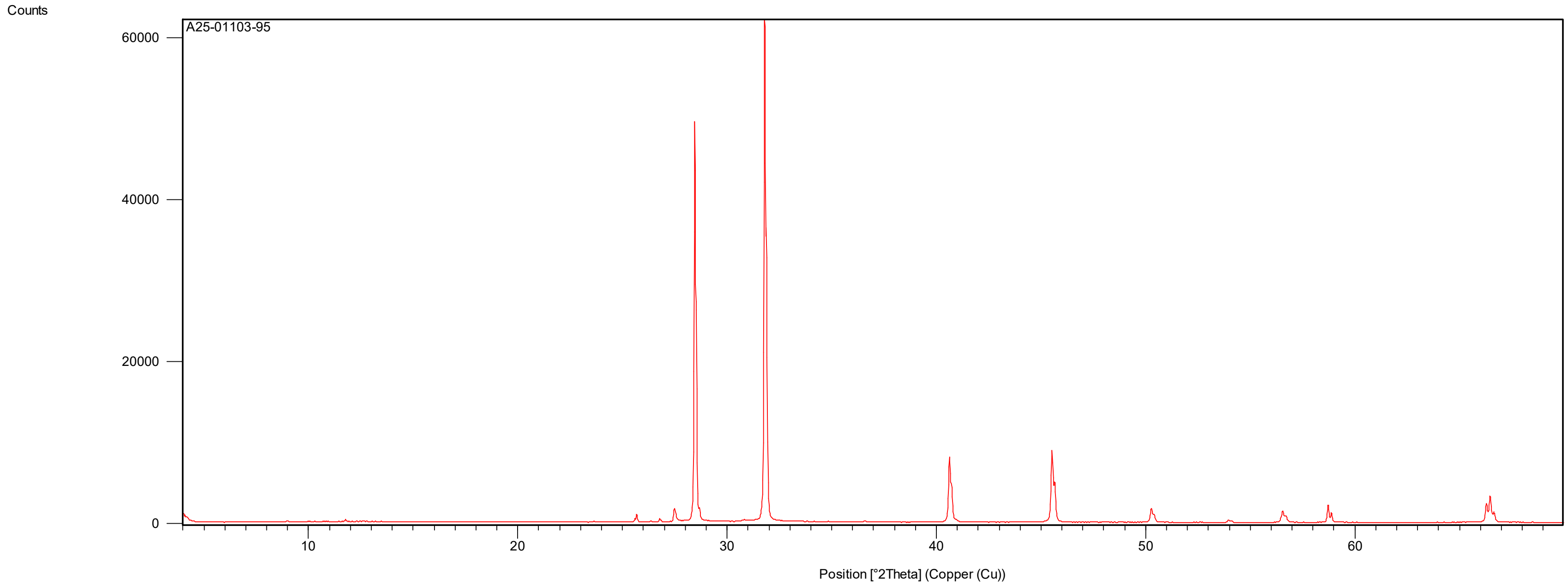

Activation Laboratories Ltd. A25-01103

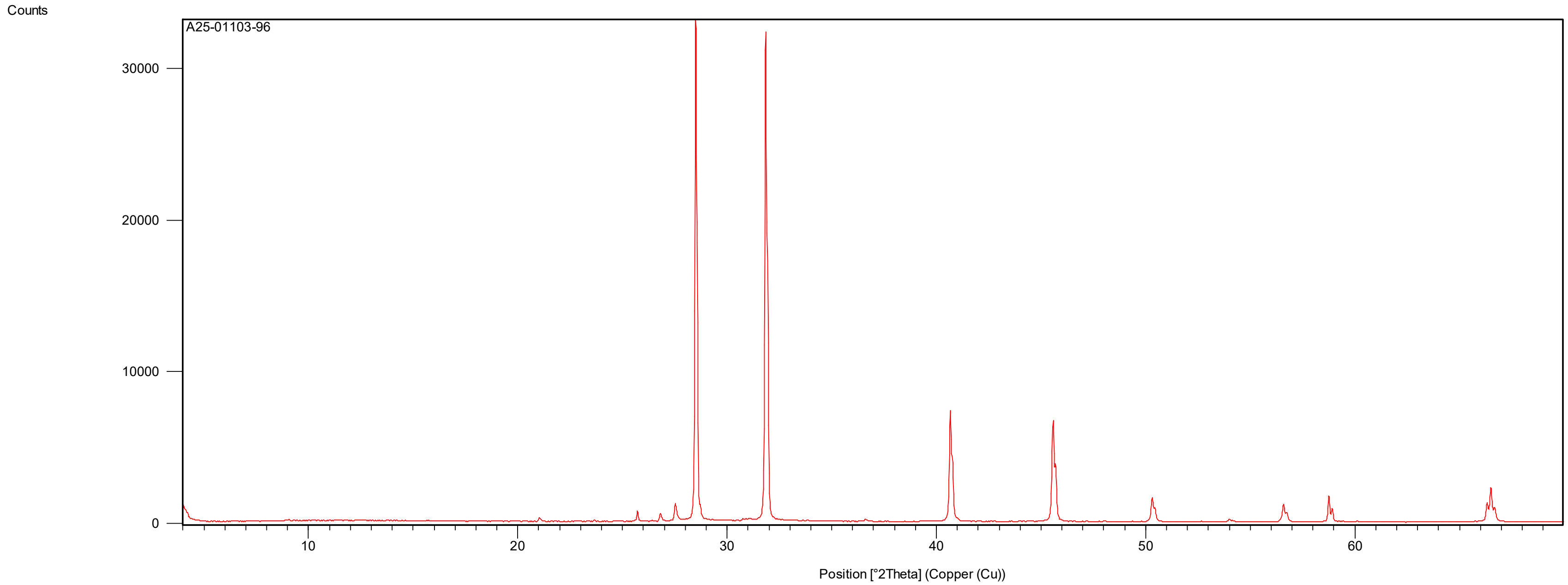

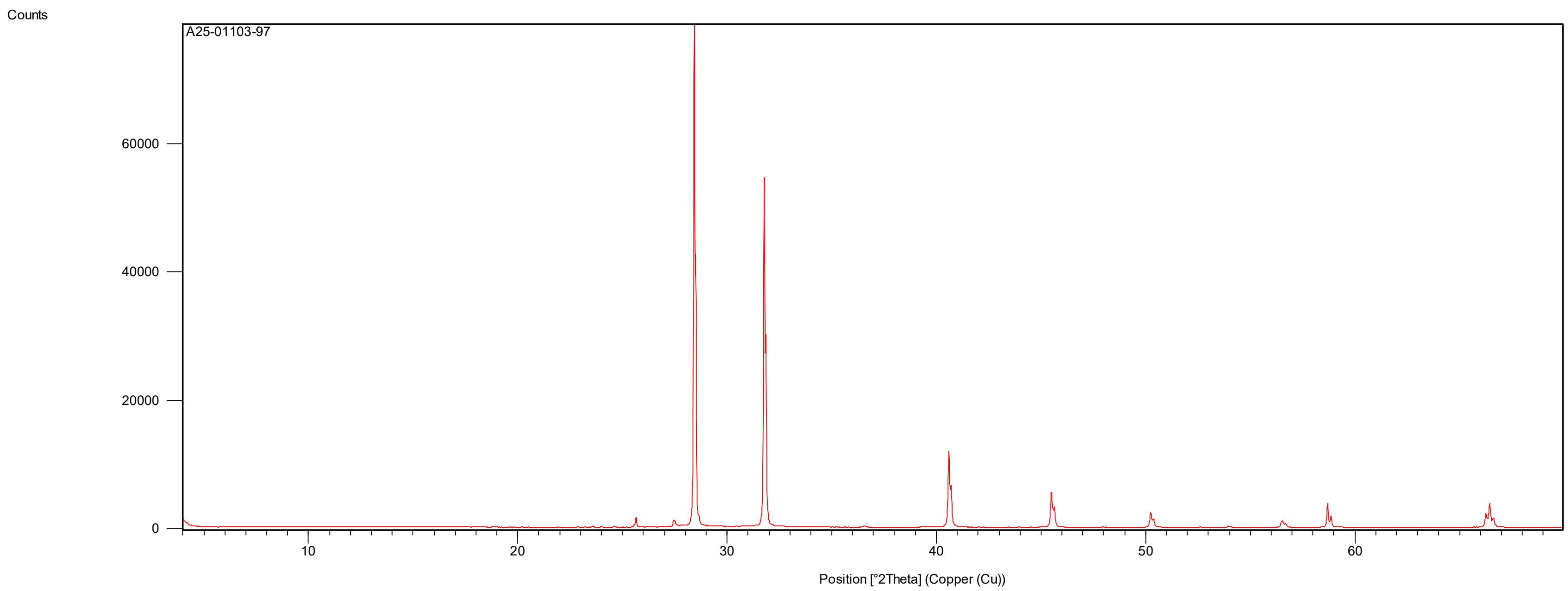
Counts
A25-01103-97
60000
40000
20000
0
10
20
30
40
50
60
Position [°2Theta] (Copper (Cu))

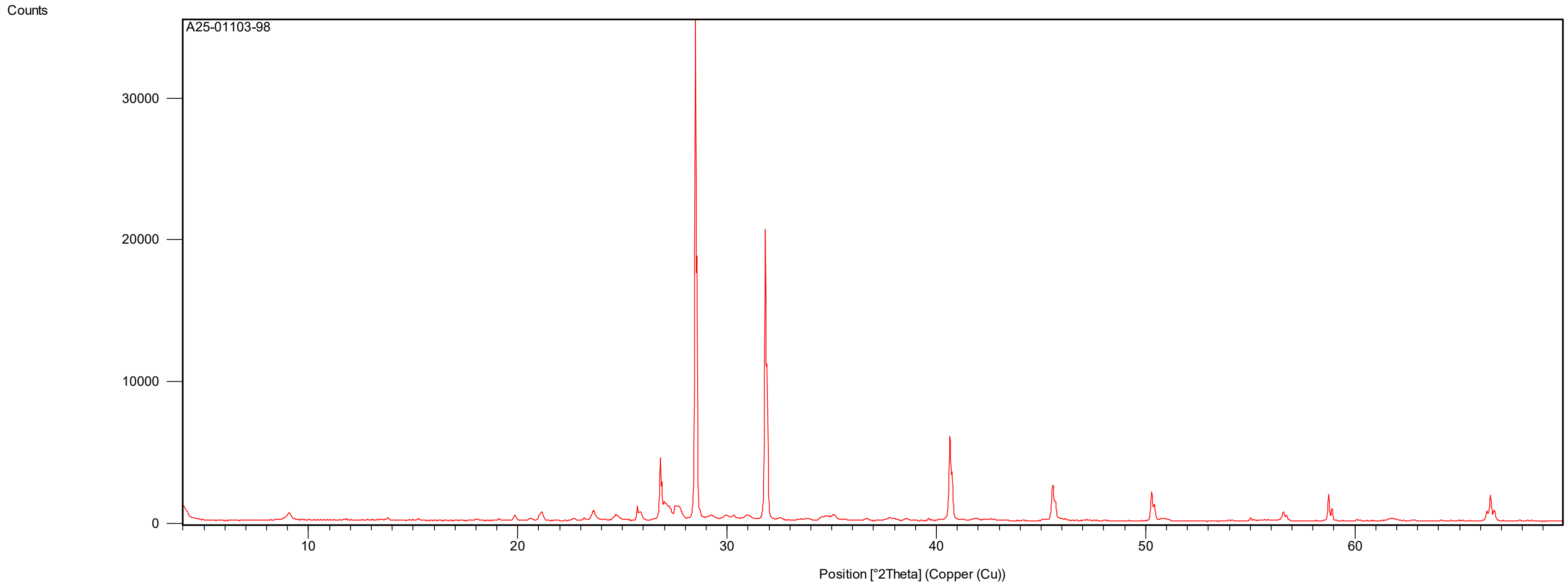
Counts
A25-01103-98
30000
20000
10000
0
10
20
30
40
50
60
Position [°2Theta] (Copper (Cu))

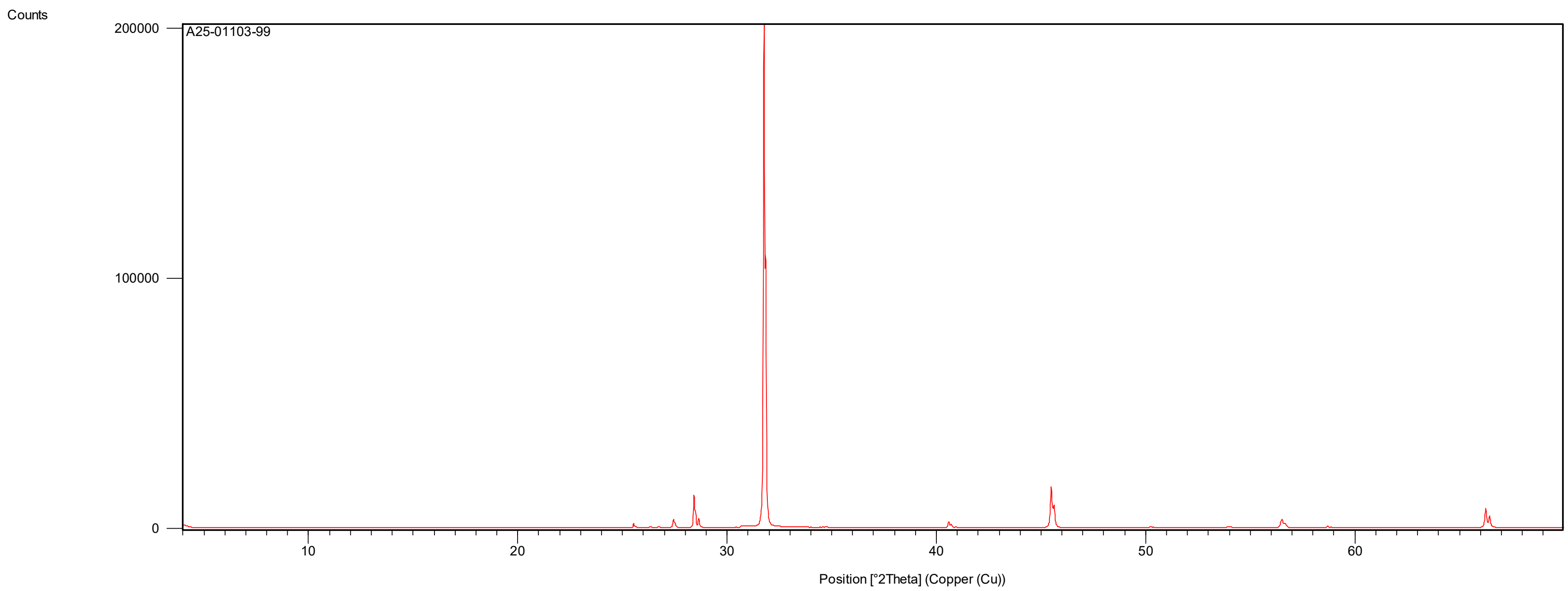
Counts
200000
100000
0
A25-01103-99
10
20
30
40
50
60
Position [°2Theta] (Copper (Cu))

Activation Laboratories Ltd. A25-01103

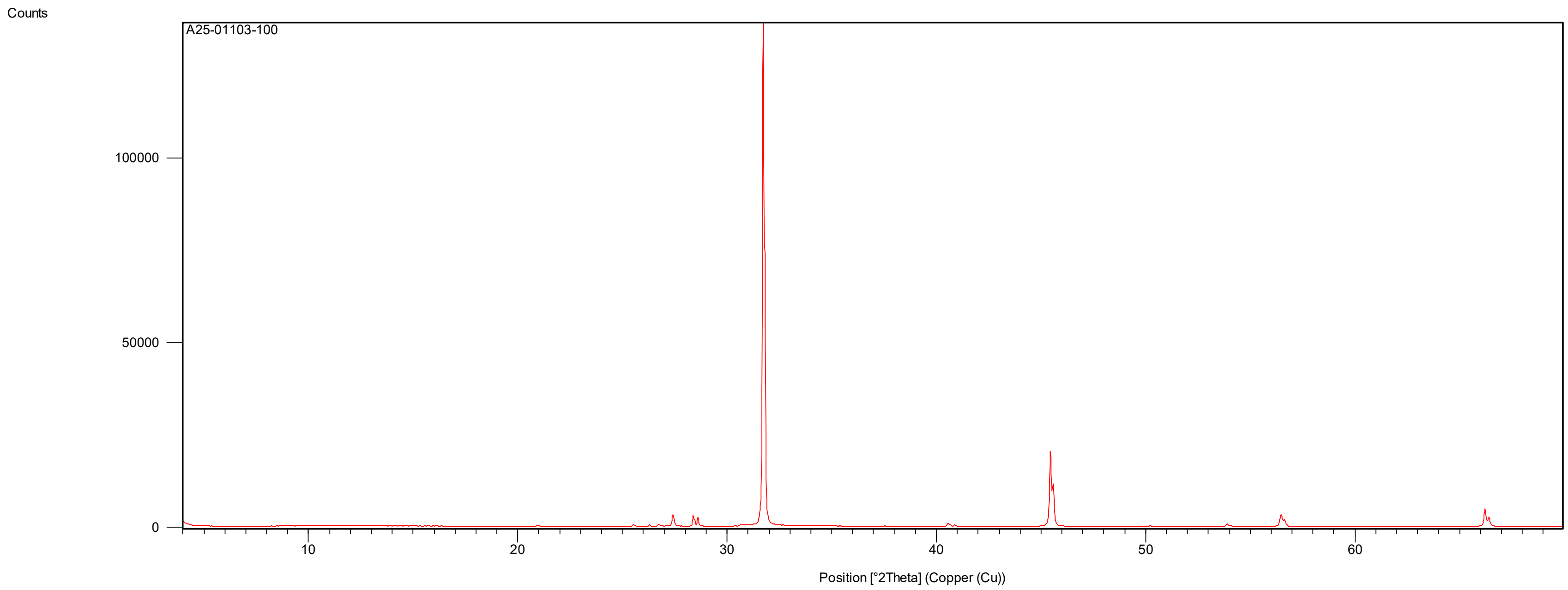

Supplement: Supplementary file 9 [file mmc9.pdf]
