# Supplementary material for: Geochemistry and X-ray diffraction data from rock salts and saltwork wastes of Canada: data compilation
Source: Data Brief. 2026 Jun 6;67:112941. doi: 10.1016/j.dib.2026.112941 (PMC13292661; doi:10.1016/j.dib.2026.112941)
Supplement: Supplementary file 10 [file mmc10.pdf]

# X-ray Diffraction Analysis of Fifty Five Samples

W.O. # A25-01979
Invoice # A25-01979

Client: GSC/NRCan

Attn: Pavel Kabanov

Date Reported: March 28, 2025

Activation Laboratories Ltd.
41 Bittern Street, Ancaster, ON L9G 4V5, Tel: +1.905.648.9611, Fax: +1.905.648.96123

## Method

Fifty five samples were submitted for semi-quantitative X-ray diffraction analysis and three samples were submitted for clay speciation analysis. For the semi-quantitative XRD analysis, a portion of each pulverized sample was loaded into a standard holder. For clay speciation analysis, a portion of each sample was dispersed in distilled water and the < 4 µm size fraction was separated by gravity settling of particles in suspension. Oriented slides of the < 4 µm size fraction were prepared by placing a portion of the suspension onto a glass slide. The oriented slides were analyzed air-dry and after treatment with ethylene glycol.

The X-ray diffraction analysis was performed on a Bruker D8 Endeavour diffractometer equipped with Cu X-ray source and operating at the following conditions: 40 kV and 40 mA; range 4 - 70 deg 2θ; step size 0.02 deg 2θ; time per step 0.5 sec; fixed divergence slit, angle $0.3^0$; sample rotation 15 rpm. The PDF4/Minerals ICDD database was used for mineral identification. The quantities of the crystalline mineral phases were determined using Rietveld method. The Rietveld method is based on the calculation of the full diffraction pattern from crystal structure data. The relative proportions of clay minerals in the < 4 µm size fraction were calculated using the relative ratios of their basal-peak areas.

## Results

The minerals identified in the samples and their abundances are in Table 1 and the relative proportions of the clay minerals in the < 4 µm size fraction are in Table 2. The diffraction patterns are in Appendix 1.

**Table 1.** Mineral abundances (wt %)

| Client ID | Actlabs ID | Halite | Sylvite | Carnallite | Tachyhydrite | Anhydrite | Gypsum | Quartz |
|---|---|---|---|---|---|---|---|---|
| Fisher2 612.14 | A25-01979-1 | 4.3 | n.d. | n.d. | n.d. | n.d. | n.d. | 12.4 |
| Fisher2 614.38 | A25-01979-2 | 2.1 | n.d. | n.d. | n.d. | 19.4 | n.d. | 19.3 |
| Fisher2 616.75 | A25-01979-3 | 9.3 | n.d. | n.d. | n.d. | 76.1 | n.d. | 8.1 |
| Fisher2 654.35 | A25-01979-4 | 91.8 | 1.5 | 5.7 | n.d. | 0.3 | n.d. | n.d. |
| Fisher2 626.50 | A25-01979-5 | 98.2 | 1.0 | n.d. | n.d. | 0.8 | n.d. | trace |
| Fisher2 634.45 | A25-01979-6 | 96.4 | 1.1 | n.d. | n.d. | 1.5 | n.d. | trace |
| Fisher2 643.65 | A25-01979-7 | 95.4 | 1.1 | n.d. | n.d. | 2.7 | n.d. | trace |
| Fisher2 652.30 | A25-01979-8 | 95.9 | 1.0 | n.d. | n.d. | 2.5 | n.d. | n.d. |
| Fisher2 653.50 | A25-01979-9 | 82.3 | 2.5 | 14.7 | n.d. | n.d. | n.d. | n.d. |
| fisher2 666.4 | A25-01979-10 | 90.3 | 0.9 | n.d. | n.d. | 7.8 | n.d. | 0.6 |
| fisher2 669.2 | A25-01979-11 | 98.0 | 1.1 | n.d. | n.d. | 0.5 | n.d. | trace |
| fisher2 672.45 | A25-01979-12 | 91.2 | 1.2 | 6.9 | n.d. | n.d. | n.d. | n.d. |
| fisher2 674.95 | A25-01979-13 | 19.5 | n.d. | n.d. | n.d. | 72.8 | trace | 3.0 |
| fisher2  676.85 | A25-01979-14 | 96.7 | 1.4 | n.d. | n.d. | 1.4 | n.d. | 0.2 |
| fisher2 678.03 | A25-01979-15 | 98.5 | 1.2 | n.d. | n.d. | 0.3 | n.d. | trace |
| fisher2 699.3 | A25-01979-16 | 97.3 | 1.2 | 0.7 | n.d. | 0.8 | n.d. | n.d. |
| fisher2 687.70 | A25-01979-17 | 76.2 | 1.0 | 22.6 | n.d. | 0.2 | n.d. | n.d. |
| fisher2 687.3 | A25-01979-18 | 98.5 | 1.1 | n.d. | n.d. | 0.4 | n.d. | trace |
| fisher2 696.6 | A25-01979-19 | 0.2 | n.d. | n.d. | n.d. | n.d. | n.d. | 1.8 |
| fisher2 691.8 | A25-01979-20 | 97.1 | 1.5 | n.d. | n.d. | 0.8 | n.d. | trace |
| fisher2 703.5 | A25-01979-21 | 94.3 | 1.0 | 1.0 | n.d. | 3.3 | n.d. | n.d. |
| fisher2 704.25 | A25-01979-22 | 97.2 | 1.0 | n.d. | n.d. | 1.5 | n.d. | n.d. |
| fisher2 707.35 | A25-01979-23 | 2.2 | n.d. | 13.4 | 5.7 | n.d. | trace | 6.6 |
| fisher2 710.1 | A25-01979-24 | 1.0 | n.d. | n.d. | 25.1 | 1.5 | 0.8 | 11.1 |
| fisher2 710.83 | A25-01979-25 | 5.5 | n.d. | n.d. | n.d. | 94.1 | n.d. | 0.4 |
| fisher2 719 | A25-01979-26 | 98.0 | 1.1 | n.d. | n.d. | 0.9 | n.d. | n.d. |
| fisher2 719.6 | A25-01979-27 | 96.3 | 1.0 | n.d. | n.d. | 2.2 | n.d. | trace |

| Client ID | Actlabs ID | Halite | Sylvite | Carnallite | Tachyhydrite | Anhydrite | Gypsum | Quartz |
|---|---|---|---|---|---|---|---|---|
| fisher2 720.85 | A25-01979-28 | 97.6 | 1.1 | n.d. | n.d. | 1.3 | n.d. | trace |
| fisher2 725.30 | A25-01979-29 | 97.4 | 1.2 | n.d. | n.d. | 1.4 | n.d. | trace |
| fisher2 727.30 | A25-01979-30 | 97.8 | 1.2 | n.d. | n.d. | 0.4 | n.d. | 0.2 |
| fisher2 728.1 | A25-01979-31 | 98.3 | 1.3 | n.d. | n.d. | 0.4 | n.d. | n.d. |
| fisher2 730.35 | A25-01979-32 | 98.2 | 1.2 | n.d. | n.d. | 0.6 | n.d. | n.d. |
| fisher2 734.4 | A25-01979-33 | 97.0 | 1.1 | n.d. | n.d. | 1.9 | n.d. | n.d. |
| fisher2 733.8 | A25-01979-34 | 97.8 | 1.2 | n.d. | n.d. | 0.3 | n.d. | n.d. |
| fisher2 738.75 | A25-01979-35 | 96.4 | 1.2 | n.d. | n.d. | 2.4 | n.d. | trace |
| fisher2 747.75 | A25-01979-36 | 98.4 | 1.2 | n.d. | n.d. | 0.3 | n.d. | 0.1 |
| fisher2 745.6 | A25-01979-37 | 97.9 | 1.1 | n.d. | n.d. | 1.0 | n.d. | n.d. |
| fisher2 749.0 | A25-01979-38 | 98.2 | 1.4 | n.d. | n.d. | n.d. | n.d. | 0.1 |
| fisher2 751.72 | A25-01979-39 | 98.7 | 1.3 | n.d. | n.d. | n.d. | n.d. | trace |
| fisher2 757.55 | A25-01979-40 | 95.9 | 1.1 | n.d. | n.d. | 2.2 | n.d. | trace |
| fisher2 761.7 | A25-01979-41 | 98.4 | 1.2 | n.d. | n.d. | n.d. | n.d. | n.d. |
| fisher 2 762.6 | A25-01979-42 | 98.1 | 1.2 | n.d. | n.d. | n.d. | n.d. | n.d. |
| fisher2 766.5 | A25-01979-43 | 97.6 | 1.2 | n.d. | n.d. | 0.5 | n.d. | trace |
| fisher2 769.4 | A25-01979-44 | 98.0 | 1.3 | n.d. | n.d. | n.d. | n.d. | n.d. |
| fisher2 771 | A25-01979-45 | 92.3 | 1.0 | n.d. | n.d. | 5.0 | n.d. | 1.1 |
| fisher2 771.45 | A25-01979-46 | 98.3 | 1.3 | n.d. | n.d. | n.d. | n.d. | n.d. |
| fisher2 774.8 | A25-01979-47 | 94.9 | 1.0 | n.d. | n.d. | 4.1 | n.d. | n.d. |
| fisher2 778 | A25-01979-48 | 98.2 | 1.1 | n.d. | n.d. | n.d. | n.d. | n.d. |
| fisher2 776.5 | A25-01979-49 | 95.5 | 1.1 | n.d. | n.d. | 3.4 | n.d. | trace |
| fisher2 780.5 | A25-01979-50 | 96.1 | 1.2 | n.d. | n.d. | 2.0 | n.d. | 0.4 |
| fisher2 783.48 | A25-01979-51 | 98.0 | 1.1 | n.d. | n.d. | 0.9 | n.d. | n.d. |
| fisher2 788 | A25-01979-52 | 98.4 | 1.2 | n.d. | n.d. | n.d. | n.d. | n.d. |
| fisher2 792.25 | A25-01979-53 | 97.9 | 1.1 | n.d. | n.d. | 0.3 | n.d. | n.d. |
| fisher2 793.25 | A25-01979-54 | 98.2 | 1.2 | n.d. | n.d. | n.d. | n.d. | n.d. |
| fisher2 790.9 | A25-01979-55 | 97.3 | 1.1 | n.d. | n.d. | 1.1 | n.d. | trace |

| Client ID | Actlabs ID | Muscovite | Chlorite | Smectite | K feldspar | Dolomite | Calcite |
|---|---|---|---|---|---|---|---|
| Fisher2 612.14 | A25-01979-1 | 11.3 | 3.6 | present | 10.4 | 58.0 | n.d. |
| Fisher2 614.38 | A25-01979-2 | 9.7 | 2.5 | n.d. | 6.5 | 40.5 | n.d. |
| Fisher2 616.75 | A25-01979-3 | 4.1 | 1.7 | trace | n.d. | 0.7 | n.d. |
| Fisher2 654.35 | A25-01979-4 | n.d. | n.d. | n.d. | n.d. | 0.7 | n.d. |
| Fisher2 626.50 | A25-01979-5 | n.d. | n.d. | n.d. | n.d. | n.d. | n.d. |
| Fisher2 634.45 | A25-01979-6 | n.d. | n.d. | n.d. | n.d. | 1.0 | n.d. |
| Fisher2 643.65 | A25-01979-7 | n.d. | n.d. | n.d. | n.d. | 0.8 | n.d. |
| Fisher2 652.30 | A25-01979-8 | n.d. | n.d. | n.d. | n.d. | 0.6 | n.d. |
| Fisher2 653.50 | A25-01979-9 | n.d. | n.d. | n.d. | n.d. | 0.5 | n.d. |
| fisher2 666.4 | A25-01979-10 | trace | trace | n.d. | n.d. | 0.4 | n.d. |
| fisher2 669.2 | A25-01979-11 | n.d. | n.d. | n.d. | n.d. | 0.4 | n.d. |
| fisher2 672.45 | A25-01979-12 | n.d. | n.d. | n.d. | n.d. | 0.7 | n.d. |
| fisher2 674.95 | A25-01979-13 | trace | trace | n.d. | n.d. | 4.7 | n.d. |
| fisher2  676.85 | A25-01979-14 | trace | n.d. | n.d. | n.d. | 0.3 | n.d. |
| fisher2 678.03 | A25-01979-15 | n.d. | n.d. | n.d. | n.d. | n.d. | n.d. |
| fisher2 699.3 | A25-01979-16 | n.d. | n.d. | n.d. | n.d. | n.d. | n.d. |
| fisher2 687.70 | A25-01979-17 | n.d. | n.d. | n.d. | n.d. | n.d. | n.d. |
| fisher2 687.3 | A25-01979-18 | n.d. | n.d. | n.d. | n.d. | n.d. | n.d. |
| fisher2 696.6 | A25-01979-19 | 0.7 | trace | n.d. | n.d. | 2.8 | 94.5 |
| fisher2 691.8 | A25-01979-20 | n.d. | n.d. | n.d. | n.d. | n.d. | 0.6 |
| fisher2 703.5 | A25-01979-21 | n.d. | n.d. | n.d. | n.d. | n.d. | 0.4 |
| fisher2 704.25 | A25-01979-22 | n.d. | n.d. | n.d. | n.d. | n.d. | 0.3 |
| fisher2 707.35 | A25-01979-23 | 2.9 | 0.5 | n.d. | n.d. | 68.7 | n.d. |
| fisher2 710.1 | A25-01979-24 | 6.3 | 1.4 | n.d. | n.d. | 52.8 | n.d. |
| fisher2 710.83 | A25-01979-25 | trace | trace | n.d. | n.d. | n.d. | n.d. |
| fisher2 719 | A25-01979-26 | n.d. | n.d. | n.d. | n.d. | n.d. | n.d. |
| fisher2 719.6 | A25-01979-27 | trace | trace | n.d. | n.d. | 0.5 | n.d. |
| fisher2 720.85 | A25-01979-28 | trace | n.d. | n.d. | n.d. | n.d. | n.d. |
| fisher2 725.30 | A25-01979-29 | n.d. | n.d. | n.d. | n.d. | n.d. | n.d. |

| Client ID | Actlabs ID | Muscovite | Chlorite | Smectite | K feldspar | Dolomite | Calcite |
|---|---|---|---|---|---|---|---|
| fisher2 727.30 | A25-01979-30 | n.d. | n.d. | n.d. | n.d. | 0.4 | n.d. |
| fisher2 728.1 | A25-01979-31 | n.d. | n.d. | n.d. | n.d. | n.d. | n.d. |
| fisher2 730.35 | A25-01979-32 | n.d. | n.d. | n.d. | n.d. | n.d. | n.d. |
| fisher2 734.4 | A25-01979-33 | n.d. | n.d. | n.d. | n.d. | n.d. | n.d. |
| fisher2 733.8 | A25-01979-34 | n.d. | n.d. | n.d. | n.d. | 0.7 | n.d. |
| fisher2 738.75 | A25-01979-35 | n.d. | n.d. | n.d. | n.d. | n.d. | n.d. |
| fisher2 747.75 | A25-01979-36 | n.d. | n.d. | n.d. | n.d. | n.d. | n.d. |
| fisher2 745.6 | A25-01979-37 | n.d. | n.d. | n.d. | n.d. | n.d. | n.d. |
| fisher2 749.0 | A25-01979-38 | n.d. | n.d. | n.d. | n.d. | 0.3 | n.d. |
| fisher2 751.72 | A25-01979-39 | n.d. | n.d. | n.d. | n.d. | n.d. | n.d. |
| fisher2 757.55 | A25-01979-40 | n.d. | n.d. | n.d. | n.d. | 0.8 | n.d. |
| fisher2 761.7 | A25-01979-41 | n.d. | n.d. | n.d. | n.d. | 0.4 | n.d. |
| fisher 2 762.6 | A25-01979-42 | n.d. | n.d. | n.d. | n.d. | 0.7 | n.d. |
| fisher2 766.5 | A25-01979-43 | trace | n.d. | n.d. | n.d. | 0.7 | n.d. |
| fisher2 769.4 | A25-01979-44 | n.d. | n.d. | n.d. | n.d. | 0.7 | n.d. |
| fisher2 771 | A25-01979-45 | trace | trace | n.d. | n.d. | 0.6 | n.d. |
| fisher2 771.45 | A25-01979-46 | n.d. | n.d. | n.d. | n.d. | 0.4 | n.d. |
| fisher2 774.8 | A25-01979-47 | n.d. | n.d. | n.d. | n.d. | n.d. | n.d. |
| fisher2 778 | A25-01979-48 | n.d. | n.d. | n.d. | n.d. | 0.7 | n.d. |
| fisher2 776.5 | A25-01979-49 | n.d. | n.d. | n.d. | n.d. | n.d. | n.d. |
| fisher2 780.5 | A25-01979-50 | trace | trace | n.d. | n.d. | 0.3 | n.d. |
| fisher2 783.48 | A25-01979-51 | n.d. | n.d. | n.d. | n.d. | n.d. | n.d. |
| fisher2 788 | A25-01979-52 | n.d. | n.d. | n.d. | n.d. | 0.4 | n.d. |
| fisher2 792.25 | A25-01979-53 | n.d. | n.d. | n.d. | n.d. | 0.7 | n.d. |
| fisher2 793.25 | A25-01979-54 | n.d. | n.d. | n.d. | n.d. | 0.6 | n.d. |
| fisher2 790.9 | A25-01979-55 | n.d. | n.d. | n.d. | n.d. | 0.5 | n.d. |

Note: n.d. = not detected; clay speciation analysis is required to positively identify smectite in sample Fisher2 612.14

**Table 2.** Relative proportions of clay minerals in the < 4 µm size fraction

| Client ID | Actlabs ID | Muscovite/Illite | Chlorite | Kaolinite | Smectite |
|---|---|---|---|---|---|
| Fisher2 616.75 | A25-01979-3 | 59 | 41 | n.d. | trace |
| fisher2 707.35 | A25-01979-23 | 82 | 18 | n.d. | n.d. |
| fisher2 710.1 | A25-01979-24 | 78 | 15 | 7 | n.d. |

Reported by:
Elitsa Hrischeva, PhD
Activation Laboratories Ltd.

# APPENDIX 1

Diffraction Patterns

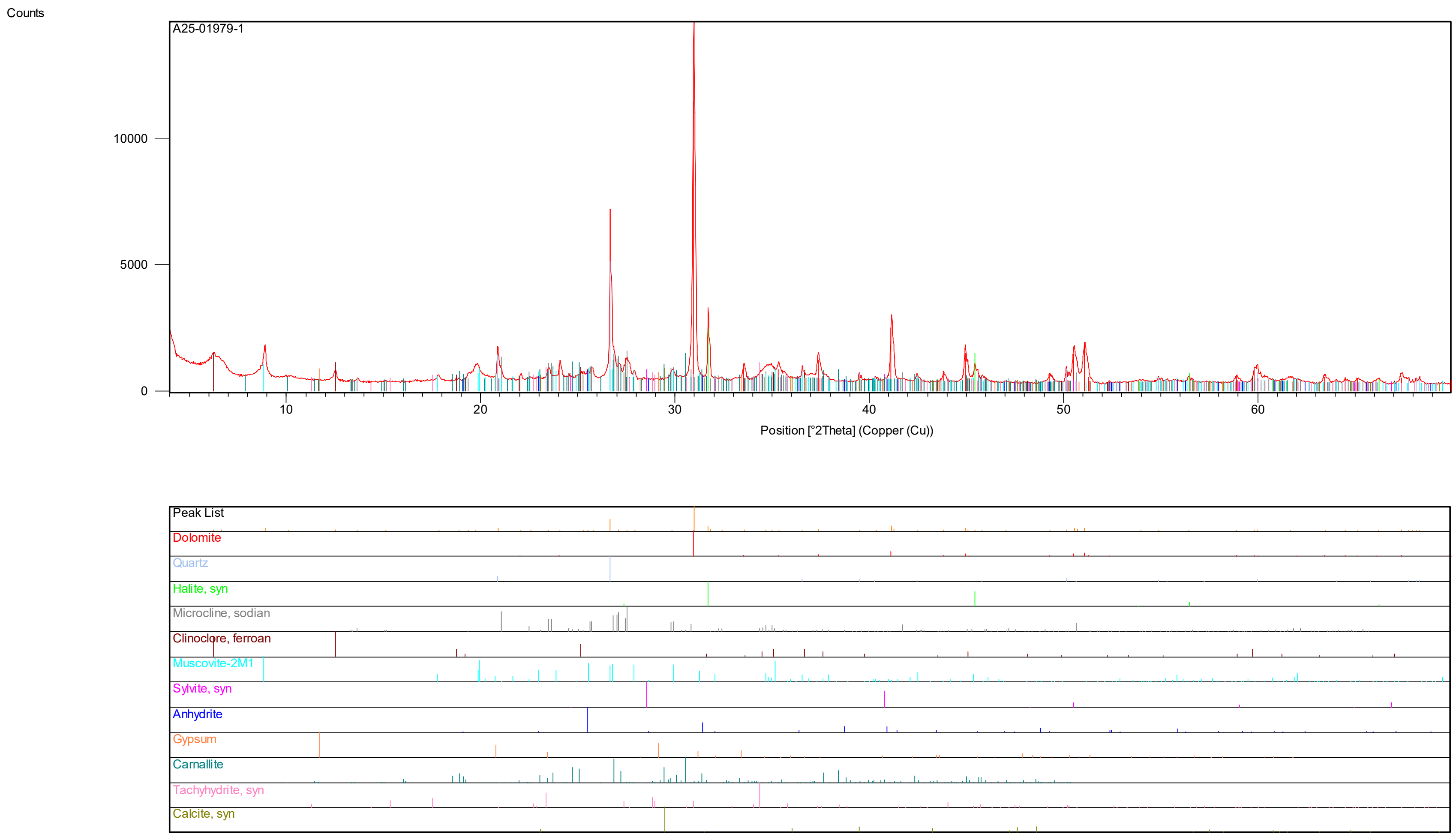
Counts
A25-01979-1
10000
5000
0
10
20
30
40
50
60
Position [°2Theta] (Copper (Cu))
Peak List
Dolomite
Quartz
Halite, syn
Microcline, sodian
Clinoclore, ferroan
Muscovite-2M1
Sylvite, syn
Anhydrite
Gypsum
Carnallite
Tachyhydrite, syn
Calcite, syn

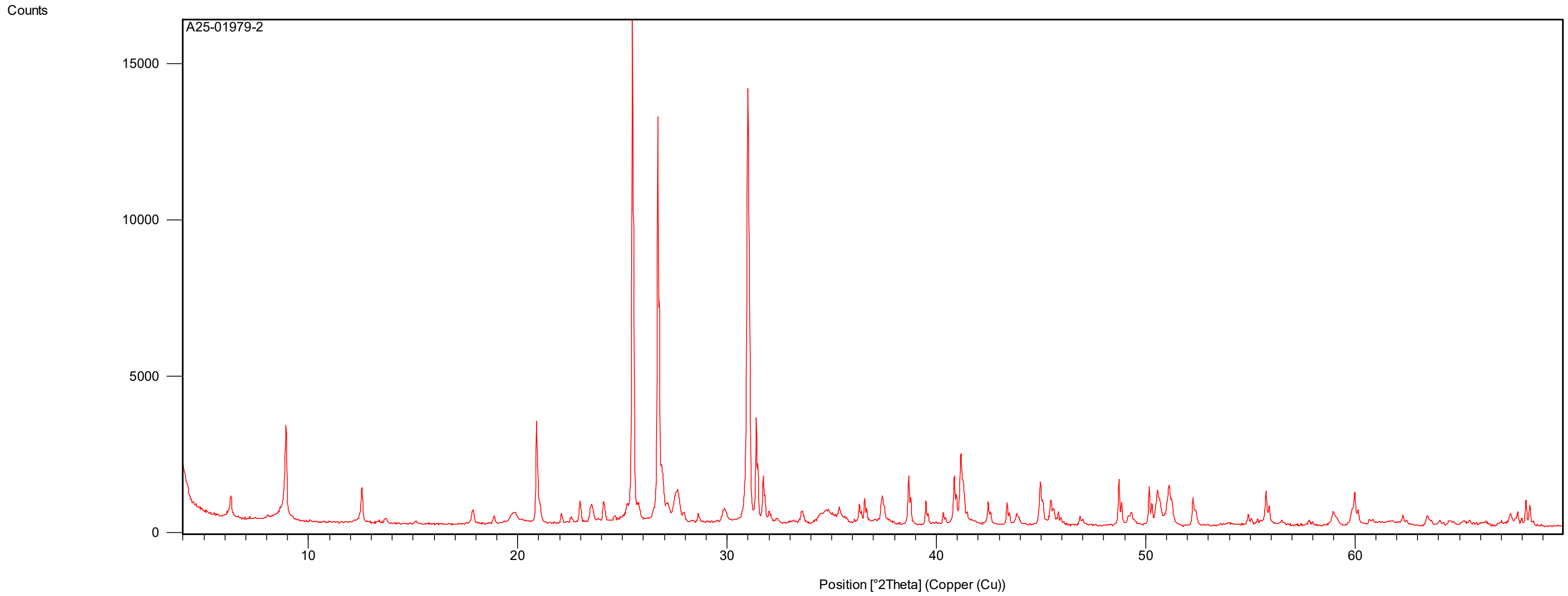
Counts
A25-01979-2
15000
10000
5000
0
10
20
30
40
50
60
Position [°2Theta] (Copper (Cu))

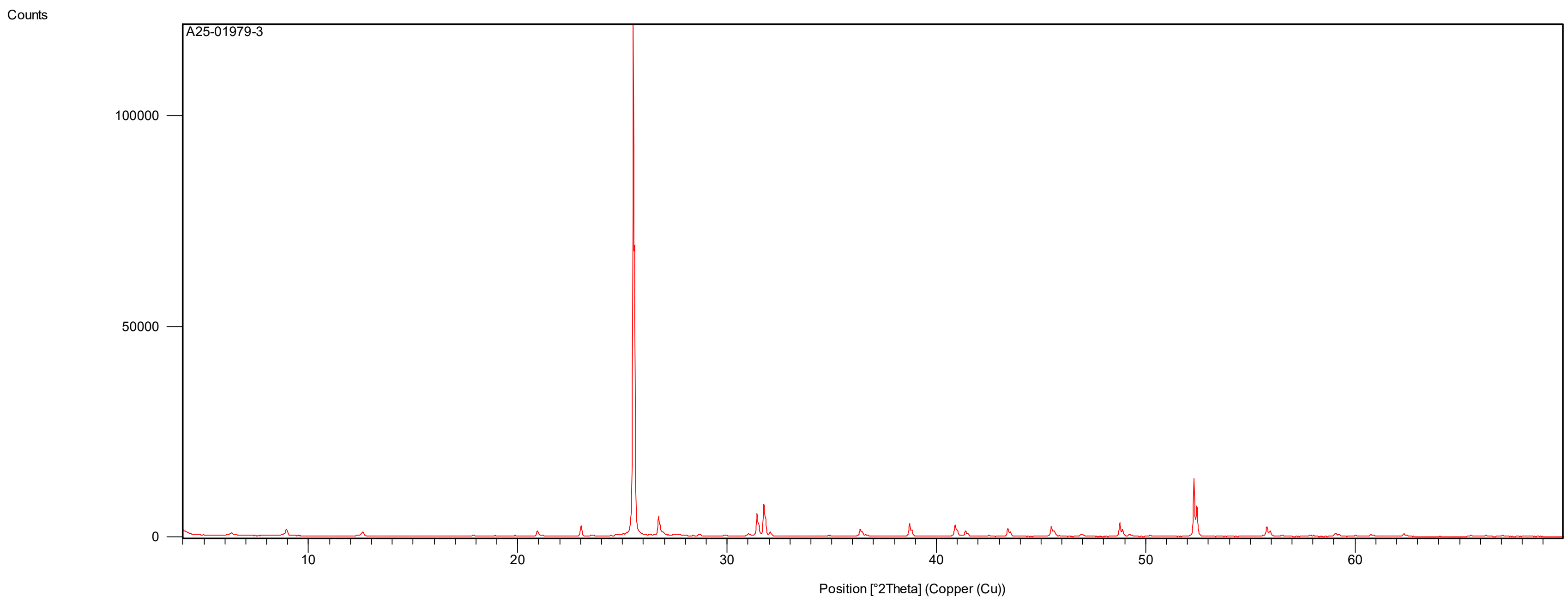
Counts
A25-01979-3
100000
50000
0
10
20
30
40
50
60
Position [°2Theta] (Copper (Cu))

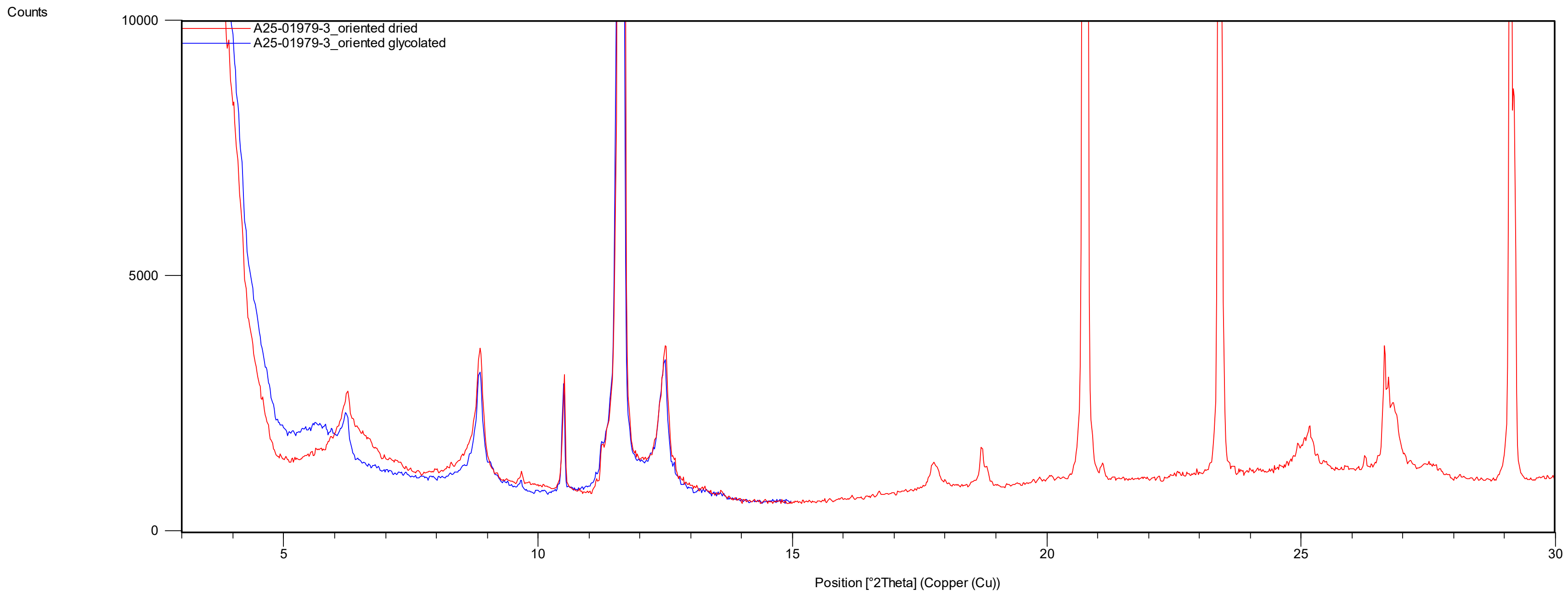
Counts
10000
5000
0
A25-01979-3_oriented dried
A25-01979-3_oriented glycolated
5
10
15
20
25
30
Position [°2Theta] (Copper (Cu))

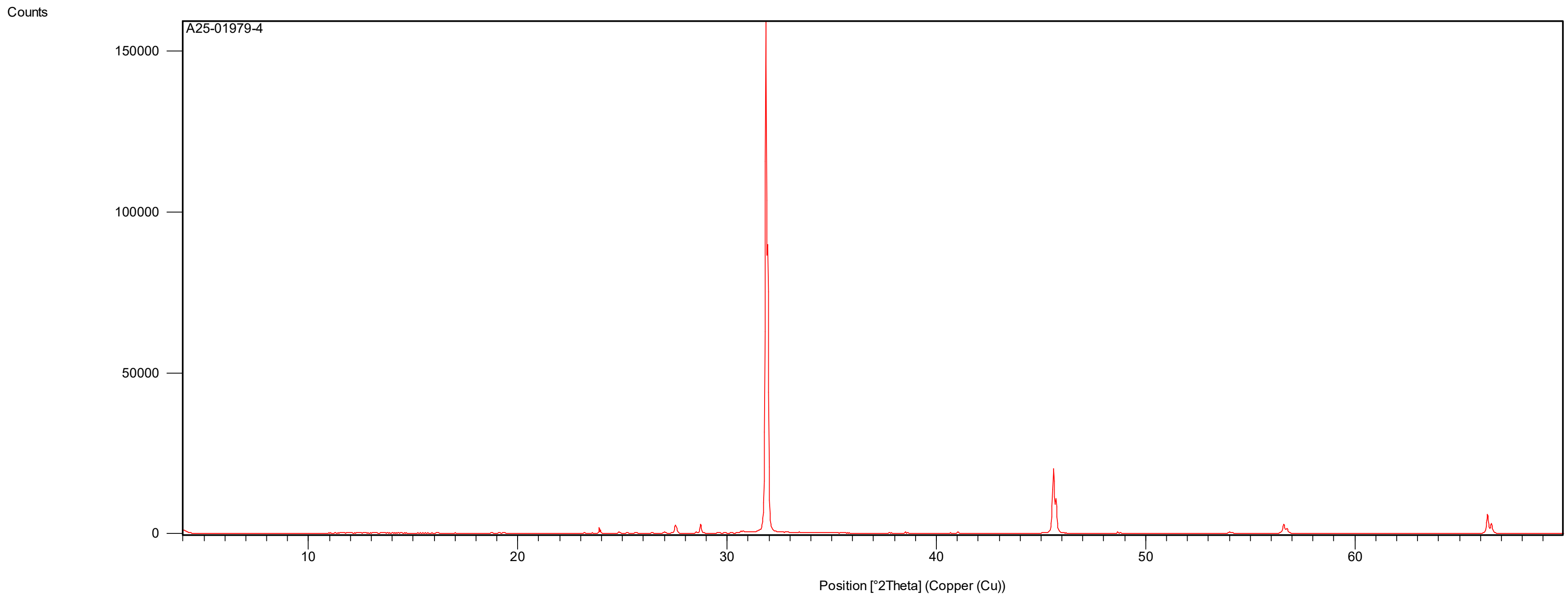
Counts
A25-01979-4
150000
100000
50000
0
10
20
30
40
50
60
Position [°2Theta] (Copper (Cu))

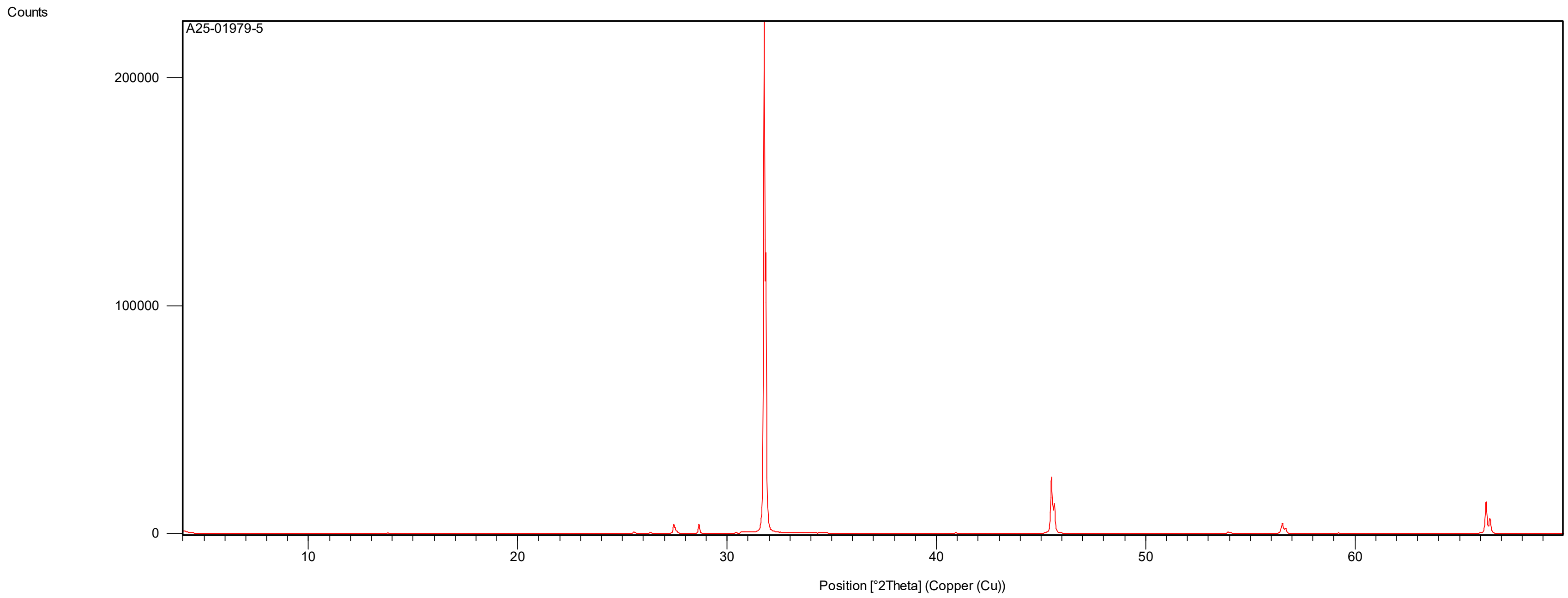
Counts
A25-01979-5
200000
100000
0
10
20
30
40
50
60
Position [°2Theta] (Copper (Cu))

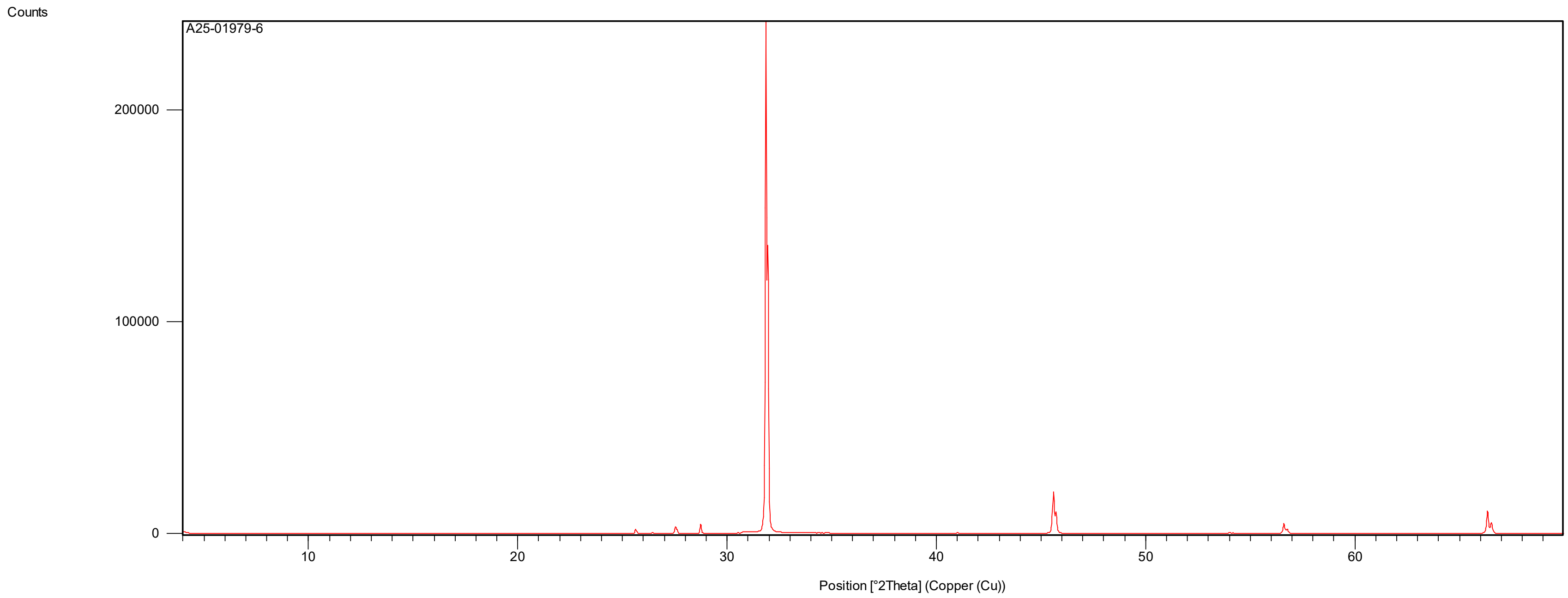
Counts
A25-01979-6
200000
100000
0
10
20
30
40
50
60
Position [°2Theta] (Copper (Cu))

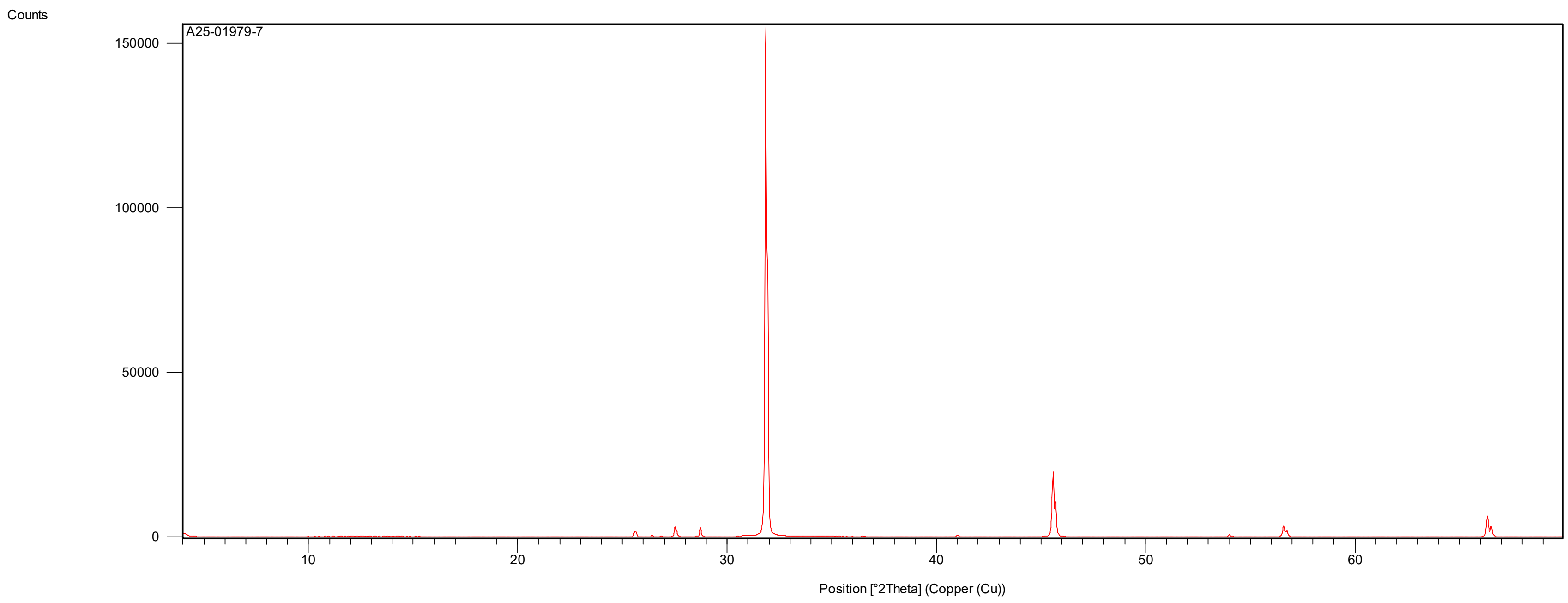
Counts
A25-01979-7
150000
100000
50000
0
10
20
30
40
50
60
Position [°2Theta] (Copper (Cu))

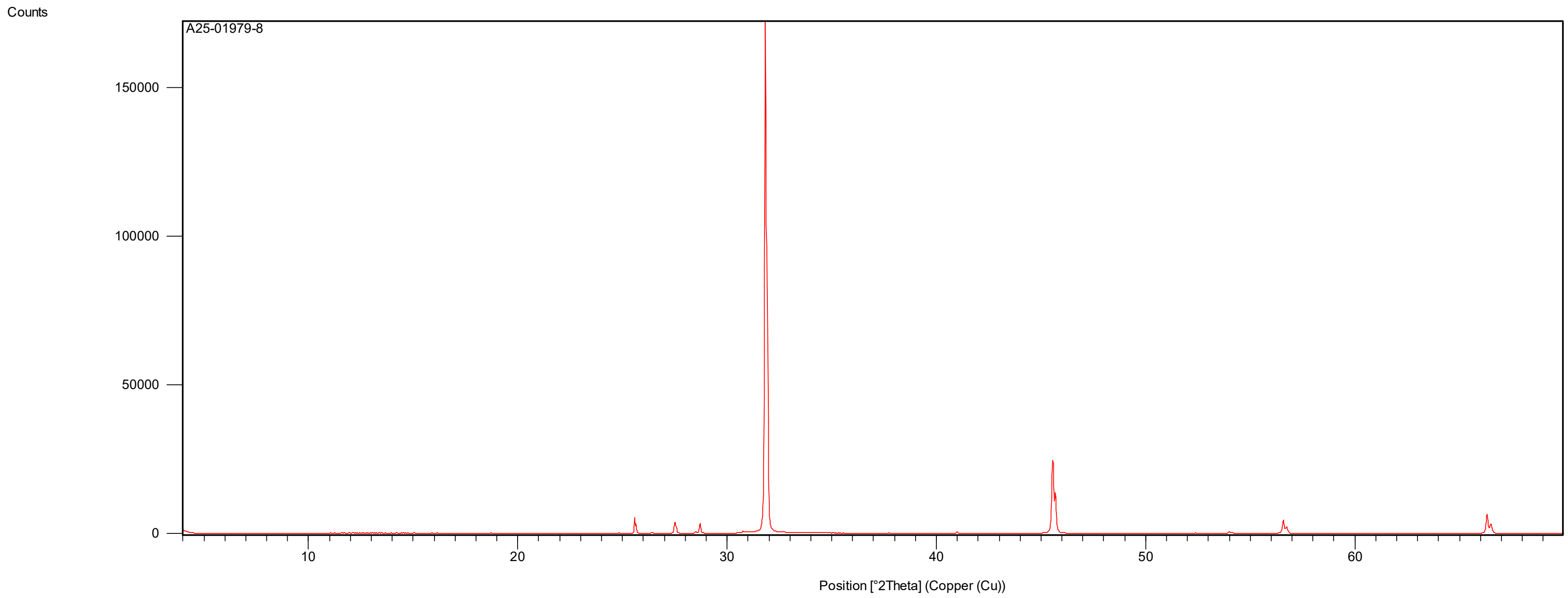
Counts
A25-01979-8
150000
100000
50000
0
10
20
30
40
50
60
Position [°2Theta] (Copper (Cu))

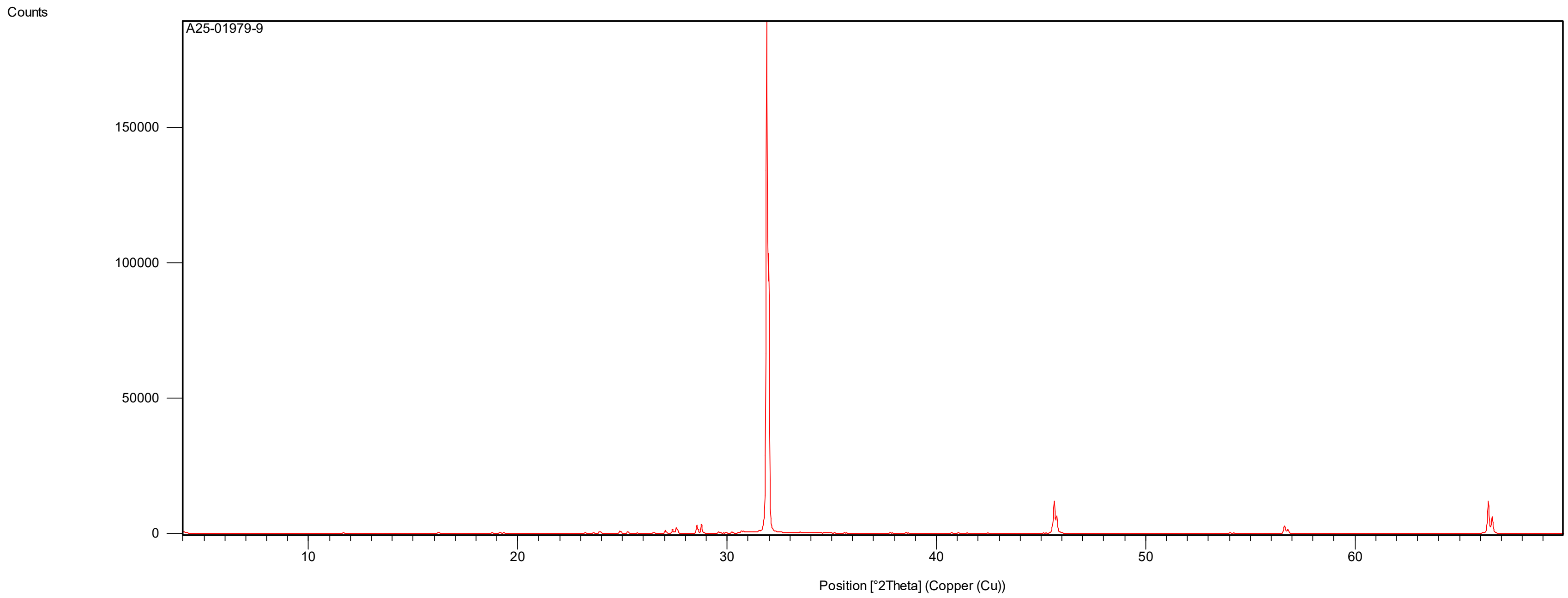
Counts
A25-01979-9
150000
100000
50000
0
10
20
30
40
50
60
Position [°2Theta] (Copper (Cu))

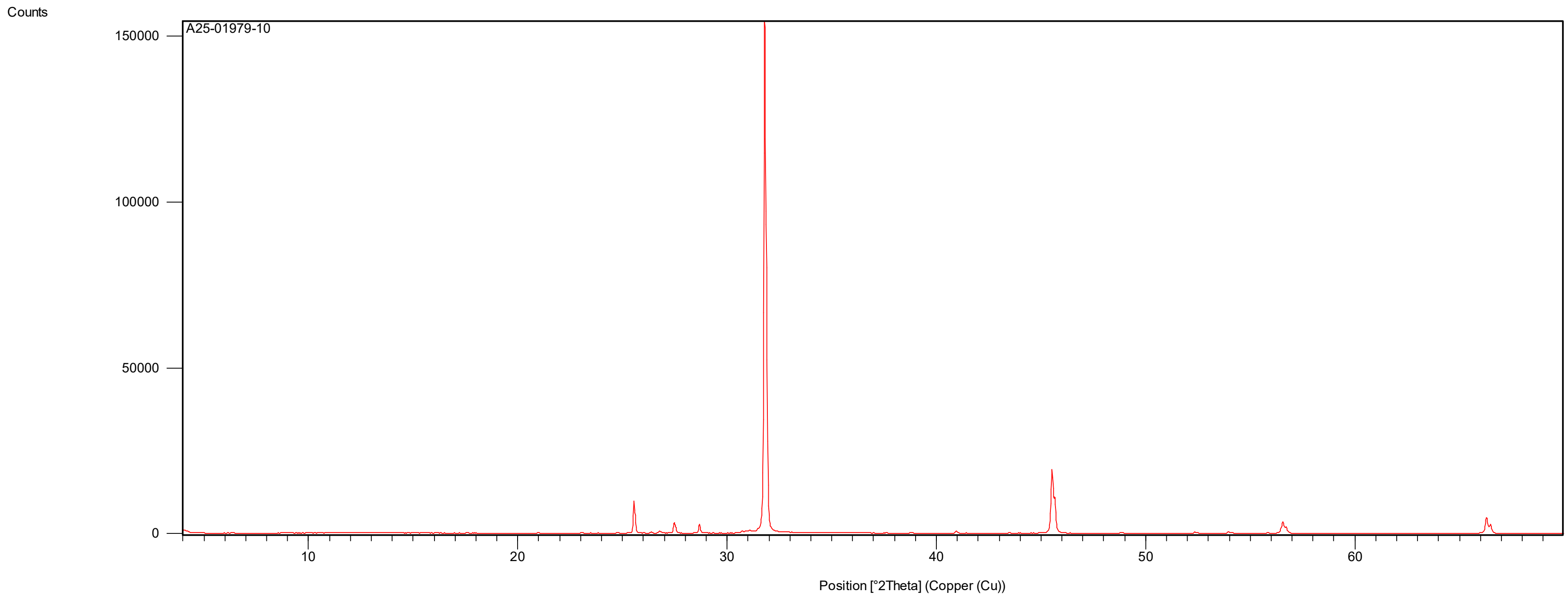
Counts
A25-01979-10
150000
100000
50000
0
10
20
30
40
50
60
Position [°2Theta] (Copper (Cu))

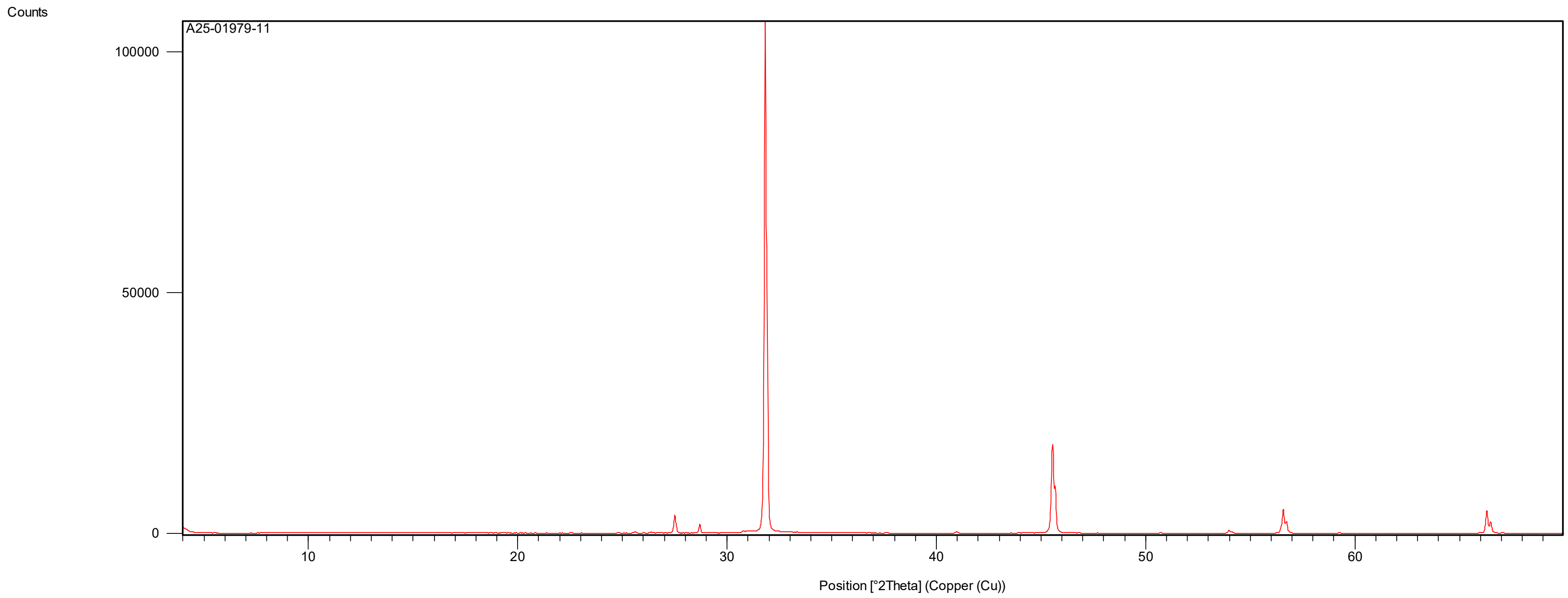
Counts
A25-01979-11
100000
50000
0
10
20
30
40
50
60
Position [°2Theta] (Copper (Cu))

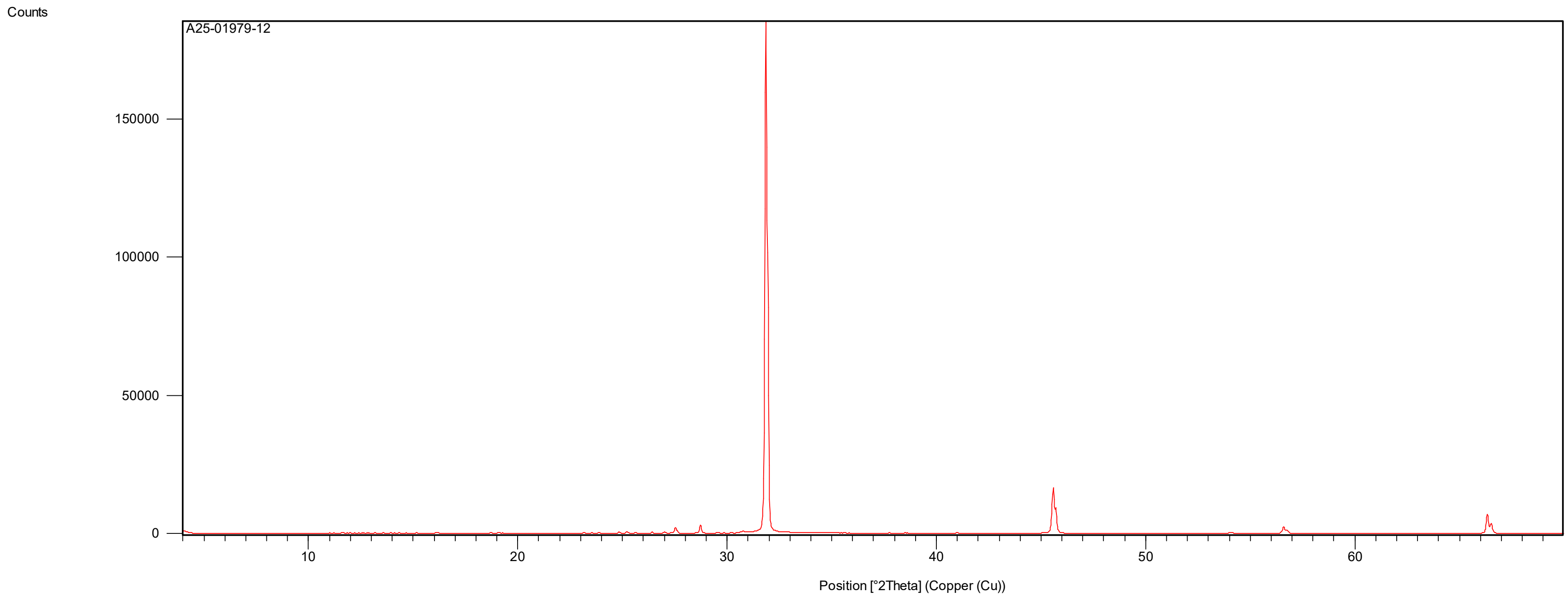
Counts
A25-01979-12
150000
100000
50000
0
10
20
30
40
50
60
Position [°2Theta] (Copper (Cu))

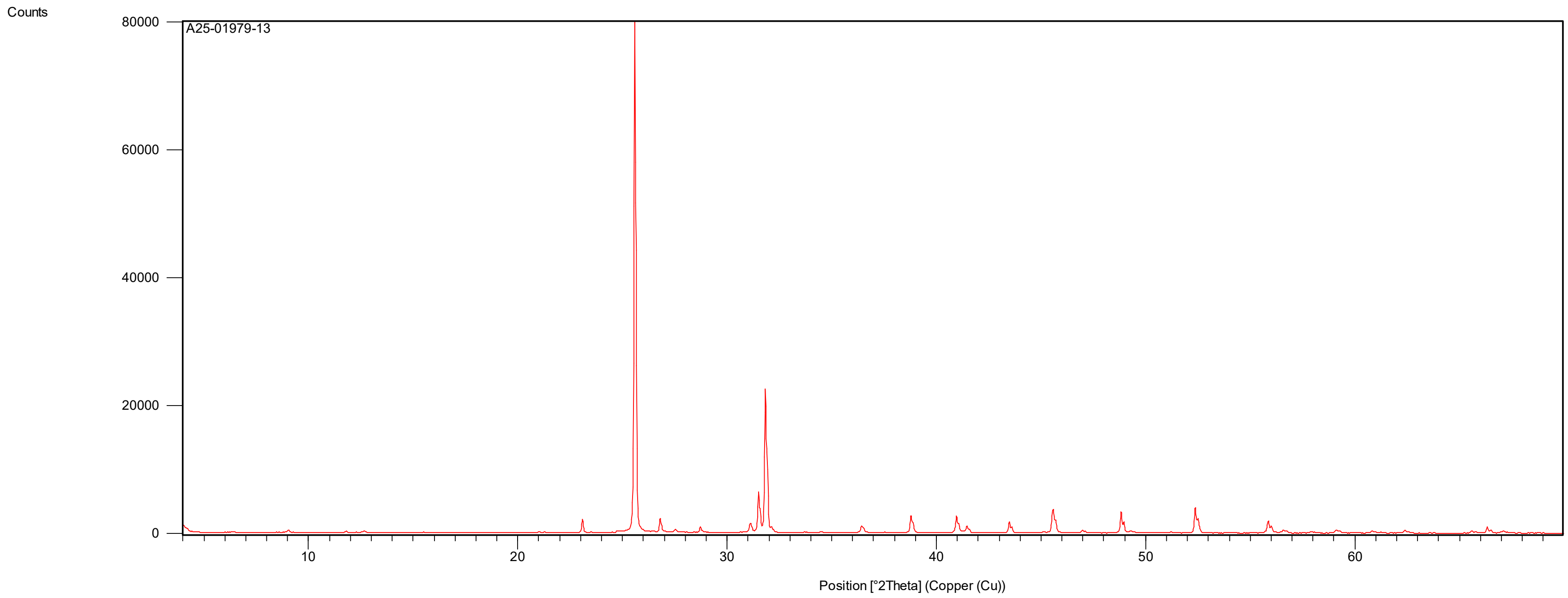
Counts
80000
60000
40000
20000
0
A25-01979-13
10
20
30
40
50
60
Position [°2Theta] (Copper (Cu))

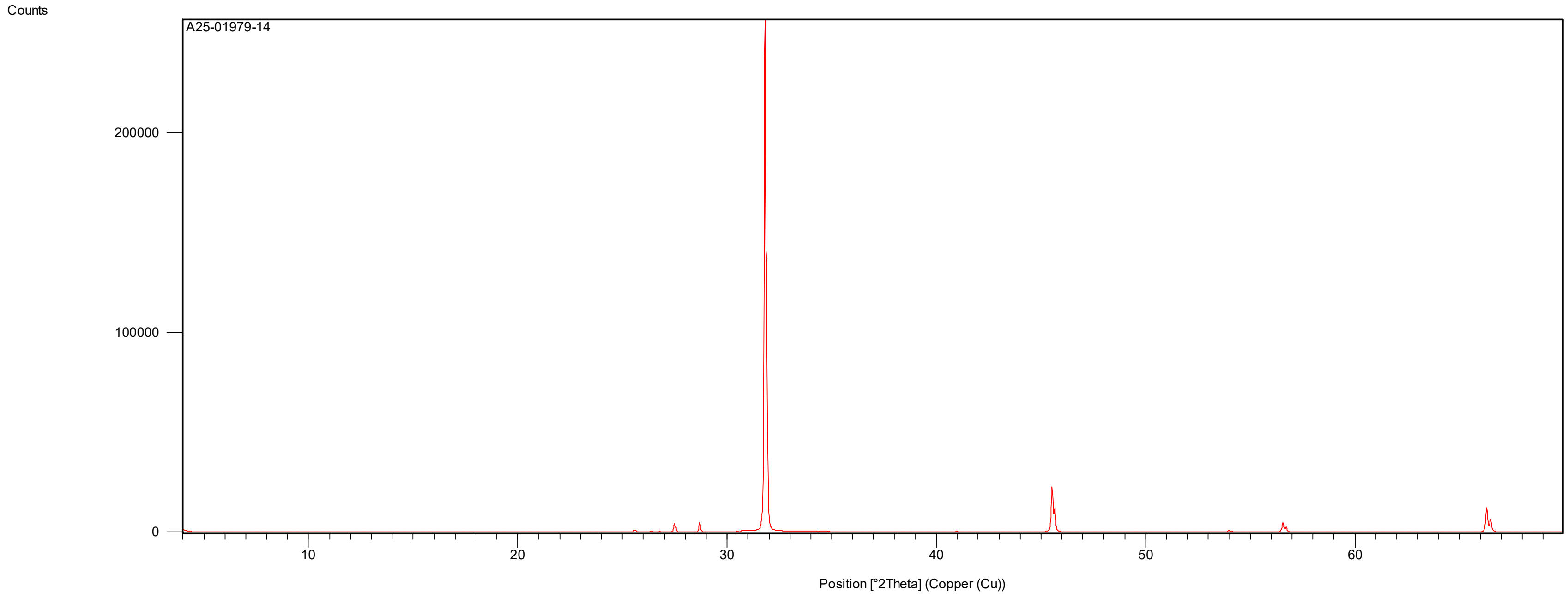
Counts
A25-01979-14
200000
100000
0
10
20
30
40
50
60
Position [°2Theta] (Copper (Cu))

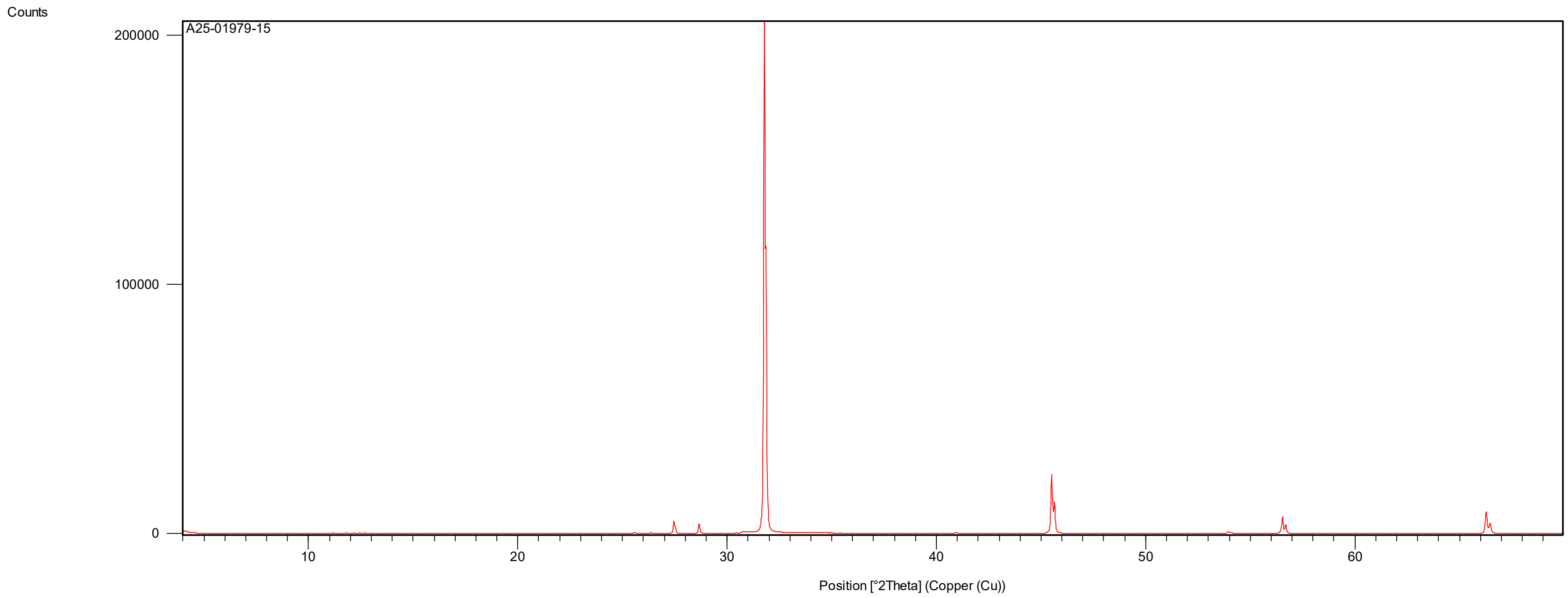

Counts
A25-01979-15
200000
100000
0
10
20
30
40
50
60
Position [°2Theta] (Copper (Cu))

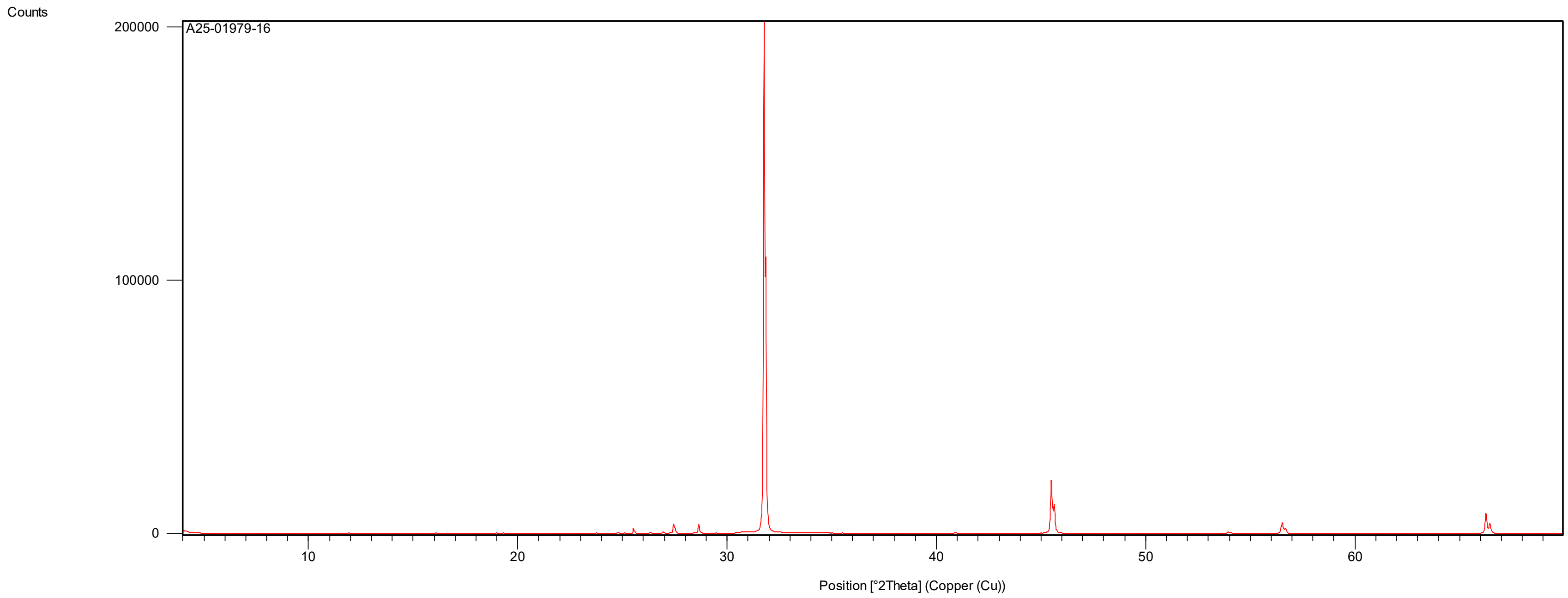
Counts
200000
100000
0
A25-01979-16
10
20
30
40
50
60
Position [°2Theta] (Copper (Cu))

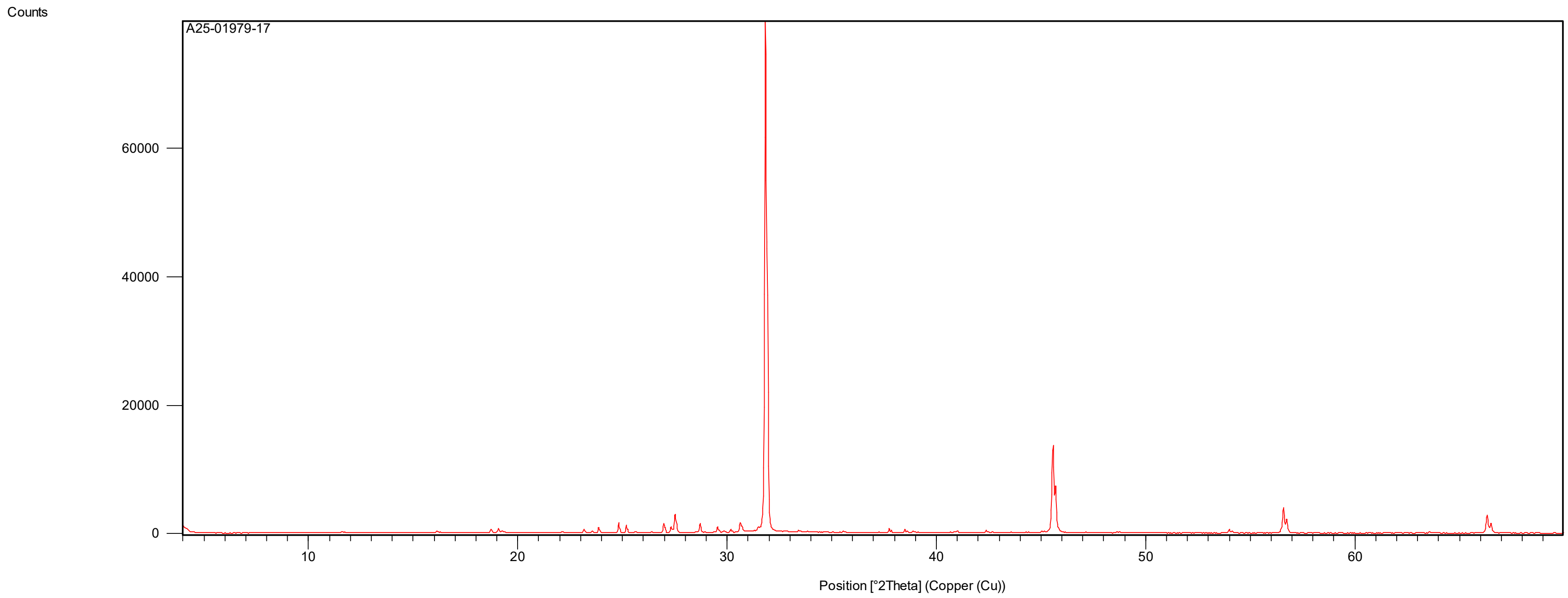
Counts
A25-01979-17
60000
40000
20000
0
10
20
30
40
50
60
Position [°2Theta] (Copper (Cu))

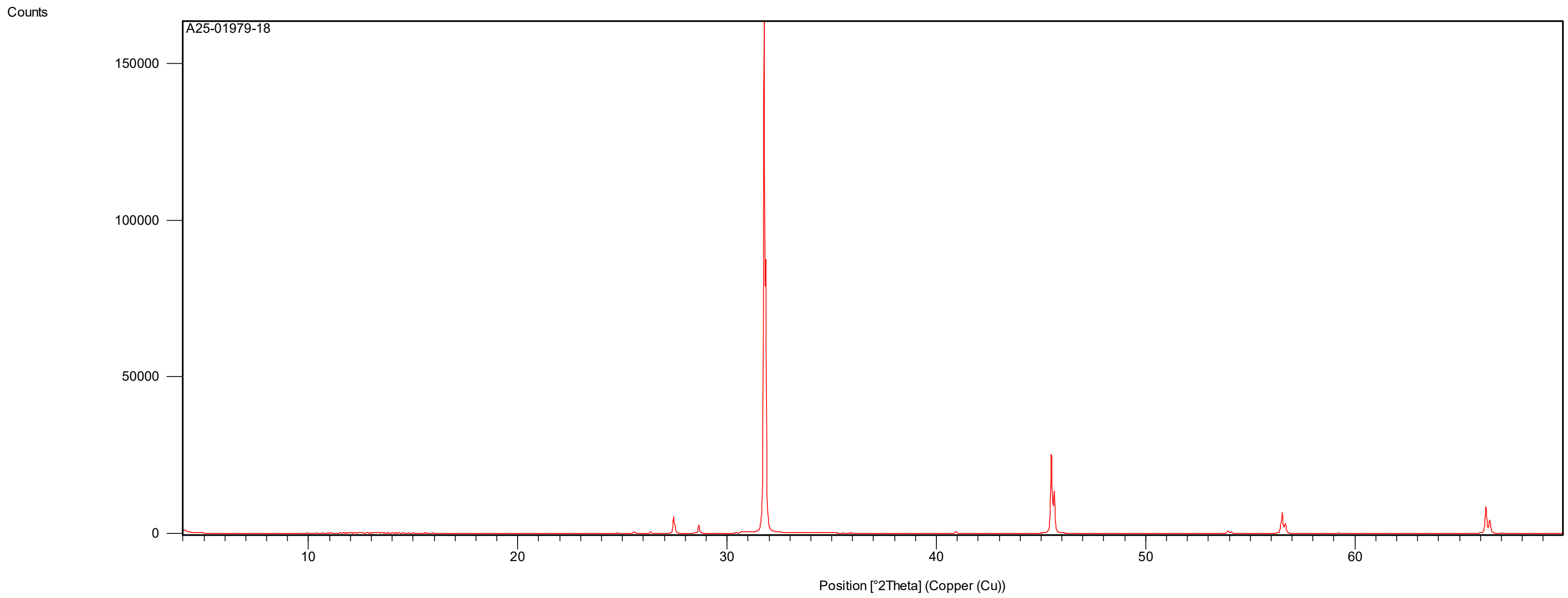
Counts
A25-01979-18
150000
100000
50000
0
10
20
30
40
50
60
Position [°2Theta] (Copper (Cu))

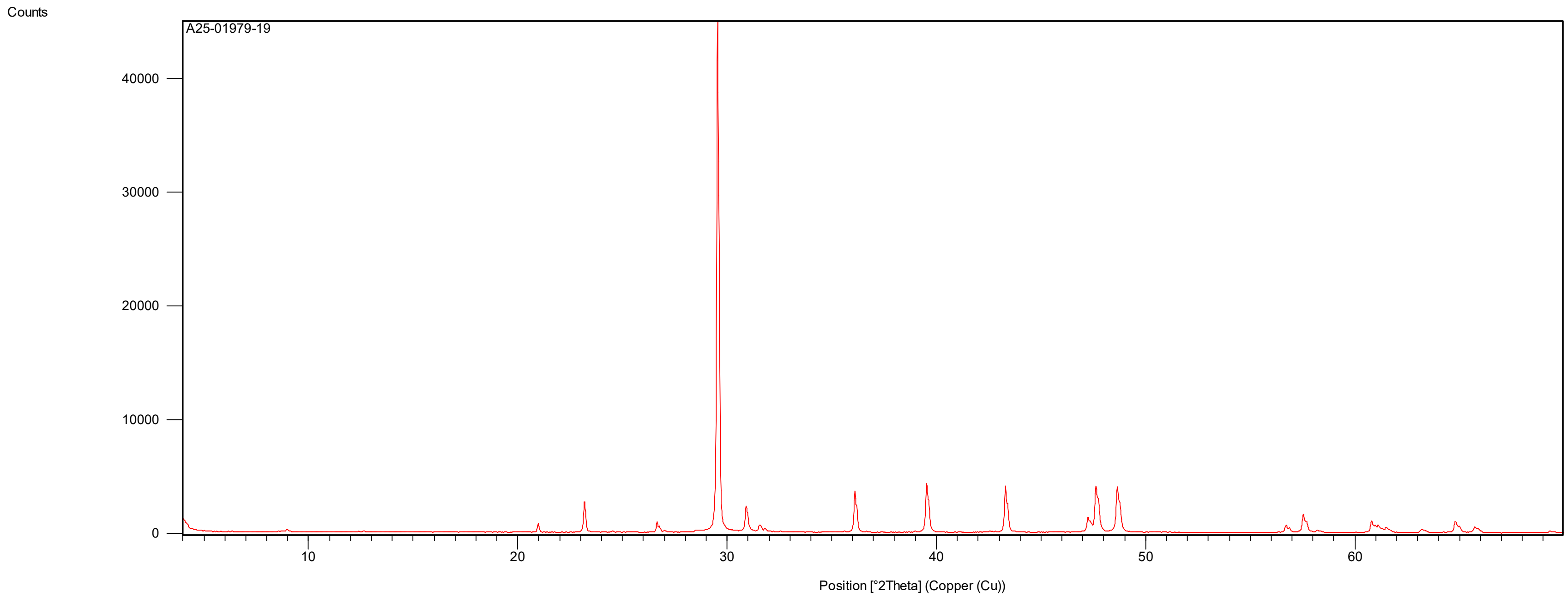
Counts
A25-01979-19
40000
30000
20000
10000
0
10
20
30
40
50
60
Position [°2Theta] (Copper (Cu))

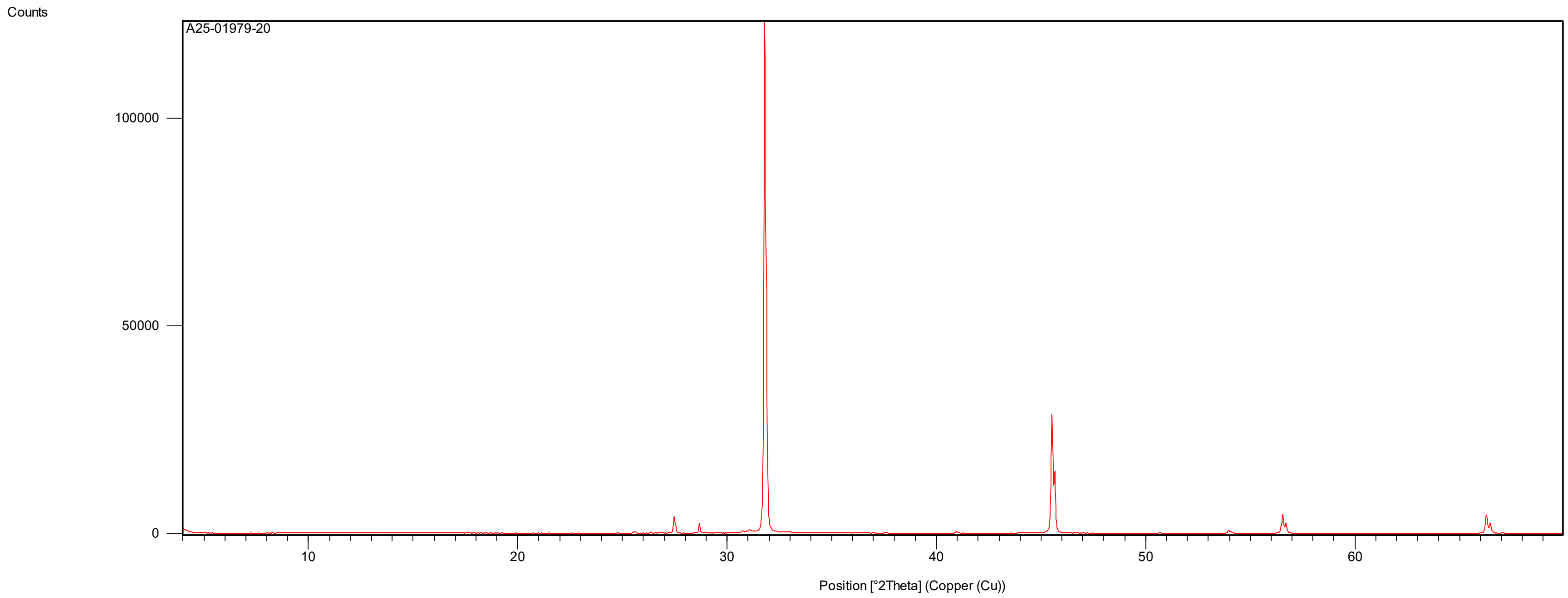
Counts
A25-01979-20
100000
50000
0
10
20
30
40
50
60
Position [°2Theta] (Copper (Cu))

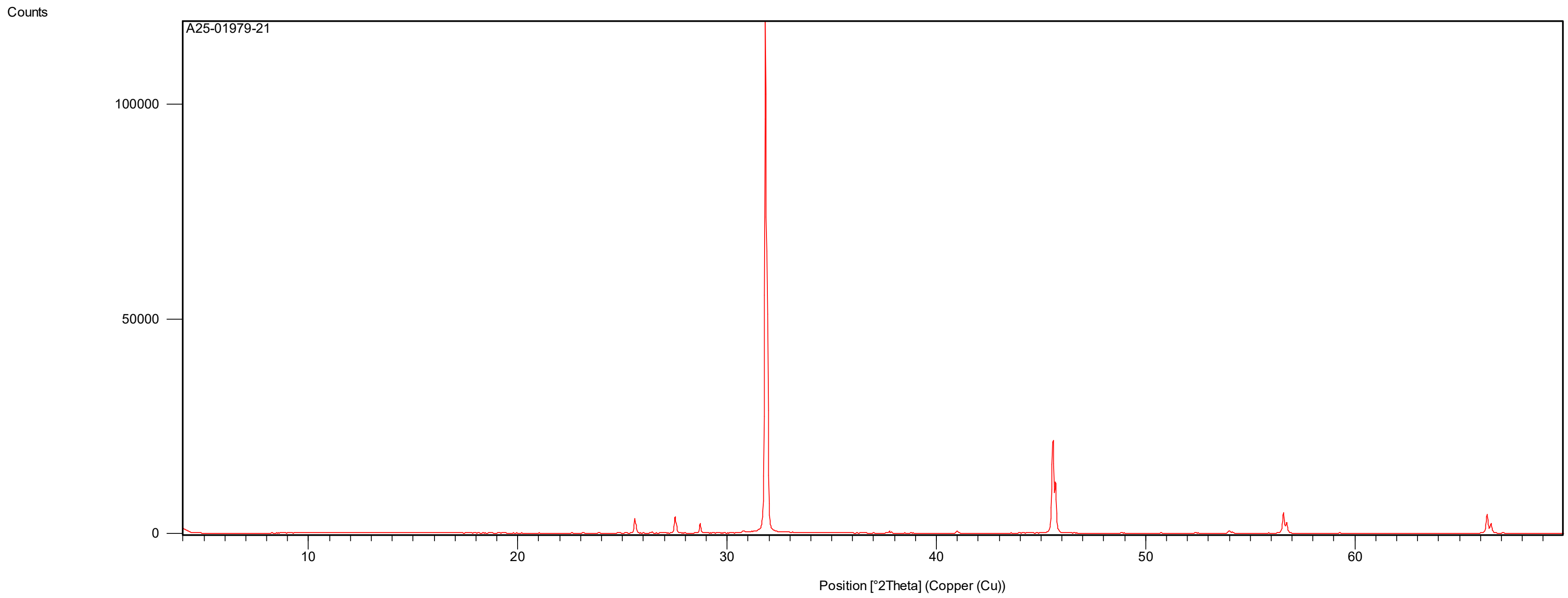
Counts
A25-01979-21
100000
50000
0
10
20
30
40
50
60
Position [°2Theta] (Copper (Cu))

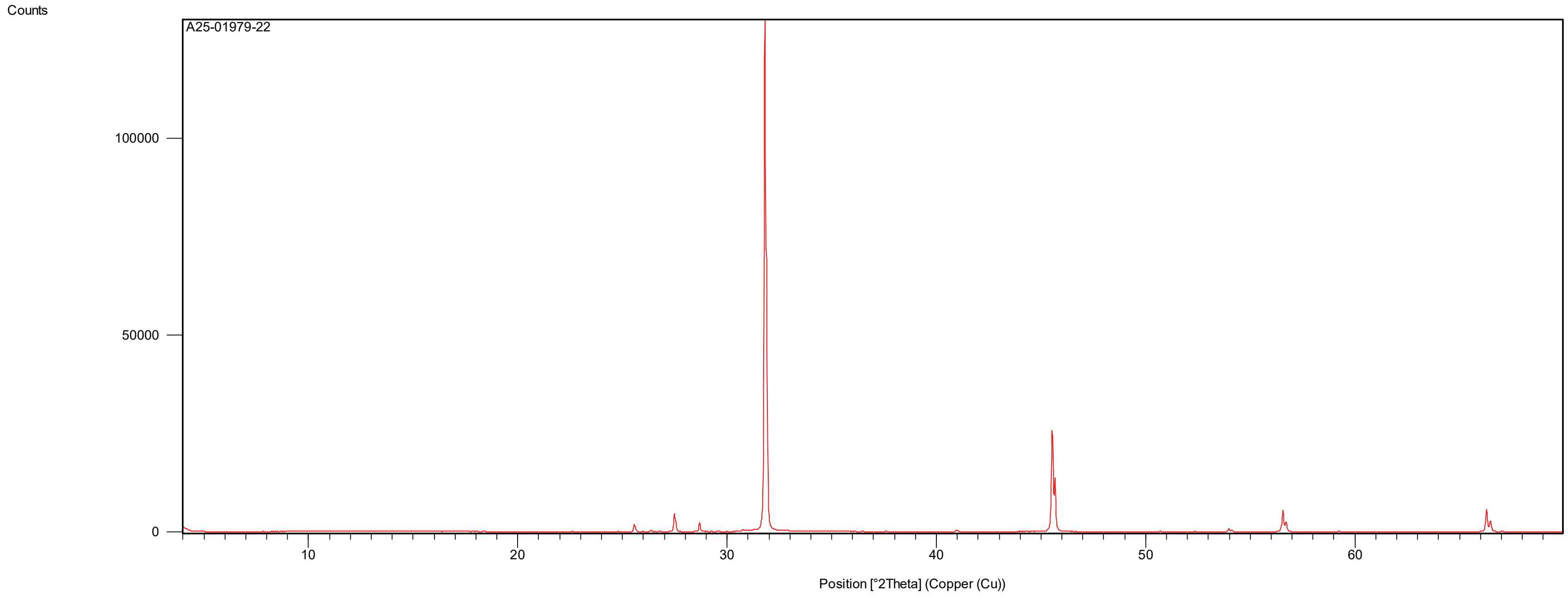
Counts
A25-01979-22
100000
50000
0
10
20
30
40
50
60
Position [°2Theta] (Copper (Cu))

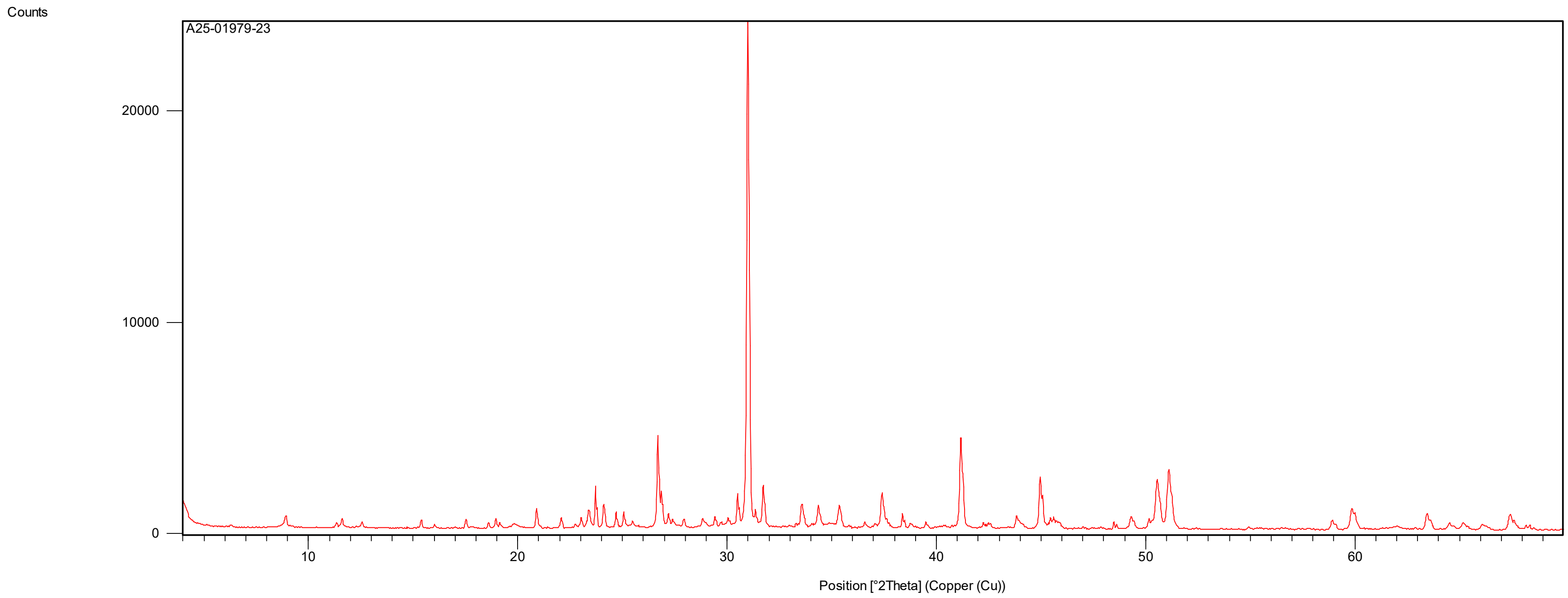
Counts
A25-01979-23
20000
10000
0
10
20
30
40
50
60
Position [°2Theta] (Copper (Cu))

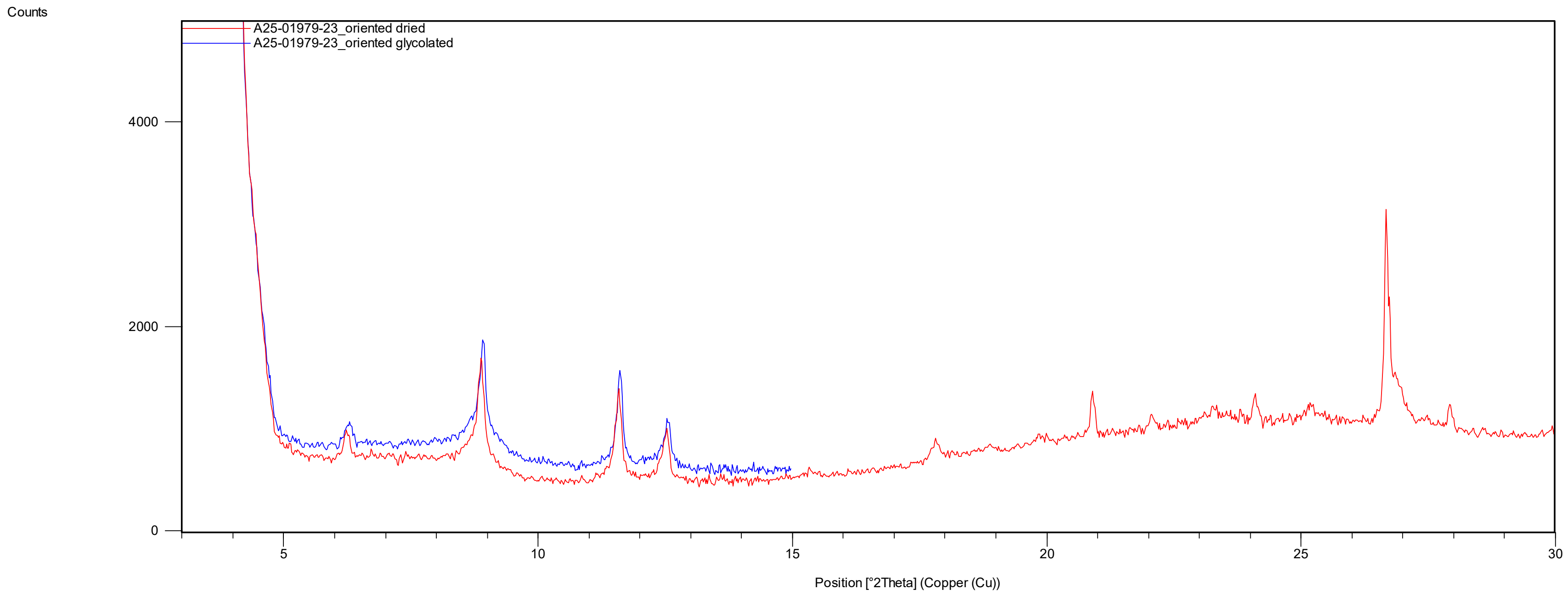
Counts
A25-01979-23_oriented dried
A25-01979-23_oriented glycolated
4000
2000
0
5
10
15
20
25
30
Position [°2Theta] (Copper (Cu))

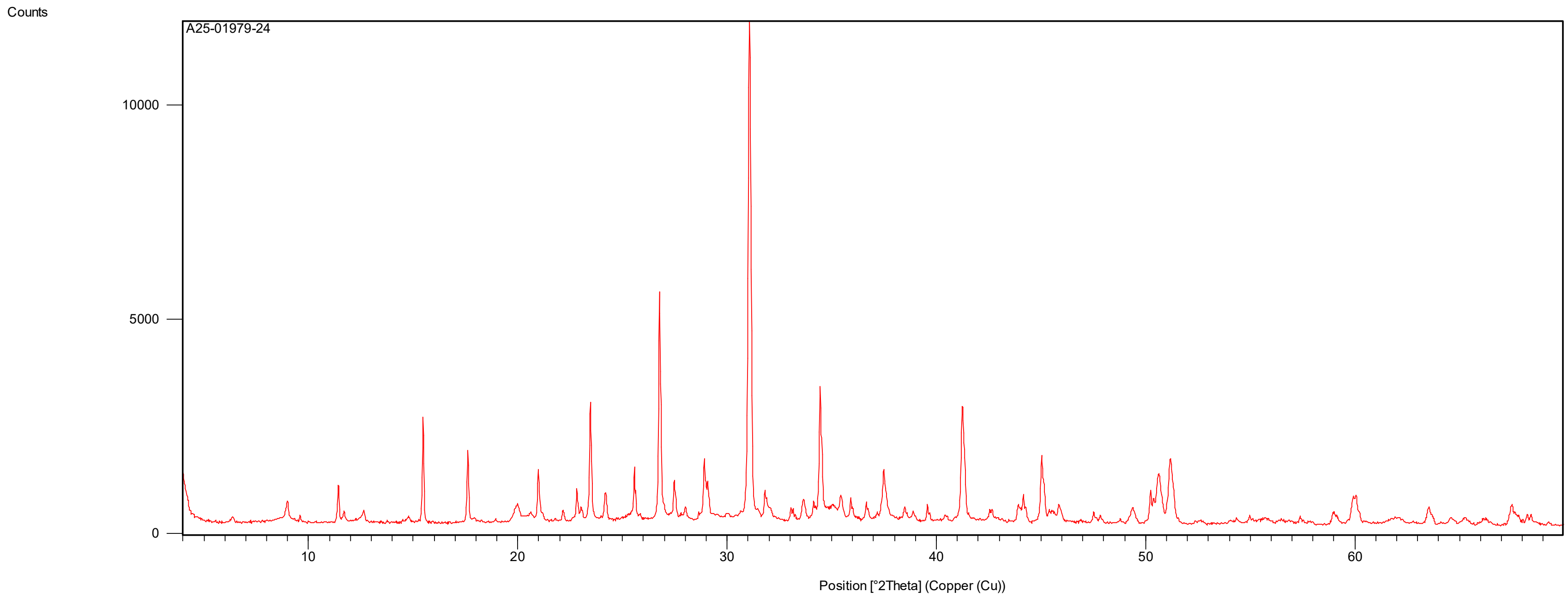
Counts
A25-01979-24
10000
5000
0
10
20
30
40
50
60
Position [°2Theta] (Copper (Cu))

Activation Laboratories Ltd. A25-01979

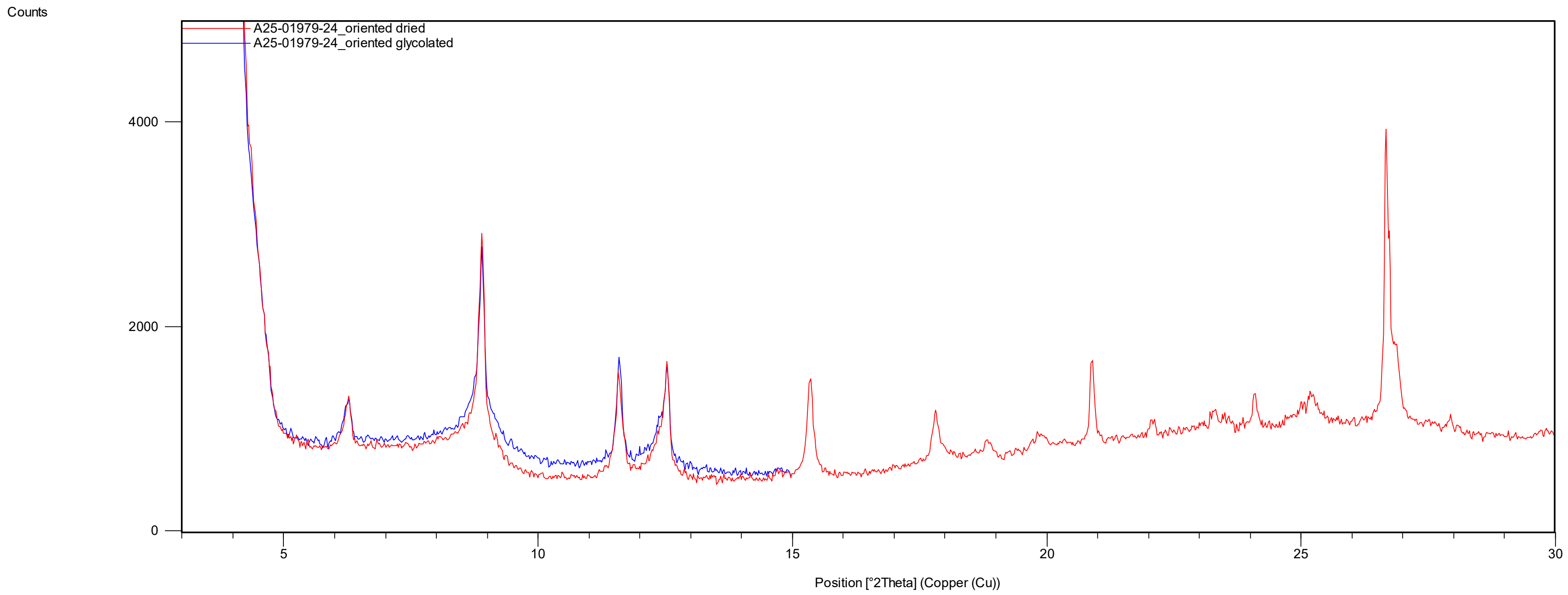

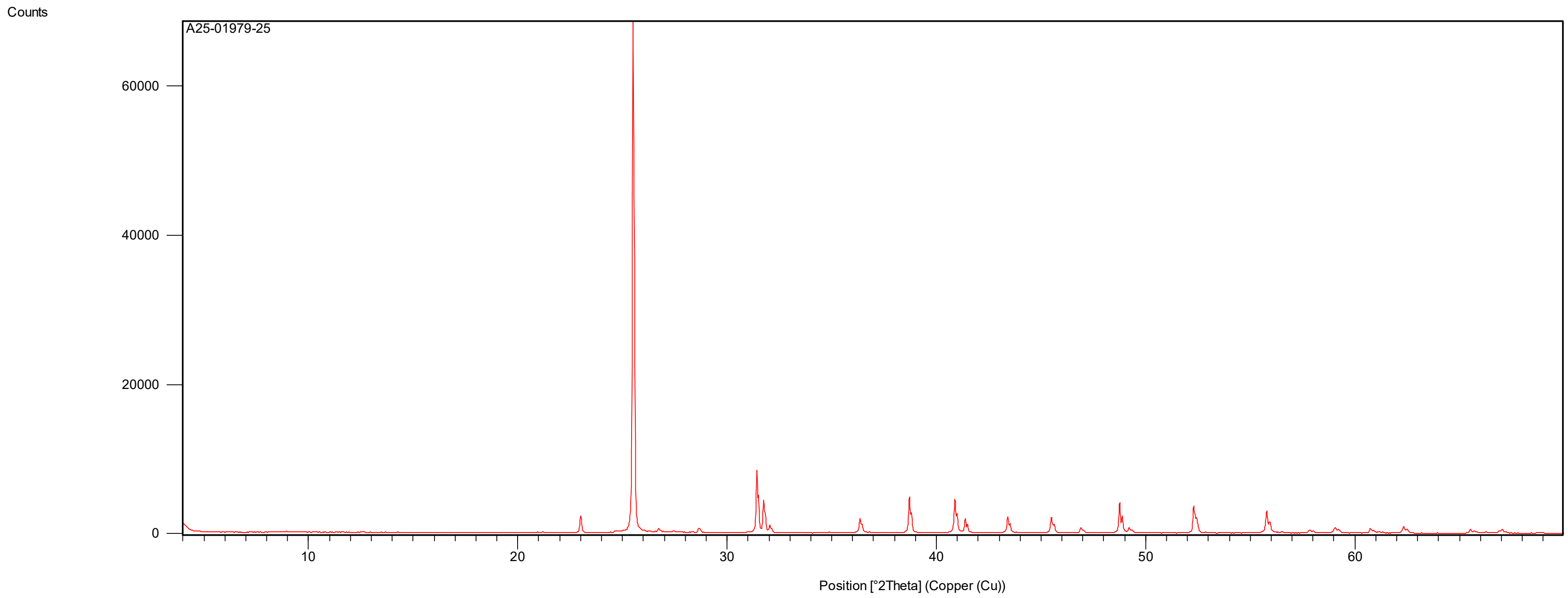
Counts
A25-01979-25
60000
40000
20000
0
10
20
30
40
50
60
Position [°2Theta] (Copper (Cu))

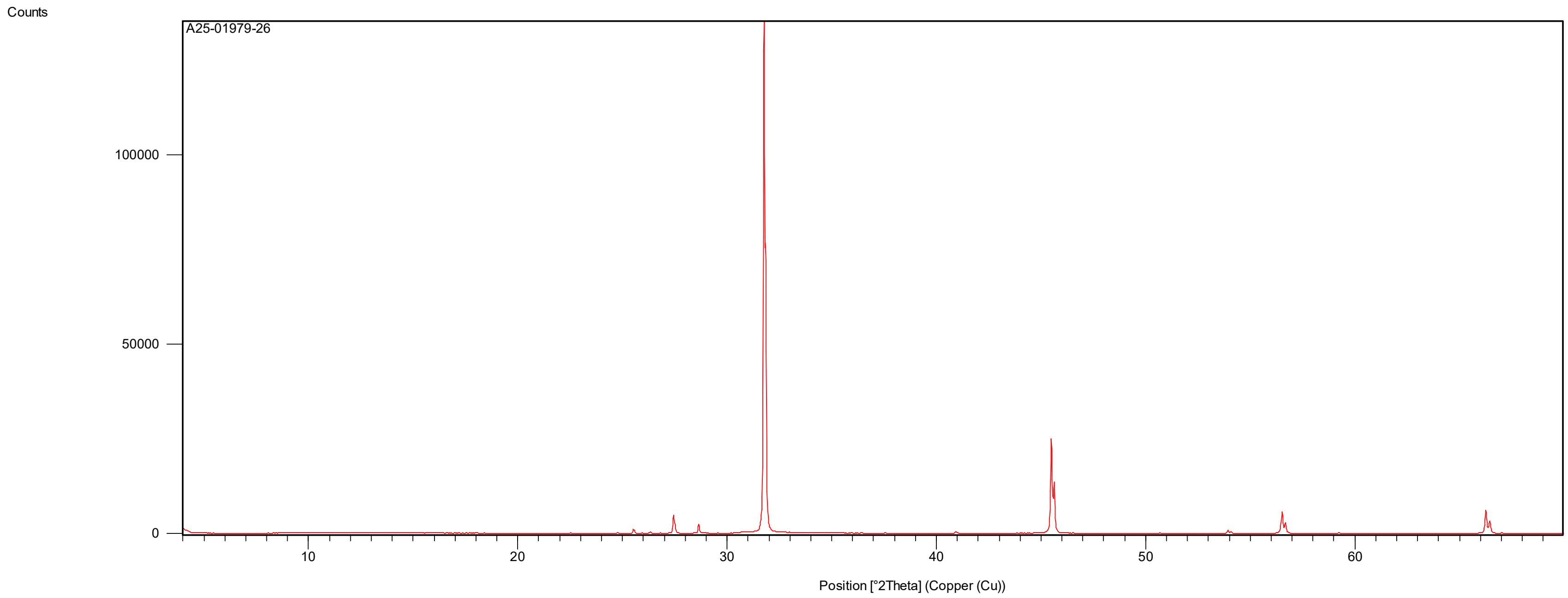
Counts
A25-01979-26
100000
50000
0
10
20
30
40
50
60
Position [°2Theta] (Copper (Cu))

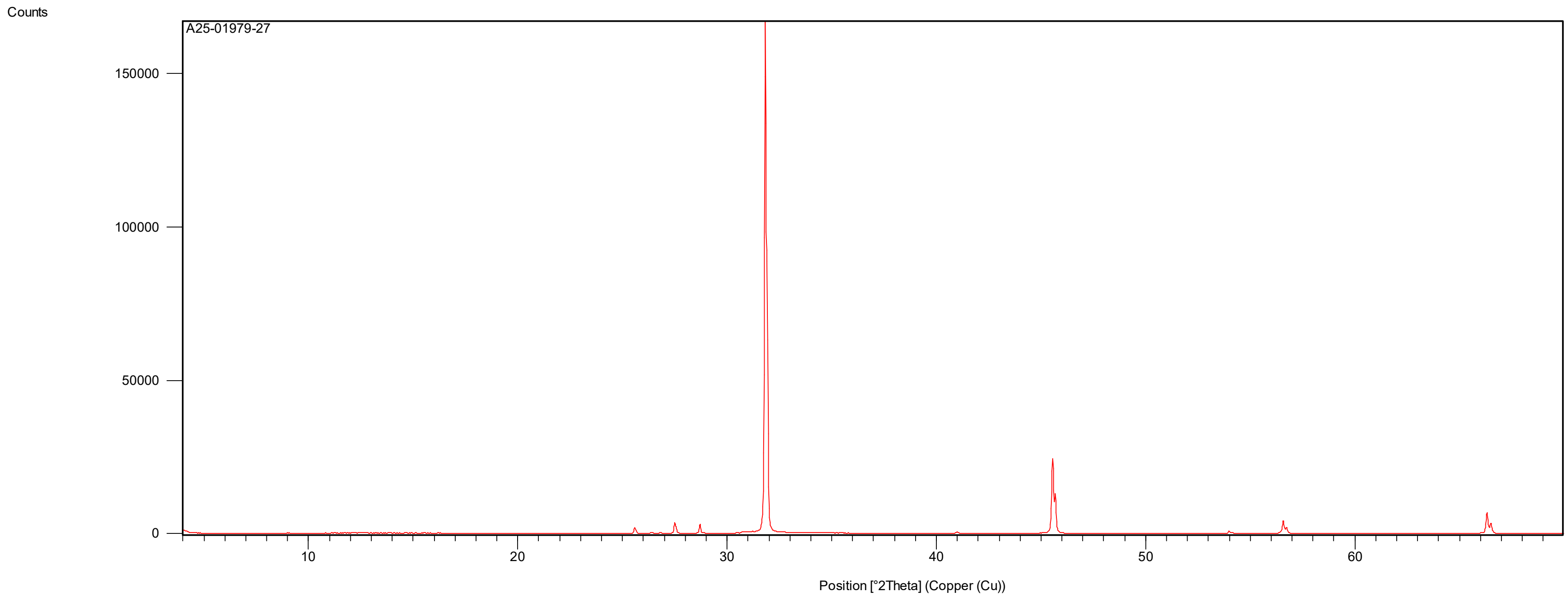

Counts
A25-01979-27
150000
100000
50000
0
10
20
30
40
50
60
Position [°2Theta] (Copper (Cu))

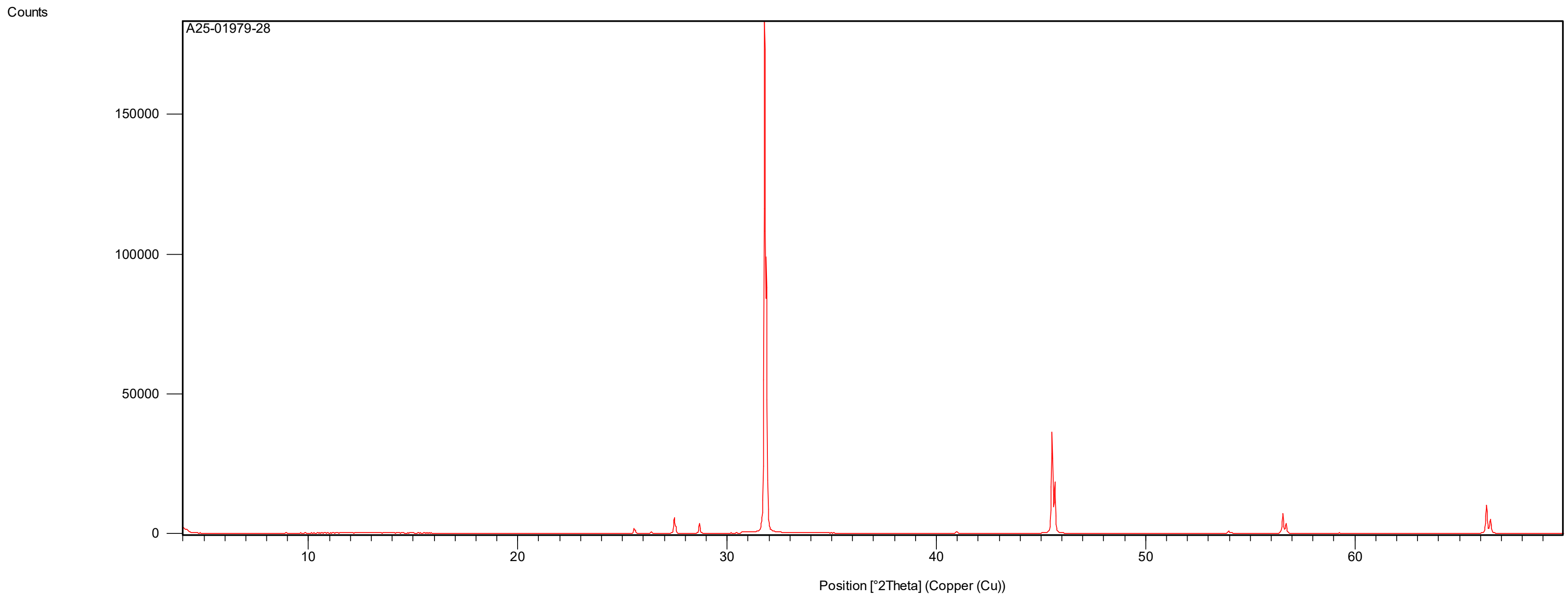
Counts
A25-01979-28
150000
100000
50000
0
10
20
30
40
50
60
Position [°2Theta] (Copper (Cu))

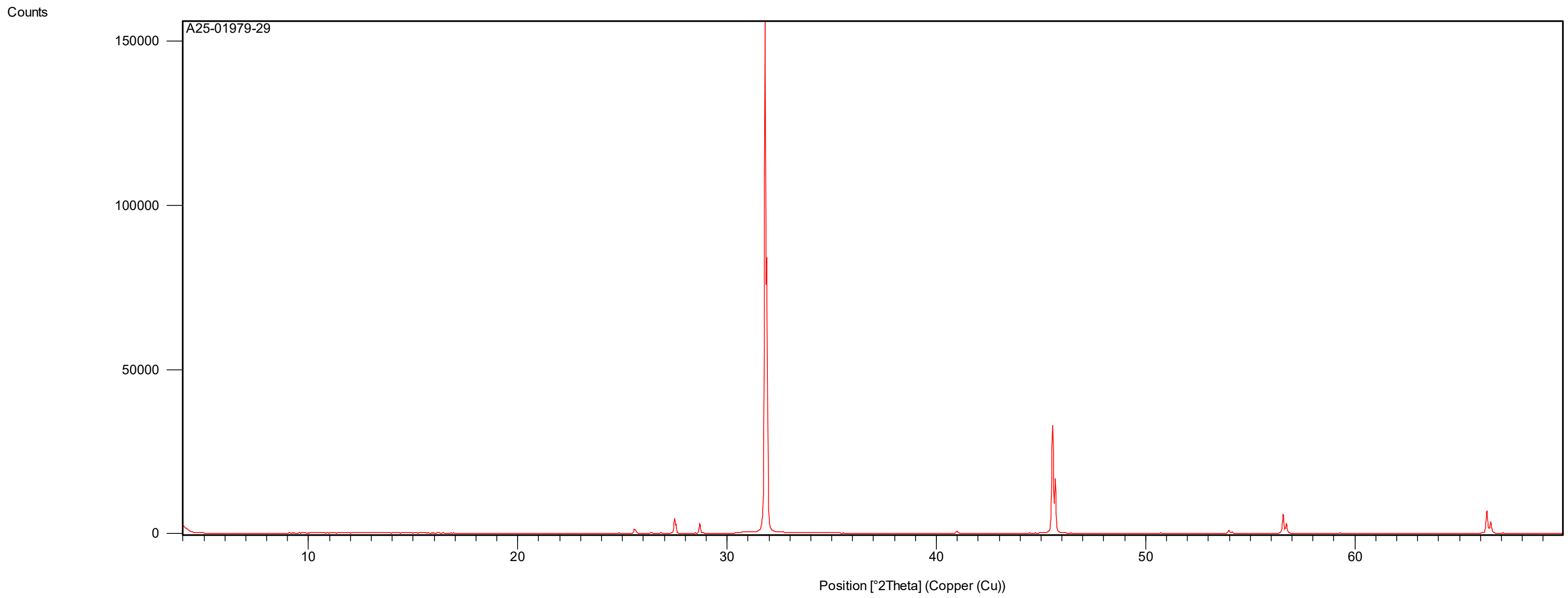
Counts
A25-01979-29
150000
100000
50000
0
10
20
30
40
50
60
Position [°2Theta] (Copper (Cu))

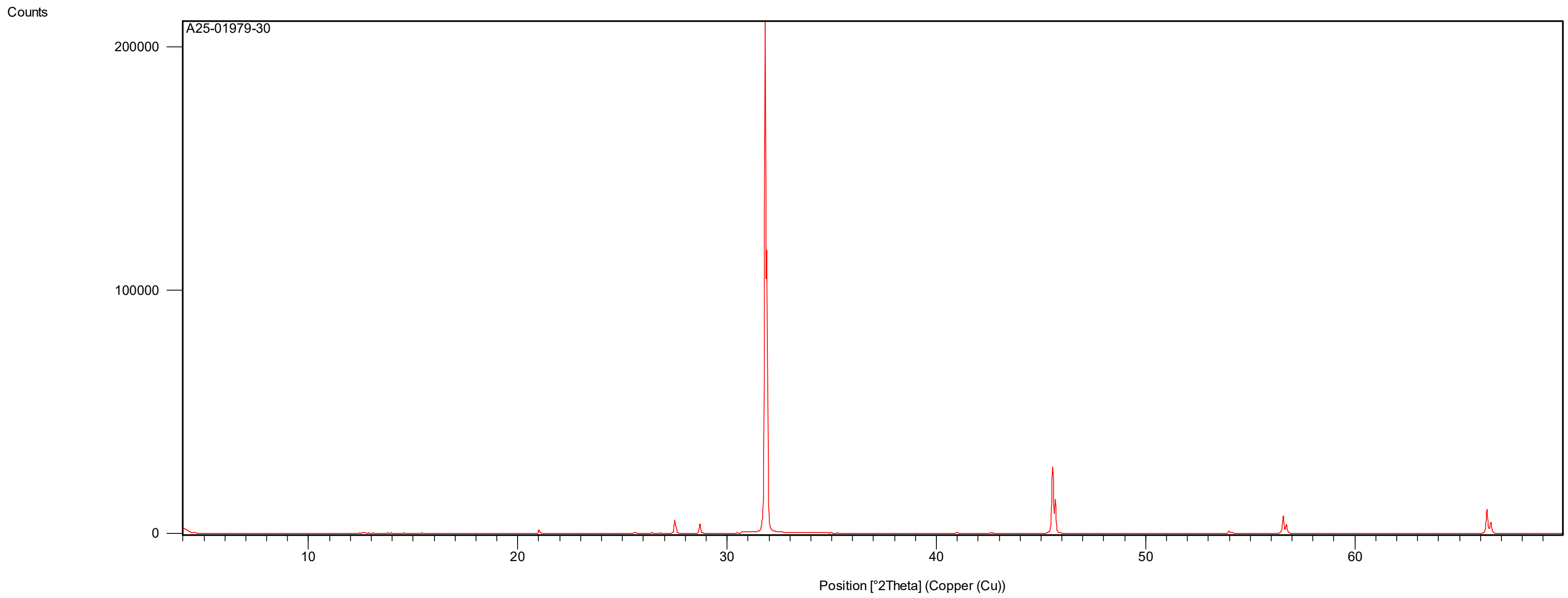
Counts
A25-01979-30
200000
100000
0
10
20
30
40
50
60
Position [°2Theta] (Copper (Cu))

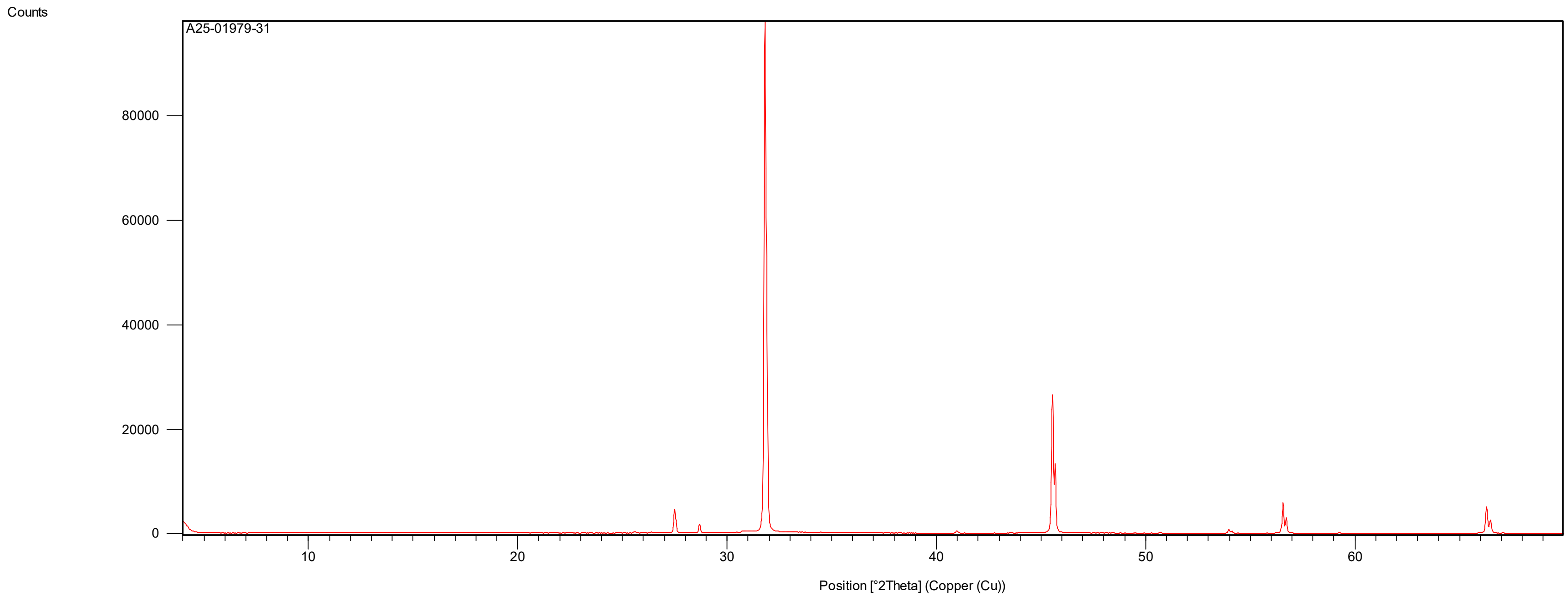
Counts
A25-01979-31
80000
60000
40000
20000
0
10
20
30
40
50
60
Position [°2Theta] (Copper (Cu))

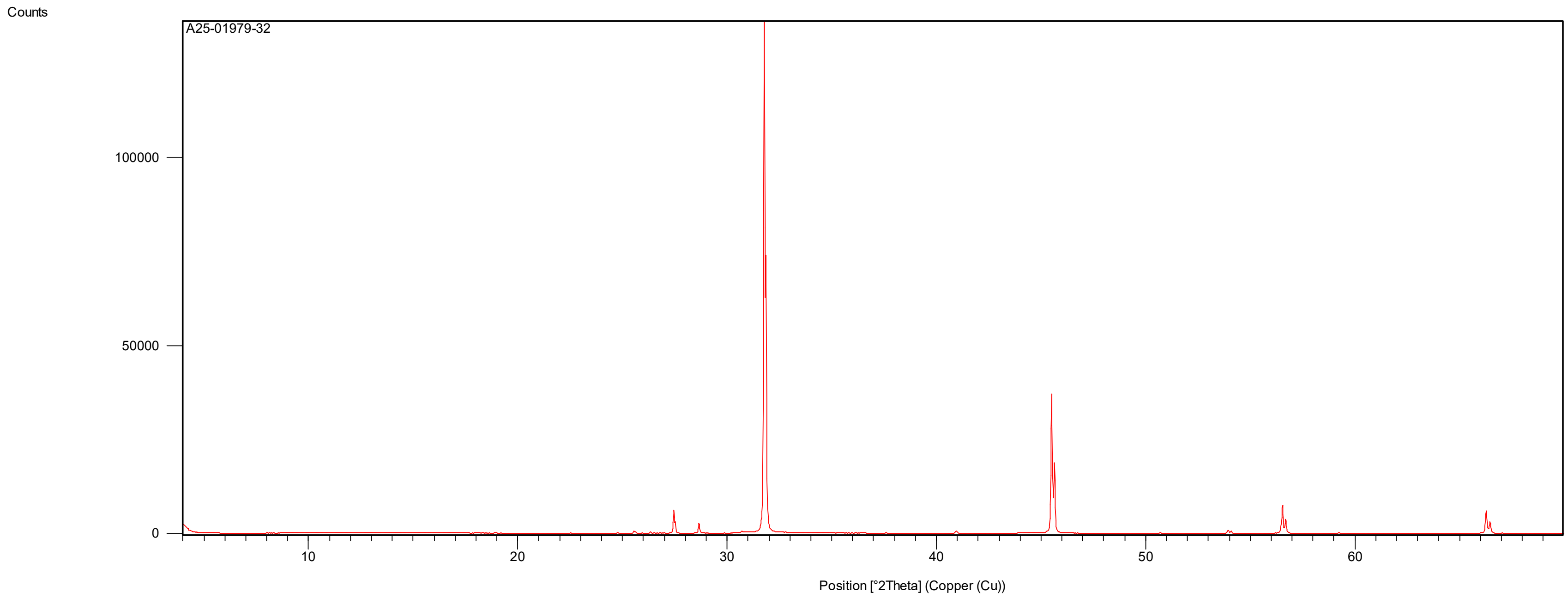
Counts
A25-01979-32
100000
50000
0
10
20
30
40
50
60
Position [°2Theta] (Copper (Cu))

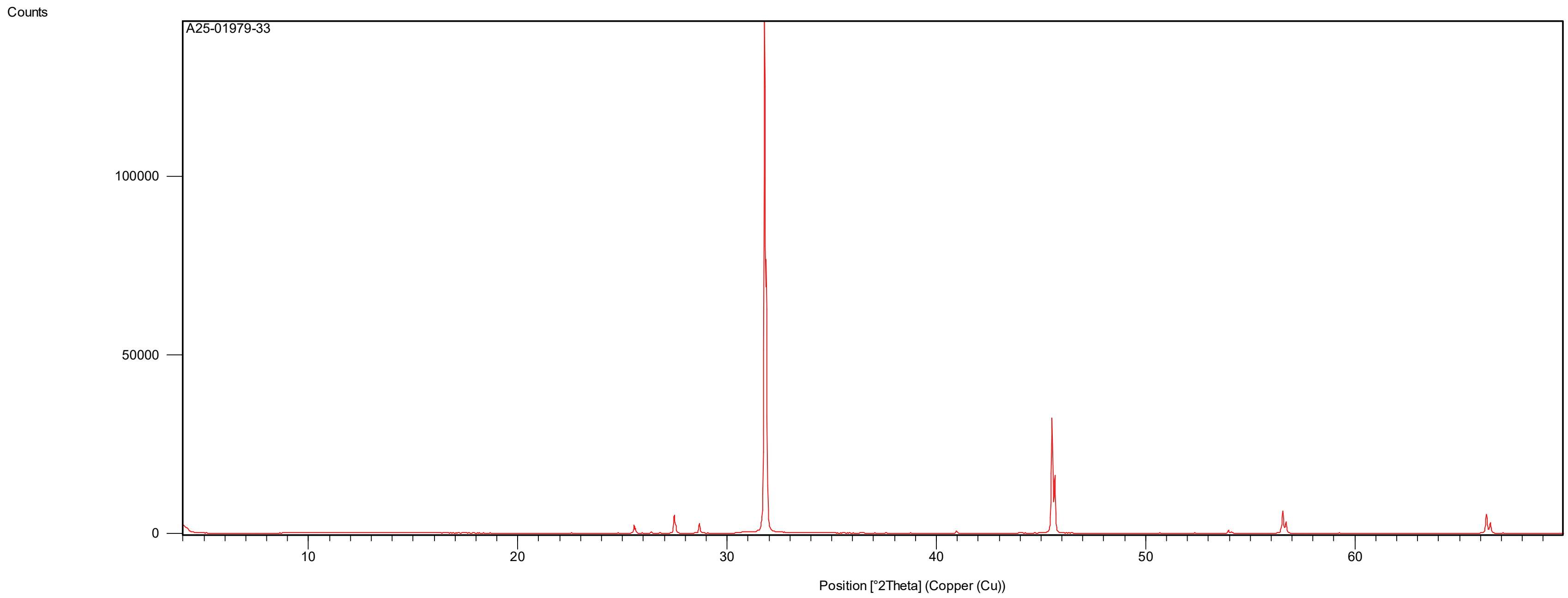
Counts
A25-01979-33
100000
50000
0
10
20
30
40
50
60
Position [°2Theta] (Copper (Cu))

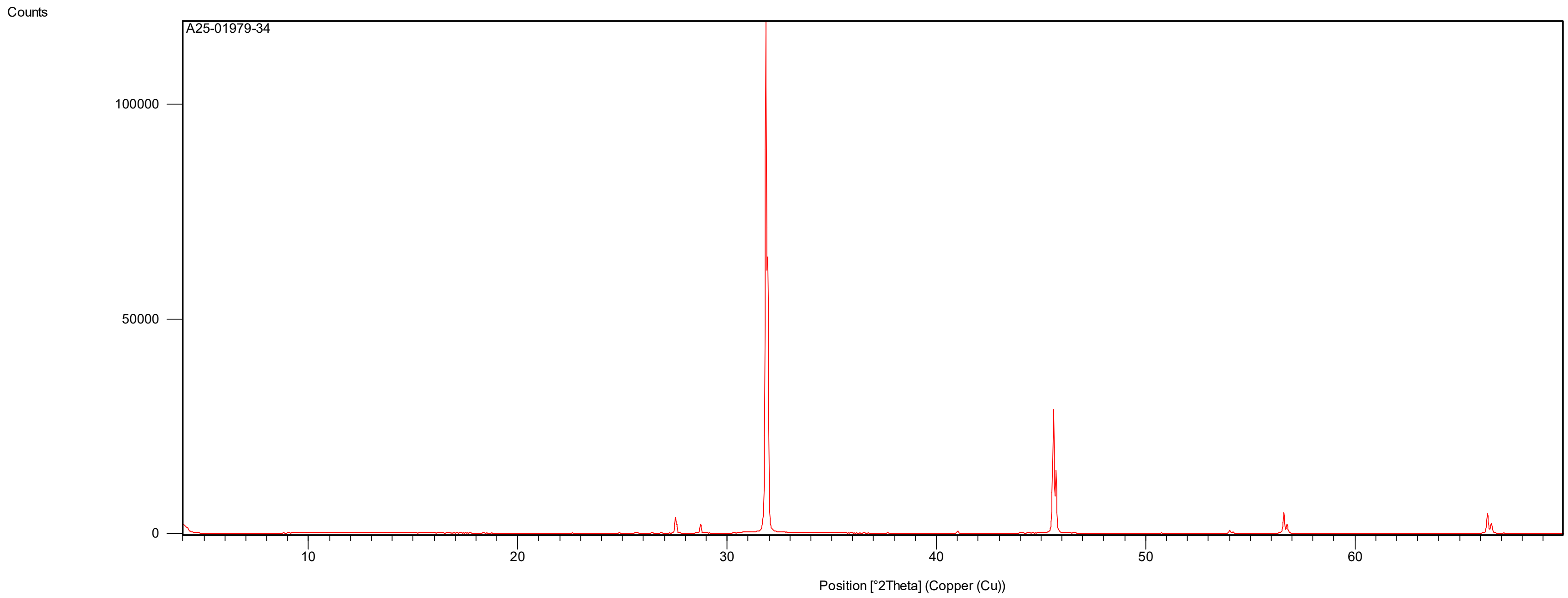
Counts
A25-01979-34
100000
50000
0
10
20
30
40
50
60
Position [°2Theta] (Copper (Cu))

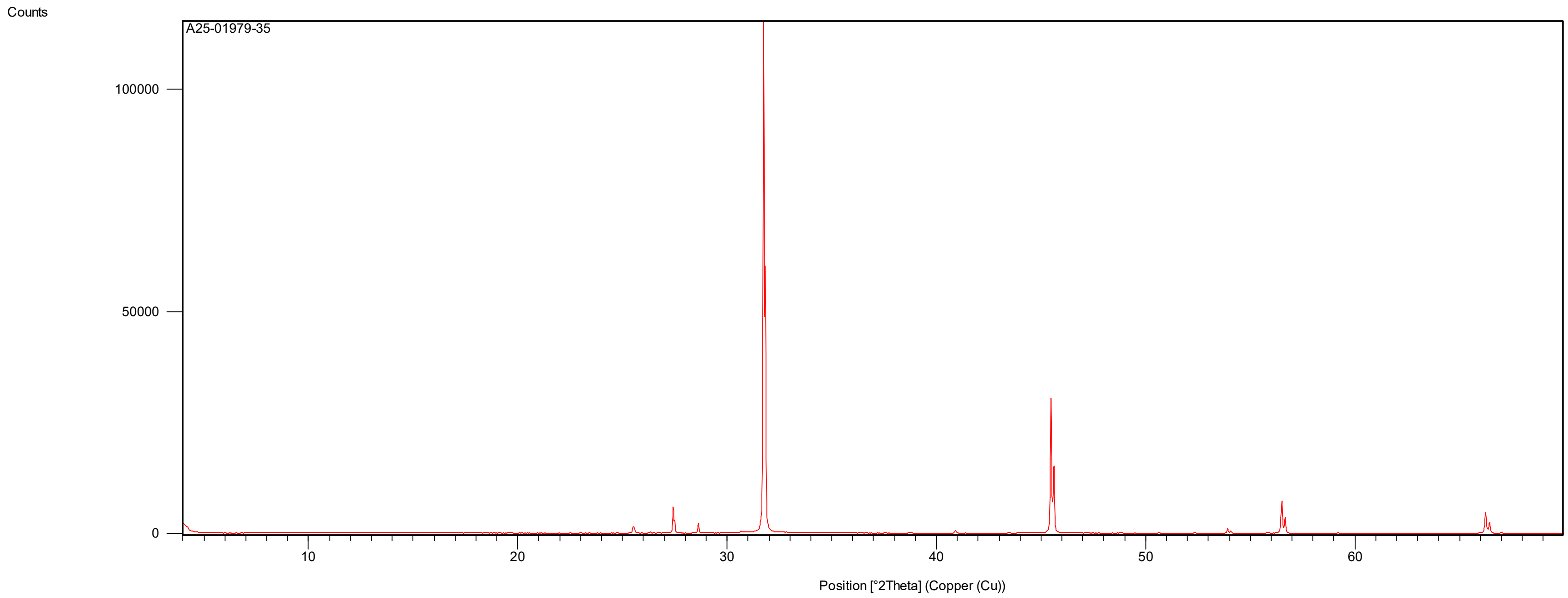
Counts
A25-01979-35
100000
50000
0
10
20
30
40
50
60
Position [°2Theta] (Copper (Cu))

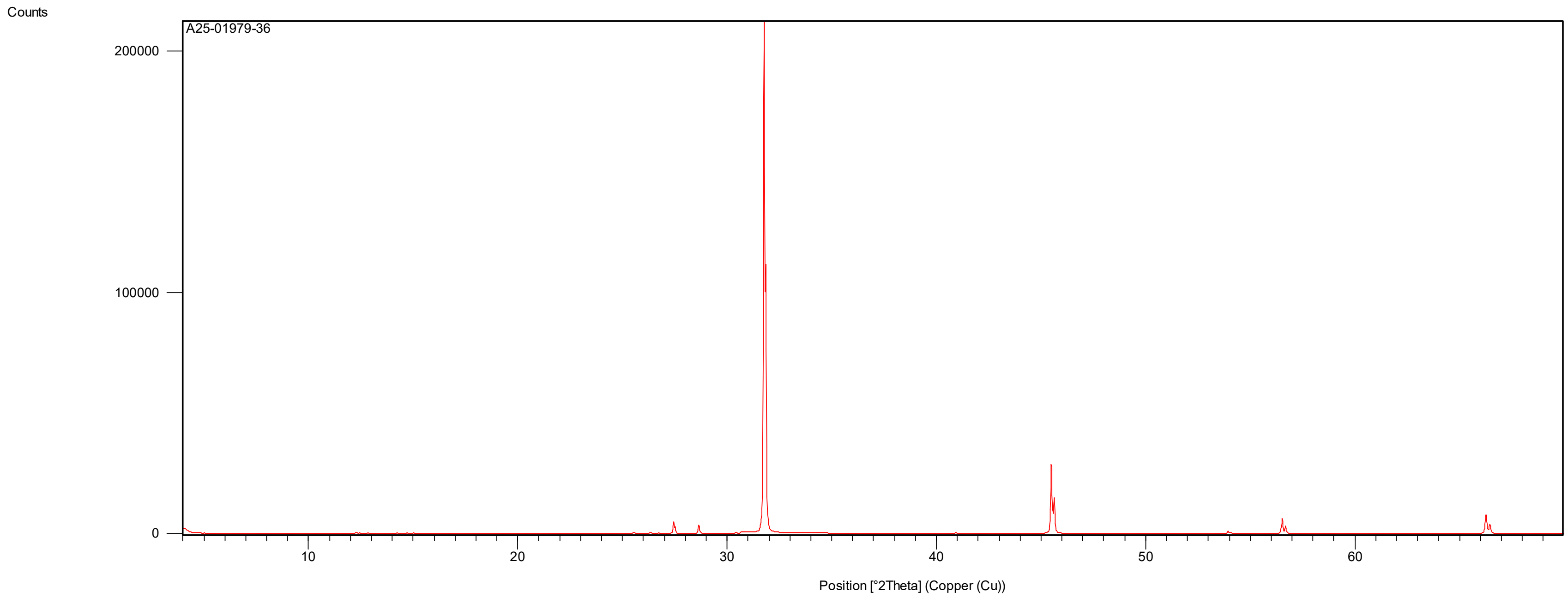
Counts
A25-01979-36
200000
100000
0
10
20
30
40
50
60
Position [°2Theta] (Copper (Cu))

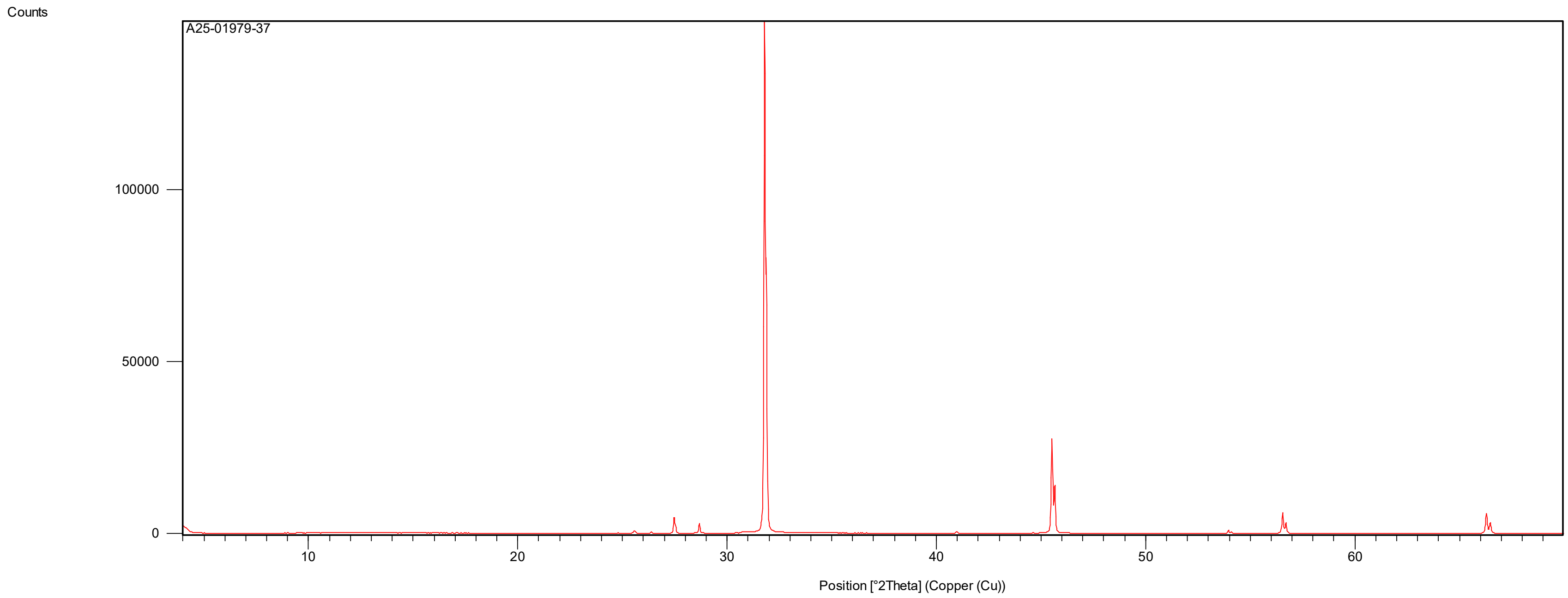
Counts
A25-01979-37
100000
50000
0
10
20
30
40
50
60
Position [°2Theta] (Copper (Cu))

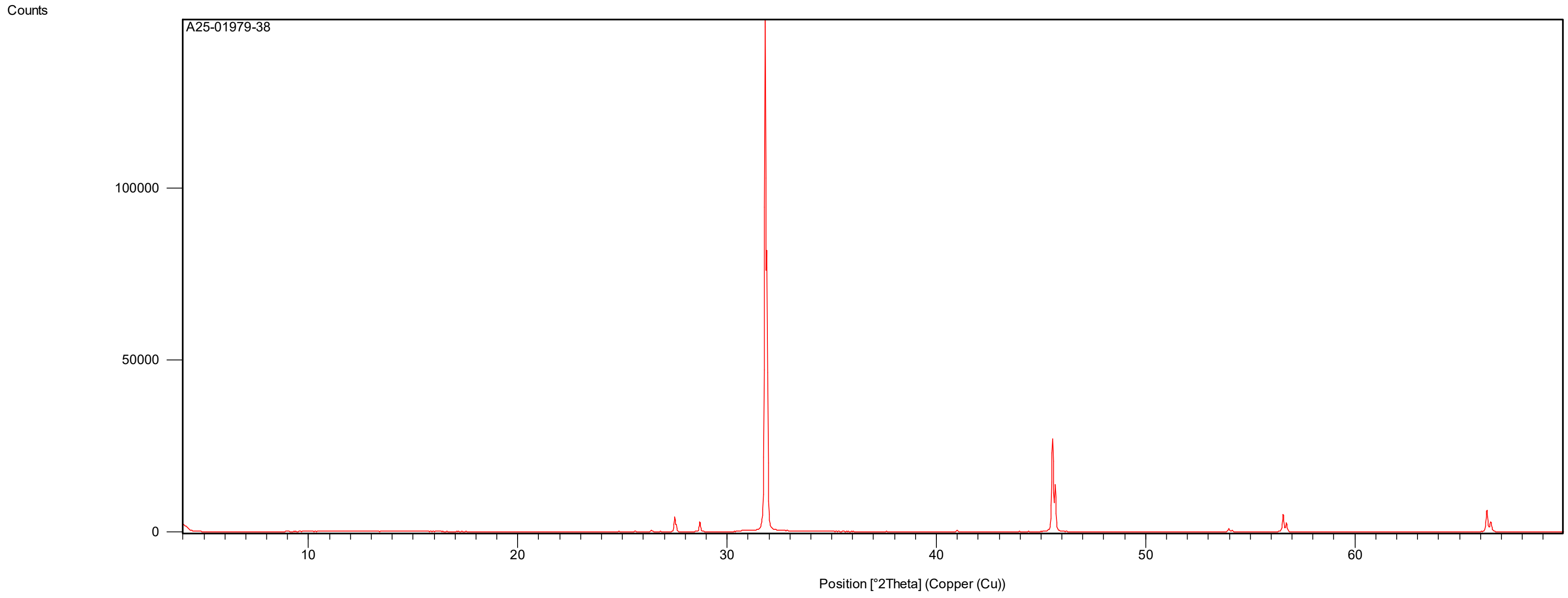
Counts
A25-01979-38
100000
50000
0
10
20
30
40
50
60
Position [°2Theta] (Copper (Cu))

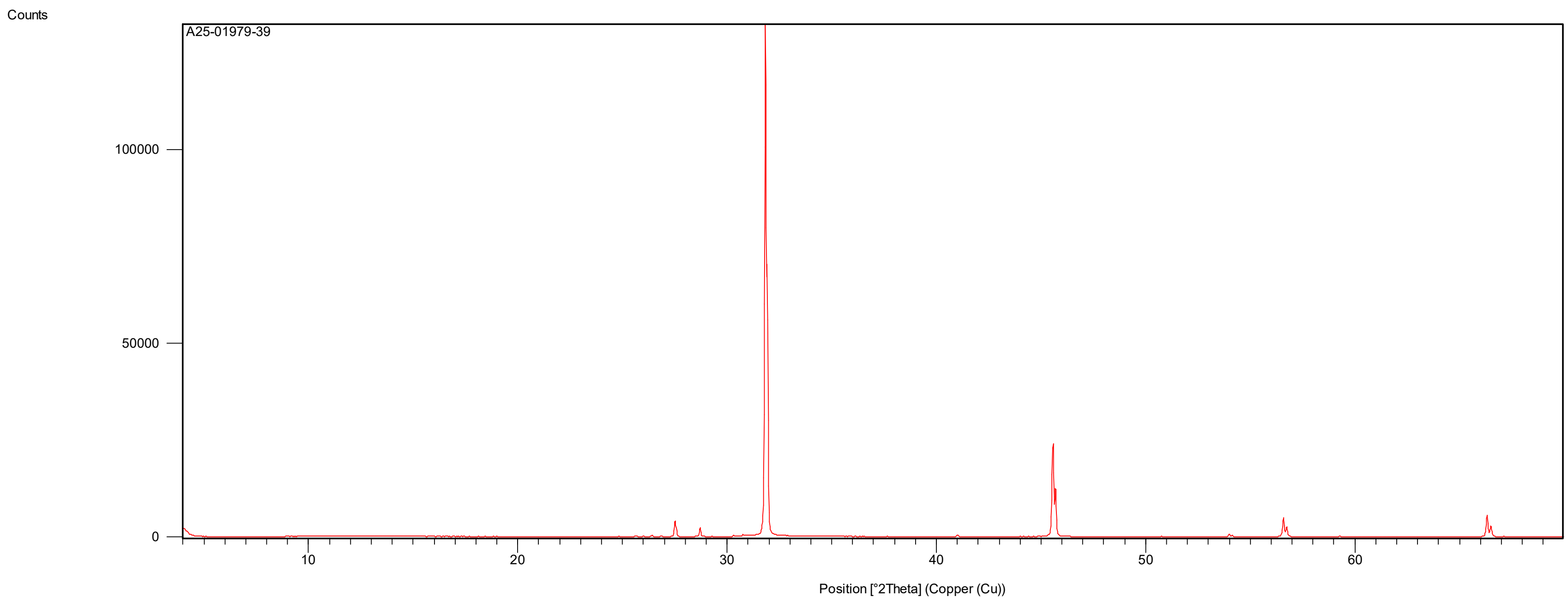
Counts
A25-01979-39
100000
50000
0
10
20
30
40
50
60
Position [°2Theta] (Copper (Cu))

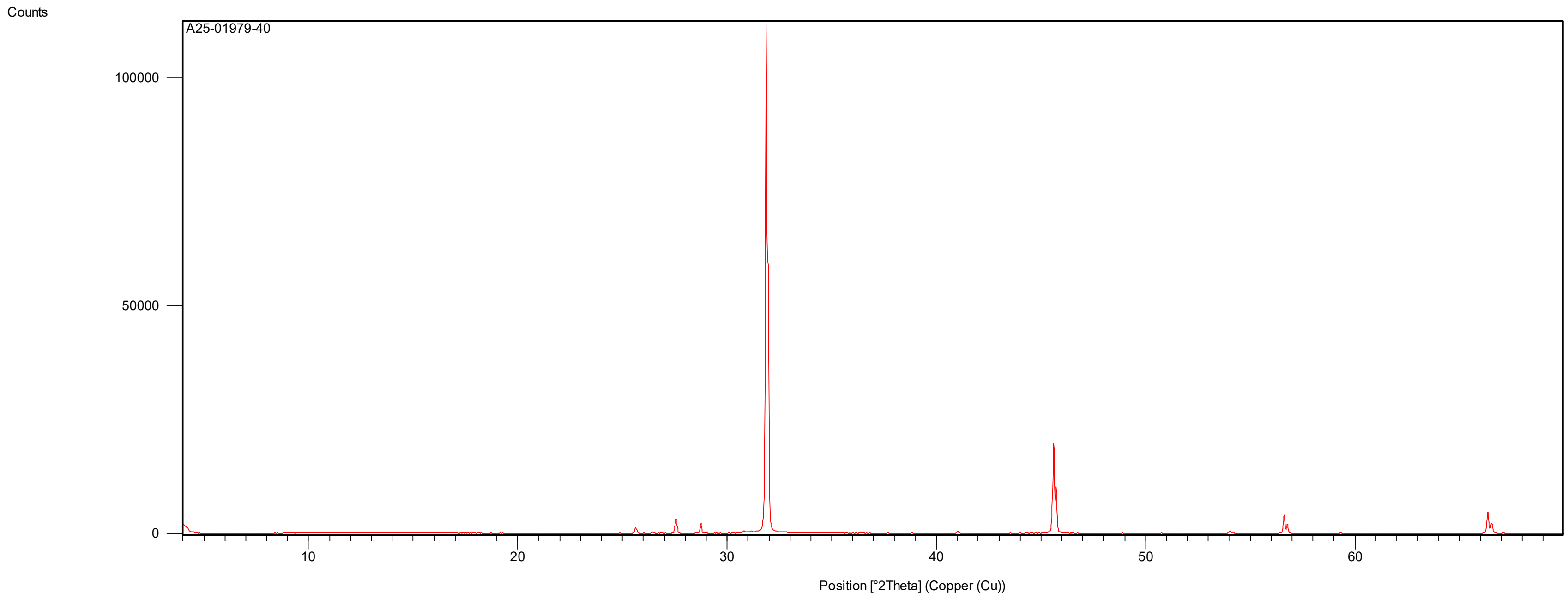
Counts
A25-01979-40
100000
50000
0
10
20
30
40
50
60
Position [°2Theta] (Copper (Cu))

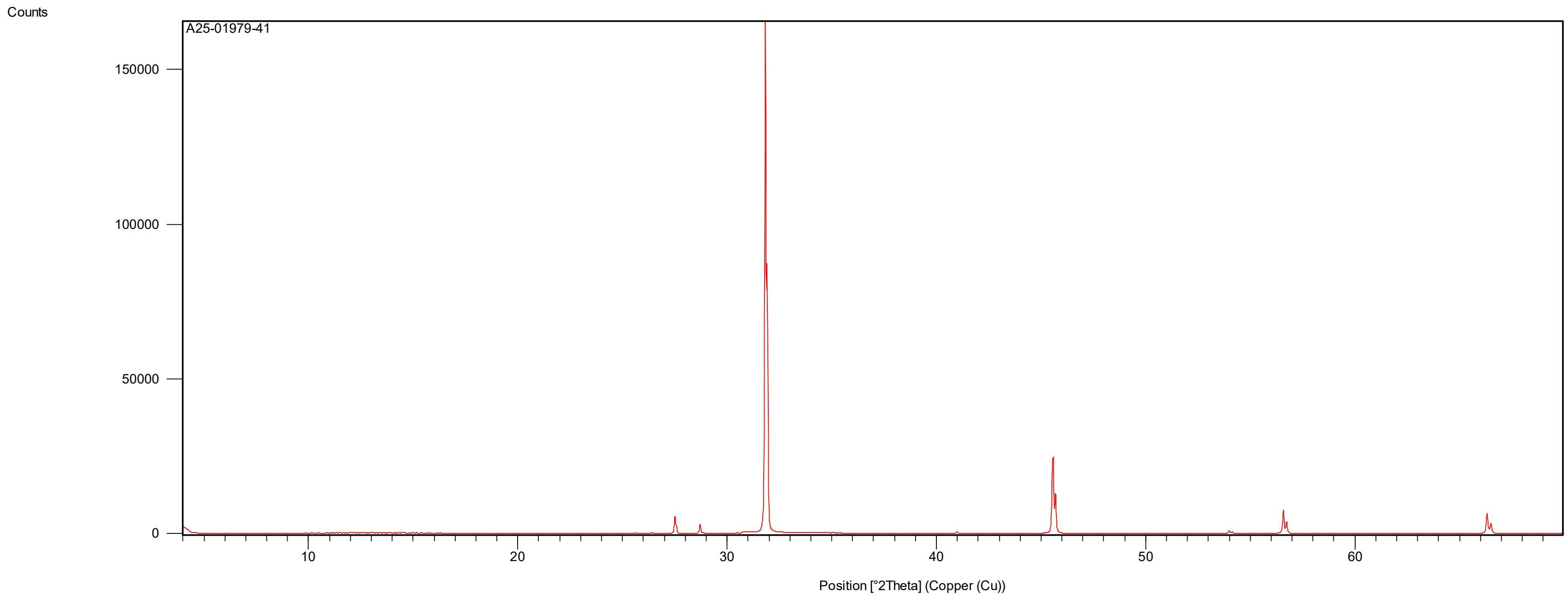
Counts
A25-01979-41
150000
100000
50000
0
10
20
30
40
50
60
Position [°2Theta] (Copper (Cu))

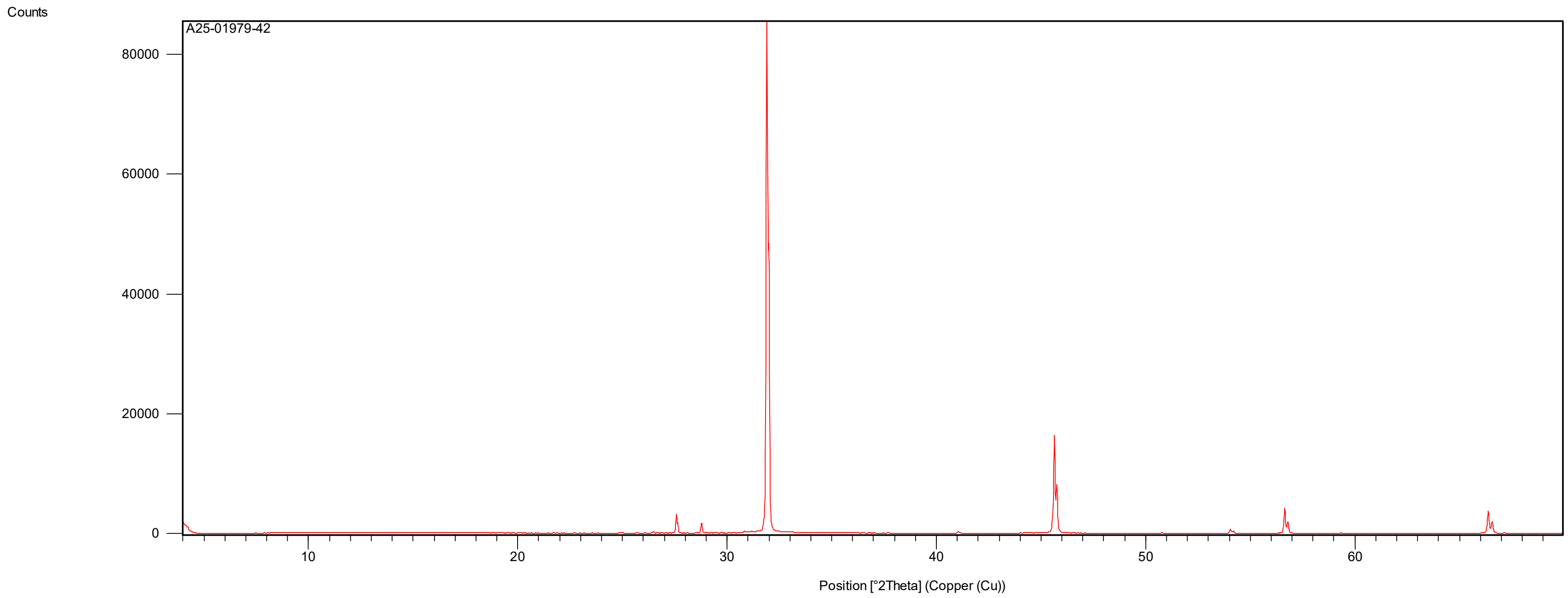
Counts
A25-01979-42
80000
60000
40000
20000
0
10
20
30
40
50
60
Position [°2Theta] (Copper (Cu))

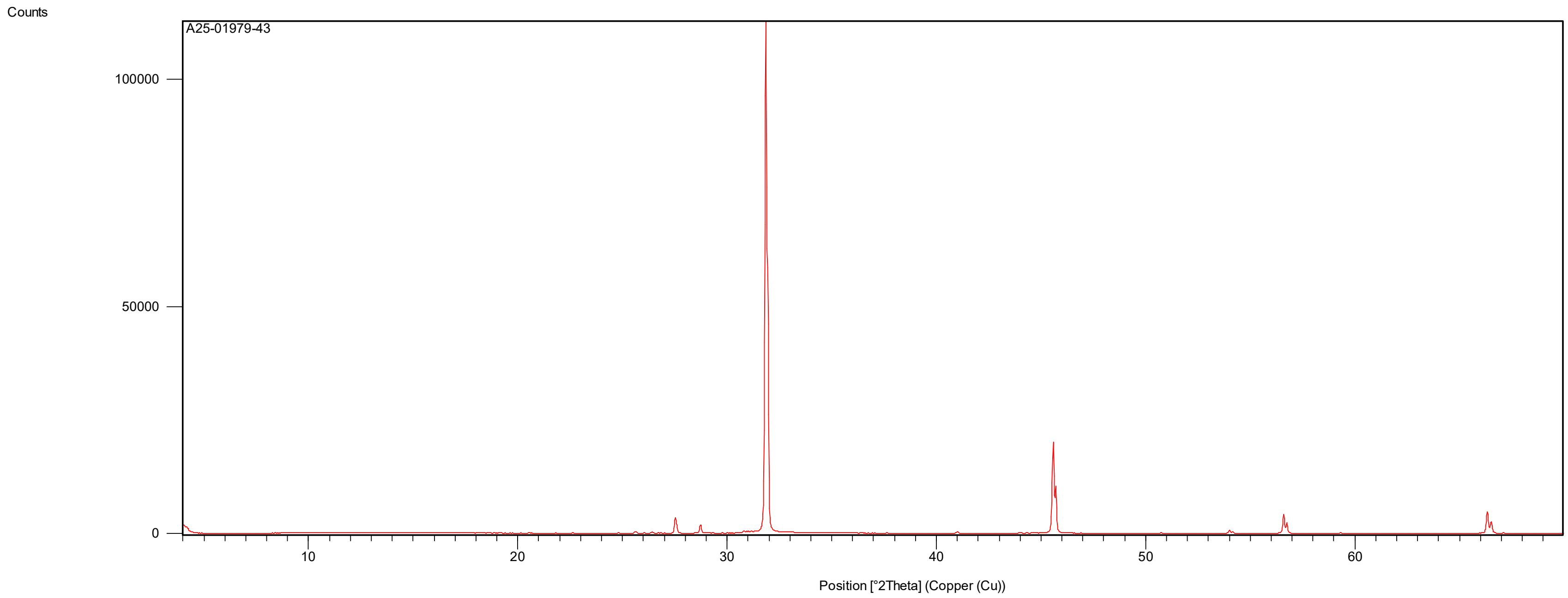
Counts
A25-01979-43
100000
50000
0
10
20
30
40
50
60
Position [°2Theta] (Copper (Cu))

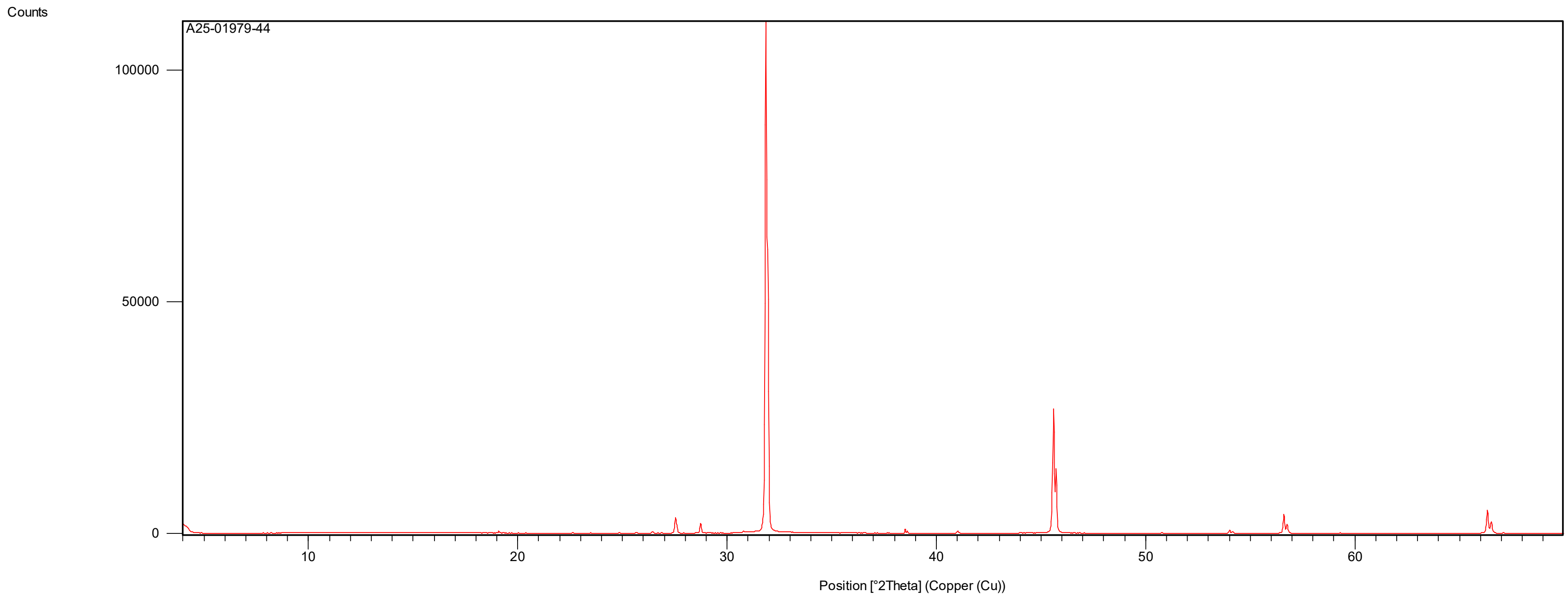
Counts
A25-01979-44
100000
50000
0
10
20
30
40
50
60
Position [°2Theta] (Copper (Cu))

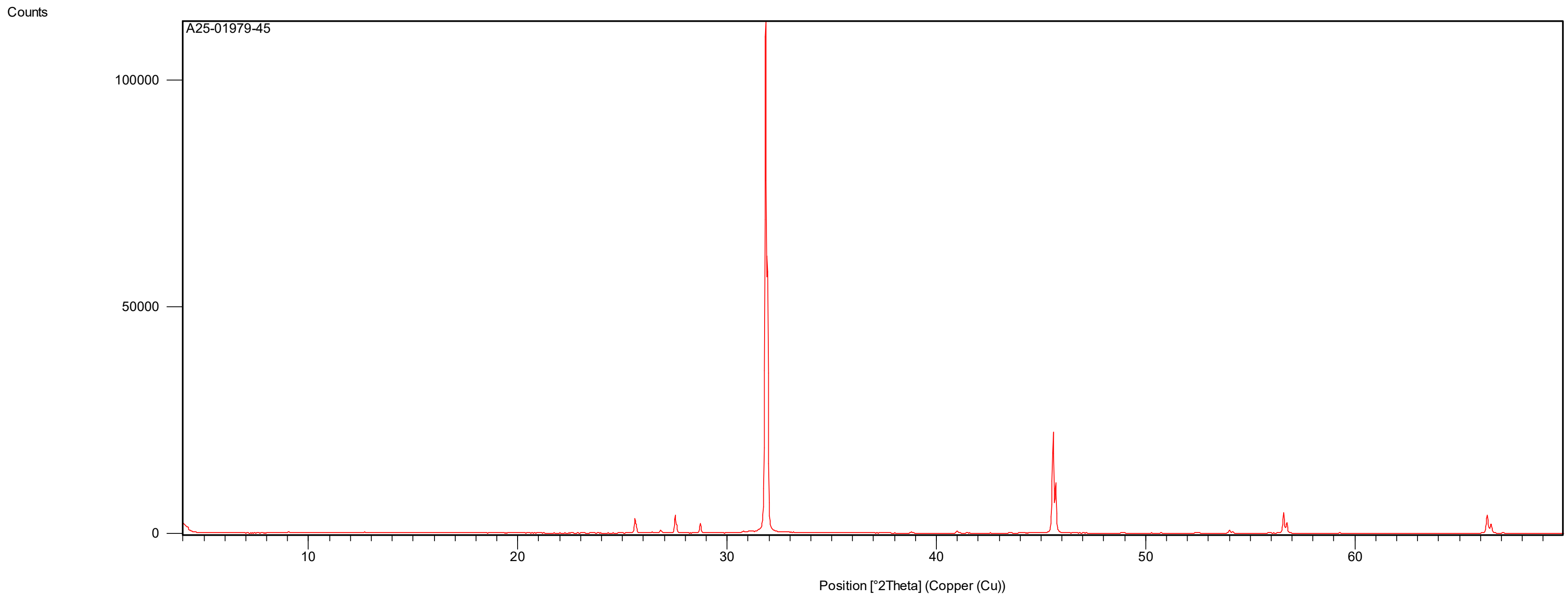
Counts
A25-01979-45
100000
50000
0
10
20
30
40
50
60
Position [°2Theta] (Copper (Cu))
