# Supplementary material for: Geochemistry and X-ray diffraction data from rock salts and saltwork wastes of Canada: data compilation
Source: Data Brief. 2026 Jun 6;67:112941. doi: 10.1016/j.dib.2026.112941 (PMC13292661; doi:10.1016/j.dib.2026.112941)
Supplement: Supplementary file 11 [file mmc11.pdf]

Quality Analysis ... *Innovative Technologies*

**Report No.:**	**A25-04983**
**Report Date:**	**02-Jun-25**
**Date Submitted:**	**01-May-25**
**Your Reference:**	**CMGD**

Geological Survey of Canada-AB
3303 33rd St NW
Calgary Alberta T2-2A7
Canada

ATTN: Pavel Kabanov

CERTIFICATE OF ANALYSIS

37 Pulp samples were submitted for analysis.

The following analytical package(s) were requested:		Testing Date:
UT-6	QOP Total/QOP Ultratrace- 4acid Digest (Total Digestion ICPOES/ICPMS)	2025-05-26 15:13:01

REPORT **A25-04983**

This report may be reproduced without our consent. If only selected portions of the report are reproduced, permission must be obtained. If no instructions were given at time of sample submittal regarding excess material, it will be discarded within 90 days of this report. Our liability is limited solely to the analytical cost of these analyses. Test results are representative only of material submitted for analysis.

Notes:

Values which exceed the upper limit should be assayed for accurate numbers.

Refer to the Scope of Accreditation for information on accredited elements.

SCC Accredited LAB / LAB Accrédité CCN TM

CERTIFIED BY:

Mark Vandergeest
Quality Control Coordinator

ACTIVATION LABORATORIES LTD.
41 Bittern Street, Ancaster, Ontario, Canada, L9G 4V5
TELEPHONE +905 648-9611 or +1.888.228.5227 FAX +1.905.648.9613
E-MAIL Ancaster@actlabs.com ACTLABS GROUP WEBSITE www.actlabs.com

Analyte Symbol	Li	Na	Mg	Al	K	Ca	Cd	V	Cr	Mn	Fe	Hf	Ni	Er	Be	Ho	Ag	Cs	Co	Eu	Bi	Se	Zn
Unit Symbol	ppm	%	%	%	%	%	ppm	ppm	ppm	ppm	%	ppm	ppm	ppm	ppm	ppm	ppm	ppm	ppm	ppm	ppm	ppm	ppm
Lower Limit	1	0.01	0.01	0.01	0.01	0.01	0.3	1	1	1	0.01	0.1	1	0.1	0.1	0.1	0.3	0.05	1	0.05	0.02	0.1	1
Method Code	TD-ICP	TD-MS	TD-MS	TD-ICP	TD-MS	TD-MS	TD-ICP	TD-MS	TD-MS	TD-MS	TD-MS	TD-MS	TD-ICP	TD-MS	TD-MS	TD-MS	TD-ICP	TD-MS	TD-ICP	TD-MS	TD-MS	TD-MS	TD-ICP
Victoria Plains 1496.95m	42	0.09	5.41	1.35	1.09	15.7	< 0.3	22	18	137	0.70	0.4	16	0.3	< 0.1	< 0.1	< 0.3	1.06	4	0.12	< 0.02	< 0.1	14
Victoria Plains 1497.8m	164	0.20	6.02	4.75	3.95	8.19	< 0.3	77	60	298	2.72	2.2	59	1.3	1.1	0.4	< 0.3	3.79	12	0.53	0.09	< 0.1	39
Victoria Plains 1498.3m	100	0.13	8.52	2.87	2.58	16.2	< 0.3	51	42	293	1.69	1.1	30	0.9	0.7	0.3	< 0.3	2.46	6	0.41	0.03	< 0.1	25
Victoria Plains 1498.8m	91	0.14	7.68	2.76	2.31	15.5	< 0.3	48	32	236	1.57	1.3	24	0.9	0.6	0.3	< 0.3	2.19	6	0.42	0.02	< 0.1	22
Victoria Plains 1499.4m	271	0.30	5.65	5.19	3.12	8.19	< 0.3	86	35	255	2.93	1.9	52	1.2	1.5	0.4	< 0.3	4.70	11	0.52	0.06	< 0.1	44
Victoria Plains 1500m	107	0.11	4.98	3.03	2.17	15.5	< 0.3	46	40	172	1.59	1.3	32	0.7	0.7	0.2	< 0.3	2.38	7	0.34	0.11	< 0.1	24
Victoria Plains 1502.87m	73	> 3.00	3.27	1.19	1.11	13.7	0.3	16	29	82	0.74	0.4	13	0.3	< 0.1	< 0.1	< 0.3	0.90	3	0.12	< 0.02	< 0.1	19
Victoria Plains 1505.28m	< 1	0.73	1.23	< 0.01	0.01	11.4	< 0.3	3	3	18	0.01	< 0.1	< 1	< 0.1	< 0.1	< 0.1	< 0.3	< 0.05	< 1	< 0.05	< 0.02	< 0.1	1
Victoria Plains 1505.87m	4	> 3.00	8.18	0.01	0.08	18.9	< 0.3	2	4	75	0.06	< 0.1	< 1	< 0.1	< 0.1	< 0.1	< 0.3	< 0.05	< 1	< 0.05	< 0.02	< 0.1	29
Victoria Plains 1509.5m	< 1	> 3.00	1.47	0.02	0.05	27.9	< 0.3	1	4	38	0.02	< 0.1	< 1	< 0.1	< 0.1	< 0.1	< 0.3	< 0.05	< 1	< 0.05	< 0.02	0.3	< 1
Victoria Plains 1514.8m	< 1	> 3.00	7.37	0.12	0.20	22.7	< 0.3	3	6	94	0.10	< 0.1	1	< 0.1	< 0.1	< 0.1	< 0.3	0.06	< 1	< 0.05	< 0.02	< 0.1	2
Victoria Plains 1515.4m	< 1	> 3.00	3.93	0.04	0.11	27.7	< 0.3	2	15	67	0.06	< 0.1	< 1	< 0.1	< 0.1	< 0.1	< 0.3	< 0.05	< 1	< 0.05	< 0.02	0.2	< 1
Victoria Plains 1517.2m	2	> 3.00	5.95	0.27	0.34	27.9	< 0.3	5	4	94	0.15	< 0.1	3	< 0.1	< 0.1	< 0.1	< 0.3	0.25	< 1	< 0.05	< 0.02	< 0.1	5
Victoria Plains 1521.75m	< 1	> 3.00	0.68	0.12	0.20	31.2	< 0.3	2	6	51	0.08	< 0.1	2	< 0.1	< 0.1	< 0.1	< 0.3	0.07	< 1	< 0.05	< 0.02	0.3	1
Victoria Plains 1523.7m	< 1	> 3.00	2.33	0.08	0.15	32.9	< 0.3	2	3	64	0.06	< 0.1	1	< 0.1	< 0.1	< 0.1	< 0.3	0.06	< 1	< 0.05	< 0.02	0.4	5
Victoria Plains 1524.75m	1	1.81	3.94	0.20	0.27	32.1	< 0.3	3	8	100	0.12	< 0.1	2	< 0.1	< 0.1	< 0.1	< 0.3	0.13	< 1	< 0.05	< 0.02	0.3	2
Victoria Plains 1526.95m	< 1	> 3.00	2.93	0.14	0.22	34.0	< 0.3	3	4	83	0.10	< 0.1	2	< 0.1	< 0.1	< 0.1	< 0.3	0.08	< 1	< 0.05	< 0.02	0.5	2
Victoria Plains 1548.45m	210	0.58	11.0	3.21	1.98	14.0	< 0.3	44	41	303	1.53	1.2	27	1.2	0.6	0.4	< 0.3	3.01	6	0.51	0.11	< 0.1	29
Victoria Plains 1661.8m	395	1.98	12.1	0.08	0.36	19.9	< 0.3	2	9	37	0.03	< 0.1	< 1	< 0.1	< 0.1	< 0.1	< 0.3	0.27	< 1	< 0.05	< 0.02	0.1	< 1
Victoria Plains 1663.25m	32	2.66	11.7	0.04	0.07	21.7	< 0.3	< 1	6	53	0.04	< 0.1	< 1	< 0.1	< 0.1	< 0.1	< 0.3	< 0.05	< 1	< 0.05	< 0.02	< 0.1	< 1
FT SASK 7-23 1650.35m	362	0.71	9.28	6.58	3.63	4.76	< 0.3	81	33	217	4.58	1.5	37	1.3	2.0	0.5	< 0.3	4.66	8	0.83	0.07	< 0.1	41
FT SASK 7-23 1670.55m	113	> 3.00	5.87	4.43	3.78	11.2	< 0.3	47	26	249	2.23	1.5	20	1.0	1.1	0.3	< 0.3	3.13	6	0.53	0.03	< 0.1	34
FT SASK 7-23 1671.05m	105	0.77	3.75	4.40	3.83	12.4	< 0.3	45	42	155	2.02	1.5	24	0.7	1.2	0.2	< 0.3	2.91	10	0.34	0.06	< 0.1	28
FT SASK 7-23 1677.1m	106	> 3.00	2.81	6.24	4.97	7.60	< 0.3	61	47	237	2.58	1.7	38	0.9	1.5	0.3	< 0.3	4.32	16	0.56	0.16	< 0.1	39
FT SASK 7-23 1677.7m	112	2.05	2.45	7.62	4.71	8.15	< 0.3	58	50	214	2.58	1.7	26	0.9	1.5	0.3	< 0.3	4.43	8	0.49	0.05	< 0.1	40
FT SASK 7-23 1680.7m	81	> 3.00	1.76	3.89	2.05	7.55	< 0.3	38	30	161	1.54	1.0	16	0.5	0.9	0.2	< 0.3	2.58	5	0.34	0.04	< 0.1	26
FT SASK 7-23 1683.6m	74	0.82	4.84	4.08	2.19	13.7	< 0.3	33	21	370	2.05	1.0	17	0.9	0.8	0.3	< 0.3	2.62	5	0.51	0.09	0.2	24
FT SASK 7-23 1684.8m	88	1.48	2.97	4.60	2.58	15.6	< 0.3	38	31	297	2.09	1.1	23	1.0	1.0	0.4	< 0.3	3.14	8	0.64	0.15	0.5	28
FT SASK 7-23 1685.4m	146	0.29	2.71	6.57	4.59	7.90	< 0.3	69	56	276	3.04	1.8	43	1.0	1.5	0.4	< 0.3	4.74	16	0.55	0.21	0.6	43

Analyte Symbol	Li	Na	Mg	Al	K	Ca	Cd	V	Cr	Mn	Fe	Hf	Ni	Er	Be	Ho	Ag	Cs	Co	Eu	Bi	Se	Zn
Unit Symbol	ppm	%	%	%	%	%	ppm	ppm	ppm	ppm	%	ppm	ppm	ppm	ppm	ppm	ppm	ppm	ppm	ppm	ppm	ppm	ppm
Lower Limit	1	0.01	0.01	0.01	0.01	0.01	0.3	1	1	1	0.01	0.1	1	0.1	0.1	0.1	0.3	0.05	1	0.05	0.02	0.1	1
Method Code	TD-ICP	TD-MS	TD-MS	TD-ICP	TD-MS	TD-MS	TD-ICP	TD-MS	TD-MS	TD-MS	TD-MS	TD-MS	TD-ICP	TD-MS	TD-MS	TD-MS	TD-ICP	TD-MS	TD-ICP	TD-MS	TD-MS	TD-MS	TD-ICP
FT SASK 7-23 1692.3m	94	> 3.00	1.51	5.49	3.54	6.51	< 0.3	45	38	174	2.16	1.3	23	0.7	1.2	0.2	< 0.3	3.53	8	0.45	0.11	0.5	30
FT SASK 7-23 1692.9m	82	0.71	2.14	4.71	3.09	16.8	< 0.3	43	28	296	2.31	1.3	18	1.0	1.1	0.4	< 0.3	3.40	6	0.65	0.04	0.6	27
FT SASK 7-23 1698.95m	86	> 3.00	2.98	3.79	2.04	16.1	< 0.3	36	33	262	1.51	1.1	18	0.9	1.0	0.3	< 0.3	2.47	7	0.53	0.10	0.3	21
FT SASK 7-23 1699.85m	176	2.32	5.21	5.12	3.13	8.27	< 0.3	58	43	303	2.20	1.3	24	0.8	1.5	0.3	< 0.3	3.49	7	0.41	0.06	0.2	31
FT SASK 7-23 1701.05m	175	> 3.00	4.55	5.04	2.39	7.47	< 0.3	53	55	277	2.25	1.5	23	0.9	1.2	0.3	< 0.3	3.53	7	0.53	0.08	0.5	31
FT SASK 7-23 1705.5m	136	1.98	2.13	5.25	2.45	11.8	< 0.3	28	47	198	2.07	1.1	21	0.9	1.2	0.3	< 0.3	3.71	7	0.55	0.08	0.6	31
PMC 140 FTSASK 1714.7m	73	> 3.00	2.54	3.51	1.93	19.7	< 0.3	29	23	393	1.76	1.1	18	0.9	1.0	0.3	< 0.3	2.41	6	0.57	0.14	0.9	20
PMC 140 FTSASK 1716m	168	> 3.00	4.38	6.21	3.18	6.94	< 0.3	60	48	269	3.06	1.6	27	1.0	1.7	0.3	< 0.3	4.43	9	0.49	0.05	0.8	38

Analyte Symbol	Ga	As	Rb	Y	Sr	Zr	Nb	Mo	In	Sn	Sb	Te	Ba	La	Ce	Pr	Nd	Sm	Gd	Tb	Dy	Cu	Ge
Unit Symbol	ppm	ppm	ppm	ppm	ppm	ppm	ppm	ppm	ppm	ppm	ppm	ppm	ppm	ppm	ppm	ppm	ppm	ppm	ppm	ppm	ppm	ppm	ppm
Lower Limit	0.1	0.1	0.2	0.1	0.2	1	0.1	1	0.1	1	0.1	0.1	1	0.1	0.1	0.1	0.1	0.1	0.1	0.1	0.1	1	0.1
Method Code	TD-MS	TD-MS	TD-MS	TD-MS	TD-MS	TD-MS	TD-MS	TD-ICP	TD-MS	TD-MS	TD-MS	TD-MS	TD-MS	TD-MS	TD-MS	TD-MS	TD-MS	TD-MS	TD-MS	TD-MS	TD-MS	TD-ICP	TD-MS
Victoria Plains 1496.95m	3.2	1.2	29.0	2.5	766	16	2.2	< 1	< 0.1	< 1	0.1	< 0.1	59	3.9	8.0	0.9	3.0	0.8	0.6	< 0.1	0.5	11	0.1
Victoria Plains 1497.8m	12.0	9.7	93.2	9.8	383	68	8.1	1	< 0.1	1	0.7	< 0.1	213	16.7	34.1	4.2	15.5	2.9	2.2	0.3	2.0	28	< 0.1
Victoria Plains 1498.3m	7.7	3.7	60.6	7.5	339	35	4.2	< 1	< 0.1	< 1	0.2	< 0.1	125	13.4	26.4	3.2	11.4	2.3	1.9	0.2	1.4	11	< 0.1
Victoria Plains 1498.8m	6.8	2.2	56.8	7.5	362	40	4.9	< 1	< 0.1	< 1	0.3	< 0.1	125	14.4	27.4	3.3	11.6	2.1	1.8	0.2	1.5	9	< 0.1
Victoria Plains 1499.4m	15.1	6.6	113	10.1	555	63	8.4	< 1	< 0.1	2	0.5	< 0.1	130	17.6	36.3	4.4	15.7	2.7	2.2	0.3	1.9	15	< 0.1
Victoria Plains 1500m	7.6	6.2	58.8	5.9	641	41	5.1	< 1	< 0.1	< 1	0.3	< 0.1	92	10.2	20.4	2.4	8.7	1.9	1.4	0.2	1.1	21	< 0.1
Victoria Plains 1502.87m	3.0	4.1	24.5	2.2	602	15	2.0	< 1	< 0.1	< 1	0.1	< 0.1	61	3.0	6.7	0.8	2.8	0.5	0.5	< 0.1	0.4	6	< 0.1
Victoria Plains 1505.28m	< 0.1	0.3	< 0.2	< 0.1	698	< 1	< 0.1	< 1	< 0.1	< 1	< 0.1	< 0.1	3	< 0.1	< 0.1	< 0.1	< 0.1	< 0.1	< 0.1	< 0.1	< 0.1	< 1	< 0.1
Victoria Plains 1505.87m	< 0.1	0.9	0.7	< 0.1	132	< 1	< 0.1	< 1	< 0.1	< 1	< 0.1	< 0.1	3	< 0.1	< 0.1	< 0.1	< 0.1	< 0.1	< 0.1	< 0.1	< 0.1	< 1	0.2
Victoria Plains 1509.5m	< 0.1	< 0.1	0.5	< 0.1	238	1	< 0.1	< 1	< 0.1	< 1	< 0.1	< 0.1	7	0.1	0.2	< 0.1	< 0.1	< 0.1	< 0.1	< 0.1	< 0.1	< 1	0.2
Victoria Plains 1514.8m	0.4	1.4	2.6	0.3	93.8	2	0.2	< 1	< 0.1	< 1	< 0.1	< 0.1	15	0.3	0.7	0.1	0.3	0.1	< 0.1	< 0.1	< 0.1	1	0.2
Victoria Plains 1515.4m	0.3	0.2	1.2	0.2	198	1	0.1	< 1	< 0.1	< 1	< 0.1	< 0.1	5	0.3	0.6	< 0.1	0.2	< 0.1	< 0.1	< 0.1	< 0.1	1	0.2
Victoria Plains 1517.2m	0.8	< 0.1	7.0	0.7	177	4	0.3	< 1	< 0.1	< 1	< 0.1	< 0.1	26	0.6	1.2	0.1	0.6	< 0.1	0.1	< 0.1	0.1	2	0.3
Victoria Plains 1521.75m	0.3	< 0.1	3.2	0.8	375	3	0.3	< 1	< 0.1	< 1	< 0.1	< 0.1	26	0.7	1.3	0.2	0.6	< 0.1	< 0.1	< 0.1	< 0.1	2	0.2
Victoria Plains 1523.7m	0.3	< 0.1	2.4	0.7	337	2	0.2	< 1	< 0.1	< 1	< 0.1	< 0.1	16	0.6	1.0	0.1	0.5	< 0.1	< 0.1	< 0.1	< 0.1	2	0.4
Victoria Plains 1524.75m	0.6	< 0.1	4.8	1.3	302	4	0.4	< 1	< 0.1	< 1	< 0.1	< 0.1	28	1.0	1.6	0.2	0.7	0.1	0.1	< 0.1	0.2	1	0.4
Victoria Plains 1526.95m	0.5	< 0.1	3.6	1.1	305	3	0.3	< 1	< 0.1	< 1	< 0.1	< 0.1	19	1.0	1.6	0.2	0.7	0.1	0.1	< 0.1	0.1	1	0.4
Victoria Plains 1548.45m	9.0	2.7	75.0	9.9	309	41	2.8	< 1	< 0.1	< 1	< 0.1	< 0.1	131	11.0	24.2	3.0	11.2	2.1	2.1	0.3	1.9	7	0.5
Victoria Plains 1661.8m	0.3	< 0.1	19.2	0.3	259	2	0.2	< 1	< 0.1	< 1	< 0.1	< 0.1	5	0.4	0.9	0.1	0.3	< 0.1	< 0.1	< 0.1	< 0.1	1	0.3
Victoria Plains 1663.25m	0.2	4.2	2.5	0.2	196	1	0.1	< 1	< 0.1	< 1	< 0.1	< 0.1	5	0.6	0.9	< 0.1	0.3	< 0.1	< 0.1	< 0.1	< 0.1	< 1	0.3
FT SASK 7-23 1650.35m	18.6	1.5	141	11.5	218	47	11.5	< 1	< 0.1	2	0.1	< 0.1	145	43.4	75.3	8.8	29.5	4.4	3.6	0.4	2.4	45	0.3
FT SASK 7-23 1670.55m	12.4	1.5	98.0	7.9	480	46	8.1	< 1	< 0.1	1	0.1	< 0.1	160	24.8	42.2	5.0	17.1	2.6	2.2	0.3	1.6	7	< 0.1
FT SASK 7-23 1671.05m	13.1	11.1	104	5.8	684	51	8.4	< 1	< 0.1	1	0.4	< 0.1	137	18.4	30.0	3.1	10.1	1.8	1.5	0.2	1.1	22	0.3
FT SASK 7-23 1677.1m	16.5	9.1	152	7.6	746	58	12.3	< 1	< 0.1	2	0.2	< 0.1	209	28.1	46.5	5.0	16.7	2.4	2.0	0.3	1.6	31	0.1
FT SASK 7-23 1677.7m	16.9	1.2	155	7.6	927	55	11.8	< 1	< 0.1	2	0.1	< 0.1	205	28.6	46.4	5.3	17.0	2.5	2.1	0.3	1.5	7	0.1
FT SASK 7-23 1680.7m	10.3	1.3	68.6	5.2	498	33	6.5	< 1	< 0.1	1	0.1	< 0.1	225	15.1	26.8	2.9	10.2	1.8	1.3	0.2	0.9	5	< 0.1
FT SASK 7-23 1683.6m	10.5	1.1	74.4	7.9	518	32	2.6	< 1	< 0.1	1	< 0.1	< 0.1	139	22.1	37.4	4.5	15.7	2.2	2.1	0.3	1.6	11	0.1
FT SASK 7-23 1684.8m	12.5	3.4	93.9	9.4	630	35	2.5	< 1	< 0.1	1	< 0.1	< 0.1	166	29.1	50.1	6.2	20.7	3.2	2.5	0.3	1.8	17	0.2
FT SASK 7-23 1685.4m	18.7	8.1	159	8.9	800	64	12.7	2	< 0.1	2	0.3	< 0.1	224	33.4	51.6	5.9	19.1	2.4	2.4	0.3	1.8	36	0.3

Analyte Symbol	Ga	As	Rb	Y	Sr	Zr	Nb	Mo	In	Sn	Sb	Te	Ba	La	Ce	Pr	Nd	Sm	Gd	Tb	Dy	Cu	Ge
Unit Symbol	ppm	ppm	ppm	ppm	ppm	ppm	ppm	ppm	ppm	ppm	ppm	ppm	ppm	ppm	ppm	ppm	ppm	ppm	ppm	ppm	ppm	ppm	ppm
Lower Limit	0.1	0.1	0.2	0.1	0.2	1	0.1	1	0.1	1	0.1	0.1	1	0.1	0.1	0.1	0.1	0.1	0.1	0.1	0.1	1	0.1
Method Code	TD-MS	TD-MS	TD-MS	TD-MS	TD-MS	TD-MS	TD-MS	TD-ICP	TD-MS	TD-MS	TD-MS	TD-MS	TD-MS	TD-MS	TD-MS	TD-MS	TD-MS	TD-MS	TD-MS	TD-MS	TD-MS	TD-ICP	TD-MS
FT SASK 7-23 1692.3m	13.8	2.3	110	6.5	614	48	8.0	< 1	< 0.1	1	< 0.1	< 0.1	162	27.0	42.4	4.8	15.2	2.0	1.8	0.2	1.2	11	0.4
FT SASK 7-23 1692.9m	12.8	0.7	105	9.4	714	49	9.9	< 1	< 0.1	1	< 0.1	< 0.1	168	31.1	52.6	6.3	20.2	3.1	2.6	0.3	1.8	6	0.1
FT SASK 7-23 1698.95m	10.2	5.0	67.7	7.8	533	38	3.3	< 1	< 0.1	1	< 0.1	< 0.1	144	23.5	42.4	4.8	17.4	3.3	2.3	0.3	1.6	14	0.1
FT SASK 7-23 1699.85m	14.9	1.9	102	7.4	481	42	7.7	< 1	< 0.1	1	0.1	< 0.1	167	21.5	36.2	3.9	13.3	2.1	1.9	0.2	1.5	11	0.2
FT SASK 7-23 1701.05m	13.0	0.7	90.8	8.2	386	51	5.0	< 1	< 0.1	1	< 0.1	< 0.1	190	24.8	40.6	4.8	15.1	2.2	2.1	0.3	1.5	12	< 0.1
FT SASK 7-23 1705.5m	13.9	0.7	95.5	8.3	782	38	1.0	< 1	< 0.1	< 1	< 0.1	< 0.1	187	27.7	45.7	5.4	17.5	2.7	2.2	0.3	1.6	8	0.2
PMC 140 FTSASK 1714.7m	9.4	0.5	65.7	8.9	531	39	3.7	< 1	< 0.1	< 1	< 0.1	< 0.1	136	26.0	44.9	5.4	18.6	2.8	2.3	0.3	1.7	10	0.2
PMC 140 FTSASK 1716m	17.1	7.8	135	8.3	439	58	12.7	< 1	< 0.1	2	0.2	< 0.1	196	35.2	51.2	5.7	17.2	2.6	2.1	0.3	1.6	18	0.2

Analyte Symbol	Tm	Yb	Lu	Ta	W	Re	Tl	Pb	Sc	Th	U	Ti	P	S
Unit Symbol	ppm	ppm	ppm	ppm	ppm	ppm	ppm	ppm	ppm	ppm	ppm	%	%	%
Lower Limit	0.1	0.1	0.1	0.1	0.1	0.001	0.05	3	1	0.1	0.1	0.0005	0.001	0.01
Method Code	TD-MS	TD-MS	TD-MS	TD-MS	TD-MS	TD-MS	TD-MS	TD-ICP	TD-ICP	TD-MS	TD-MS	TD-ICP	TD-ICP	TD-ICP
Victoria Plains 1496.95m	< 0.1	0.3	< 0.1	0.1	0.4	< 0.001	0.10	< 3	3	1.7	0.5	0.0760	0.013	8.10
Victoria Plains 1497.8m	0.2	1.3	0.2	0.6	1.0	< 0.001	0.47	6	10	7.3	2.4	0.283	0.032	0.75
Victoria Plains 1498.3m	0.1	0.8	0.1	0.2	0.6	< 0.001	0.23	< 3	6	4.0	1.6	0.154	0.017	0.36
Victoria Plains 1498.8m	0.1	0.8	0.1	0.3	0.6	< 0.001	0.21	< 3	6	4.4	1.8	0.167	0.022	1.32
Victoria Plains 1499.4m	0.2	1.2	0.2	0.6	1.1	< 0.001	0.38	< 3	11	7.4	2.9	0.279	0.027	2.45
Victoria Plains 1500m	< 0.1	0.7	0.1	0.4	0.7	< 0.001	0.27	< 3	6	4.4	1.5	0.172	0.022	7.53
Victoria Plains 1502.87m	< 0.1	0.3	< 0.1	0.1	0.3	< 0.001	0.16	15	3	1.6	1.1	0.0690	0.008	9.05
Victoria Plains 1505.28m	< 0.1	< 0.1	< 0.1	< 0.1	< 0.1	< 0.001	< 0.05	< 3	< 1	< 0.1	< 0.1	< 0.0005	< 0.001	12.7
Victoria Plains 1505.87m	< 0.1	< 0.1	< 0.1	< 0.1	0.2	< 0.001	< 0.05	< 3	< 1	< 0.1	0.4	0.0006	< 0.001	0.38
Victoria Plains 1509.5m	< 0.1	< 0.1	< 0.1	< 0.1	0.1	< 0.001	< 0.05	10	< 1	< 0.1	0.3	0.0010	< 0.001	0.08
Victoria Plains 1514.8m	< 0.1	< 0.1	< 0.1	< 0.1	0.2	< 0.001	< 0.05	< 3	< 1	0.2	0.8	0.0060	0.002	0.11
Victoria Plains 1515.4m	< 0.1	< 0.1	< 0.1	< 0.1	0.2	< 0.001	< 0.05	< 3	< 1	< 0.1	0.6	0.0022	0.001	0.09
Victoria Plains 1517.2m	< 0.1	< 0.1	< 0.1	< 0.1	0.1	< 0.001	< 0.05	3	< 1	0.3	0.8	0.0099	0.003	0.12
Victoria Plains 1521.75m	< 0.1	< 0.1	< 0.1	< 0.1	0.1	< 0.001	< 0.05	< 3	< 1	0.2	0.4	0.0070	0.003	0.08
Victoria Plains 1523.7m	< 0.1	< 0.1	< 0.1	< 0.1	0.1	< 0.001	< 0.05	< 3	< 1	0.1	0.5	0.0037	0.002	0.10
Victoria Plains 1524.75m	< 0.1	< 0.1	< 0.1	< 0.1	0.1	< 0.001	< 0.05	< 3	< 1	0.3	0.5	0.0111	0.004	0.09
Victoria Plains 1526.95m	< 0.1	< 0.1	< 0.1	< 0.1	< 0.1	< 0.001	< 0.05	< 3	< 1	0.2	0.5	0.0071	0.003	0.08
Victoria Plains 1548.45m	0.2	1.1	0.1	< 0.1	0.3	< 0.001	0.22	< 3	7	5.0	1.2	0.159	0.028	0.10
Victoria Plains 1661.8m	< 0.1	< 0.1	< 0.1	< 0.1	0.3	< 0.001	< 0.05	< 3	< 1	0.1	2.7	0.0035	< 0.001	1.42
Victoria Plains 1663.25m	< 0.1	< 0.1	< 0.1	< 0.1	0.2	< 0.001	< 0.05	< 3	< 1	< 0.1	1.0	0.0025	< 0.001	1.12
FT SASK 7-23 1650.35m	0.2	1.2	0.2	0.7	1.3	< 0.001	0.50	< 3	13	11.6	3.0	0.269	0.049	1.76
FT SASK 7-23 1670.55m	0.1	0.9	0.1	0.5	0.7	< 0.001	0.28	< 3	8	8.1	2.0	0.194	0.030	1.69
FT SASK 7-23 1671.05m	< 0.1	0.7	< 0.1	0.6	0.7	0.001	0.36	4	7	7.8	2.2	0.199	0.027	8.73
FT SASK 7-23 1677.1m	0.1	0.9	0.1	0.8	0.9	< 0.001	0.48	18	11	11.1	2.7	0.270	0.018	0.56
FT SASK 7-23 1677.7m	0.1	0.8	0.1	0.7	0.9	< 0.001	0.42	< 3	12	10.0	2.2	0.281	0.026	0.65
FT SASK 7-23 1680.7m	< 0.1	0.6	< 0.1	0.4	0.7	< 0.001	0.24	< 3	6	6.0	1.5	0.155	0.037	1.06
FT SASK 7-23 1683.6m	0.1	0.8	0.1	< 0.1	0.2	< 0.001	0.27	6	7	7.1	1.5	0.154	0.030	0.30
FT SASK 7-23 1684.8m	0.1	0.9	0.1	< 0.1	0.2	< 0.001	0.35	9	8	8.7	1.9	0.166	0.029	0.37
FT SASK 7-23 1685.4m	0.1	1.0	0.2	0.6	1.0	< 0.001	0.56	29	10	11.0	2.8	0.292	0.030	0.53

Analyte Symbol	Tm	Yb	Lu	Ta	W	Re	Tl	Pb	Sc	Th	U	Ti	P	S
Unit Symbol	ppm	ppm	ppm	ppm	ppm	ppm	ppm	ppm	ppm	ppm	ppm	%	%	%
Lower Limit	0.1	0.1	0.1	0.1	0.1	0.001	0.05	3	1	0.1	0.1	0.0005	0.001	0.01
Method Code	TD-MS	TD-MS	TD-MS	TD-MS	TD-MS	TD-MS	TD-MS	TD-ICP	TD-ICP	TD-MS	TD-MS	TD-ICP	TD-ICP	TD-ICP
FT SASK 7-23 1692.3m	0.1	0.7	0.1	0.3	0.4	< 0.001	0.34	5	8	8.6	1.7	0.204	0.022	0.25
FT SASK 7-23 1692.9m	0.1	0.9	0.1	0.5	0.7	< 0.001	0.31	< 3	8	8.7	1.6	0.198	0.026	0.17
FT SASK 7-23 1698.95m	0.1	0.8	0.1	< 0.1	0.2	0.002	0.28	7	6	7.5	1.8	0.155	0.028	0.39
FT SASK 7-23 1699.85m	0.1	0.8	0.1	0.5	0.6	< 0.001	0.37	< 3	8	8.0	2.6	0.205	0.026	0.21
FT SASK 7-23 1701.05m	0.1	0.9	0.1	< 0.1	0.3	< 0.001	0.34	4	9	9.1	2.9	0.219	0.036	0.22
FT SASK 7-23 1705.5m	0.1	0.9	0.1	< 0.1	0.2	< 0.001	0.36	5	9	9.8	2.4	0.135	0.022	0.08
PMC 140 FTSASK 1714.7m	0.1	0.8	0.1	< 0.1	0.3	< 0.001	0.24	5	6	7.4	1.4	0.147	0.025	0.32
PMC 140 FTSASK 1716m	0.1	0.9	0.2	0.7	1.0	< 0.001	0.47	10	9	9.4	3.2	0.252	0.054	0.58

Analyte Symbol	Li	Na	Mg	Al	K	Ca	Cd	V	Cr	Mn	Fe	Hf	Ni	Er	Be	Ho	Ag	Cs	Co	Eu	Bi	Se	Zn
Unit Symbol	ppm	%	%	%	%	%	ppm	ppm	ppm	ppm	%	ppm	ppm	ppm	ppm	ppm	ppm	ppm	ppm	ppm	ppm	ppm	ppm
Lower Limit	1	0.01	0.01	0.01	0.01	0.01	0.3	1	1	1	0.01	0.1	1	0.1	0.1	0.1	0.3	0.05	1	0.05	0.02	0.1	1
Method Code	TD-ICP	TD-MS	TD-MS	TD-ICP	TD-MS	TD-MS	TD-ICP	TD-MS	TD-MS	TD-MS	TD-MS	TD-MS	TD-ICP	TD-MS	TD-MS	TD-MS	TD-ICP	TD-MS	TD-ICP	TD-MS	TD-MS	TD-MS	TD-ICP
OREAS 101b (4 Acid) Meas			1.22		1.96			73		912	10.2		9	14.1		5.0			44	7.91			
OREAS 101b (4 Acid) Cert			1.23		2.36			77		927	10.7		8.2	15		5.2			45	8.1			
OREAS 101b (4 Acid) Meas			1.24		2.66			78		977	10.8			13.9		4.9				7.04			
OREAS 101b (4 Acid) Cert			1.23		2.36			77		927	10.7			15		5.2				8.1			
OREAS 903 (4 Acid) Meas	18	0.04	0.81	5.71	3.80	0.66	0.3	80	76	656	4.28	4.5	56		4.7		< 0.3	3.54	142		9.27	5.3	25
OREAS 903 (4 Acid) Cert	18.3	0.0300	0.714	5.89	3.31	0.625	0.200	74.0	73.0	690	4.16	4.56	54.0		4.42		0.432	3.57	131		8.90	6.06	24.3
OREAS 903 (4 Acid) Meas	18	0.04	0.68	5.70	3.70	0.60	< 0.3	77	74	644	3.99	4.4	54		4.4		0.3	3.75	144		9.06	6.0	26
OREAS 903 (4 Acid) Cert	18.3	0.0300	0.714	5.89	3.31	0.625	0.200	74.0	73.0	690	4.16	4.56	54.0		4.42		0.432	3.57	131		8.90	6.06	24.3
OREAS 903 (4 Acid) Meas	17			5.62			< 0.3						54				0.5		144				26
OREAS 903 (4 Acid) Cert	18.3			5.89			0.200						54.0				0.432		131				24.3
Oreas 77b (4 Acid) Meas	17	0.35	2.12	1.64	0.34	2.69	6.5	28	210	606	25.8	1.1	> 10000		0.5		1.0	2.17	1390		3.31		183
Oreas 77b (4 Acid) Cert	18.8	0.434	2.59	1.94	0.361	3.06	1.20	33.6	280	640	29.9	1.15	113000		0.470		1.62	2.32	1550		3.44		205
Oreas 72b (4 Acid) Meas	31	1.16	11.0	4.40	1.30	2.93	0.6	69	599	1090	7.21	2.5	6470		0.8		< 0.3	3.15	126		0.67		95
Oreas 72b (4 Acid) Cert	33.3	1.01	9.59	4.79	1.14	2.79	0.310	73.6	771	1010	6.84	2.51	6860		1.02		0.230	3.37	131		0.680		99.0
OREAS 683 (4 Acid) Meas	7	1.10	8.96	6.53	0.57	5.40	0.4	45	> 5000	1280	7.27	0.9	1100	0.8	0.6	0.3	< 0.3	1.26	79	0.60	0.16		82
OREAS 683 (4 Acid) Cert	6.51	1.03	8.63	7.15	0.507	5.23	0.072	187	7710	1200	7.32	0.75	1180	0.93	0.56	0.32	0.172	1.32	85	0.58	0.16		92
OREAS 681 (4 Acid) Meas		1.62	5.69		1.24	6.12		235	1540	1270	7.42	1.7		1.8	1.5	0.6		3.77		1.38	0.10		
OREAS 681 (4 Acid) Cert		1.61	5.19		1.35	5.98		253	1640	1310	7.47	1.70		1.97	1.41	0.690		4.02		1.37	0.0980		
OREAS 247 (4 Acid) Meas	32			5.80			0.4						47				1.8		12				84
OREAS 247 (4 Acid) Cert	31.8			6.08			0.0650						45.9				2.16		12.0				86.0
OREAS 247 (4 Acid) Meas	31			5.81			< 0.3						47				1.9		13				86
OREAS 247 (4 Acid) Cert	31.8			6.08			0.0650						45.9				2.16		12.0				86.0
OREAS 601c (4 acid) Meas	27	1.88	0.14	6.70	3.16	1.01	2.4	18	22	230	2.32	5.1	8	0.8	2.4	0.4	47.4	5.45	4	1.14	21.2	8.9	444
OREAS 601c (4 acid) Cert	26.7	1.94	0.169	7.06	2.72	0.953	2.77	15.5	17.5	230.000	2.41	4.93	6.83	0.78	2.46	0.38	50.3	5.17	4.99	1.08	21.1	8.75	425
OREAS 601c (4 acid) Meas		1.74	0.12		2.57	0.78		14	23	208	2.36	5.4		0.8	2.3	0.4		5.89		1.13	21.3	9.3	
OREAS 601c (4 acid) Cert		1.94	0.169		2.72	0.953		15.5	17.5	230.000	2.41	4.93		0.78	2.46	0.38		5.17		1.08	21.1	8.75	
OREAS 931 (4 Acid) Meas	26	0.21	1.52	5.75		0.48		75	58	957	11.4		29		2.0		14.7		47		191	42.0	467
OREAS 931 (4 Acid) Cert	24.0	0.201	1.50	5.96		0.453		74.0	58.0	950	11.3		28.8		1.99		14.0		46.9		204	43.5	480
OREAS 931 (4 Acid) Meas	26			5.72									29				14.2		47				472

Analyte Symbol	Li	Na	Mg	Al	K	Ca	Cd	V	Cr	Mn	Fe	Hf	Ni	Er	Be	Ho	Ag	Cs	Co	Eu	Bi	Se	Zn
Unit Symbol	ppm	%	%	%	%	%	ppm	ppm	ppm	ppm	%	ppm	ppm	ppm	ppm	ppm	ppm	ppm	ppm	ppm	ppm	ppm	ppm
Lower Limit	1	0.01	0.01	0.01	0.01	0.01	0.3	1	1	1	0.01	0.1	1	0.1	0.1	0.1	0.3	0.05	1	0.05	0.02	0.1	1
Method Code	TD-ICP	TD-MS	TD-MS	TD-ICP	TD-MS	TD-MS	TD-ICP	TD-MS	TD-MS	TD-MS	TD-MS	TD-MS	TD-ICP	TD-MS	TD-MS	TD-MS	TD-ICP	TD-MS	TD-ICP	TD-MS	TD-MS	TD-MS	TD-ICP
OREAS 931 (4 Acid) Cert	24.0			5.96									28.8				14.0		46.9				480
OREAS 750 (4 Acid) Meas	2290			5.31			0.6						13						3				64
OREAS 750 (4 Acid) Cert	2320.000			5.42			0.58						11.4						3.99				65
OREAS 625 (4 Acid) Meas	17			6.54			82.8						6				10.7		4				> 10000
OREAS 625 (4 Acid) Cert	17.9			6.68			83						5.98				11.7		4.21				31700.00
Victoria Plains 1521.75m Orig	< 1	> 3.00	0.70	0.11	0.20	31.0	< 0.3	2	7	49	0.08	< 0.1	2	< 0.1	< 0.1	< 0.1	< 0.3	0.08	< 1	< 0.05	< 0.02	0.3	2
Victoria Plains 1521.75m Dup	< 1	> 3.00	0.66	0.12	0.19	31.3	< 0.3	2	4	53	0.08	< 0.1	2	< 0.1	< 0.1	< 0.1	< 0.3	0.07	< 1	< 0.05	< 0.02	0.3	1
Method Blank	< 1	< 0.01	< 0.01	< 0.01	< 0.01	< 0.01	< 0.3	3	7	2	< 0.01	< 0.1	< 1	< 0.1	< 0.1	< 0.1	< 0.3	< 0.05	< 1	< 0.05	< 0.02	< 0.1	2
Method Blank	< 1			< 0.01			< 0.3						< 1				< 0.3		< 1				< 1
Method Blank	< 1	< 0.01	< 0.01	< 0.01	< 0.01	< 0.01	< 0.3	1	7	14	< 0.01	< 0.1	< 1	< 0.1	< 0.1	< 0.1	< 0.3	< 0.05	< 1	< 0.05	< 0.02	< 0.1	< 1
Method Blank	< 1	< 0.01	< 0.01	< 0.01	< 0.01	< 0.01	< 0.3	< 1	6	12	< 0.01	< 0.1	< 1	< 0.1	< 0.1	< 0.1	< 0.3	< 0.05	< 1	< 0.05	< 0.02	< 0.1	< 1
Method Blank	< 1			< 0.01			< 0.3						< 1				< 0.3		< 1				< 1
Method Blank	< 1			< 0.01			< 0.3						< 1				< 0.3		< 1				< 1
Method Blank	< 1	< 0.01	< 0.01	< 0.01	< 0.01	< 0.01	< 0.3	< 1	10	15	< 0.01	< 0.1	< 1	< 0.1	0.4	< 0.1	< 0.3	< 0.05	< 1	< 0.05	< 0.02	0.9	< 1
Method Blank	< 1	< 0.01	< 0.01	< 0.01	< 0.01	< 0.01	< 0.3	< 1	9	7	< 0.01	< 0.1	< 1	< 0.1	0.2	< 0.1	< 0.3	< 0.05	< 1	< 0.05	< 0.02	0.6	< 1
Method Blank	< 1			< 0.01			< 0.3						< 1				< 0.3		< 1				< 1

Analyte Symbol	Ga	As	Rb	Y	Sr	Zr	Nb	Mo	In	Sn	Sb	Te	Ba	La	Ce	Pr	Nd	Sm	Gd	Tb	Dy	Cu	Ge
Unit Symbol	ppm	ppm	ppm	ppm	ppm	ppm	ppm	ppm	ppm	ppm	ppm	ppm	ppm	ppm	ppm	ppm	ppm	ppm	ppm	ppm	ppm	ppm	ppm
Lower Limit	0.1	0.1	0.2	0.1	0.2	1	0.1	1	0.1	1	0.1	0.1	1	0.1	0.1	0.1	0.1	0.1	0.1	0.1	0.1	1	0.1
Method Code	TD-MS	TD-MS	TD-MS	TD-MS	TD-MS	TD-MS	TD-MS	TD-ICP	TD-MS	TD-MS	TD-MS	TD-MS	TD-MS	TD-MS	TD-MS	TD-MS	TD-MS	TD-MS	TD-MS	TD-MS	TD-MS	TD-ICP	TD-MS
OREAS 101b (4 Acid) Meas				127				19						803	1420	130	384	52.2	42.4	4.6	25.5	405	
OREAS 101b (4 Acid) Cert				133				20.1						754	1325	127	388	48	40	5.4	27	412	
OREAS 101b (4 Acid) Meas				128										767	1330	127	361	44.7	38.6	4.3	24.5		
OREAS 101b (4 Acid) Cert				133										754	1325	127	388	48	40	5.4	27		
OREAS 903 (4 Acid) Meas	16.1	53.9	132	21.7	75.4	151		4	0.1	3	1.4		199	43.3	81.2					0.7		6500	
OREAS 903 (4 Acid) Cert	15.0	49.7	137	22.5	77.0	152		4.32	0.160	2.63	1.57		197	40.0	82.0					0.830		6520	
OREAS 903 (4 Acid) Meas	16.3	55.4	154	22.5	79.1	157		5	0.1	3	1.7		209	45.7	87.0					0.7		6460	
OREAS 903 (4 Acid) Cert	15.0	49.7	137	22.5	77.0	152		4.32	0.160	2.63	1.57		197	40.0	82.0					0.830		6520	
OREAS 903 (4 Acid) Meas								4														6420	
OREAS 903 (4 Acid) Cert								4.32														6520	
Oreas 77b (4 Acid) Meas	3.9	1530	19.6	6.0	36.2	40	3.1		0.1	2	8.2	1.8	73	16.1	27.3							3150	
Oreas 77b (4 Acid) Cert	4.61	2050	19.1	6.55	34.4	37.9	3.26		0.112	1.59	9.100	1.35	118	15.8	27.7							3426	
Oreas 72b (4 Acid) Meas	11.4	159	51.9	12.1	64.3	82	5.5	4	< 0.1	2	0.9	< 0.1	341	25.3	43.0					0.4		217	
Oreas 72b (4 Acid) Cert	11.7	146	50.8	12.8	63.8	88.0	5.50	4.01	0.0490	1.43	0.870	0.0920	330	24.4	43.6					0.440		222	
OREAS 683 (4 Acid) Meas	13.4		28.8	7.4	282	30	2.7	1	< 0.1	< 1			181	8.5	17.5	2.2	8.6	2.0	1.6	0.2	1.5	395	
OREAS 683 (4 Acid) Cert	13.8		26.8	8.02	276	26	2.61	1.00	0.028	0.85			188	8.17	17.1	2.2	8.75	1.86	1.64	0.25	1.54	404	
OREAS 681 (4 Acid) Meas	17.1		78.6	16.1	466	52	3.5		< 0.1	1	0.1		410	19.0	38.2	5.0	20.9	4.8	3.9	0.6	3.3		
OREAS 681 (4 Acid) Cert	17.6		80.0	17.5	478	58.0	6.17		0.0420	1.89	0.240		442	18.8	40.6	5.32	21.9	4.82	4.06	0.580	3.40		
OREAS 247 (4 Acid) Meas								< 1														41	
OREAS 247 (4 Acid) Cert								1.76														42.2	
OREAS 247 (4 Acid) Meas								< 1														41	
OREAS 247 (4 Acid) Cert								1.76														42.2	
OREAS 601c (4 acid) Meas	26.9	436	134	12.0	229	188	13.9	4	0.6	5	10.0	4.9		32.5	73.6	8.6	32.8	6.3	4.8	0.6	2.7	1180	0.1
OREAS 601c (4 acid) Cert	23.5	390.000	115	11.5	230	178	14.7	3.66	0.56	4.23	37.2	7.50		37.1	75	8.41	29.8	5.76	4.65	0.55	2.66	1160.000	0.18
OREAS 601c (4 acid) Meas	23.9	414	146	13.0	237	202	15.7		0.6	5	10.4	7.7		37.9	78.5	9.8	34.6	6.2	5.1	0.6	2.9		0.1
OREAS 601c (4 acid) Cert	23.5	390.000	115	11.5	230	178	14.7		0.56	4.23	37.2	7.50		37.1	75	8.41	29.8	5.76	4.65	0.55	2.66		0.18
OREAS 931 (4 Acid) Meas		12.1		18.9	31.3		11.5			44	1.8			36.9								> 10000	
OREAS 931 (4 Acid) Cert		11.6		19.6	34.5		11.0			42.1	1.70			34.0								38200	
OREAS 931 (4 Acid) Meas																						> 10000	

Analyte Symbol	Ga	As	Rb	Y	Sr	Zr	Nb	Mo	In	Sn	Sb	Te	Ba	La	Ce	Pr	Nd	Sm	Gd	Tb	Dy	Cu	Ge
Unit Symbol	ppm	ppm	ppm	ppm	ppm	ppm	ppm	ppm	ppm	ppm	ppm	ppm	ppm	ppm	ppm	ppm	ppm	ppm	ppm	ppm	ppm	ppm	ppm
Lower Limit	0.1	0.1	0.2	0.1	0.2	1	0.1	1	0.1	1	0.1	0.1	1	0.1	0.1	0.1	0.1	0.1	0.1	0.1	0.1	1	0.1
Method Code	TD-MS	TD-MS	TD-MS	TD-MS	TD-MS	TD-MS	TD-MS	TD-ICP	TD-MS	TD-MS	TD-MS	TD-MS	TD-MS	TD-MS	TD-MS	TD-MS	TD-MS	TD-MS	TD-MS	TD-MS	TD-MS	TD-ICP	TD-MS
OREAS 931 (4 Acid) Cert																						38200	
OREAS 750 (4 Acid) Meas								3														21	
OREAS 750 (4 Acid) Cert								2.17														20.4	
OREAS 625 (4 Acid) Meas								13														1660	
OREAS 625 (4 Acid) Cert								12.3														1710.000	
Victoria Plains 1521.75m Orig	0.3	0.2	3.1	0.7	366	3	0.2	< 1	< 0.1	< 1	< 0.1	< 0.1	26	0.7	1.2	0.1	0.6	< 0.1	< 0.1	< 0.1	< 0.1	2	0.2
Victoria Plains 1521.75m Dup	0.3	< 0.1	3.3	0.8	384	3	0.3	< 1	< 0.1	< 1	< 0.1	< 0.1	26	0.8	1.3	0.2	0.6	< 0.1	0.1	< 0.1	< 0.1	2	0.2
Method Blank	0.3	< 0.1	< 0.2	< 0.1	< 0.2	< 1	< 0.1	< 1	< 0.1	< 1	< 0.1	< 0.1	< 1	< 0.1	< 0.1	< 0.1	< 0.1	< 0.1	< 0.1	< 0.1	< 0.1	< 1	< 0.1
Method Blank								< 1														< 1	
Method Blank	0.1	< 0.1	< 0.2	< 0.1	< 0.2	< 1	< 0.1	< 1	< 0.1	< 1	< 0.1	< 0.1	< 1	< 0.1	< 0.1	< 0.1	< 0.1	< 0.1	< 0.1	< 0.1	< 0.1	< 1	< 0.1
Method Blank	0.1	0.4	< 0.2	< 0.1	< 0.2	< 1	< 0.1	< 1	< 0.1	< 1	< 0.1	< 0.1	< 1	< 0.1	< 0.1	< 0.1	< 0.1	< 0.1	< 0.1	< 0.1	< 0.1	< 1	< 0.1
Method Blank								< 1														< 1	
Method Blank								< 1														< 1	
Method Blank	0.2	< 0.1	< 0.2	< 0.1	< 0.2	< 1	< 0.1	< 1	< 0.1	< 1	< 0.1	< 0.1	< 1	< 0.1	< 0.1	< 0.1	< 0.1	< 0.1	< 0.1	< 0.1	< 0.1	< 1	< 0.1
Method Blank	0.1	< 0.1	< 0.2	< 0.1	< 0.2	< 1	< 0.1	< 1	< 0.1	< 1	< 0.1	< 0.1	< 1	< 0.1	< 0.1	< 0.1	< 0.1	< 0.1	< 0.1	< 0.1	< 0.1	< 1	< 0.1
Method Blank								< 1														< 1	

Analyte Symbol	Tm	Yb	Lu	Ta	W	Re	Tl	Pb	Sc	Th	U	Ti	P	S
Unit Symbol	ppm	ppm	ppm	ppm	ppm	ppm	ppm	ppm	ppm	ppm	ppm	%	%	%
Lower Limit	0.1	0.1	0.1	0.1	0.1	0.001	0.05	3	1	0.1	0.1	0.0005	0.001	0.01
Method Code	TD-MS	TD-MS	TD-MS	TD-MS	TD-MS	TD-MS	TD-MS	TD-ICP	TD-ICP	TD-MS	TD-MS	TD-ICP	TD-ICP	TD-ICP
OREAS 101b (4 Acid) Meas	2.0	12.8	1.8					19		38.8	393	0.328	0.113	
OREAS 101b (4 Acid) Cert	2.08	13.9	1.96					23		36.4	387	0.35	0.1118	
OREAS 101b (4 Acid) Meas	2.0	12.2	1.8							37.9	380			
OREAS 101b (4 Acid) Cert	2.08	13.9	1.96							36.4	387			
OREAS 903 (4 Acid) Meas		2.3	0.4	0.8			0.63	7	11	14.4	7.6	0.324	0.104	0.49
OREAS 903 (4 Acid) Cert		2.36	0.360	0.540			0.620	11.3	10.2	13.6	7.58	0.192	0.107	0.500
OREAS 903 (4 Acid) Meas		2.2	0.3	0.7			0.63	6	11	14.3	7.7	0.296	0.111	0.49
OREAS 903 (4 Acid) Cert		2.36	0.360	0.540			0.620	11.3	10.2	13.6	7.58	0.192	0.107	0.500
OREAS 903 (4 Acid) Meas								8	11			0.303	0.112	0.50
OREAS 903 (4 Acid) Cert								11.3	10.2			0.192	0.107	0.500
Oreas 77b (4 Acid) Meas				0.3	3.1	0.019	1.35	58	3	6.5	1.7	0.0566		
Oreas 77b (4 Acid) Cert				0.280	3.07	0.0220	1.37	61.0	3.51	6.61	1.71	0.0640		
Oreas 72b (4 Acid) Meas				0.4	4.4		0.35	13	13	11.4	4.5	0.205	0.026	1.41
Oreas 72b (4 Acid) Cert				0.430	4.00		0.350	14.9	12.8	11.3	4.68	0.216	0.0260	1.49
OREAS 683 (4 Acid) Meas	0.1	0.8	0.1		1.4			5	20	2.5	0.5	0.248	0.047	0.19
OREAS 683 (4 Acid) Cert	0.13	0.88	0.13		1.23			10.2	19.7	2.42	0.58	0.263	0.050	0.205
OREAS 681 (4 Acid) Meas	0.3	1.7	0.3	0.1	0.7					7.5	1.5			
OREAS 681 (4 Acid) Cert	0.280	1.77	0.270	0.420	1.09					6.55	1.44			
OREAS 247 (4 Acid) Meas								28	12			0.365	0.043	0.68
OREAS 247 (4 Acid) Cert								31.9	11.4			0.390	0.0480	0.714
OREAS 247 (4 Acid) Meas								27	12			0.406	0.049	0.72
OREAS 247 (4 Acid) Cert								31.9	11.4			0.390	0.0480	0.714
OREAS 601c (4 acid) Meas	< 0.1	0.5	< 0.1	0.7	4.5		1.88	287	4	10.3	4.6	0.149	0.039	1.58
OREAS 601c (4 acid) Cert	0.094	0.54	0.077	1.11	4.67		1.75	328	4.01	12.4	4.40	0.135	0.039	1.58
OREAS 601c (4 acid) Meas	< 0.1	0.5	< 0.1	0.6	4.4		1.82			9.6	4.5			
OREAS 601c (4 acid) Cert	0.094	0.54	0.077	1.11	4.67		1.75			12.4	4.40			
OREAS 931 (4 Acid) Meas					20.7			137		11.5		0.315	0.059	4.40
OREAS 931 (4 Acid) Cert					19.8			147		12.3		0.294	0.0510	4.12
OREAS 931 (4 Acid) Meas								137				0.323	0.059	4.43

Analyte Symbol	Tm	Yb	Lu	Ta	W	Re	Tl	Pb	Sc	Th	U	Ti	P	S
Unit Symbol	ppm	ppm	ppm	ppm	ppm	ppm	ppm	ppm	ppm	ppm	ppm	%	%	%
Lower Limit	0.1	0.1	0.1	0.1	0.1	0.001	0.05	3	1	0.1	0.1	0.0005	0.001	0.01
Method Code	TD-MS	TD-MS	TD-MS	TD-MS	TD-MS	TD-MS	TD-MS	TD-ICP	TD-ICP	TD-MS	TD-MS	TD-ICP	TD-ICP	TD-ICP
OREAS 931 (4 Acid) Cert								147				0.294	0.0510	4.12
OREAS 750 (4 Acid) Meas								9	4			0.159	0.069	0.07
OREAS 750 (4 Acid) Cert								13.8	3.72			0.158	0.070	0.073
OREAS 625 (4 Acid) Meas								> 5000	5			0.129	0.030	3.87
OREAS 625 (4 Acid) Cert								8220.000	4.60			0.120	0.030	3.80
Victoria Plains 1521.75m Orig	< 0.1	< 0.1	< 0.1	< 0.1	0.1	< 0.001	< 0.05	< 3	< 1	0.2	0.4	0.0069	0.003	0.08
Victoria Plains 1521.75m Dup	< 0.1	< 0.1	< 0.1	< 0.1	0.1	< 0.001	< 0.05	< 3	< 1	0.2	0.4	0.0071	0.003	0.08
Method Blank	< 0.1	< 0.1	< 0.1	< 0.1	0.5	< 0.001	< 0.05	< 3	< 1	< 0.1	< 0.1	< 0.0005	< 0.001	< 0.01
Method Blank								< 3	< 1			< 0.0005	< 0.001	< 0.01
Method Blank	< 0.1	< 0.1	< 0.1	< 0.1	0.2	< 0.001	< 0.05	< 3	< 1	< 0.1	< 0.1	< 0.0005	< 0.001	< 0.01
Method Blank	< 0.1	< 0.1	< 0.1	< 0.1	< 0.1	< 0.001	< 0.05	< 3	< 1	< 0.1	< 0.1	< 0.0005	< 0.001	< 0.01
Method Blank								< 3	< 1			< 0.0005	< 0.001	< 0.01
Method Blank								< 3	< 1			< 0.0005	< 0.001	< 0.01
Method Blank	< 0.1	< 0.1	< 0.1	< 0.1	0.2	0.004	< 0.05	< 3	< 1	< 0.1	< 0.1	< 0.0005	< 0.001	< 0.01
Method Blank	< 0.1	< 0.1	< 0.1	< 0.1	< 0.1	0.002	< 0.05	< 3	< 1	< 0.1	< 0.1	< 0.0005	< 0.001	< 0.01
Method Blank								< 3	< 1			< 0.0005	< 0.001	< 0.01
