# Supplementary material for: Geochemistry and X-ray diffraction data from rock salts and saltwork wastes of Canada: data compilation
Source: Data Brief. 2026 Jun 6;67:112941. doi: 10.1016/j.dib.2026.112941 (PMC13292661; doi:10.1016/j.dib.2026.112941)
Supplement: Supplementary file 12 [file mmc12.pdf]

Quality Analysis ... *Innovative Technologies*

**Report No.:**	**A25-06818**
**Report Date:**	**10-Jul-25**
**Date Submitted:**	**04-Jun-25**
**Your Reference:**	**CMGD**

Geological Survey of Canada-AB
3303 33rd St NW
Calgary Alberta T2-2A7
Canada

ATTN: Pavel Kabanov

CERTIFICATE OF ANALYSIS

54 Pulp samples were submitted for analysis.

The following analytical package(s) were requested:		Testing Date:
UT-6	QOP Total/QOP Ultratrace- 4acid Digest (Total Digestion ICPOES/ICPMS)	2025-07-01 16:18:45

REPORT **A25-06818**

This report may be reproduced without our consent. If only selected portions of the report are reproduced, permission must be obtained. If no instructions were given at time of sample submittal regarding excess material, it will be discarded within 90 days of this report. Our liability is limited solely to the analytical cost of these analyses. Test results are representative only of material submitted for analysis.

Notes:

Values which exceed the upper limit should be assayed for accurate numbers.

Refer to the Scope of Accreditation for information on accredited elements.

SCC Accredited LAB
LAB Accrédité CCN TM

CERTIFIED BY:

Mark Vandergeest
Quality Control Coordinator

ACTIVATION LABORATORIES LTD.
41 Bittern Street, Ancaster, Ontario, Canada, L9G 4V5
TELEPHONE +905 648-9611 or +1.888.228.5227 FAX +1.905.648.9613
E-MAIL Ancaster@actlabs.com ACTLABS GROUP WEBSITE www.actlabs.com

Analyte Symbol	Li	Na	Mg	Al	K	Ca	Cd	V	Cr	Mn	Fe	Hf	Ni	Er	Be	Ho	Ag	Cs	Co	Eu	Bi	Se	Zn
Unit Symbol	ppm	%	%	%	%	%	ppm	ppm	ppm	ppm	%	ppm	ppm	ppm	ppm	ppm	ppm	ppm	ppm	ppm	ppm	ppm	ppm
Lower Limit	1	0.01	0.01	0.01	0.01	0.01	0.3	1	1	1	0.01	0.1	1	0.1	0.1	0.1	0.3	0.05	1	0.05	0.02	0.1	1
Method Code	TD-ICP	TD-MS	TD-MS	TD-ICP	TD-MS	TD-MS	TD-ICP	TD-MS	TD-MS	TD-MS	TD-MS	TD-MS	TD-ICP	TD-MS	TD-MS	TD-MS	TD-ICP	TD-MS	TD-ICP	TD-MS	TD-MS	TD-MS	TD-ICP
PMC 140 FTSASK 1768.3m	6	0.30	11.8	0.29	0.22	20.2	< 0.3	17	9	269	0.45	0.1	15	0.1	0.1	< 0.1	< 0.3	0.21	< 1	0.13	< 0.02	0.9	6
PMC 140 FTSASK 1769.04m	16	0.42	11.3	1.21	1.22	17.8	< 0.3	20	16	177	0.74	1.0	16	0.5	0.3	0.2	< 0.3	0.83	2	0.29	0.08	0.8	10
PMC 140 FTSASK 1769.5m	81	0.17	6.96	5.72	2.06	9.18	< 0.3	32	24	203	2.18	1.3	25	0.9	1.7	0.3	< 0.3	4.25	8	0.51	0.17	< 0.1	33
PMC 140 FTSASK 1770.35m	24	0.09	10.6	1.85	1.63	16.7	< 0.3	20	12	224	1.11	0.5	10	0.6	0.6	0.2	< 0.3	1.23	4	0.42	0.06	0.6	15
PMC 140 FTSASK 1771m	59	0.14	7.69	4.36	3.83	11.4	< 0.3	46	21	229	1.76	1.3	22	0.8	1.3	0.3	< 0.3	3.25	8	0.54	0.07	0.5	27
PMC 140 FTSASK 1771.65m	55	0.16	8.18	4.31	3.60	11.9	< 0.3	38	17	204	1.81	1.5	22	0.8	1.2	0.3	< 0.3	2.67	11	0.48	0.13	0.4	24
PMC 140 FTSASK 1772.2m	59	0.12	7.92	4.32	3.83	11.3	< 0.3	41	17	219	1.78	1.5	19	1.0	1.2	0.3	< 0.3	2.83	8	0.55	0.10	0.3	24
PMC 140 FTSASK 1773.4m	56	0.17	9.36	3.48	3.07	13.6	< 0.3	32	13	201	1.26	1.0	15	0.7	1.0	0.2	< 0.3	2.28	6	0.43	0.07	0.2	21
PMC 140 FTSASK 1774.05m	52	0.14	8.71	3.50	1.82	12.8	< 0.3	35	29	189	1.35	1.2	16	0.9	1.1	0.3	< 0.3	2.19	7	0.44	0.08	0.5	27
PMC 140 FTSASK 1774.67m	24	0.14	11.7	1.13	1.10	17.9	< 0.3	16	15	207	0.47	0.3	5	0.3	0.4	0.1	< 0.3	0.70	2	0.24	0.03	0.3	14
PMC 140 FTSASK 1775m	24	0.16	11.8	1.10	1.05	17.9	< 0.3	15	11	212	0.47	0.3	5	0.3	0.4	0.1	< 0.3	0.68	2	0.26	0.04	0.3	14
PMC 140 FTSASK 1775.9m	43	0.19	10.6	2.21	1.92	15.3	< 0.3	20	10	230	0.90	1.0	22	0.6	0.7	0.2	< 0.3	1.48	13	0.40	0.16	< 0.1	17
PMC 140 FTSASK 1776.9m	22	0.12	12.0	1.23	1.18	18.0	< 0.3	17	8	222	0.53	0.4	6	0.4	0.5	0.2	< 0.3	0.74	3	0.27	0.07	0.3	14
PMC 140 FTSASK 1777.7m	34	0.23	11.4	1.95	1.59	16.7	< 0.3	22	10	241	0.80	0.6	12	0.6	0.7	0.2	< 0.3	1.33	4	0.35	0.09	0.2	19
PMC 140 FTSASK 1778.4m	55	0.15	10.1	3.22	2.50	13.8	< 0.3	26	14	256	1.19	1.2	16	0.9	0.9	0.3	< 0.3	2.98	4	0.46	0.03	0.3	30
PMC 140 FTSASK 1779.3m	76	0.29	8.51	4.83	3.81	10.8	< 0.3	19	19	247	1.91	0.8	28	1.0	1.7	0.3	< 0.3	3.42	11	0.57	0.39	0.1	29
PMC 140 FTSASK 1780.2m	48	0.17	9.95	3.24	2.49	14.2	< 0.3	27	16	236	1.34	1.0	15	0.8	1.2	0.3	< 0.3	2.39	4	0.46	0.04	0.2	22
PMC 140 FTSASK 1781.25m	39	0.16	9.94	2.81	2.05	14.2	< 0.3	24	16	226	1.16	1.0	12	0.7	0.9	0.3	< 0.3	2.04	3	0.46	0.02	0.4	19
PMC 140 FTSASK 1782.45m	33	0.16	10.7	2.37	1.94	15.1	< 0.3	22	12	229	1.02	1.0	23	0.7	0.9	0.2	< 0.3	1.58	3	0.41	0.03	0.3	20
PMC 140 FTSASK 1783.4m	36	0.12	10.7	2.22	2.11	15.2	< 0.3	23	9	237	0.88	0.8	10	0.6	0.8	0.2	< 0.3	1.44	3	0.39	0.03	0.4	15
PMC 140 FTSASK 1784.4m	18	0.11	12.5	0.81	0.83	18.7	< 0.3	15	6	228	0.40	0.3	6	0.4	0.3	0.1	< 0.3	0.64	2	0.25	0.24	0.3	9

Analyte Symbol	Li	Na	Mg	Al	K	Ca	Cd	V	Cr	Mn	Fe	Hf	Ni	Er	Be	Ho	Ag	Cs	Co	Eu	Bi	Se	Zn
Unit Symbol	ppm	%	%	%	%	%	ppm	ppm	ppm	ppm	%	ppm	ppm	ppm	ppm	ppm	ppm	ppm	ppm	ppm	ppm	ppm	ppm
Lower Limit	1	0.01	0.01	0.01	0.01	0.01	0.3	1	1	1	0.01	0.1	1	0.1	0.1	0.1	0.3	0.05	1	0.05	0.02	0.1	1
Method Code	TD-ICP	TD-MS	TD-MS	TD-ICP	TD-MS	TD-MS	TD-ICP	TD-MS	TD-MS	TD-MS	TD-MS	TD-MS	TD-ICP	TD-MS	TD-MS	TD-MS	TD-ICP	TD-MS	TD-ICP	TD-MS	TD-MS	TD-MS	TD-ICP
PMC 140 FTSASK 1785.15m	35	0.12	11.8	1.57	1.48	17.0	< 0.3	18	10	207	0.63	0.9	8	0.5	0.6	0.2	< 0.3	1.04	2	0.31	0.05	0.5	16
PMC 140 FTSASK 1785.85m	29	0.12	11.9	1.10	0.94	16.9	< 0.3	14	7	226	0.51	0.5	9	0.4	0.3	0.1	< 0.3	0.69	3	0.25	0.26	0.2	17
PMC 140 FTSASK 1786.8m	20	0.12	12.3	0.94	0.92	18.0	< 0.3	19	7	225	0.39	0.2	6	0.4	0.3	0.1	< 0.3	0.56	2	0.21	0.10	0.4	9
PMC 140 FTSASK 1787.7m	19	0.11	12.4	0.81	0.85	18.3	< 0.3	16	9	277	0.39	0.4	9	0.4	0.4	0.1	< 0.3	0.49	2	0.24	0.05	0.3	9
PMC 140 FTSASK 1788.5m	18	0.10	12.4	0.86	0.93	18.7	< 0.3	13	5	185	0.35	< 0.1	5	0.3	0.3	0.1	< 0.3	0.54	2	0.21	0.02	0.4	9
PMC 140 FTSASK 1789.45m	23	0.13	12.7	1.08	1.11	18.5	< 0.3	16	6	320	0.59	0.7	8	0.3	0.4	0.1	< 0.3	0.68	2	0.24	0.03	0.5	11
PMC 140 FTSASK 1790.45m	22	0.15	12.1	1.36	1.35	17.4	< 0.3	15	8	174	0.76	1.1	7	0.5	0.5	0.2	< 0.3	0.72	2	0.26	< 0.02	0.4	10
PMC 140 FTSASK 1791.4m	11	0.13	13.5	0.65	0.73	20.1	< 0.3	10	6	286	0.35	< 0.1	5	0.3	0.2	0.1	< 0.3	0.33	2	0.17	< 0.02	0.5	7
PMC 140 FTSASK 1792.4m	7	0.10	14.2	0.32	0.39	21.8	< 0.3	10	5	184	0.20	0.2	3	0.2	0.1	< 0.1	< 0.3	0.17	1	0.09	0.12	0.4	6
PMC 140 FTSASK 1793.1m	61	0.11	8.78	3.30	1.89	11.4	< 0.3	27	15	188	1.62	1.2	19	0.9	1.0	0.3	< 0.3	1.94	5	0.45	0.06	0.2	19
PMC 140 FTSASK 1793.7m	7	0.12	12.7	0.09	0.10	22.1	< 0.3	6	4	233	0.14	< 0.1	3	< 0.1	< 0.1	< 0.1	< 0.3	0.06	< 1	0.06	0.02	0.4	10
PMC 140 FTSASK 1794.55m	8	0.10	12.9	0.37	0.40	19.9	0.3	8	7	184	0.22	0.2	5	0.2	0.2	< 0.1	< 0.3	0.20	1	0.16	< 0.02	0.4	5
PMC 140 FTSASK 1795.25m	12	0.15	13.4	0.50	0.50	20.7	< 0.3	11	10	205	0.31	0.4	4	0.2	0.3	< 0.1	< 0.3	0.28	< 1	0.13	< 0.02	0.4	10
PMC 140 FTSASK 1796.08m	12	0.07	13.6	0.47	0.45	20.9	< 0.3	12	5	203	0.36	< 0.1	5	0.3	0.2	< 0.1	< 0.3	0.30	1	0.13	< 0.02	0.8	12
PMC 140 FTSASK 1796.8m	12	0.11	13.7	0.51	0.44	20.7	< 0.3	12	4	286	0.46	< 0.1	9	0.2	0.3	< 0.1	< 0.3	0.33	1	0.12	0.02	0.4	7
PMC 140 FTSASK 1797.4m	10	0.14	12.9	0.49	0.53	20.0	< 0.3	10	5	219	0.27	< 0.1	4	0.2	0.2	< 0.1	< 0.3	0.26	< 1	0.14	< 0.02	0.9	6
PMC 140 FTSASK 1798.2m	18	0.10	12.5	0.91	1.01	18.7	< 0.3	19	8	231	0.34	0.3	5	0.4	0.3	0.1	< 0.3	0.57	1	0.17	0.12	0.4	8
PMC 140 FTSASK 1799m	8	0.09	12.8	0.39	0.44	20.3	< 0.3	9	7	221	0.20	0.3	3	0.2	0.2	< 0.1	< 0.3	0.17	< 1	0.09	0.03	1.3	8
PMC 140 FTSASK 1799.7m	6	0.17	13.8	0.24	0.31	21.4	< 0.3	8	5	211	0.17	0.2	2	0.1	0.1	< 0.1	< 0.3	0.11	< 1	0.09	0.04	0.5	6
PMC 140 FTSASK 1800.6m	23	0.10	10.1	1.82	1.38	15.0	< 0.3	20	15	207	0.82	< 0.1	9	0.8	0.4	0.3	< 0.3	0.78	2	0.36	0.03	0.4	23
PMC 140 FTSASK 1801.45m	7	0.10	13.3	0.30	0.33	20.3	< 0.3	9	8	196	0.20	< 0.1	2	0.1	0.2	< 0.1	< 0.3	0.13	< 1	0.09	< 0.02	0.3	7

Analyte Symbol	Li	Na	Mg	Al	K	Ca	Cd	V	Cr	Mn	Fe	Hf	Ni	Er	Be	Ho	Ag	Cs	Co	Eu	Bi	Se	Zn
Unit Symbol	ppm	%	%	%	%	%	ppm	ppm	ppm	ppm	%	ppm	ppm	ppm	ppm	ppm	ppm	ppm	ppm	ppm	ppm	ppm	ppm
Lower Limit	1	0.01	0.01	0.01	0.01	0.01	0.3	1	1	1	0.01	0.1	1	0.1	0.1	0.1	0.3	0.05	1	0.05	0.02	0.1	1
Method Code	TD-ICP	TD-MS	TD-MS	TD-ICP	TD-MS	TD-MS	TD-ICP	TD-MS	TD-MS	TD-MS	TD-MS	TD-MS	TD-ICP	TD-MS	TD-MS	TD-MS	TD-ICP	TD-MS	TD-ICP	TD-MS	TD-MS	TD-MS	TD-ICP
PMC 140 FTSASK 1802.15m	9	0.13	13.6	0.46	0.51	20.8	< 0.3	9	4	198	0.28	< 0.1	4	0.2	0.3	< 0.1	< 0.3	0.24	< 1	0.15	< 0.02	0.5	5
PMC 140 FTSASK 1803m	8	0.16	13.8	0.43	0.51	21.1	< 0.3	11	4	197	0.28	< 0.1	3	0.2	0.2	< 0.1	< 0.3	0.23	< 1	0.11	< 0.02	0.5	6
PMC 140 FTSASK 1803.8m	9	0.24	13.6	0.32	0.34	21.2	< 0.3	8	5	190	0.20	0.2	2	0.2	0.2	< 0.1	< 0.3	0.16	< 1	0.10	< 0.02	1.6	6
PMC 140 FTSASK 1804.5m	8	0.28	13.2	0.24	0.25	19.8	< 0.3	6	4	199	0.15	< 0.1	2	0.2	0.1	< 0.1	< 0.3	0.13	1	0.09	< 0.02	0.5	6
PMC 140 FTSASK 1805.25m	75	0.17	10.2	2.96	2.06	12.0	< 0.3	29	15	186	1.57	1.9	19	0.9	1.0	0.3	< 0.3	1.65	5	0.59	0.04	0.1	22
PMC 140 FTSASK 1806.15m	23	0.13	11.4	1.08	1.04	17.0	< 0.3	12	12	201	0.62	< 0.1	6	0.5	0.4	0.2	< 0.3	0.54	2	0.31	< 0.02	0.2	9
PMC 140 FTSASK 1806.6m	18	0.13	12.2	0.51	0.54	18.2	< 0.3	12	5	180	0.18	< 0.1	3	0.3	0.2	0.1	< 0.3	0.23	1	0.17	0.03	0.6	7
PMC 140 FTSASK 1807.25m	19	0.15	12.5	0.85	0.80	18.3	< 0.3	12	5	219	0.42	0.5	5	0.4	0.3	0.2	< 0.3	0.46	2	0.21	< 0.02	0.5	9
PMC 140 FTSASK 1808m	55	0.15	11.3	2.01	1.35	14.5	< 0.3	19	10	205	1.30	0.9	13	0.8	0.7	0.3	< 0.3	1.17	4	0.43	< 0.02	0.1	14
PMC 140 FTSASK 1808.8m	76	0.15	10.9	2.63	1.74	13.1	< 0.3	23	12	200	1.73	1.5	16	0.9	0.9	0.3	< 0.3	1.58	5	0.56	0.05	0.1	18
PMC 140 FTSASK 1809.6m	63	0.16	11.1	2.45	1.69	13.6	< 0.3	15	11	198	1.44	1.9	15	0.9	0.8	0.3	< 0.3	1.32	5	0.50	0.04	< 0.1	16
PMC 140 FTSASK 1810.6m	46	0.20	10.8	2.28	1.54	13.9	< 0.3	22	16	235	1.36	0.9	13	0.8	0.8	0.3	< 0.3	1.32	5	0.48	< 0.02	< 0.1	16

Analyte Symbol	Ga	As	Rb	Y	Sr	Zr	Nb	Mo	In	Sn	Sb	Te	Ba	La	Ce	Pr	Nd	Sm	Gd	Tb	Dy	Cu	Ge
Unit Symbol	ppm	ppm	ppm	ppm	ppm	ppm	ppm	ppm	ppm	ppm	ppm	ppm	ppm	ppm	ppm	ppm	ppm	ppm	ppm	ppm	ppm	ppm	ppm
Lower Limit	0.1	0.1	0.2	0.1	0.2	1	0.1	1	0.1	1	0.1	0.1	1	0.1	0.1	0.1	0.1	0.1	0.1	0.1	0.1	1	0.1
Method Code	TD-MS	TD-MS	TD-MS	TD-MS	TD-MS	TD-MS	TD-MS	TD-ICP	TD-MS	TD-MS	TD-MS	TD-MS	TD-MS	TD-MS	TD-MS	TD-MS	TD-MS	TD-MS	TD-MS	TD-MS	TD-MS	TD-ICP	TD-MS
PMC 140 FTSASK 1768.3m	0.9	14.6	6.2	1.9	85.2	6	0.7	3	< 0.1	< 1	0.4	< 0.1	27	4.4	8.1	1.0	3.8	0.7	0.5	< 0.1	0.3	22	< 0.1
PMC 140 FTSASK 1769.04m	2.6	17.5	32.0	4.6	138	38	3.7	17	< 0.1	< 1	1.4	< 0.1	100	7.6	14.8	1.7	7.0	1.2	1.0	0.2	0.9	16	< 0.1
PMC 140 FTSASK 1769.5m	15.6	3.5	118	8.2	199	50	4.4	< 1	< 0.1	1	< 0.1	< 0.1	151	34.8	54.1	6.0	18.3	3.1	2.0	0.3	1.7	16	< 0.1
PMC 140 FTSASK 1770.35m	4.3	6.8	46.7	5.5	131	20	4.8	< 1	< 0.1	< 1	0.3	< 0.1	80	11.9	23.1	2.9	11.2	1.6	1.6	0.2	1.1	11	< 0.1
PMC 140 FTSASK 1771m	11.3	9.5	117	7.8	168	49	13.2	< 1	< 0.1	1	0.8	< 0.1	157	26.0	43.6	5.2	17.4	2.7	2.1	0.3	1.4	48	< 0.1
PMC 140 FTSASK 1771.65m	11.2	9.7	111	7.6	188	55	13.7	< 1	< 0.1	1	0.2	< 0.1	153	30.7	48.6	5.3	15.7	2.8	1.9	0.3	1.4	48	< 0.1
PMC 140 FTSASK 1772.2m	11.4	3.5	112	8.7	184	58	14.9	1	< 0.1	1	0.2	< 0.1	160	31.2	50.3	5.5	18.4	2.5	2.1	0.3	1.6	14	0.1
PMC 140 FTSASK 1773.4m	9.1	1.3	86.1	7.0	159	38	1.2	< 1	< 0.1	< 1	< 0.1	< 0.1	136	22.1	37.1	4.0	13.8	2.5	1.7	0.2	1.3	17	< 0.1
PMC 140 FTSASK 1774.05m	8.9	8.4	69.9	7.2	150	45	4.0	< 1	< 0.1	< 1	< 0.1	< 0.1	125	21.1	36.1	3.9	13.2	1.7	1.6	0.2	1.3	13	< 0.1
PMC 140 FTSASK 1774.67m	2.8	2.1	26.8	3.6	130	14	3.4	< 1	< 0.1	< 1	0.1	< 0.1	48	7.5	15.7	1.9	7.2	1.2	0.9	0.1	0.6	5	< 0.1
PMC 140 FTSASK 1775m	2.7	1.3	26.5	3.7	131	13	3.2	< 1	< 0.1	< 1	< 0.1	< 0.1	51	7.6	15.6	1.9	6.9	1.3	1.0	0.1	0.6	6	< 0.1
PMC 140 FTSASK 1775.9m	5.4	3.1	56.3	5.9	112	35	0.8	< 1	< 0.1	< 1	0.1	< 0.1	118	14.6	27.6	3.2	11.3	2.3	1.5	0.2	1.1	10	< 0.1
PMC 140 FTSASK 1776.9m	3.0	1.0	29.6	4.1	109	17	3.8	< 1	< 0.1	< 1	< 0.1	< 0.1	56	8.8	18.5	2.3	8.4	1.2	1.0	0.2	0.7	8	< 0.1
PMC 140 FTSASK 1777.7m	4.8	1.2	51.8	5.6	135	26	5.0	< 1	< 0.1	< 1	< 0.1	< 0.1	97	15.5	27.6	3.0	10.6	1.6	1.5	0.2	1.1	9	< 0.1
PMC 140 FTSASK 1778.4m	8.0	0.1	88.6	7.6	134	44	2.7	< 1	< 0.1	< 1	< 0.1	< 0.1	141	23.2	39.5	4.5	14.5	2.7	2.0	0.3	1.5	7	0.2
PMC 140 FTSASK 1779.3m	12.9	1.3	121	9.3	168	30	1.0	< 1	< 0.1	< 1	< 0.1	< 0.1	176	41.1	62.9	6.4	20.9	3.1	2.2	0.3	2.0	32	0.1
PMC 140 FTSASK 1780.2m	8.6	1.2	88.0	7.2	148	37	1.6	< 1	< 0.1	< 1	< 0.1	< 0.1	117	29.4	48.0	5.0	15.8	2.3	2.1	0.3	1.4	9	0.2
PMC 140 FTSASK 1781.25m	7.1	0.5	77.1	6.7	146	37	1.2	< 1	< 0.1	< 1	< 0.1	< 0.1	106	25.5	42.1	4.4	14.5	2.3	1.8	0.2	1.2	13	0.1
PMC 140 FTSASK 1782.45m	6.0	< 0.1	64.5	6.3	126	39	4.8	< 1	< 0.1	< 1	< 0.1	< 0.1	122	20.8	34.9	3.8	12.3	2.2	1.7	0.2	1.3	25	0.1
PMC 140 FTSASK 1783.4m	5.6	1.0	57.1	6.1	111	35	4.8	< 1	< 0.1	< 1	< 0.1	< 0.1	91	18.1	31.8	3.3	11.0	1.7	1.5	0.2	1.1	6	0.1
PMC 140 FTSASK 1784.4m	2.2	1.5	23.6	3.5	128	14	4.8	< 1	< 0.1	< 1	< 0.1	< 0.1	41	9.2	18.7	2.1	8.1	1.4	0.9	0.1	0.7	7	< 0.1

Analyte Symbol	Ga	As	Rb	Y	Sr	Zr	Nb	Mo	In	Sn	Sb	Te	Ba	La	Ce	Pr	Nd	Sm	Gd	Tb	Dy	Cu	Ge
Unit Symbol	ppm	ppm	ppm	ppm	ppm	ppm	ppm	ppm	ppm	ppm	ppm	ppm	ppm	ppm	ppm	ppm	ppm	ppm	ppm	ppm	ppm	ppm	ppm
Lower Limit	0.1	0.1	0.2	0.1	0.2	1	0.1	1	0.1	1	0.1	0.1	1	0.1	0.1	0.1	0.1	0.1	0.1	0.1	0.1	1	0.1
Method Code	TD-MS	TD-MS	TD-MS	TD-MS	TD-MS	TD-MS	TD-MS	TD-ICP	TD-MS	TD-MS	TD-MS	TD-MS	TD-MS	TD-MS	TD-MS	TD-MS	TD-MS	TD-MS	TD-MS	TD-MS	TD-MS	TD-ICP	TD-MS
PMC 140 FTSASK 1785.15m	3.9	1.4	46.4	5.2	145	36	4.3	< 1	< 0.1	< 1	< 0.1	< 0.1	120	15.0	26.0	2.8	9.4	1.5	1.2	0.2	0.9	28	0.1
PMC 140 FTSASK 1785.85m	2.7	0.5	29.6	3.9	101	22	6.9	< 1	< 0.1	< 1	< 0.1	< 0.1	57	10.9	19.4	2.1	7.6	1.3	1.0	0.1	0.6	13	< 0.1
PMC 140 FTSASK 1786.8m	2.1	1.0	25.6	3.3	140	9	5.2	< 1	< 0.1	< 1	< 0.1	< 0.1	70	9.4	17.0	1.8	6.2	0.9	0.8	0.1	0.6	9	< 0.1
PMC 140 FTSASK 1787.7m	2.1	0.4	21.7	3.5	119	20	7.3	< 1	< 0.1	< 1	< 0.1	< 0.1	47	11.2	20.0	2.1	7.3	1.1	0.9	0.1	0.6	7	< 0.1
PMC 140 FTSASK 1788.5m	2.0	0.5	24.9	3.5	139	5	3.3	< 1	< 0.1	< 1	< 0.1	< 0.1	82	9.6	17.1	1.9	6.7	0.9	0.7	0.1	0.7	7	0.1
PMC 140 FTSASK 1789.45m	2.8	1.2	32.4	4.1	107	29	7.6	< 1	< 0.1	< 1	< 0.1	< 0.1	75	11.7	19.3	2.2	7.1	1.1	0.8	0.1	0.7	11	0.1
PMC 140 FTSASK 1790.45m	3.1	1.3	35.3	4.9	106	44	8.6	< 1	< 0.1	< 1	0.1	< 0.1	140	9.9	15.3	1.9	6.7	1.2	1.0	0.2	0.9	8	0.1
PMC 140 FTSASK 1791.4m	1.5	1.7	17.8	3.0	121	2	2.1	< 1	< 0.1	< 1	< 0.1	< 0.1	68	5.9	11.9	1.3	4.5	0.6	0.6	< 0.1	0.5	5	0.1
PMC 140 FTSASK 1792.4m	0.7	< 0.1	9.1	1.8	204	9	2.1	< 1	< 0.1	< 1	< 0.1	< 0.1	107	3.6	6.4	0.7	2.6	0.7	0.4	< 0.1	0.3	8	0.2
PMC 140 FTSASK 1793.1m	7.8	5.0	61.7	8.7	135	50	1.0	< 1	< 0.1	< 1	< 0.1	< 0.1	163	13.9	29.5	3.5	12.8	2.4	1.9	0.3	1.6	10	< 0.1
PMC 140 FTSASK 1793.7m	< 0.1	0.4	2.2	1.0	235	3	0.3	< 1	< 0.1	< 1	< 0.1	< 0.1	87	2.3	4.2	0.4	1.7	0.2	0.2	< 0.1	0.2	8	0.1
PMC 140 FTSASK 1794.55m	< 0.1	0.5	9.6	1.6	191	10	2.4	< 1	< 0.1	< 1	0.1	< 0.1	847	5.6	11.5	1.3	4.4	0.8	0.6	< 0.1	0.4	7	0.1
PMC 140 FTSASK 1795.25m	1.3	1.2	13.2	2.2	153	15	3.9	< 1	< 0.1	< 1	< 0.1	< 0.1	84	5.4	9.9	1.1	3.8	0.7	0.5	< 0.1	0.4	6	0.1
PMC 140 FTSASK 1796.08m	1.4	1.7	11.7	2.0	130	2	1.2	< 1	< 0.1	< 1	< 0.1	< 0.1	41	5.4	11.7	1.3	4.3	0.8	0.5	< 0.1	0.3	21	0.1
PMC 140 FTSASK 1796.8m	1.6	0.7	12.7	2.1	102	2	1.8	< 1	< 0.1	< 1	< 0.1	< 0.1	34	5.1	9.6	1.0	3.5	0.4	0.5	< 0.1	0.4	9	< 0.1
PMC 140 FTSASK 1797.4m	1.2	1.2	11.7	2.1	117	1	0.8	< 1	< 0.1	< 1	< 0.1	< 0.1	85	7.0	15.1	1.6	5.7	0.6	0.6	< 0.1	0.3	4	0.1
PMC 140 FTSASK 1798.2m	2.6	0.6	24.3	3.2	101	13	2.9	< 1	< 0.1	< 1	< 0.1	< 0.1	101	6.9	12.7	1.3	4.9	0.6	0.7	0.1	0.6	5	0.2
PMC 140 FTSASK 1799m	0.9	1.5	10.1	1.6	101	15	2.2	< 1	< 0.1	< 1	< 0.1	< 0.1	58	4.3	7.2	0.8	2.7	0.3	0.3	< 0.1	0.3	4	< 0.1
PMC 140 FTSASK 1799.7m	0.7	1.1	6.2	1.3	101	8	1.2	< 1	< 0.1	< 1	< 0.1	< 0.1	35	4.0	7.6	0.9	2.7	0.4	0.3	< 0.1	0.3	2	0.2
PMC 140 FTSASK 1800.6m	3.7	2.0	41.4	6.7	136	5	0.3	< 1	< 0.1	< 1	< 0.1	< 0.1	248	14.8	30.7	3.3	11.7	1.8	1.4	0.2	1.1	17	< 0.1
PMC 140 FTSASK 1801.45m	0.7	0.1	7.3	1.6	94.3	2	1.2	< 1	< 0.1	< 1	< 0.1	< 0.1	47	4.2	7.0	0.8	2.7	0.2	0.4	< 0.1	0.3	3	0.2

Analyte Symbol	Ga	As	Rb	Y	Sr	Zr	Nb	Mo	In	Sn	Sb	Te	Ba	La	Ce	Pr	Nd	Sm	Gd	Tb	Dy	Cu	Ge
Unit Symbol	ppm	ppm	ppm	ppm	ppm	ppm	ppm	ppm	ppm	ppm	ppm	ppm	ppm	ppm	ppm	ppm	ppm	ppm	ppm	ppm	ppm	ppm	ppm
Lower Limit	0.1	0.1	0.2	0.1	0.2	1	0.1	1	0.1	1	0.1	0.1	1	0.1	0.1	0.1	0.1	0.1	0.1	0.1	0.1	1	0.1
Method Code	TD-MS	TD-MS	TD-MS	TD-MS	TD-MS	TD-MS	TD-MS	TD-ICP	TD-MS	TD-MS	TD-MS	TD-MS	TD-MS	TD-MS	TD-MS	TD-MS	TD-MS	TD-MS	TD-MS	TD-MS	TD-MS	TD-ICP	TD-MS
PMC 140 FTSASK 1802.15m	1.2	1.3	12.5	2.4	108	4	4.2	< 1	< 0.1	< 1	< 0.1	< 0.1	54	7.5	12.7	1.3	4.1	0.8	0.6	< 0.1	0.5	5	0.2
PMC 140 FTSASK 1803m	1.2	1.2	11.1	1.9	107	3	2.5	< 1	< 0.1	< 1	< 0.1	< 0.1	58	5.3	9.9	1.0	3.7	0.6	0.5	< 0.1	0.3	3	0.2
PMC 140 FTSASK 1803.8m	0.9	0.8	8.2	1.6	112	10	2.8	< 1	< 0.1	< 1	< 0.1	< 0.1	45	5.0	8.6	0.8	2.9	0.5	0.4	< 0.1	0.3	12	0.2
PMC 140 FTSASK 1804.5m	0.6	0.7	5.9	1.6	97.6	3	1.6	< 1	< 0.1	< 1	< 0.1	< 0.1	22	4.9	8.1	0.8	2.9	0.4	0.4	< 0.1	0.2	4	0.2
PMC 140 FTSASK 1805.25m	7.1	2.0	71.7	9.1	136	73	19.4	< 1	< 0.1	< 1	0.3	< 0.1	223	31.1	54.9	5.5	18.8	3.1	2.4	0.3	1.7	60	< 0.1
PMC 140 FTSASK 1806.15m	2.0	1.6	31.5	4.3	125	6	3.5	< 1	< 0.1	< 1	< 0.1	< 0.1	113	11.9	21.9	2.3	8.1	1.3	1.1	0.1	0.9	3	< 0.1
PMC 140 FTSASK 1806.6m	1.0	0.4	12.9	2.8	125	2	2.1	< 1	< 0.1	< 1	< 0.1	< 0.1	65	6.7	12.1	1.3	4.3	1.1	0.8	< 0.1	0.5	1	0.2
PMC 140 FTSASK 1807.25m	2.0	2.4	23.8	3.7	113	21	5.2	< 1	< 0.1	< 1	0.1	< 0.1	73	8.9	15.5	1.6	5.7	1.0	0.9	0.1	0.7	2	0.1
PMC 140 FTSASK 1808m	4.5	2.1	54.9	7.1	134	43	3.7	< 1	< 0.1	< 1	< 0.1	< 0.1	137	21.6	34.0	3.7	12.7	2.0	1.7	0.2	1.3	3	< 0.1
PMC 140 FTSASK 1808.8m	5.7	2.9	64.7	8.5	165	66	24.3	< 1	< 0.1	< 1	0.2	< 0.1	156	32.0	48.0	5.1	16.5	2.4	2.0	0.3	1.7	6	0.1
PMC 140 FTSASK 1809.6m	5.2	1.8	61.6	8.3	126	74	5.0	< 1	< 0.1	< 1	0.1	< 0.1	192	28.4	47.1	4.7	16.2	2.9	2.1	0.3	1.6	3	< 0.1
PMC 140 FTSASK 1810.6m	4.6	2.0	64.3	7.6	137	39	4.5	< 1	< 0.1	< 1	< 0.1	< 0.1	212	21.4	35.0	4.0	13.4	2.6	1.9	0.3	1.4	3	< 0.1

Analyte Symbol	Tm	Yb	Lu	Ta	W	Re	Tl	Pb	Sc	Th	U	Ti	P	S
Unit Symbol	ppm	ppm	ppm	ppm	ppm	ppm	ppm	ppm	ppm	ppm	ppm	%	%	%
Lower Limit	0.1	0.1	0.1	0.1	0.1	0.001	0.05	3	1	0.1	0.1	0.0005	0.001	0.01
Method Code	TD-MS	TD-MS	TD-MS	TD-MS	TD-MS	TD-MS	TD-MS	TD-ICP	TD-ICP	TD-MS	TD-MS	TD-ICP	TD-ICP	TD-ICP
PMC 140 FTSASK 1768.3m	< 0.1	0.1	< 0.1	< 0.1	< 0.1	0.018	0.27	6	< 1	0.6	2.1	0.0177	0.004	0.22
PMC 140 FTSASK 1769.04m	< 0.1	0.4	< 0.1	0.2	0.4	0.019	0.58	15	2	3.1	3.1	0.0636	0.009	0.48
PMC 140 FTSASK 1769.5m	0.1	0.9	0.1	< 0.1	< 0.1	< 0.001	0.34	10	9	10.5	2.1	0.154	0.019	0.29
PMC 140 FTSASK 1770.35m	< 0.1	0.5	< 0.1	0.3	0.3	< 0.001	0.20	15	3	3.4	0.8	0.0671	0.010	0.61
PMC 140 FTSASK 1771m	0.1	0.7	0.1	0.8	0.6	< 0.001	0.37	14	7	8.5	1.8	0.185	0.030	0.50
PMC 140 FTSASK 1771.65m	0.1	0.9	0.1	0.6	0.5	< 0.001	0.35	11	7	9.1	1.7	0.201	0.017	0.45
PMC 140 FTSASK 1772.2m	0.1	1.0	0.1	0.7	0.6	< 0.001	0.29	4	7	9.6	1.9	0.208	0.032	0.39
PMC 140 FTSASK 1773.4m	0.1	0.7	0.1	< 0.1	< 0.1	< 0.001	0.23	5	6	7.1	1.8	0.126	0.019	0.13
PMC 140 FTSASK 1774.05m	0.1	0.8	0.1	< 0.1	0.1	< 0.001	0.24	5	6	7.7	1.7	0.147	0.015	0.23
PMC 140 FTSASK 1774.67m	< 0.1	0.3	< 0.1	0.2	0.2	< 0.001	0.08	4	2	2.0	0.8	0.0416	0.009	0.12
PMC 140 FTSASK 1775m	< 0.1	0.3	< 0.1	0.2	0.2	< 0.001	0.08	5	2	2.0	0.8	0.0403	0.009	0.10
PMC 140 FTSASK 1775.9m	< 0.1	0.6	< 0.1	< 0.1	< 0.1	< 0.001	0.23	7	4	4.6	1.4	0.0793	0.025	0.15
PMC 140 FTSASK 1776.9m	< 0.1	0.4	< 0.1	0.1	0.2	< 0.001	0.08	< 3	2	2.6	0.8	0.0519	0.018	0.07
PMC 140 FTSASK 1777.7m	< 0.1	0.6	< 0.1	0.1	0.2	< 0.001	0.12	5	3	4.3	1.1	0.0874	0.009	0.05
PMC 140 FTSASK 1778.4m	0.1	0.8	0.1	< 0.1	0.1	< 0.001	0.19	4	6	7.5	1.7	0.133	0.017	0.03
PMC 140 FTSASK 1779.3m	0.2	0.9	0.1	< 0.1	< 0.1	< 0.001	0.31	7	8	11.9	2.4	0.0997	0.026	0.05
PMC 140 FTSASK 1780.2m	0.1	0.7	0.1	< 0.1	< 0.1	< 0.001	0.20	5	5	7.2	1.4	0.118	0.015	0.02
PMC 140 FTSASK 1781.25m	0.1	0.7	0.1	< 0.1	< 0.1	< 0.001	0.17	4	5	6.5	1.3	0.104	0.017	0.03
PMC 140 FTSASK 1782.45m	0.1	0.7	< 0.1	< 0.1	< 0.1	< 0.001	0.16	3	4	6.1	1.2	0.104	0.016	0.04
PMC 140 FTSASK 1783.4m	< 0.1	0.6	0.1	< 0.1	0.2	< 0.001	0.15	< 3	4	5.8	1.4	0.0949	0.017	0.03
PMC 140 FTSASK 1784.4m	< 0.1	0.3	< 0.1	0.1	0.2	< 0.001	0.06	< 3	1	2.1	1.2	0.0364	0.013	0.04

Analyte Symbol	Tm	Yb	Lu	Ta	W	Re	Tl	Pb	Sc	Th	U	Ti	P	S
Unit Symbol	ppm	ppm	ppm	ppm	ppm	ppm	ppm	ppm	ppm	ppm	ppm	%	%	%
Lower Limit	0.1	0.1	0.1	0.1	0.1	0.001	0.05	3	1	0.1	0.1	0.0005	0.001	0.01
Method Code	TD-MS	TD-MS	TD-MS	TD-MS	TD-MS	TD-MS	TD-MS	TD-ICP	TD-ICP	TD-MS	TD-MS	TD-ICP	TD-ICP	TD-ICP
PMC 140 FTSASK 1785.15m	< 0.1	0.5	< 0.1	0.1	0.1	< 0.001	0.13	4	3	4.6	1.5	0.0685	0.013	0.03
PMC 140 FTSASK 1785.85m	< 0.1	0.4	< 0.1	0.3	0.2	< 0.001	0.09	9	2	3.0	1.1	0.0530	0.010	0.03
PMC 140 FTSASK 1786.8m	< 0.1	0.3	< 0.1	< 0.1	0.3	< 0.001	0.12	6	2	2.5	1.5	0.0416	0.011	0.03
PMC 140 FTSASK 1787.7m	< 0.1	0.3	< 0.1	0.3	0.3	< 0.001	0.07	5	1	2.6	1.3	0.0351	0.009	0.02
PMC 140 FTSASK 1788.5m	< 0.1	0.3	< 0.1	< 0.1	0.3	< 0.001	0.09	5	1	2.4	1.3	0.0419	0.011	0.02
PMC 140 FTSASK 1789.45m	< 0.1	0.4	< 0.1	0.2	0.4	< 0.001	0.20	10	2	3.0	1.2	0.0495	0.009	0.02
PMC 140 FTSASK 1790.45m	< 0.1	0.6	< 0.1	0.3	0.3	< 0.001	0.13	3	2	4.4	1.2	0.0669	0.011	0.02
PMC 140 FTSASK 1791.4m	< 0.1	0.3	< 0.1	< 0.1	0.2	< 0.001	0.11	5	1	2.0	1.0	0.0332	0.039	0.02
PMC 140 FTSASK 1792.4m	< 0.1	0.2	< 0.1	< 0.1	0.1	< 0.001	0.08	5	< 1	0.9	0.6	0.0170	0.006	0.11
PMC 140 FTSASK 1793.1m	0.1	1.1	0.2	< 0.1	0.1	< 0.001	0.23	5	6	8.4	2.3	0.139	0.055	0.01
PMC 140 FTSASK 1793.7m	< 0.1	< 0.1	< 0.1	< 0.1	< 0.1	< 0.001	< 0.05	3	< 1	0.2	0.4	0.0038	0.007	0.70
PMC 140 FTSASK 1794.55m	< 0.1	0.1	< 0.1	0.1	0.1	< 0.001	< 0.05	< 3	< 1	0.9	0.6	0.0177	0.036	0.04
PMC 140 FTSASK 1795.25m	< 0.1	0.2	< 0.1	0.1	0.3	< 0.001	< 0.05	3	< 1	1.5	0.7	0.0250	0.012	0.22
PMC 140 FTSASK 1796.08m	< 0.1	0.2	< 0.1	< 0.1	0.2	< 0.001	0.05	4	< 1	1.3	0.8	0.0246	0.058	0.04
PMC 140 FTSASK 1796.8m	< 0.1	0.2	< 0.1	< 0.1	0.3	< 0.001	< 0.05	5	< 1	1.3	0.9	0.0262	0.042	0.02
PMC 140 FTSASK 1797.4m	< 0.1	0.2	< 0.1	< 0.1	0.1	< 0.001	< 0.05	4	< 1	1.3	0.8	0.0222	0.109	0.07
PMC 140 FTSASK 1798.2m	< 0.1	0.3	< 0.1	< 0.1	0.3	< 0.001	0.10	4	2	2.6	2.4	0.0475	0.027	0.02
PMC 140 FTSASK 1799m	< 0.1	0.1	< 0.1	< 0.1	0.2	< 0.001	< 0.05	4	< 1	1.0	0.5	0.0198	0.011	0.03
PMC 140 FTSASK 1799.7m	< 0.1	0.1	< 0.1	< 0.1	0.1	< 0.001	< 0.05	4	< 1	0.6	0.5	0.0115	0.040	0.06
PMC 140 FTSASK 1800.6m	< 0.1	0.7	0.1	< 0.1	< 0.1	< 0.001	0.17	5	3	5.6	1.5	0.0670	0.132	0.02
PMC 140 FTSASK 1801.45m	< 0.1	0.1	< 0.1	< 0.1	0.1	< 0.001	< 0.05	4	< 1	0.8	0.5	0.0149	0.014	0.02

Analyte Symbol	Tm	Yb	Lu	Ta	W	Re	Tl	Pb	Sc	Th	U	Ti	P	S
Unit Symbol	ppm	ppm	ppm	ppm	ppm	ppm	ppm	ppm	ppm	ppm	ppm	%	%	%
Lower Limit	0.1	0.1	0.1	0.1	0.1	0.001	0.05	3	1	0.1	0.1	0.0005	0.001	0.01
Method Code	TD-MS	TD-MS	TD-MS	TD-MS	TD-MS	TD-MS	TD-MS	TD-ICP	TD-ICP	TD-MS	TD-MS	TD-ICP	TD-ICP	TD-ICP
PMC 140 FTSASK 1802.15m	< 0.1	0.2	< 0.1	< 0.1	0.1	< 0.001	< 0.05	4	< 1	1.8	0.6	0.0255	0.013	0.03
PMC 140 FTSASK 1803m	< 0.1	0.2	< 0.1	< 0.1	0.1	< 0.001	< 0.05	6	< 1	1.4	0.5	0.0223	0.018	0.02
PMC 140 FTSASK 1803.8m	< 0.1	0.1	< 0.1	0.1	0.1	< 0.001	< 0.05	3	< 1	0.9	0.4	0.0161	0.012	0.32
PMC 140 FTSASK 1804.5m	< 0.1	0.1	< 0.1	< 0.1	< 0.1	< 0.001	< 0.05	5	< 1	0.7	0.3	0.0124	0.009	0.03
PMC 140 FTSASK 1805.25m	0.1	0.8	0.1	0.6	0.5	< 0.001	0.21	< 3	5	8.8	2.2	0.149	0.071	0.03
PMC 140 FTSASK 1806.15m	< 0.1	0.5	< 0.1	< 0.1	0.2	< 0.001	0.10	5	2	3.1	0.9	0.0577	0.018	0.02
PMC 140 FTSASK 1806.6m	< 0.1	0.3	< 0.1	< 0.1	0.1	< 0.001	0.05	4	< 1	1.5	0.9	0.0261	0.005	0.03
PMC 140 FTSASK 1807.25m	< 0.1	0.4	< 0.1	< 0.1	0.2	< 0.001	0.07	4	1	2.6	0.8	0.0472	0.008	0.02
PMC 140 FTSASK 1808m	0.1	0.7	0.1	0.2	0.1	< 0.001	0.15	4	3	6.1	1.6	0.0877	0.029	0.02
PMC 140 FTSASK 1808.8m	0.1	0.9	0.1	0.5	0.4	< 0.001	0.18	< 3	4	8.9	2.1	0.126	0.034	0.02
PMC 140 FTSASK 1809.6m	0.1	0.9	0.1	0.1	< 0.1	< 0.001	0.18	5	4	8.2	1.8	0.107	0.031	0.02
PMC 140 FTSASK 1810.6m	0.1	0.7	0.1	< 0.1	0.2	< 0.001	0.17	4	4	6.4	1.5	0.109	0.034	0.03

Analyte Symbol	Li	Na	Mg	Al	K	Ca	Cd	V	Cr	Mn	Fe	Hf	Ni	Er	Be	Ho	Ag	Cs	Co	Eu	Bi	Se	Zn
Unit Symbol	ppm	%	%	%	%	%	ppm	ppm	ppm	ppm	%	ppm	ppm	ppm	ppm	ppm	ppm	ppm	ppm	ppm	ppm	ppm	ppm
Lower Limit	1	0.01	0.01	0.01	0.01	0.01	0.3	1	1	1	0.01	0.1	1	0.1	0.1	0.1	0.3	0.05	1	0.05	0.02	0.1	1
Method Code	TD-ICP	TD-MS	TD-MS	TD-ICP	TD-MS	TD-MS	TD-ICP	TD-MS	TD-MS	TD-MS	TD-MS	TD-MS	TD-ICP	TD-MS	TD-MS	TD-MS	TD-ICP	TD-MS	TD-ICP	TD-MS	TD-MS	TD-MS	TD-ICP
OREAS 101b (4 Acid) Meas			1.34		2.46			73		903	10.0		9	13.8		4.9			44	7.59			
OREAS 101b (4 Acid) Cert			1.23		2.36			77		927	10.7		8.2	15		5.2			45	8.1			
OREAS 903 (4 Acid) Meas	18	0.03	0.77	5.66	1.72	0.60	0.4	75	64	635	4.12	0.3	54		4.9		< 0.3	3.54	138		8.90	6.1	27
OREAS 903 (4 Acid) Cert	18.3	0.0300	0.714	5.89	3.31	0.625	0.200	74.0	73.0	690	4.16	4.56	54.0		4.42		0.432	3.57	131		8.90	6.06	24.3
OREAS 520 (4 Acid) Meas	18			5.33									75				0.6		188				26
OREAS 520 (4 Acid) Cert	16.9			5.63									76.0				0.450		203				22.7
Oreas 72b (4 Acid) Meas	31	0.95	10.00	4.44	1.03	2.64	0.4	74	856	911	6.58	2.6	6480		0.9		< 0.3	3.24	125		0.72		94
Oreas 72b (4 Acid) Cert	33.3	1.01	9.59	4.79	1.14	2.79	0.310	73.6	771	1010	6.84	2.51	6860		1.02		0.230	3.37	131		0.680		99.0
OREAS 683 (4 Acid) Meas	7	0.98	8.97	6.77	0.50	5.04	< 0.3	100	> 5000	1210	7.42	0.9	1120	0.8	0.5	0.3	< 0.3	1.45	79	0.61	0.15		86
OREAS 683 (4 Acid) Cert	6.51	1.03	8.63	7.15	0.507	5.23	0.072	187	7710	1200	7.32	0.75	1180	0.93	0.56	0.32	0.172	1.32	85	0.58	0.16		92
OREAS 247 (4 Acid) Meas		0.46	1.31		1.36	0.78		63	75	350	3.34	3.5		1.8	2.3	0.6		8.34		0.99	0.59		
OREAS 247 (4 Acid) Cert		0.499	1.22		2.45	0.826		82.0	97.0	360	3.32	3.57		1.49	2.23	0.540		8.49		0.960	0.580		
OREAS 601c (4 acid) Meas	28			6.67			2.3						8				50.6		5				441
OREAS 601c (4 acid) Cert	26.7			7.06			2.77						6.83				50.3		4.99				425
PMC 140 FTSASK 1769.04m Orig	16	0.42	11.3	1.22	1.21	17.7	< 0.3	20	16	180	0.74	1.0	15	0.5	0.3	0.2	< 0.3	0.87	2	0.28	0.08	0.8	10
PMC 140 FTSASK 1769.04m Dup	16	0.43	11.3	1.20	1.24	17.9	< 0.3	20	16	175	0.75	1.0	16	0.5	0.3	0.2	< 0.3	0.78	2	0.30	0.09	0.8	10
PMC 140 FTSASK 1775m Orig	23	0.16	11.8	1.10	1.07	17.6	< 0.3	15	14	212	0.48	0.3	5	0.3	0.4	0.1	< 0.3	0.68	2	0.26	0.04	0.2	14
PMC 140 FTSASK 1775m Dup	24	0.16	11.9	1.09	1.03	18.2	< 0.3	15	9	213	0.47	0.3	5	0.4	0.4	0.1	< 0.3	0.68	2	0.27	0.04	0.4	15
PMC 140 FTSASK 1787.7m Orig	19	0.11	12.4	0.81	0.85	18.3	< 0.3	16	10	275	0.39	0.4	9	0.4	0.4	0.1	< 0.3	0.47	2	0.24	0.05	0.2	9
PMC 140 FTSASK 1787.7m Dup	19	0.11	12.5	0.81	0.85	18.3	< 0.3	16	8	279	0.38	0.4	9	0.3	0.4	0.1	< 0.3	0.51	2	0.23	0.05	0.5	10
PMC 140 FTSASK 1797.4m Orig	10	0.14	13.1	0.49	0.54	20.3	< 0.3	10	6	219	0.28	< 0.1	4	0.2	0.2	< 0.1	< 0.3	0.27	1	0.14	0.02	1.1	6
PMC 140 FTSASK 1797.4m Dup	10	0.13	12.7	0.49	0.51	19.7	< 0.3	10	4	220	0.26	< 0.1	4	0.2	0.2	< 0.1	< 0.3	0.25	< 1	0.15	< 0.02	0.6	6
PMC 140 FTSASK 1810.6m Orig	46	0.20	10.9	2.29	1.64	14.0	< 0.3	19	15	226	1.36	1.4	13	0.8	0.7	0.3	< 0.3	1.31	5	0.48	< 0.02	< 0.1	16
PMC 140 FTSASK 1810.6m Dup	46	0.20	10.7	2.28	1.43	13.9	< 0.3	24	16	244	1.36	0.4	13	0.8	0.8	0.3	< 0.3	1.32	4	0.48	0.02	< 0.1	16
Method Blank	< 1			< 0.01			< 0.3						< 1				< 0.3		< 1				< 1
Method Blank	< 1			< 0.01			< 0.3						< 1				< 0.3		< 1				< 1

Analyte Symbol	Li	Na	Mg	Al	K	Ca	Cd	V	Cr	Mn	Fe	Hf	Ni	Er	Be	Ho	Ag	Cs	Co	Eu	Bi	Se	Zn
Unit Symbol	ppm	%	%	%	%	%	ppm	ppm	ppm	ppm	%	ppm	ppm	ppm	ppm	ppm	ppm	ppm	ppm	ppm	ppm	ppm	ppm
Lower Limit	1	0.01	0.01	0.01	0.01	0.01	0.3	1	1	1	0.01	0.1	1	0.1	0.1	0.1	0.3	0.05	1	0.05	0.02	0.1	1
Method Code	TD-ICP	TD-MS	TD-MS	TD-ICP	TD-MS	TD-MS	TD-ICP	TD-MS	TD-MS	TD-MS	TD-MS	TD-MS	TD-ICP	TD-MS	TD-MS	TD-MS	TD-ICP	TD-MS	TD-ICP	TD-MS	TD-MS	TD-MS	TD-ICP
Method Blank		< 0.01	< 0.01		< 0.01	< 0.01		2			< 0.01	< 0.1		< 0.1	< 0.1	< 0.1		< 0.05		< 0.05	< 0.02	< 0.1	
Method Blank		< 0.01	< 0.01		< 0.01	< 0.01		1			< 0.01	< 0.1		< 0.1	< 0.1	< 0.1		< 0.05		< 0.05	< 0.02	< 0.1	
Method Blank		< 0.01	< 0.01		< 0.01	< 0.01		1	2	4	< 0.01	< 0.1		< 0.1	< 0.1	< 0.1		< 0.05		< 0.05	< 0.02	0.2	
Method Blank																							

Analyte Symbol	Ga	As	Rb	Y	Sr	Zr	Nb	Mo	In	Sn	Sb	Te	Ba	La	Ce	Pr	Nd	Sm	Gd	Tb	Dy	Cu	Ge
Unit Symbol	ppm	ppm	ppm	ppm	ppm	ppm	ppm	ppm	ppm	ppm	ppm	ppm	ppm	ppm	ppm	ppm	ppm	ppm	ppm	ppm	ppm	ppm	ppm
Lower Limit	0.1	0.1	0.2	0.1	0.2	1	0.1	1	0.1	1	0.1	0.1	1	0.1	0.1	0.1	0.1	0.1	0.1	0.1	0.1	1	0.1
Method Code	TD-MS	TD-MS	TD-MS	TD-MS	TD-MS	TD-MS	TD-MS	TD-ICP	TD-MS	TD-MS	TD-MS	TD-MS	TD-MS	TD-MS	TD-MS	TD-MS	TD-MS	TD-MS	TD-MS	TD-MS	TD-MS	TD-ICP	TD-MS
OREAS 101b (4 Acid) Meas				131				18						855	1450	123	388	48.7	38.0	4.5	25.1	411	
OREAS 101b (4 Acid) Cert				133				20.1						754	1325	127	388	48	40	5.4	27	412	
OREAS 903 (4 Acid) Meas	15.4	50.1	118	21.3	76.2	25		5	0.2	2	1.7		159	41.9	80.7					0.8		6440	
OREAS 903 (4 Acid) Cert	15.0	49.7	137	22.5	77.0	152		4.32	0.160	2.63	1.57		197	40.0	82.0					0.830		6520	
OREAS 520 (4 Acid) Meas								54														2930	
OREAS 520 (4 Acid) Cert								65.0														2930	
Oreas 72b (4 Acid) Meas	10.2	151	50.2	12.4	62.1	86	5.7	4	< 0.1	1	0.8	< 0.1	166	24.3	43.0					0.4		216	
Oreas 72b (4 Acid) Cert	11.7	146	50.8	12.8	63.8	88.0	5.50	4.01	0.0490	1.43	0.870	0.0920	330	24.4	43.6					0.440		222	
OREAS 683 (4 Acid) Meas	13.0		30.7	7.8	282	29	2.9	3	< 0.1	< 1			202	8.5	17.3	2.1	8.5	2.0	1.6	0.3	1.5	404	
OREAS 683 (4 Acid) Cert	13.8		26.8	8.02	276	26	2.61	1.00	0.028	0.85			188	8.17	17.1	2.2	8.75	1.86	1.64	0.25	1.54	404	
OREAS 247 (4 Acid) Meas	16.0	3070	118	16.2	102	116	0.9		< 0.1	2	403		465	33.7	66.3	7.8	29.9	4.6	4.3	0.6	3.5		
OREAS 247 (4 Acid) Cert	16.3	3510	144	13.1	96.0	125	11.7		0.0580	3.31	3300		550	33.1	67.0	7.90	29.3	5.50	42.3	0.530	2.73		
OREAS 601c (4 acid) Meas								4														1160	
OREAS 601c (4 acid) Cert								3.66														1160.000	
PMC 140 FTSASK 1769.04m Orig	2.6	17.8	31.3	4.6	137	39	3.9	17	< 0.1	< 1	1.4	< 0.1	100	7.4	14.5	1.7	6.9	1.0	1.0	0.1	0.9	16	< 0.1
PMC 140 FTSASK 1769.04m Dup	2.6	17.3	32.8	4.5	140	36	3.4	16	< 0.1	< 1	1.4	< 0.1	101	7.7	15.1	1.8	7.2	1.3	1.1	0.2	0.9	16	< 0.1
PMC 140 FTSASK 1775m Orig	2.7	1.7	27.0	3.6	126	13	3.3	< 1	< 0.1	< 1	< 0.1	< 0.1	51	7.5	15.4	1.9	6.9	1.0	1.0	0.1	0.7	5	< 0.1
PMC 140 FTSASK 1775m Dup	2.7	0.9	26.1	3.8	135	13	3.1	< 1	< 0.1	< 1	< 0.1	< 0.1	50	7.7	15.8	1.9	6.9	1.5	1.0	0.1	0.6	7	< 0.1
PMC 140 FTSASK 1787.7m Orig	2.1	0.4	21.9	3.5	118	19	7.8	< 1	< 0.1	< 1	< 0.1	< 0.1	47	11.3	20.1	2.1	7.2	1.1	0.9	0.1	0.6	7	< 0.1
PMC 140 FTSASK 1787.7m Dup	2.1	0.4	21.6	3.5	120	21	6.9	< 1	< 0.1	< 1	< 0.1	< 0.1	47	11.1	19.8	2.1	7.5	1.0	0.9	0.1	0.7	8	0.1
PMC 140 FTSASK 1797.4m Orig	1.1	1.7	11.9	2.1	121	1	0.8	< 1	< 0.1	< 1	< 0.1	< 0.1	90	7.2	15.2	1.7	5.9	0.8	0.6	< 0.1	0.4	4	0.1
PMC 140 FTSASK 1797.4m Dup	1.3	0.7	11.4	2.0	113	1	0.8	< 1	< 0.1	< 1	< 0.1	< 0.1	79	6.9	14.9	1.6	5.5	0.5	0.7	< 0.1	0.3	4	0.2
PMC 140 FTSASK 1810.6m Orig	4.6	1.5	64.6	7.5	134	54	3.6	< 1	< 0.1	< 1	0.1	< 0.1	215	21.6	34.9	4.0	13.2	2.7	1.9	0.2	1.4	3	< 0.1
PMC 140 FTSASK 1810.6m Dup	4.7	2.6	64.0	7.7	140	24	5.3	< 1	< 0.1	< 1	< 0.1	< 0.1	209	21.2	35.0	4.0	13.6	2.5	1.9	0.3	1.4	3	< 0.1
Method Blank								< 1														< 1	
Method Blank								< 1														< 1	

Analyte Symbol	Ga	As	Rb	Y	Sr	Zr	Nb	Mo	In	Sn	Sb	Te	Ba	La	Ce	Pr	Nd	Sm	Gd	Tb	Dy	Cu	Ge
Unit Symbol	ppm	ppm	ppm	ppm	ppm	ppm	ppm	ppm	ppm	ppm	ppm	ppm	ppm	ppm	ppm	ppm	ppm	ppm	ppm	ppm	ppm	ppm	ppm
Lower Limit	0.1	0.1	0.2	0.1	0.2	1	0.1	1	0.1	1	0.1	0.1	1	0.1	0.1	0.1	0.1	0.1	0.1	0.1	0.1	1	0.1
Method Code	TD-MS	TD-MS	TD-MS	TD-MS	TD-MS	TD-MS	TD-MS	TD-ICP	TD-MS	TD-MS	TD-MS	TD-MS	TD-MS	TD-MS	TD-MS	TD-MS	TD-MS	TD-MS	TD-MS	TD-MS	TD-MS	TD-ICP	TD-MS
Method Blank	0.1	0.2	< 0.2	< 0.1	< 0.2	< 1	< 0.1		< 0.1	< 1	< 0.1	< 0.1	< 1	< 0.1	< 0.1	< 0.1	< 0.1	< 0.1	< 0.1	< 0.1	< 0.1		< 0.1
Method Blank	0.1	0.7	< 0.2	< 0.1	< 0.2	< 1	< 0.1		< 0.1	< 1	< 0.1	< 0.1	< 1	< 0.1	< 0.1	< 0.1	< 0.1	< 0.1	< 0.1	< 0.1	< 0.1		< 0.1
Method Blank	0.1	< 0.1	< 0.2	< 0.1	< 0.2	< 1	< 0.1		< 0.1	< 1	< 0.1	< 0.1	< 1	< 0.1	< 0.1	< 0.1	< 0.1	< 0.1	< 0.1	< 0.1	< 0.1		< 0.1
Method Blank																						< 1	

Analyte Symbol	Tm	Yb	Lu	Ta	W	Re	Tl	Pb	Sc	Th	U	Ti	P	S
Unit Symbol	ppm	ppm	ppm	ppm	ppm	ppm	ppm	ppm	ppm	ppm	ppm	%	%	%
Lower Limit	0.1	0.1	0.1	0.1	0.1	0.001	0.05	3	1	0.1	0.1	0.0005	0.001	0.01
Method Code	TD-MS	TD-MS	TD-MS	TD-MS	TD-MS	TD-MS	TD-MS	TD-ICP	TD-ICP	TD-MS	TD-MS	TD-ICP	TD-ICP	TD-ICP
OREAS 101b (4 Acid) Meas	2.0	12.4	1.8					21		39.6	417	0.317	0.105	
OREAS 101b (4 Acid) Cert	2.08	13.9	1.96					23		36.4	387	0.35	0.1118	
OREAS 903 (4 Acid) Meas		2.1	0.3	< 0.1			0.63	9	11	14.6	7.6	0.313	0.099	0.47
OREAS 903 (4 Acid) Cert		2.36	0.360	0.540			0.620	11.3	10.2	13.6	7.58	0.192	0.107	0.500
OREAS 520 (4 Acid) Meas								18	18			0.364	0.069	0.92
OREAS 520 (4 Acid) Cert								5.85	17.0			0.445	0.0740	1.01
Oreas 72b (4 Acid) Meas				0.4	4.9		0.33	16	13	11.8	4.7	0.206	0.024	1.38
Oreas 72b (4 Acid) Cert				0.430	4.00		0.350	14.9	12.8	11.3	4.68	0.216	0.0260	1.49
OREAS 683 (4 Acid) Meas	0.1	0.9	0.1		1.3			11	21	2.6	0.6	0.259	0.047	0.19
OREAS 683 (4 Acid) Cert	0.13	0.88	0.13		1.23			10.2	19.7	2.42	0.58	0.263	0.050	0.205
OREAS 247 (4 Acid) Meas	0.3	1.9	0.3	< 0.1	0.3		0.78			13.8	2.6			
OREAS 247 (4 Acid) Cert	0.230	1.54	0.240	0.920	7.88		0.800			12.6	2.53			
OREAS 601c (4 acid) Meas								321	4			0.146	0.038	1.54
OREAS 601c (4 acid) Cert								328	4.01			0.135	0.039	1.58
PMC 140 FTSASK 1769.04m Orig	< 0.1	0.4	< 0.1	0.2	0.3	0.019	0.57	15	2	3.1	3.1	0.0633	0.009	0.48
PMC 140 FTSASK 1769.04m Dup	< 0.1	0.4	< 0.1	0.2	0.4	0.019	0.59	15	2	3.1	3.1	0.0639	0.009	0.48
PMC 140 FTSASK 1775m Orig	< 0.1	0.3	< 0.1	0.2	0.2	< 0.001	0.08	5	2	2.0	0.8	0.0403	0.008	0.10
PMC 140 FTSASK 1775m Dup	< 0.1	0.3	< 0.1	0.1	0.2	< 0.001	0.08	5	2	2.0	0.9	0.0404	0.009	0.10
PMC 140 FTSASK 1787.7m Orig	< 0.1	0.3	< 0.1	0.3	0.3	< 0.001	0.07	5	1	2.6	1.3	0.0350	0.009	0.02
PMC 140 FTSASK 1787.7m Dup	< 0.1	0.3	< 0.1	0.2	0.3	< 0.001	0.07	5	1	2.6	1.3	0.0351	0.009	0.02
PMC 140 FTSASK 1797.4m Orig	< 0.1	0.2	< 0.1	< 0.1	0.1	< 0.001	0.05	4	< 1	1.3	0.8	0.0223	0.109	0.07
PMC 140 FTSASK 1797.4m Dup	< 0.1	0.2	< 0.1	< 0.1	0.2	< 0.001	< 0.05	4	< 1	1.3	0.8	0.0222	0.109	0.07
PMC 140 FTSASK 1810.6m Orig	0.1	0.7	0.1	< 0.1	0.2	< 0.001	0.17	4	4	6.4	1.5	0.102	0.035	0.03
PMC 140 FTSASK 1810.6m Dup	0.1	0.7	0.1	0.1	0.2	< 0.001	0.16	5	4	6.5	1.5	0.116	0.034	0.03
Method Blank								< 3	< 1			< 0.0005	< 0.001	< 0.01

Analyte Symbol	Tm	Yb	Lu	Ta	W	Re	Tl	Pb	Sc	Th	U	Ti	P	S
Unit Symbol	ppm	ppm	ppm	ppm	ppm	ppm	ppm	ppm	ppm	ppm	ppm	%	%	%
Lower Limit	0.1	0.1	0.1	0.1	0.1	0.001	0.05	3	1	0.1	0.1	0.0005	0.001	0.01
Method Code	TD-MS	TD-MS	TD-MS	TD-MS	TD-MS	TD-MS	TD-MS	TD-ICP	TD-ICP	TD-MS	TD-MS	TD-ICP	TD-ICP	TD-ICP
Method Blank								< 3	< 1			< 0.0005	< 0.001	< 0.01
Method Blank	< 0.1	< 0.1	< 0.1	< 0.1	< 0.1	< 0.001	< 0.05			< 0.1	< 0.1			
Method Blank	< 0.1	< 0.1	< 0.1	< 0.1	< 0.1	< 0.001	< 0.05			< 0.1	< 0.1			
Method Blank	< 0.1	< 0.1	< 0.1	< 0.1	< 0.1	< 0.001	< 0.05			< 0.1	< 0.1			
Method Blank														
