# Supplementary material for: Geochemistry and X-ray diffraction data from rock salts and saltwork wastes of Canada: data compilation
Source: Data Brief. 2026 Jun 6;67:112941. doi: 10.1016/j.dib.2026.112941 (PMC13292661; doi:10.1016/j.dib.2026.112941)
Supplement: Supplementary file 13 [file mmc13.pdf]

**Report No.:**	**A25-18721**
**Report Date:**	**13-Jan-26**
**Date Submitted:**	**18-Dec-25**
**Your Reference:**	**CMGD**

Geological Survey of Canada-AB
3303 33rd St NW
Calgary Alberta T2-2A7
Canada

ATTN: Pavel Kabanov

CERTIFICATE OF ANALYSIS

16 Pulp samples were submitted for analysis.

The following analytical package(s) were requested:		Testing Date:
UT-6	QOP Total/QOP Ultratrace- 4acid Digest (Total Digestion ICPOES/ICPMS)	2025-12-29 16:45:38

REPORT **A25-18721**

This report may be reproduced without our consent. If only selected portions of the report are reproduced, permission must be obtained. If no instructions were given at time of sample submittal regarding excess material, it will be discarded within 90 days of this report. Our liability is limited solely to the analytical cost of these analyses. Test results are representative only of material submitted for analysis.

Notes:

Values which exceed the upper limit should be assayed for accurate numbers.

Refer to the Scope of Accreditation for information on accredited elements.

SCC Accredited LAB / LAB Accrédité CCN ™

CERTIFIED BY:

Elitsa Hrischeva, Ph.D.
Quality Control Coordinator

ACTIVATION LABORATORIES LTD.
41 Bittern Street, Ancaster, Ontario, Canada, L9G 4V5
TELEPHONE +905 648-9611 or +1.888.228.5227 FAX +1.905.648.9613
E-MAIL Ancaster@actlabs.com ACTLABS GROUP WEBSITE www.actlabs.com

Results **Activation Laboratories** **Report: A25-18721**

Analyte Symbol	Li	Na	Mg	Al	K	Ca	Cd	V	Cr	Mn	Fe	Hf	Ni	Er	Be	Ho	Ag	Cs	Co	Eu	Bi	Se	Zn
Unit Symbol	ppm	%	%	%	%	%	ppm	ppm	ppm	ppm	%	ppm	ppm	ppm	ppm	ppm	ppm	ppm	ppm	ppm	ppm	ppm	ppm
Lower Limit	1	0.01	0.01	0.01	0.01	0.01	0.3	1	1	1	0.01	0.1	1	0.1	0.1	0.1	0.3	0.05	1	0.05	0.02	0.1	1
Method Code	TD-ICP	TD-MS	TD-MS	TD-ICP	TD-MS	TD-MS	TD-ICP	TD-MS	TD-MS	TD-MS	TD-MS	TD-MS	TD-ICP	TD-MS	TD-MS	TD-MS	TD-ICP	TD-MS	TD-ICP	TD-MS	TD-MS	TD-MS	TD-ICP
PF-1 877.2 m	1	> 3.00	< 0.01	0.02	0.01	4.10	< 0.3	1	1	3	0.01	< 0.1	< 1	< 0.1	< 0.1	< 0.1	< 0.3	< 0.05	< 1	< 0.05	< 0.02	< 0.1	< 1
PF-1 849.92 m	1	> 3.00	< 0.01	0.01	0.01	2.99	< 0.3	1	< 1	2	< 0.01	< 0.1	< 1	< 0.1	< 0.1	< 0.1	< 0.3	< 0.05	< 1	< 0.05	< 0.02	0.1	< 1
PF-1 824.4 m	< 1	> 3.00	< 0.01	< 0.01	0.01	1.79	< 0.3	< 1	< 1	2	< 0.01	< 0.1	< 1	< 0.1	< 0.1	< 0.1	< 0.3	< 0.05	< 1	< 0.05	< 0.02	< 0.1	< 1
PF-1 798.10 m	1	> 3.00	< 0.01	< 0.01	< 0.01	1.49	< 0.3	< 1	2	< 1	< 0.01	< 0.1	< 1	< 0.1	< 0.1	< 0.1	< 0.3	< 0.05	< 1	< 0.05	< 0.02	< 0.1	< 1
PF-1 751.96 m	1	> 3.00	0.05	0.01	0.02	6.71	< 0.3	< 1	< 1	3	< 0.01	< 0.1	< 1	< 0.1	< 0.1	< 0.1	< 0.3	< 0.05	< 1	< 0.05	< 0.02	< 0.1	< 1
LR98-1 381.45 m	3	> 3.00	0.10	0.14	0.06	0.20	< 0.3	2	3	13	0.05	< 0.1	< 1	< 0.1	0.1	< 0.1	< 0.3	0.15	< 1	< 0.05	< 0.02	0.2	2
LR98-1 390.22 m	6	> 3.00	0.30	0.39	0.11	0.49	< 0.3	5	5	36	0.21	0.1	2	0.1	0.2	< 0.1	< 0.3	0.32	2	0.12	0.03	< 0.1	11
LR98-1 668.90 m	4	> 3.00	0.19	0.17	0.12	2.93	< 0.3	3	2	10	0.08	< 0.1	< 1	< 0.1	0.1	< 0.1	< 0.3	0.14	1	< 0.05	0.03	< 0.1	5
LR98-1 642.1 m	2	> 3.00	0.12	0.11	3.54	0.44	< 0.3	2	2	14	0.05	< 0.1	< 1	< 0.1	< 0.1	< 0.1	< 0.3	0.08	< 1	< 0.05	< 0.02	< 0.1	2
LR98-1 639.91 m	< 1	> 3.00	0.02	0.01	0.06	0.30	< 0.3	< 1	1	3	< 0.01	< 0.1	< 1	< 0.1	< 0.1	< 0.1	< 0.3	< 0.05	< 1	< 0.05	< 0.02	0.2	< 1
LR98-1 617.90 m	< 1	> 3.00	< 0.01	< 0.01	0.05	0.39	< 0.3	< 1	< 1	1	< 0.01	< 0.1	< 1	< 0.1	< 0.1	< 0.1	< 0.3	< 0.05	< 1	< 0.05	< 0.02	< 0.1	< 1
LR98-1 675.97 m	1	> 3.00	< 0.01	0.03	0.04	2.65	< 0.3	< 1	1	4	0.01	< 0.1	< 1	< 0.1	< 0.1	< 0.1	< 0.3	< 0.05	< 1	< 0.05	< 0.02	< 0.1	< 1
LR98-1 682.75 m	2	> 3.00	0.10	0.02	0.05	2.88	< 0.3	< 1	1	4	0.02	< 0.1	< 1	< 0.1	< 0.1	< 0.1	< 0.3	< 0.05	< 1	< 0.05	< 0.02	< 0.1	< 1
LR98-1 601.62 m	1	> 3.00	0.01	0.02	0.04	0.16	< 0.3	< 1	1	2	< 0.01	< 0.1	< 1	< 0.1	< 0.1	< 0.1	< 0.3	< 0.05	< 1	< 0.05	0.02	< 0.1	< 1
LR98-1 512.08 m	3	> 3.00	0.08	0.12	0.05	0.57	< 0.3	2	2	9	0.06	< 0.1	< 1	0.6	< 0.1	< 0.1	< 0.3	0.12	< 1	< 0.05	0.02	< 0.1	2
LR98-1 505.20 m	12	> 3.00	0.78	0.85	0.30	1.36	< 0.3	12	9	73	0.52	0.4	8	0.4	0.3	0.1	< 0.3	0.72	3	0.19	< 0.02	0.2	9

Analyte Symbol	Ga	As	Rb	Y	Sr	Zr	Nb	Mo	In	Sn	Sb	Te	Ba	La	Ce	Pr	Nd	Sm	Gd	Tb	Dy	Cu	Ge
Unit Symbol	ppm	ppm	ppm	ppm	ppm	ppm	ppm	ppm	ppm	ppm	ppm	ppm	ppm	ppm	ppm	ppm	ppm	ppm	ppm	ppm	ppm	ppm	ppm
Lower Limit	0.1	0.1	0.2	0.1	0.2	1	0.1	1	0.1	1	0.1	0.1	1	0.1	0.1	0.1	0.1	0.1	0.1	0.1	0.1	1	0.1
Method Code	TD-MS	TD-MS	TD-MS	TD-MS	TD-MS	TD-MS	TD-MS	TD-ICP	TD-MS	TD-MS	TD-MS	TD-MS	TD-MS	TD-MS	TD-MS	TD-MS	TD-MS	TD-MS	TD-MS	TD-MS	TD-MS	TD-ICP	TD-MS
PF-1 877.2 m	< 0.1	< 0.1	0.6	< 0.1	197	2	< 0.1	< 1	< 0.1	< 1	0.5	< 0.1	2	0.1	0.3	< 0.1	0.1	< 0.1	< 0.1	< 0.1	< 0.1	1	< 0.1
PF-1 849.92 m	< 0.1	< 0.1	0.3	< 0.1	91.7	< 1	< 0.1	< 1	< 0.1	< 1	0.3	< 0.1	2	< 0.1	0.1	< 0.1	< 0.1	< 0.1	< 0.1	< 0.1	< 0.1	< 1	< 0.1
PF-1 824.4 m	< 0.1	< 0.1	< 0.2	< 0.1	57.7	< 1	< 0.1	< 1	< 0.1	< 1	0.3	< 0.1	< 1	< 0.1	0.1	< 0.1	< 0.1	< 0.1	< 0.1	< 0.1	< 0.1	< 1	< 0.1
PF-1 798.10 m	< 0.1	< 0.1	0.2	< 0.1	41.6	< 1	< 0.1	< 1	< 0.1	< 1	0.1	< 0.1	< 1	< 0.1	0.1	< 0.1	0.1	< 0.1	< 0.1	< 0.1	< 0.1	< 1	< 0.1
PF-1 751.96 m	< 0.1	< 0.1	0.4	< 0.1	168	< 1	< 0.1	< 1	< 0.1	< 1	0.4	< 0.1	< 1	0.2	0.4	< 0.1	0.1	< 0.1	< 0.1	< 0.1	< 0.1	< 1	< 0.1
LR98-1 381.45 m	0.4	0.2	2.3	0.4	13.7	1	0.2	< 1	< 0.1	< 1	0.5	< 0.1	6	0.7	1.2	0.1	0.6	0.1	< 0.1	< 0.1	< 0.1	2	< 0.1
LR98-1 390.22 m	0.9	1.1	5.1	1.2	33.4	5	0.6	< 1	< 0.1	< 1	0.6	< 0.1	20	1.7	3.7	0.4	1.5	0.3	0.3	< 0.1	0.3	7	< 0.1
LR98-1 668.90 m	0.4	0.3	2.5	0.6	83.4	3	0.3	< 1	< 0.1	< 1	0.5	< 0.1	8	0.8	1.6	0.2	0.7	0.1	0.2	< 0.1	0.1	< 1	< 0.1
LR98-1 642.1 m	0.3	0.3	8.2	0.3	14.5	2	0.2	< 1	< 0.1	< 1	0.5	< 0.1	4	0.4	1.0	0.1	0.5	< 0.1	< 0.1	< 0.1	< 0.1	< 1	< 0.1
LR98-1 639.91 m	< 0.1	< 0.1	0.2	< 0.1	7.3	< 1	< 0.1	< 1	< 0.1	< 1	0.6	< 0.1	< 1	< 0.1	0.2	< 0.1	< 0.1	< 0.1	< 0.1	< 0.1	< 0.1	< 1	< 0.1
LR98-1 617.90 m	< 0.1	< 0.1	0.2	< 0.1	6.5	< 1	< 0.1	< 1	< 0.1	< 1	0.5	< 0.1	< 1	< 0.1	< 0.1	< 0.1	< 0.1	< 0.1	< 0.1	< 0.1	< 0.1	1	< 0.1
LR98-1 675.97 m	< 0.1	< 0.1	0.7	0.1	111	< 1	< 0.1	< 1	< 0.1	< 1	0.5	< 0.1	2	0.2	0.3	< 0.1	0.1	< 0.1	< 0.1	< 0.1	< 0.1	< 1	< 0.1
LR98-1 682.75 m	< 0.1	< 0.1	0.6	0.1	116	< 1	< 0.1	< 1	< 0.1	< 1	0.2	< 0.1	2	0.1	0.3	< 0.1	0.2	< 0.1	< 0.1	< 0.1	< 0.1	< 1	< 0.1
LR98-1 601.62 m	< 0.1	0.1	0.2	< 0.1	3.4	< 1	< 0.1	< 1	< 0.1	< 1	0.6	< 0.1	1	< 0.1	0.2	< 0.1	0.1	< 0.1	< 0.1	< 0.1	< 0.1	< 1	< 0.1
LR98-1 512.08 m	0.3	0.1	1.5	0.8	27.5	4	0.4	< 1	< 0.1	< 1	0.6	< 0.1	5	0.8	1.6	0.2	0.9	0.2	0.1	< 0.1	0.1	1	< 0.1
LR98-1 505.20 m	2.1	1.8	12.7	4.0	68.3	17	1.7	< 1	< 0.1	< 1	0.5	< 0.1	37	4.5	9.9	1.2	4.5	1.0	0.8	0.1	0.9	8	< 0.1

Analyte Symbol	Tm	Yb	Lu	Ta	W	Re	Tl	Pb	Sc	Th	U	Ti	P	S
Unit Symbol	ppm	ppm	ppm	ppm	ppm	ppm	ppm	ppm	ppm	ppm	ppm	%	%	%
Lower Limit	0.1	0.1	0.1	0.1	0.1	0.001	0.05	3	1	0.1	0.1	0.0005	0.001	0.01
Method Code	TD-MS	TD-MS	TD-MS	TD-MS	TD-MS	TD-MS	TD-MS	TD-ICP	TD-ICP	TD-MS	TD-MS	TD-ICP	TD-ICP	TD-ICP
PF-1 877.2 m	< 0.1	< 0.1	< 0.1	< 0.1	< 0.1	< 0.001	< 0.05	< 3	< 1	0.1	< 0.1	0.0012	< 0.001	2.79
PF-1 849.92 m	< 0.1	< 0.1	< 0.1	< 0.1	< 0.1	< 0.001	< 0.05	< 3	< 1	< 0.1	< 0.1	0.0007	< 0.001	2.17
PF-1 824.4 m	< 0.1	< 0.1	< 0.1	< 0.1	< 0.1	< 0.001	< 0.05	< 3	< 1	< 0.1	< 0.1	< 0.0005	< 0.001	1.28
PF-1 798.10 m	< 0.1	< 0.1	< 0.1	< 0.1	< 0.1	< 0.001	< 0.05	< 3	< 1	< 0.1	< 0.1	< 0.0005	< 0.001	1.18
PF-1 751.96 m	< 0.1	< 0.1	< 0.1	< 0.1	0.7	< 0.001	< 0.05	< 3	< 1	< 0.1	< 0.1	0.0005	< 0.001	5.44
LR98-1 381.45 m	< 0.1	< 0.1	< 0.1	< 0.1	0.1	< 0.001	< 0.05	< 3	< 1	0.1	0.1	0.0054	0.001	0.11
LR98-1 390.22 m	< 0.1	< 0.1	< 0.1	< 0.1	0.2	< 0.001	< 0.05	< 3	< 1	0.3	0.2	0.0162	0.003	0.26
LR98-1 668.90 m	< 0.1	< 0.1	< 0.1	< 0.1	0.1	< 0.001	< 0.05	< 3	< 1	0.2	< 0.1	0.0086	0.001	2.11
LR98-1 642.1 m	< 0.1	< 0.1	< 0.1	< 0.1	0.1	< 0.001	0.16	< 3	< 1	0.1	< 0.1	0.0055	0.002	0.29
LR98-1 639.91 m	< 0.1	< 0.1	< 0.1	< 0.1	< 0.1	< 0.001	< 0.05	< 3	< 1	< 0.1	< 0.1	< 0.0005	< 0.001	0.21
LR98-1 617.90 m	< 0.1	< 0.1	< 0.1	< 0.1	< 0.1	< 0.001	< 0.05	< 3	< 1	< 0.1	< 0.1	< 0.0005	< 0.001	0.27
LR98-1 675.97 m	< 0.1	< 0.1	< 0.1	< 0.1	< 0.1	< 0.001	< 0.05	< 3	< 1	< 0.1	< 0.1	0.0015	< 0.001	1.75
LR98-1 682.75 m	< 0.1	< 0.1	< 0.1	< 0.1	< 0.1	< 0.001	< 0.05	< 3	< 1	< 0.1	< 0.1	0.0016	< 0.001	2.08
LR98-1 601.62 m	< 0.1	< 0.1	< 0.1	< 0.1	< 0.1	< 0.001	< 0.05	< 3	< 1	< 0.1	< 0.1	0.0015	< 0.001	0.11
LR98-1 512.08 m	< 0.1	< 0.1	< 0.1	< 0.1	< 0.1	< 0.001	< 0.05	< 3	< 1	0.1	< 0.1	0.0061	0.002	0.41
LR98-1 505.20 m	< 0.1	0.3	< 0.1	0.1	0.2	< 0.001	0.07	< 3	2	1.0	0.4	0.0390	0.009	0.54

Analyte Symbol	Li	Na	Mg	Al	K	Ca	Cd	V	Cr	Mn	Fe	Hf	Ni	Er	Be	Ho	Ag	Cs	Co	Eu	Bi	Se	Zn
Unit Symbol	ppm	%	%	%	%	%	ppm	ppm	ppm	ppm	%	ppm	ppm	ppm	ppm	ppm	ppm	ppm	ppm	ppm	ppm	ppm	ppm
Lower Limit	1	0.01	0.01	0.01	0.01	0.01	0.3	1	1	1	0.01	0.1	1	0.1	0.1	0.1	0.3	0.05	1	0.05	0.02	0.1	1
Method Code	TD-ICP	TD-MS	TD-MS	TD-ICP	TD-MS	TD-MS	TD-ICP	TD-MS	TD-MS	TD-MS	TD-MS	TD-MS	TD-ICP	TD-MS	TD-MS	TD-MS	TD-ICP	TD-MS	TD-ICP	TD-MS	TD-MS	TD-MS	TD-ICP
OREAS 101b (4 Acid) Meas			1.16		2.24			64		889	8.66		< 1	14.2		4.9			47	7.88			
OREAS 101b (4 Acid) Cert			1.23		2.36			77		927	10.7		8.2	15		5.2			45	8.1			
OREAS 101b (4 Acid) Meas													5						43				
OREAS 101b (4 Acid) Cert													8.2						45				
OREAS 903 (4 Acid) Meas	19	0.04	0.73	6.23	3.54	0.64	< 0.3	75	83	699	4.21	4.4	55		4.8		1.2	3.62	148		8.23	6.1	26
OREAS 903 (4 Acid) Cert	18.3	0.0300	0.714	5.89	3.31	0.625	0.200	74.0	73.0	690	4.16	4.56	54.0		4.42		0.432	3.57	131		8.90	6.06	24.3
Oreas 523 (4 Acid) Meas	17			2.62									51				2.8		656				36
Oreas 523 (4 Acid) Cert	15.5			3.02									68.0				2.61		728				40.2
Oreas 72b (4 Acid) Meas	37	1.04	9.86	5.15	1.14	2.90	< 0.3	75	462	1010	6.87	2.5	7210		0.9		0.8	3.26	142		0.67		97
Oreas 72b (4 Acid) Cert	33.3	1.01	9.59	4.79	1.14	2.79	0.310	73.6	771	1010	6.84	2.51	6860		1.02		0.230	3.37	131		0.680		99.0
OREAS 683 (4 Acid) Meas	8	1.05	8.93	7.29	0.51	5.72	< 0.3	201	> 5000	1270	8.12	0.9	1190	0.9	0.6	0.3	< 0.3	1.32	78	0.63	0.17		90
OREAS 683 (4 Acid) Cert	6.51	1.03	8.63	7.15	0.507	5.23	0.072	187	7710	1200	7.32	0.75	1180	0.93	0.56	0.32	0.172	1.32	85	0.58	0.16		92
OREAS 505 (4-Acid) Meas	49			7.98			< 0.3						15				2.3		9				86
OREAS 505 (4-Acid) Cert	46.5			7.45			0.30						16.5				1.53		8.39				88
OREAS 505 (4-Acid) Meas	40			7.49			< 0.3						17				1.8		8				89
OREAS 505 (4-Acid) Cert	46.5			7.45			0.30						16.5				1.53		8.39				88
OREAS 681 (4 Acid) Meas	13			7.65									487				0.4		45				84
OREAS 681 (4 Acid) Cert	13.0			7.91									503				0.118		51.0				88.0
OREAS 247 (4 Acid) Meas	31	0.49	1.22	5.81	2.52	0.82	< 0.3	83	80	355	3.32	3.5	42	1.4	2.3	0.5	2.5	8.73	12	0.96	0.58		77
OREAS 247 (4 Acid) Cert	31.8	0.499	1.22	6.08	2.45	0.826	0.0650	82.0	97.0	360	3.32	3.57	45.9	1.49	2.23	0.540	2.16	8.49	12.0	0.960	0.580		86.0
OREAS 247 (4 Acid) Meas	29			5.96			< 0.3						47				2.3		11				85
OREAS 247 (4 Acid) Cert	31.8			6.08			0.0650						45.9				2.16		12.0				86.0
OREAS 601c (4 acid) Meas	30	1.91	0.16	7.68	2.73	0.90	2.2	15	16	222	2.29	4.8	5	0.9	2.5	0.4	55.2	5.13	5	1.06	19.9	8.0	421
OREAS 601c (4 acid) Cert	26.7	1.94	0.169	7.06	2.72	0.953	2.77	15.5	17.5	230.000	2.41	4.93	6.83	0.78	2.46	0.38	50.3	5.17	4.99	1.08	21.1	8.75	425
OREAS 601c (4 acid) Meas		1.94	0.17		2.69	0.91		15	16	220	2.33	4.7		0.8	2.6	0.4		5.14		1.06	19.1	8.3	
OREAS 601c (4 acid) Cert		1.94	0.169		2.72	0.953		15.5	17.5	230.000	2.41	4.93		0.78	2.46	0.38		5.17		1.08	21.1	8.75	
OREAS 601c (4 acid) Meas		1.92	0.17		2.70	0.93		15	15	228	2.47	4.9		0.9	2.3	0.4		5.22		1.09	20.9	8.9	
OREAS 601c (4 acid) Cert		1.94	0.169		2.72	0.953		15.5	17.5	230.000	2.41	4.93		0.78	2.46	0.38		5.17		1.08	21.1	8.75	
OREAS 931 (4 Acid) Meas	30	0.20	1.49	6.62		0.47		76	56	982	11.3		30		1.7		15.4		53		188	47.0	489

Analyte Symbol	Li	Na	Mg	Al	K	Ca	Cd	V	Cr	Mn	Fe	Hf	Ni	Er	Be	Ho	Ag	Cs	Co	Eu	Bi	Se	Zn
Unit Symbol	ppm	%	%	%	%	%	ppm	ppm	ppm	ppm	%	ppm	ppm	ppm	ppm	ppm	ppm	ppm	ppm	ppm	ppm	ppm	ppm
Lower Limit	1	0.01	0.01	0.01	0.01	0.01	0.3	1	1	1	0.01	0.1	1	0.1	0.1	0.1	0.3	0.05	1	0.05	0.02	0.1	1
Method Code	TD-ICP	TD-MS	TD-MS	TD-ICP	TD-MS	TD-MS	TD-ICP	TD-MS	TD-MS	TD-MS	TD-MS	TD-MS	TD-ICP	TD-MS	TD-MS	TD-MS	TD-ICP	TD-MS	TD-ICP	TD-MS	TD-MS	TD-MS	TD-ICP
OREAS 931 (4 Acid) Cert	24.0	0.201	1.50	5.96		0.453		74.0	58.0	950	11.3		28.8		1.99		14.0		46.9		204	43.5	480
OREAS 504d (4 Acid) Meas	42	2.04	0.94	7.37	2.75	1.92	0.6		32	363	4.48	1.7	33	1.4	2.6	0.5	2.9	7.37	22	1.12	3.21	12.7	428
OREAS 504d (4 Acid) Cert	39.5	2.05	0.980	7.36	2.97	2.01	1.30		41.1	380	4.57	1.86	35.3	1.45	2.07	0.550	2.69	7.59	21.5	1.16	3.16	11.8	446
OREAS 750 (4 Acid) Meas	2490			5.89			< 0.3						10						4				63
OREAS 750 (4 Acid) Cert	2320.000			5.42			0.58						11.4						3.99				65
OREAS 625 (4 Acid) Meas	19	2.02	0.27	6.55	2.96	1.14	81.8	11	8	4510	4.01	6.5	< 1	1.3	2.8	0.6	13.0	5.99	5	1.32	8.33		> 10000
OREAS 625 (4 Acid) Cert	17.9	1.91	0.261	6.68	2.86	1.14	83	11.5	13.3	4330.000	3.98	6.36	5.98	1.24	2.57	0.54	11.7	5.94	4.21	1.36	8.61		31700.00
OREAS 611b (4 Acid) Meas	37	2.05	0.10	6.35	2.32	0.79	12.1	12	18	234	3.38	5.3	< 1	1.0	2.5	0.5	76.7	5.16	8	0.99	128	18.9	2610
OREAS 611b (4 Acid) Cert	36.0	1.99	0.101	6.38	2.39	0.825	12.8	12.4	22.0	240.000	3.48	5.33	10.0	1.01	2.42	0.44	76.1	5.12	7.32	1.02	138	19.0	2803
OREAS 611b (4 Acid) Meas	36			6.34			12.0						8				77.3		8				2660
OREAS 611b (4 Acid) Cert	36.0			6.38			12.8						10.0				76.1		7.32				2803
OREAS 611b (4 Acid) Meas	31			6.04			11.8						10				70.6		6				2820
OREAS 611b (4 Acid) Cert	36.0			6.38			12.8						10.0				76.1		7.32				2803
OREAS 503e (4 Acid) Meas	47	1.97	0.87	7.40	2.67	1.82	< 0.3	77	33	415	4.01	1.7	44	1.4	2.4	0.6	1.6	8.85	17	1.26	2.29	5.6	240
OREAS 503e (4 Acid) Cert	46.3	2.01	0.917	7.55	2.95	1.95	0.75	79	45.9	430.000	4.11	1.83	47.6	1.44	2.34	0.58	1.52	9.33	16.3	1.29	1.86	6.03	261
OREAS 520c (4 Acid) Meas	20			6.12			< 0.3						37				1.6		121				467
OREAS 520c (4 Acid) Cert	18.3			6.22			0.36						45.9				0.761		121				496
OREAS 520c (4 Acid) Meas	20			6.47			< 0.3						44				1.6		124				485
OREAS 520c (4 Acid) Cert	18.3			6.22			0.36						45.9				0.761		121				496
LR98-1 675.97 m Orig	1	> 3.00	< 0.01	0.03	0.04	2.71	< 0.3	< 1	1	4	0.01	< 0.1	< 1	< 0.1	< 0.1	< 0.1	< 0.3	< 0.05	< 1	< 0.05	< 0.02	0.2	1
LR98-1 675.97 m Dup	1	> 3.00	< 0.01	0.03	0.04	2.58	< 0.3	< 1	1	3	0.01	< 0.1	< 1	< 0.1	< 0.1	< 0.1	< 0.3	< 0.05	< 1	< 0.05	< 0.02	< 0.1	< 1
Method Blank		< 0.01	< 0.01		< 0.01	< 0.01		< 1	1	< 1	< 0.01	< 0.1		< 0.1	< 0.1	< 0.1		< 0.05		< 0.05	0.04	0.1	
Method Blank		< 0.01	< 0.01		< 0.01	< 0.01		< 1	1	< 1	< 0.01	< 0.1		< 0.1	< 0.1	< 0.1		< 0.05		< 0.05	< 0.02	< 0.1	
Method Blank		< 0.01	< 0.01		< 0.01	< 0.01		< 1	1	< 1	< 0.01	< 0.1		< 0.1	< 0.1	< 0.1		< 0.05		< 0.05	< 0.02	0.1	
Method Blank		< 0.01	< 0.01		< 0.01	< 0.01		< 1	< 1	< 1	< 0.01	< 0.1		< 0.1	< 0.1	< 0.1		< 0.05		< 0.05	< 0.02	< 0.1	
Method Blank	< 1			< 0.01			< 0.3						< 1				< 0.3		< 1				< 1
Method Blank	< 1			< 0.01			< 0.3						< 1				< 0.3		< 1				< 1
Method Blank	< 1			< 0.01			< 0.3						< 1				< 0.3		< 1				< 1
Method Blank	< 1			< 0.01			< 0.3						< 1				< 0.3		< 1				< 1
Method Blank	< 1			< 0.01			< 0.3						< 1				< 0.3		< 1				< 1
Method Blank	< 1			< 0.01			< 0.3						< 1				< 0.3		< 1				< 1
Method Blank	< 1			< 0.01			< 0.3						< 1				< 0.3		< 1				< 1
Method Blank	< 1			< 0.01			< 0.3						< 1				< 0.3		< 1				< 1
Method Blank	< 1			< 0.01			< 0.3						< 1				< 0.3		< 1				1

Analyte Symbol	Ga	As	Rb	Y	Sr	Zr	Nb	Mo	In	Sn	Sb	Te	Ba	La	Ce	Pr	Nd	Sm	Gd	Tb	Dy	Cu	Ge
Unit Symbol	ppm	ppm	ppm	ppm	ppm	ppm	ppm	ppm	ppm	ppm	ppm	ppm	ppm	ppm	ppm	ppm	ppm	ppm	ppm	ppm	ppm	ppm	ppm
Lower Limit	0.1	0.1	0.2	0.1	0.2	1	0.1	1	0.1	1	0.1	0.1	1	0.1	0.1	0.1	0.1	0.1	0.1	0.1	0.1	1	0.1
Method Code	TD-MS	TD-MS	TD-MS	TD-MS	TD-MS	TD-MS	TD-MS	TD-ICP	TD-MS	TD-MS	TD-MS	TD-MS	TD-MS	TD-MS	TD-MS	TD-MS	TD-MS	TD-MS	TD-MS	TD-MS	TD-MS	TD-ICP	TD-MS
OREAS 101b (4 Acid) Meas				129				18						809	1530	124	363	46.4	33.4	4.9	25.3	394	
OREAS 101b (4 Acid) Cert				133				20.1						754	1325	127	388	48	40	5.4	27	412	
OREAS 101b (4 Acid) Meas								14														371	
OREAS 101b (4 Acid) Cert								20.1														412	
OREAS 903 (4 Acid) Meas	15.5	52.2	147	22.4	76.9	161		4	0.1	3	1.7		201	40.4	81.9					0.8		6690	
OREAS 903 (4 Acid) Cert	15.0	49.7	137	22.5	77.0	152		4.32	0.160	2.63	1.57		197	40.0	82.0					0.830		6520	
Oreas 523 (4 Acid) Meas								300														> 10000	
Oreas 523 (4 Acid) Cert								313														17200	
Oreas 72b (4 Acid) Meas	12.1	152	47.5	12.4	65.8	88	5.5	3	< 0.1	1	0.9	< 0.1	346	25.0	44.3					0.4		226	
Oreas 72b (4 Acid) Cert	11.7	146	50.8	12.8	63.8	88.0	5.50	4.01	0.0490	1.43	0.870	0.0920	330	24.4	43.6					0.440		222	
OREAS 683 (4 Acid) Meas	14.3		27.7	8.6	285	31	2.8	< 1	< 0.1	1			206	8.1	17.5	2.2	8.7	1.9	1.7	0.3	1.6	408	
OREAS 683 (4 Acid) Cert	13.8		26.8	8.02	276	26	2.61	1.00	0.028	0.85			188	8.17	17.1	2.2	8.75	1.86	1.64	0.25	1.54	404	
OREAS 505 (4-Acid) Meas								70														3180	
OREAS 505 (4-Acid) Cert								66														3210	
OREAS 505 (4-Acid) Meas								64														3200	
OREAS 505 (4-Acid) Cert								66														3210	
OREAS 681 (4 Acid) Meas								< 1														252	
OREAS 681 (4 Acid) Cert								1.38														264	
OREAS 247 (4 Acid) Meas	16.4	3600	149	12.3	102	116	11.4	1	< 0.1	3	> 500		555	33.4	68.0	8.1	30.8	5.6	4.1	0.5	2.5	38	
OREAS 247 (4 Acid) Cert	16.3	3510	144	13.1	96.0	125	11.7	1.76	0.0580	3.31	3300		550	33.1	67.0	7.90	29.3	5.50	42.3	0.530	2.73	42.2	
OREAS 247 (4 Acid) Meas								2														40	
OREAS 247 (4 Acid) Cert								1.76														42.2	
OREAS 601c (4 acid) Meas	22.4	364	114	11.8	223	175	14.9	3	0.5	4	34.3	7.3		34.8	71.2	8.3	29.8	5.7	4.6	0.6	2.6	1170	1.1
OREAS 601c (4 acid) Cert	23.5	390.000	115	11.5	230	178	14.7	3.66	0.56	4.23	37.2	7.50		37.1	75	8.41	29.8	5.76	4.65	0.55	2.66	1160.000	0.18
OREAS 601c (4 acid) Meas	23.0	394	109	11.6	223	178	14.2		0.5	4	34.3	7.3		34.8	71.8	8.2	30.7	5.9	4.7	0.6	2.9		0.3
OREAS 601c (4 acid) Cert	23.5	390.000	115	11.5	230	178	14.7		0.56	4.23	37.2	7.50		37.1	75	8.41	29.8	5.76	4.65	0.55	2.66		0.18
OREAS 601c (4 acid) Meas	23.1	361	112	12.1	227	176	15.2		0.6	4	36.7	7.2		36.9	75.5	8.8	30.7	6.1	5.2	0.6	2.9		0.9
OREAS 601c (4 acid) Cert	23.5	390.000	115	11.5	230	178	14.7		0.56	4.23	37.2	7.50		37.1	75	8.41	29.8	5.76	4.65	0.55	2.66		0.18
OREAS 931 (4 Acid) Meas		6.5		21.0	34.5		11.2			43	1.9			37.4								> 10000	

Analyte Symbol	Ga	As	Rb	Y	Sr	Zr	Nb	Mo	In	Sn	Sb	Te	Ba	La	Ce	Pr	Nd	Sm	Gd	Tb	Dy	Cu	Ge
Unit Symbol	ppm	ppm	ppm	ppm	ppm	ppm	ppm	ppm	ppm	ppm	ppm	ppm	ppm	ppm	ppm	ppm	ppm	ppm	ppm	ppm	ppm	ppm	ppm
Lower Limit	0.1	0.1	0.2	0.1	0.2	1	0.1	1	0.1	1	0.1	0.1	1	0.1	0.1	0.1	0.1	0.1	0.1	0.1	0.1	1	0.1
Method Code	TD-MS	TD-MS	TD-MS	TD-MS	TD-MS	TD-MS	TD-MS	TD-ICP	TD-MS	TD-MS	TD-MS	TD-MS	TD-MS	TD-MS	TD-MS	TD-MS	TD-MS	TD-MS	TD-MS	TD-MS	TD-MS	TD-ICP	TD-MS
OREAS 931 (4 Acid) Cert		11.6		19.6	34.5		11.0			42.1	1.70			34.0								38200	
OREAS 504d (4 Acid) Meas	17.9	52.3	129	14.5	274	59	10.2	525	0.7	4	4.5	1.5	746	27.1	55.4	6.5	26.1	5.4	4.8	0.6	3.3	> 10000	
OREAS 504d (4 Acid) Cert	18.1	49.6	127	14.2	279	59.0	9.77	507	0.730	4.22	5.52	1.61	816	27.6	55.0	6.63	26.8	5.38	4.77	0.620	3.23	11000	
OREAS 750 (4 Acid) Meas								2														20	
OREAS 750 (4 Acid) Cert								2.17														20.4	
OREAS 625 (4 Acid) Meas	21.0	135	140	15.9	134	235	14.5	11	2.0	4	35.3	< 0.1		37.3	78.4	9.5	37.2	7.4	5.9	0.7	3.5	1630	
OREAS 625 (4 Acid) Cert	20.6	143	135	14.9	130	229	14.8	12.3	2.12	3.57	35.1	0.063		34.7	72	9.74	37.3	6.80	5.46	0.72	3.52	1710.000	
OREAS 611b (4 Acid) Meas	22.1	2040	104	13.1	200	193	14.7	23	2.8	15	311	25.5		34.6	72.9	8.5	30.2	5.8	4.7	0.6	3.1	8730	0.5
OREAS 611b (4 Acid) Cert	22.9	1928	109	13.0	201	198	15.0	27.2	2.69	15.3	345	26.3		34.8	72	8.23	31.2	5.85	4.59	0.63	3.06	9140.000	0.24
OREAS 611b (4 Acid) Meas								28														8630	
OREAS 611b (4 Acid) Cert								27.2														9140.000	
OREAS 611b (4 Acid) Meas								26														8640	
OREAS 611b (4 Acid) Cert								27.2														9140.000	
OREAS 503e (4 Acid) Meas	19.1	31.8	147	14.8	224	56	11.3	350	0.4	4	201	0.8	877	31.9	66.2	7.7	30.0	6.1	5.5	0.7	3.5	4950	0.2
OREAS 503e (4 Acid) Cert	19.4	28.8	146	14.9	229	57	11.0	343	0.38	4.51	236	0.85	926	33.3	67	7.83	31.0	6.21	5.50	0.69	3.50	5310.000	0.18
OREAS 520c (4 Acid) Meas								44														2700	
OREAS 520c (4 Acid) Cert								45.3														2910.000	
OREAS 520c (4 Acid) Meas								43														2880	
OREAS 520c (4 Acid) Cert								45.3														2910.000	
LR98-1 675.97 m Orig	0.1	< 0.1	0.7	0.1	114	1	< 0.1	< 1	< 0.1	< 1	0.5	< 0.1	2	0.2	0.3	< 0.1	0.2	< 0.1	< 0.1	< 0.1	< 0.1	< 1	< 0.1
LR98-1 675.97 m Dup	< 0.1	0.1	0.6	0.1	109	< 1	< 0.1	< 1	< 0.1	< 1	0.5	< 0.1	2	0.1	0.2	< 0.1	0.1	< 0.1	< 0.1	< 0.1	< 0.1	< 1	< 0.1
Method Blank	< 0.1	< 0.1	< 0.2	< 0.1	< 0.2	< 1	< 0.1		< 0.1	< 1	< 0.1	< 0.1	< 1	< 0.1	< 0.1	< 0.1	< 0.1	< 0.1	< 0.1	< 0.1	< 0.1		< 0.1
Method Blank	< 0.1	< 0.1	< 0.2	< 0.1	< 0.2	< 1	< 0.1		< 0.1	< 1	0.3	< 0.1	< 1	< 0.1	< 0.1	< 0.1	< 0.1	< 0.1	< 0.1	< 0.1	< 0.1		< 0.1
Method Blank	< 0.1	< 0.1	< 0.2	< 0.1	< 0.2	< 1	< 0.1		< 0.1	< 1	0.4	< 0.1	< 1	< 0.1	< 0.1	< 0.1	< 0.1	< 0.1	< 0.1	< 0.1	< 0.1		< 0.1
Method Blank	< 0.1	< 0.1	0.4	< 0.1	0.2	1	< 0.1		< 0.1	< 1	< 0.1	< 0.1	1	0.1	0.2	< 0.1	< 0.1	< 0.1	< 0.1	< 0.1	< 0.1		< 0.1
Method Blank								< 1														< 1	
Method Blank								< 1														< 1	
Method Blank								< 1														< 1	
Method Blank								< 1														< 1	
Method Blank								< 1														< 1	
Method Blank								< 1														< 1	
Method Blank								< 1														< 1	
Method Blank								< 1														< 1	
Method Blank								< 1														< 1	

Analyte Symbol	Tm	Yb	Lu	Ta	W	Re	Tl	Pb	Sc	Th	U	Ti	P	S
Unit Symbol	ppm	ppm	ppm	ppm	ppm	ppm	ppm	ppm	ppm	ppm	ppm	%	%	%
Lower Limit	0.1	0.1	0.1	0.1	0.1	0.001	0.05	3	1	0.1	0.1	0.0005	0.001	0.01
Method Code	TD-MS	TD-MS	TD-MS	TD-MS	TD-MS	TD-MS	TD-MS	TD-ICP	TD-ICP	TD-MS	TD-MS	TD-ICP	TD-ICP	TD-ICP
OREAS 101b (4 Acid) Meas	2.0	13.4	1.7					31		34.9	409	0.339	0.114	
OREAS 101b (4 Acid) Cert	2.08	13.9	1.96					23		36.4	387	0.35	0.1118	
OREAS 101b (4 Acid) Meas								21				0.258	0.112	
OREAS 101b (4 Acid) Cert								23				0.35	0.1118	
OREAS 903 (4 Acid) Meas		2.5	0.4	0.5			0.61	12	10	12.7	7.6	0.174	0.117	0.48
OREAS 903 (4 Acid) Cert		2.36	0.360	0.540			0.620	11.3	10.2	13.6	7.58	0.192	0.107	0.500
Oreas 523 (4 Acid) Meas								30	6			0.219	0.081	3.17
Oreas 523 (4 Acid) Cert								26.3	7.42			0.283	0.0920	3.82
Oreas 72b (4 Acid) Meas				0.4	4.3		0.35	15	12	10.5	4.6	0.215	0.027	1.46
Oreas 72b (4 Acid) Cert				0.430	4.00		0.350	14.9	12.8	11.3	4.68	0.216	0.0260	1.49
OREAS 683 (4 Acid) Meas	0.1	0.8	0.2		1.2			9	18	2.9	0.6	0.246	0.049	0.20
OREAS 683 (4 Acid) Cert	0.13	0.88	0.13		1.23			10.2	19.7	2.42	0.58	0.263	0.050	0.205
OREAS 505 (4-Acid) Meas								28	8			0.317	0.088	0.40
OREAS 505 (4-Acid) Cert								26.7	8.81			0.340	0.086	0.446
OREAS 505 (4-Acid) Meas								27	8			0.329	0.088	0.42
OREAS 505 (4-Acid) Cert								26.7	8.81			0.340	0.086	0.446
OREAS 681 (4 Acid) Meas								8	25			0.546	0.140	0.11
OREAS 681 (4 Acid) Cert								10.2	27.7			0.588	0.141	0.109
OREAS 247 (4 Acid) Meas	0.2	1.3	0.2	0.9	8.0		0.81	29	10	12.8	2.5	0.359	0.047	0.63
OREAS 247 (4 Acid) Cert	0.230	1.54	0.240	0.920	7.88		0.800	31.9	11.4	12.6	2.53	0.390	0.0480	0.714
OREAS 247 (4 Acid) Meas								29	11			0.350	0.050	0.71
OREAS 247 (4 Acid) Cert								31.9	11.4			0.390	0.0480	0.714
OREAS 601c (4 acid) Meas	< 0.1	0.6	< 0.1	1.1	4.3		1.72	320	4	12.3	4.2	0.131	0.040	1.54
OREAS 601c (4 acid) Cert	0.094	0.54	0.077	1.11	4.67		1.75	328	4.01	12.4	4.40	0.135	0.039	1.58
OREAS 601c (4 acid) Meas	0.1	0.6	< 0.1	1.1	4.4		1.73			12.0	4.4			
OREAS 601c (4 acid) Cert	0.094	0.54	0.077	1.11	4.67		1.75			12.4	4.40			
OREAS 601c (4 acid) Meas	0.1	0.6	< 0.1	1.1	4.6		1.81			12.5	4.4			
OREAS 601c (4 acid) Cert	0.094	0.54	0.077	1.11	4.67		1.75			12.4	4.40			
OREAS 931 (4 Acid) Meas					19.5			157		12.8		0.308	0.056	3.62

Analyte Symbol	Tm	Yb	Lu	Ta	W	Re	Tl	Pb	Sc	Th	U	Ti	P	S
Unit Symbol	ppm	ppm	ppm	ppm	ppm	ppm	ppm	ppm	ppm	ppm	ppm	%	%	%
Lower Limit	0.1	0.1	0.1	0.1	0.1	0.001	0.05	3	1	0.1	0.1	0.0005	0.001	0.01
Method Code	TD-MS	TD-MS	TD-MS	TD-MS	TD-MS	TD-MS	TD-MS	TD-ICP	TD-ICP	TD-MS	TD-MS	TD-ICP	TD-ICP	TD-ICP
OREAS 931 (4 Acid) Cert					19.8			147		12.3		0.294	0.0510	4.12
OREAS 504d (4 Acid) Meas	0.2	1.2	0.2	0.8	8.7	0.108	0.77	109	10	11.3	3.2	0.296	0.085	1.52
OREAS 504d (4 Acid) Cert	0.200	1.23	0.200	0.840	8.79	0.110	0.720	116	9.74	10.0	2.94	0.326	0.0870	1.72
OREAS 750 (4 Acid) Meas								13	4			0.172	0.076	0.08
OREAS 750 (4 Acid) Cert								13.8	3.72			0.158	0.070	0.073
OREAS 625 (4 Acid) Meas	0.2	1.0	0.1	1.1	3.7	0.004	10.0	> 5000	3	13.8	5.8	0.115	0.028	3.40
OREAS 625 (4 Acid) Cert	0.17	0.96	0.14	1.11	3.99	0.004	9.79	8220.000	4.60	12.7	5.52	0.120	0.030	3.80
OREAS 611b (4 Acid) Meas	0.1	0.6	< 0.1	1.0	4.7	0.012	1.50	982	3	11.8	4.6	0.104	0.028	2.39
OREAS 611b (4 Acid) Cert	0.12	0.69	0.092	1.10	4.65	0.013	1.53	970	3.66	11.5	4.69	0.113	0.029	2.67
OREAS 611b (4 Acid) Meas								915	3			0.100	0.029	2.46
OREAS 611b (4 Acid) Cert								970	3.66			0.113	0.029	2.67
OREAS 611b (4 Acid) Meas								915	3			0.103	0.029	2.61
OREAS 611b (4 Acid) Cert								970	3.66			0.113	0.029	2.67
OREAS 503e (4 Acid) Meas	0.2	1.2	0.2	1.0	10.0	0.017	0.86	80	9	13.4	4.3	0.304	0.085	0.76
OREAS 503e (4 Acid) Cert	0.20	1.18	0.19	0.97	10.6	0.016	0.82	78	9.66	12.3	3.47	0.351	0.088	0.875
OREAS 520c (4 Acid) Meas								130	7			0.291	0.065	0.50
OREAS 520c (4 Acid) Cert								127	8.49			0.312	0.069	0.575
OREAS 520c (4 Acid) Meas								123	8			0.280	0.071	0.52
OREAS 520c (4 Acid) Cert								127	8.49			0.312	0.069	0.575
LR98-1 675.97 m Orig	< 0.1	< 0.1	< 0.1	< 0.1	< 0.1	< 0.001	< 0.05	< 3	< 1	< 0.1	< 0.1	0.0014	< 0.001	1.79
LR98-1 675.97 m Dup	< 0.1	< 0.1	< 0.1	< 0.1	< 0.1	< 0.001	< 0.05	< 3	< 1	< 0.1	< 0.1	0.0017	< 0.001	1.70
Method Blank	< 0.1	< 0.1	< 0.1	< 0.1	< 0.1	< 0.001	< 0.05			< 0.1	< 0.1			
Method Blank	< 0.1	< 0.1	< 0.1	< 0.1	< 0.1	< 0.001	< 0.05			< 0.1	< 0.1			
Method Blank	< 0.1	< 0.1	< 0.1	< 0.1	< 0.1	< 0.001	< 0.05			< 0.1	< 0.1			
Method Blank	< 0.1	< 0.1	< 0.1	< 0.1	< 0.1	< 0.001	< 0.05			< 0.1	< 0.1			
Method Blank								< 3	< 1			< 0.0005	< 0.001	< 0.01
Method Blank								< 3	< 1			< 0.0005	< 0.001	< 0.01
Method Blank								< 3	< 1			< 0.0005	< 0.001	< 0.01
Method Blank								< 3	< 1			< 0.0005	< 0.001	< 0.01
Method Blank								< 3	< 1			< 0.0005	< 0.001	< 0.01
Method Blank								< 3	< 1			< 0.0005	< 0.001	< 0.01

Analyte Symbol	Tm	Yb	Lu	Ta	W	Re	Tl	Pb	Sc	Th	U	Ti	P	S
Unit Symbol	ppm	ppm	ppm	ppm	ppm	ppm	ppm	ppm	ppm	ppm	ppm	%	%	%
Lower Limit	0.1	0.1	0.1	0.1	0.1	0.001	0.05	3	1	0.1	0.1	0.0005	0.001	0.01
Method Code	TD-MS	TD-MS	TD-MS	TD-MS	TD-MS	TD-MS	TD-MS	TD-ICP	TD-ICP	TD-MS	TD-MS	TD-ICP	TD-ICP	TD-ICP
Method Blank								< 3	< 1			< 0.0005	< 0.001	< 0.01
Method Blank								< 3	< 1			< 0.0005	< 0.001	< 0.01
Method Blank								< 3	< 1			< 0.0005	< 0.001	< 0.01
